# Tunable Heteroaromatic Nitriles for Selective Bioorthogonal Click Reaction with Cysteine

**DOI:** 10.1021/acs.bioconjchem.3c00163

**Published:** 2023-06-24

**Authors:** Matic Proj, Nika Strašek, Stane Pajk, Damijan Knez, Izidor Sosič

**Affiliations:** Faculty of Pharmacy, Department of Pharmaceutical Chemistry, University of Ljubljana, Askerceva 7, Ljubljana 1000, Slovenia

## Abstract

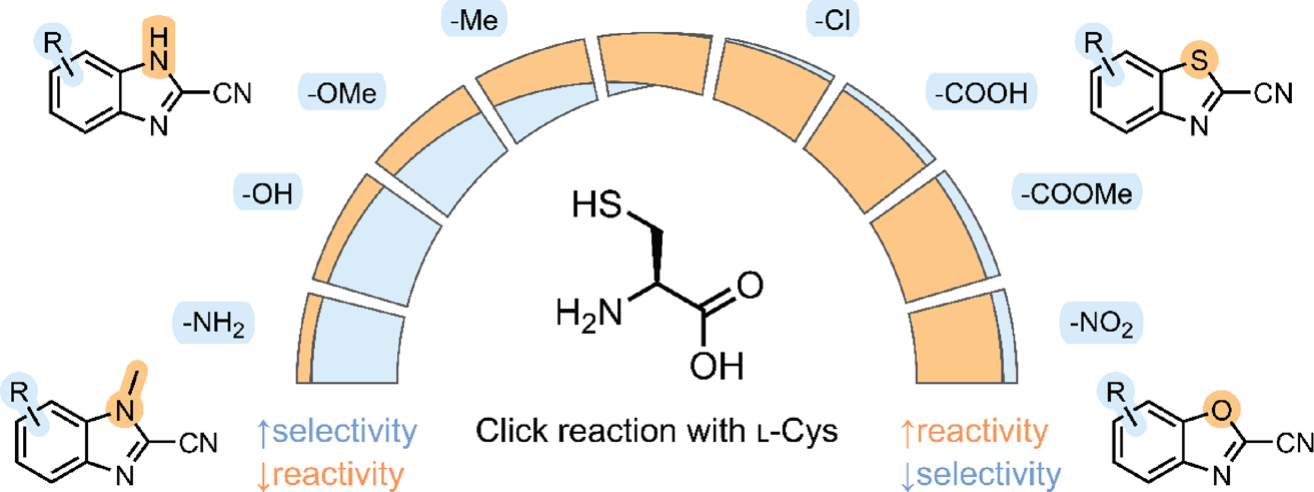

The binucleophilic properties of 1,2-aminothiol and its rare occurrence in nature make it a useful reporter for tracking molecules in living systems. The 1,2-aminothiol moiety is present in cysteine, which is a substrate for a biocompatible click reaction with heteroaromatic nitriles. Despite the wide range of applications for this reaction, the scope of nitrile substrates has been explored only to a limited extent. In this study, we expand the chemical space of heteroaromatic nitriles for bioconjugation under physiologically relevant conditions. We systematically assembled a library of 116 2-cyanobenzimidazoles, 1-methyl-2-cyanobenzimidazoles, 2-cyanobenzothiazoles, and 2-cyanobenzoxazoles containing electron-donating and electron-withdrawing substituents at all positions of the benzene ring. The compounds were evaluated for their stability, reactivity, and selectivity toward the N-terminal cysteine of model oligopeptides. In comparison to the benchmark 6-hydroxy-2-cyanobenzothiazole or 6-amino-2-cyanobenzothiazole, we provide highly selective and moderately reactive nitriles as well as highly reactive yet less selective analogs with a variety of enabling attachment chemistries to aid future applications in bioconjugation, chemical biology, and nanomaterial science.

## Introduction

The 1,2-aminothiol moiety is rarely found in nature, yet its unique property, i.e., binucleophilicity, makes it a useful functionality for manipulating molecules in complex matrices such as intracellular environments. For example, proteins containing N-terminal cysteine (Cys) can be site-specifically modified at this solvent-exposed site, and the modifications have minimal structural and functional effects.^1^ Although such proteins are rare in nature, this small tag can be readily engineered into any protein. Most commonly, proteins are expressed with a recognition sequence for a sequence-selective protease (e.g., tobacco etch virus, thrombin, or factor Xa),^2,3^ and the selective cleavage of the tag yields a protein with free N-terminal Cys. Alternatively, Met-Cys is expressed at the N-terminus, which is recognized by endogenous methionine aminopeptidases that cleave the Met residue and expose the 1,2-aminothiol motif.^4^ Recently, a more efficient and highly specific approach was developed in which a Met-Pro-Cys sequence with a short peptidase recognition motif (4–10 residues) is attached to the protein N-terminus and subjected to recombinant methionine and proline aminopeptidases.^1^ In addition, 1,2-aminothiols can be introduced into other sites in the protein sequence by using unnatural amino acids containing this particular substructure.^5^

**Figure 1:**
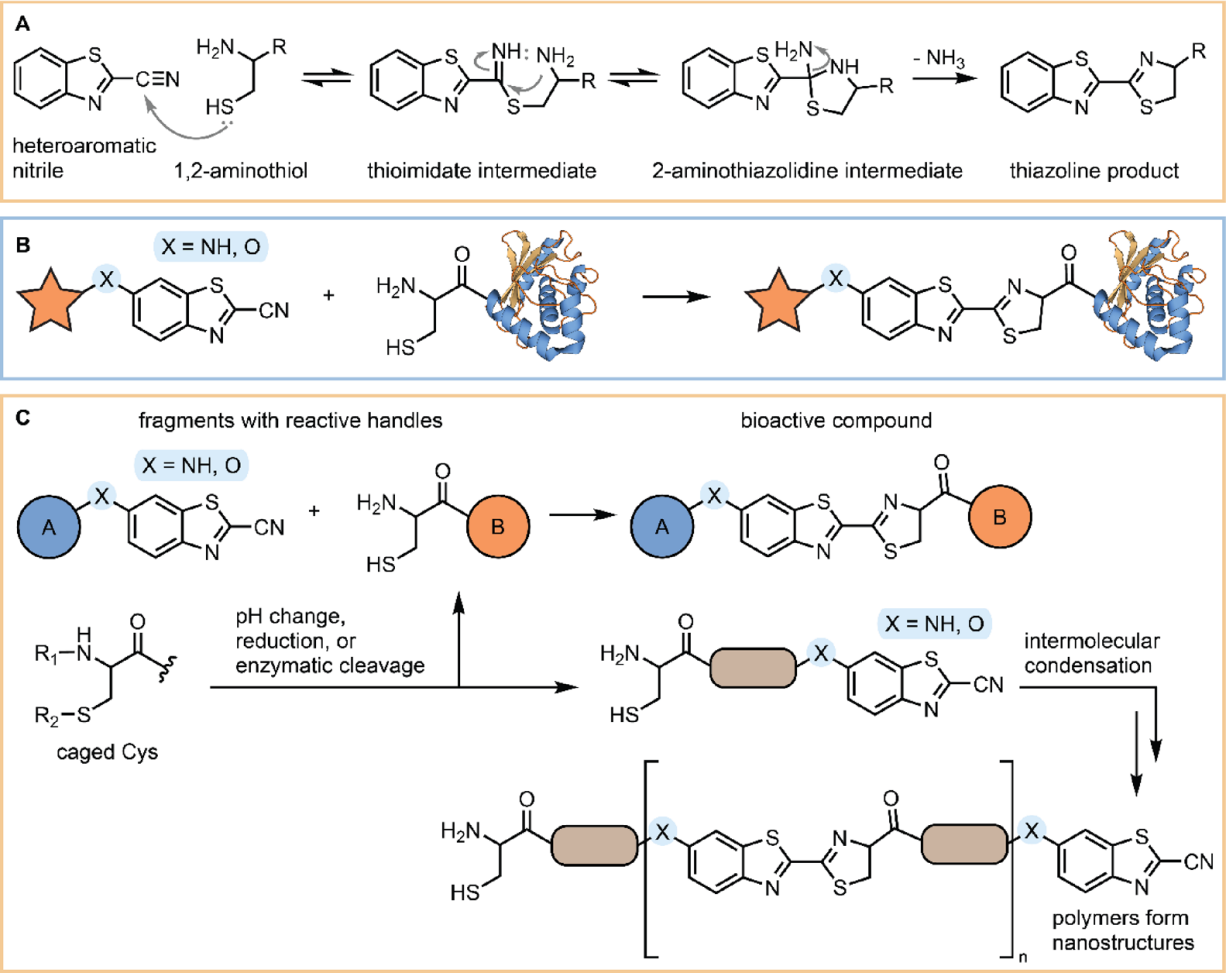
Biocompatible click reaction between heteroaromatic nitriles and 1,2-aminothiols. (A) Reaction mechanism. (B) Site-specific labeling of proteins with N-terminal Cys. (C) Controllable *in situ* assembly of bioactive compounds and nanostructures. Click reaction can be initiated by deprotection of a caged Cys by changing pH, reduction, or enzymatic cleavage.

Several electrophilic groups were developed to selectively target proteins with N-terminal Cys,^6−10^ e.g., thioesters,^11^*O*-salicylaldehyde esters,^12^ activated aldehydes,^8^ activated nitriles,^13^ 2-((alkylthio)(aryl)methylene)malononitriles,^14^*N*-hydroxysuccinimide-activated acrylamides,^15^ 2-benzylacrylaldehydes,^16^ 2-formylphenylboronic acids,^17−19^ and monosubstituted cyclopropenones.^20^ Here, we focused on activated heteroaromatic nitriles, which were found to have a wide range of applicability in recent years.^7,21−24^ Inspired by the final step in the biosynthesis of d-luciferin, Rao and co-workers utilized a click reaction between 2-cyanobenzothiazole and N-terminal Cys for protein labeling.^25^ In the first step, addition of the thiol to the nitrile leads to reversible formation of the thioimidate intermediate (Figure 1A), which is followed by an intramolecular condensation reaction with amine to form the 2-aminothiazolidine intermediate. Finally, the ammonia is irreversibly eliminated to form the final thiazoline product.^26^ The reaction is specific and proceeds in quantitative yield and under biocompatible conditions. Moreover, the kinetics is fast (second-order reaction rate constant around 10 s^–1^ M^–1^), and only 30 min is required for complete labeling when performed at low micromolar concentration. The reaction proceeds with 1,2- or 1,3-aminothiols, but not when the thiol group is replaced by a hydroxy group.^25^

A wide range of applications have been demonstrated for the reaction between heterocyclic nitriles and 1,2-aminothiols as summarized in recent reviews.^21,23,24^ To exemplify, site-specific labeling of proteins with N-terminal Cys has been exploited for imaging, drug delivery, and protein immobilization (Figure 1B). In addition to protein labeling, the same reaction is also used for the controllable *in situ* assembly of bioactive compounds and nanostructures (Figure 1C). Controlled assembly is achieved by deprotection of the caged Cys moiety by changes in the pH, reductive environment, or an enzymatic cleavage and can be applied to targeted drug delivery or building molecular imaging probes. Most recently, the reaction was exploited for intracellular assembly of enzyme-derived clicking proteolysis-targeting chimeras,^27^ preparation of chemically enhanced phage display libraries,^28^ and in-cell protein macrocyclization and protein stapling.^13^

Despite numerous possible applications, most studies resorted to the use of nitriles derived from 6-hydroxy- or 6-amino-2-cyanobenzothiazole, inspired by firefly luciferin. The scope of the nitrile substrates has been explored to a very limited extent experimentally, whereby the electron-withdrawing substituents and heteroatoms increase the rate of reaction with N-terminal Cys.^29−31^ This was also confirmed by theoretical calculations.^32,33^ Here, we investigated the scope of the heteroaromatic nitriles in click reaction with Cys under physiologically relevant conditions. In search of stable, selective, and fast-reacting heteroaromatic nitriles, we designed and synthesized a library of 116 2-cyanobenzimidazoles (CBIs), 1-methyl-2-cyanobenzimidazoles (Me-CBIs), 2-cyanobenzothiazoles (CBTs), and 2-cyanobenzoxazoles (CBOs) with a general name 2-cyanobenz’X’azoles (CBXs) that encompassed different substitution vectors and chemistries on the heteroaromatic scaffold beyond the typical luciferin structure.

## Results and Discussion

### Chemistry

**Figure 2:**
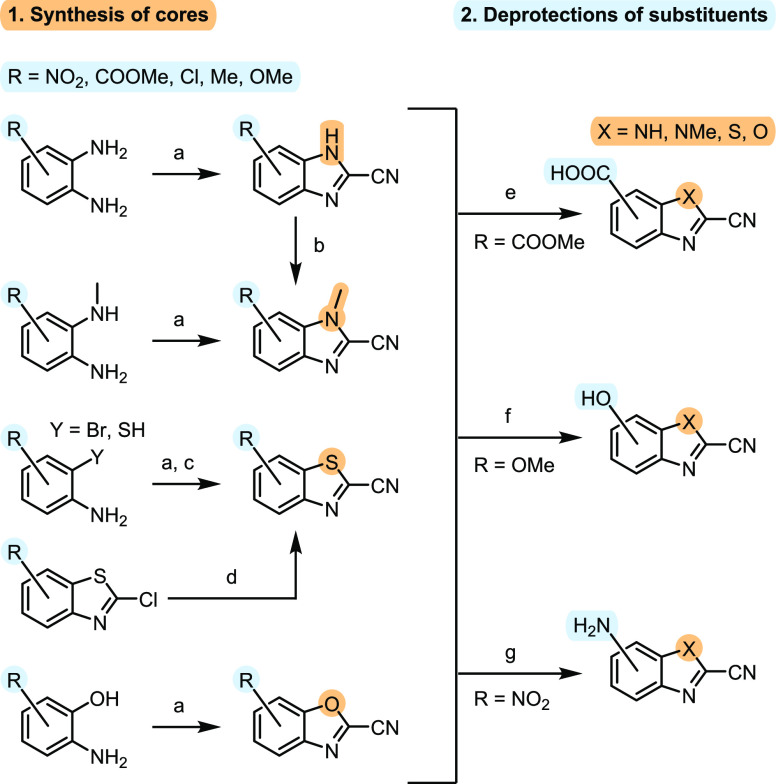
General procedures for synthesis of a library of CBXs. Reaction conditions: (a) corresponding *ortho*-substituted aniline (−NH_2_, −NHMe, −OH, and −SH), Appel’s salt (4,5-dichloro-1,2,3-dithiazolium chloride), pyridine, 70 °C, 16 h; (b) MeI, K_2_CO_3_, MeCN, rt, 72 h; (c) 1. corresponding 2-bromoaniline, Appel’s salt (4,5-dichloro-1,2,3-dithiazolium chloride), pyridine, dry DCM, rt, 2 h; 2. CuI, dry pyridine, MWI, 115 °C, 20 min; (d) NaCN, DABCO, MeCN/H_2_O, rt, 7–24 h (for **83**: KCN, DABCO, dry DMF, 100 °C, 48 h); (e) Al, I_2_, dry MeCN, 80 °C, 18 h; (f) AlCl_3_, dry DCM, 40 °C, 18 h (for **41**: Al, I_2_, dry MeCN, 80 °C, 18 h); (g) Fe, AcOH, rt, 2 h.

The four different CBX cores were functionalized with eight different substituents (i.e., nitro, methyl carboxylate, carboxylic acid, chloro, methyl, methoxy, hydroxyl, and amino) that cover a range of electron-withdrawing or electron-donating properties via resonance or inductive effects and provide a variety of chemical attachment points to aid future applications. During assembly of the library, we noticed that only a few CBXs are available commercially (16 compounds); therefore, most had to be synthesized (Figure 2, Scheme S1). Corresponding aniline derivatives were reacted with Appel’s salt (4,5-dichloro-1,2,3-dithiazolium chloride) via direct cyclization or two-step imine formation and subsequent Cu-catalyzed cyclization to obtain CBXs.^34^ Three CBTs were prepared using an alternative approach by reacting 2-chlorobenzothiazoles with potassium/sodium cyanide in the presence of DABCO. Three methyl-substituted benzoxazoles (**89**, **97**, and **113**) were unstable in solid form, as described previously.^35^ Me-CBIs were prepared by methylation of CBIs that led to two positional isomers, which were separated by column chromatography and the structures confirmed by 2D and nuclear Overhauser effect NMR experiments (for details, see the Supporting Information). Methyl esters were converted into carboxylic acid using *in situ* generated AlI_3_ under anhydrous conditions, which prevented hydrolysis of the nitrile moiety.^36^ Despite several attempts, two compounds—carboxylic acids **54** and **87**—could not be prepared with only traces detected in complex reaction mixtures. Demethylation of *O*-methyl ethers was accomplished using AlCl_3_ or *in situ* generated AlI_3_, whereas amines were prepared by reducing the nitro group in the presence of iron powder in acetic acid (Figure 2). References for compounds that were previously described or purchased from commercial vendors are provided in Table S1. The structures of all compounds and synthetic intermediates are presented in the Supporting Excel File.

### Stability and Reactivity Screening

**Figure 3:**
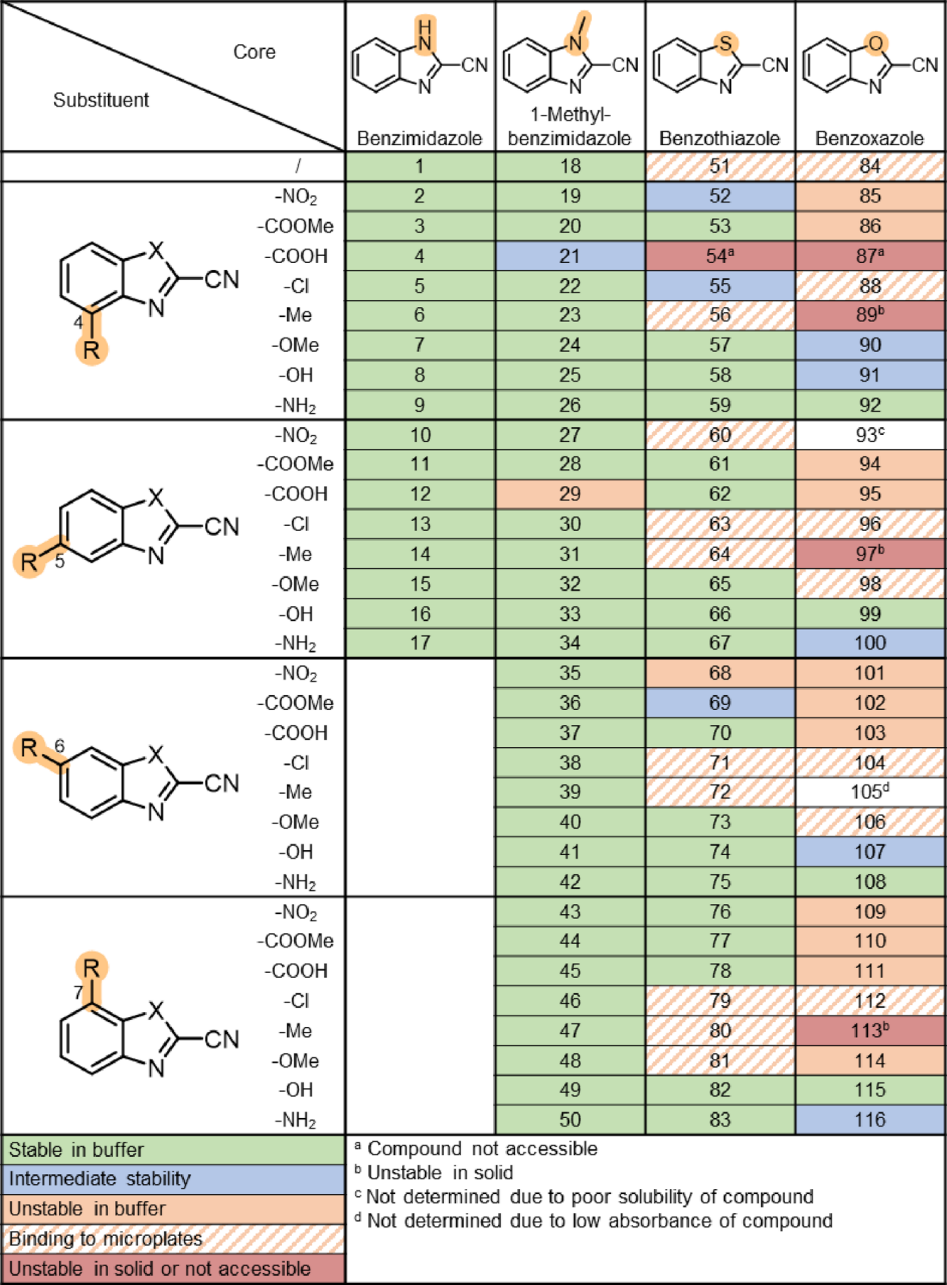
Results from the UV–vis-based stability assay. Stability in buffer was evaluated after 4 h at 37 °C. Compounds with a relative absorbance difference below 0.1, between 0.1 and 0.2, or above 0.2 were classified as stable, intermediate, or unstable, respectively. The absorbance spectra are provided in the Supporting Information, Stability and Reactivity Screening. Stable and intermediately stable compounds were evaluated further.

CBXs were first evaluated for stability in the buffer solution pH 7.4 at 37 °C using a high-throughput UV–vis-based assay, where we followed the changes in the absorption spectra of the compounds for 4 h (Figure 3).^37,38^ To achieve high throughput, the assay was performed in UV-transparent 96-well microplates, and the analysis was automated using a Python script (available at https://github.com/maticproj/UV-Vis-analysis). First, we determined the most responsive wavelengths for each compound and calculated the absolute difference in absorbance between the first time point and after 4 h. The absolute difference in absorbance was then divided by the absorbance at the first time point to obtain the relative difference in absorbance. The thresholds for stability were determined based on repeatability experiments for five compounds (Table S2), where the 10-fold standard deviation for the relative difference in absorbance ranged from 0.02 to 0.13 for three independent experiments. Therefore, compounds with a relative absorbance difference below 0.1, between 0.1 and 0.2, or above 0.2 were classified as stable, intermediate, or unstable, respectively.

For unstable compounds, we expected formation of degradation products with different absorption spectra, resulting in a clear isosbestic point when aligning spectra for the stability samples over the course of 4 h. However, for some compounds, we observed only a decrease in absorbance at all wavelengths. This effect could be due to the compound binding to the microplate walls made of an acrylic copolymer, thereby decreasing its concentration in solution.^39^ We confirmed this by incubating the samples in glass vials and then transferring them to the UV-transparent microplates just before measurement. Compounds without isosbestic points (e.g., **56** and **71**) were stable in glass vials for 4 h, whereas the concentration in the microplates decreased after 4 h (Figure S1). Compounds with isosbestic points (e.g., **85** and **101**) were unstable and formed degradation product(s) regardless of incubation in glass vials or in microplates (Figure S1). Nevertheless, binding of the compounds in aqueous solutions to the labware (e.g., microplates, pipette tips, plastic containers) is an undesirable property,^39−42^ and we thus excluded them from further evaluation.

To validate the UV–vis-based stability assay, an NMR-based stability assay was performed. Although the final compound concentration had to be higher in this experiment to obtain response (1 mM vs 50 μM in the UV–vis-based assay), this should not affect the outcomes since water was still present in excess, and pseudo-first-order conditions were warranted. The results were consistent with those obtained in the UV–vis-based assay (Figures S2–S7). For the unstable compound **94** with isosbestic point, additional peaks appeared in the aromatic proton region of the spectrum after 1 h, whereas for the stable compounds **15**, **57**, **65**, **73**, and **74**, no significant changes appeared in the spectra after 4 h.

We observed that CBIs and Me-CBIs were stable after 4 h in buffer, except for two carboxylates (Figure 3). The set of CBTs showed the greatest variability in stability, and CBOs were generally unstable. Electron-donating substituents (i.e., hydroxyl, amino, and methoxy) increased the stability of CBTs and CBOs. Of the CBTs and CBOs substituted with the strongest electron-withdrawing group (nitro), only **76** was stable. Unsubstituted CBTs, CBOs, and their most lipophilic derivatives (i.e., chloro, methyl, and some methoxy analogues) were binding to the microplate walls. This gives rise to some practical implications, namely, C-C coupling strategy with chloro (halogen) analogs and requires special attention to avoid loss of starting material in bioconjugation reactions performed in plastic glassware. An elegant solution would be functionalization of the benz’X’azole core with ligands of interest prior to nitrile bioconjugation. Nonetheless, one has to bear in mind that benz’X’azole derivatization must be selective and under mild conditions, which do not affect the nitrile moiety.

Stable and intermediately stable compounds were then evaluated for their reactivity with various thiol-, hydroxy-, and amino-containing surrogates as well as with *tris*(2-carboxyethyl)phosphine (TCEP), a common reducing agent used to stabilize protein solutions. We employed the same assay as for stability evaluation but with additional reagents, i.e., Lys, Ser, TCEP, *N*-acetyl cysteine (NAC), or Cys, in 10-fold excess over the compounds. Results were compared to blank values (compound in buffer without the reagents) and monitored for 4 h. The threshold for reactivity was a relative absorbance difference greater than 0.2. None of the assayed compounds reacted with Lys or Ser, and only two compounds (**21** and **76**) were flagged as reactive with TCEP (Figures S8–S10). Two-thirds of the assayed CBOs reacted with NAC before the first timepoint were acquired, i.e., in less than 3 min. Chloro-substituted CBT was the only NAC-reactive CBT (Figure S11). However, all assayed compounds reacted with Cys that contains an unprotected 1,2-aminothiole moiety (Figure S12). Under the assay conditions, CBTs and CBOs were mostly hyperreactive (a significant change in the spectrum when the first time point was acquired), while CBIs and their 1-methyl derivatives were moderately reactive. These results show that our set of compounds exhibits a broad range of reactivities toward Cys, which were quantitatively evaluated in the following steps.

To additionally probe for selectivity toward 1,2-aminothiole, reactivity was examined with another thiol-containing reagent. In an assay with reduced 5,5-dithio-bis-(2-nitrobenzoic acid) (DTNB), the absorbance of 5-mercapto-2-nitrobenzoic acid at 412 nm is followed when the compound is added.^43,44^ The reagent contains an aromatic thiol group (pKa = 4.53)^45^ with a pKa value lower than that of the aliphatic thiols in NAC (pKa = 9.52)^46^ or Cys (pKa = 8.30),^46^ which in turn makes it highly nucleophilic. Indeed, more compounds were reactive compared to the results with NAC (Figure S13): most CBOs were reactive, except for 5- or 6-amino derivatives (**100** and **108**, respectively). Another five CBTs were reactive (**52**, **59**, **75**, **76**, and **77**), as were two CBIs (**7** and **17**).

Next, we aimed to confirm reactivity with TCEP, since only two compounds were flagged as reactive. We used the same procedure as for oligopeptide labeling, i.e., the compounds were incubated with TCEP (5 equiv) for 30 min and analyzed by LCMS. The benchmark 6-amino-CBT (**75**) was used as a negative control, and indeed, no new peaks appeared (Figure S14). Similarly, no new peaks were detected at 280 nm for **21** (Figure S15), from which we conclude that this compound does not react with TCEP. The false-positive result of the screening was not surprising because the compound was intermediately stable in buffer and without clear isosbestic points that would indicate formation of a product with TCEP. On the other hand, an additional peak appeared for compound **76** corresponding to a compound–TCEP adduct [M + H]^+^ with *m*/*z* = 440.0676 (−0.9 ppm) (Figure S16). Similarly, we checked reactivity of benchmark 6-amino-CBT **75** with NAC (5 equiv). Although this compound was flagged as not reactive in the UV–vis-based screening assay, the **75**–NAC thioimidate adduct was detected in small amounts besides the parent compound (Figure S17). As expected, when the experiment was performed in the presence of Cys (5 equiv), no parent compound **75** was detected since all starting nitrile was consumed to form a **75**–Cys thiazoline adduct (Figure S18).

### Kinetics

Based on the results obtained in stability and reactivity screening, compounds with desirable properties were scrutinized further. Unstable compounds and compounds binding to microplates were excluded in the initial step, as were compounds that were unstable in solid form. Seven compounds were excluded based on their reactivity with NAC, and one compound reacted with TCEP. The reaction rate with Cys was then evaluated for the remaining 71 compounds. We selected Cys to allow comparison with previous results, as it was already used by others.^25,31^ To evaluate such a large set of compounds, we needed a method with higher throughput than conventional kinetic HPLC measurements. We resorted to nonchromatographic spectrophotometric measurements of the reaction mixtures at the most responsive wavelength. All measurements were performed under pseudo-first-order conditions with at least a 10-fold excess of Cys over the compound. This approach allows curve fitting without converting the absorbance data to concentrations prior to calculations.^47^ A similar method was used recently to determine the reaction rate of heteroaromatic sulfones.^48^

For compounds that were flagged as reactive in the UV–vis-based screening assay with Cys, the data obtained over 4 h and at a single Cys concentration were used to calculate reaction rates. The timepoints obtained at the most responsive wavelength were used (see the Supporting Information, Stability and Reactivity Screening). Since the experiment was performed under pseudo-first-order conditions, we obtained *k*_obs_ by fitting experimental data to a one-phase decay equation, which in turn was converted to the second-order reaction rate constant (*k*_2_) by dividing *k*_obs_ by the Cys concentration.

**Figure 4:**
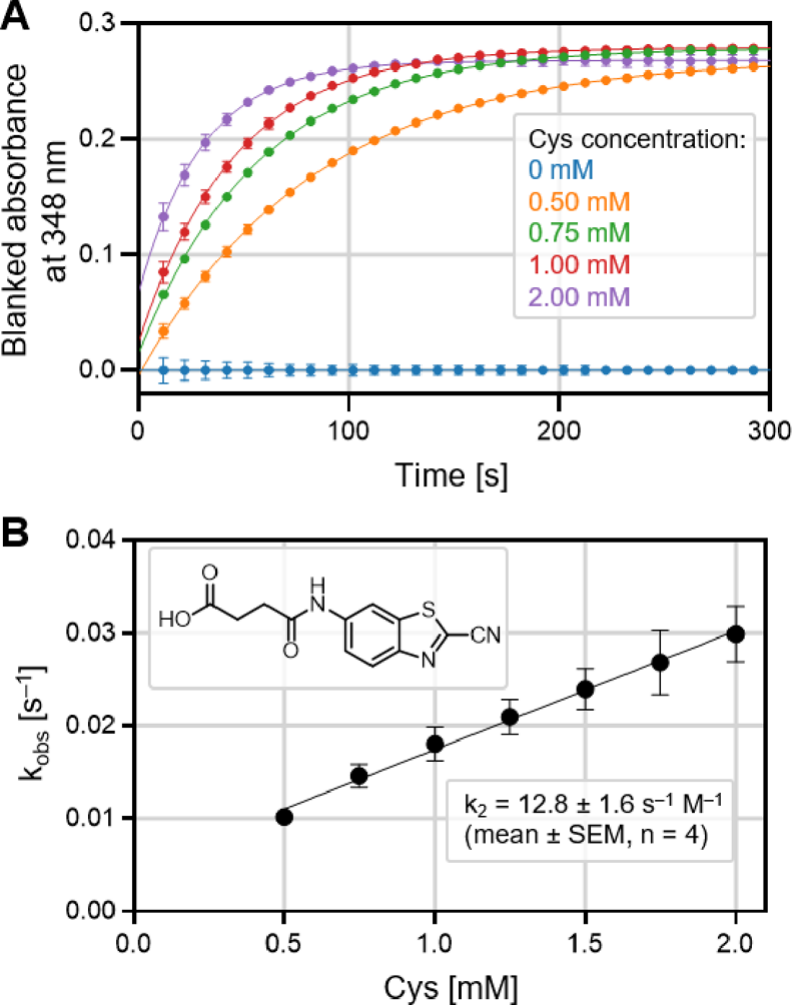
Reaction rate evaluation from multiple Cys concentrations for a reference compound **117**.^25^ (A) Blanked absorbance signals at 348 nm of **117** (50 μM) in the presence of different concentrations of Cys. The solid lines represent the best fit for a single exponential function. (B) Linear relationship plot of *k*_obs_ vs Cys concentration to give second-order reaction rate constant (*k*_2_). The results for **117** are in accordance with the literature data (Table S3).

Compounds that were found to be hyperreactive with Cys in the UV–vis-based screening assay were evaluated in a faster and more accurate assay, as we were primarily interested in compounds that could react rapidly with Cys. Again, we followed changes in absorbance of the compounds over time in the presence of seven different concentrations of Cys. TCEP was added to the reaction mixture to mimic the conditions used in assays with peptides or proteins. For each experiment, *k*_obs_ was determined by fitting experimental data to a one-phase decay equation. Then, *k*_obs_ values at different Cys concentrations were plotted, and *k*_2_ was obtained from the slope by linear regression (Figure 4).

First, we compared the results for representative compounds evaluated by both methods, and *k*_2_ values were in the same range (Table S3). Moreover, the results for a resynthesized reference compound **117** agreed with the literature data (Table S3).^25,31^ We should note that the reaction rates in the literature were determined at room temperature (23 °C), whereas our experiments were performed at 37 °C.

**Figure 5:**
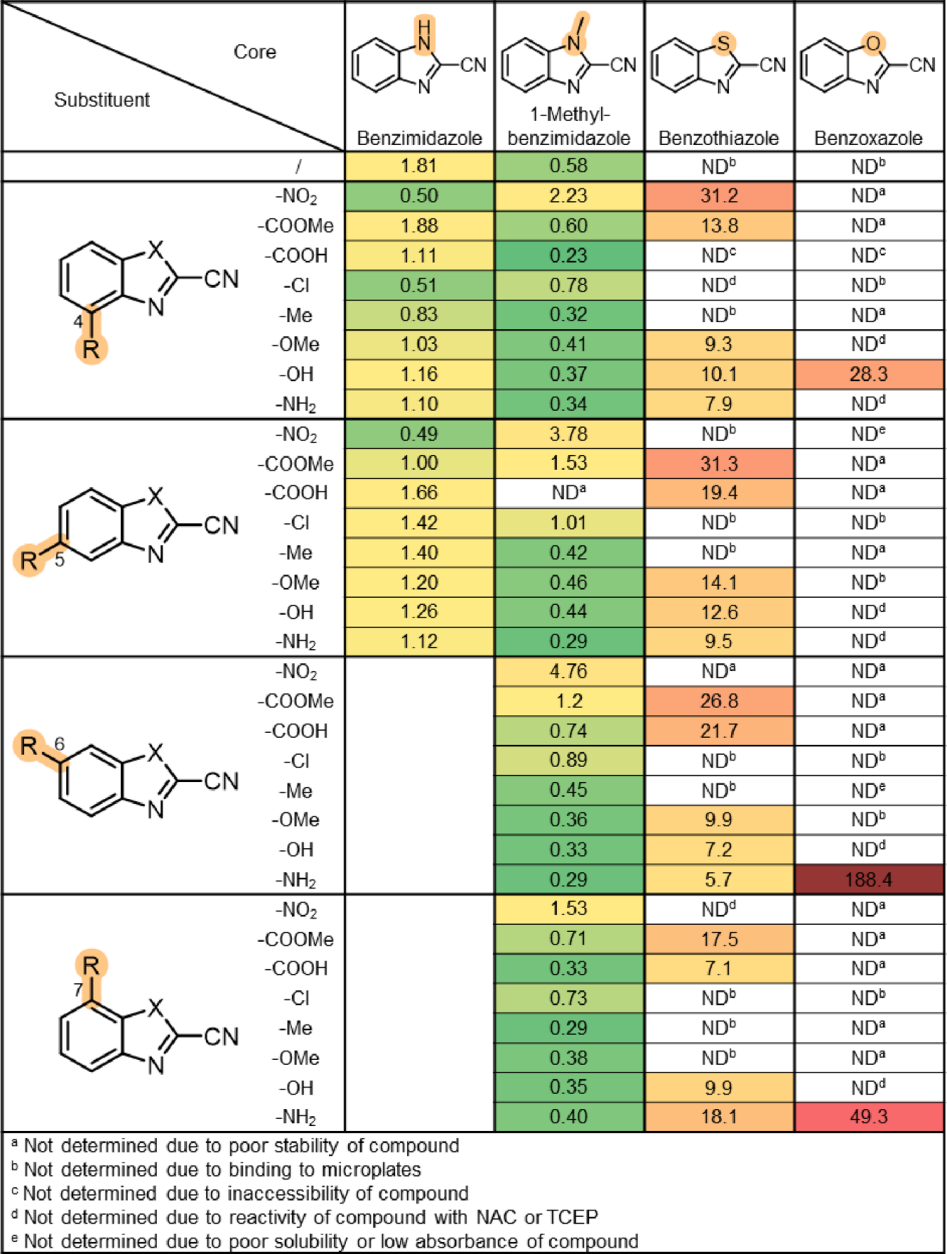
Second-order reaction rate constants (*k*_2_ in s^–1^ M^–1^) for reaction with Cys at 37 °C. Transposed tables for each core separately are provided in Figure S19.

The results of the Cys reactivity assay revealed that CBXs cover a wide range of reactivities with Cys with *k*_2_ values spanning three orders of magnitude (Figure 5, Figure S19). The trends were consistent with those from the stability experiments. Essentially, CBIs showed similar reactivity in comparison to their 1-methyl counterparts (average *k*_2_ values of 1.1 and 0.9 s^–1^ M^–1^, respectively), whereas the reactivities of CBTs and CBOs were significantly higher (average *k*_2_ values of 14.9 and 88.7 s^–1^ M^–1^, respectively). Overall, the reactivity increased from electron-donating to electron-withdrawing substituents, except for 7-substituted CBTs and nitro-substituted CBIs. For all four core sets, we observed that 5- and 6-substituted compounds were more reactive than 4- and 7-substituted analogs. Interestingly, 6-amino-CBT (**75**) and 6-hydroxy-CBT (**74**) (the most commonly utilized representatives) were among the least reactive CBTs. For example, the most reactive CBTs (**52** and **61**) were 5.5-fold more reactive than 6-amino-CBT (**75**). We should note here that further derivatization of the compounds in future applications is likely to impact the reactivity. In particular, the succinic acid derivative (**177**) of 6-amino-CBT (**75**) is about 2-fold more reactive (Table S3, Figure 5).

### Oligopeptide Labeling

To determine the selectivity of our compounds, we explored the formation of adducts with oligopeptides by an LCMS method using two undecapeptides containing various nucleophilic amino acids. They differed only by the N-terminal residue, with Cys (**UP1**, CGKGCGSGYGW) replaced by Ala for the negative control (**UP2**, AGKGCGSGYGW). Both oligopeptides contained nonterminal nucleophilic Cys, Lys, Ser, and Tyr residues separated by Gly residues and Trp residue at the C-terminus to enable UV detection. The addition of TCEP (10 equiv) was necessary to prevent disulfide formation, especially intramolecular disulfides of **UP1**. Nevertheless, a small amount of UP1-disulfide was still detected in some samples (as well as in a blank sample) regardless of the reactivity of compounds. The compounds (2 equiv) were incubated with the oligopeptides (1 equiv) at 37 °C for 30 min and immediately analyzed by LCMS to determine the labeling. The excess of compounds over the oligopeptides in our assay setup was used to thoroughly test the selectivity and possible labeling of other residues.

**Figure 6:**
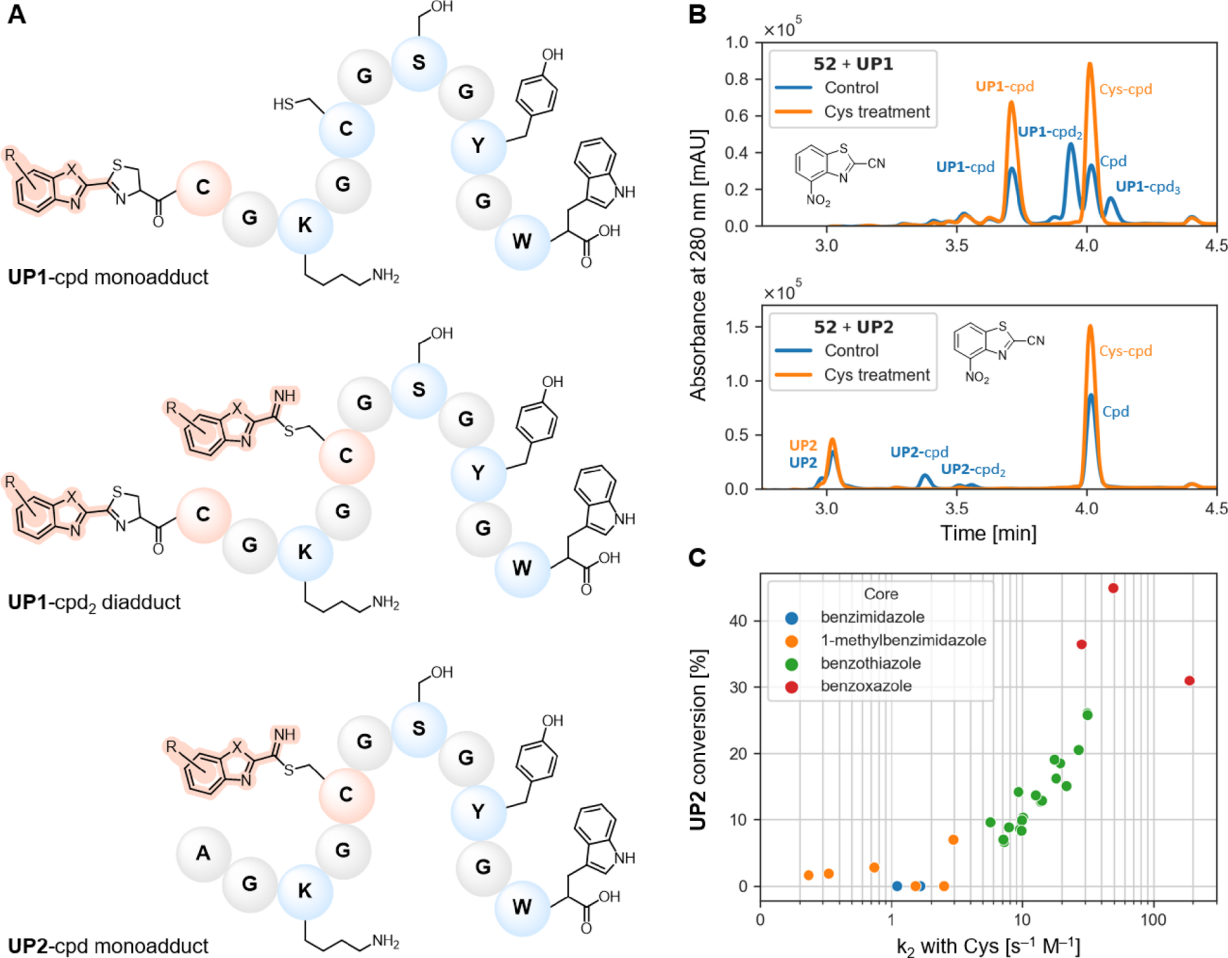
(A) Proposed adducts between CBXs and oligopeptides (**UP1**, CGKGCGSGYGW; **UP2**, AGKGCGSGYGW), i.e., irreversible thiazoline with N-terminal Cys and reversible thioimidate with internal Cys. **UP1** adducts with monolabeled internal residues were not detected. (B) UHPLC chromatograms at 280 nm for the Cys treatment experiment with compound **52**. Oligopeptides were incubated with the compound for 30 min at 37 °C; then, Cys was added and incubated for another 30 min. Full chromatograms are provided in the Supporting Information, Figure S21 and Oligopeptide Labeling. (C) Correlation between *k*_2_ with Cys (Figure 5) and conversion of **UP2** oligopeptide without the N-terminal Cys.

For each UV chromatogram peak, we analyzed the mass spectra to determine which species were present. We were able to distinguish between the labeling of the N-terminal and the internal residues of the oligopeptide **UP1** because the mass difference between these two products corresponds to one NH_3_ molecule (17.0265 Da), which is eliminated during the formation of the thiazoline ring. For the oligopeptide **UP1**, the N-terminal Cys was labeled in all cases and **UP1** was either monolabeled (**UP1**-cpd monoadduct), dilabeled (**UP1**-cpd_2_ diadduct), or trilabeled (**UP1**-cpd_3_ triadduct). Correspondingly, **UP2** was either monolabeled (**UP2**-cpd monoadduct) or dilabeled (**UP2**-cpd_2_ diadduct). Proposed adducts are presented in Figure 6A and Figure S20.

In general, for the CBIs and Me-CBIs investigated, the conversion of **UP1** was not complete under the conditions used, and only monoadducts with N-terminal Cys were detected (except for compound **27**), whereas no adducts with **UP2** were present (see Table S4 and the chromatograms provided in the Supporting Information, Oligopeptide Labeling). These compounds showed the highest selectivity toward N-terminal Cys. In contrast, the conversion of **UP1** in the presence of CBTs and CBOs was complete, and predominantly, mono- and dilabeled **UP1** adducts were detected. On the other hand, incubation of **UP2** with the latter two compound classes resulted in monolabeled **UP2** with parent oligopeptide still present (incomplete conversion of **UP2**). Since internal residues were labeled in addition to N-terminal Cys, CBTs and CBOs are considered less selective, but not to such an extent that all compounds (2 equiv) would be consumed. In comparison, the labeling profile for benchmark 6-hydroxy-CBT and 6-amino-CBT was similar to the rest of CBTs and CBOs, i.e., mono- and dilabeled **UP1** adducts and monolabeled **UP2** and parent **UP2**.

We should emphasize here that lack of complete selectivity is not necessarily an issue for the relevance of click reaction with Cys. Namely, the thioimidate adduct with internal Cys is reversible and thus susceptible to hydrolysis,^25,49^ unless stabilized by nearby residues.^30^ To confirm the reversibility of the adducts, we performed a Cys treatment in which Cys was added after the compound had been incubated with the oligopeptides for 30 min and then incubated for an additional 30 min (Figure 6B, Figure S21, see also chromatograms for compounds **35**, **52**, and **83**). We observed that only monoadducts with **UP1** remained (thiazoline with N-terminal Cys), while the excess compound formed a thiazoline product with the added Cys. This altogether confirms the reversibility of the thioimidate with internal Cys. However, when the treatment was performed with dithiothreitol (DTT) instead of Cys, no reversibility was observed (Figure S22, see also chromatograms for compounds **35**, **52**, and **83**). This indicates that the labeled peptide tolerates the presence of DTT and that the 1,2-aminothiol moiety is necessary to achieve reversibility. Of note, DTT did not completely quench the excess nitrile, whereas Cys did (Figures S21 and S22).

The stability of adducts with **UP1** was monitored for 24 h in phosphate buffer at pH 7.4, 6.5, and 5.5. In addition, Tris buffer (containing free amine) at pH 7.4 was used. Compounds **35** and **83** were found to be stable for at least for 4–8 h in PBS at pH 7.4 and for more than 24 h under other conditions tested (Figures S23 and S24).

We then questioned whether nucleophilic residues other than Cys could be labeled. Based on the results of reactivity with individual amino acids, we expected only Cys residues to be labeled (N-terminal or internal), since none of the compounds reacted with Lys or Ser. However, we observed multiple labeling for certain derivatives, which were also among the most reactive ones (i.e., **52**, **61**, **69**, **108**, and **116**). We propose that the third-labeled residue of **UP1** (or second in case of **UP2**) is one of the nucleophilic residues Lys, Ser, or Tyr (Figure S20), but their adducts cannot be distinguished by mass. Moreover, the third-labeled residue of **UP1** (or second in case of **UP2**) for compound **52** is reversible in nature (Figure 6B, Figure S21). This implies that labeling of Lys is less likely, since it was shown that amidine adducts with Lys are nonreversible,^50^ and proposedly, Ser or Tyr is labeled. Although a fragmentation study would shed light on which residues were labeled, this goes beyond the main message of this manuscript.

Next, we semiquantified the conversion of the oligopeptides following incubation with the compounds by comparing the AUCs of parent oligopeptide peaks at 280 nm with the blank nontreated sample. Stable CBTs and CBOs with complete **UP1** conversion (100%) in 30 min at 37 °C were considered fast-reacting (Table S4). In contrast, the CBIs and Me-CBIs under investigation converted up to 72% of **UP1**. From this, we deduced that the threshold for *k*_2_ with Cys must be greater than 5 s^–1^ M^–1^ for complete labeling in less than 30 min, when performed at low micromolar concentration. The conversion of **UP2** without the N-terminal Cys correlated with the Cys reactivity (Figure 6C), implying that more reactive compounds are less selective for N-terminal Cys, although we have shown that all side reactions are reversible (Figure S21).

**Figure 7:**
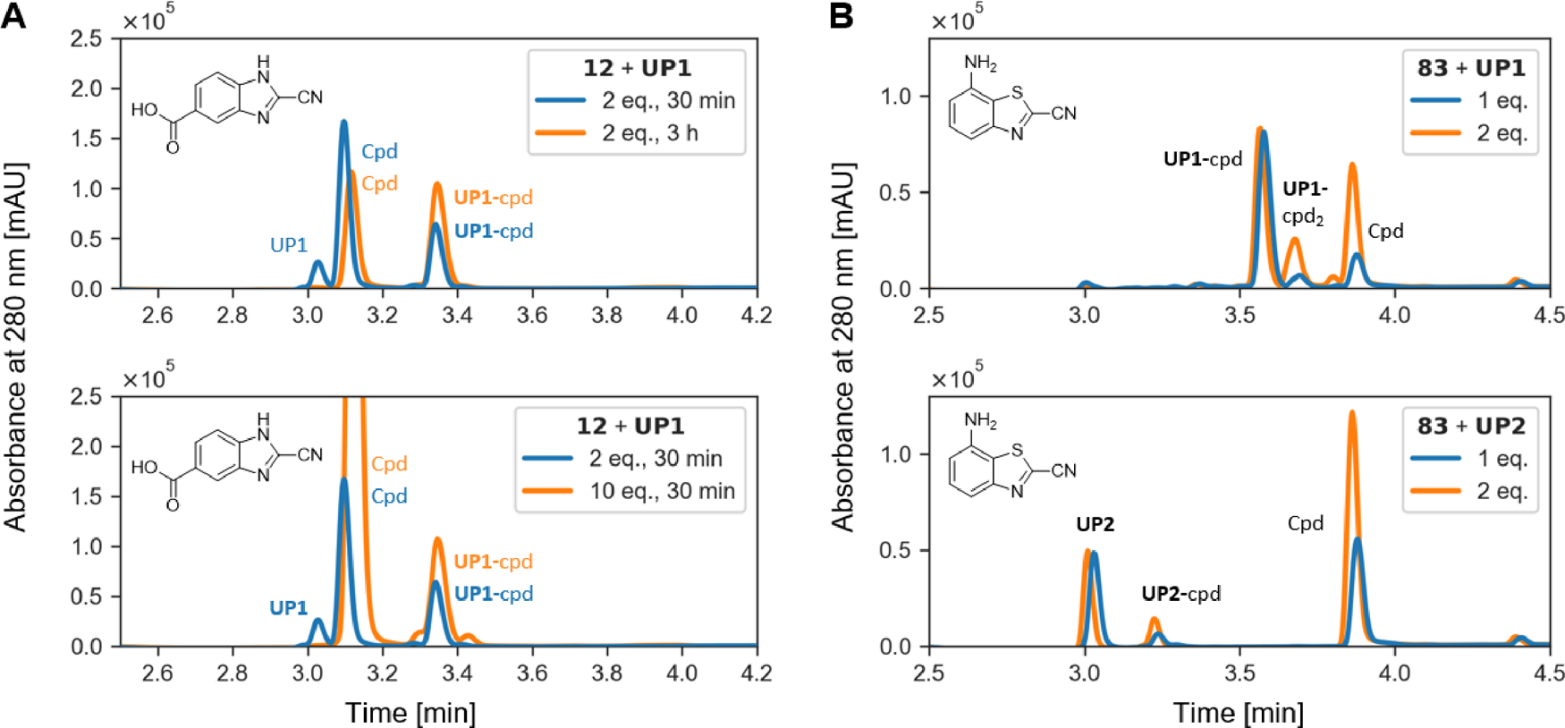
UHPLC chromatograms at 280 nm for tailored labeling experimental conditions. Full chromatograms are provided in the Supporting Information, Oligopeptide Labeling. (A) Highly selective CBI **12** was incubated for 3 h or with a 10-fold excess to achieve complete conversion of **UP1**. Full chromatograms are provided in the Supporting Information, Figure S25. (B) When highly reactive CBT **83** was incubated in an equimolar amount with the oligopeptides, labeling of residues other than the N-terminal Cys was significantly reduced compared with 2-fold excess of the compound. Full chromatograms are provided in the Supporting Information, Figure S26.

Finally, we tailored the experimental conditions to achieve complete labeling of **UP1** with highly selective and less reactive compounds on the one hand and to limit side reactions with highly reactive and less selective compounds on the other hand. For the CBIs, which are the most selective, but less reactive, we could achieve complete labeling of **UP1** with longer incubation times (3 h) or with a 10-fold excess of the compound without labeling residues other than the N-terminal Cys (Figure 7A and Figure S25 show an example for CBI **12**). As shown for two compounds among the most reactive CBTs (**52** and **83**), side reactions can be reversed by adding free Cys (Figure 6B, Figure S21) or can be limited by using equimolar quantity of the compound while maintaining complete conversion of **UP1** (Figure 7B, Figure S26).

**Figure 8:**
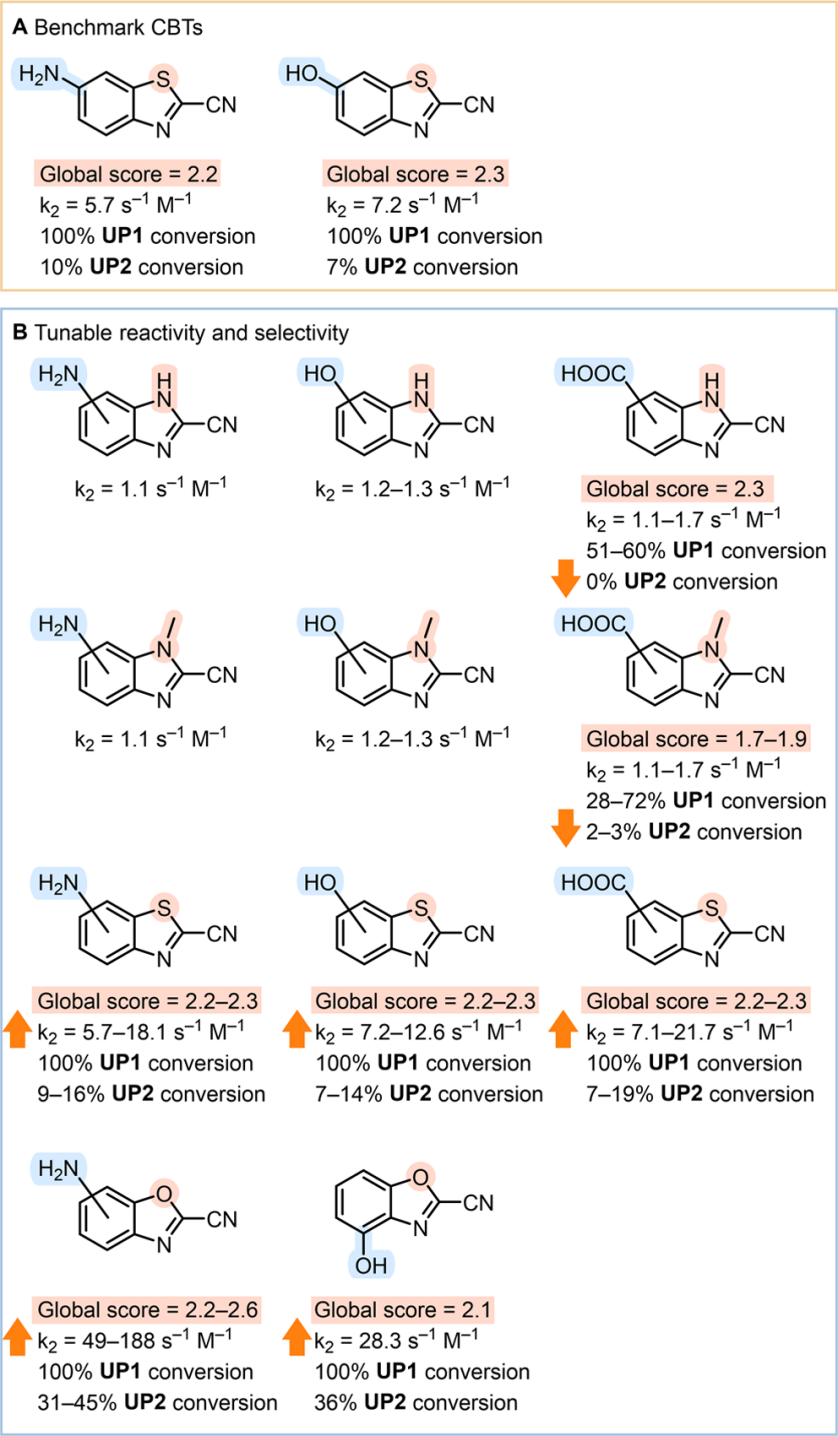
Comparison of global scores, reactivity with Cys (*k*_2_ in s^–1^ M^–1^), and the conversion of oligopeptides (**UP1**, CGKGCGSGYGW; **UP2**, AGKGCGSGYGW). (A) Benchmark 6-amino-CBT and 6-hydroxy-CBT. (B) An overview of more reactive or more selective analogs with hydroxyl, amino, and carboxyl substituents that are readily available for functionalization.

To simplify the comparison of compounds, we used a global scoring function, which accounted for reactivity, selectivity, and derivatization capability for future applications. All tested compounds with derivatization capability (amino, hydroxyl, and carboxylic acid substituents) scored above 1.5, and among those, all CBTs and CBOs were in the same range (2.1–2.3) as benchmark 6-hydroxy-CBT and 6-amino-CBT (Table S4, Figure 8).

## Conclusions

We assembled a systematic library of 116 CBXs and evaluated them in a high-throughput UV–vis-based assay for their aqueous stability and, for stable compounds, reactivity with Cys, NAC, Ser, Lys, TCEP, and DTNB. Kinetic evaluation of the reaction with Cys showed that the reactivity of the nitrile warhead is tunable and can be modulated by electron-withdrawing or electron-donating groups that increase or decrease the reactivity, respectively. To demonstrate applicability, we designed two oligopeptides with multiple nucleophilic residues and determined the selectivity of the nitriles toward N-terminal Cys. All in all, we present a well-characterized set of heteroaromatic nitriles with different electronic properties, substitution vectors, and possible attachment chemistries as an extension to the benchmark 6-hydroxy-CBT or 6-amino-CBT (Figure 8). Changing substituents or the heteroaromatic core enables fine-tuning of reactivity and selectivity to achieve fast and clean N-terminal Cys bioconjugation. Moreover, the tailored experimental conditions that we provide for differentially reactive derivatives can importantly support future applications of this biocompatible click reaction with N-terminal Cys.

## Supporting Information Available

List of compounds and synthetic intermediates (structure, canonical SMILES, and experimental results) (XLSX)
^1^H, ^13^C, and ^1^H-^1^H NOE NMR spectra (PDF)
Experimental procedures; supporting schemes, tables and figures; time-dependent absorbance spectra from the UV–vis-based stability and reactivity assays; oligopeptide labeling chromatograms; and chemistry (PDF)

## Abbreviations Used

CBI: 2-cyanobenzimidazole
Me-CBI: 1-methyl-2-cyanobenzimidazole
CBO: 2-cyanobenzoxazole
CBT: 2-cyanobenzothiazole
CBX: 2-cyanobenz’X’azole
DTT: dithiothreitol
NAC: *N*-acetyl cysteine
DTNB: Ellman’s reagent (5,5-dithio-bis-(2-nitrobenzoic acid))
TCEP: *tris*(2-carboxyethyl)phosphine
TNB^2–^: 5-mercapto-2-nitrobenzoic acid
Cys: cysteine
