# Supplementary material for: Tunable Heteroaromatic Nitriles for Selective Bioorthogonal Click Reaction with Cysteine
Source: Bioconjug Chem. 2023 Jun 24;34(7):1271–81. doi: 10.1021/acs.bioconjchem.3c00163 (PMC10360065; doi:10.1021/acs.bioconjchem.3c00163)

^{1}H, ^{13}C and ^{1}H-^{1}H NOE NMR spectra

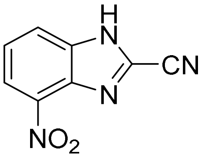

Compound 2

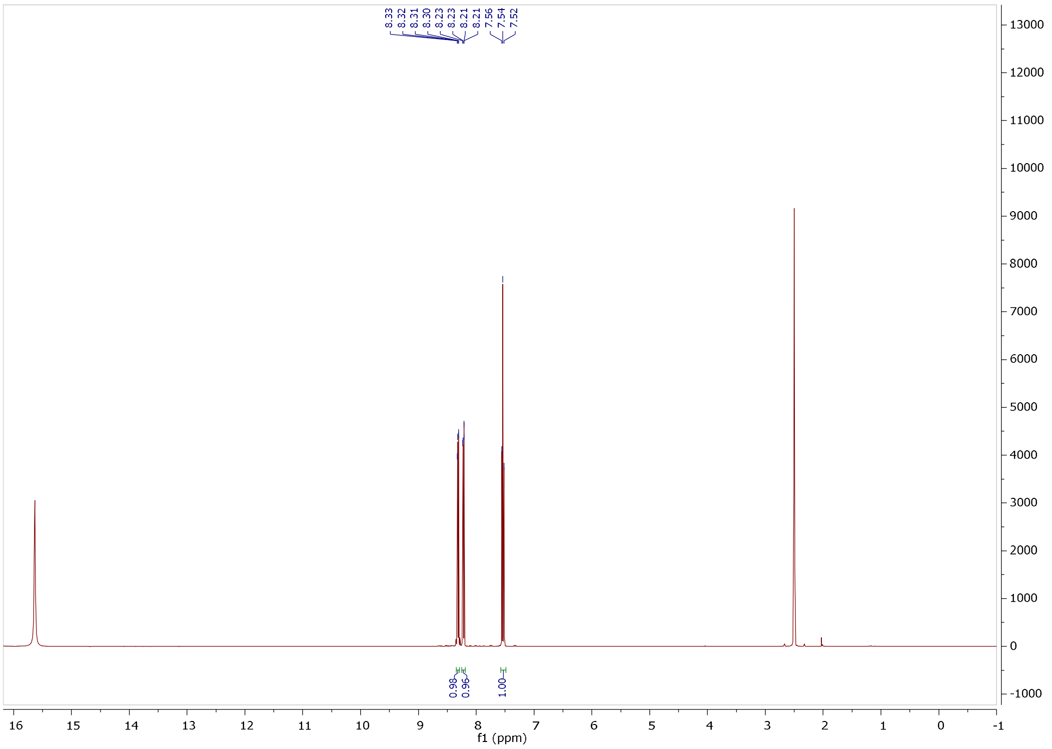

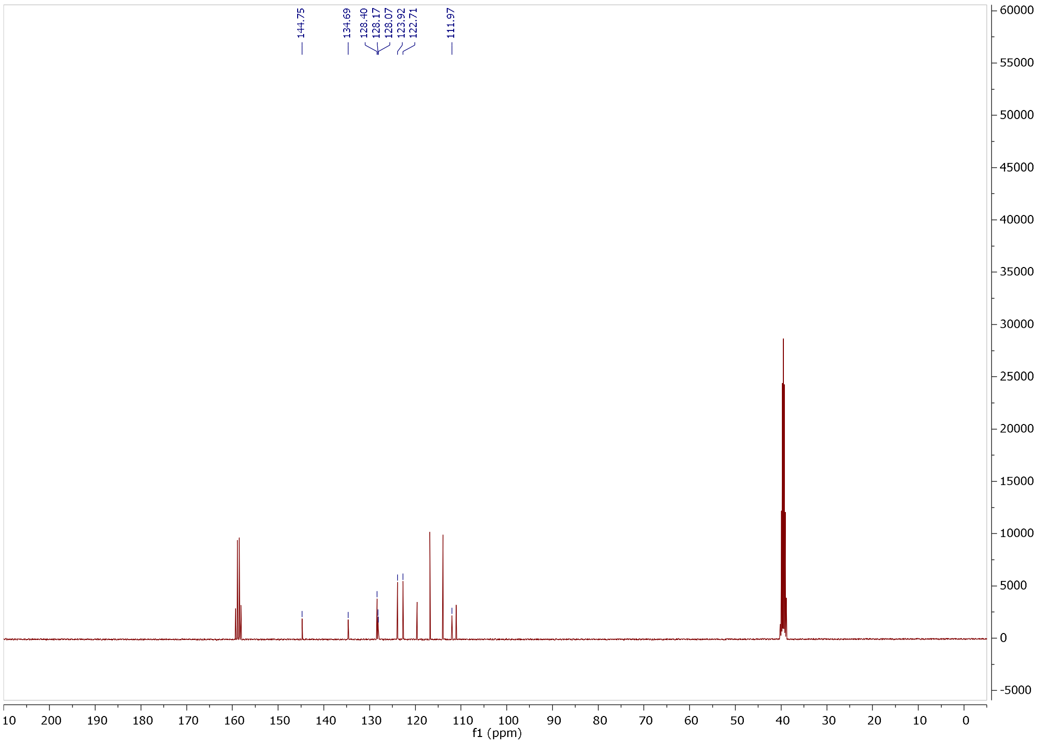

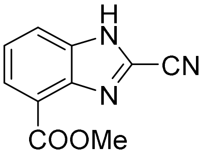
H
N
CN
N
COOMe

Compound 3

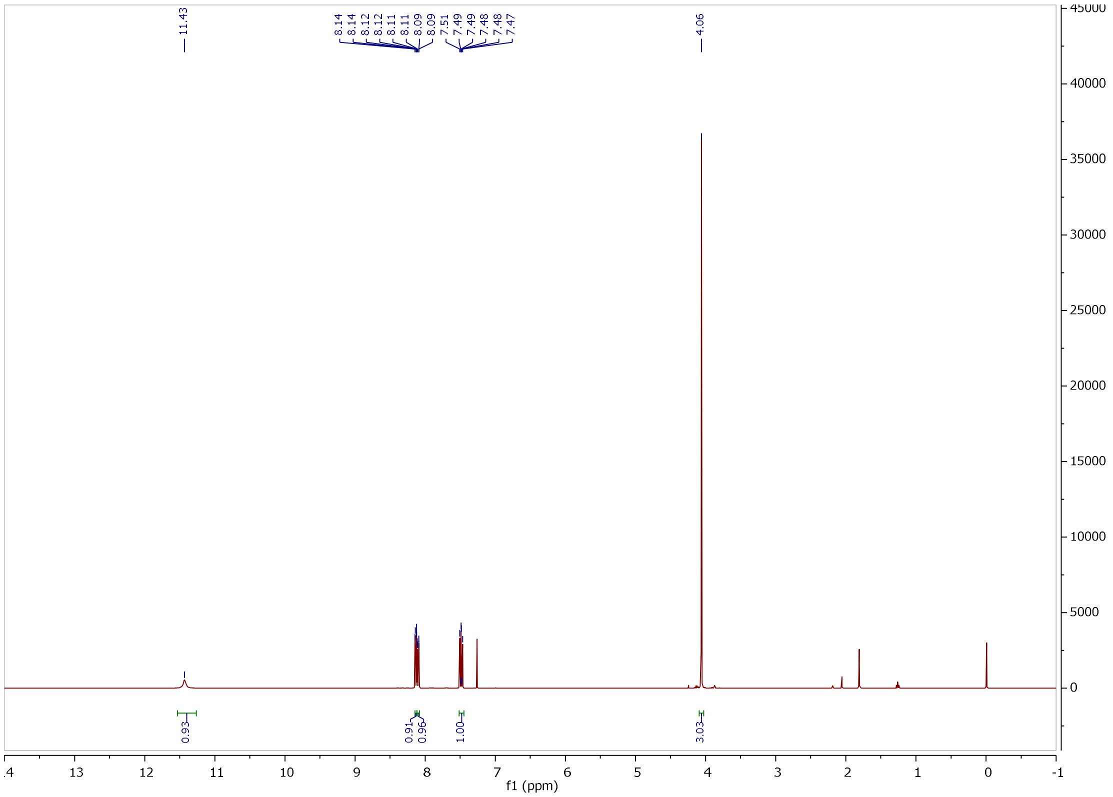
11.43
8.14
8.14
8.12
8.12
8.11
8.11
8.09
8.09
7.51
7.49
7.49
7.48
7.48
7.47
4.06
0.93
0.91
0.96
1.00
3.03
f1 (ppm)

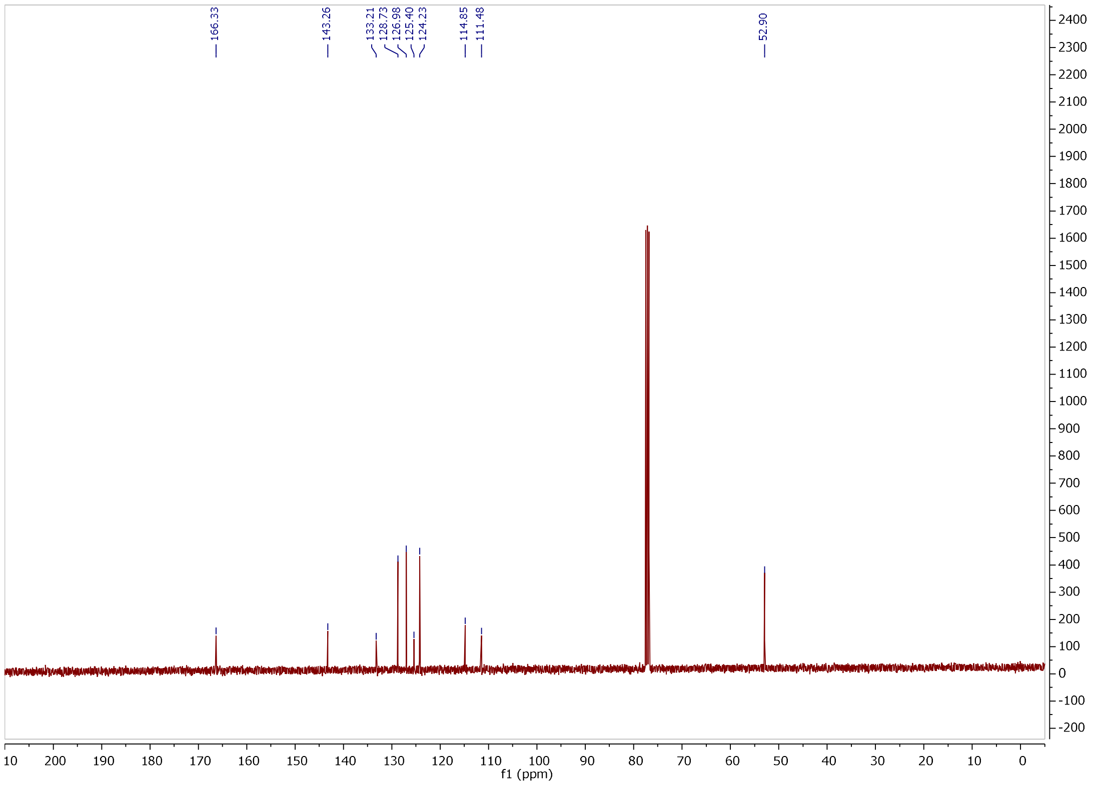
166.33
143.26
133.21
128.73
126.98
125.40
124.23
114.85
111.48
52.90
f1 (ppm)

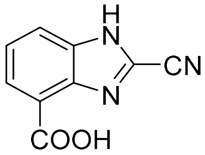

Compound 4

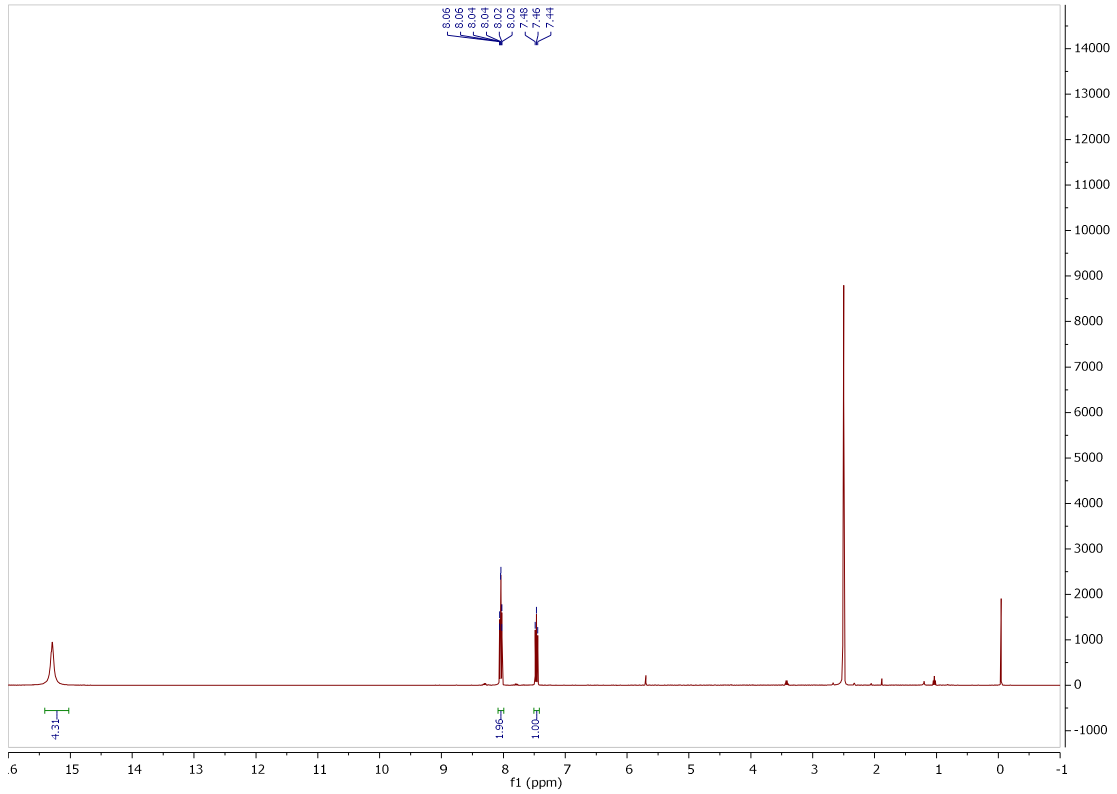

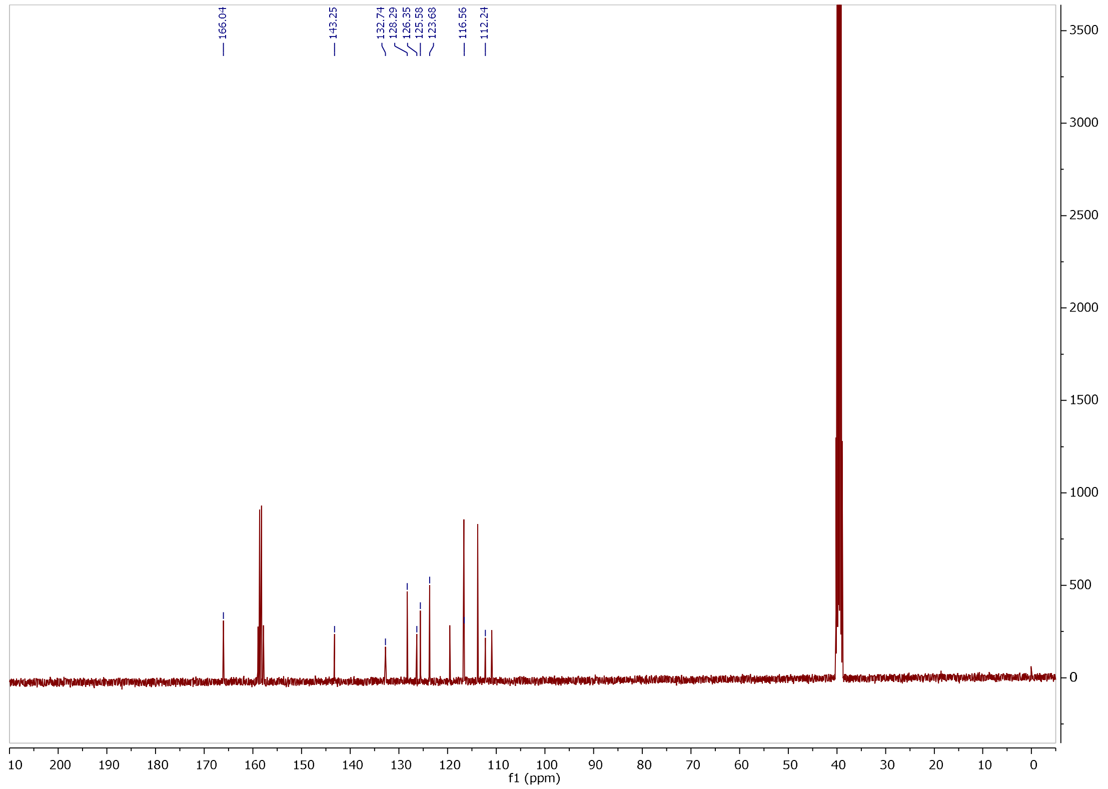

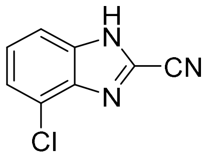
H
N
CN
N
Cl

Compound 5

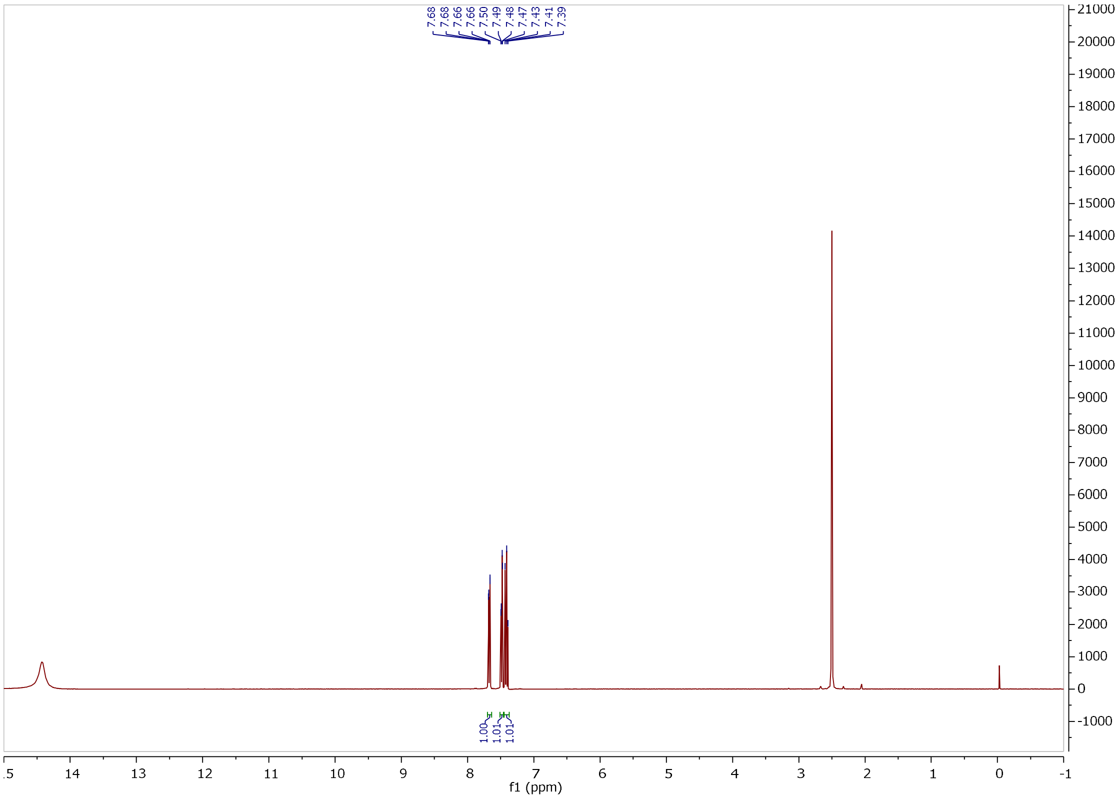
7.68
7.68
7.66
7.66
7.50
7.49
7.48
7.47
7.43
7.41
7.39
1.00
1.01
1.01
f1 (ppm)

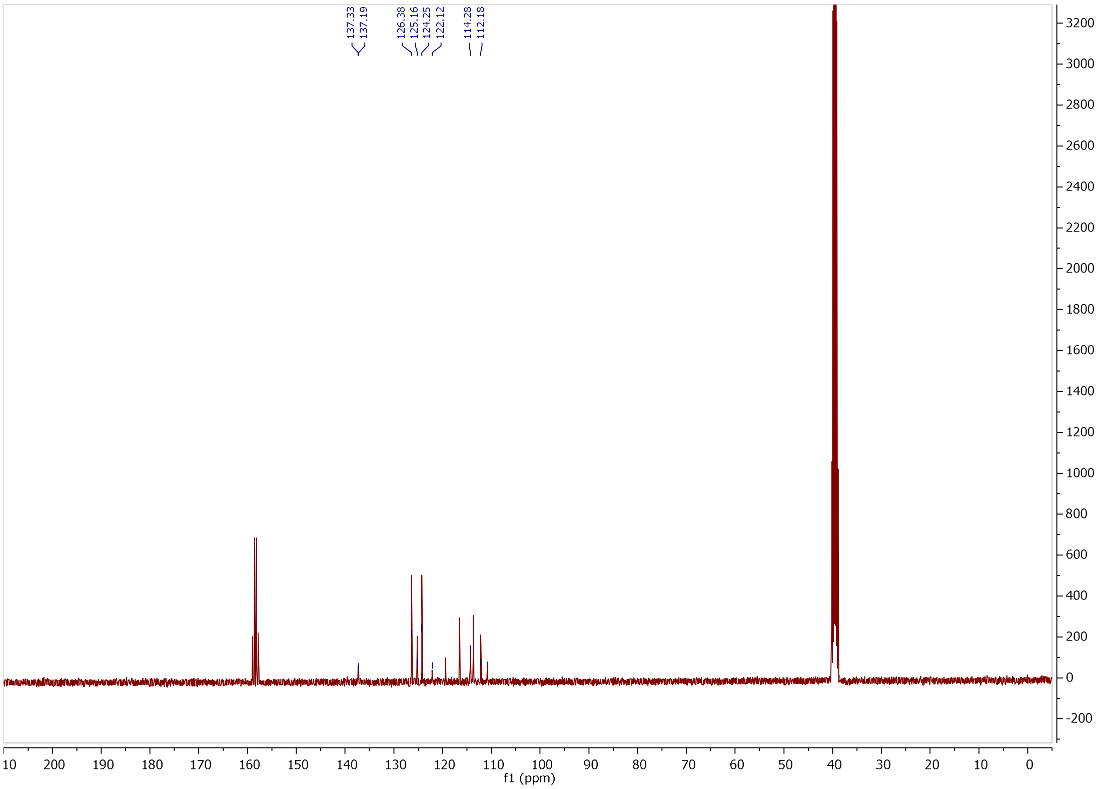
137.33
137.19
126.38
125.16
124.25
122.12
114.28
112.18
f1 (ppm)

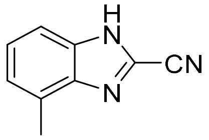
H
N
CN
N

Compound 6

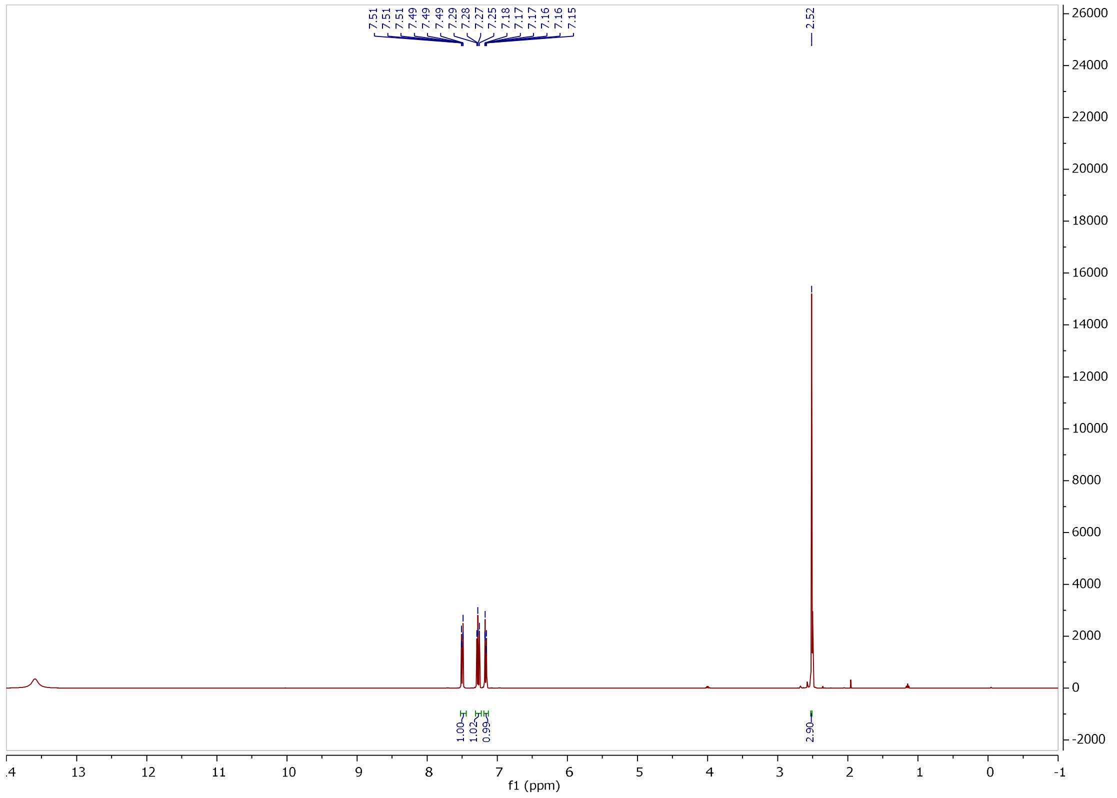
7.51
7.51
7.51
7.49
7.49
7.49
7.29
7.28
7.27
7.25
7.18
7.17
7.17
7.16
7.15
2.52
1.00
1.02
0.99
2.90
f1 (ppm)

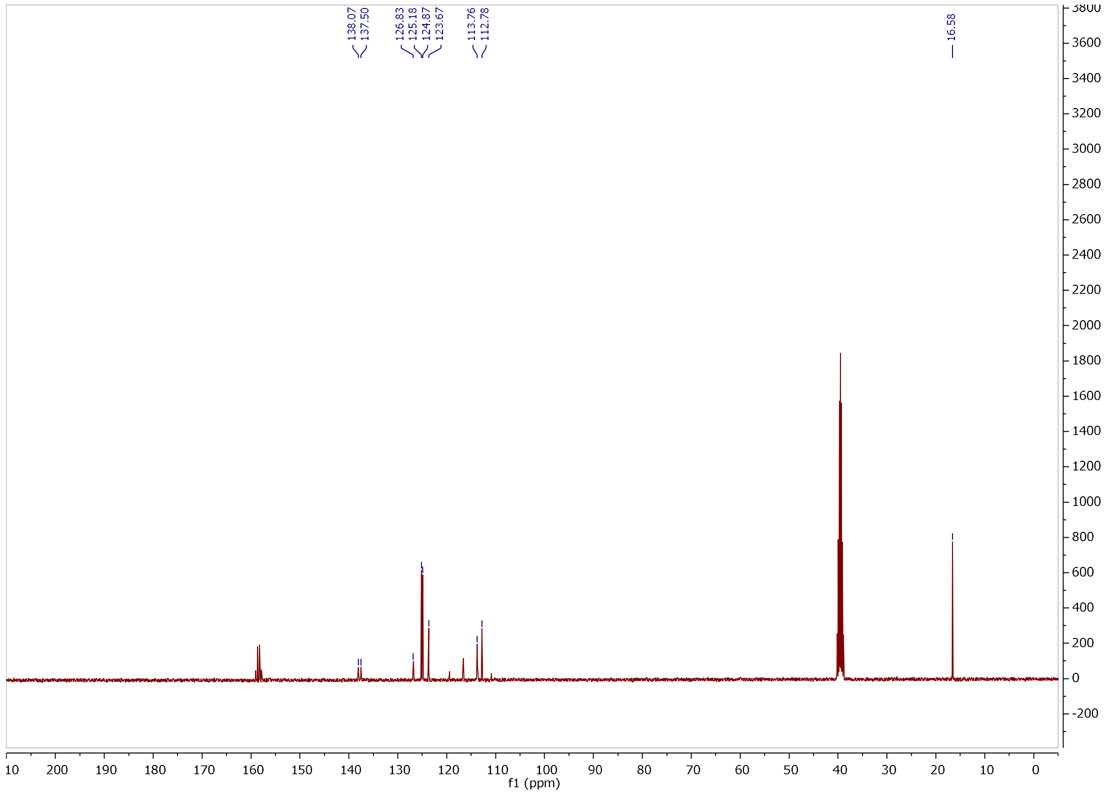
138.07
137.50
126.83
125.18
124.87
123.67
113.76
112.78
16.58
f1 (ppm)

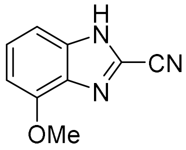
H
N
CN
N
OMe

Compound 7

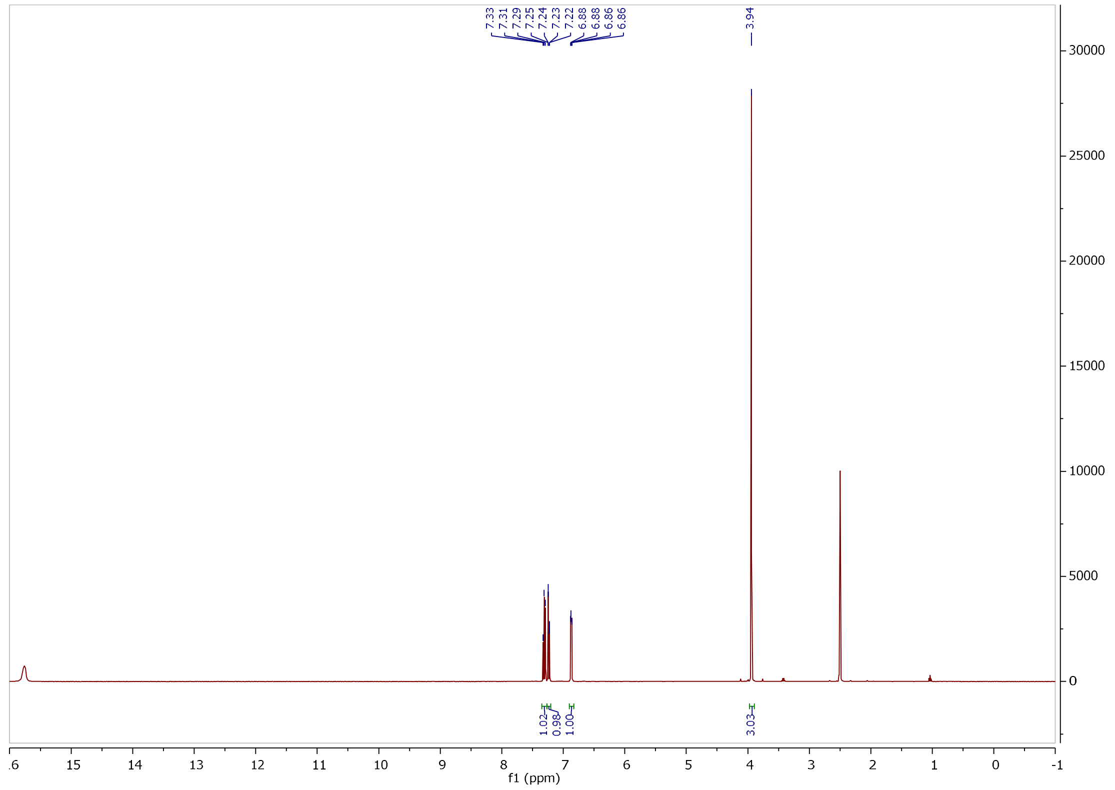
7.33
7.31
7.29
7.25
7.24
7.23
7.22
6.88
6.88
6.86
6.86
3.94
1.02
0.98
1.00
3.03
f1 (ppm)

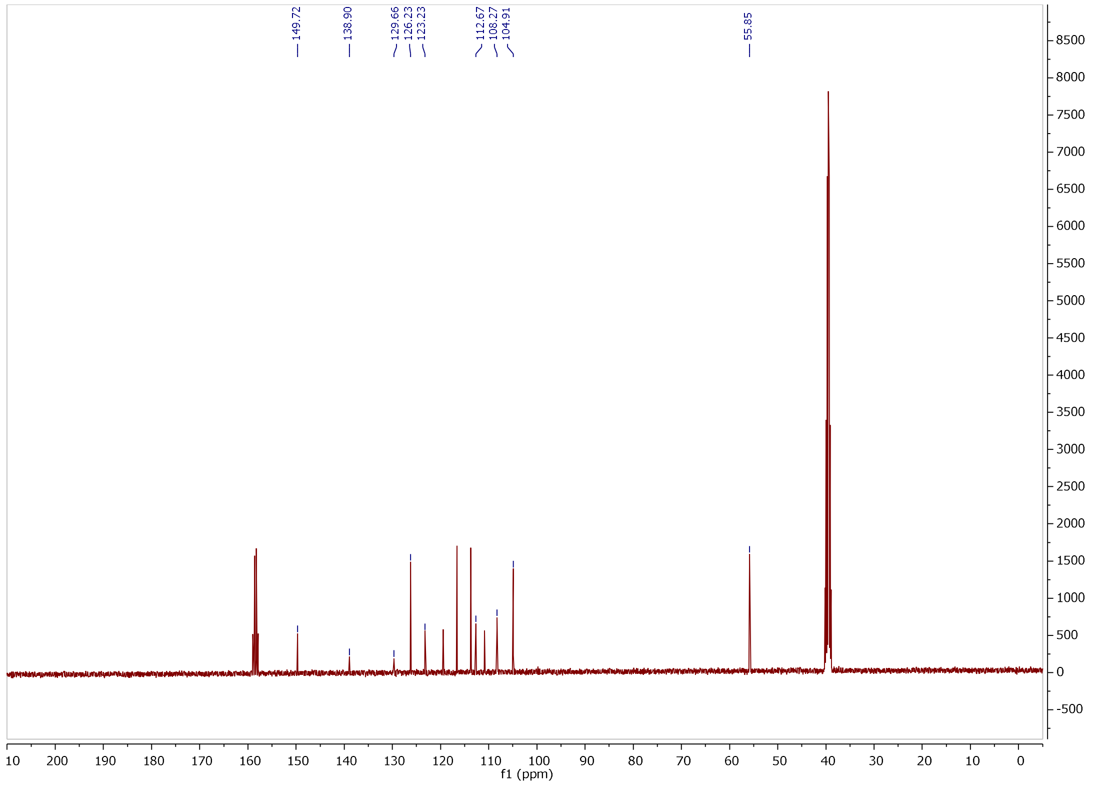
149.72
138.90
129.66
126.23
123.23
112.67
108.27
104.91
55.85
f1 (ppm)

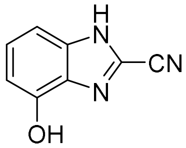
H
N
CN
N
OH

Compound 8

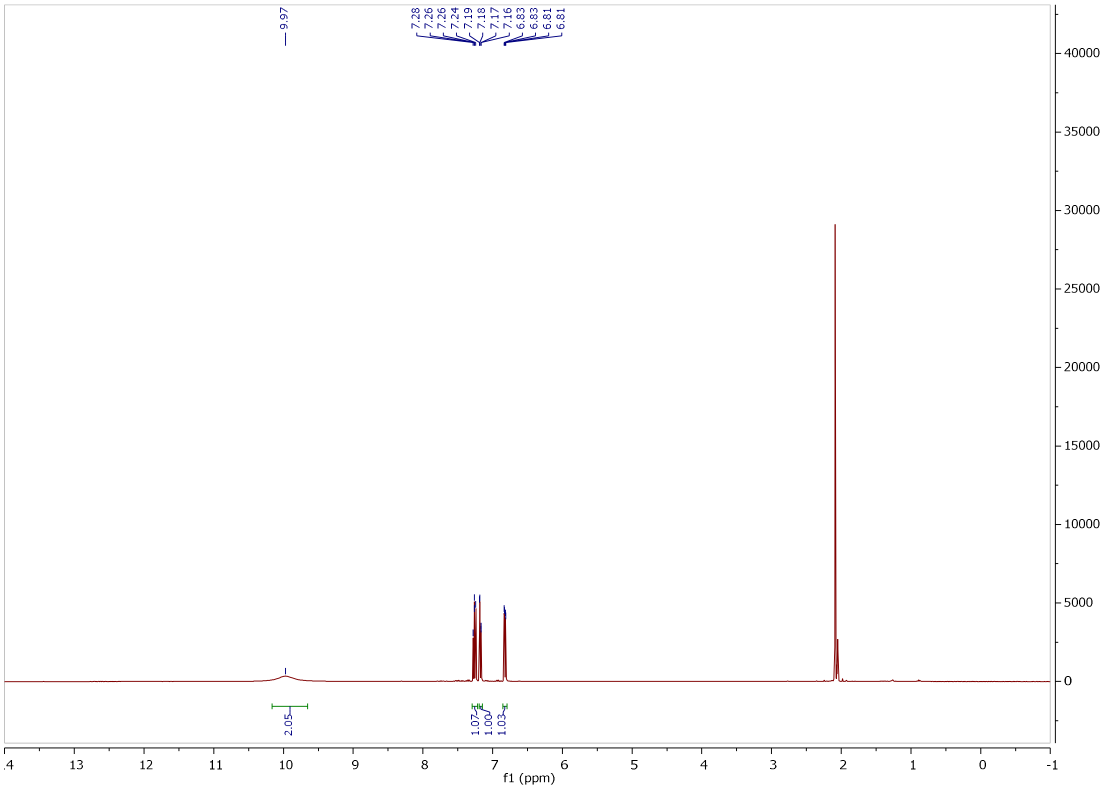
9.97
7.28
7.26
7.26
7.24
7.19
7.18
7.17
7.16
6.83
6.83
6.81
6.81
2.05
1.07
1.00
1.03
f1 (ppm)

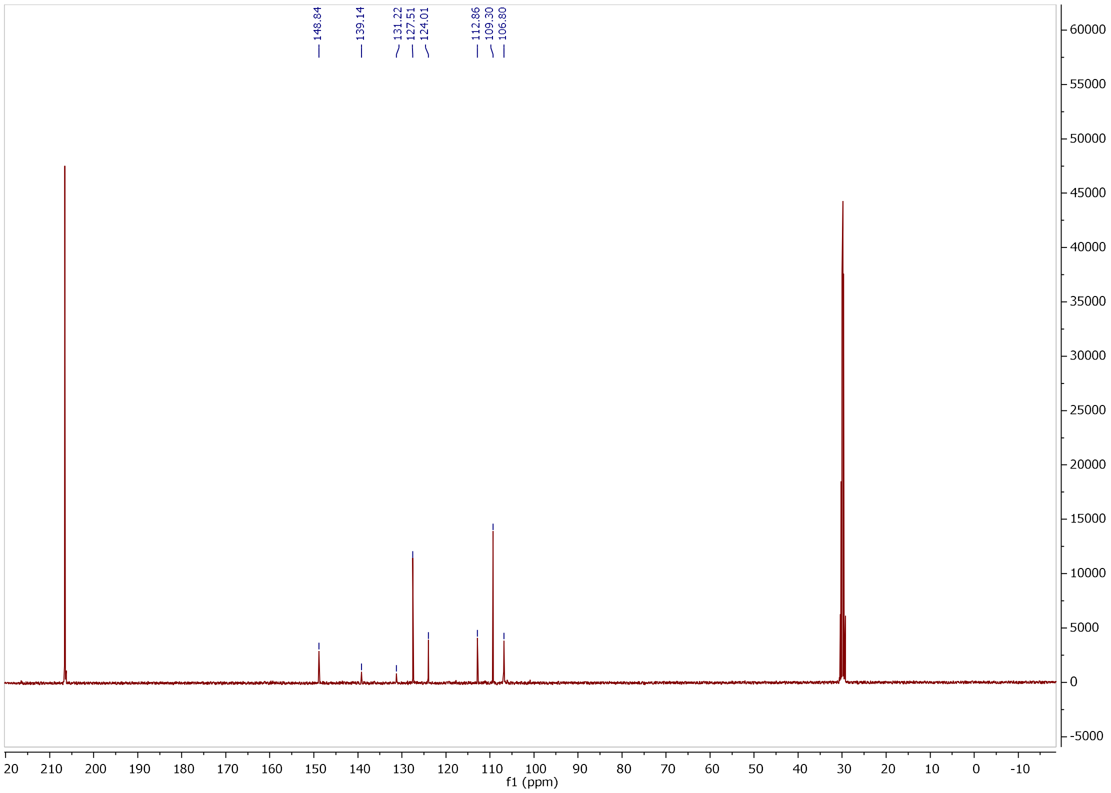
148.84
139.14
131.22
127.51
124.01
112.86
109.30
106.80
f1 (ppm)

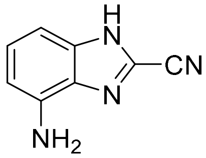
H
N
N
CN
NH2

Compound 9

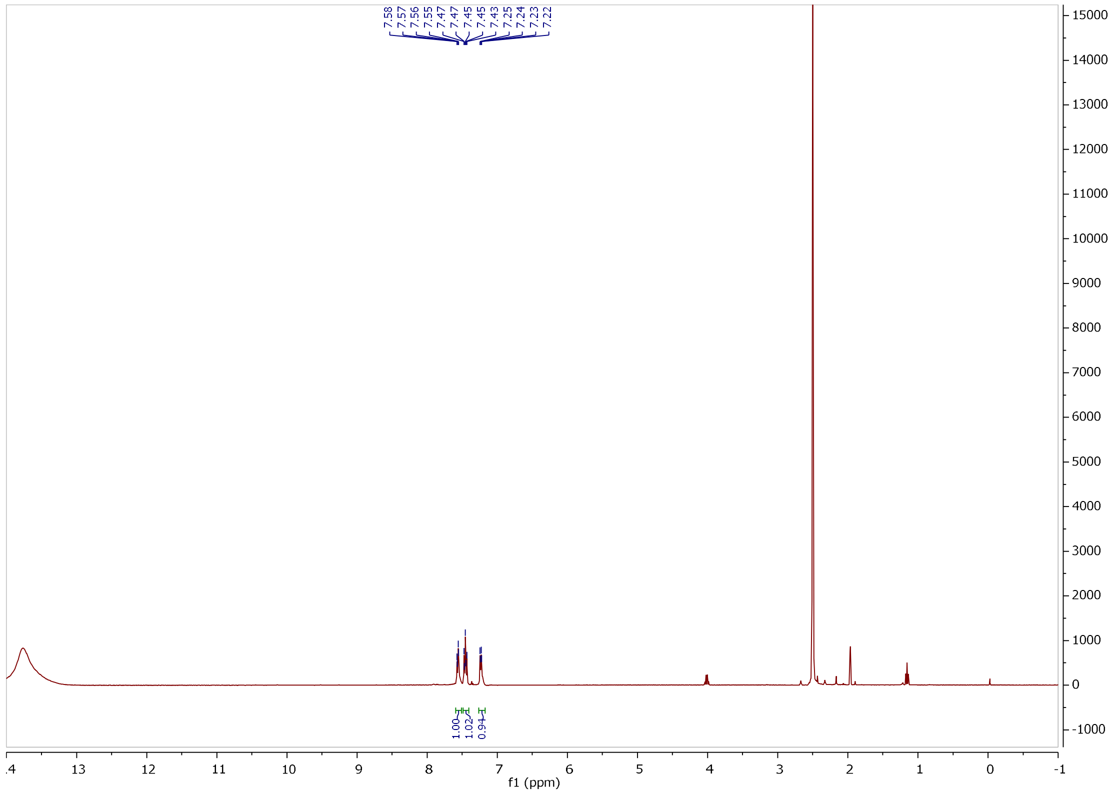
7.58
7.57
7.56
7.55
7.47
7.47
7.45
7.45
7.43
7.25
7.24
7.23
7.22
1.00
1.02
0.94
f1 (ppm)

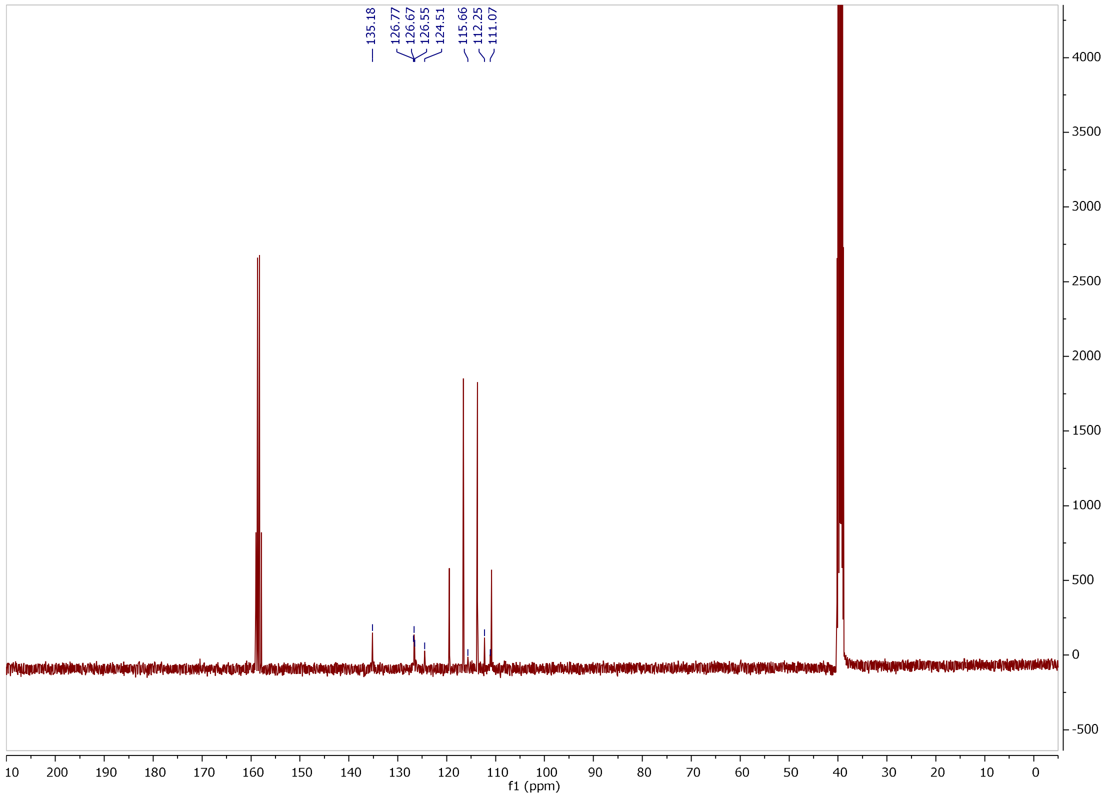
135.18
126.77
126.67
126.55
124.51
115.66
112.25
111.07
f1 (ppm)

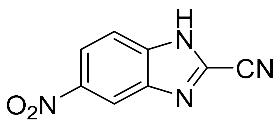
H
N
CN
N
O2N

Compound 10

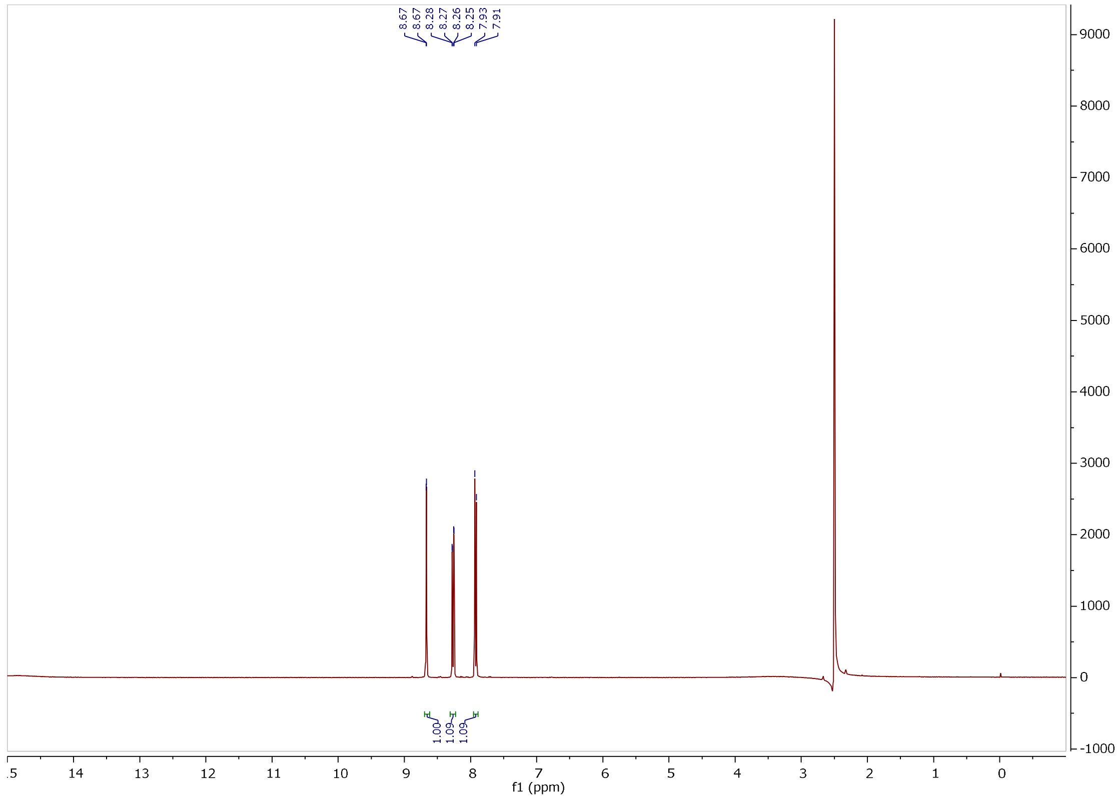
8.67
8.67
8.28
8.27
8.26
8.25
7.93
7.91
1.00
1.09
1.09
f1 (ppm)

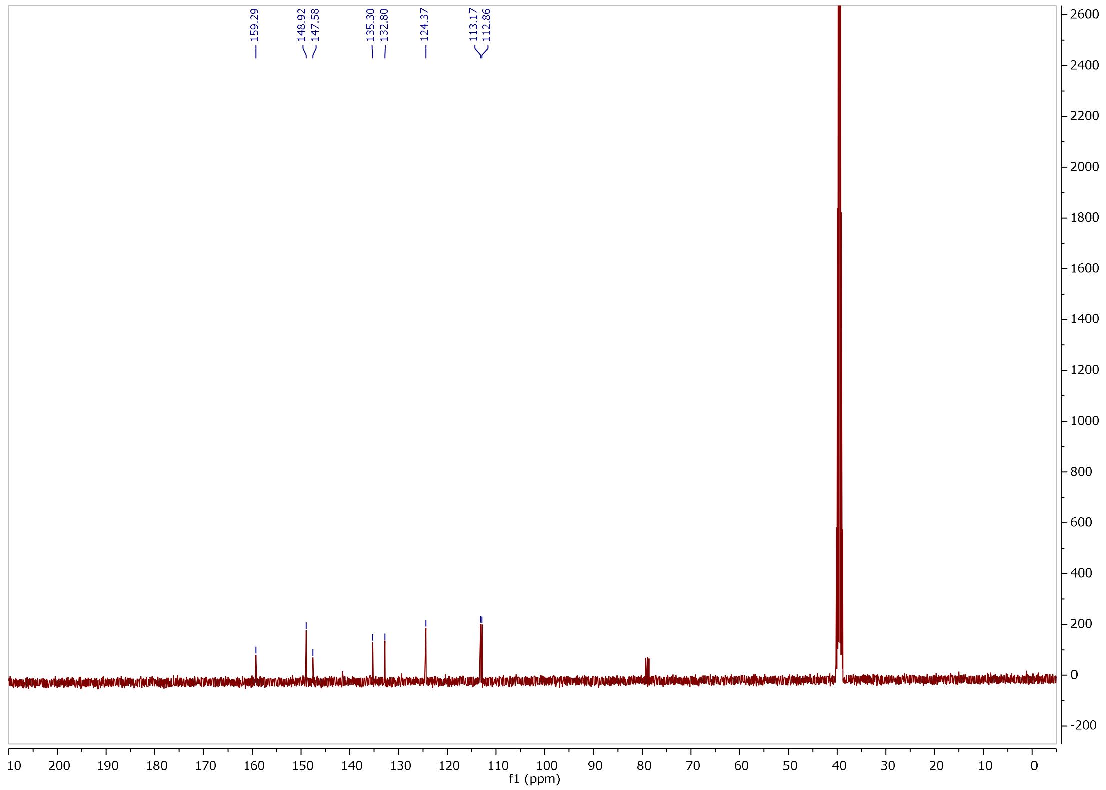
159.29
148.92
147.58
135.30
132.80
124.37
113.17
112.86
f1 (ppm)

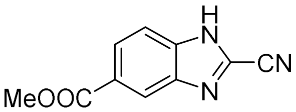

Compound 11

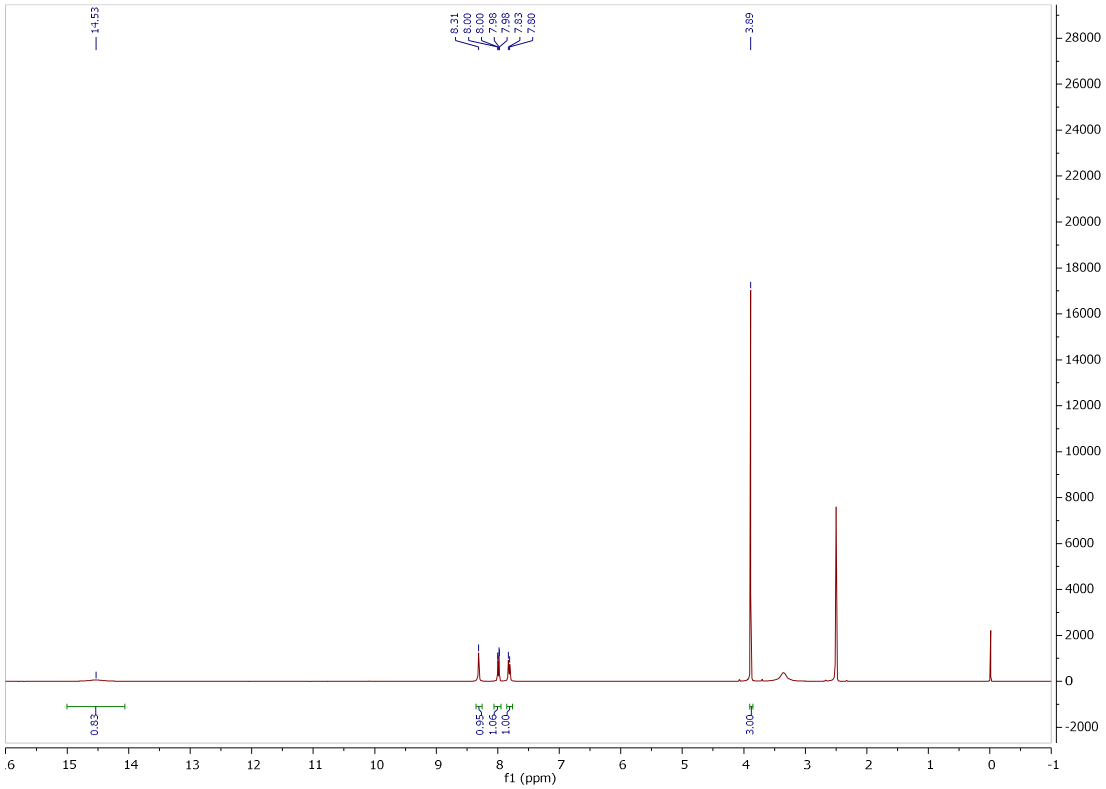

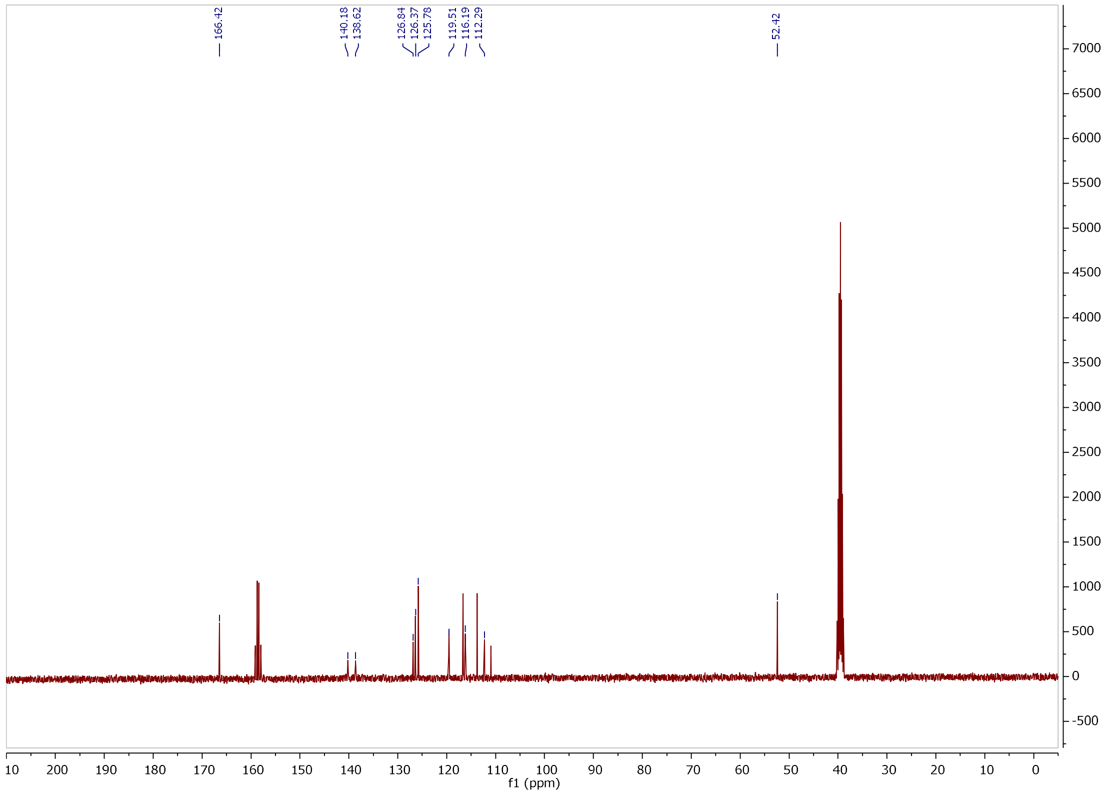

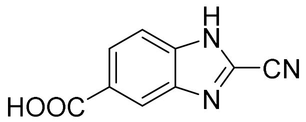
H
N
CN
HOOC
N

Compound 12

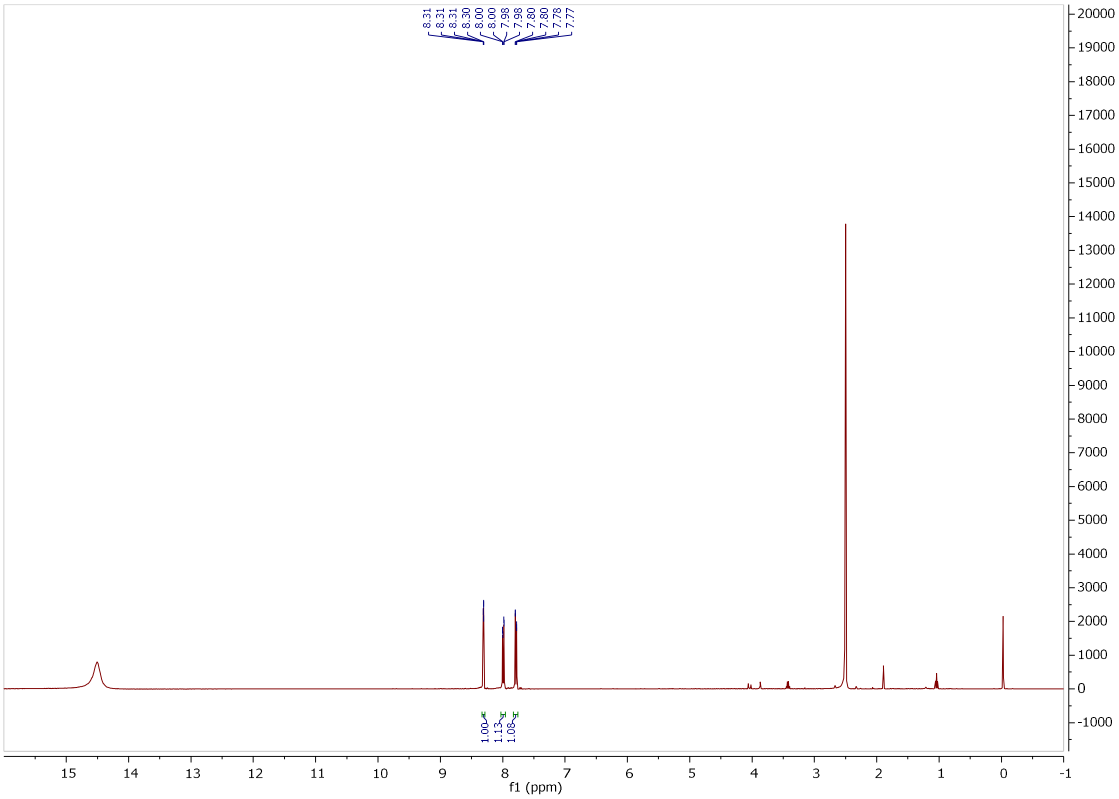
8.31
8.31
8.31
8.30
8.00
8.00
7.98
7.98
7.80
7.80
7.78
7.77
1.00
1.13
1.08
f1 (ppm)

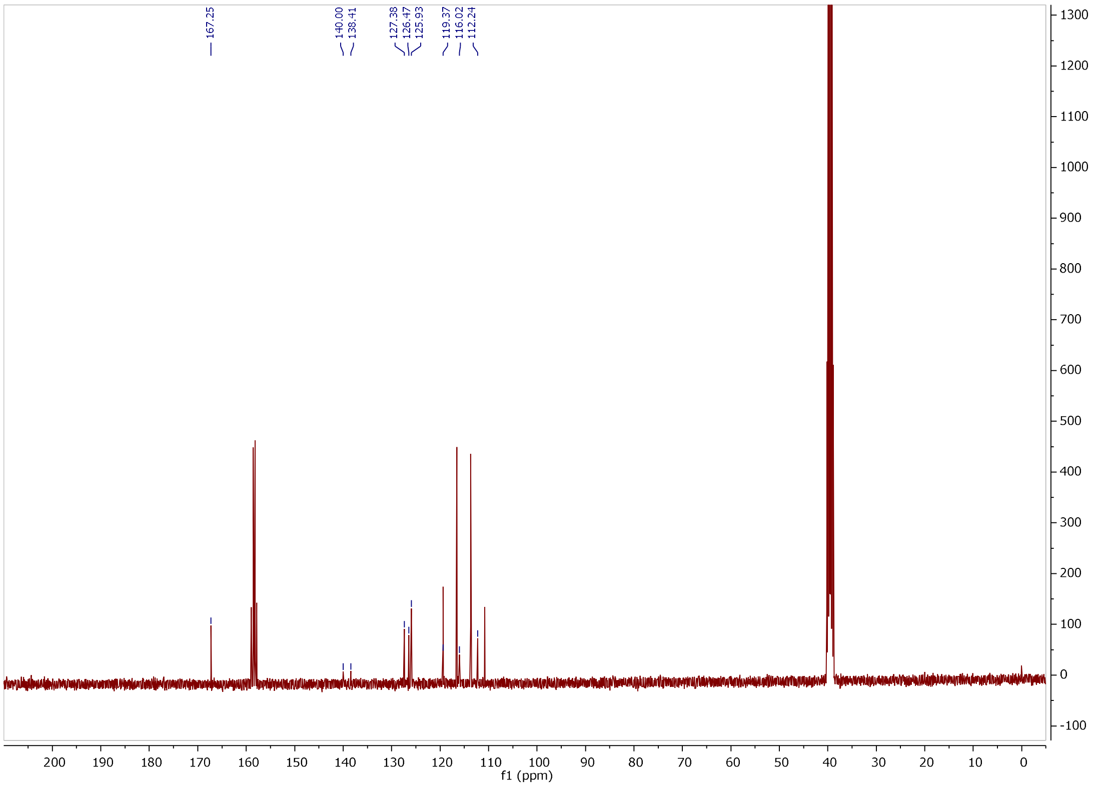
167.25
140.00
138.41
127.38
126.47
125.93
119.37
116.02
112.24
f1 (ppm)

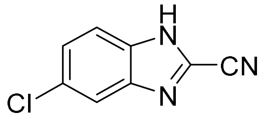

Compound 13

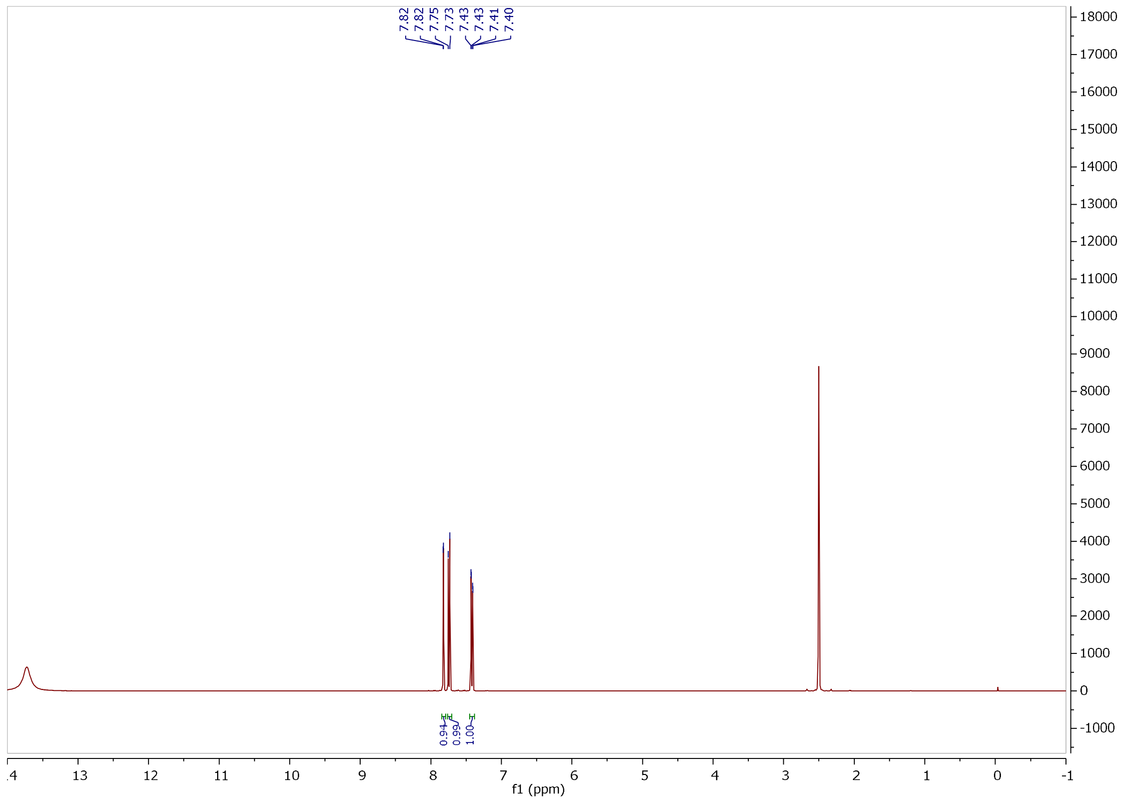

7.82
7.82
7.75
7.73
7.43
7.43
7.41
7.40
0.91
0.99
1.00
f1 (ppm)

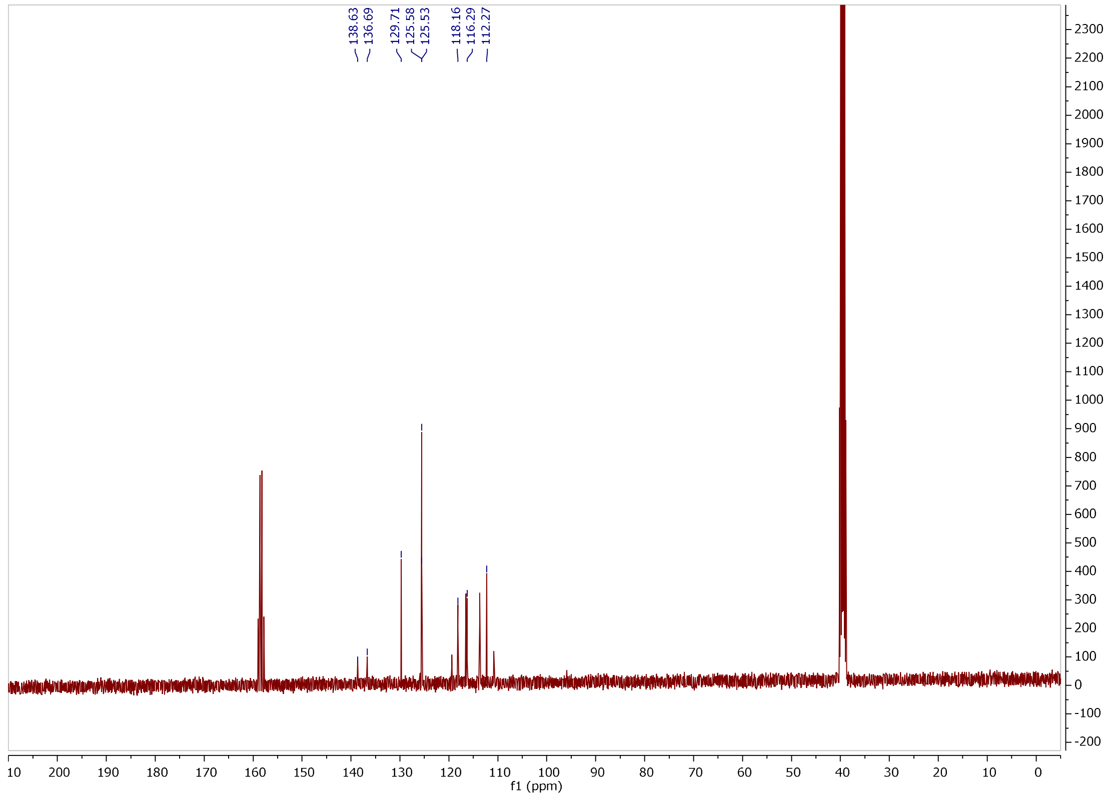

138.63
136.69
129.71
125.58
125.53
118.16
116.29
112.27
f1 (ppm)

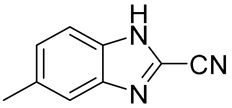

Compound 14

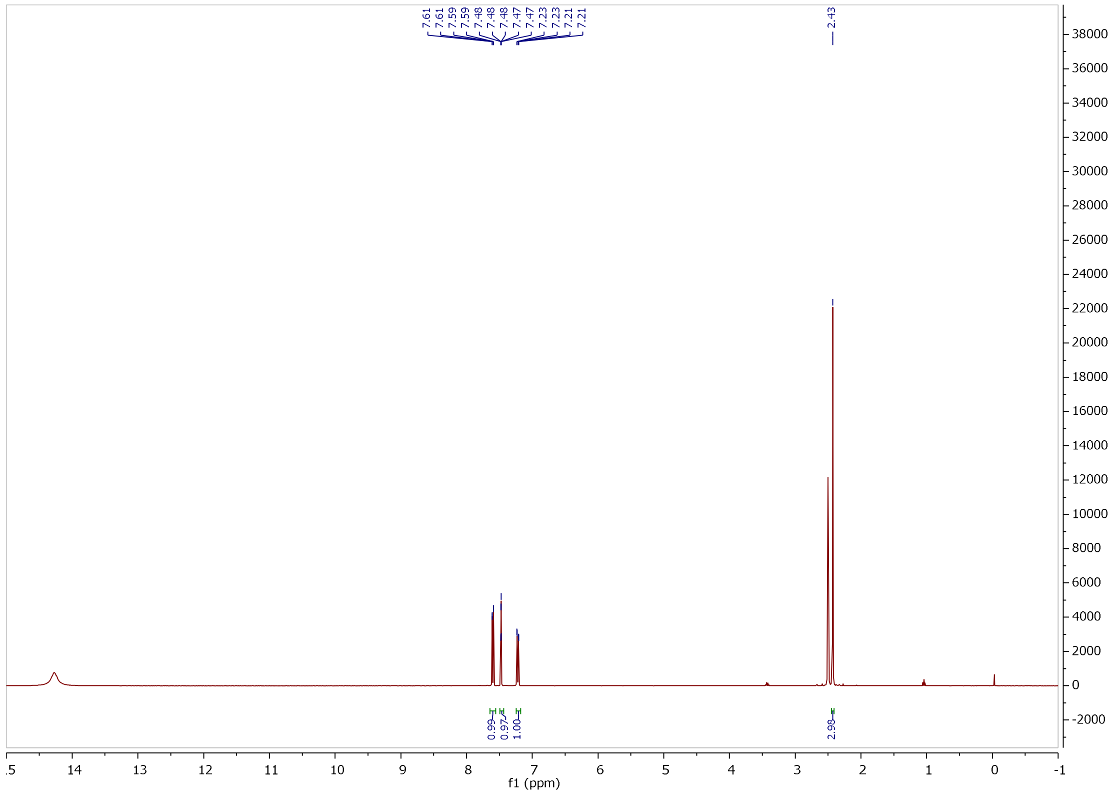
7.61
7.61
7.59
7.59
7.48
7.48
7.48
7.47
7.47
7.23
7.23
7.21
7.21
2.43
0.99
0.97
1.00
2.98
f1 (ppm)

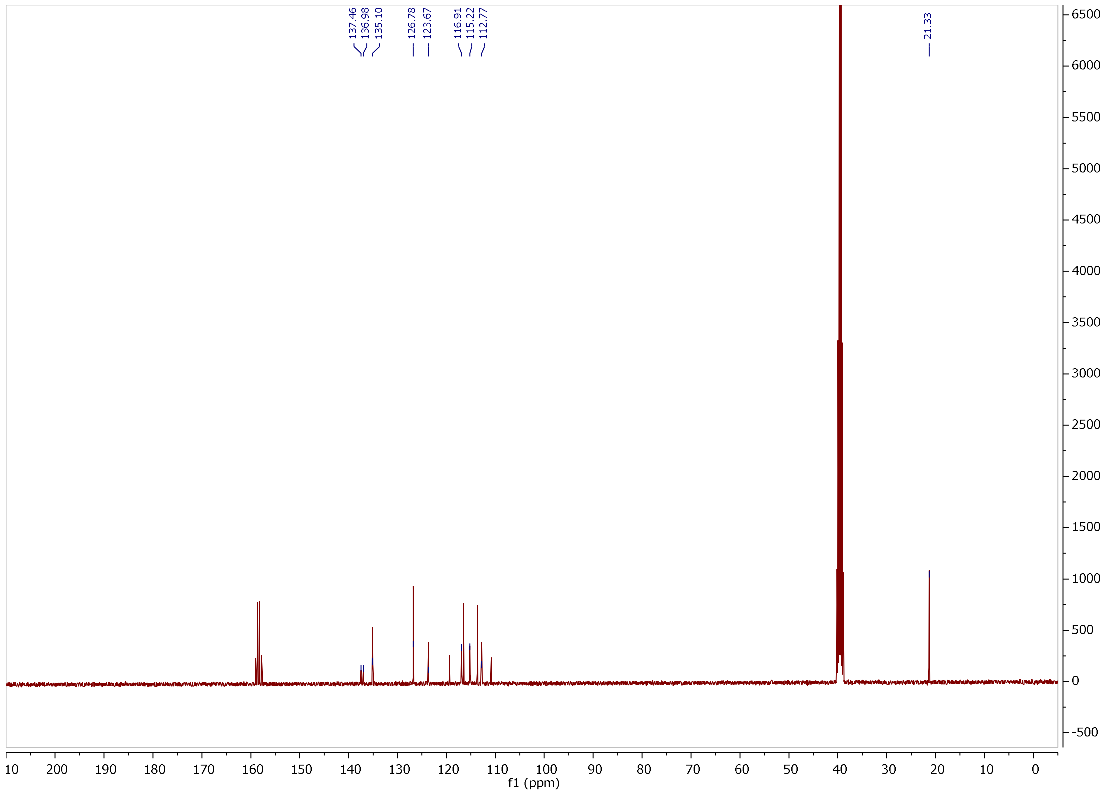
137.46
136.98
135.10
126.78
123.67
116.91
115.22
112.77
21.33
f1 (ppm)

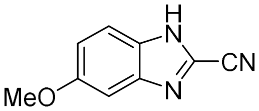

Compound 15

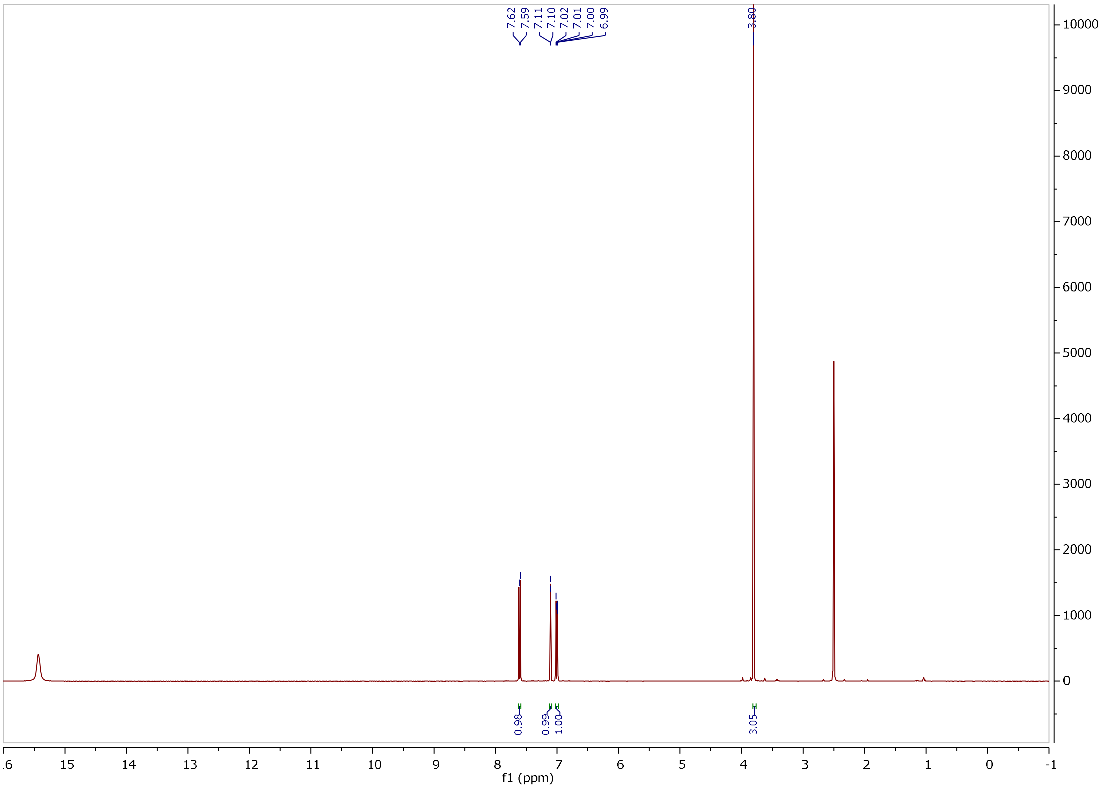

7.62
7.59
7.11
7.10
7.02
7.01
7.00
6.99
3.80
0.98
0.99
1.00
3.05
f1 (ppm)

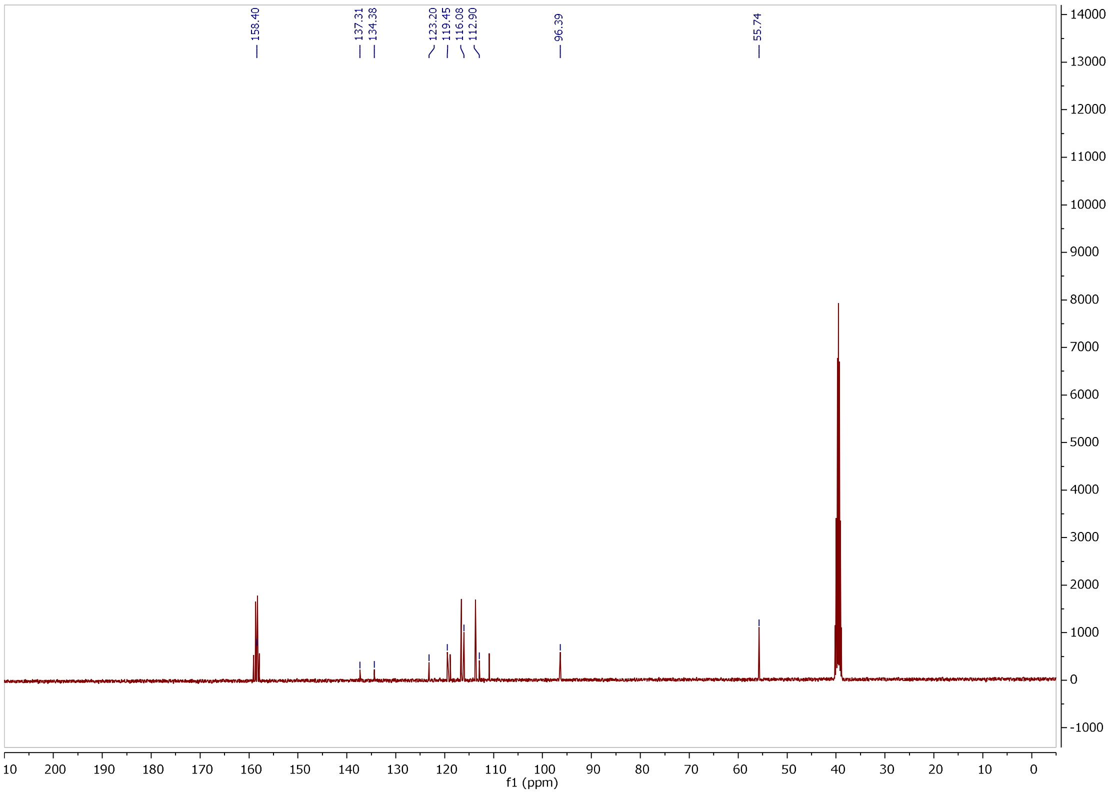

158.40
137.31
134.38
123.20
119.45
116.08
112.90
96.39
55.74
f1 (ppm)

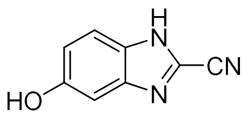
H
N
CN
HO
N

Compound 16

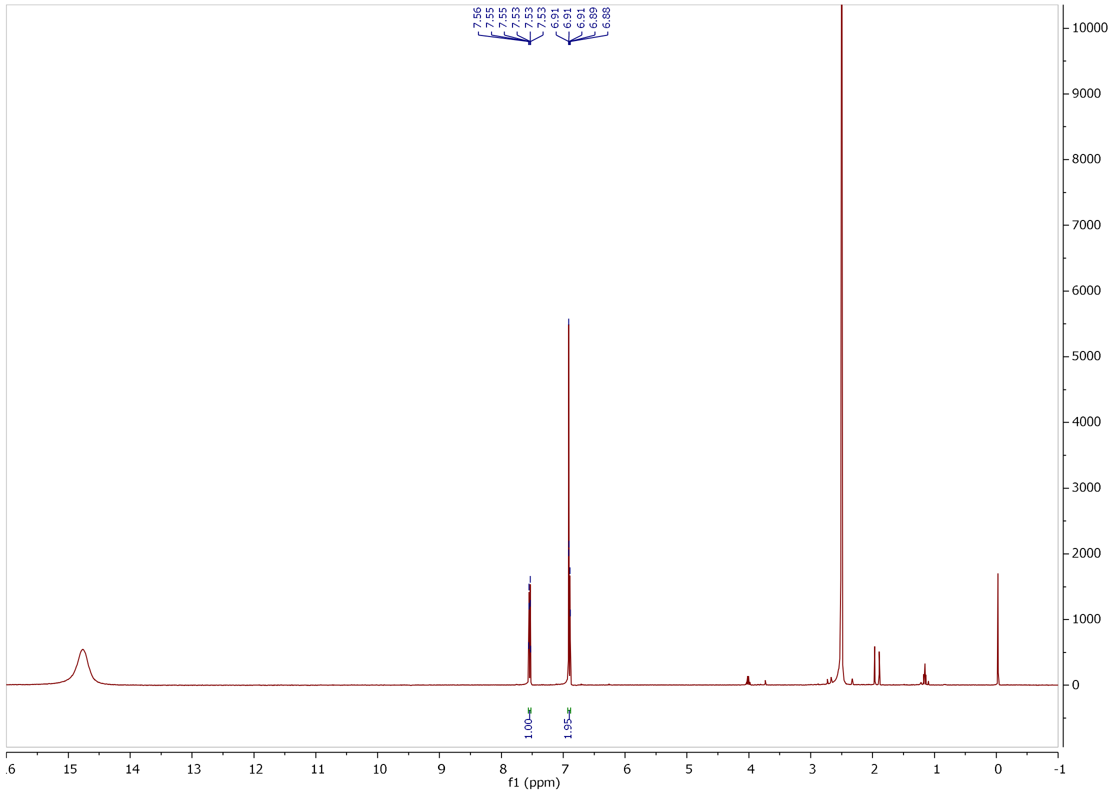
7.56
7.55
7.55
7.53
7.53
7.53
6.91
6.91
6.91
6.89
6.88
1.00
1.95
f1 (ppm)

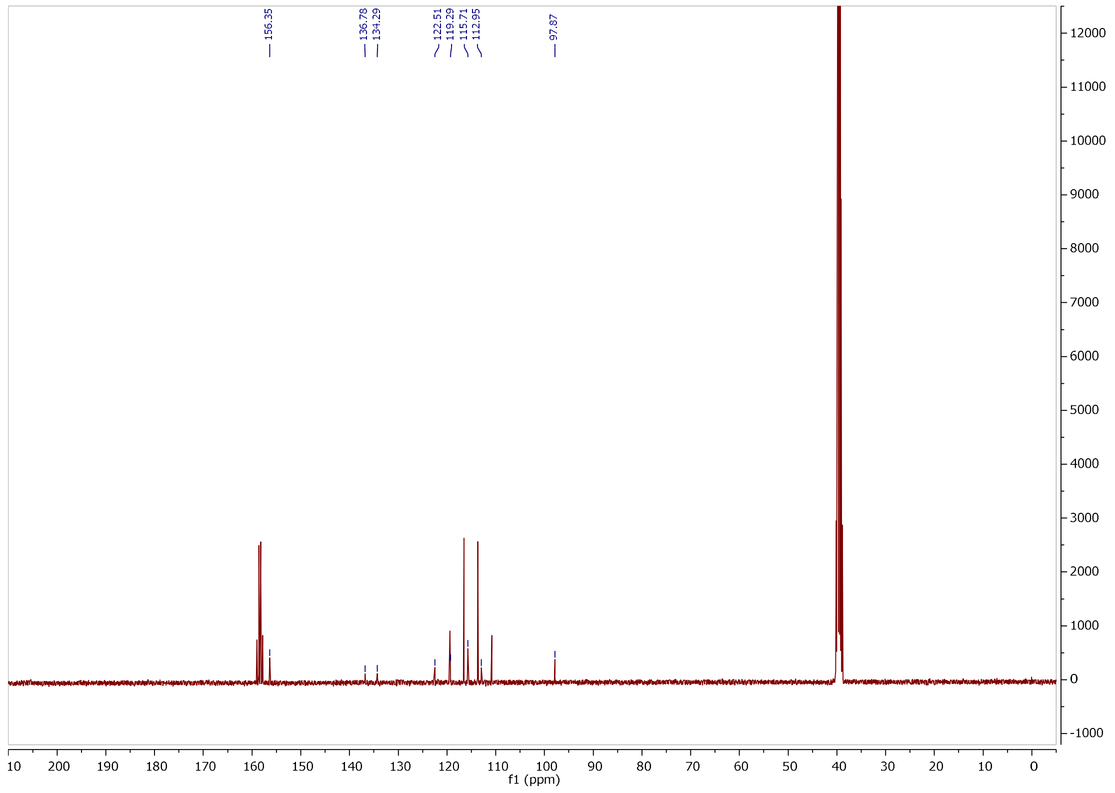
156.35
136.78
134.29
122.51
119.29
115.71
112.95
97.87
f1 (ppm)

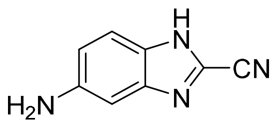
H
N
CN
H_2N
N

Compound 17

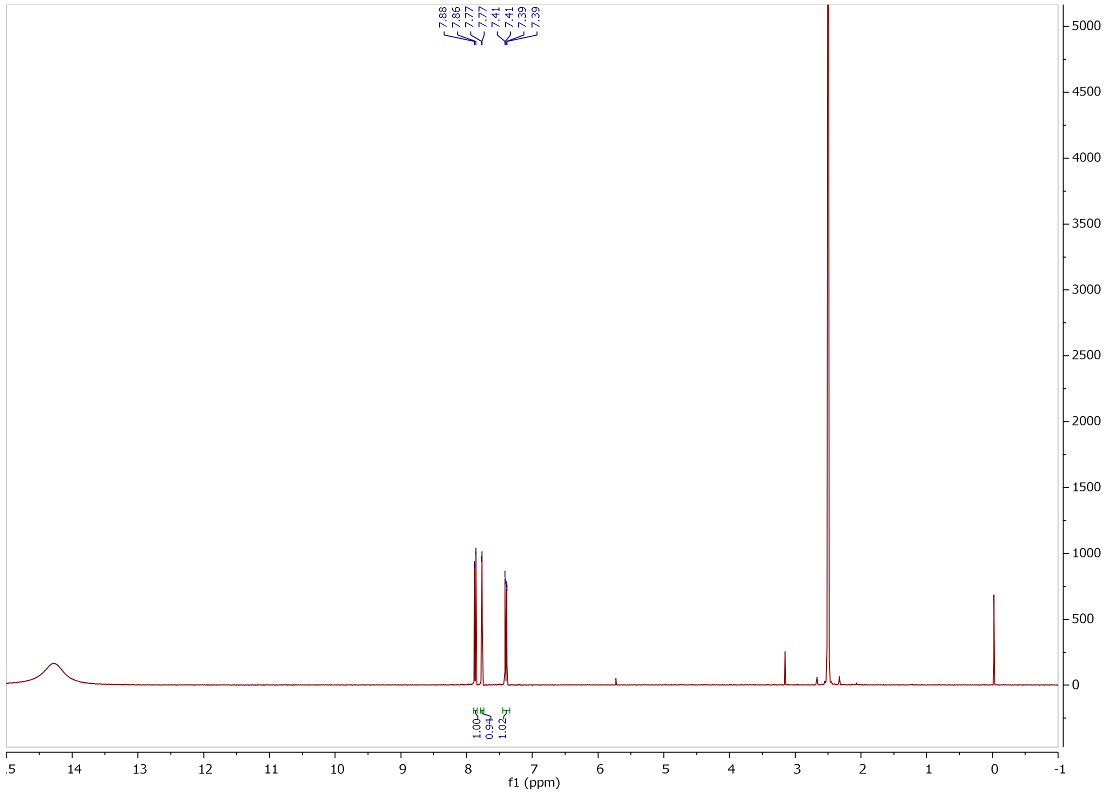
7.88
7.86
7.77
7.77
7.41
7.41
7.39
7.39
1.00
0.94
1.02
f1 (ppm)

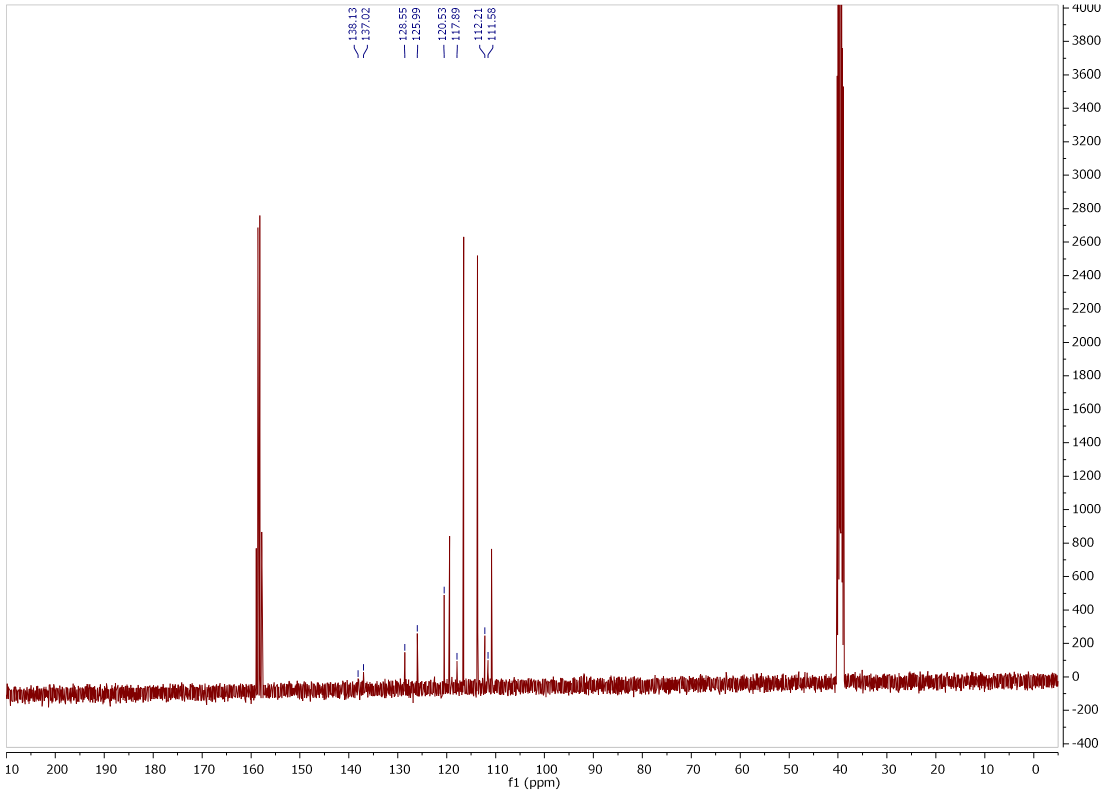
138.13
137.02
128.55
125.99
120.53
117.89
112.21
111.58
f1 (ppm)

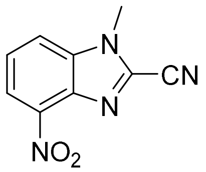

Compound 19

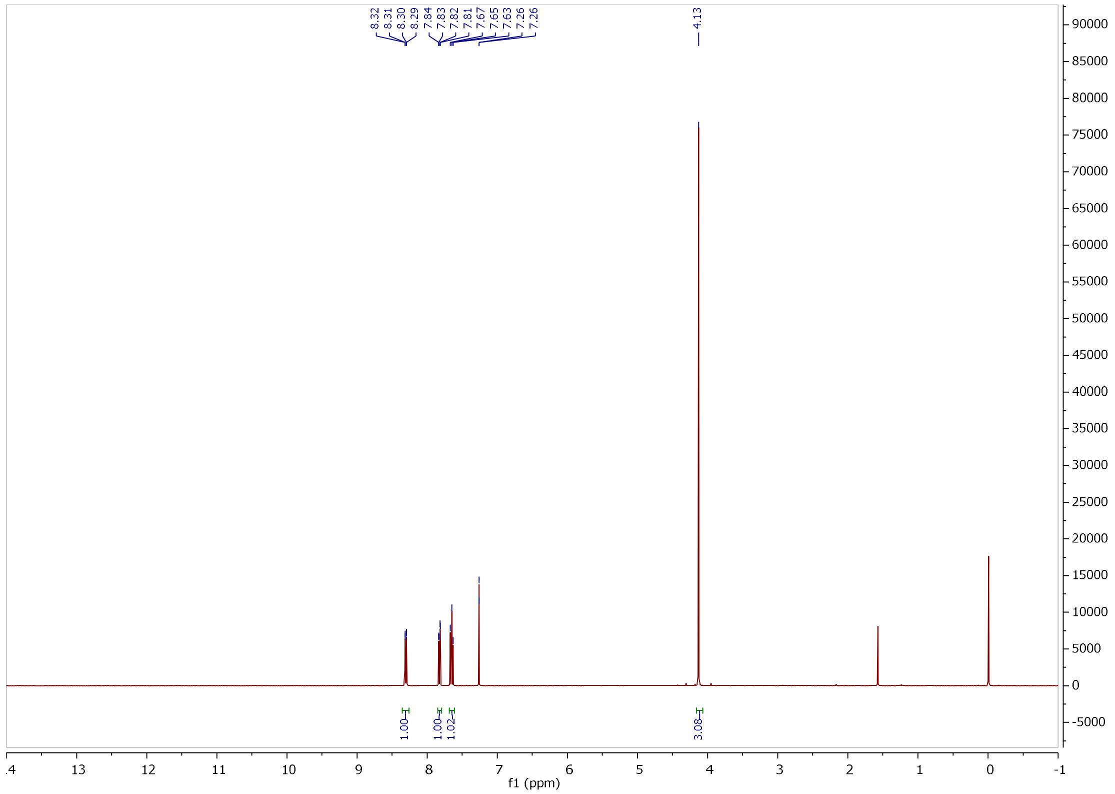

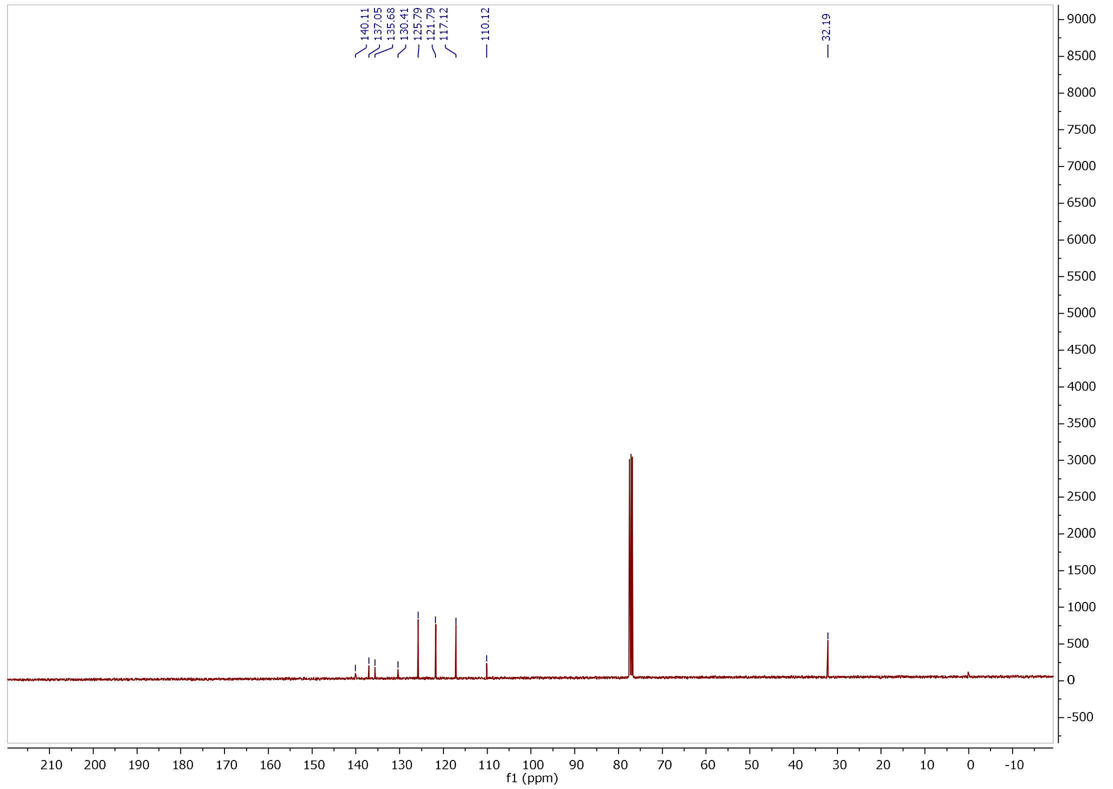

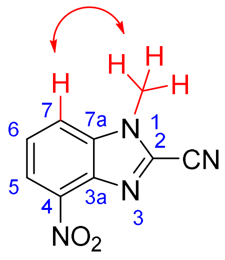

H
7
7a
N
1
2
CN
6
5
4
3a
N
3
NO2

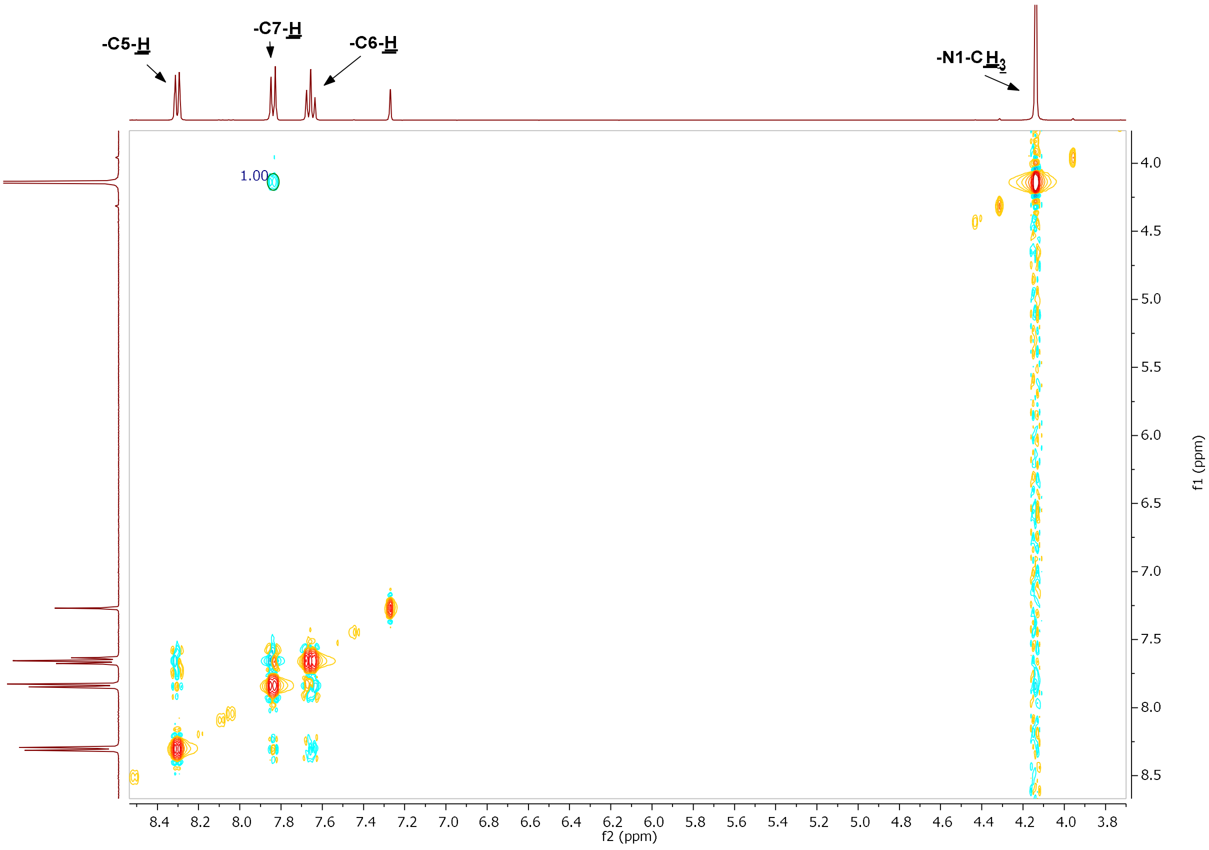

-C5-H
-C7-H
-C6-H
-N1-CH3
1.00
f2 (ppm)
f1 (ppm)

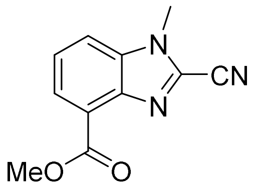

Compound 20

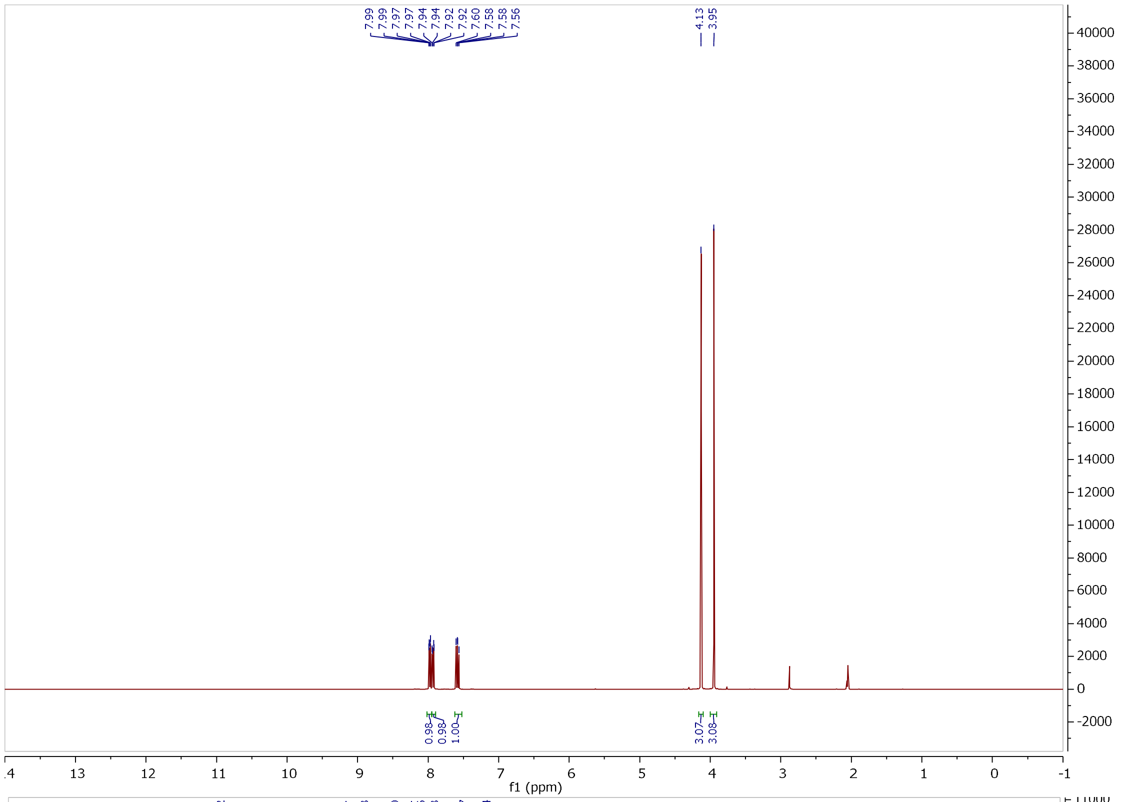

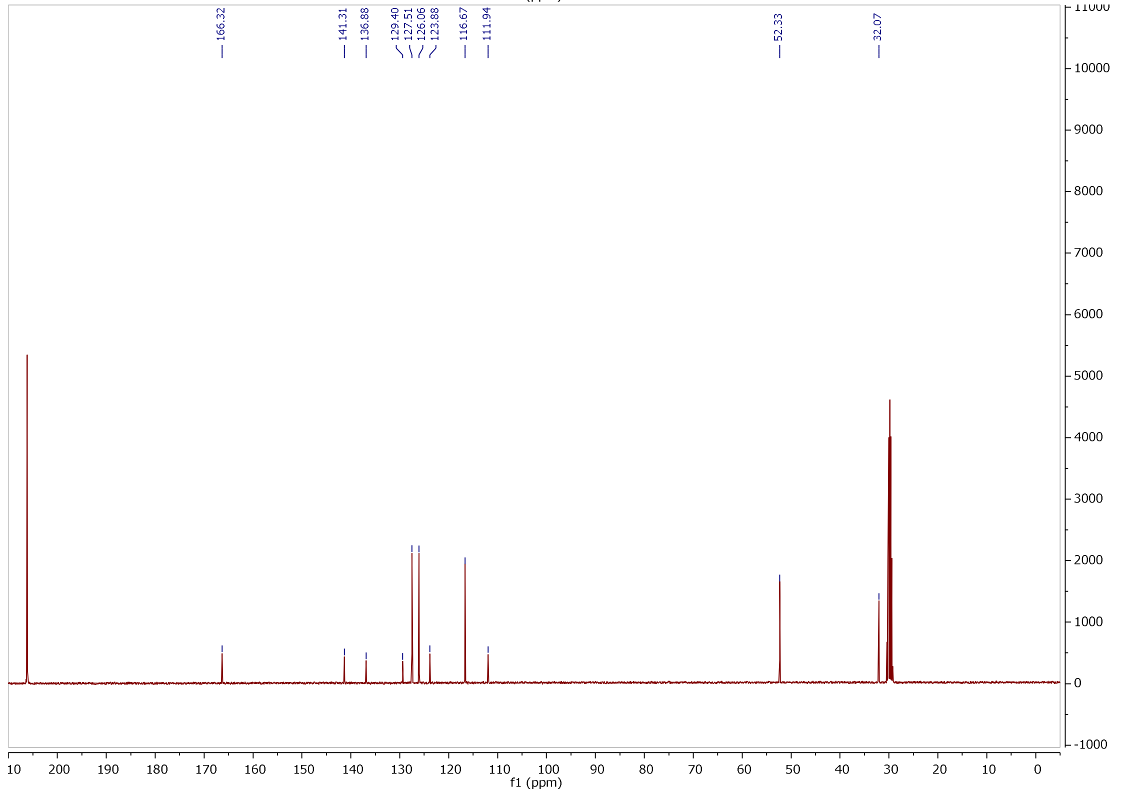

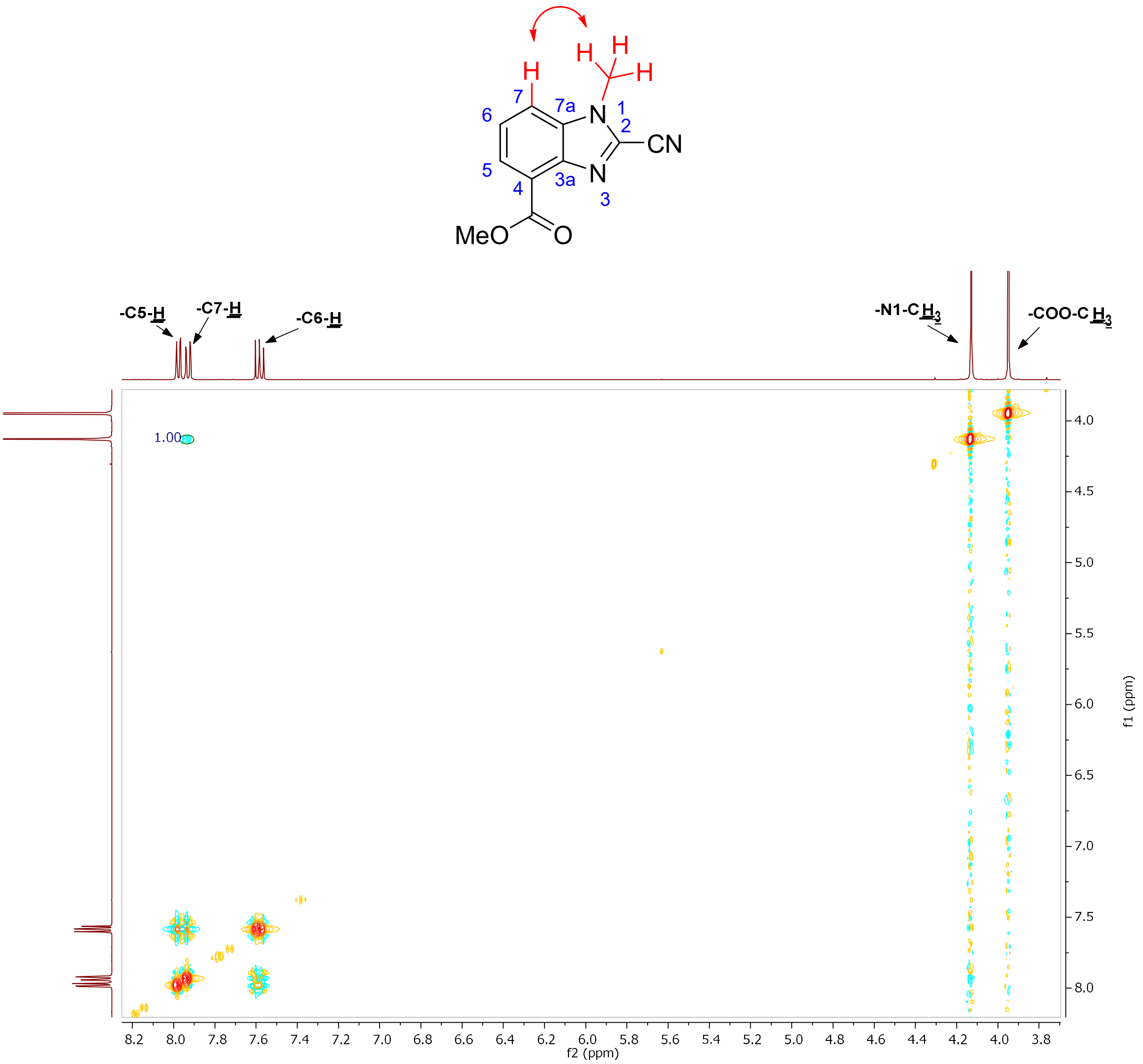
-C5-H
-C7-H
-C6-H
-N1-CH3
-COO-CH3
1.00
f2 (ppm)
f1 (ppm)

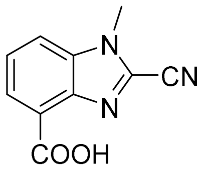
N
CN
N
COOH

Compound 21

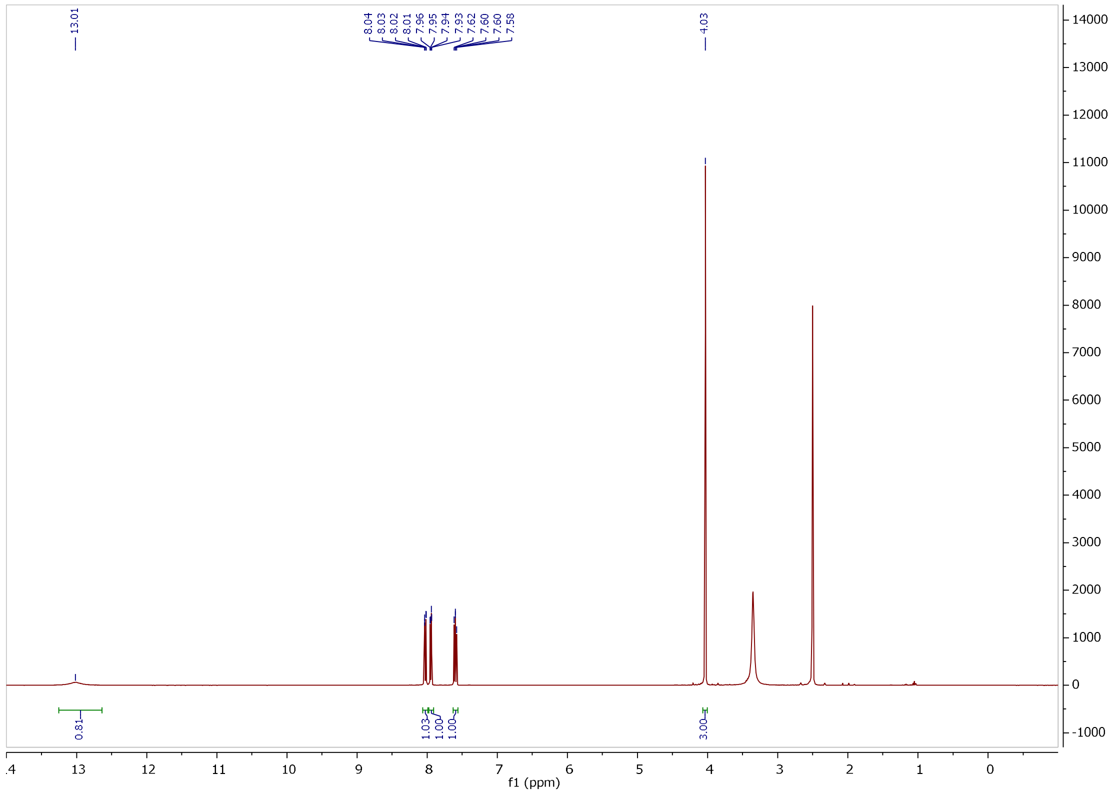
13.01
8.04
8.03
8.02
8.01
7.96
7.95
7.94
7.93
7.62
7.60
7.60
7.58
4.03
0.81
1.03
1.00
1.00
3.00
f1 (ppm)

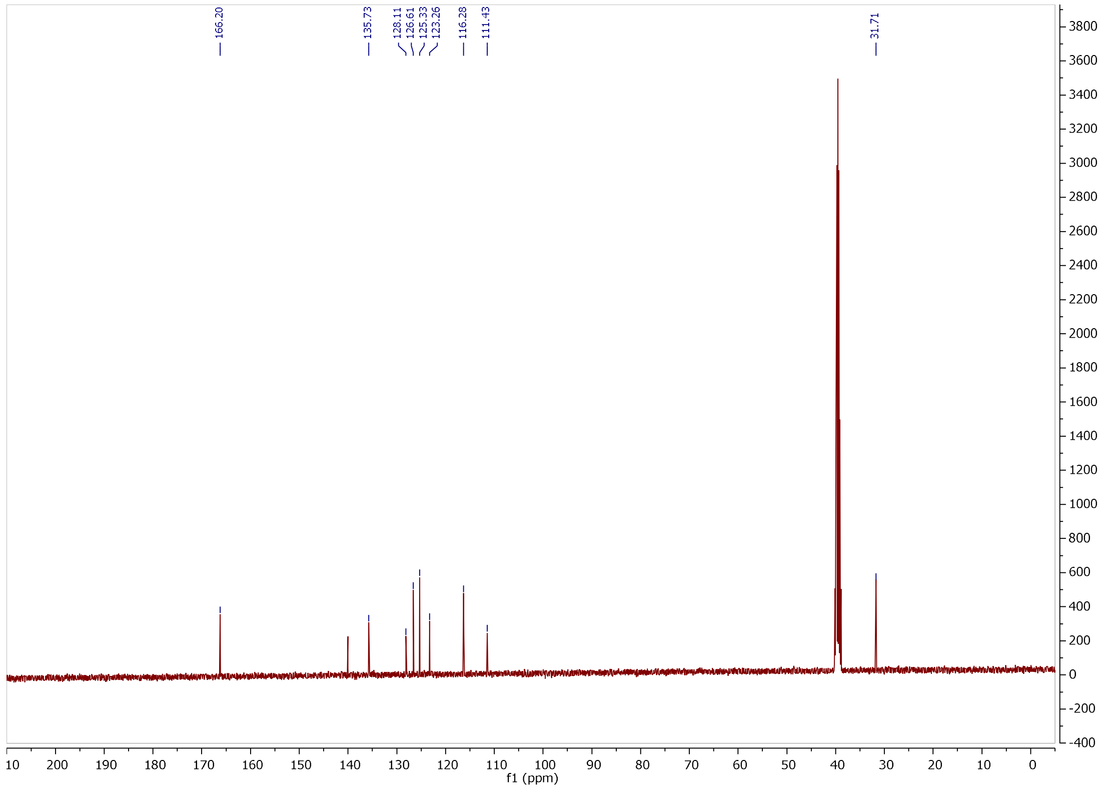
166.20
135.73
128.11
126.61
125.33
123.26
116.28
111.43
31.71
f1 (ppm)

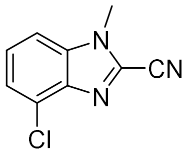
N
CN
N
Cl

Compound 22

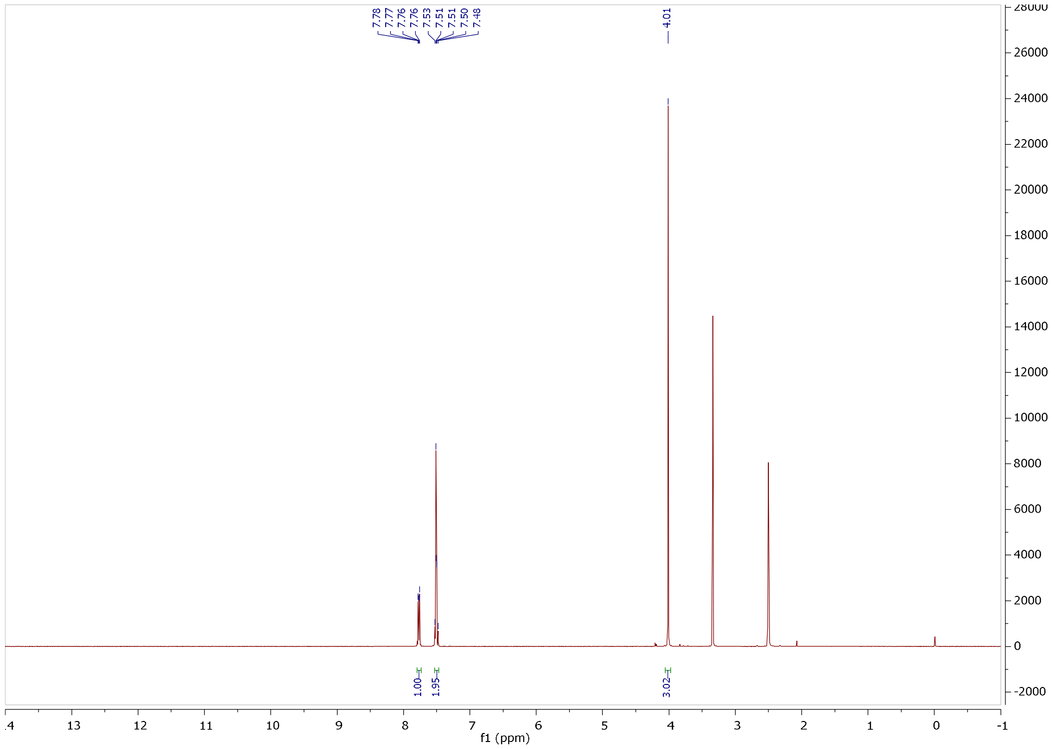
7.78
7.77
7.76
7.76
7.53
7.51
7.51
7.50
7.48
4.01
1.00
1.95
3.02
f1 (ppm)

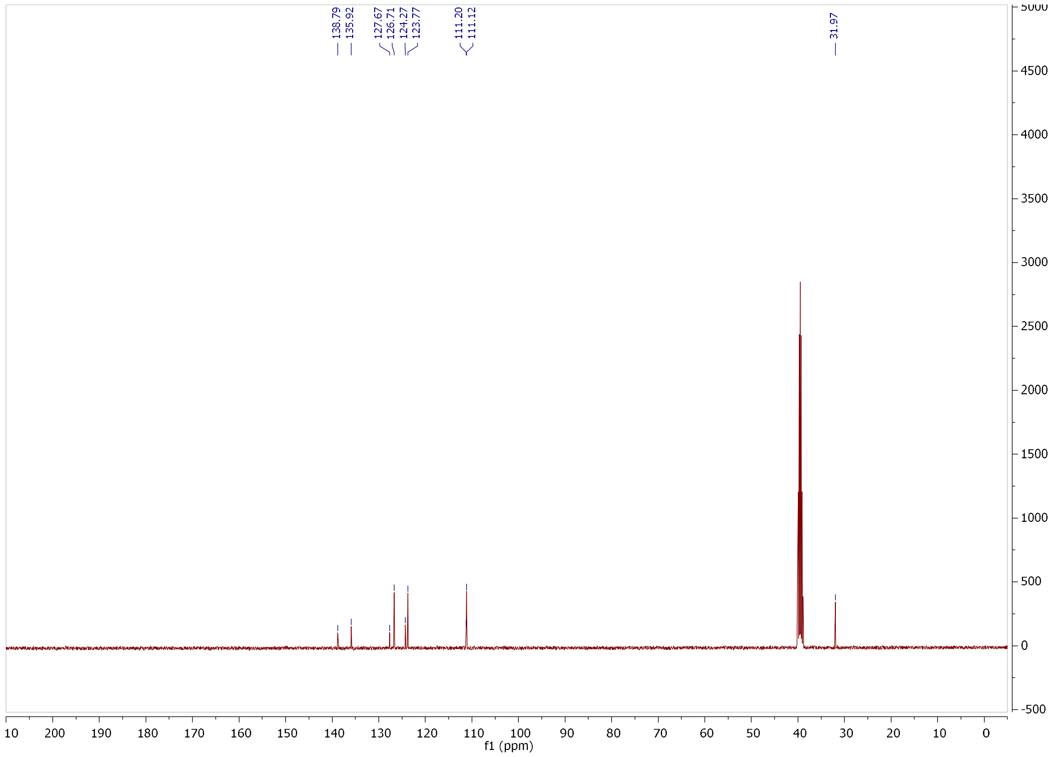
138.79
135.92
127.67
126.71
124.27
123.77
111.20
111.12
31.97
f1 (ppm)

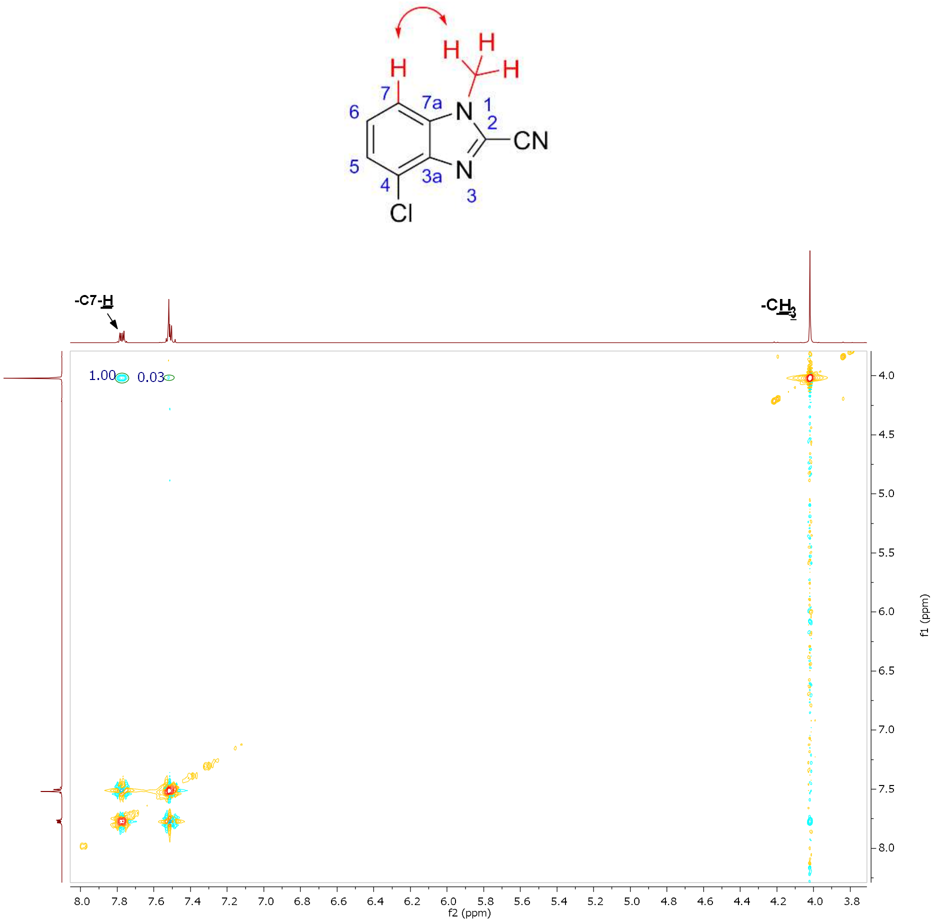

H
H
H
H
7
7a
1
6
N
2
CN
5
N
4
3a
3
Cl
-C7-H
-CH3
1.00
0.03
f2 (ppm)
f1 (ppm)

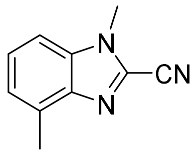

Compound 23

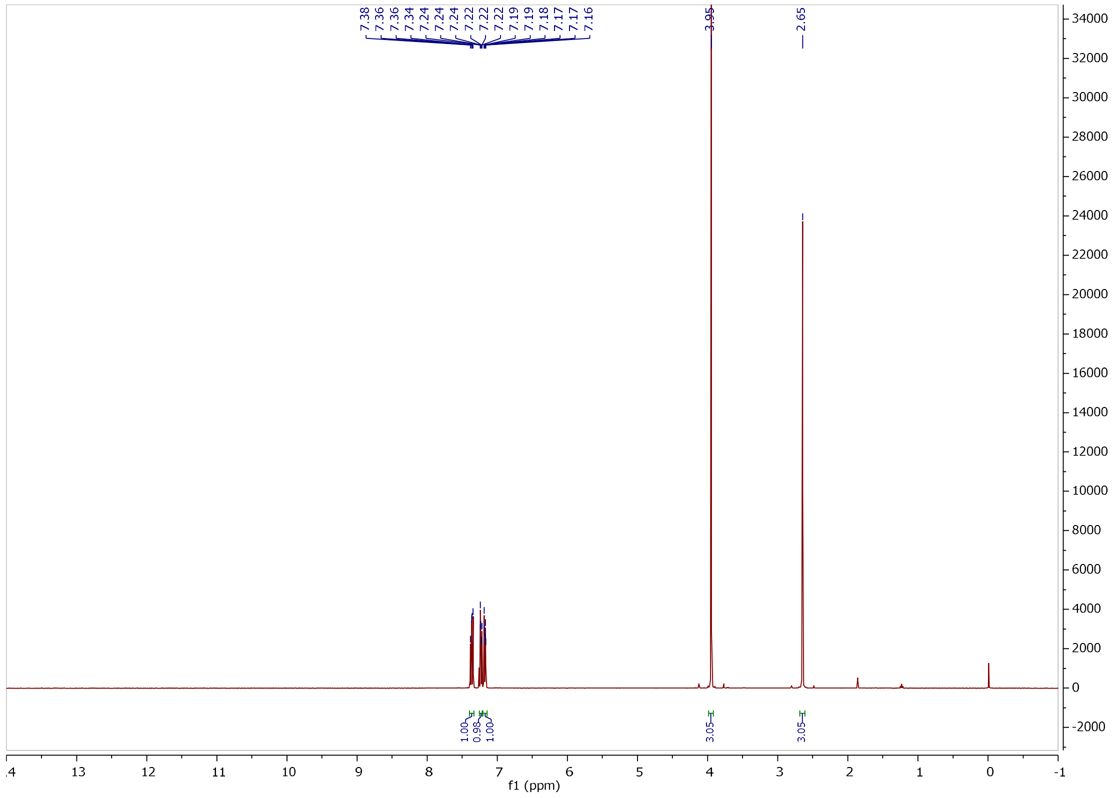

7.38
7.36
7.36
7.34
7.24
7.24
7.24
7.22
7.22
7.22
7.19
7.19
7.18
7.17
7.17
7.16
3.95
2.65
1.00
0.98
1.00
3.05
3.05
f1 (ppm)

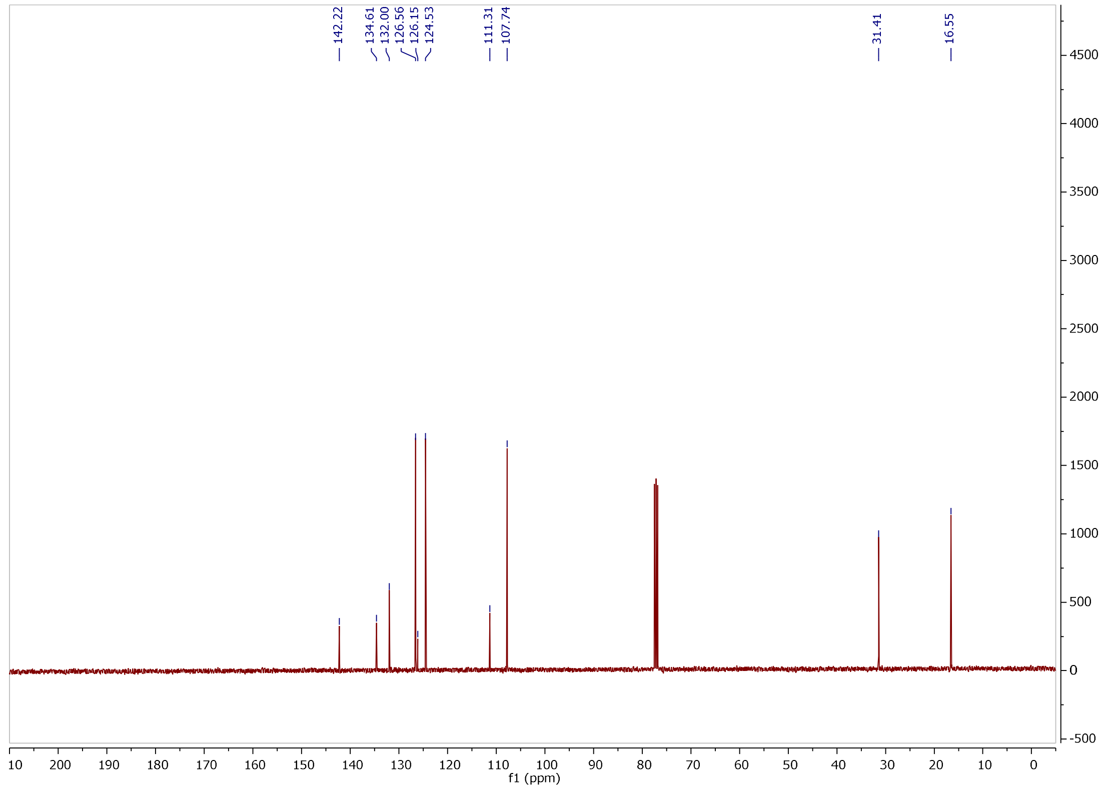

142.22
134.61
132.00
126.56
126.15
124.53
111.31
107.74
31.41
16.55
f1 (ppm)

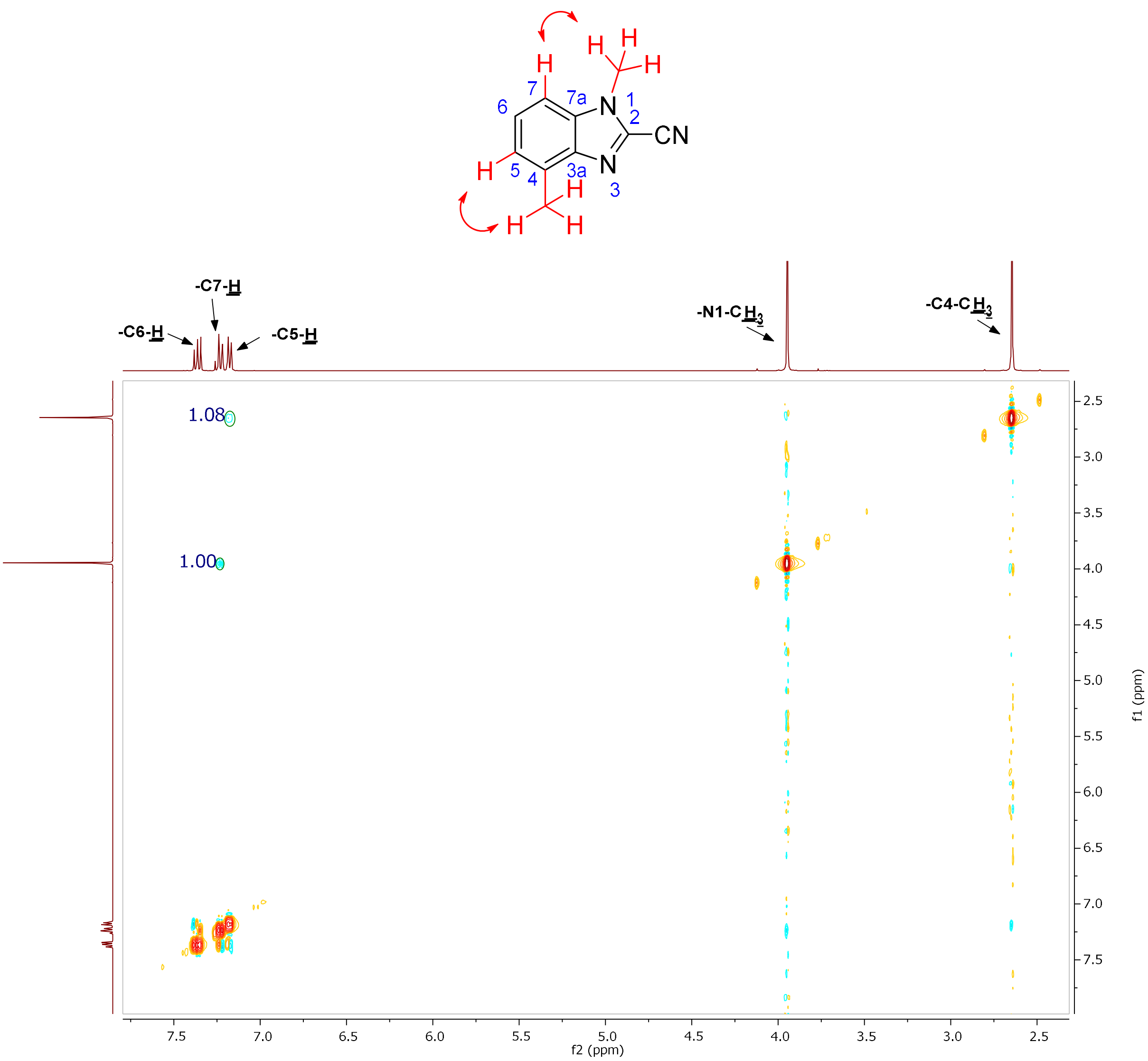

H
7
6
7a
N
1
2
CN
H
5
4
3a
N
3
-C6-H
-C7-H
-C5-H
-N1-CH3
-C4-CH3
1.08
1.00
f2 (ppm)
f1 (ppm)

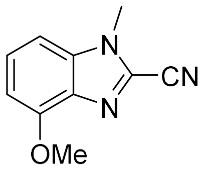
N
CN
N
OMe

Compound 24

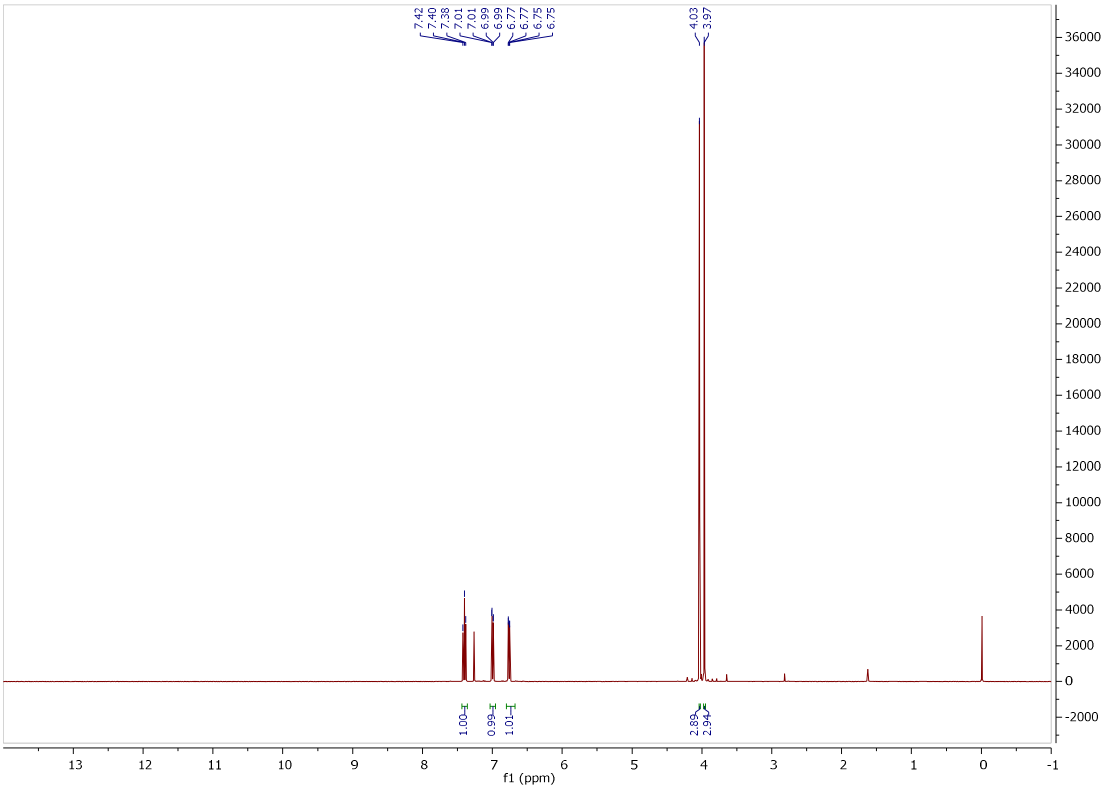
7.42
7.40
7.38
7.01
7.01
6.99
6.99
6.77
6.77
6.75
6.75
4.03
3.97
1.00
0.99
1.01
2.89
2.94
f1 (ppm)

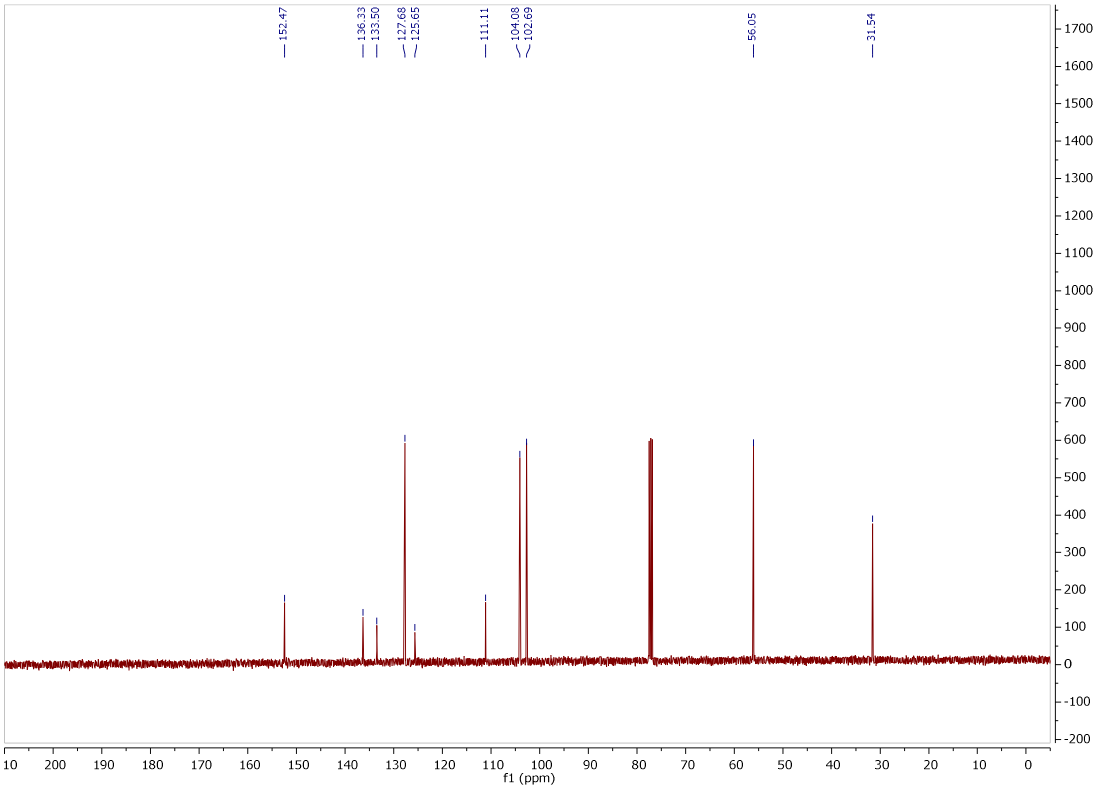
152.47
136.33
133.50
127.68
125.65
111.11
104.08
102.69
56.05
31.54
f1 (ppm)

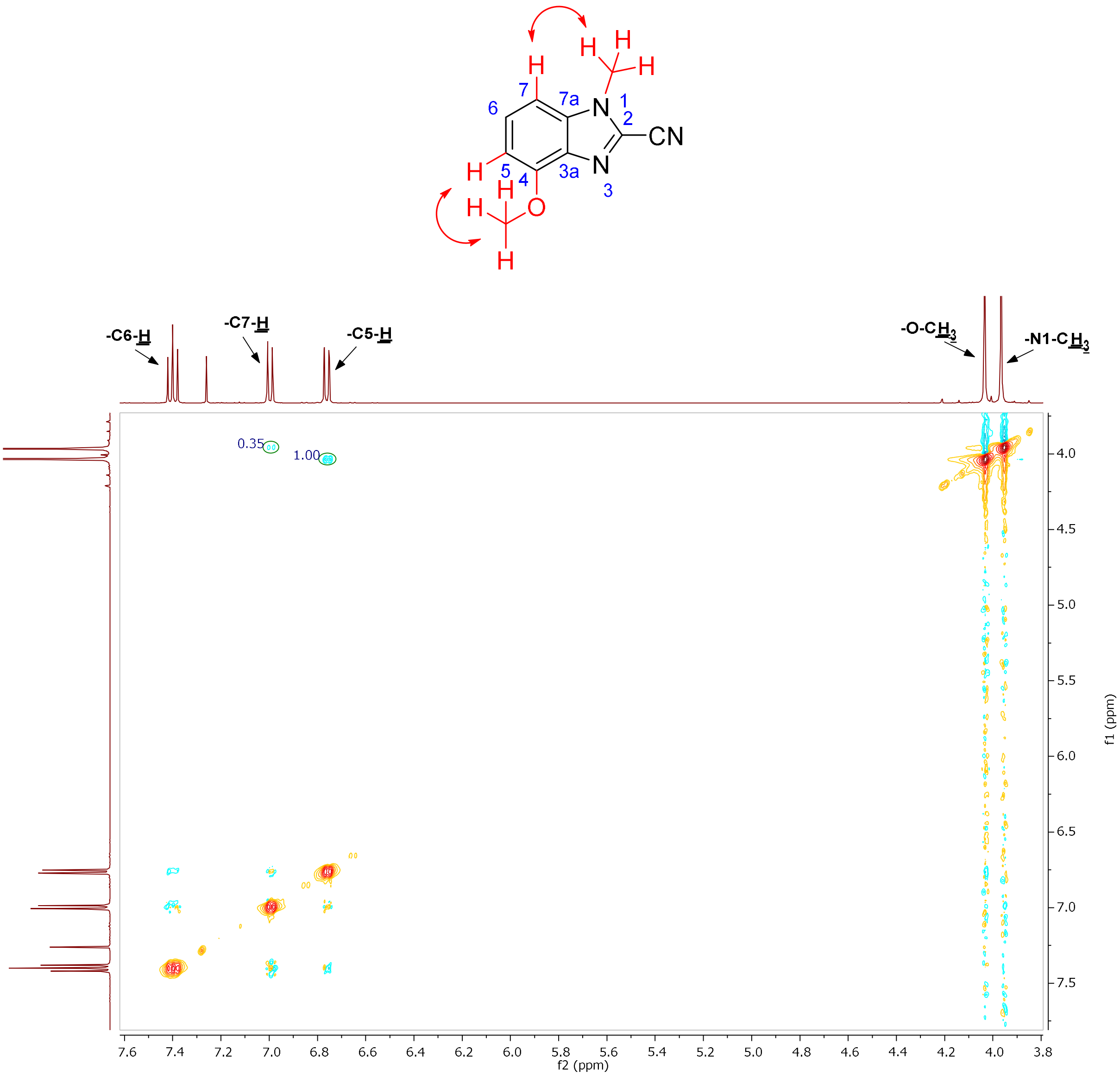

-C6-H
-C7-H
-C5-H
-O-CH3
-N1-CH3
0.35
1.00
f2 (ppm)
f1 (ppm)

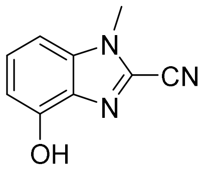

Compound 25

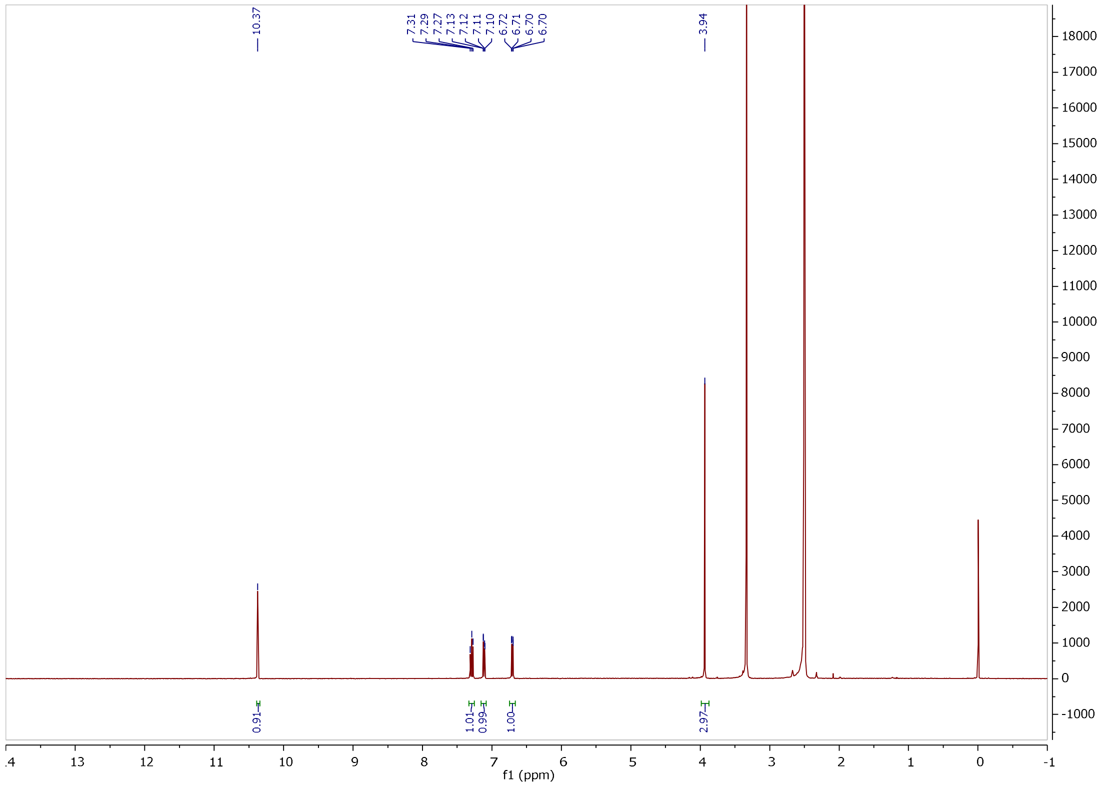

10.37
7.31
7.29
7.27
7.13
7.12
7.11
7.10
6.72
6.71
6.70
6.70
3.94
0.91
1.01
0.99
1.00
2.97
f1 (ppm)

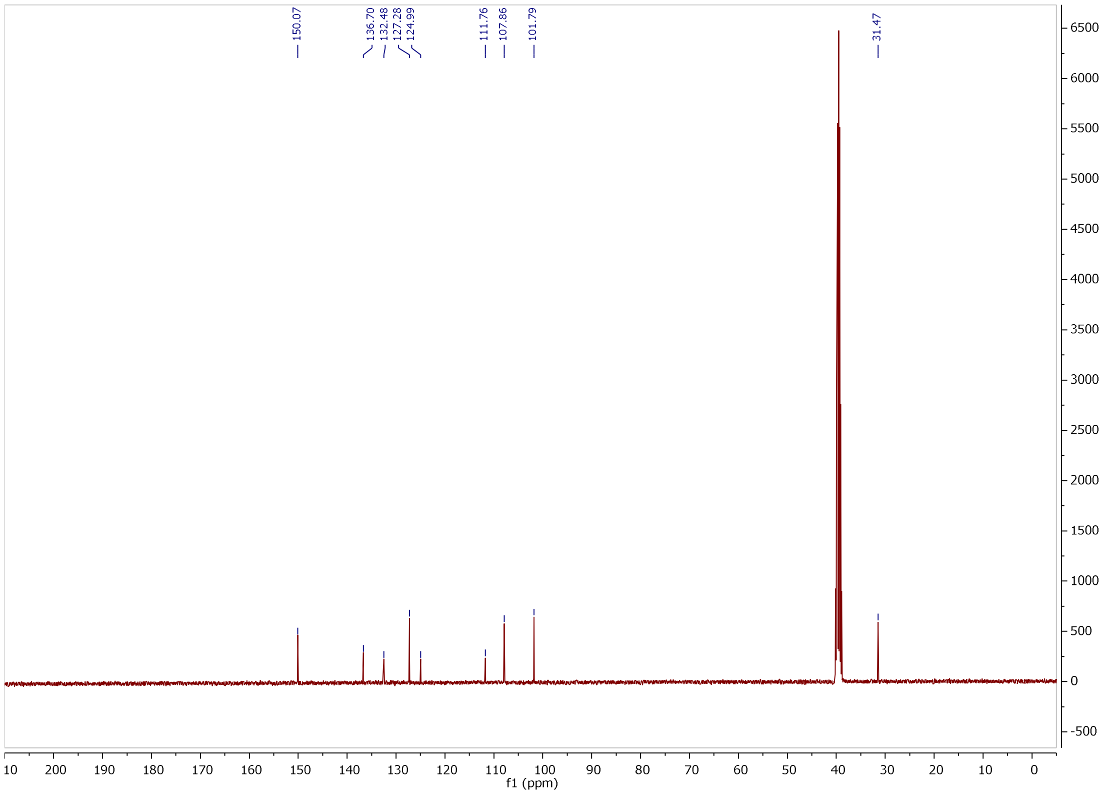

150.07
136.70
132.48
127.28
124.99
111.76
107.86
101.79
31.47
f1 (ppm)

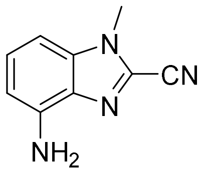

Compound 26

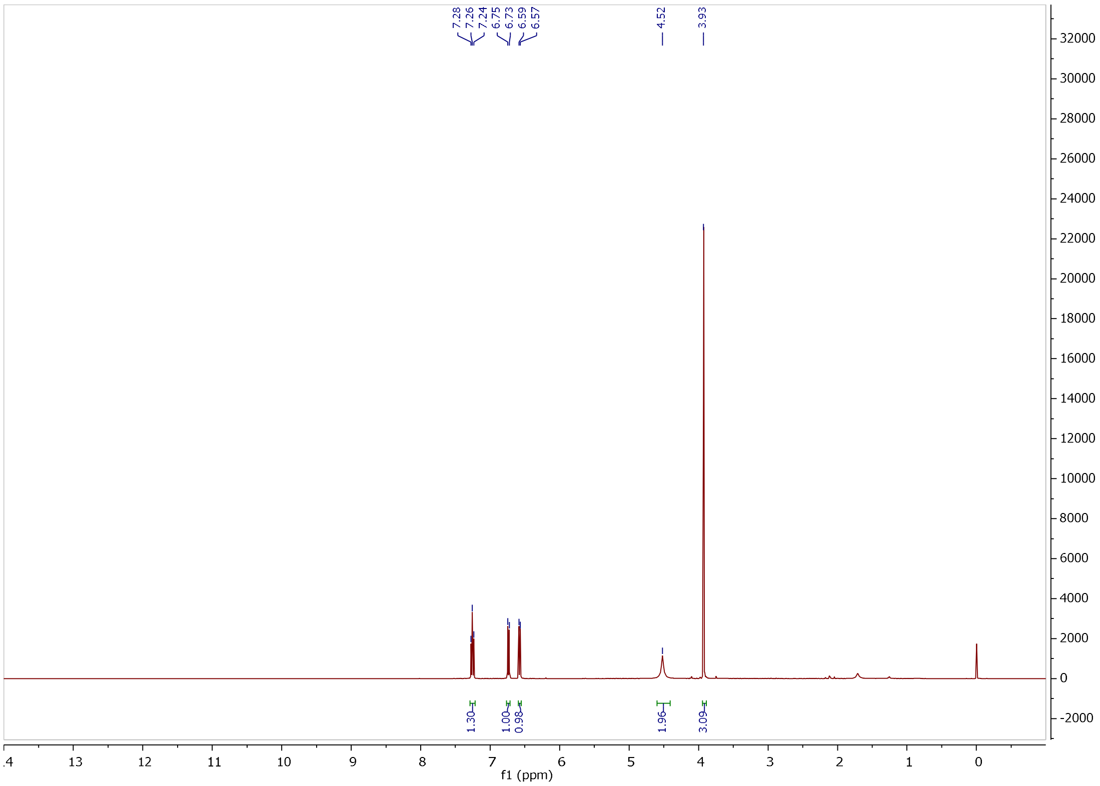

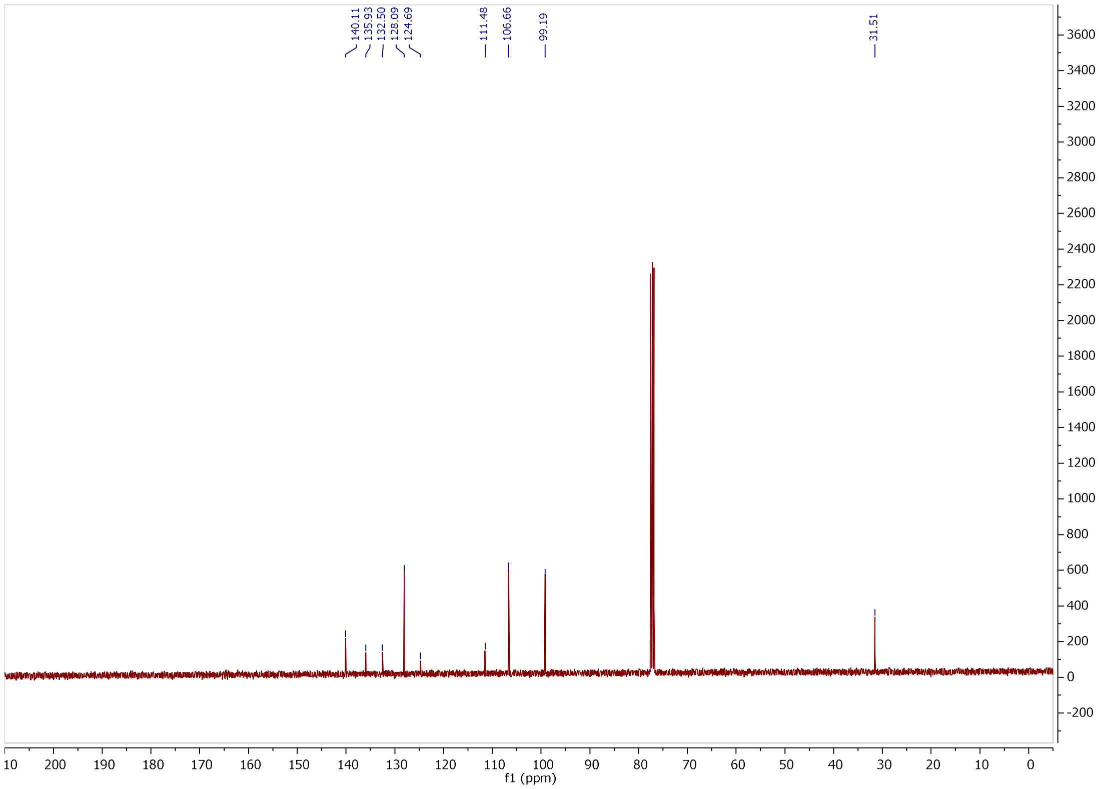

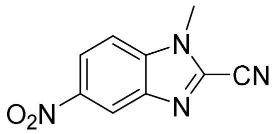

Compound 27

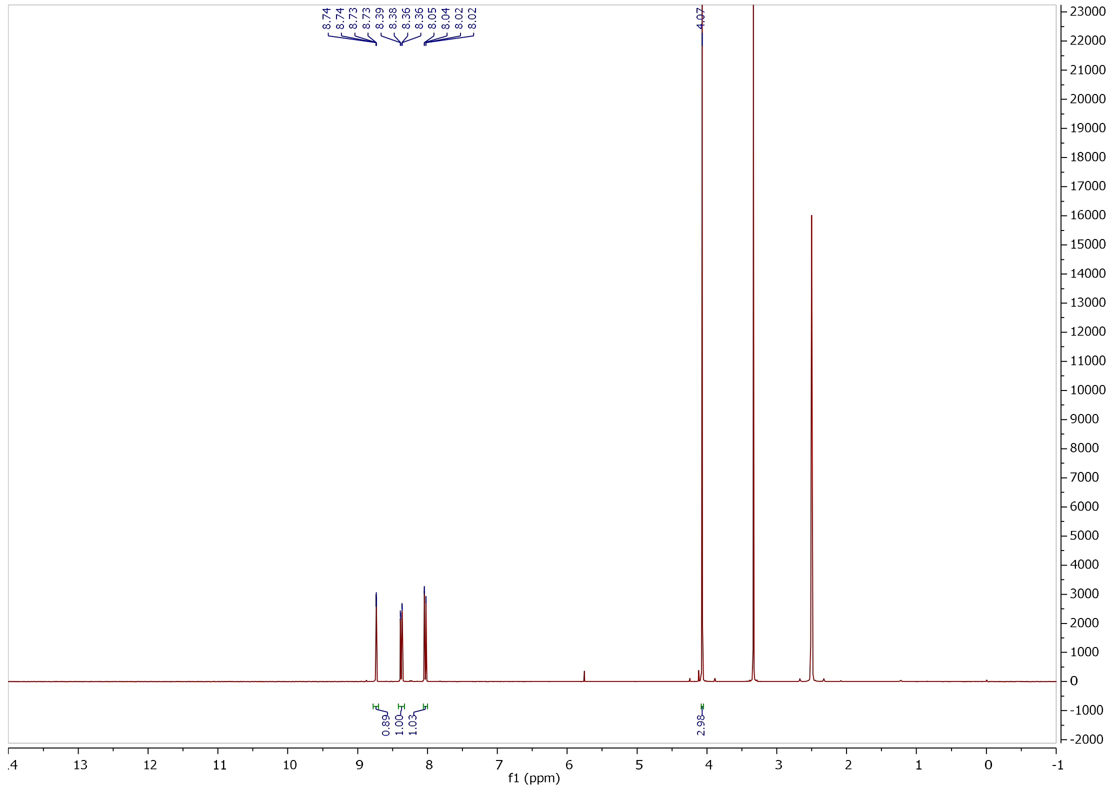
8.74
8.74
8.73
8.73
8.39
8.38
8.36
8.36
8.05
8.04
8.02
8.02
4.07
0.89
1.00
1.03
2.98
f1 (ppm)

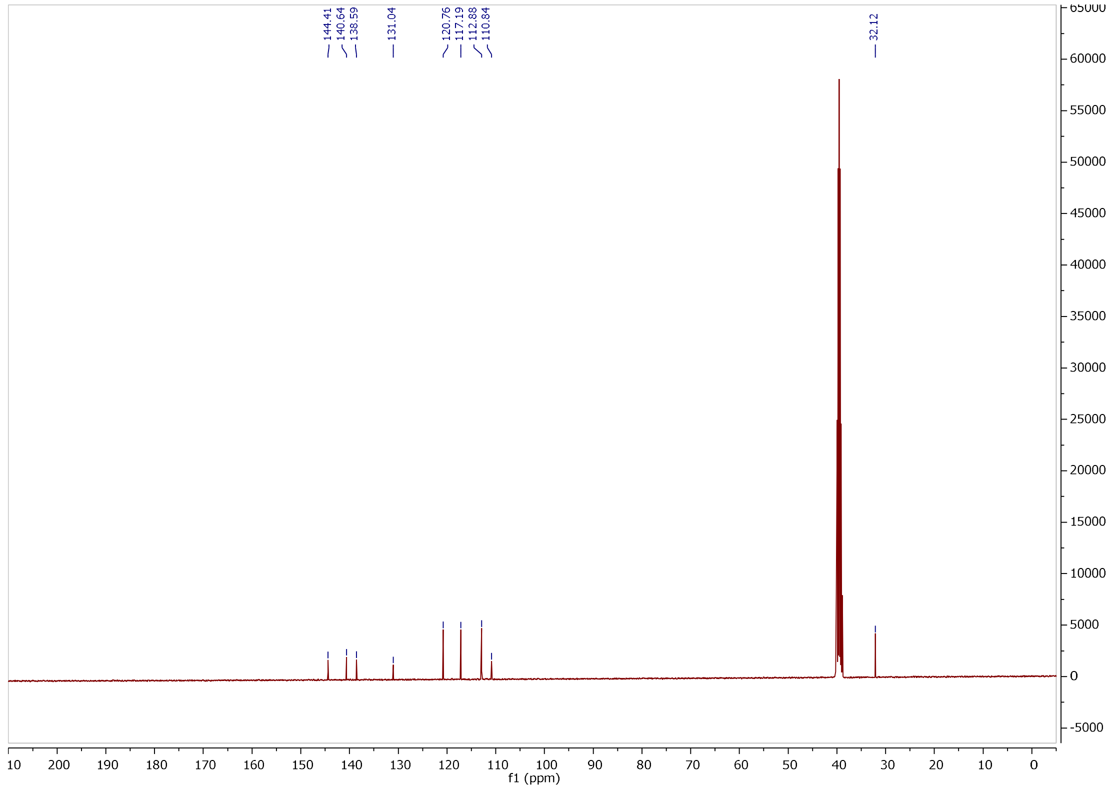
144.41
140.64
138.59
131.04
120.76
117.19
112.88
110.84
32.12
f1 (ppm)

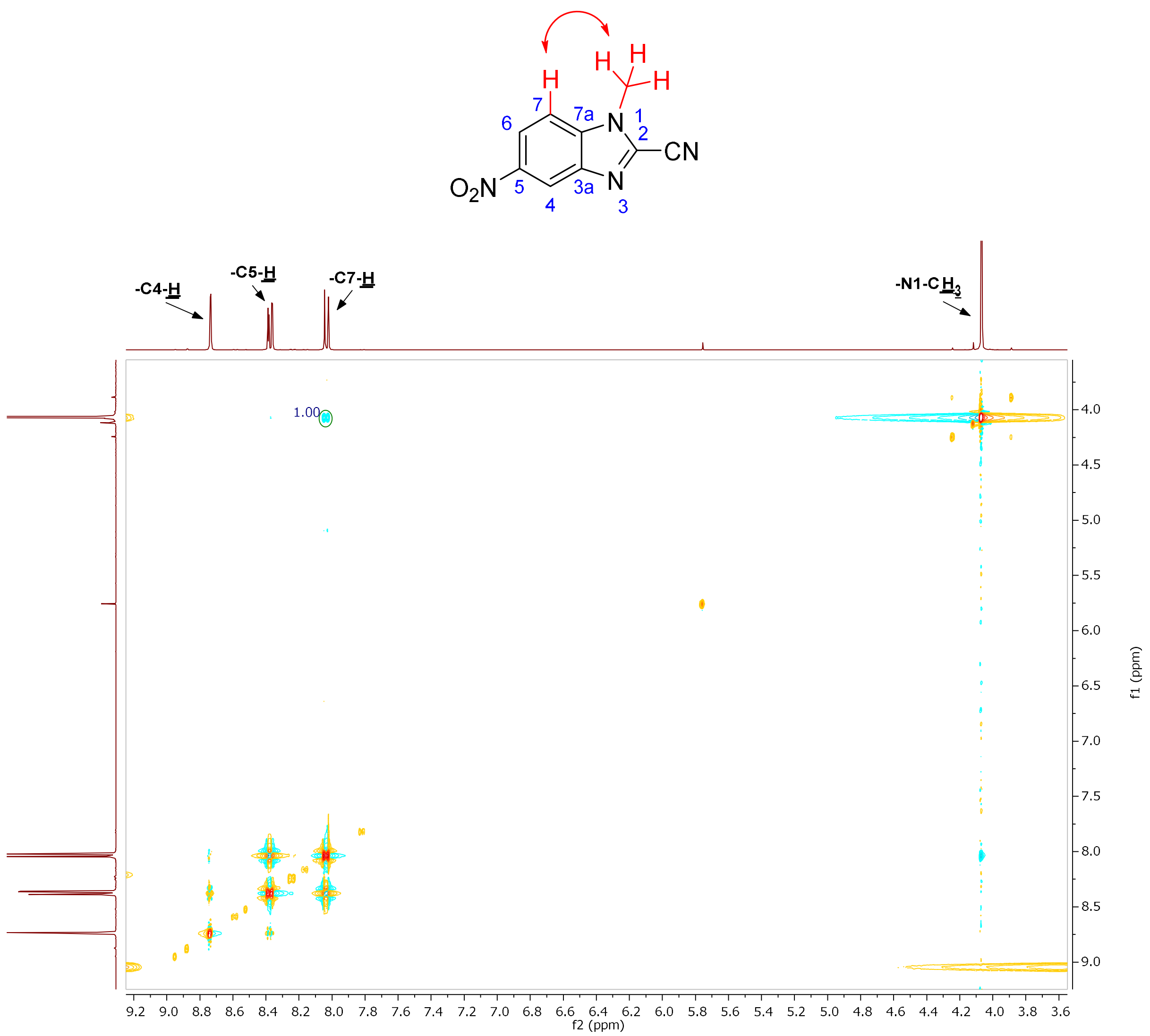
7
7a
1
2
6
CN
O2N
5
4
3a
3
-C4-H
-C5-H
-C7-H
-N1-CH3
1.00
f2 (ppm)
f1 (ppm)

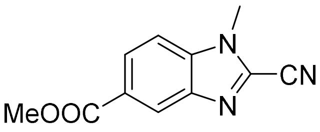

Compound 28

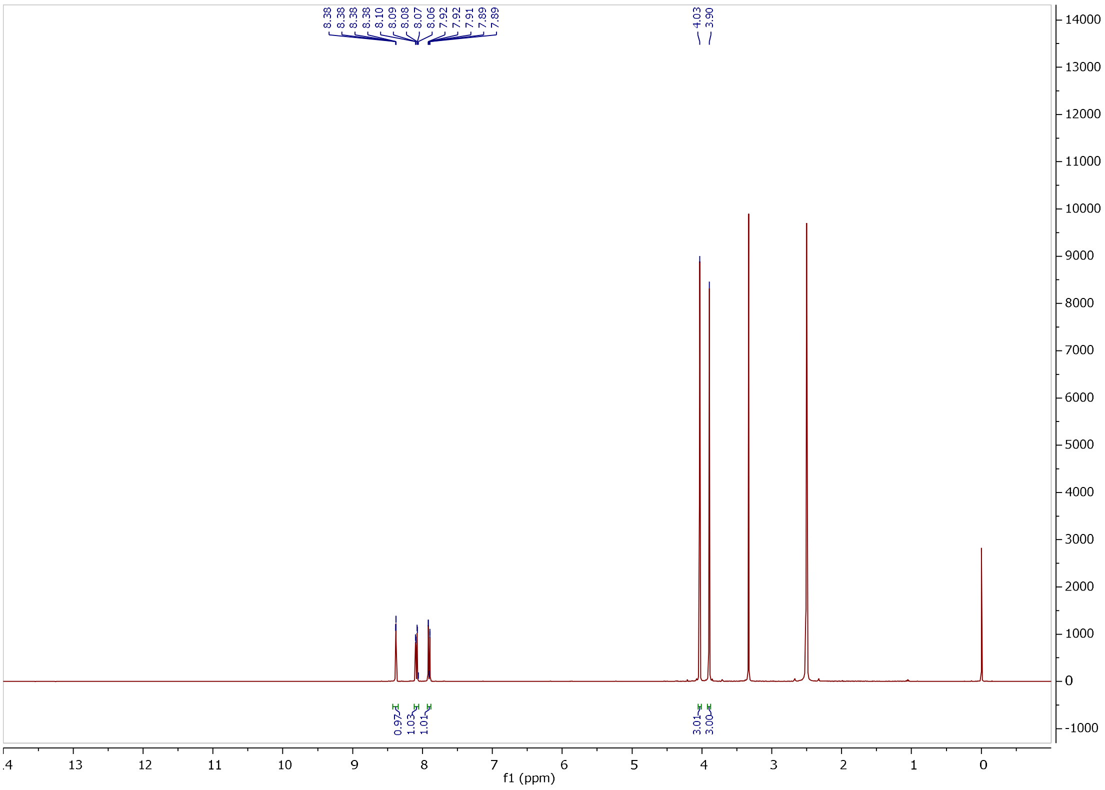

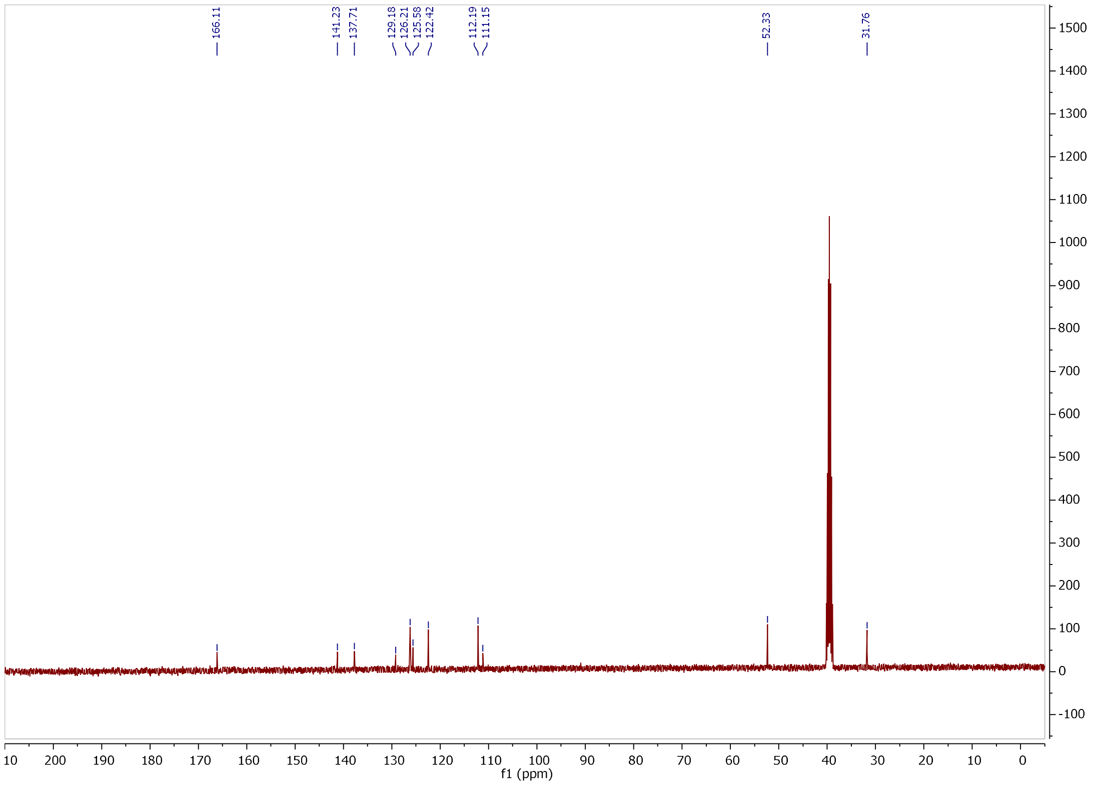

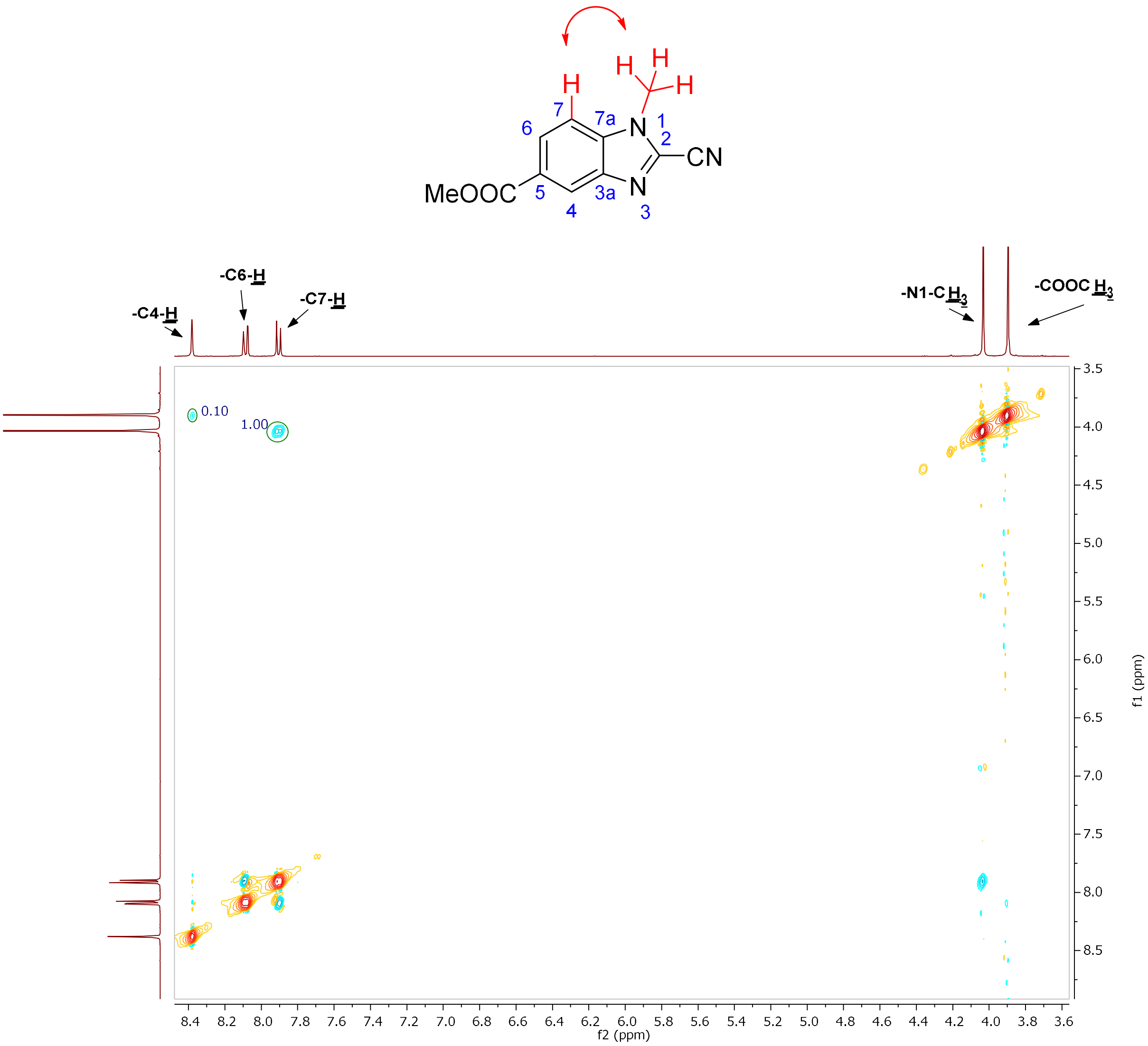
MeOOC
CN
-C4-H
-C6-H
-C7-H
-N1-CH3
-COOCH3
0.10
1.00
f2 (ppm)
f1 (ppm)

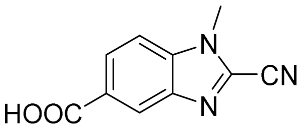

HOOC
N
N
CN

Compound 29

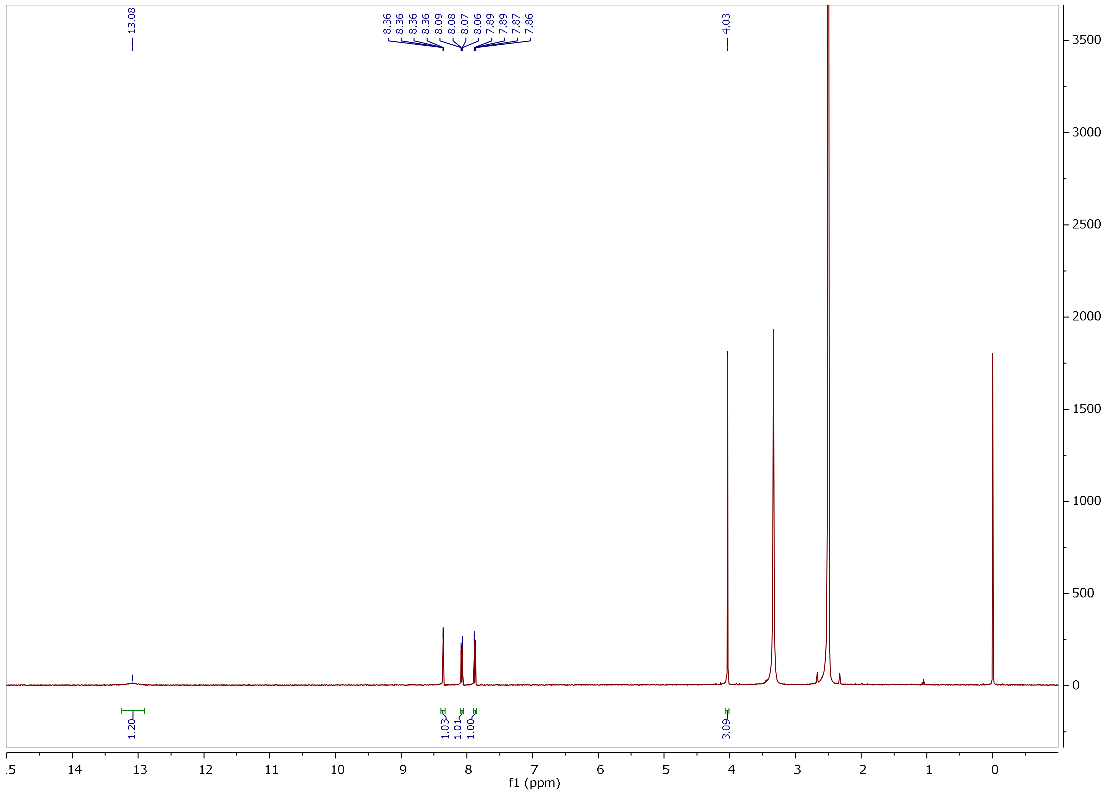

13.08
8.36
8.36
8.36
8.36
8.09
8.08
8.07
8.06
7.89
7.89
7.87
7.86
4.03
1.20
1.03
1.01
1.00
3.09
f1 (ppm)

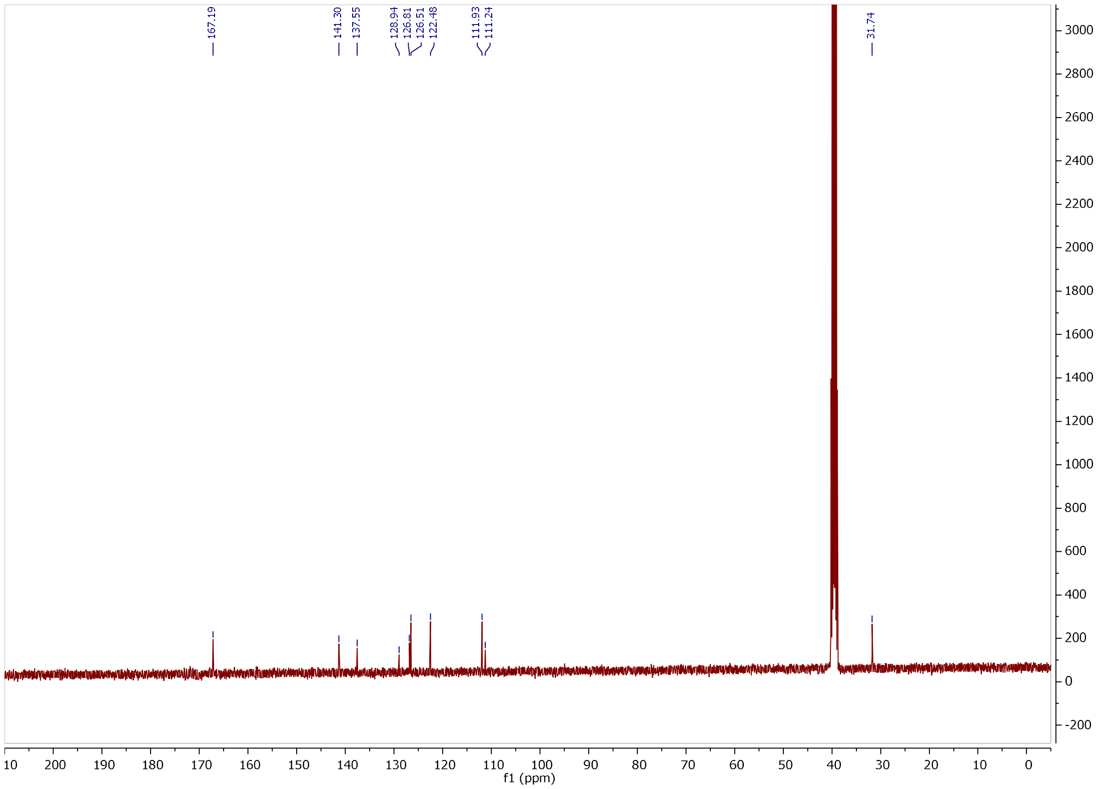

167.19
141.30
137.55
128.94
126.81
126.51
122.48
111.93
111.24
31.74
f1 (ppm)

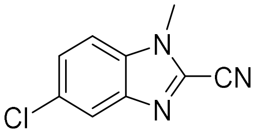

Compound 30

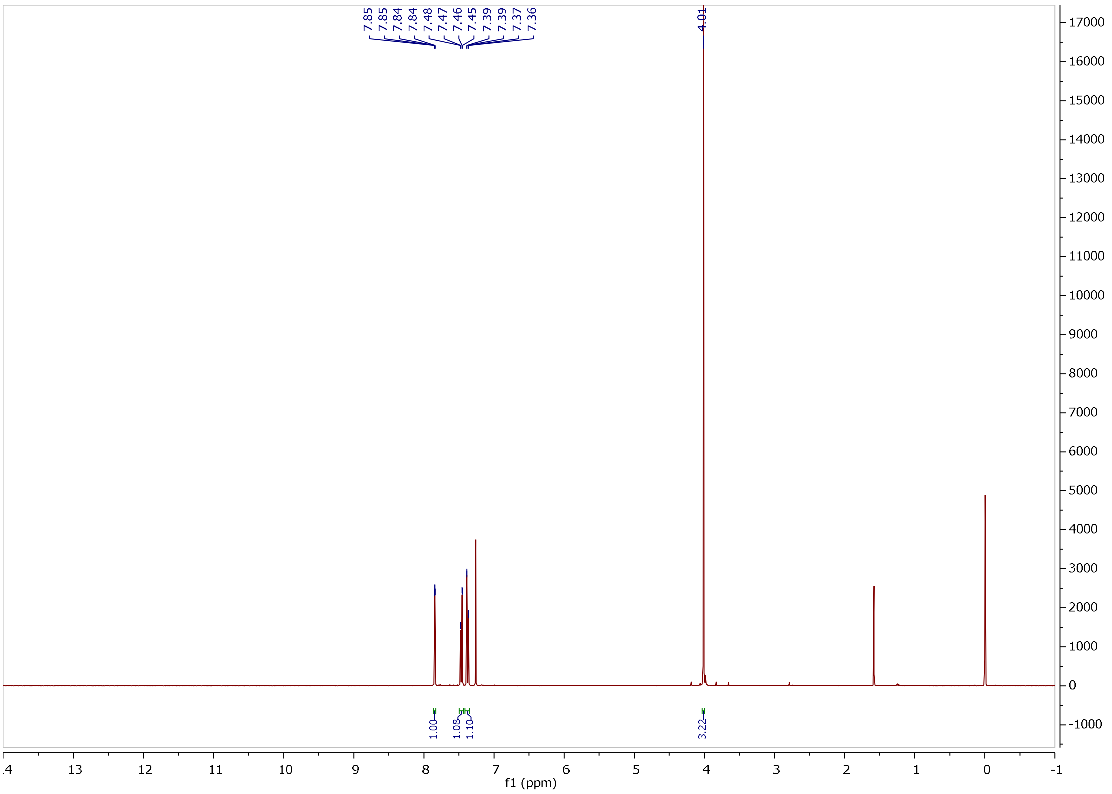

7.85
7.85
7.84
7.84
7.48
7.47
7.46
7.45
7.39
7.39
7.37
7.36
4.01
1.00
1.08
1.10
3.22
f1 (ppm)

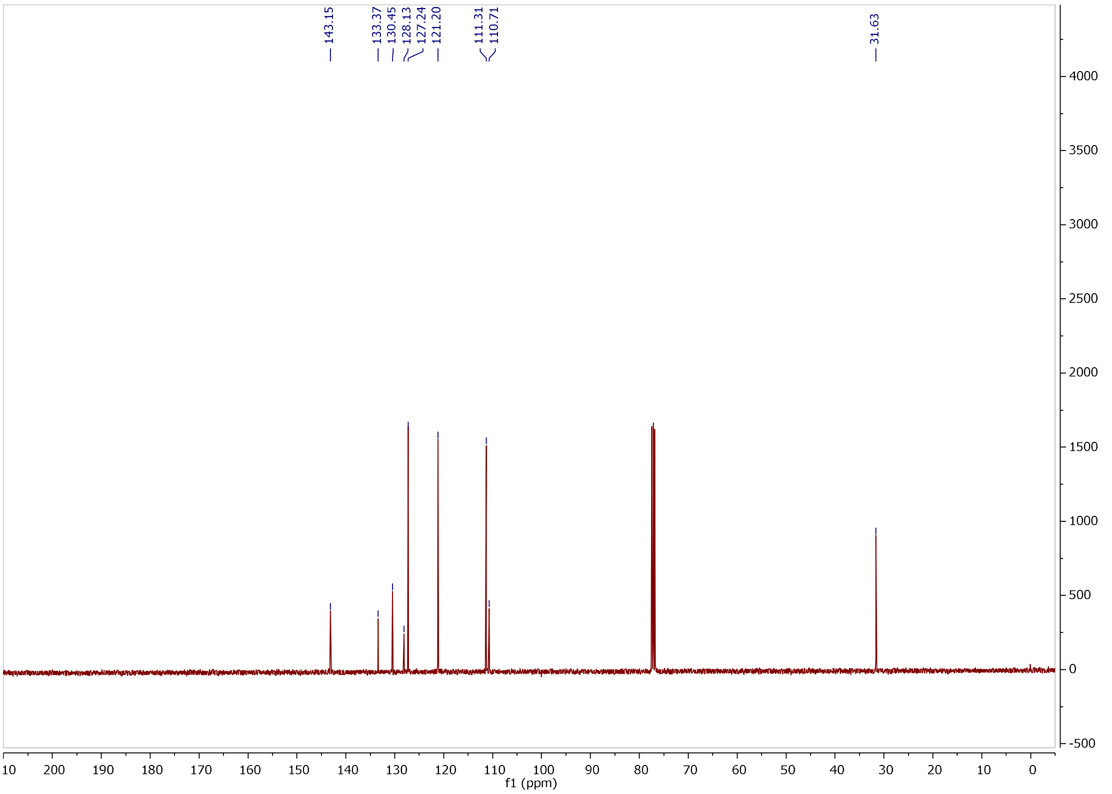

143.15
133.37
130.45
128.13
127.24
121.20
111.31
110.71
31.63
f1 (ppm)

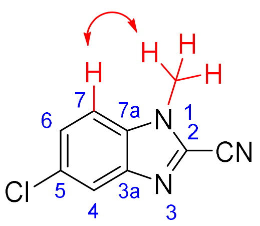

H
H
H
H
7
7a
1
6
2
N
CN
Cl
5
3a
N
4
3

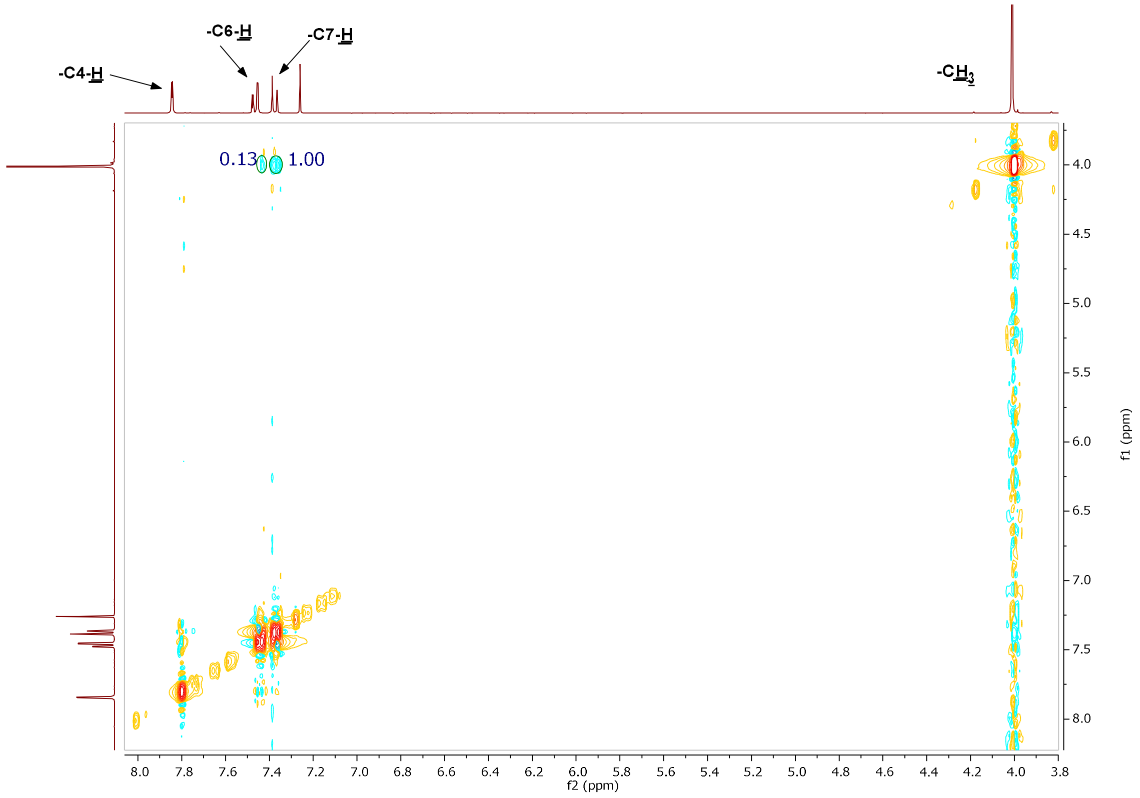

-C4-H
-C6-H
-C7-H
-CH3
0.13
1.00
f2 (ppm)
f1 (ppm)

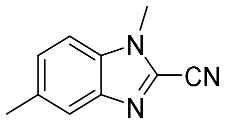
N
N
CN

Compound 31

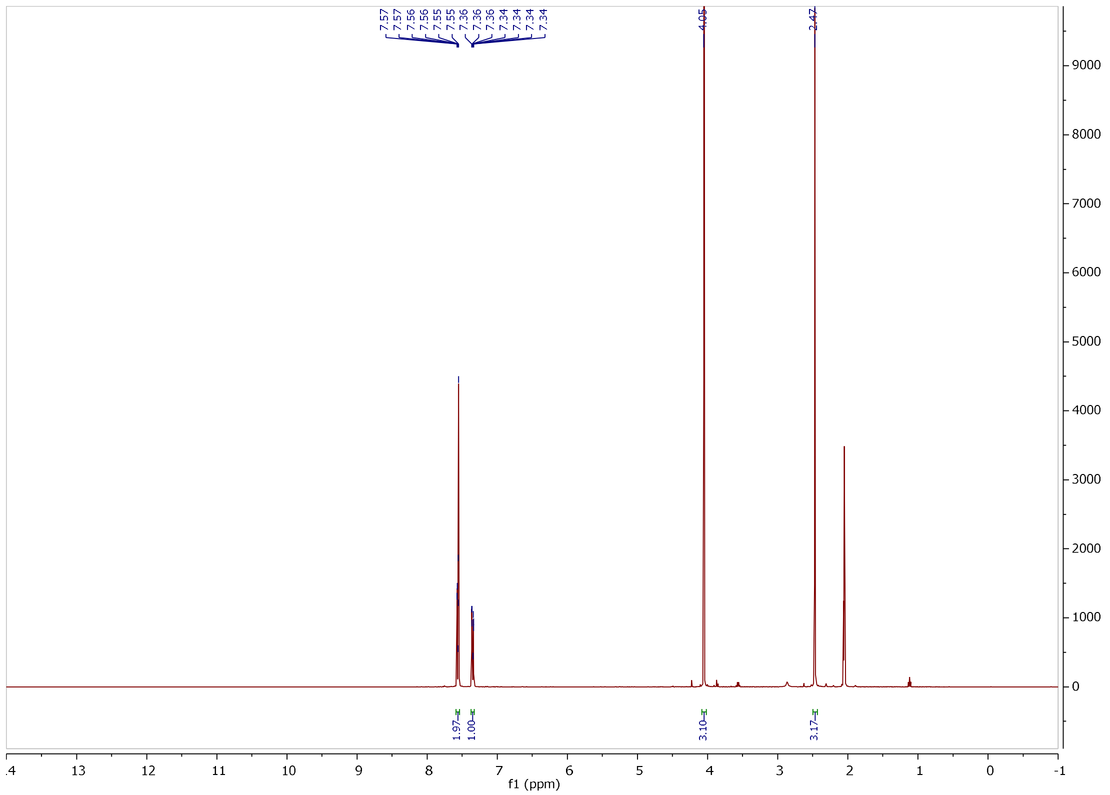
7.57
7.57
7.56
7.56
7.55
7.55
7.36
7.36
7.36
7.34
7.34
7.34
7.34
4.05
2.47
1.97
1.00
3.10
3.17
f1 (ppm)

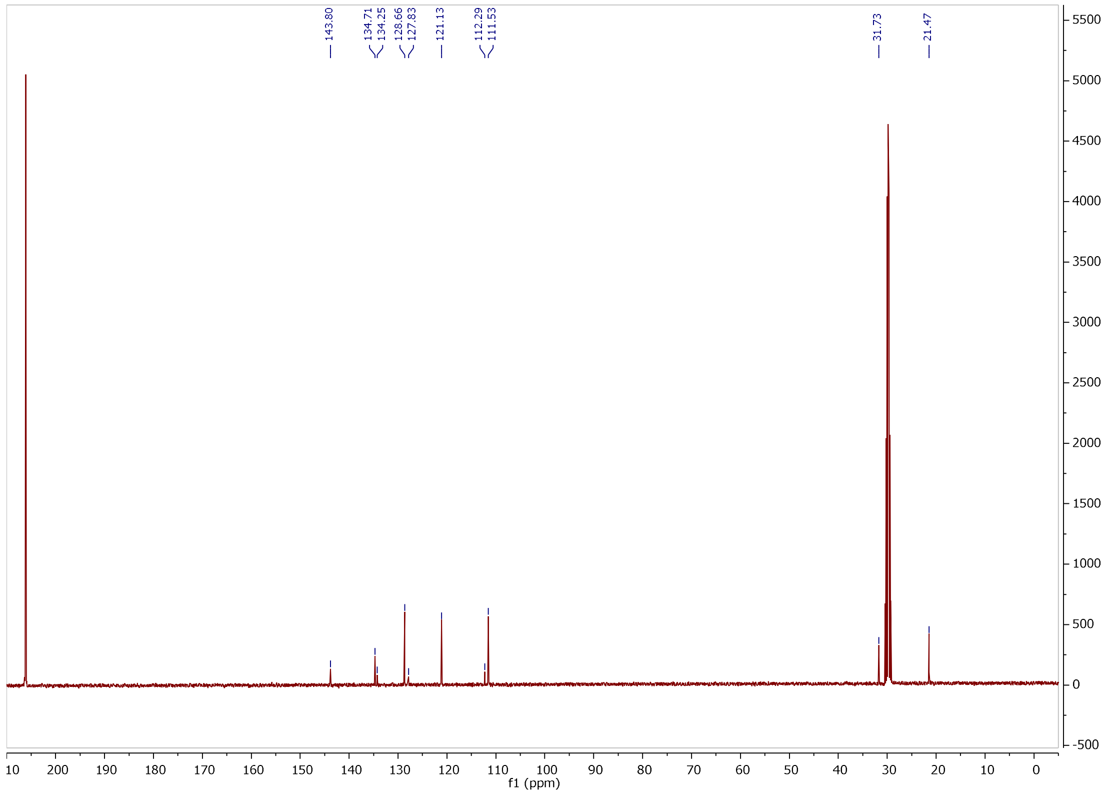
143.80
134.71
134.25
128.66
127.83
121.13
112.29
111.53
31.73
21.47
f1 (ppm)

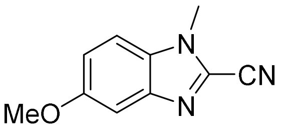
N
CN
MeO
N

Compound 32

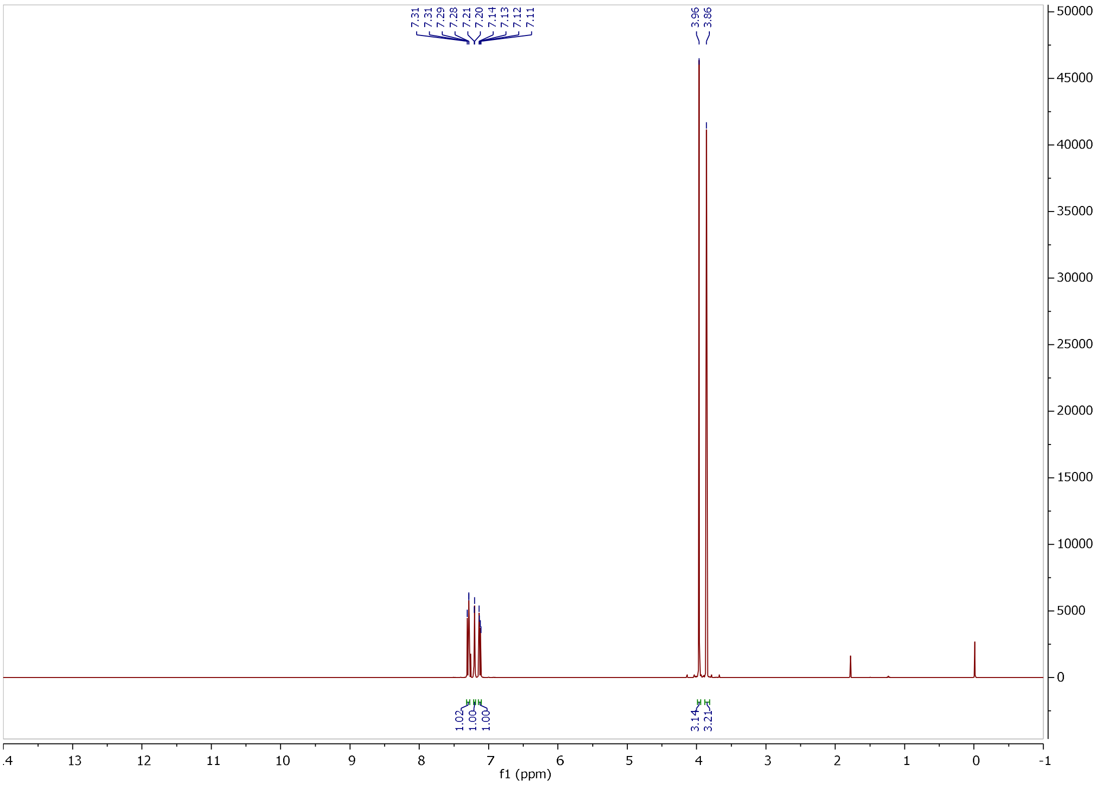
7.31
7.31
7.29
7.28
7.21
7.20
7.14
7.13
7.12
7.11
3.96
3.86
1.02
1.00
1.00
3.14
3.21
f1 (ppm)

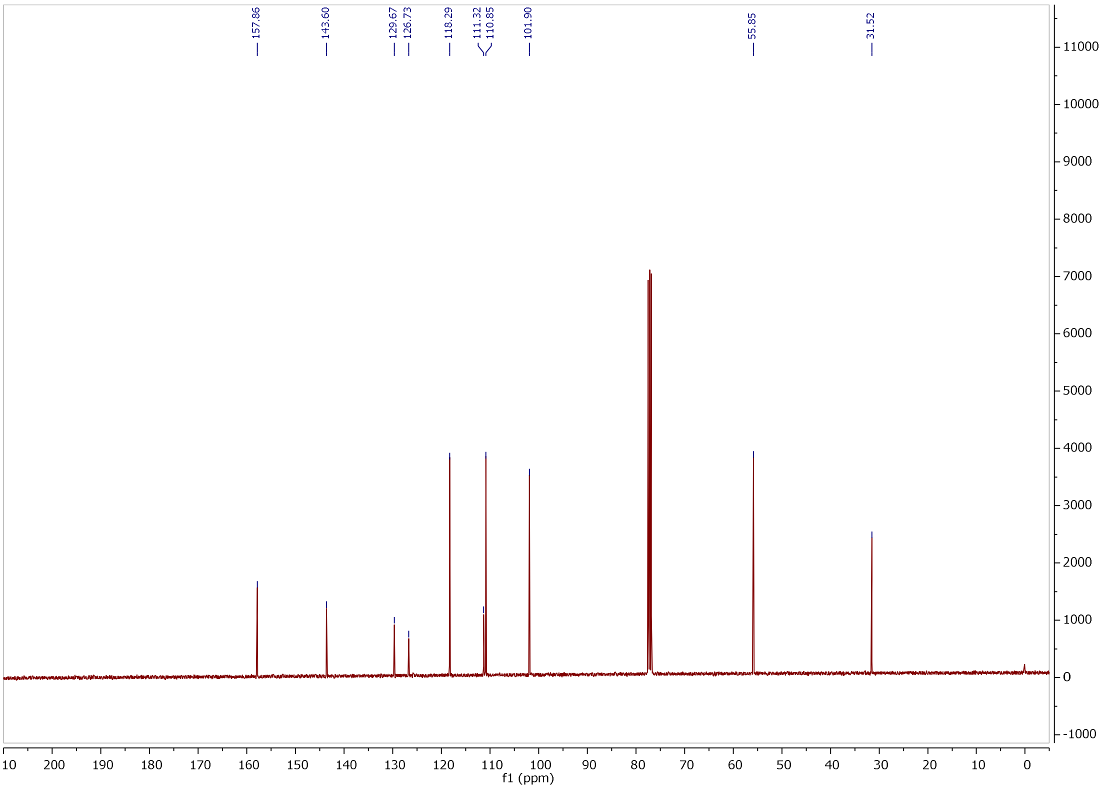
157.86
143.60
129.67
126.73
118.29
111.32
110.85
101.90
55.85
31.52
f1 (ppm)

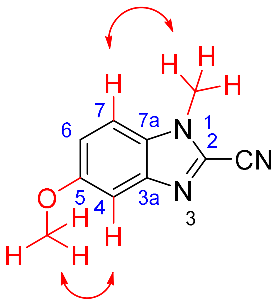

7
7a
1
2
CN
6
5
4
3a
3
N
N
O
H

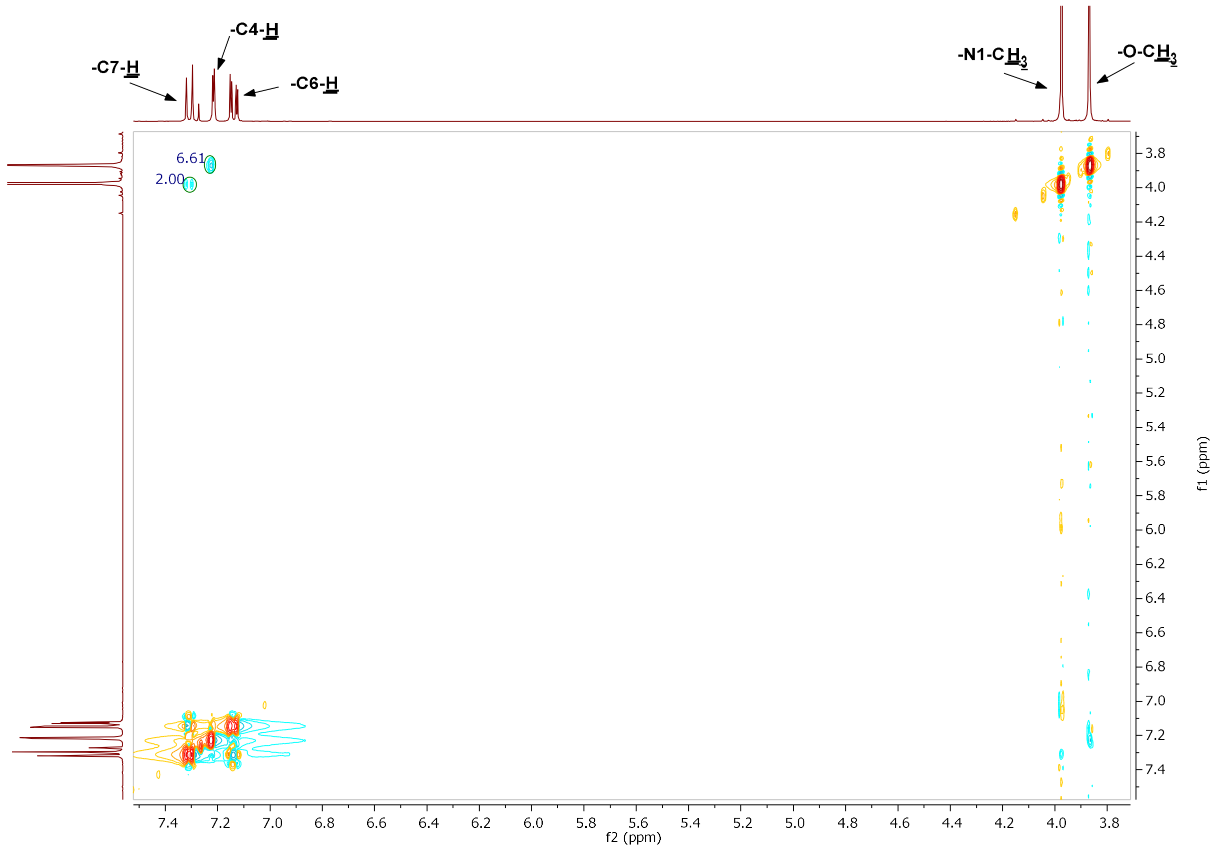

-C7-H
-C4-H
-C6-H
-N1-CH3
-O-CH3
6.61
2.00
f2 (ppm)
f1 (ppm)

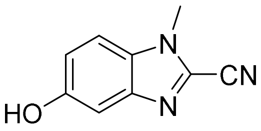

Compound 33

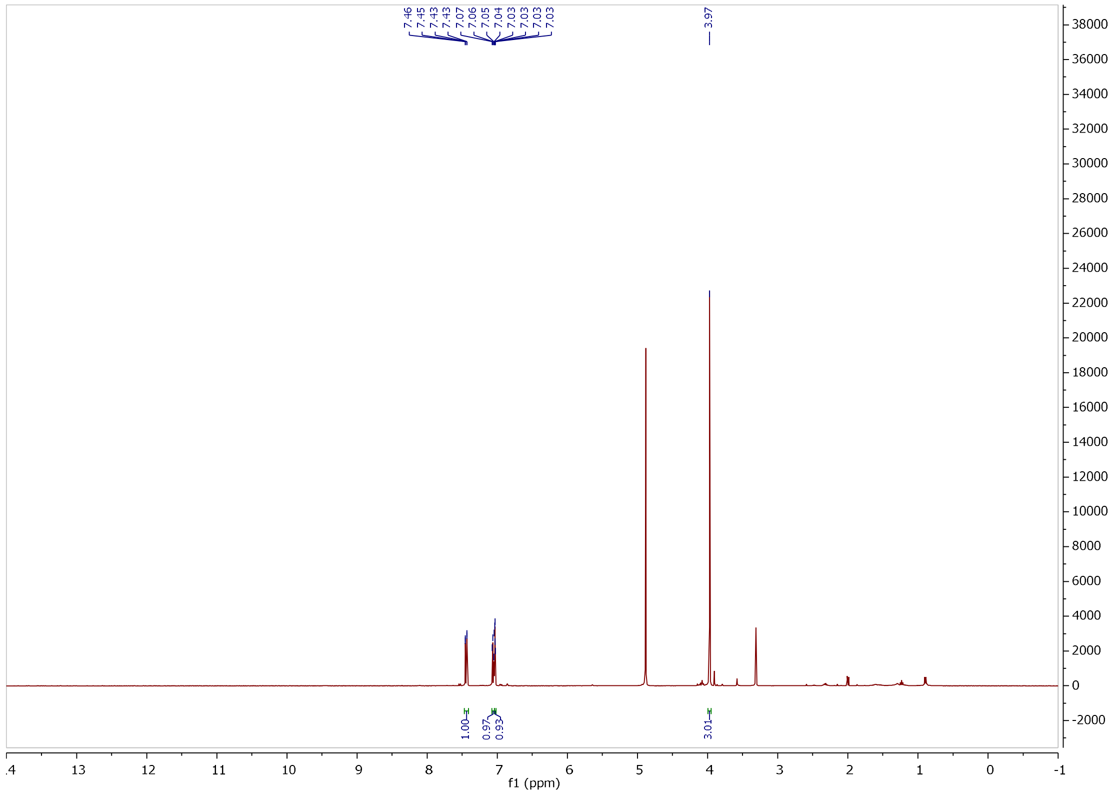
7.46
7.45
7.43
7.43
7.07
7.06
7.05
7.04
7.03
7.03
7.03
7.03
3.97
1.00
0.97
0.93
3.01
f1 (ppm)

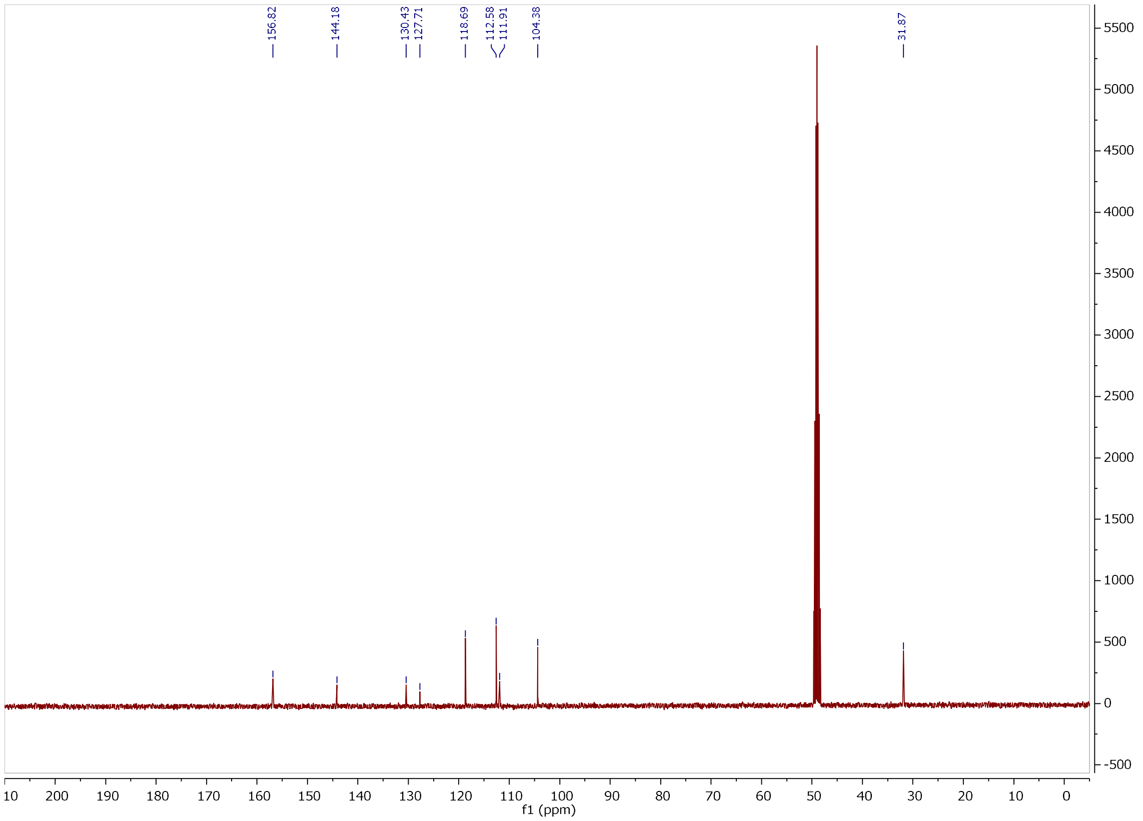
156.82
144.18
130.43
127.71
118.69
112.58
111.91
104.38
31.87
f1 (ppm)

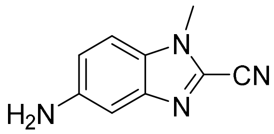

Compound 34

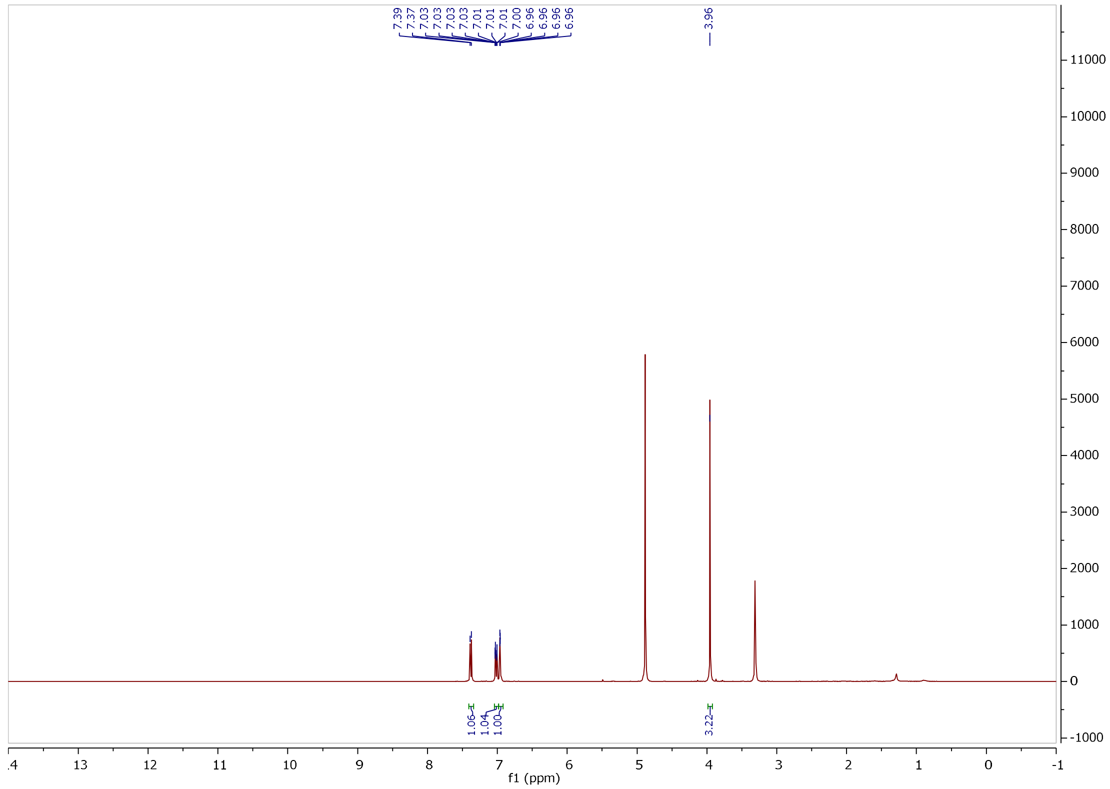

7.39
7.37
7.03
7.03
7.03
7.03
7.01
7.01
7.01
7.00
6.96
6.96
6.96
6.96
3.96
1.06
1.04
1.00
3.22
f1 (ppm)

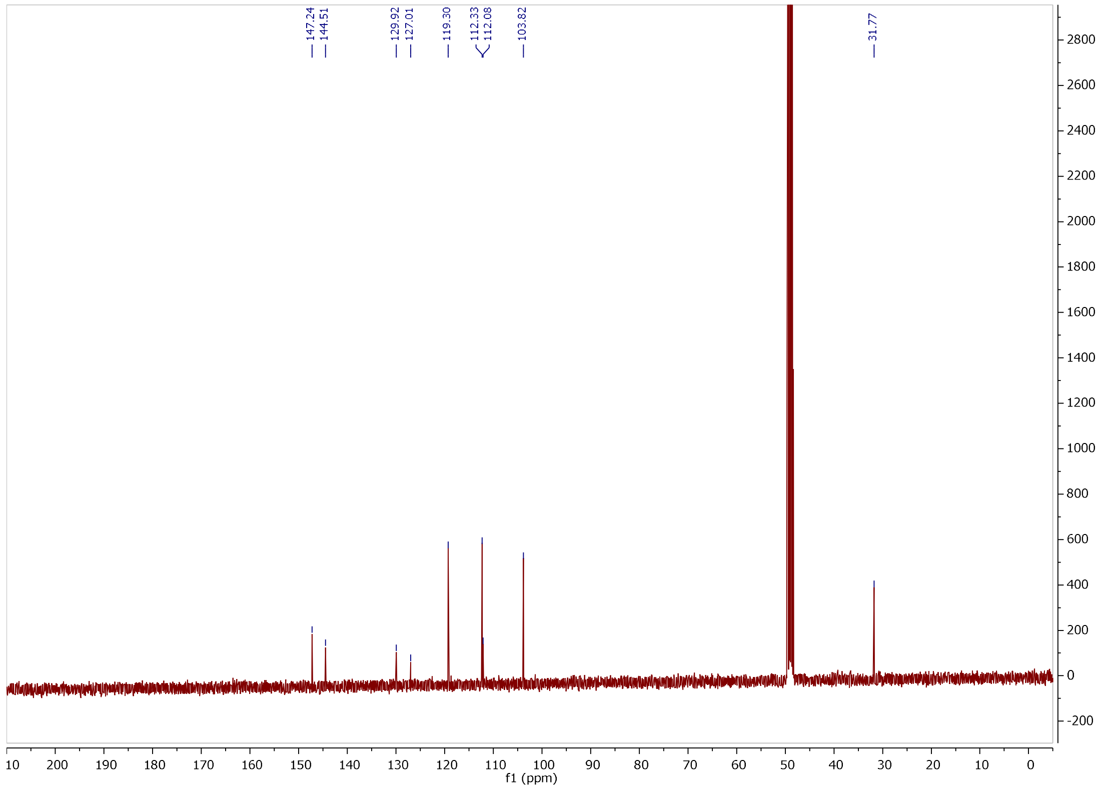

147.24
144.51
129.92
127.01
119.30
112.33
112.08
103.82
31.77
f1 (ppm)

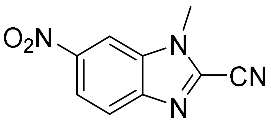
O2N
N
CN
N

Compound 35

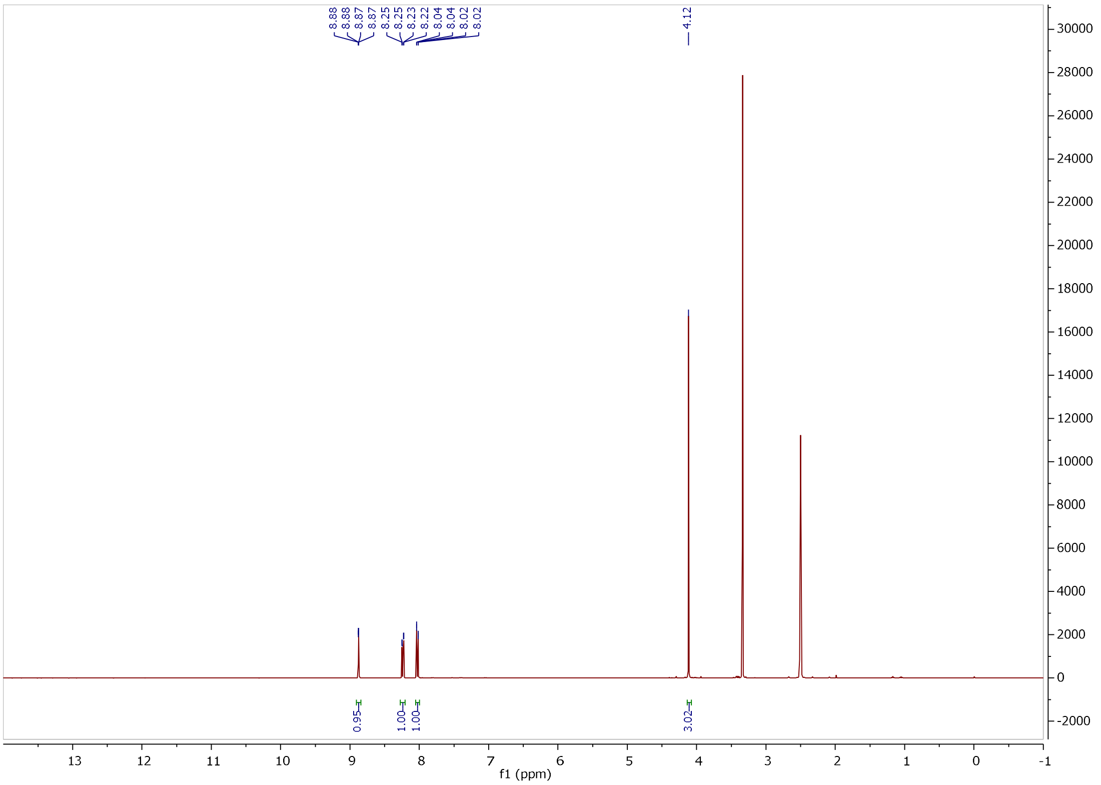
8.88
8.88
8.87
8.87
8.25
8.25
8.23
8.22
8.04
8.04
8.02
8.02
4.12
0.95
1.00
1.00
3.02
f1 (ppm)

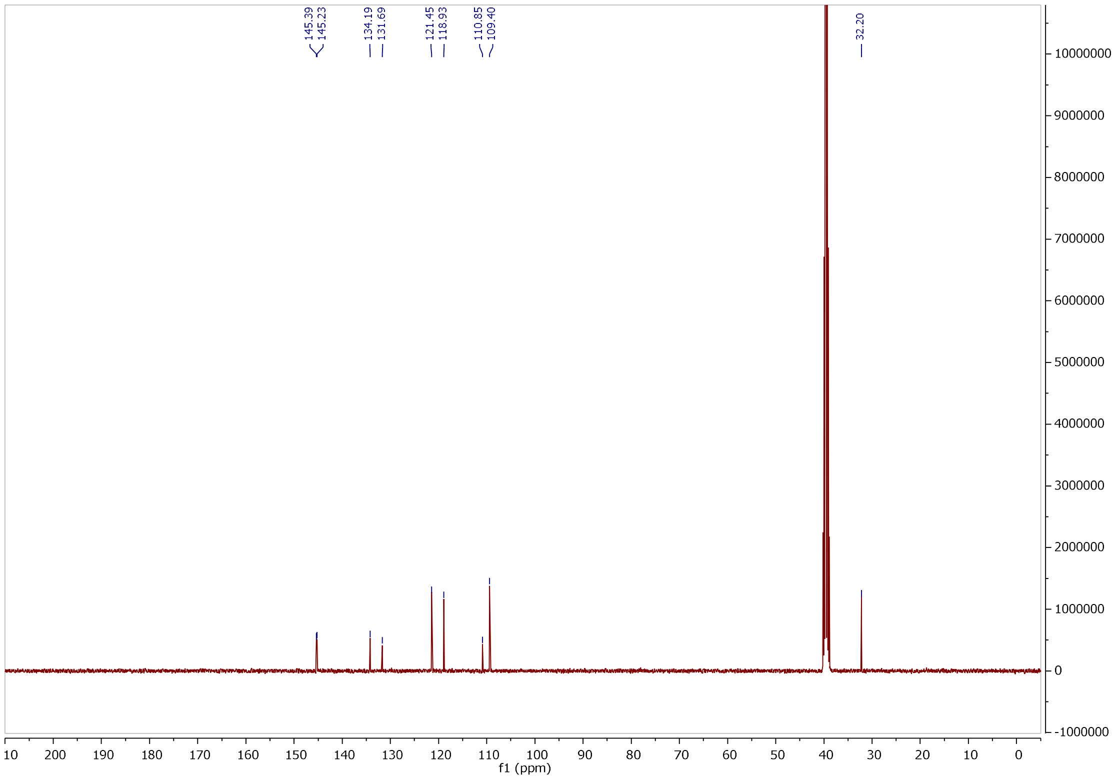
145.39
145.23
134.19
131.69
121.45
118.93
110.85
109.40
32.20
f1 (ppm)

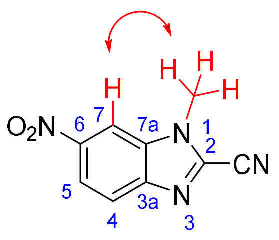

O2N
H
7
7a
N
1
2
CN
6
5
4
3a
N
3

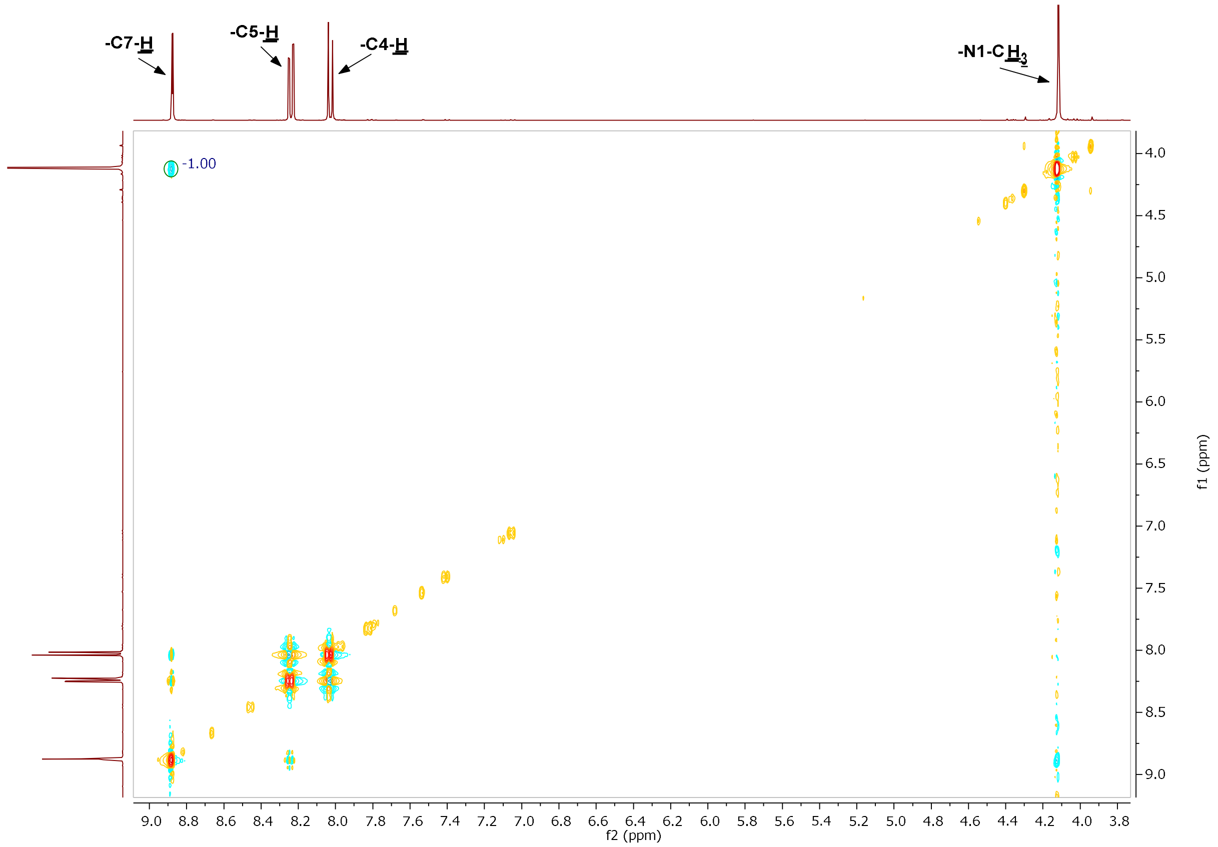

-C7-H
-C5-H
-C4-H
-N1-CH3
-1.00
f2 (ppm)
f1 (ppm)

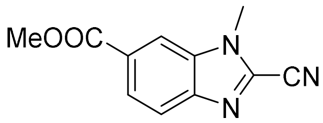
MeOOC
N
N
CN

Compound 36

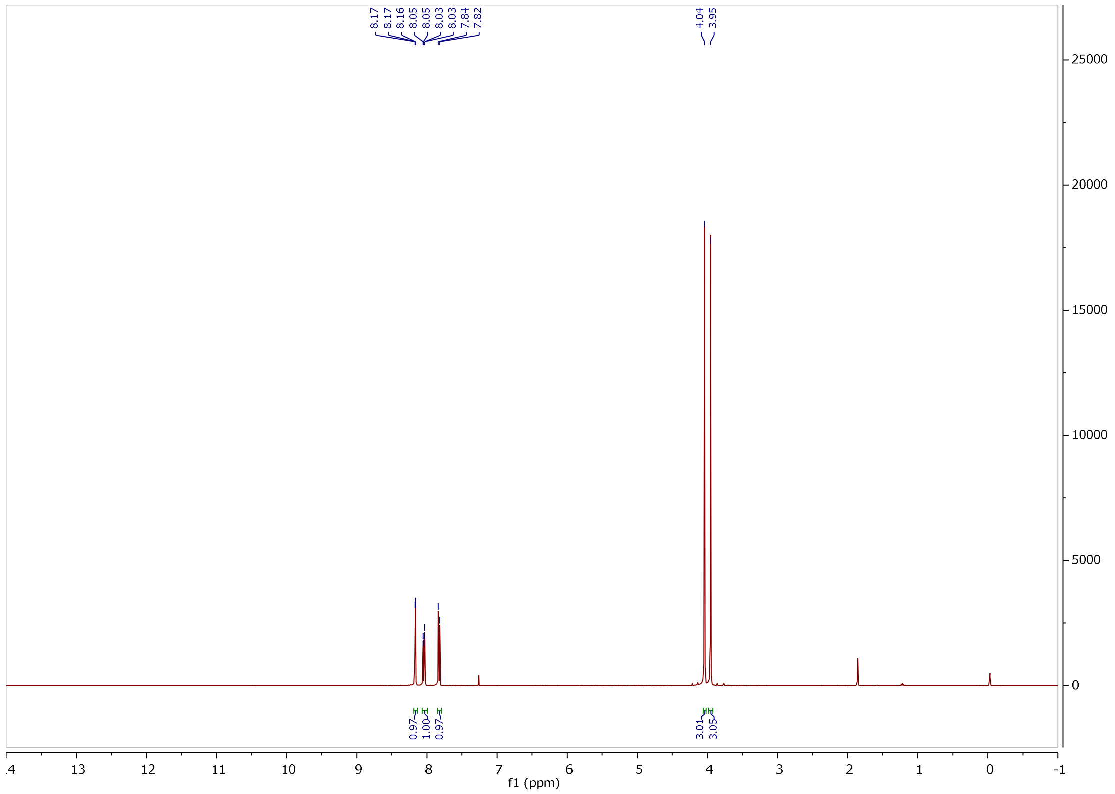
8.17
8.17
8.16
8.05
8.05
8.03
8.03
7.84
7.82
4.04
3.95
0.97
1.00
0.97
3.01
3.05
f1 (ppm)

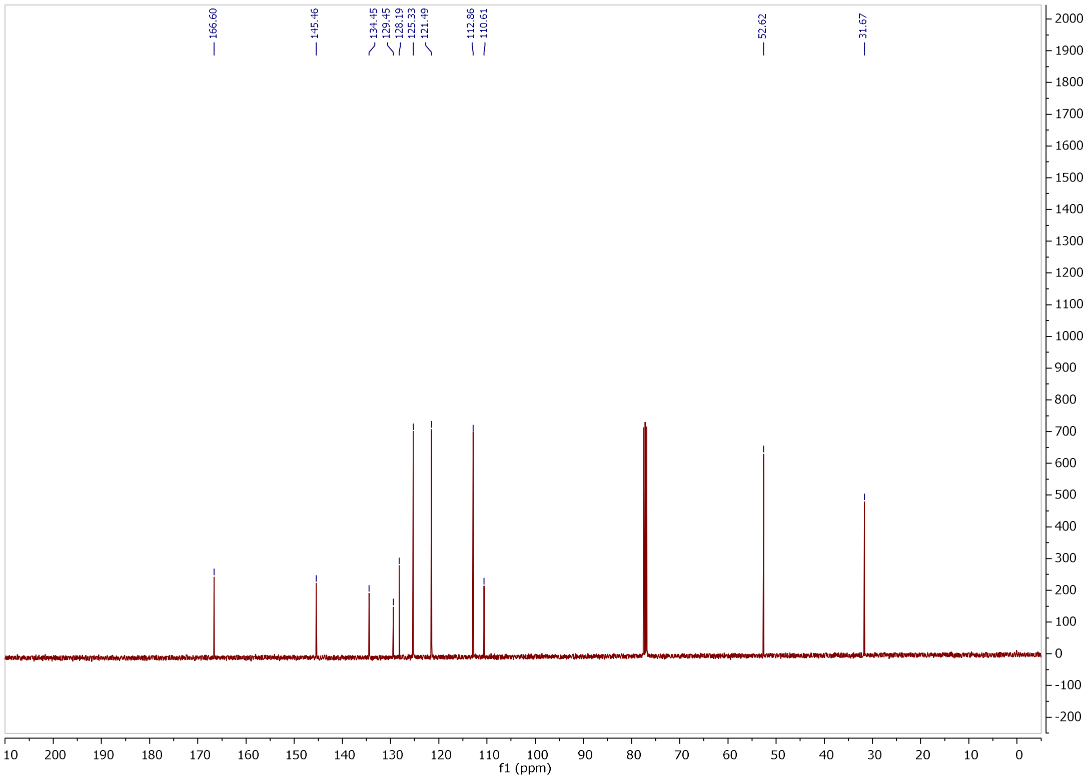
166.60
145.46
134.45
129.45
128.19
125.33
121.49
112.86
110.61
52.62
31.67
f1 (ppm)

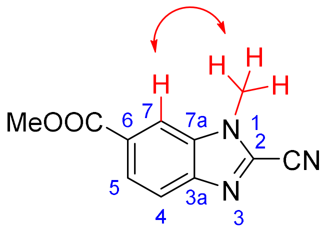
MeOOC
6
7
H
7a
N
1
2
CN
5
4
3a
N
3
H
H
H

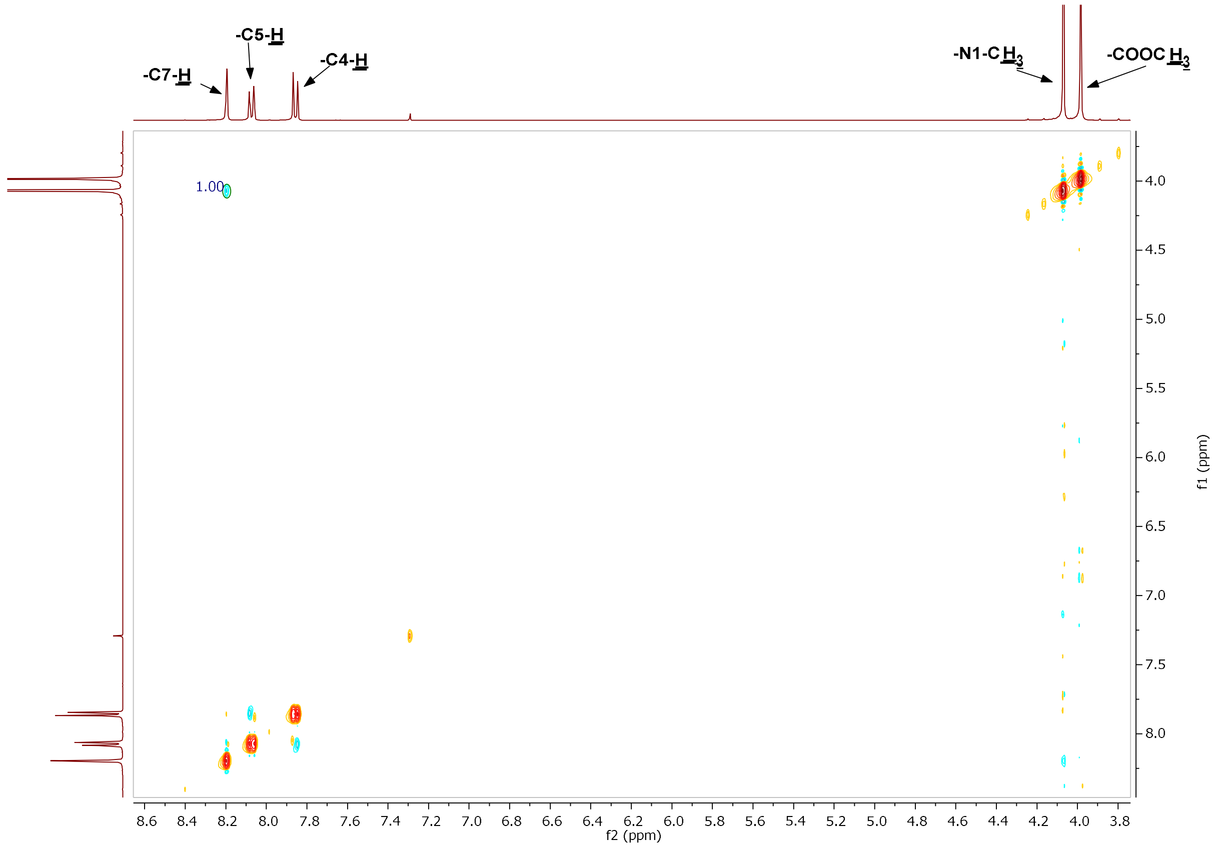
-C7-H
-C5-H
-C4-H
-N1-CH3
-COOCH3
1.00
f2 (ppm)
f1 (ppm)

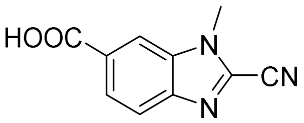

Compound 37

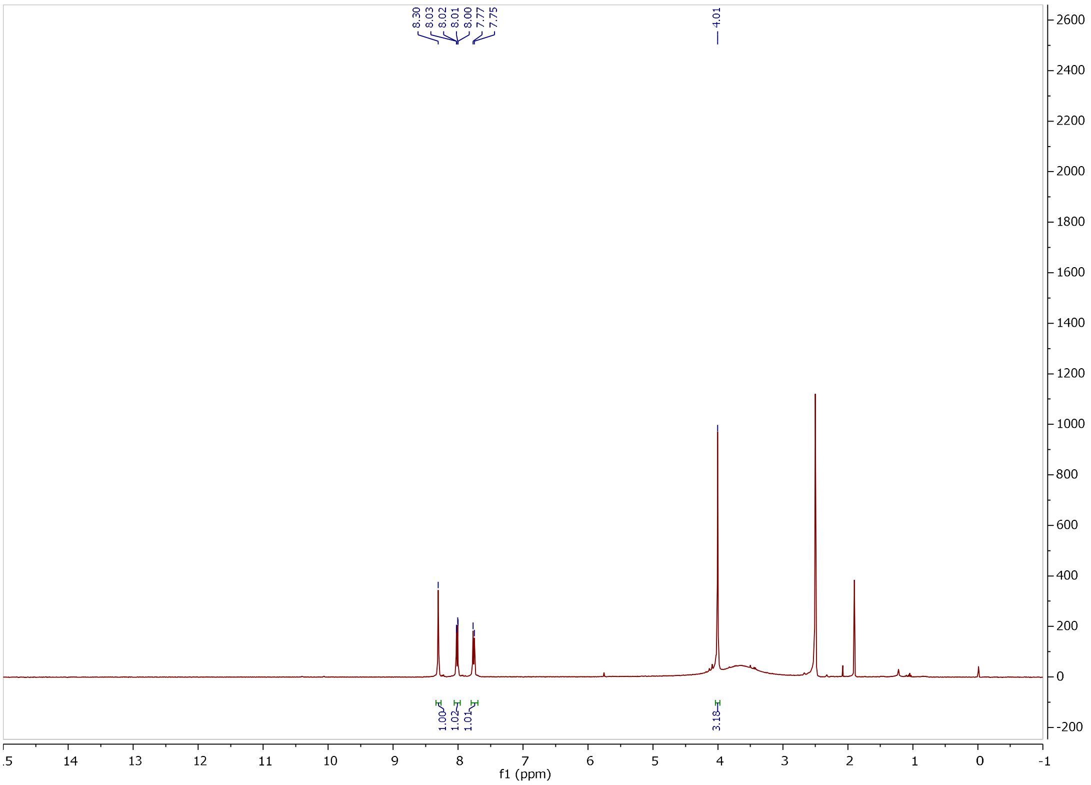

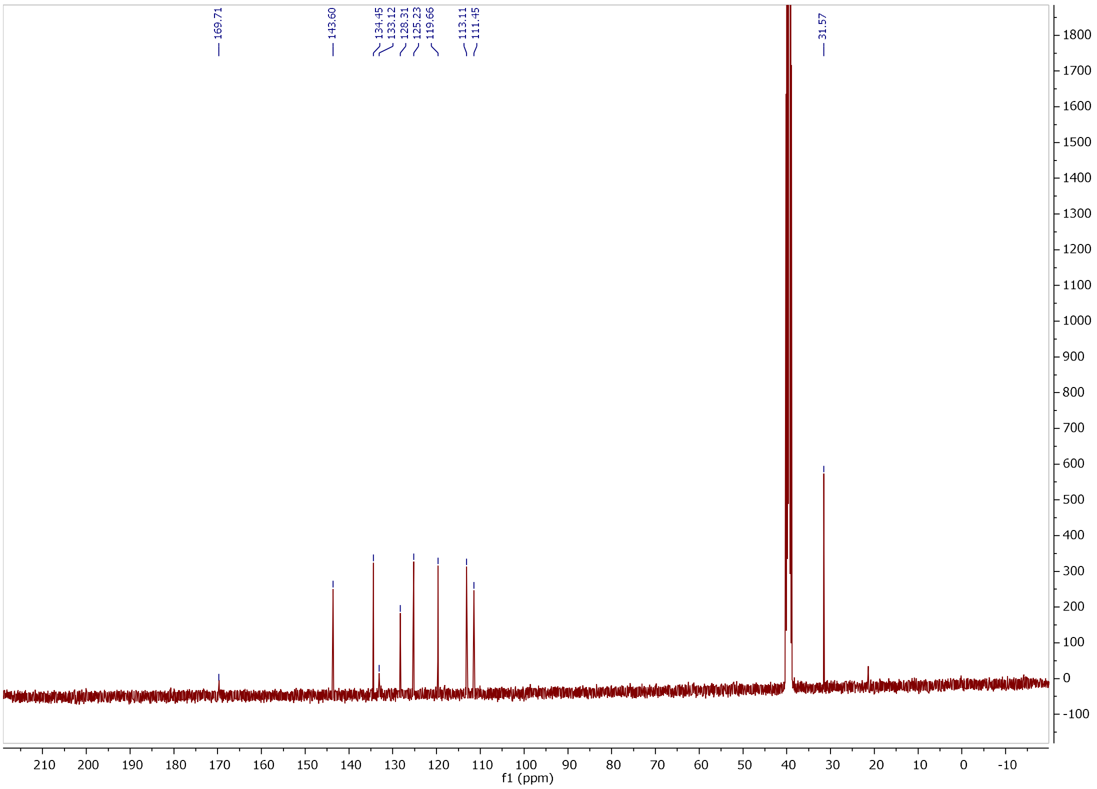

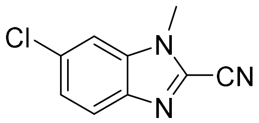

Compound 38

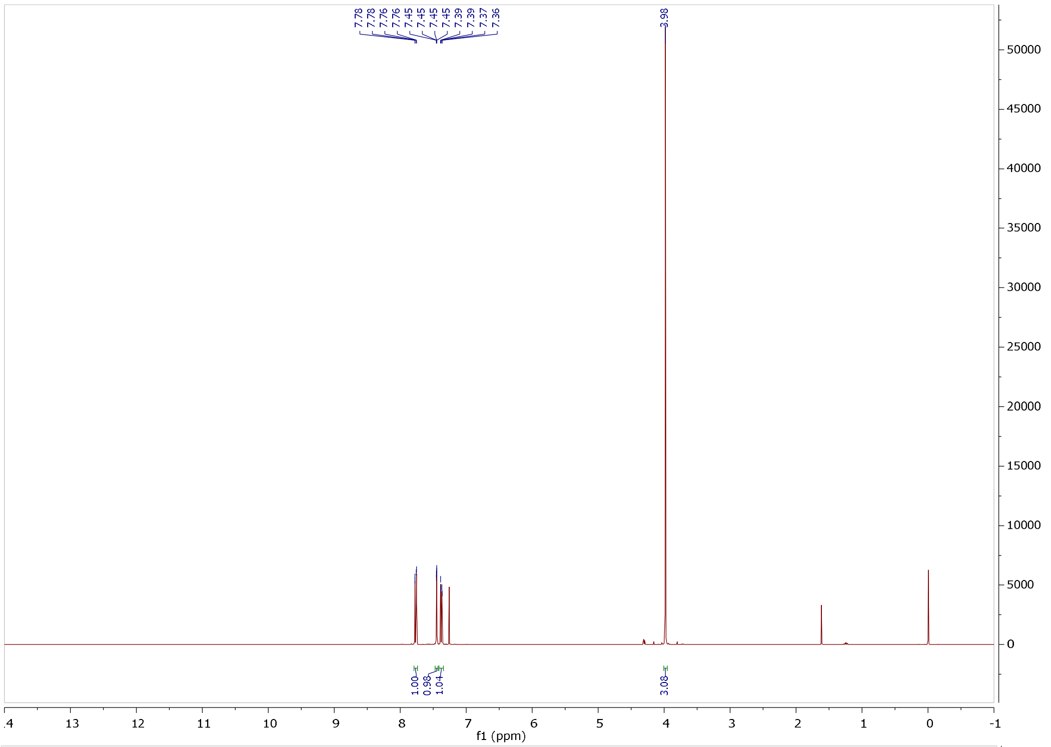

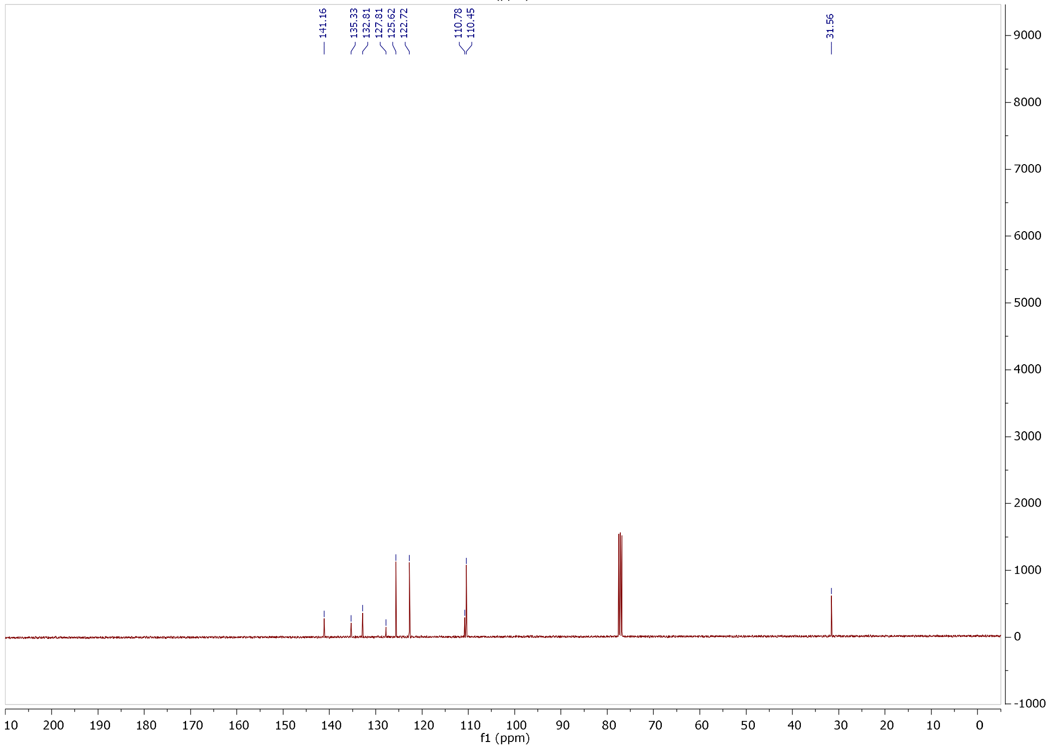

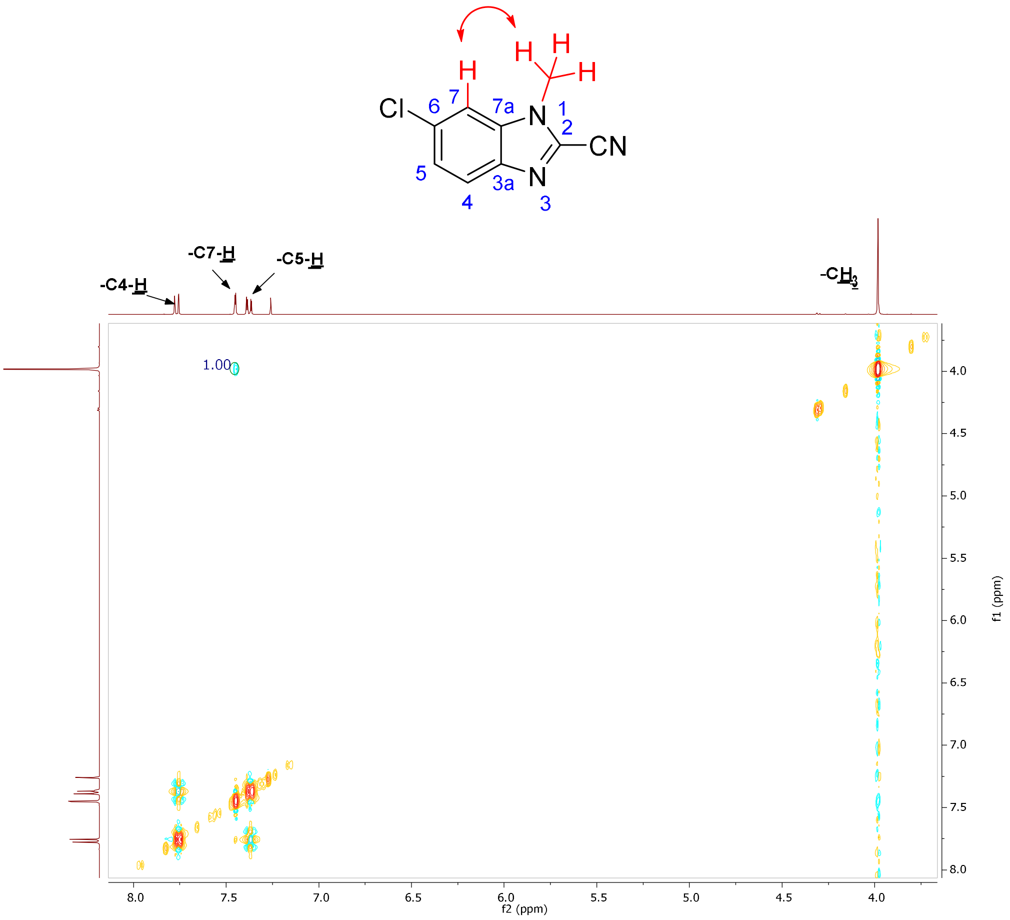
Cl
CN
N
-C4-H
-C7-H
-C5-H
-CH3
1.00
f2 (ppm)
f1 (ppm)

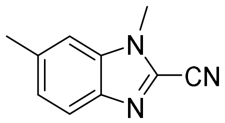

Compound 39

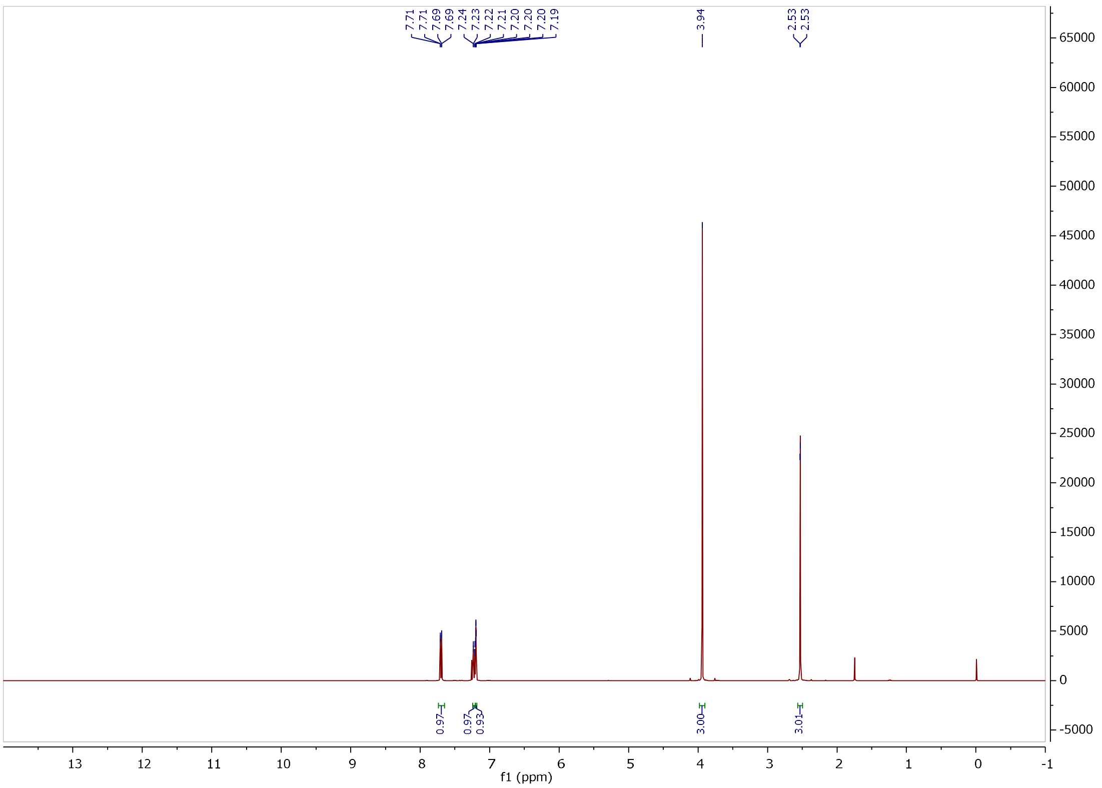

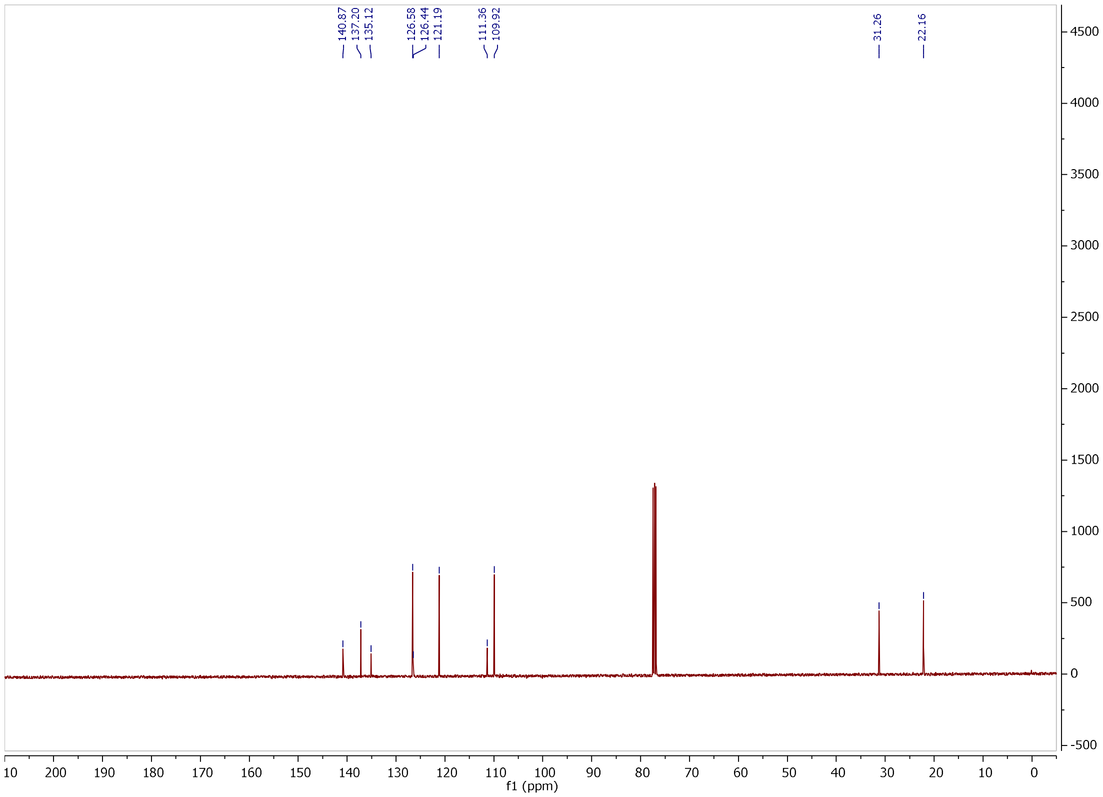

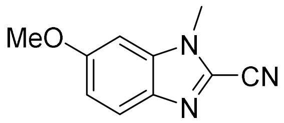
MeO
N
N
CN

Compound 40

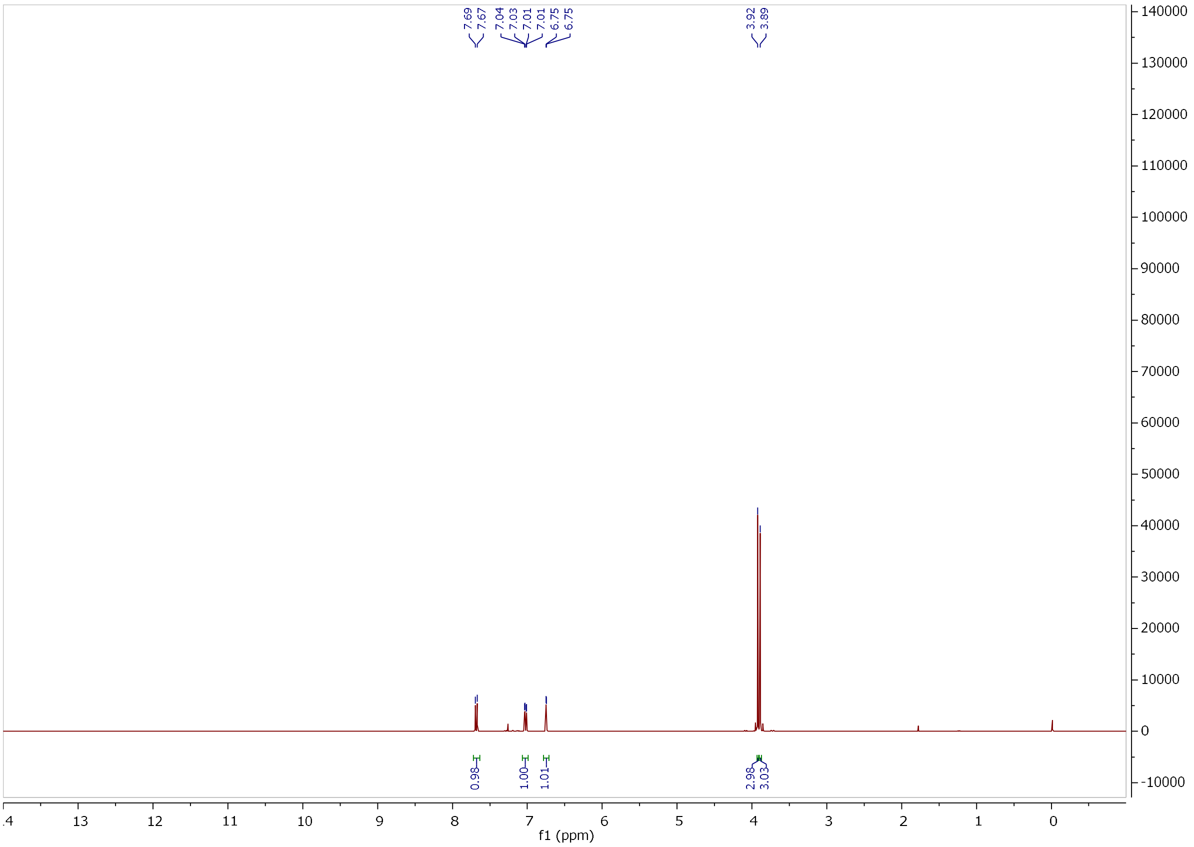
7.69
7.67
7.04
7.03
7.01
7.01
6.75
6.75
3.92
3.89
0.98
1.00
1.01
2.98
3.03
f1 (ppm)

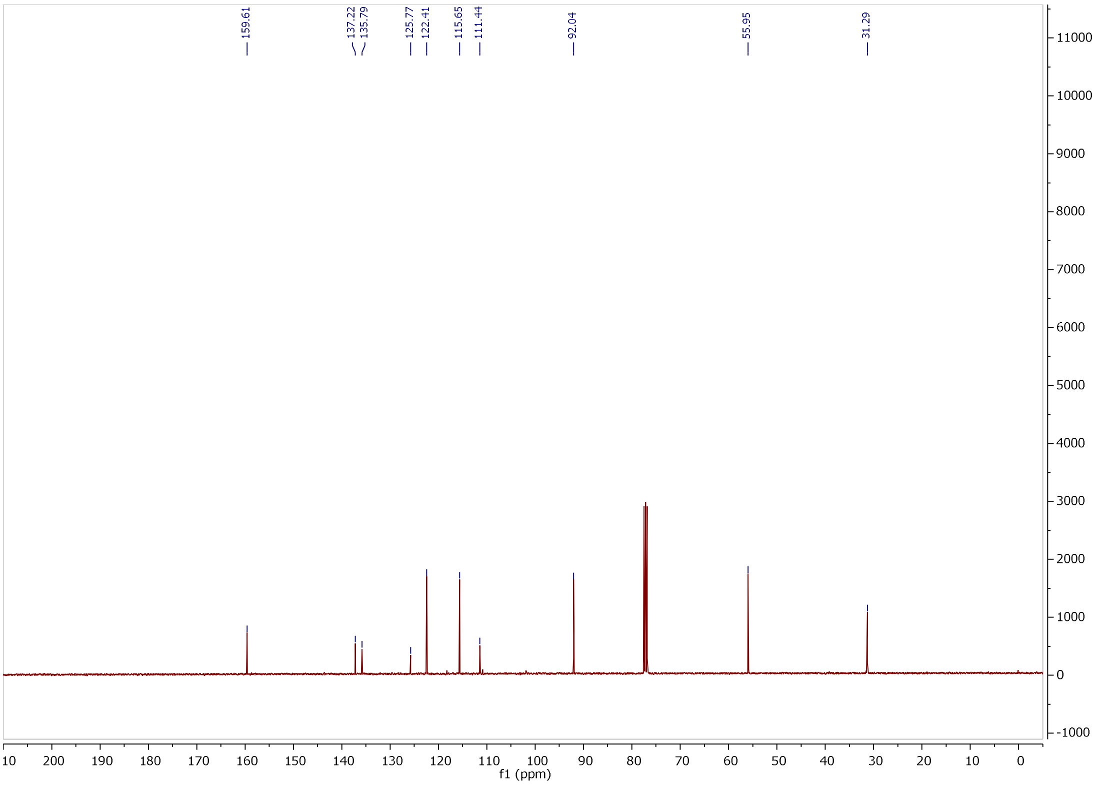
159.61
137.22
135.79
125.77
122.41
115.65
111.44
92.04
55.95
31.29
f1 (ppm)

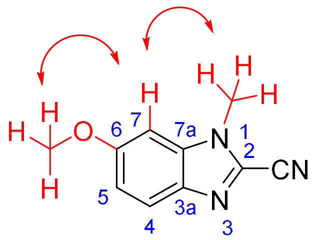

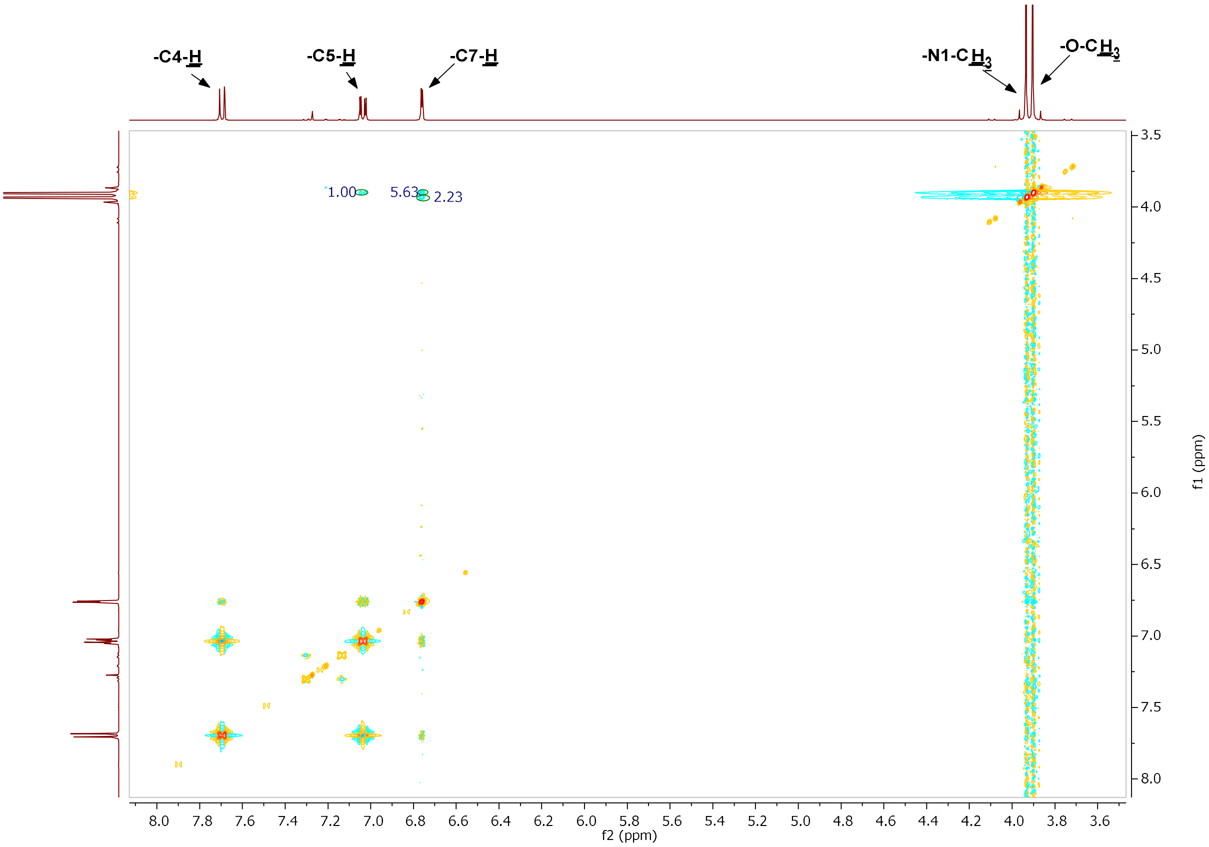
-C4-H
-C5-H
-C7-H
-N1-CH3
-O-CH3
1.00
5.63
2.23
f2 (ppm)
f1 (ppm)

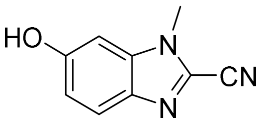
HO
N
CN
N

Compound 41

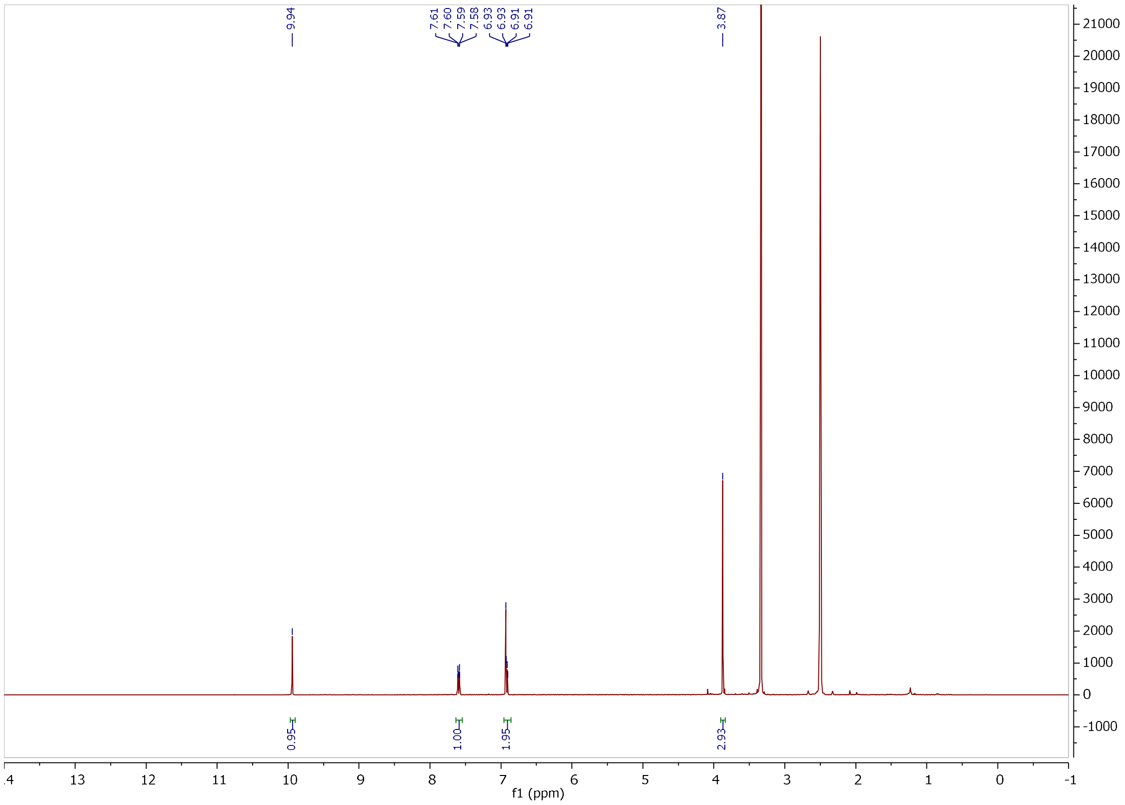
9.94
7.61
7.60
7.59
7.58
6.93
6.93
6.91
6.91
3.87
0.95
1.00
1.95
2.93
f1 (ppm)

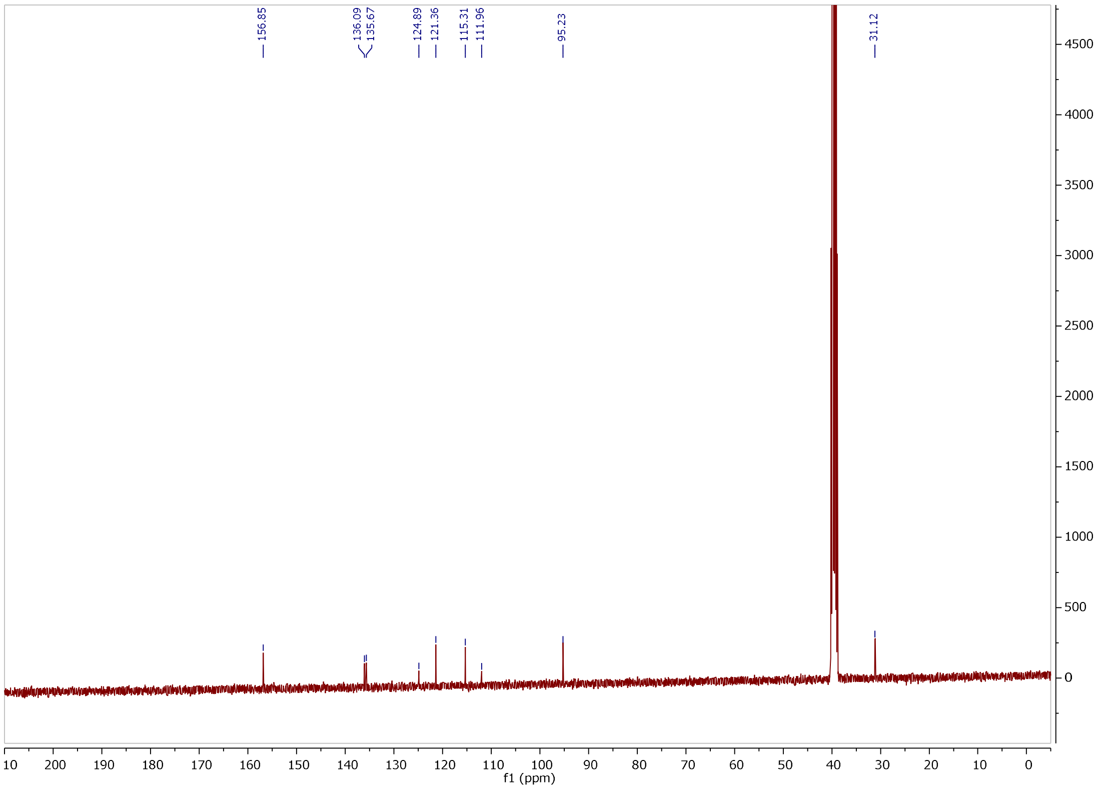
156.85
136.09
135.67
124.89
121.36
115.31
111.96
95.23
31.12
f1 (ppm)

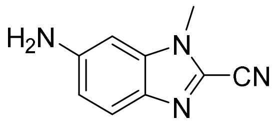

Compound 42

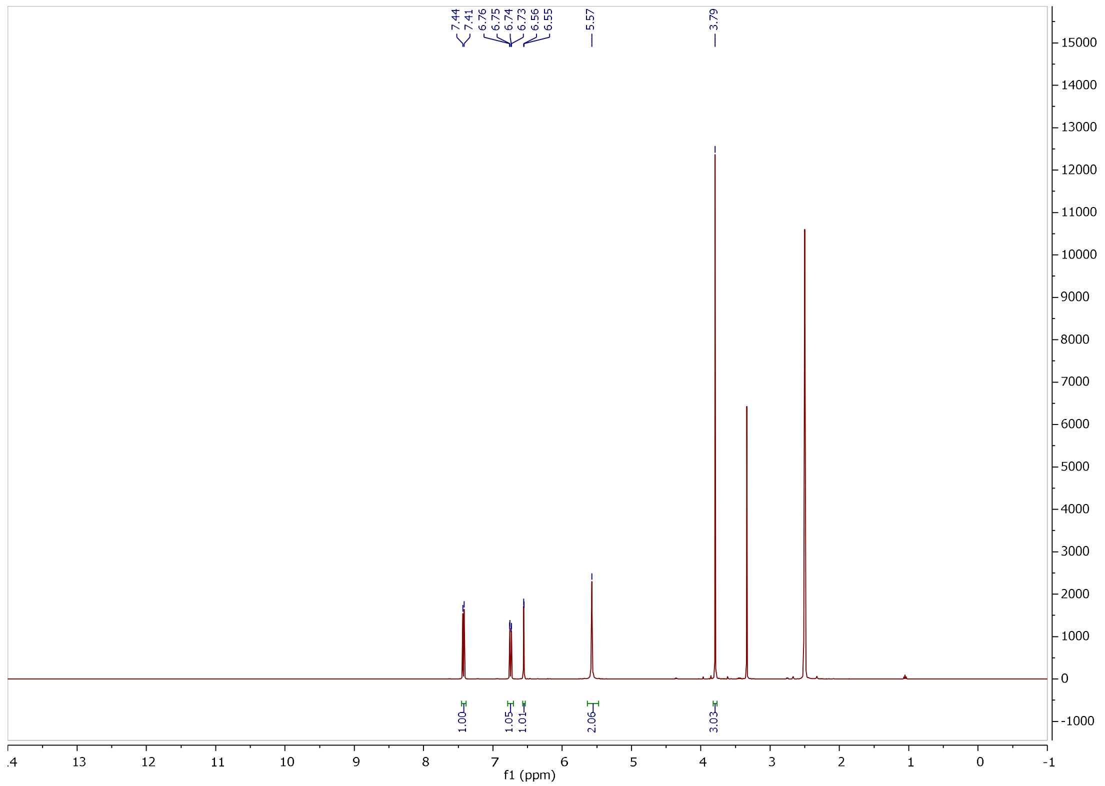

7.44
7.41
6.76
6.75
6.74
6.73
6.56
6.55
5.57
3.79
1.00
1.05
1.01
2.06
3.03
f1 (ppm)

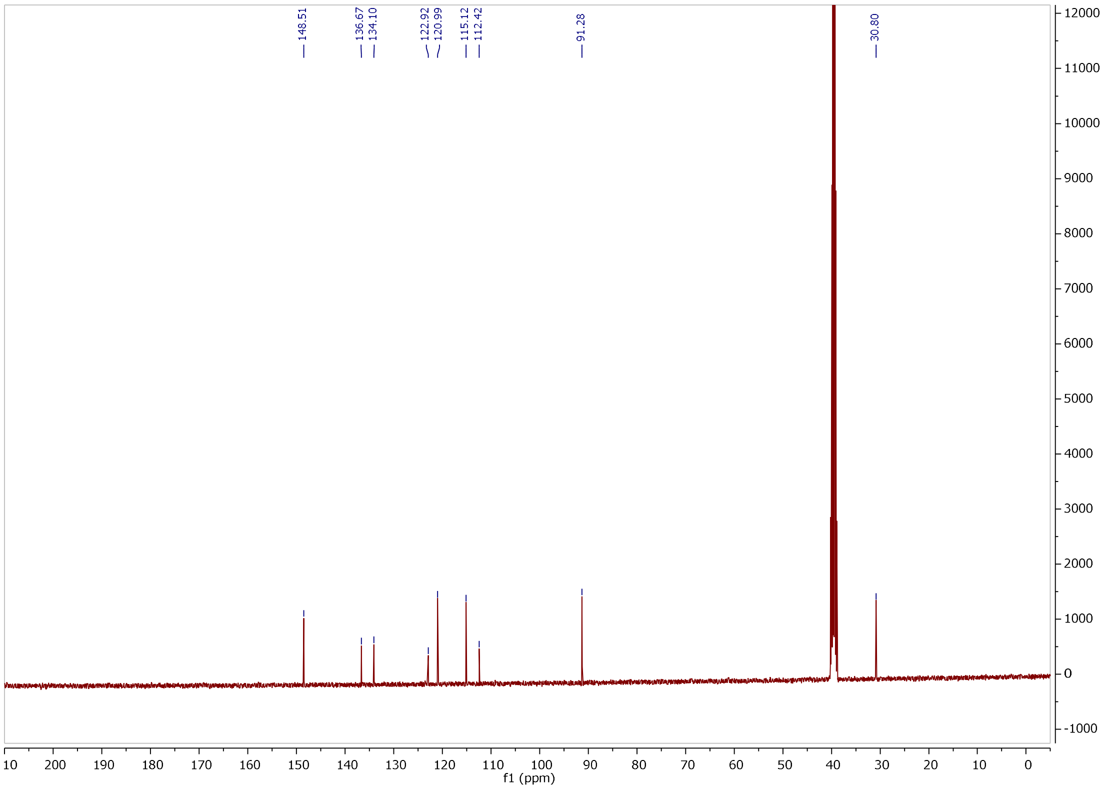

148.51
136.67
134.10
122.92
120.99
115.12
112.42
91.28
30.80
f1 (ppm)

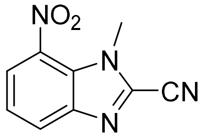

Compound 43

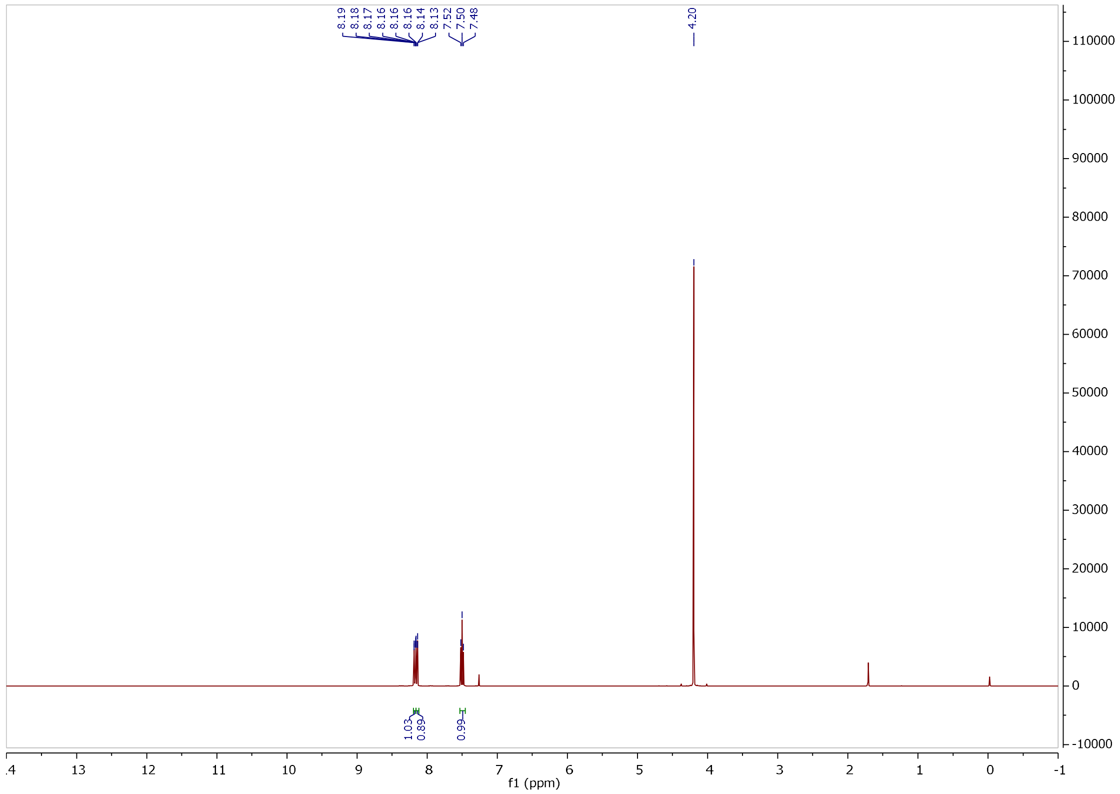

8.19
8.18
8.17
8.16
8.16
8.16
8.14
8.13
7.52
7.50
7.48
4.20
1.03
0.89
0.99
f1 (ppm)

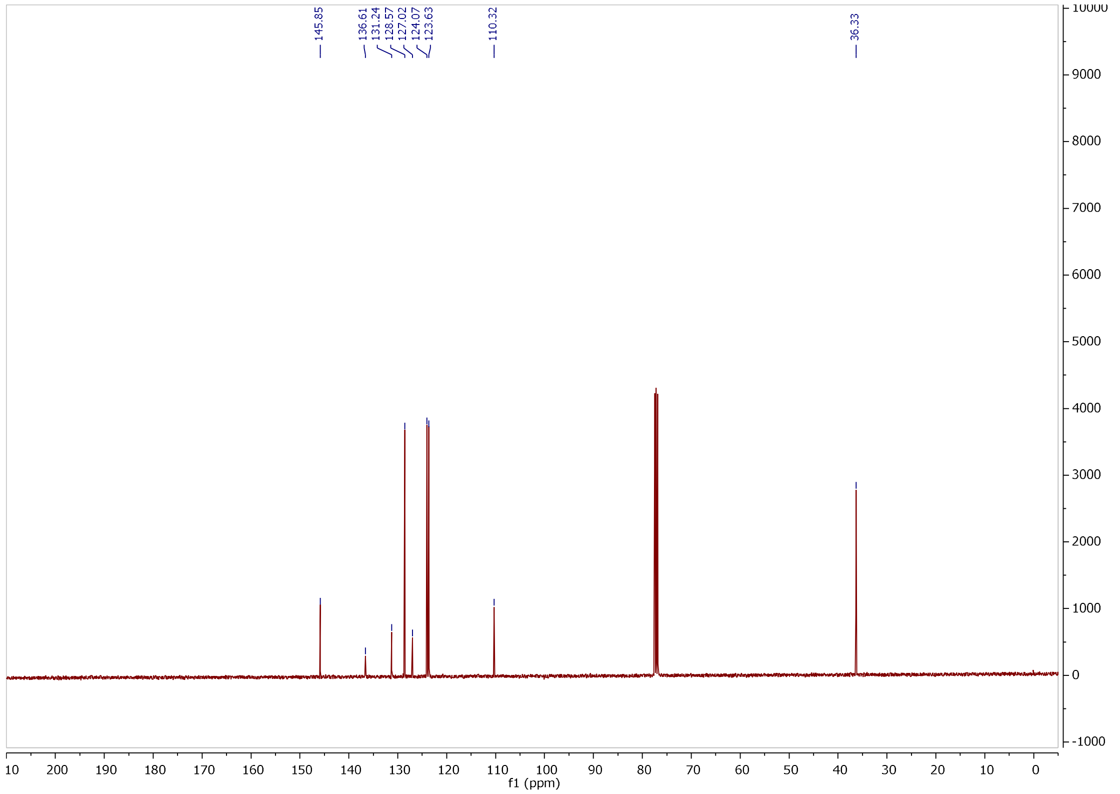

145.85
136.61
131.24
128.57
127.02
124.07
123.63
110.32
36.33
f1 (ppm)

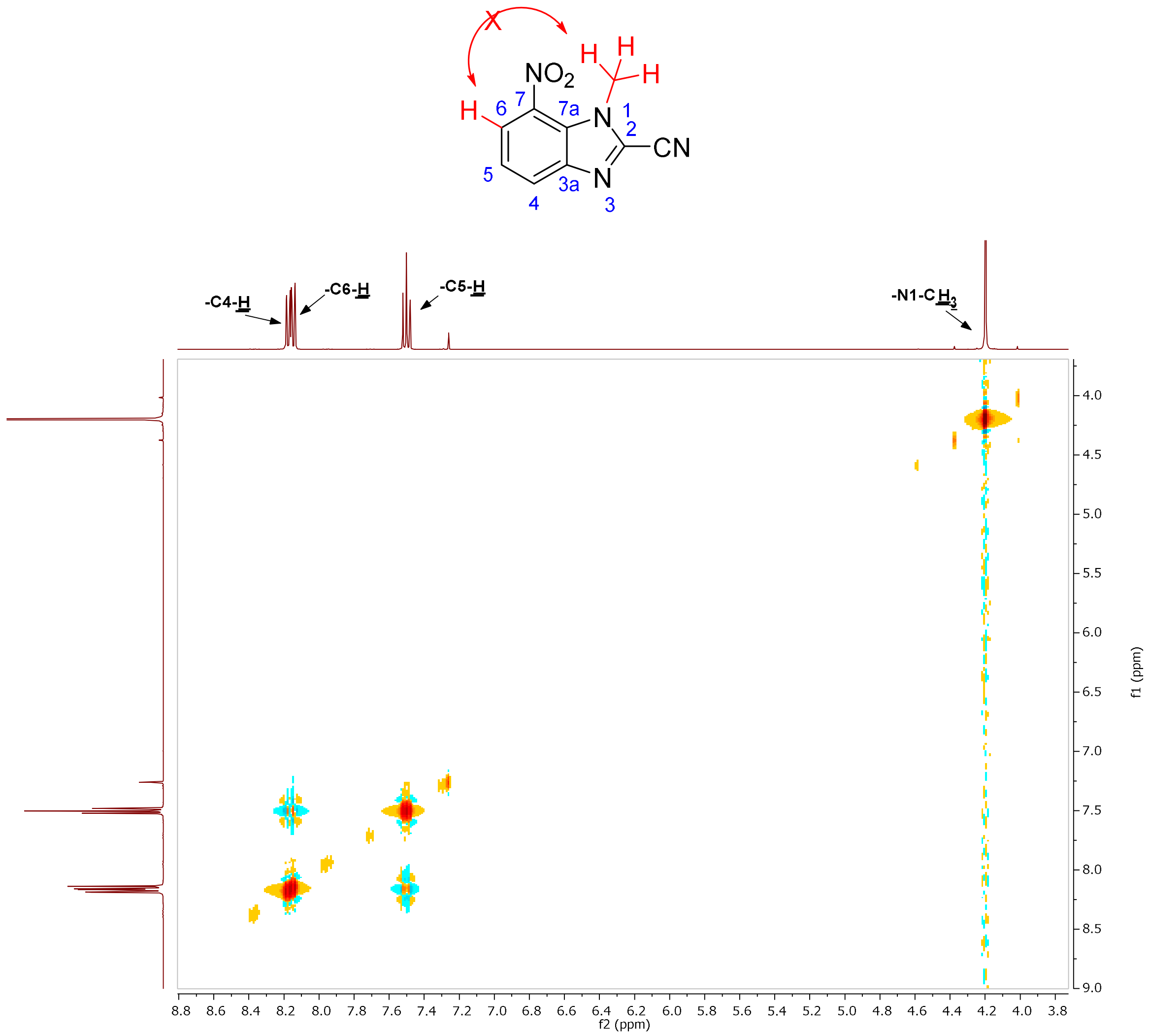

NO2
CN
-C4-H
-C6-H
-C5-H
-N1-CH3
f2 (ppm)
f1 (ppm)

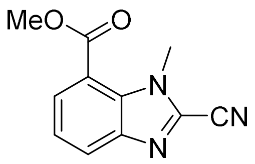
MeO
O
N
N
CN

Compound 44

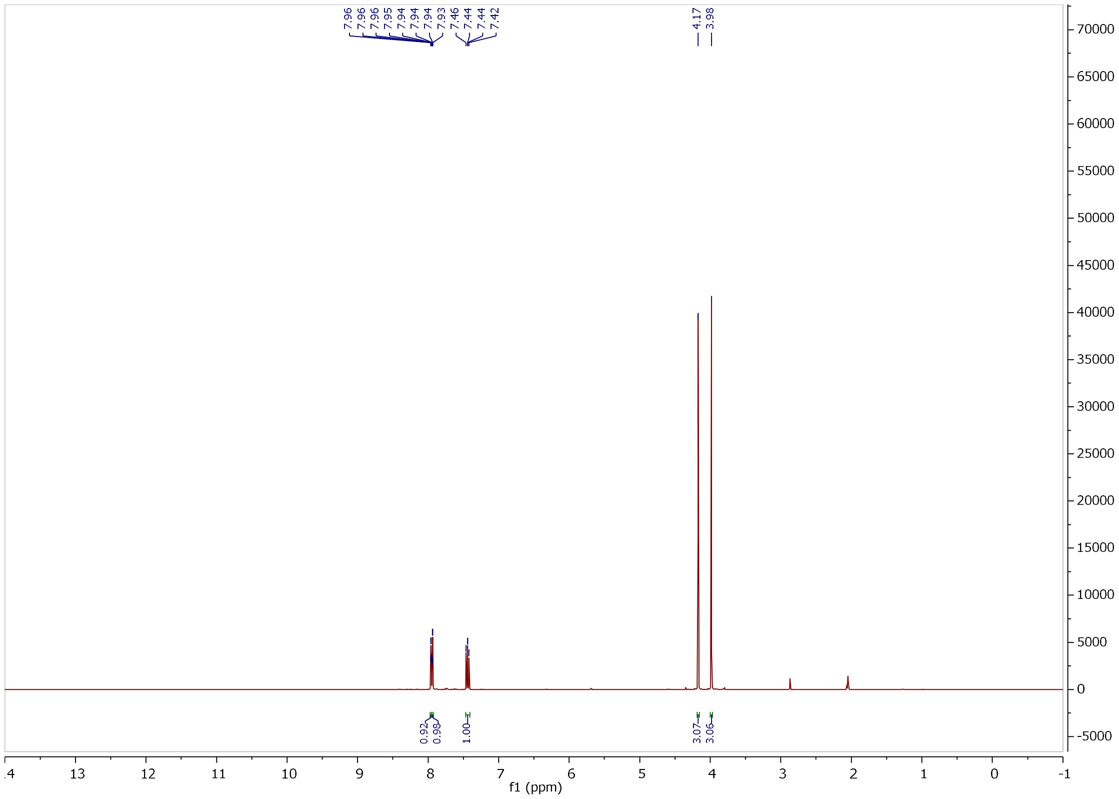
7.96
7.96
7.96
7.95
7.94
7.94
7.94
7.93
7.46
7.44
7.44
7.42
4.17
3.98
0.92
0.98
1.00
3.07
3.06
f1 (ppm)

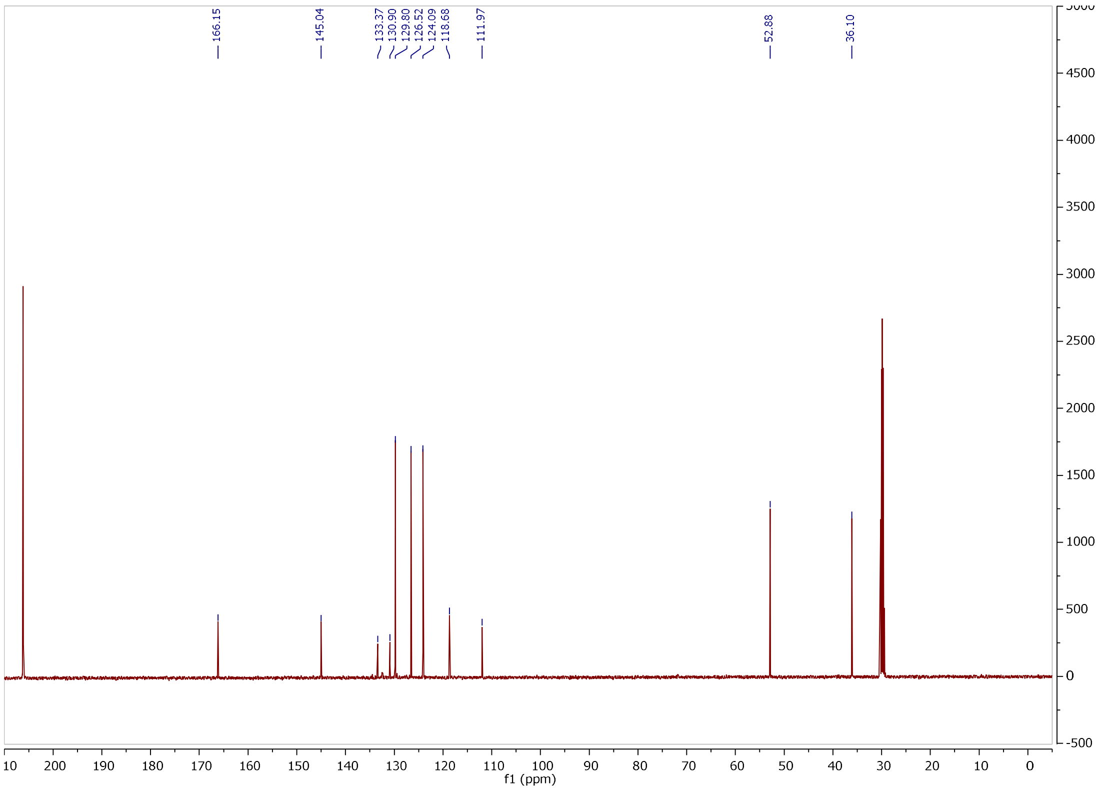
166.15
145.04
133.37
130.90
129.80
126.52
124.09
118.68
111.97
52.88
36.10
f1 (ppm)

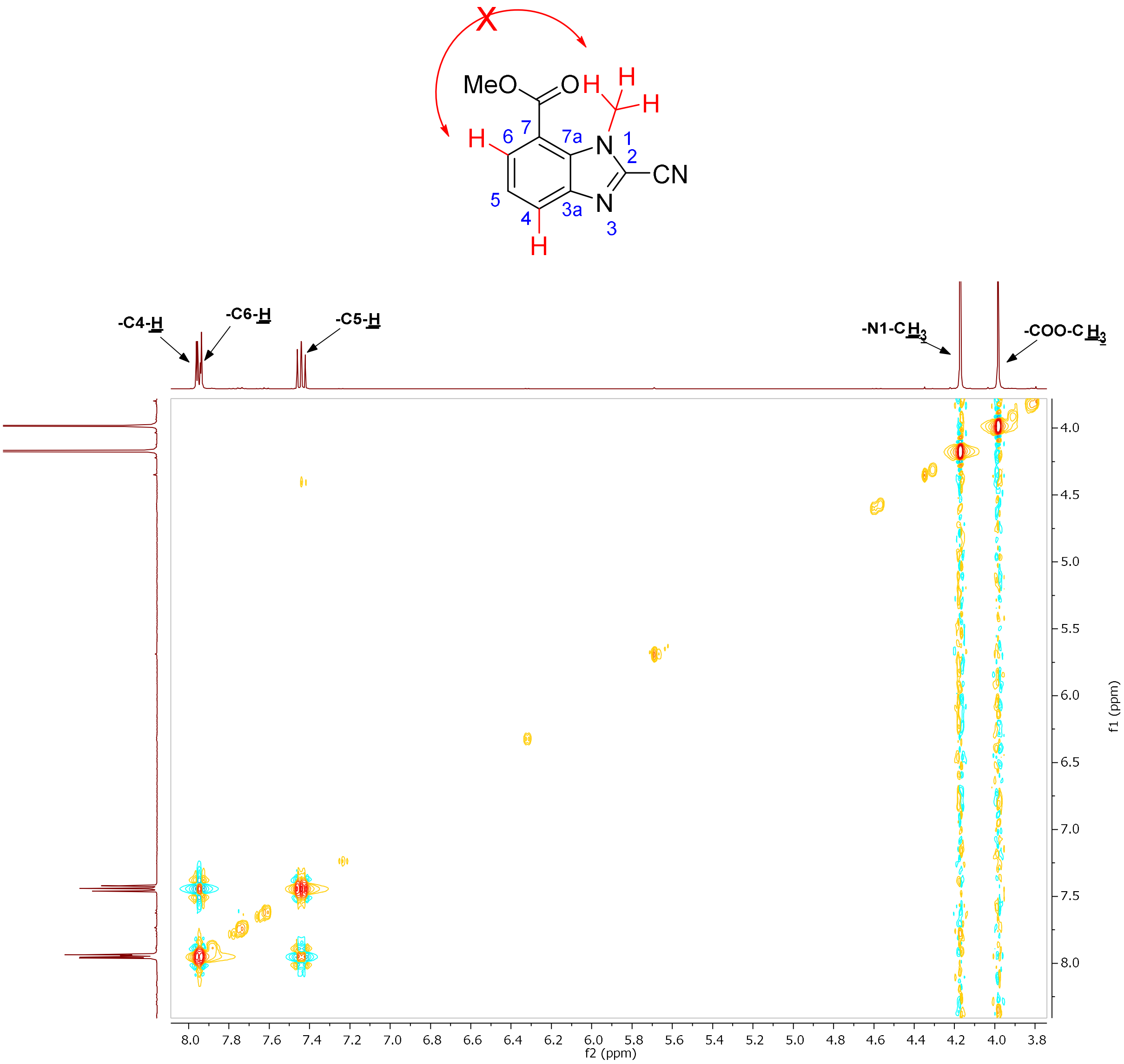

MeO
O
H
H
H
N
CN
N
1
2
3
3a
4
5
6
7
7a
-C4-H
-C6-H
-C5-H
-N1-CH3
-COO-CH3
f2 (ppm)
f1 (ppm)

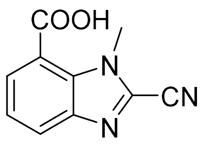
COOH
N
CN
N

Compound 45

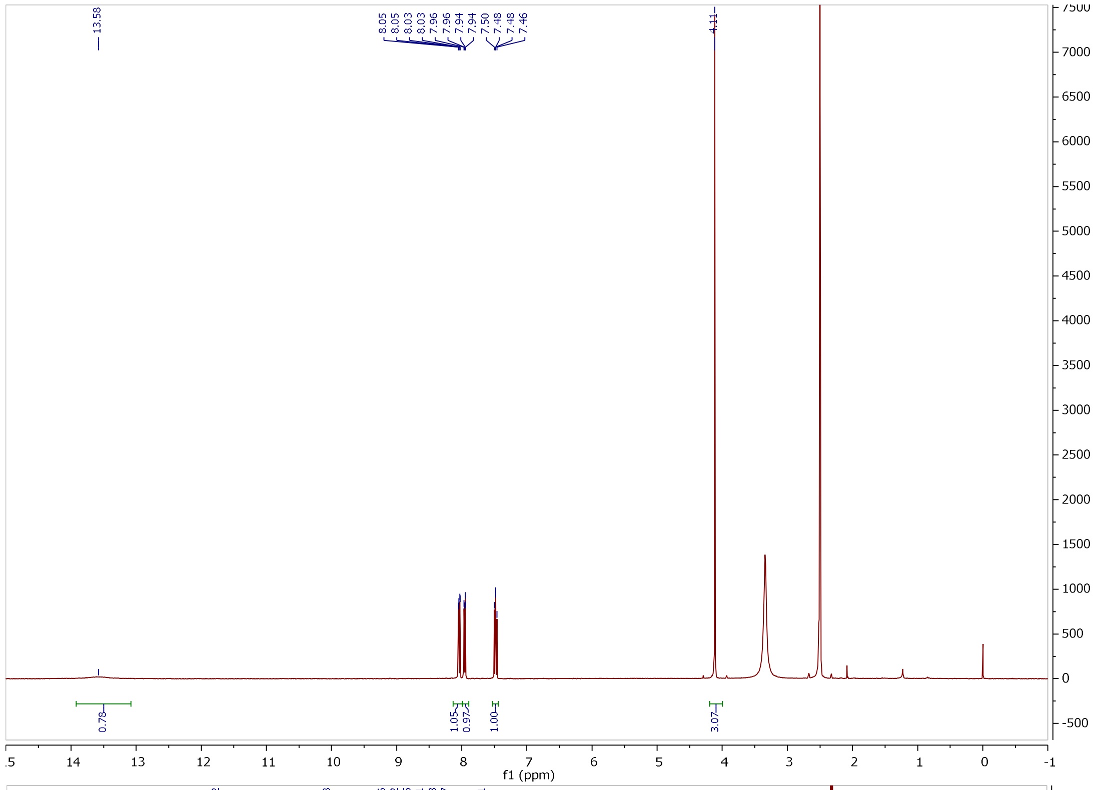
13.58
8.05
8.05
8.03
8.03
7.96
7.96
7.94
7.94
7.50
7.48
7.48
7.46
4.11
0.78
1.05
0.97
1.00
3.07
f1 (ppm)

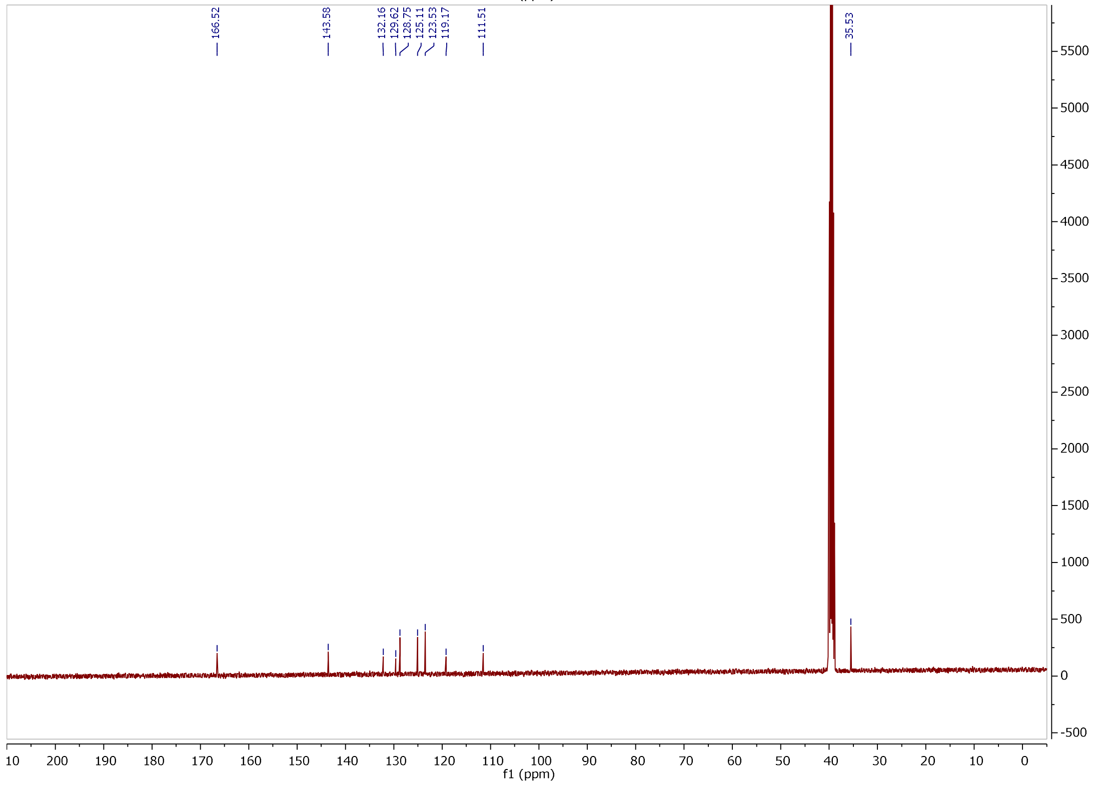
166.52
143.58
132.16
129.62
128.75
125.11
123.53
119.17
111.51
35.53
f1 (ppm)

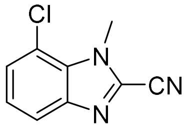
Cl
N
CN
N

Compound 46

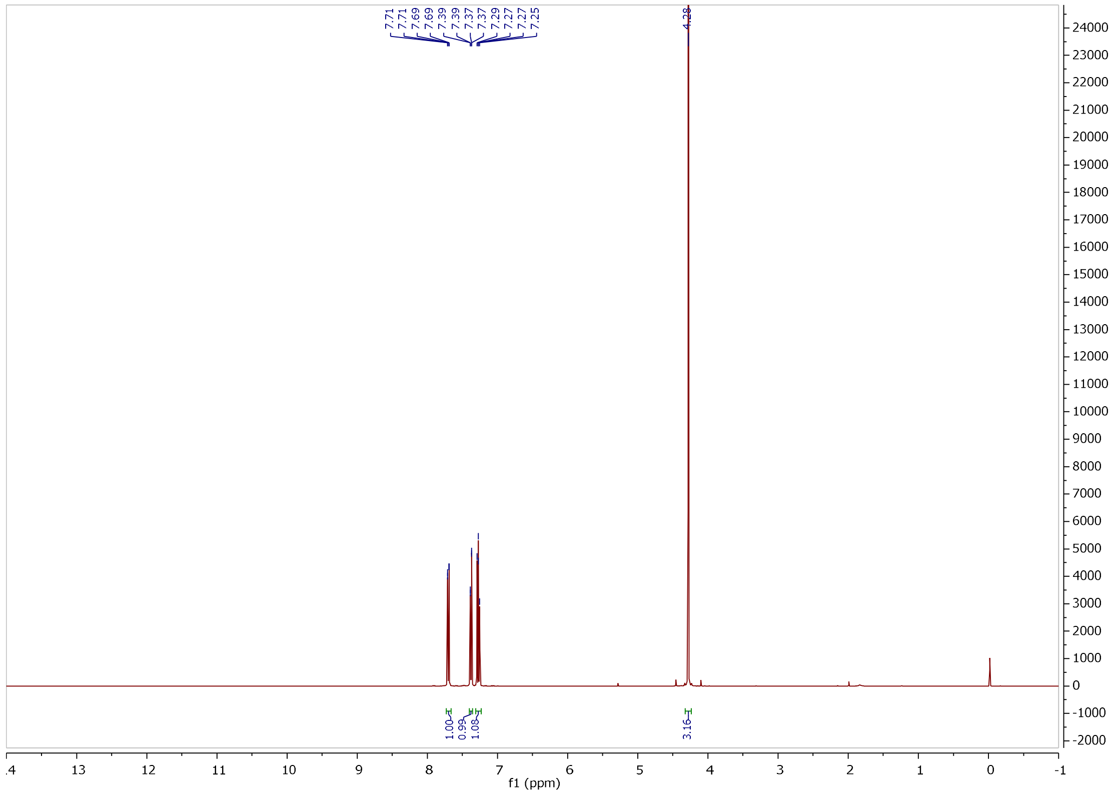
7.71
7.71
7.69
7.69
7.39
7.39
7.37
7.37
7.29
7.27
7.27
7.25
4.28
1.00
0.99
1.08
3.16
f1 (ppm)

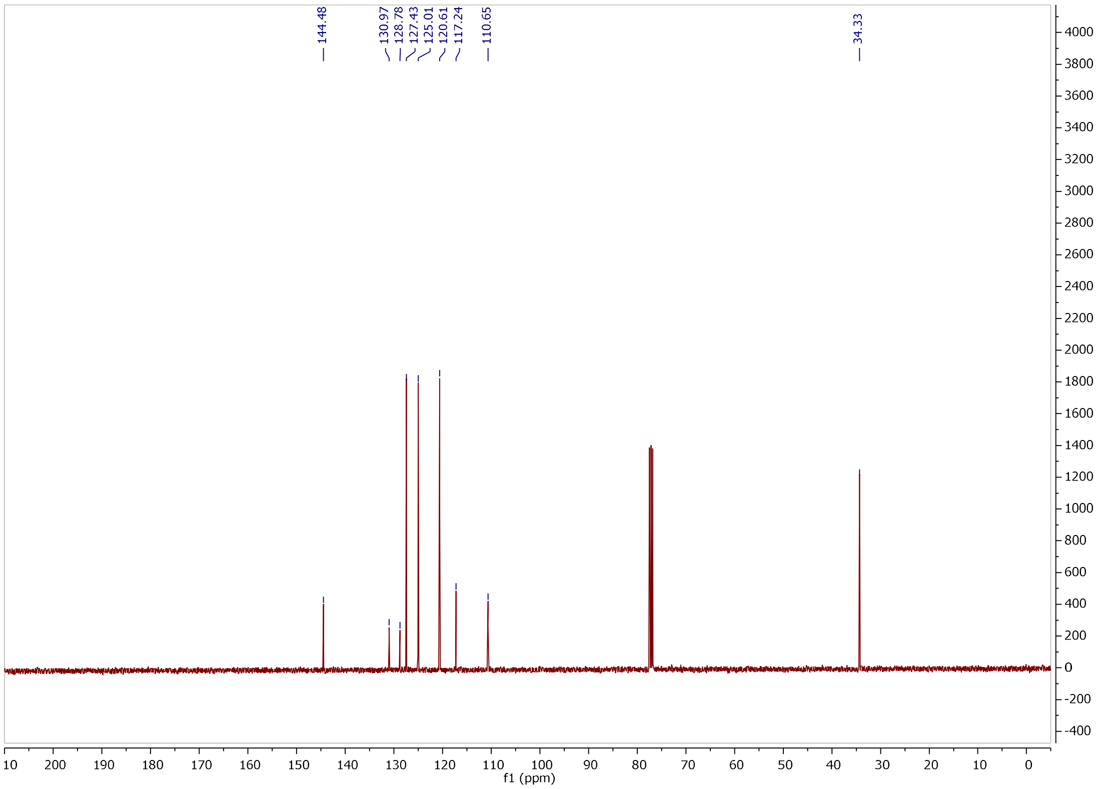
144.48
130.97
128.78
127.43
125.01
120.61
117.24
110.65
34.33
f1 (ppm)

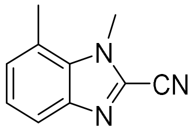

Compound 47

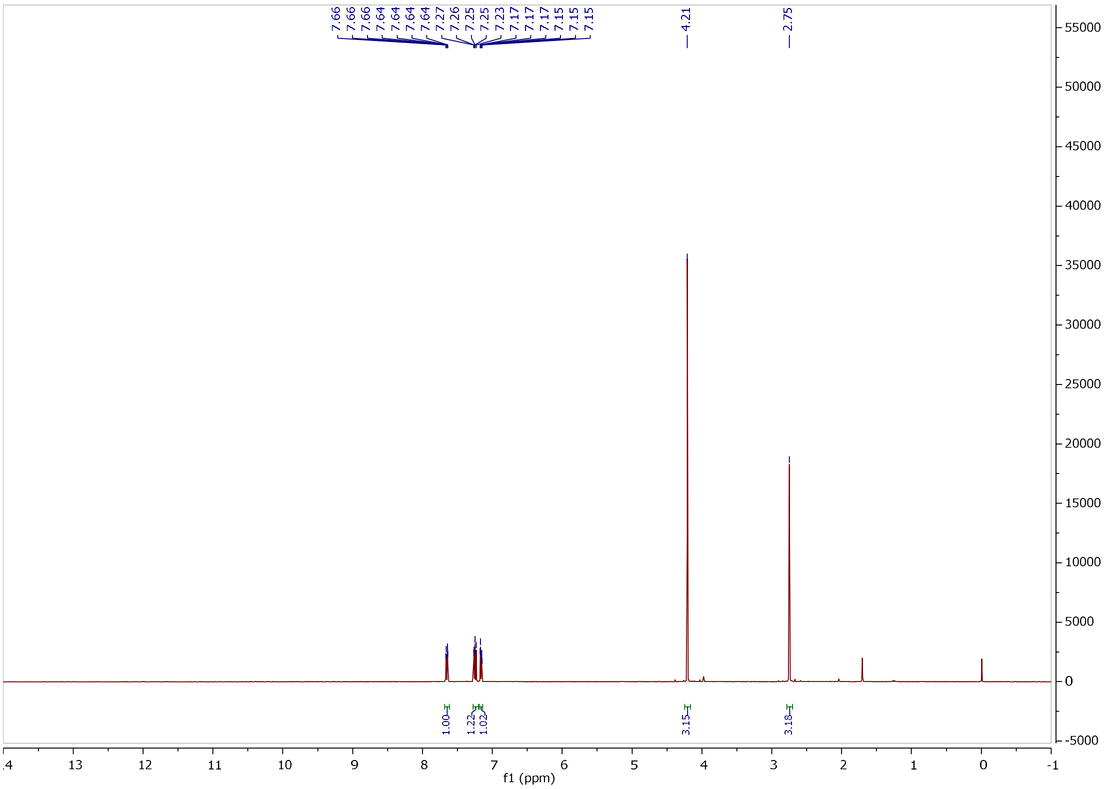
7.66
7.66
7.66
7.64
7.64
7.64
7.27
7.26
7.25
7.25
7.23
7.17
7.17
7.17
7.15
7.15
7.15
4.21
2.75
1.00
1.22
1.02
3.15
3.18
f1 (ppm)

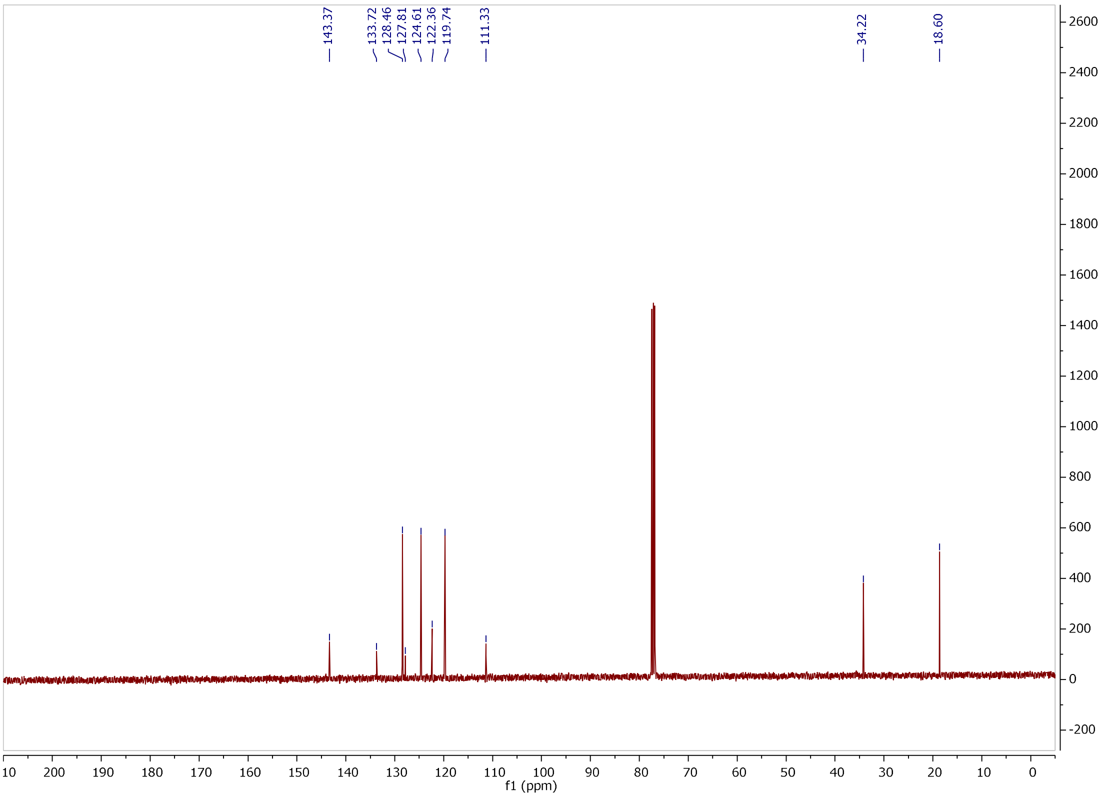
143.37
133.72
128.46
127.81
124.61
122.36
119.74
111.33
34.22
18.60
f1 (ppm)

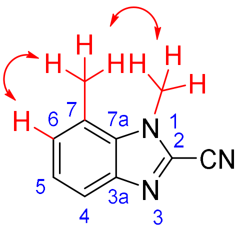

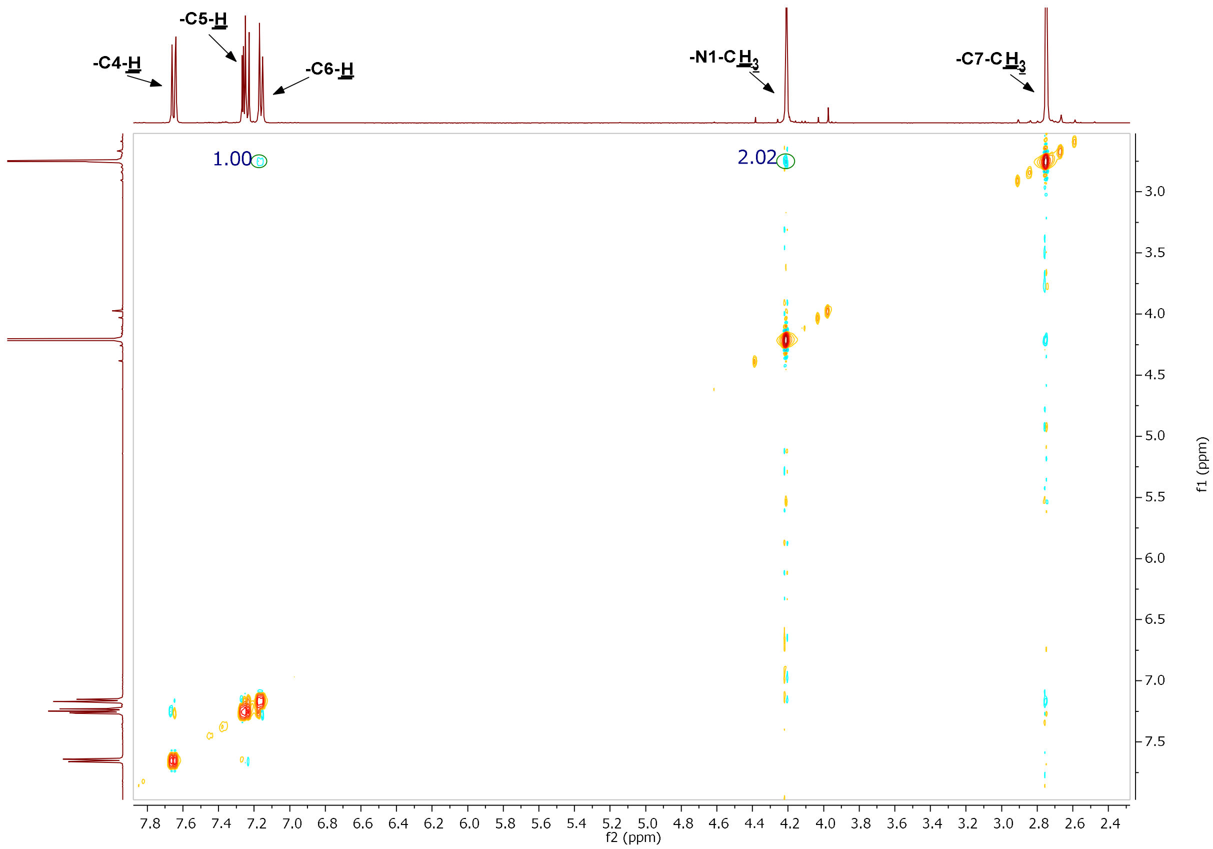

-C4-H
-C5-H
-C6-H
-N1-CH3
-C7-CH3
1.00
2.02
f2 (ppm)
f1 (ppm)

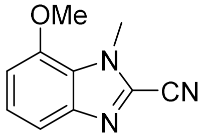

Compound 48

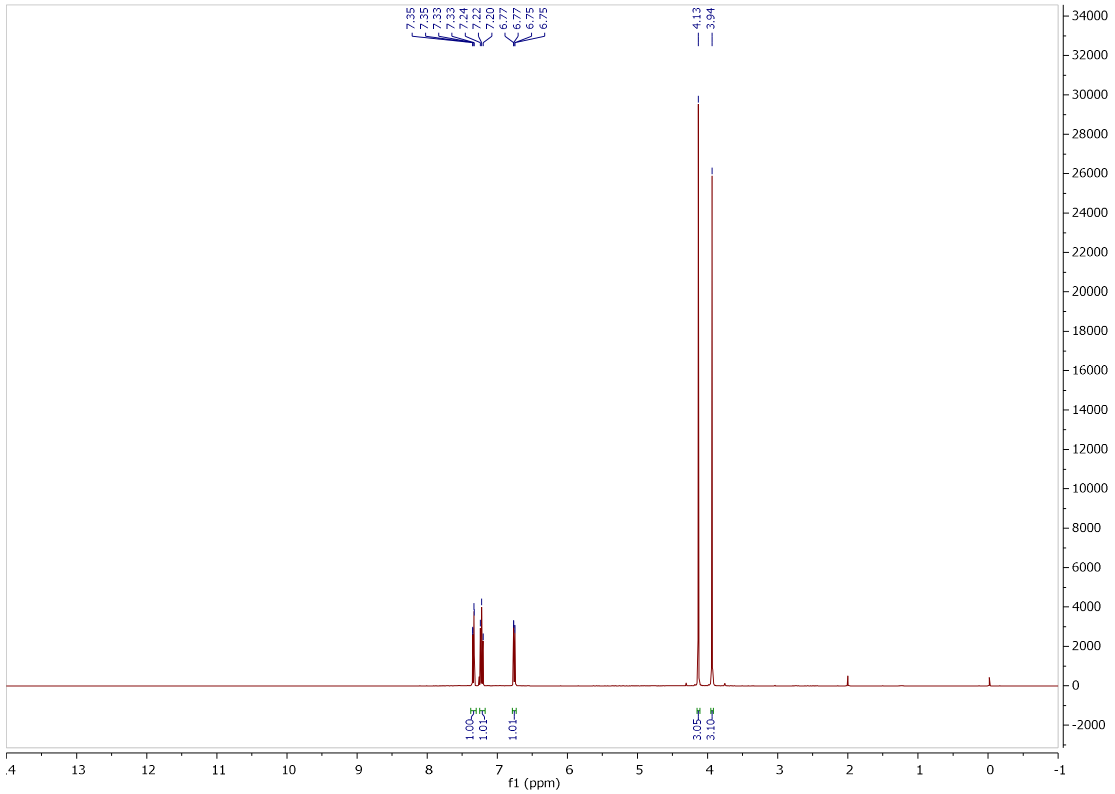

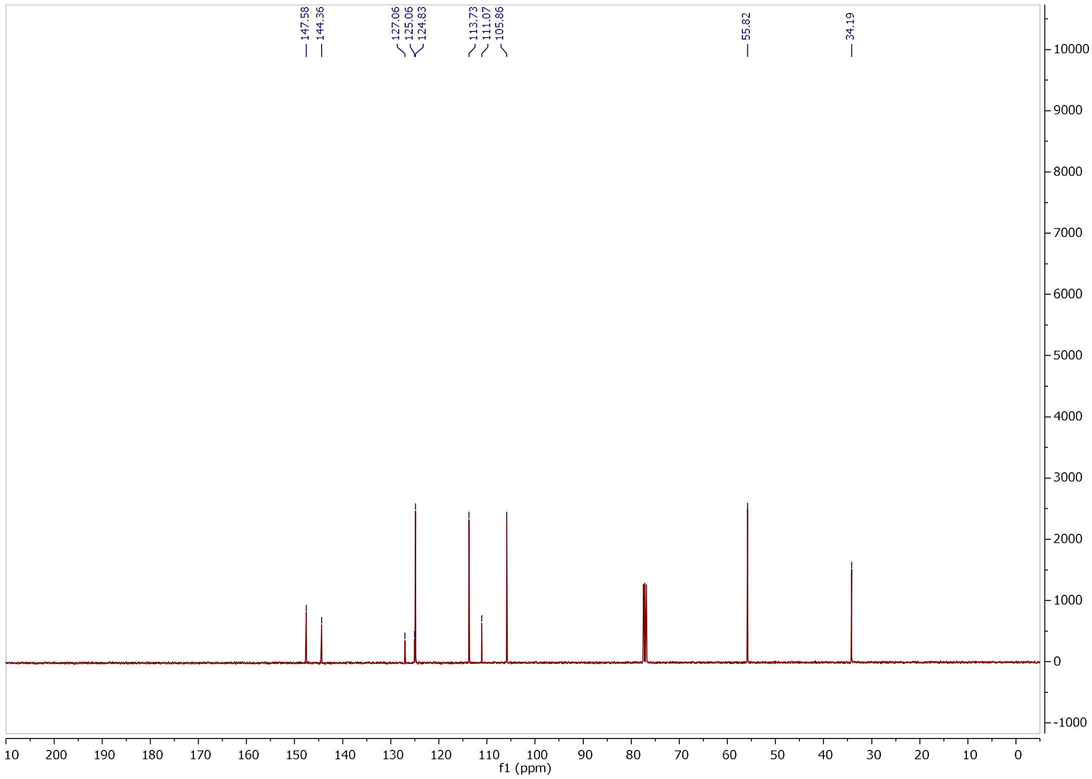

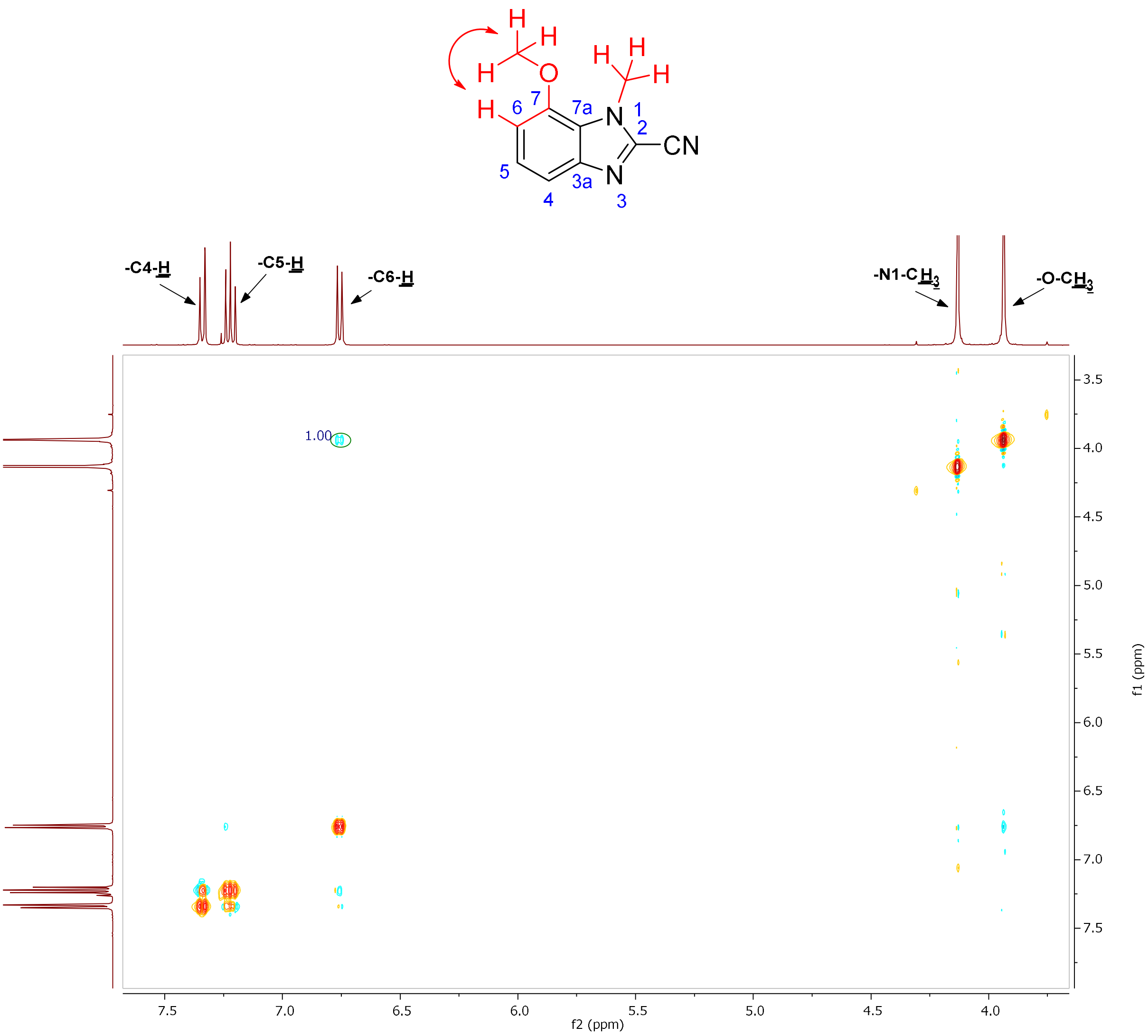
CN
-C4-H
-C5-H
-C6-H
-N1-CH3
-O-CH3
1.00
f2 (ppm)
f1 (ppm)

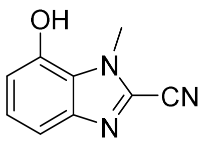
OH
N
CN
N

Compound 49

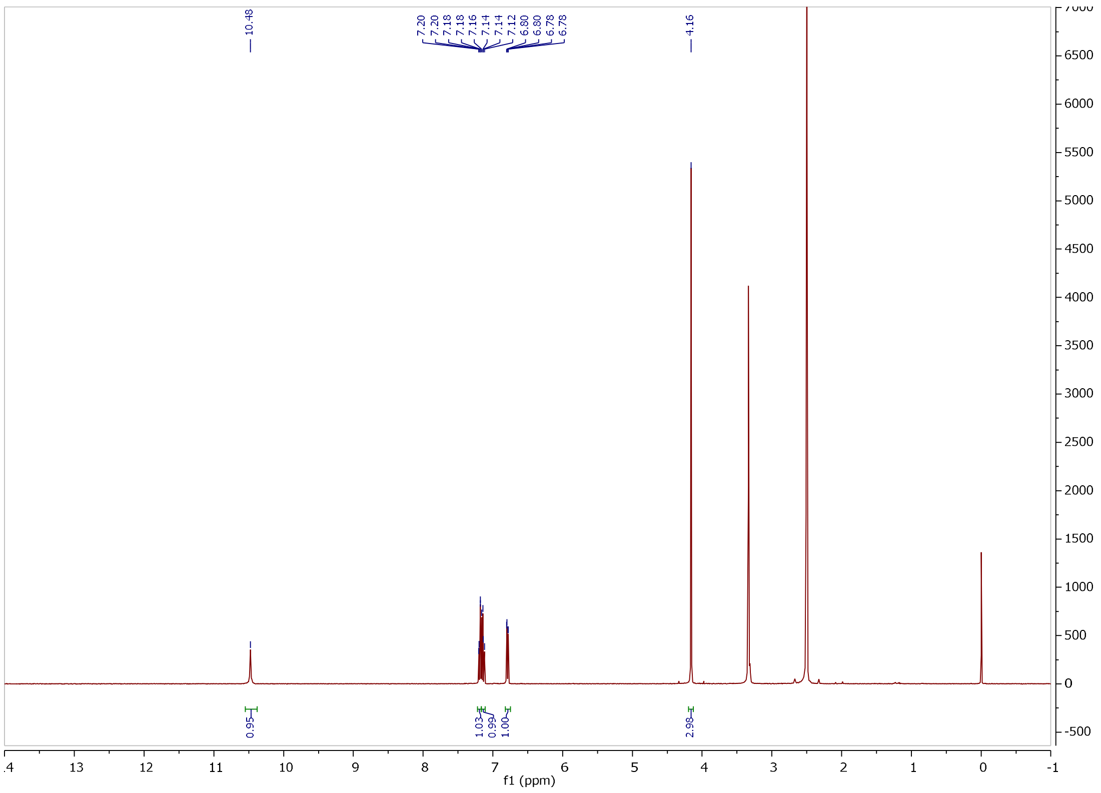
10.48
7.20
7.20
7.18
7.18
7.16
7.14
7.14
7.12
6.80
6.80
6.78
6.78
4.16
0.95
1.03
0.99
1.00
2.98
f1 (ppm)

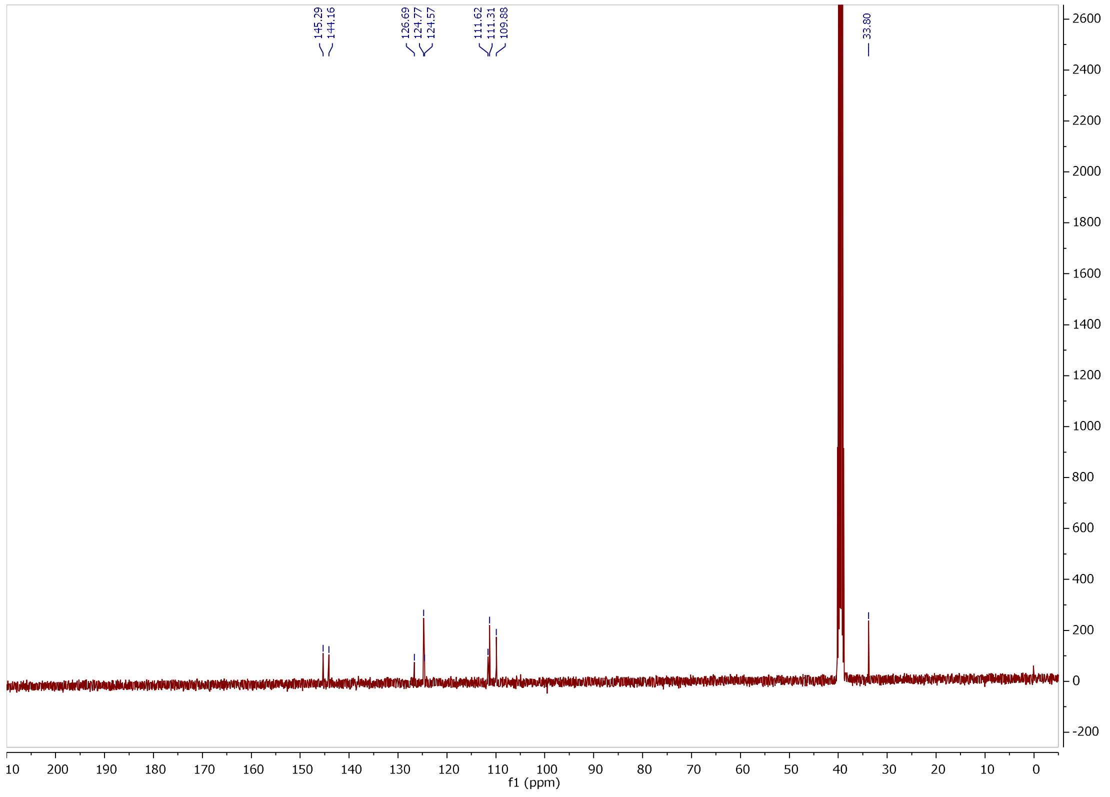
145.29
144.16
126.69
124.77
124.57
111.62
111.31
109.88
33.80
f1 (ppm)

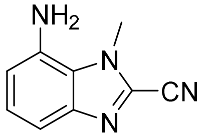
NH2
N
CN
N

Compound 50

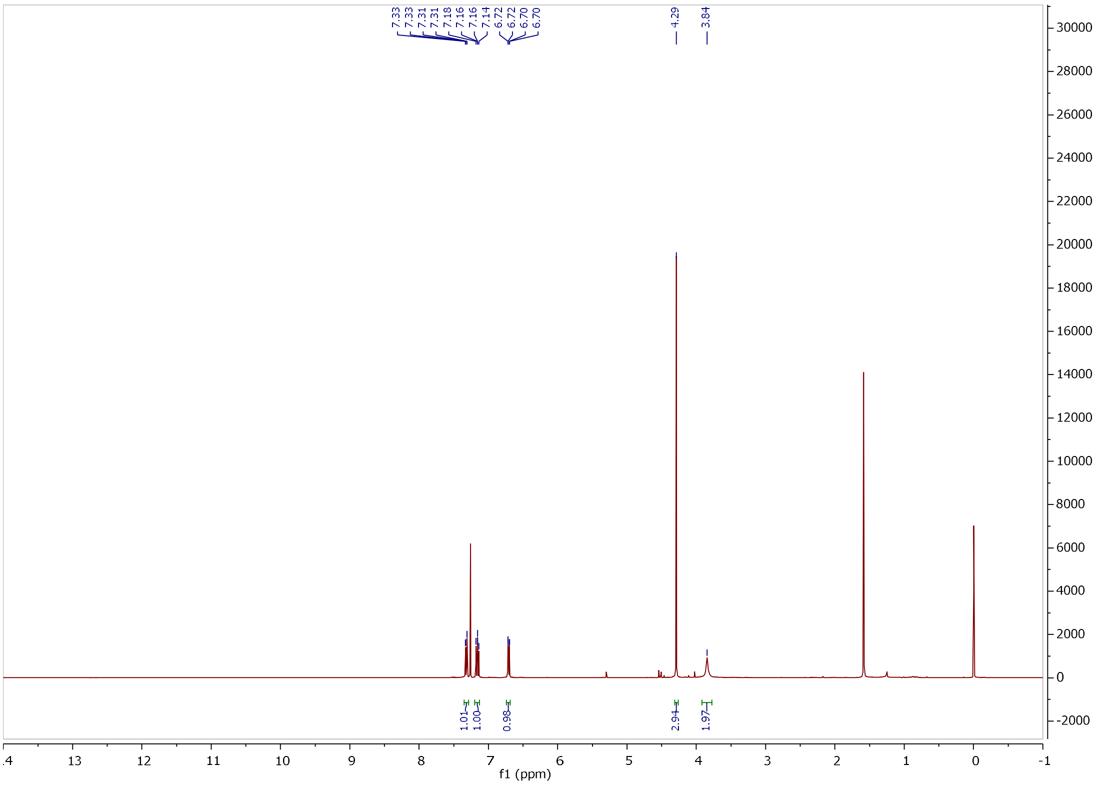
7.33
7.33
7.31
7.31
7.18
7.16
7.16
7.14
6.72
6.72
6.70
6.70
4.29
3.84
1.01
1.00
0.98
2.94
1.97
f1 (ppm)

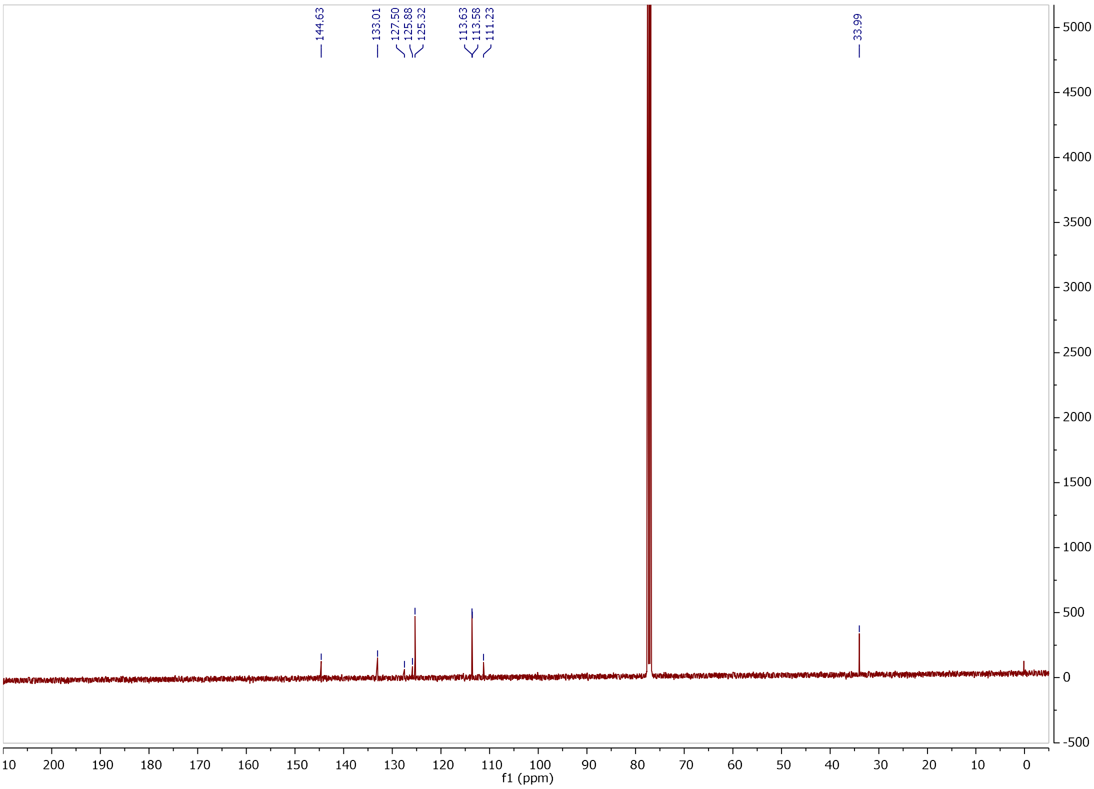
144.63
133.01
127.50
125.88
125.32
113.63
113.58
111.23
33.99
f1 (ppm)

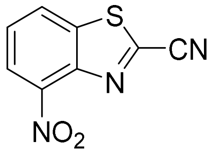
S
CN
N
NO2

Compound 52

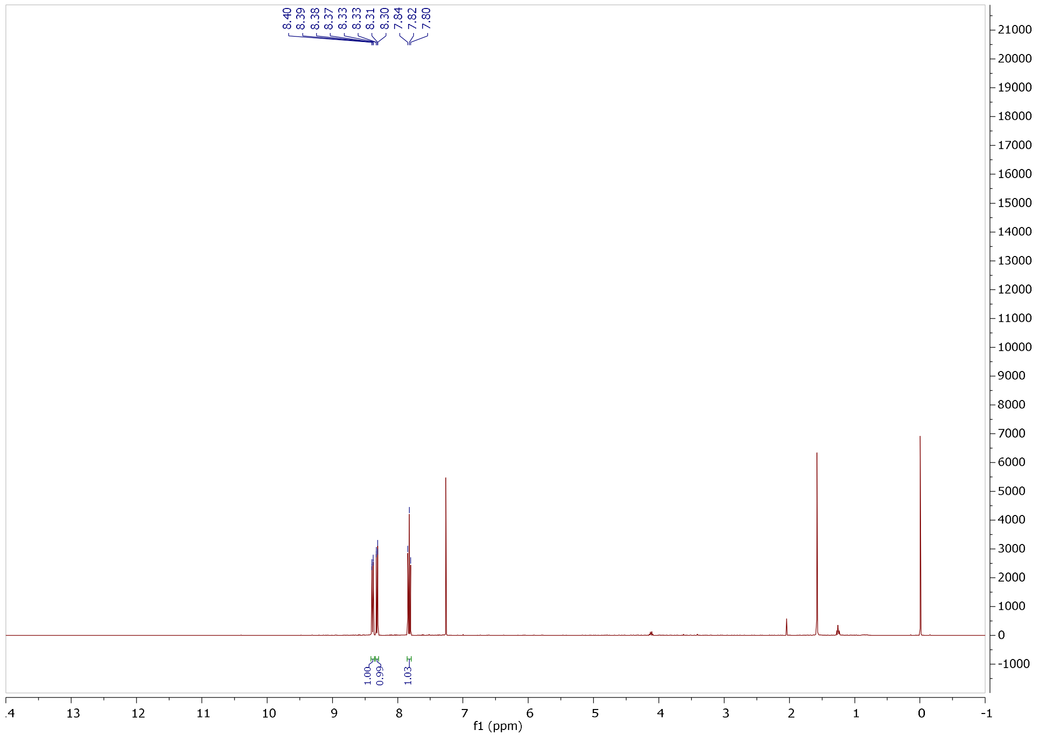
8.40
8.39
8.38
8.37
8.33
8.33
8.31
8.30
7.84
7.82
7.80
1.00
0.99
1.03
f1 (ppm)

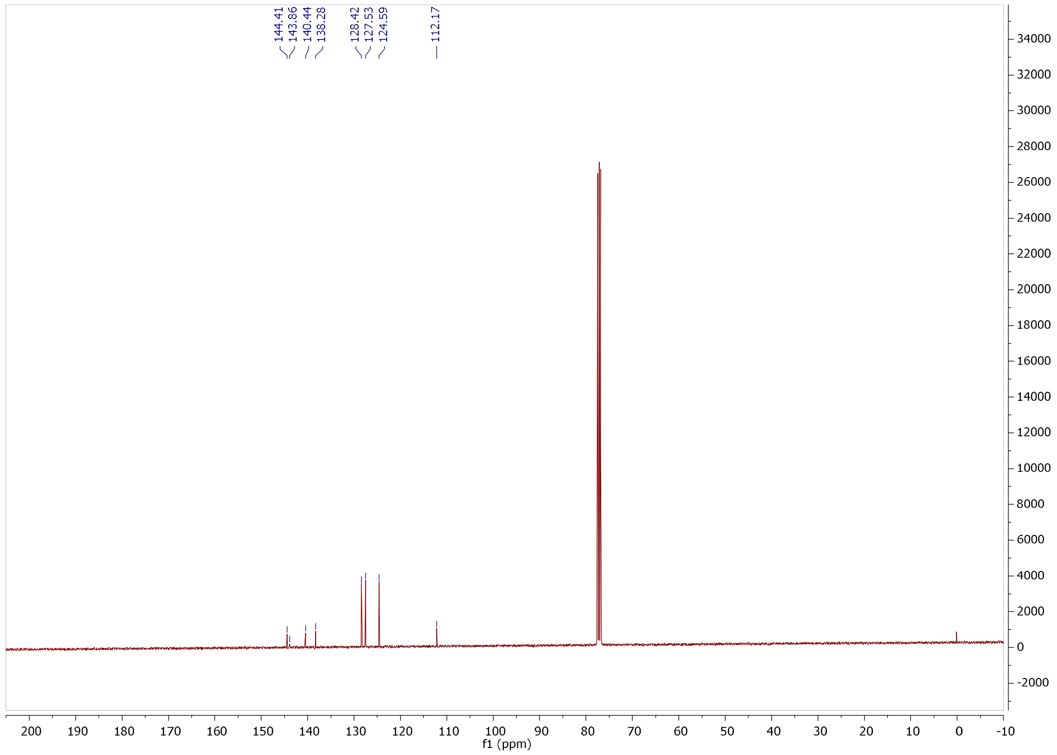
144.41
143.86
140.44
138.28
128.42
127.53
124.59
112.17
f1 (ppm)

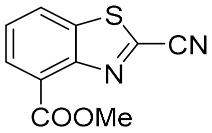

Compound 53

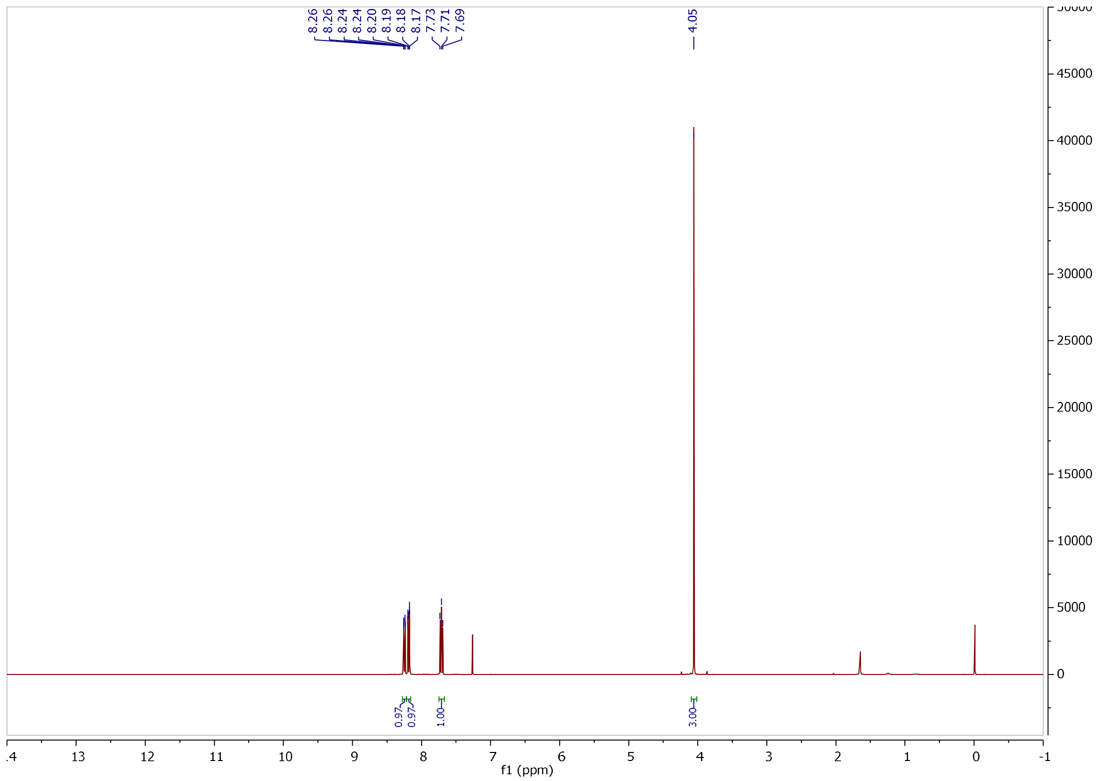

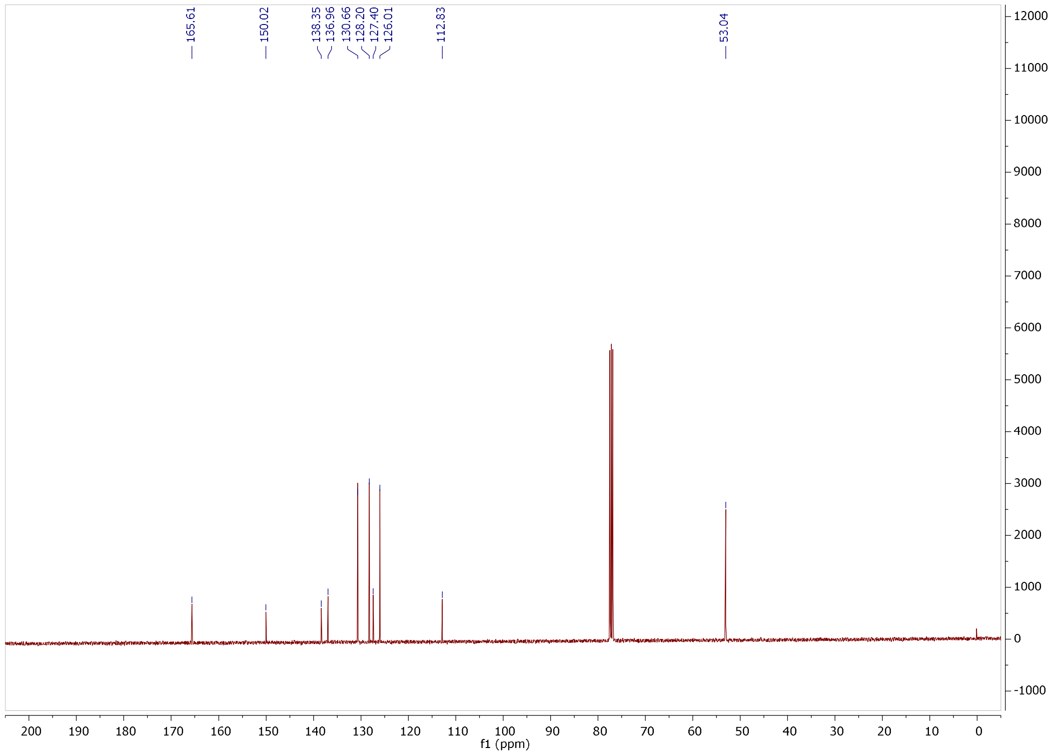

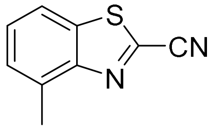
S
CN
N

Compound 56

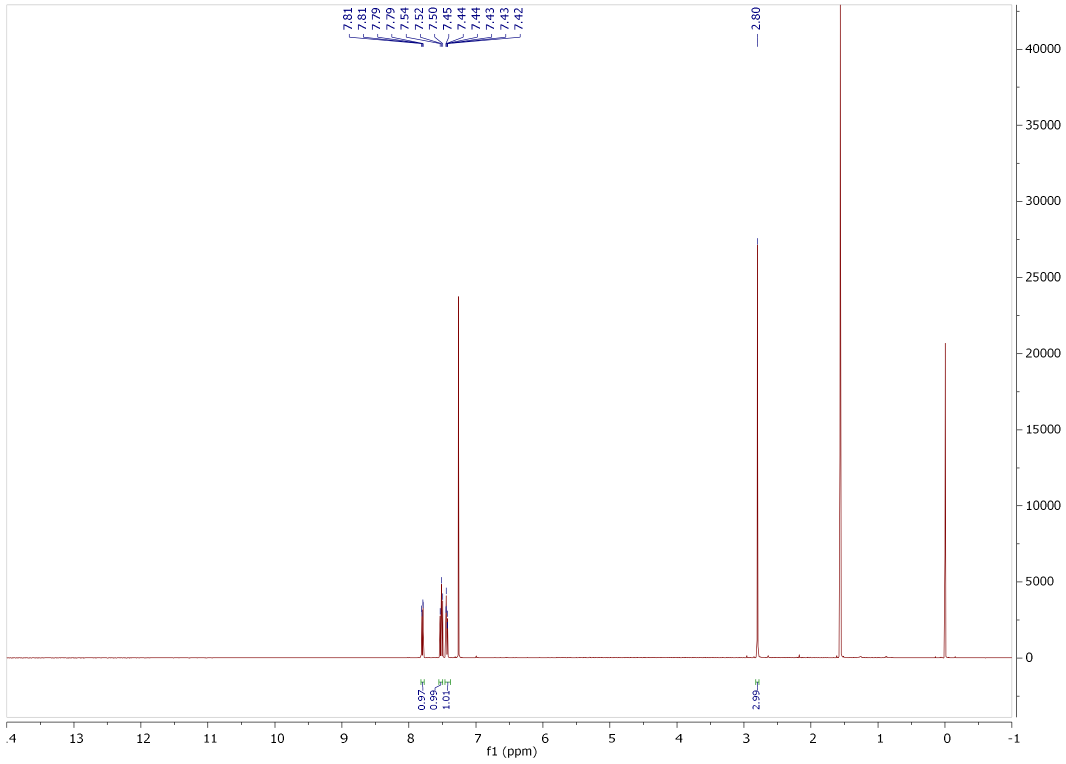
7.81
7.81
7.79
7.79
7.54
7.52
7.50
7.45
7.44
7.44
7.43
7.43
7.42
2.80
0.97
0.99
1.01
2.99
f1 (ppm)

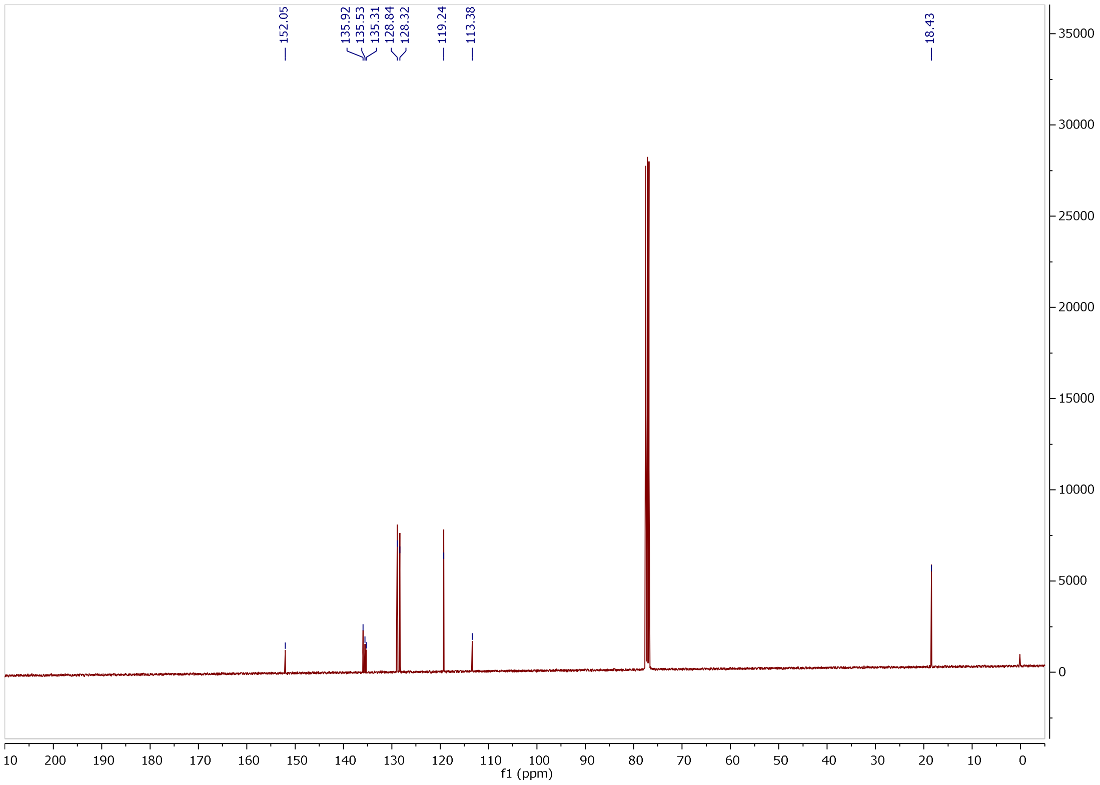
152.05
135.92
135.53
135.31
128.84
128.32
119.24
113.38
18.43
f1 (ppm)

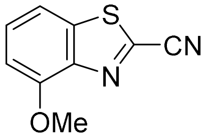

Compound 57

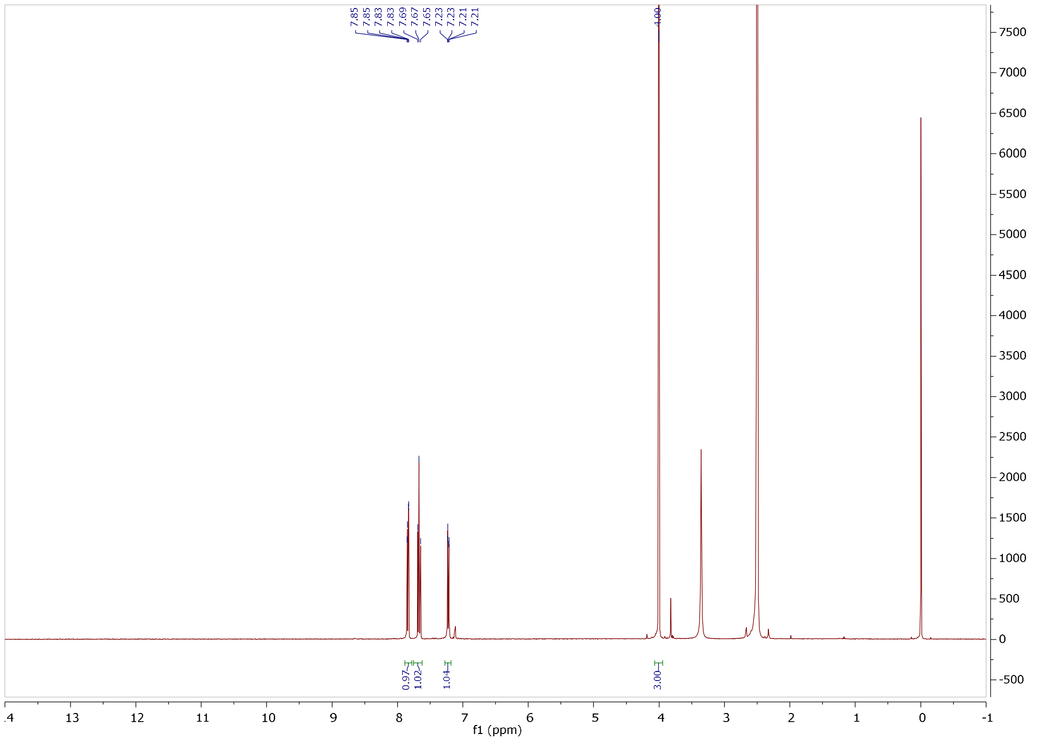

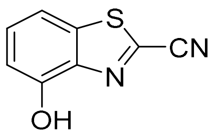
S
CN
N
OH

Compound 58

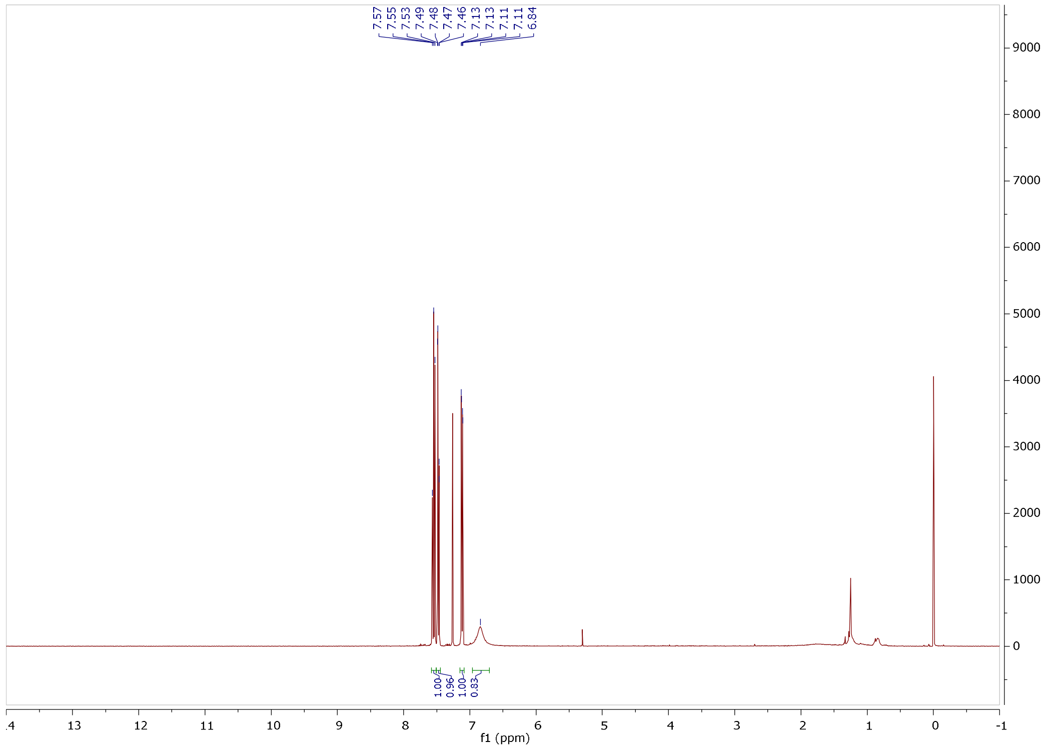
7.57
7.55
7.53
7.49
7.48
7.47
7.46
7.13
7.13
7.11
7.11
6.84
1.00
0.96
1.00
0.83
f1 (ppm)

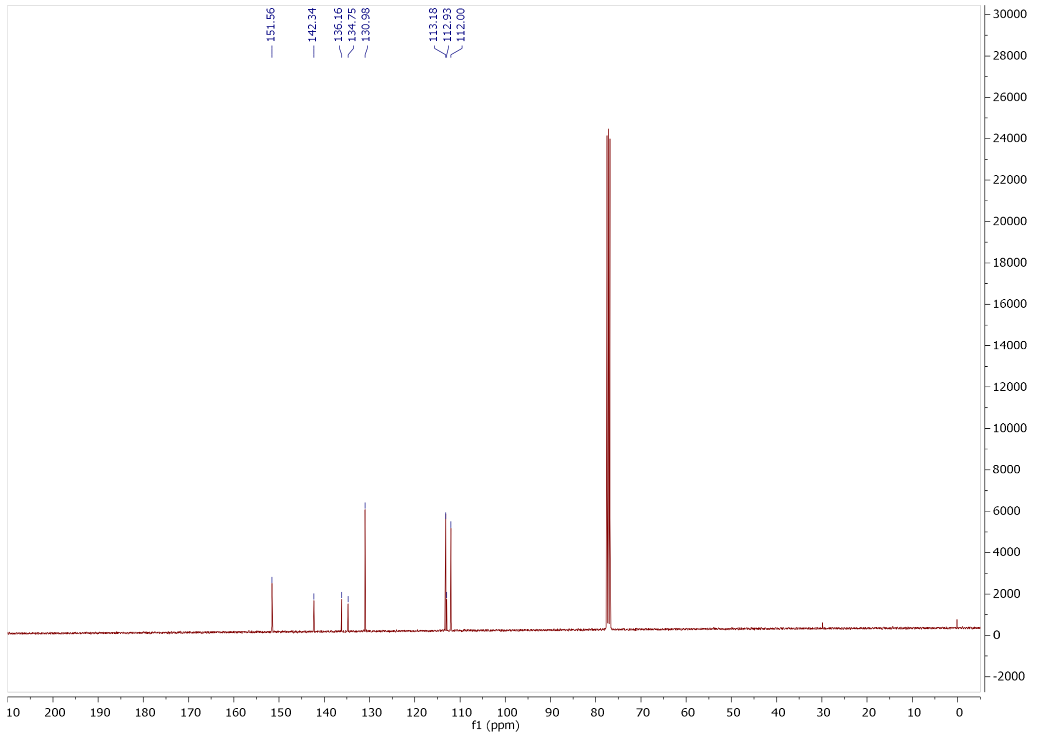
151.56
142.34
136.16
134.75
130.98
113.18
112.93
112.00
f1 (ppm)

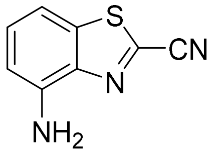
S
CN
N
NH2

Compound 59

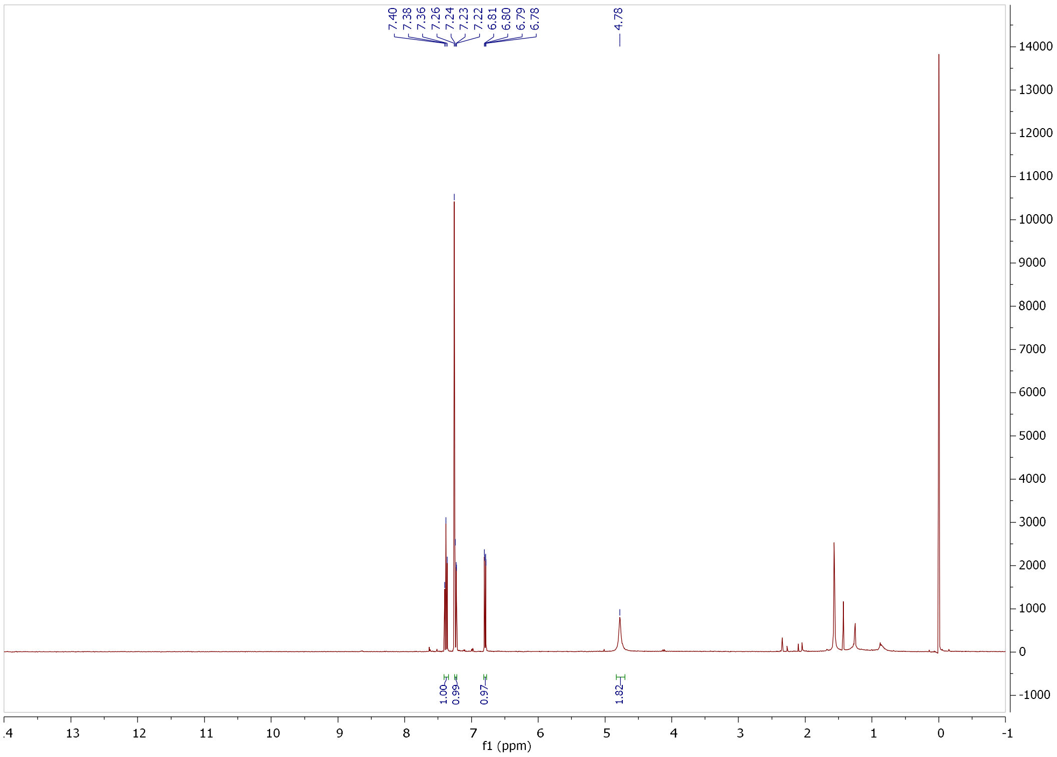
7.40
7.38
7.36
7.26
7.24
7.23
7.22
6.81
6.80
6.79
6.78
4.78
1.00
0.99
0.97
1.82
f1 (ppm)

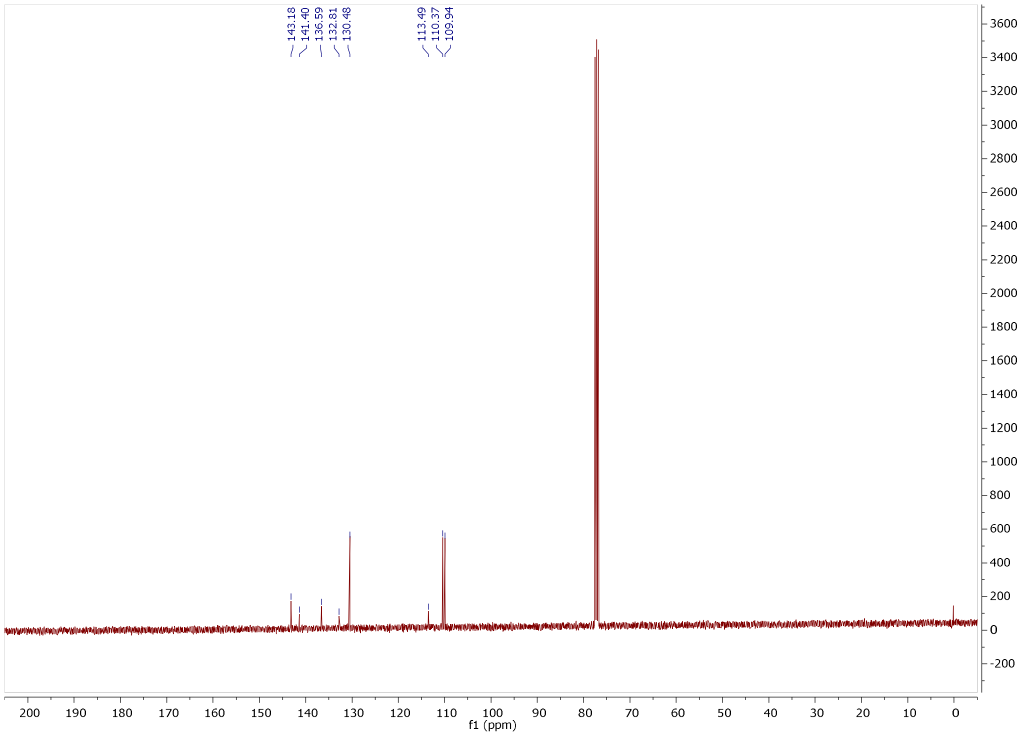
143.18
141.40
136.59
132.81
130.48
113.49
110.37
109.94
f1 (ppm)

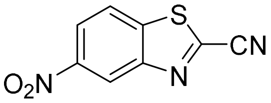

Compound 60

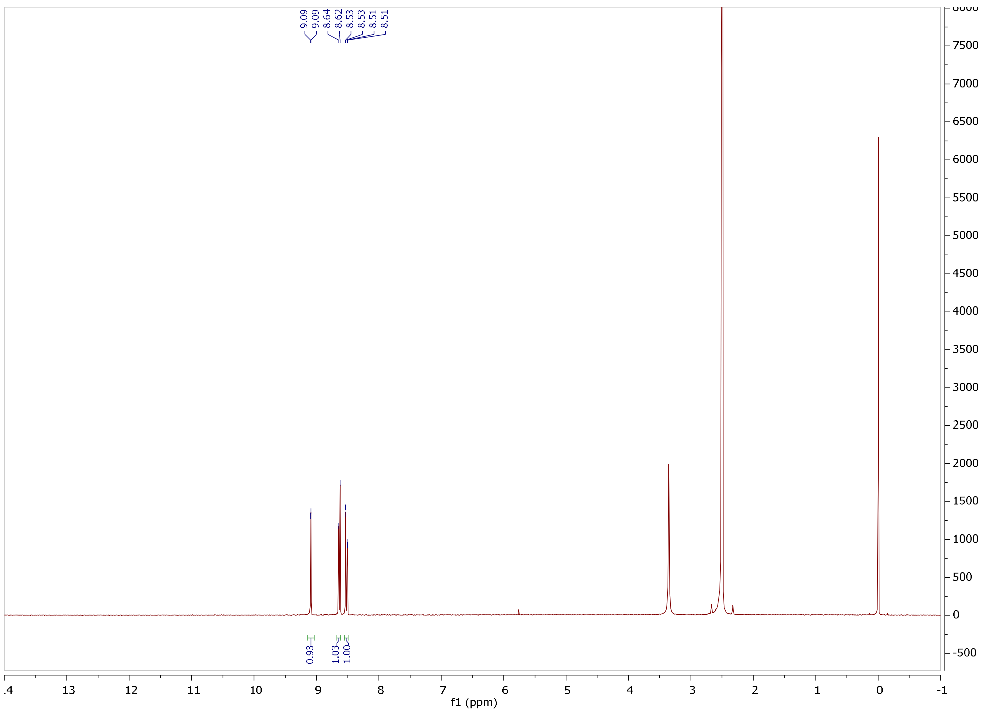

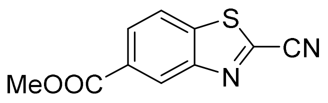
S
CN
N
MeOOC

Compound 61

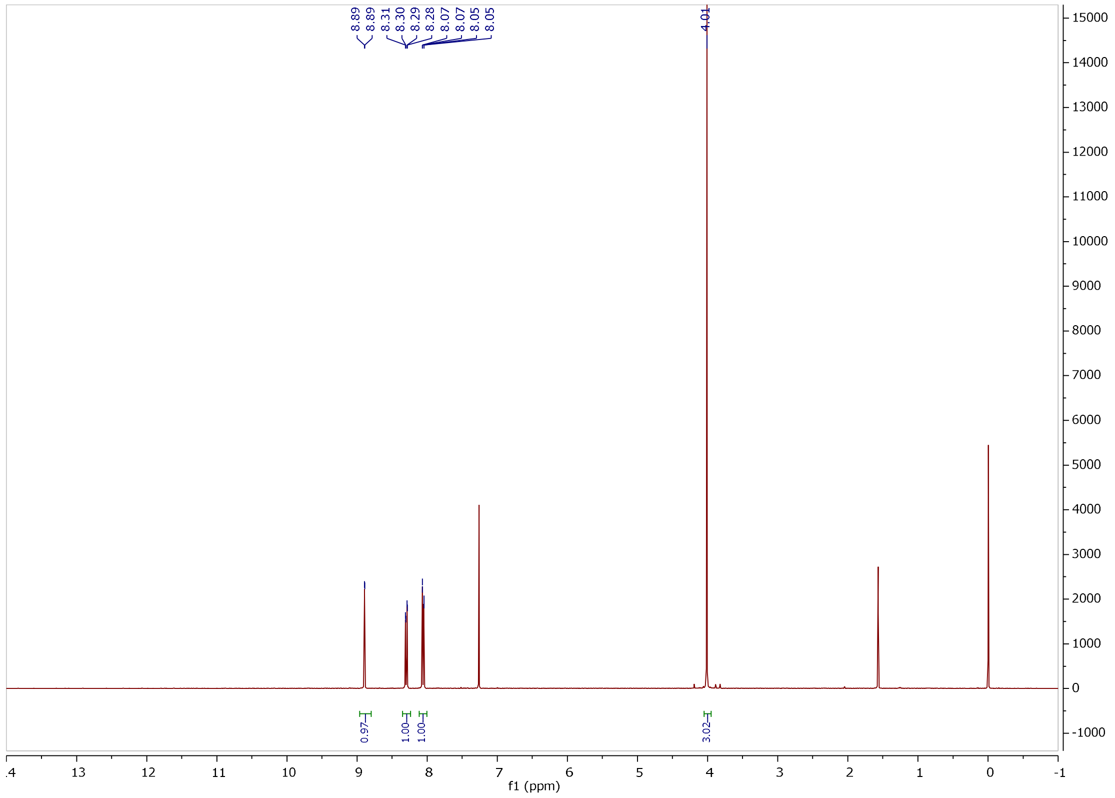
8.89
8.89
8.31
8.30
8.29
8.28
8.07
8.07
8.05
8.05
4.01
0.97
1.00
1.00
3.02
f1 (ppm)

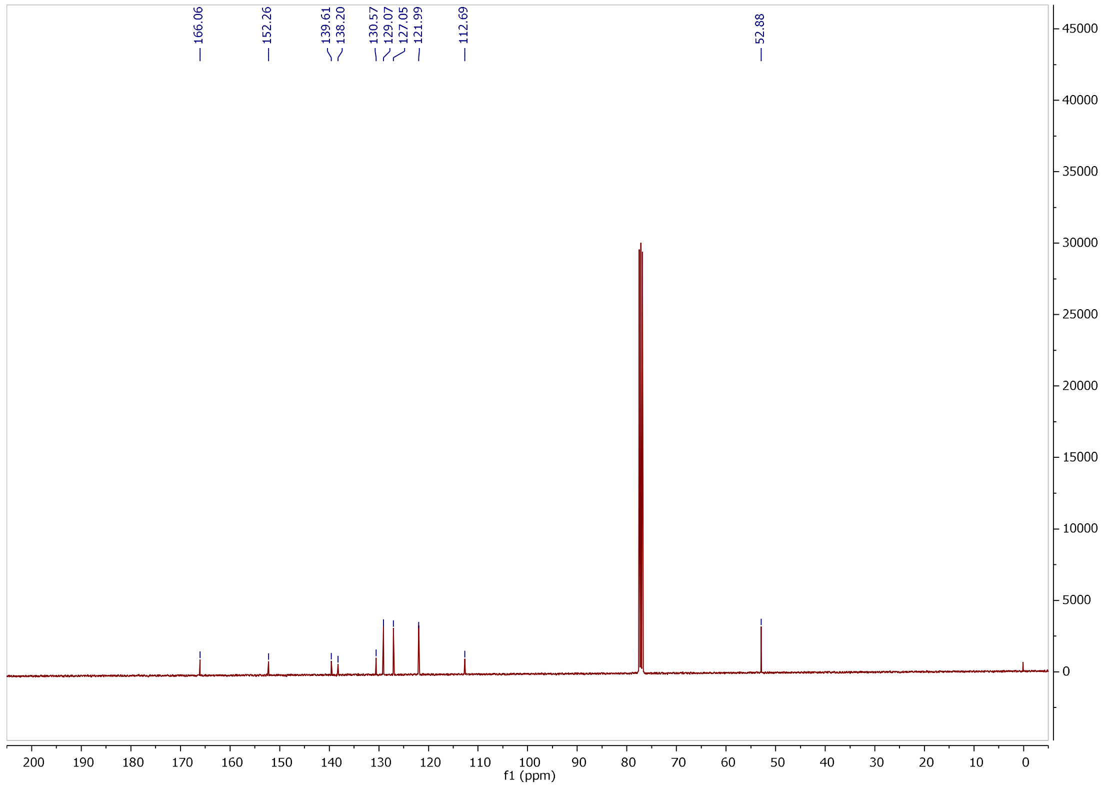
166.06
152.26
139.61
138.20
130.57
129.07
127.05
121.99
112.69
52.88
f1 (ppm)

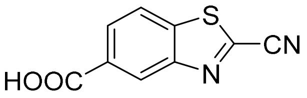

HOOC
S
N
CN

Compound 62

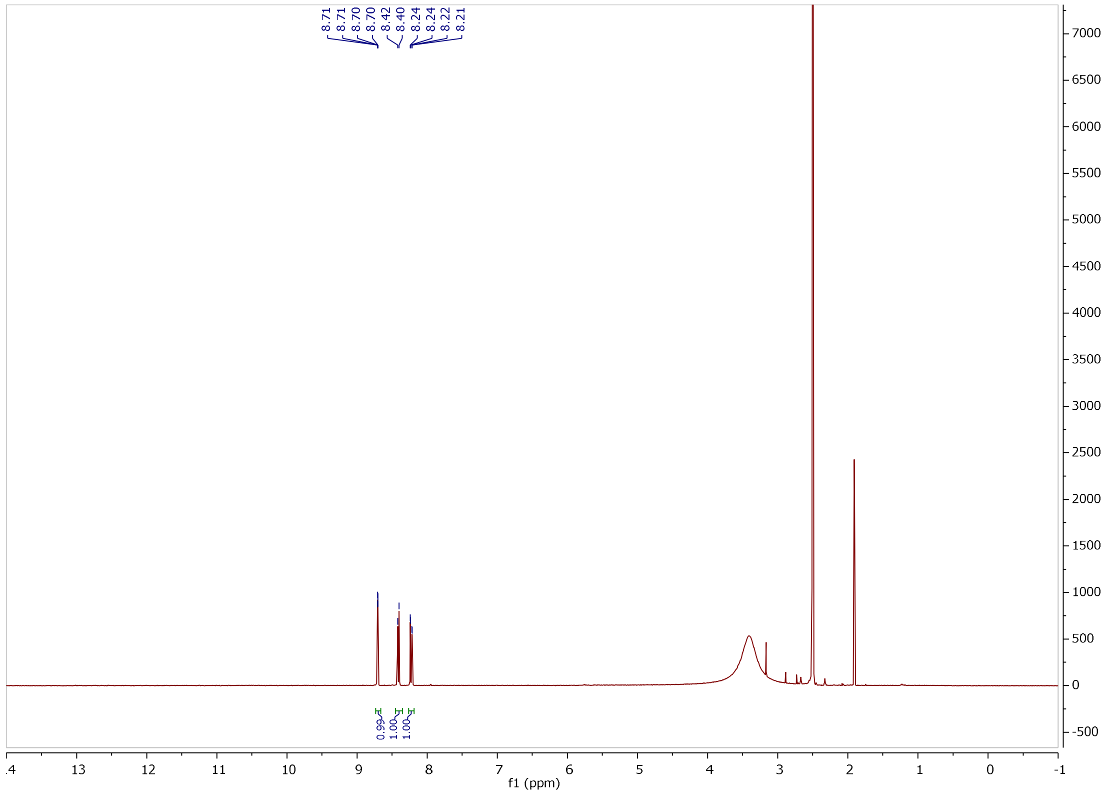

8.71
8.71
8.70
8.70
8.42
8.40
8.24
8.24
8.22
8.21
0.99
1.00
1.00
f1 (ppm)

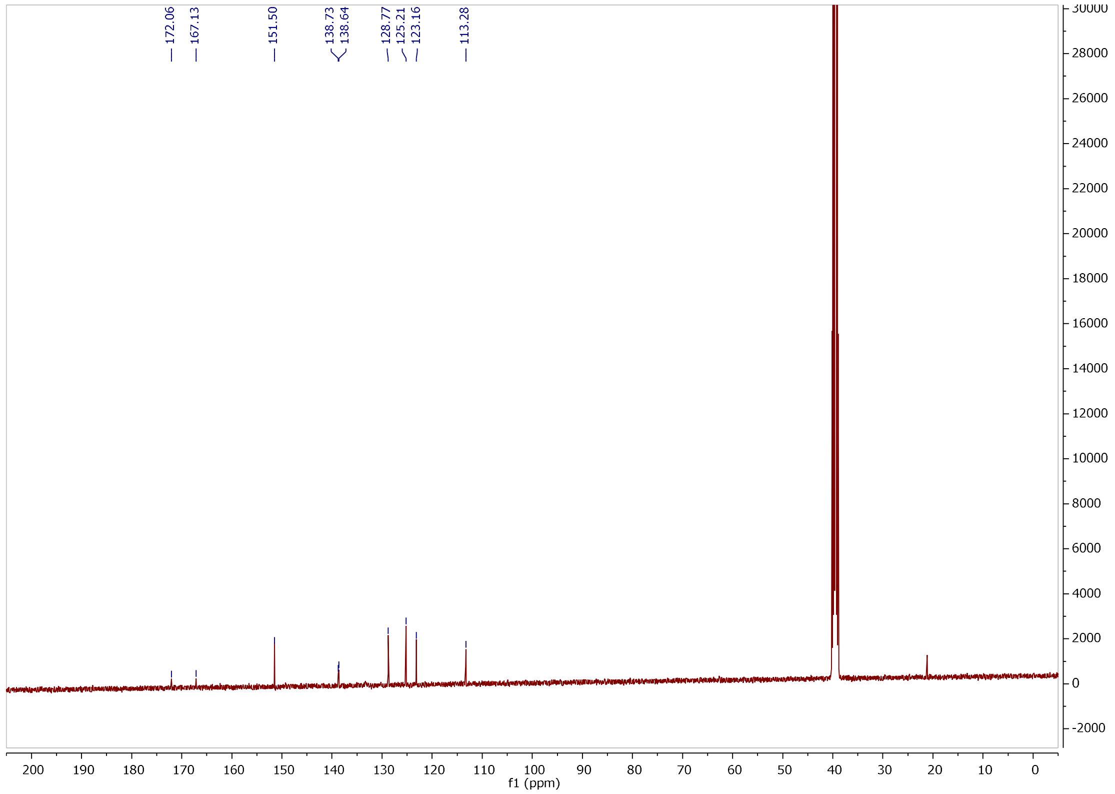

172.06
167.13
151.50
138.73
138.64
128.77
125.21
123.16
113.28
f1 (ppm)

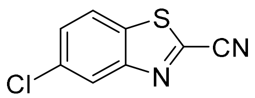
S
CN
N
Cl

Compound 63

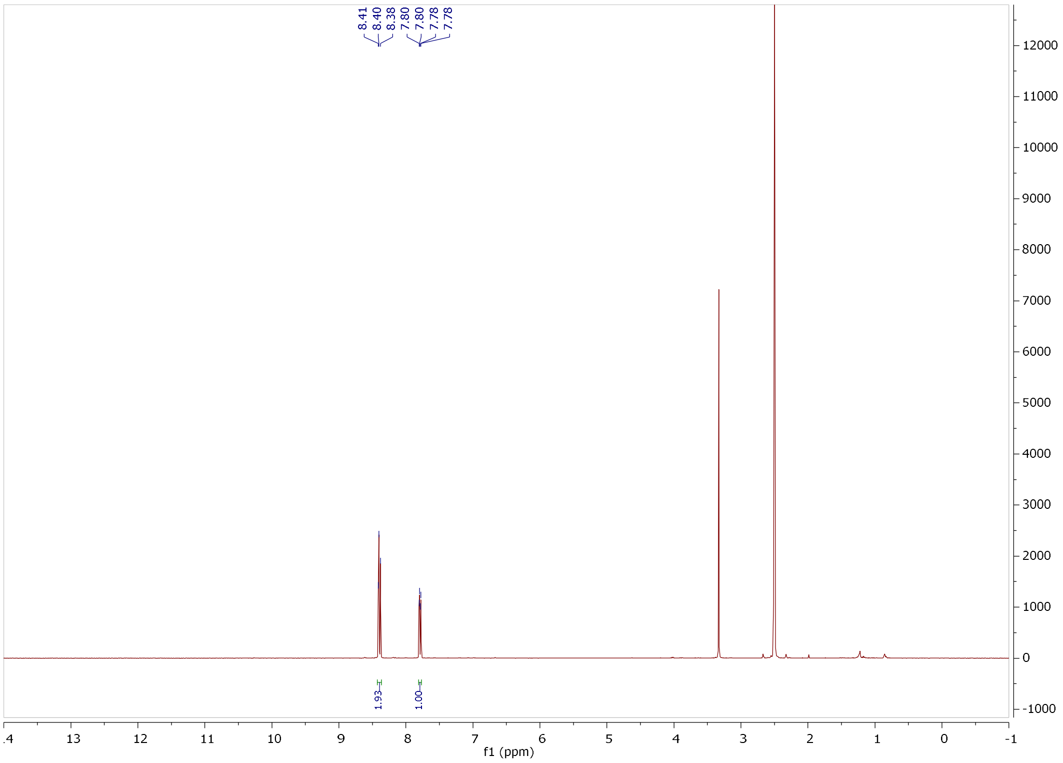
8.41
8.40
8.38
7.80
7.80
7.78
7.78
1.93
1.00
f1 (ppm)

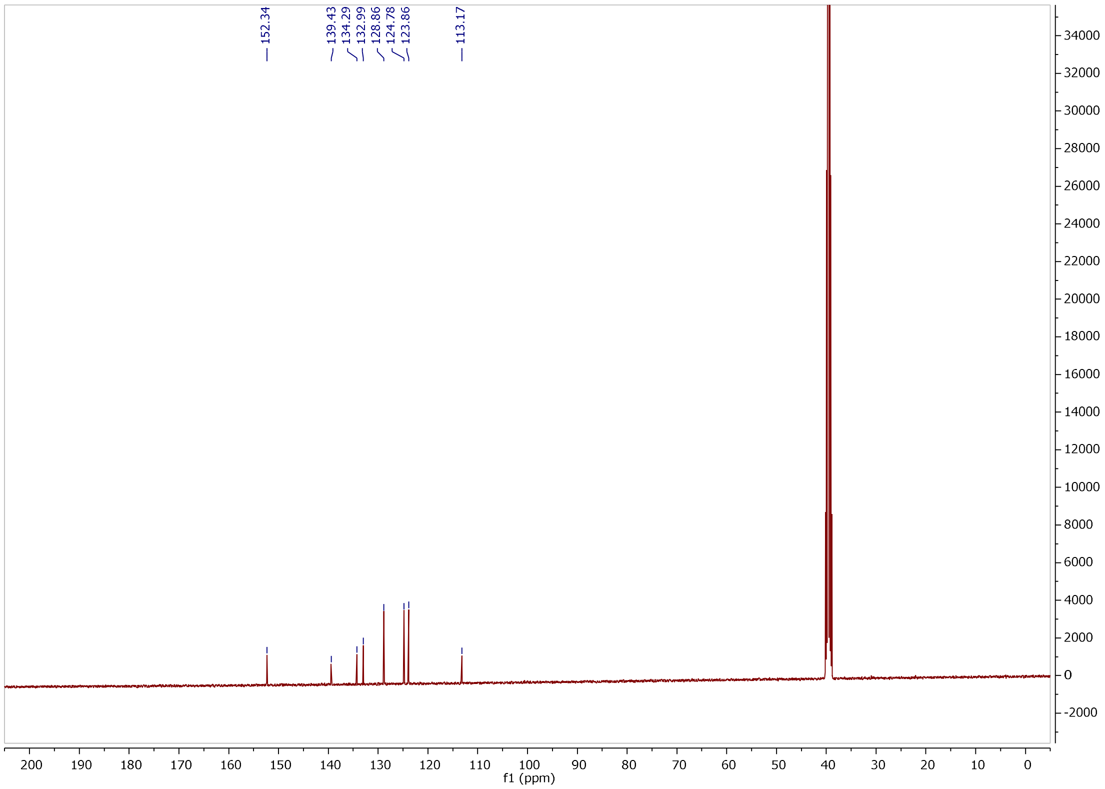
152.34
139.43
134.29
132.99
128.86
124.78
123.86
113.17
f1 (ppm)

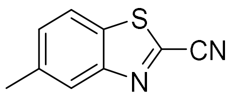
S
CN
N

Compound 64

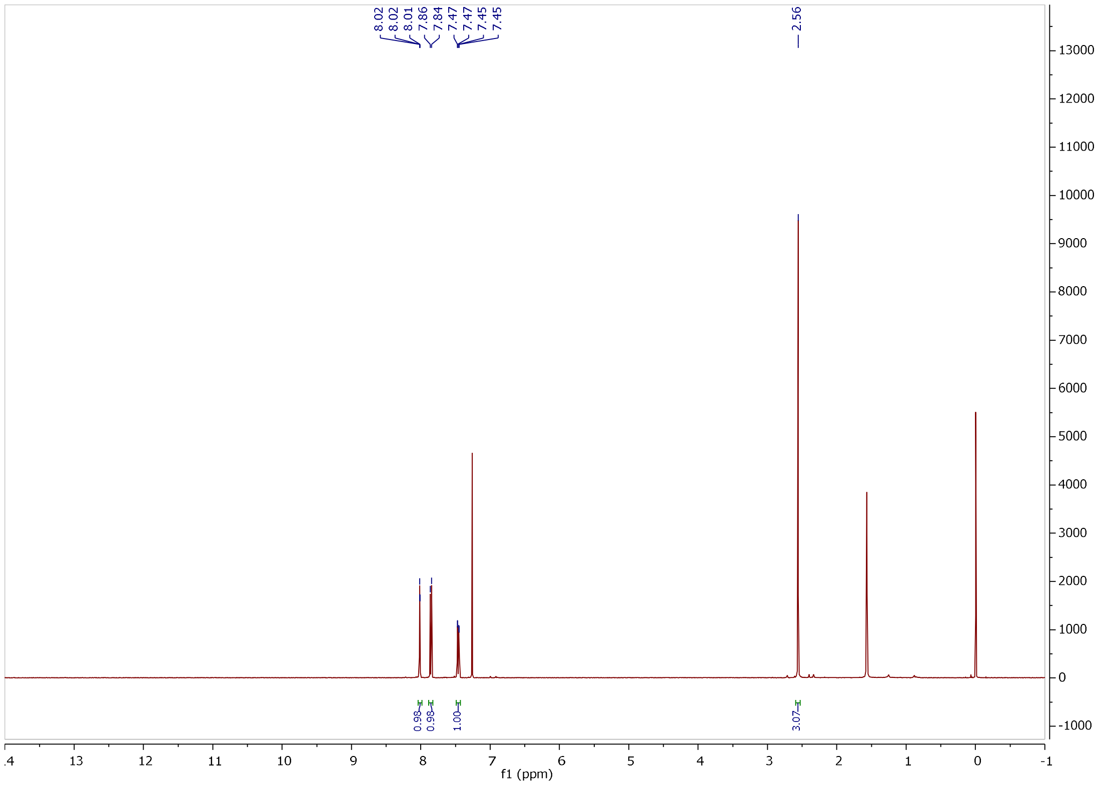

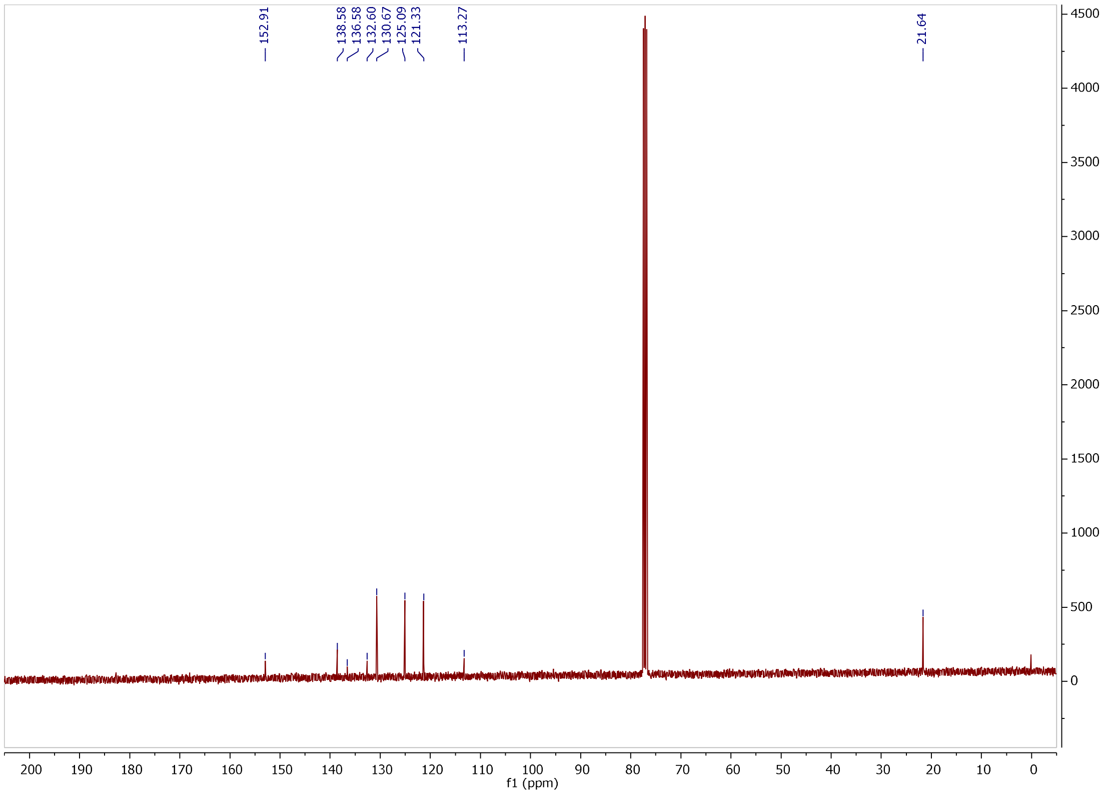

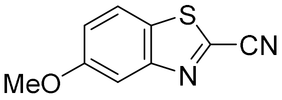

Compound 65

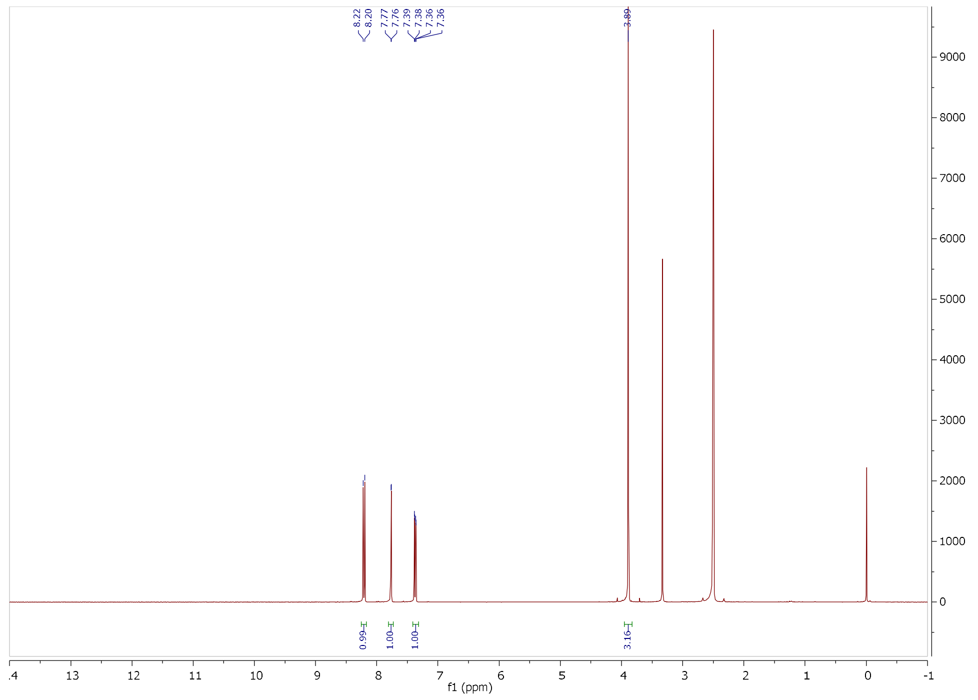
8.22
8.20
7.77
7.76
7.39
7.38
7.36
7.36
3.89
0.99
1.00
1.00
3.16
f1 (ppm)

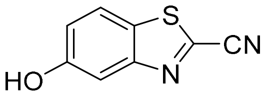

S
CN
N
HO

Compound 66

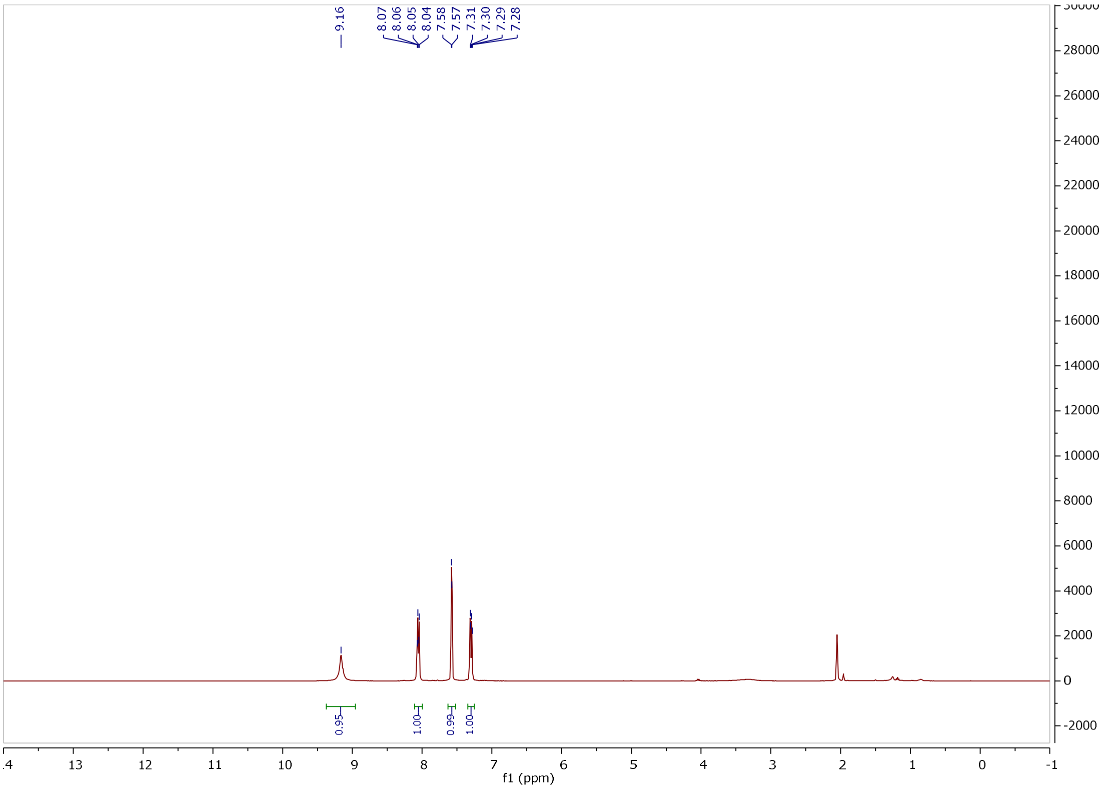

9.16
8.07
8.06
8.05
8.04
7.58
7.57
7.31
7.30
7.29
7.28
0.95
1.00
0.99
1.00
f1 (ppm)

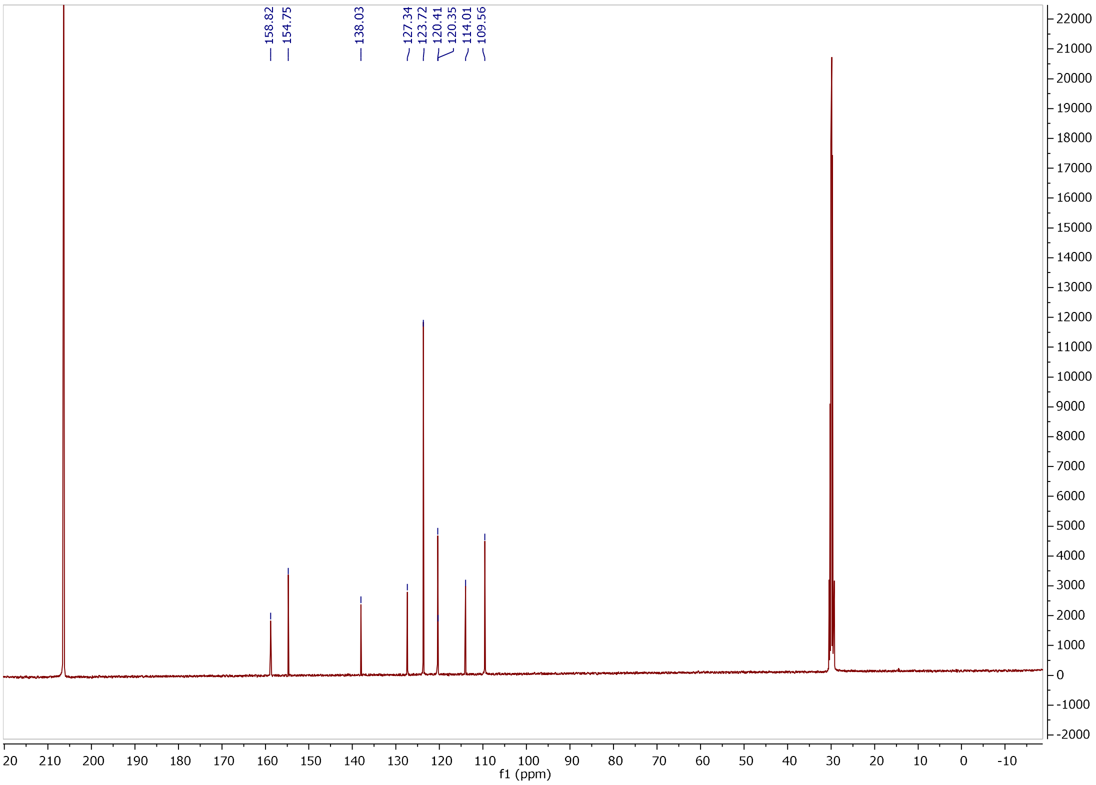

158.82
154.75
138.03
127.34
123.72
120.41
120.35
114.01
109.56
f1 (ppm)

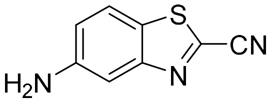

Compound 67

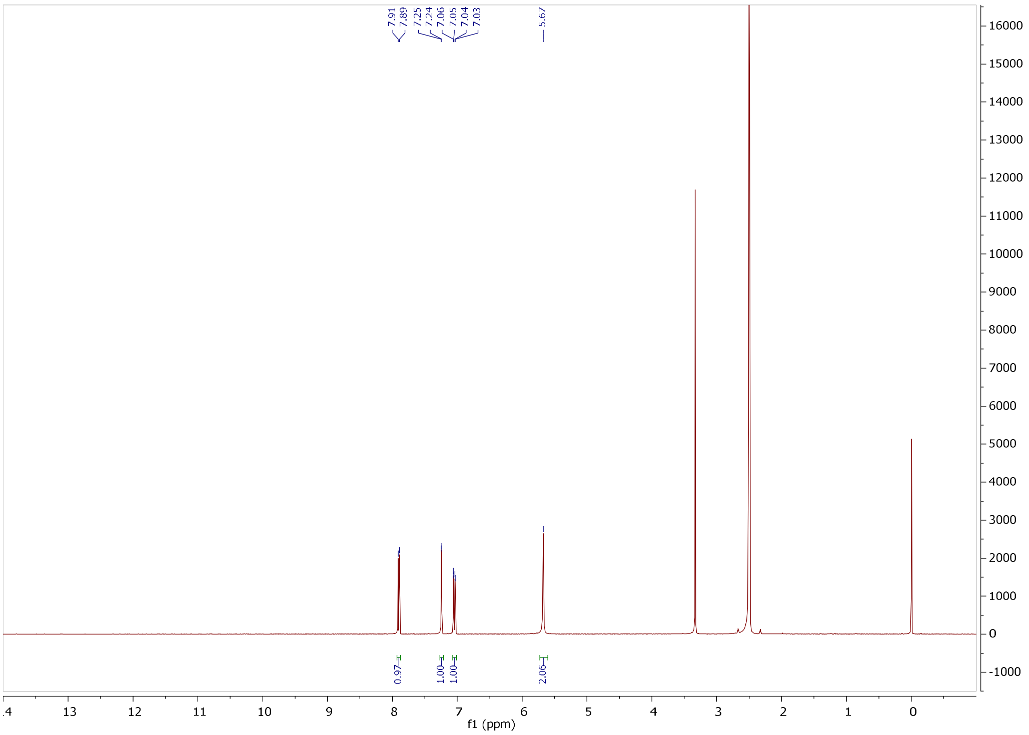

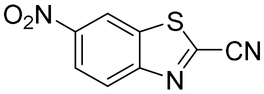

Compound 68

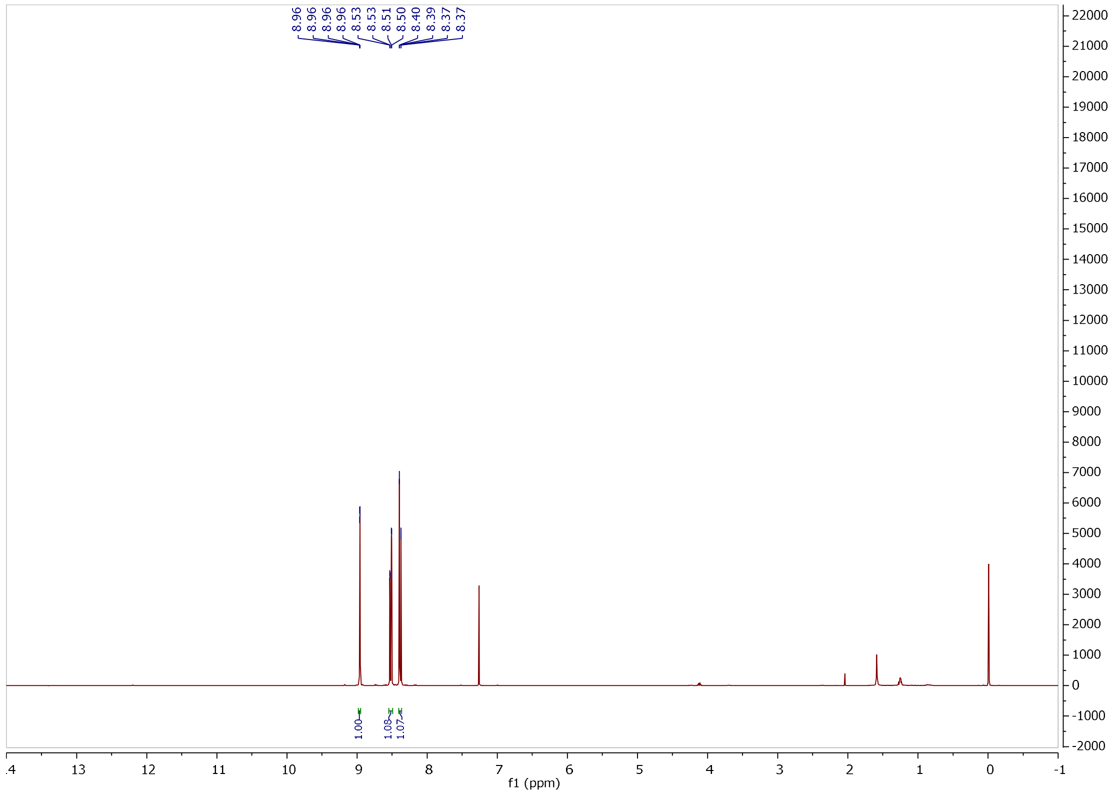

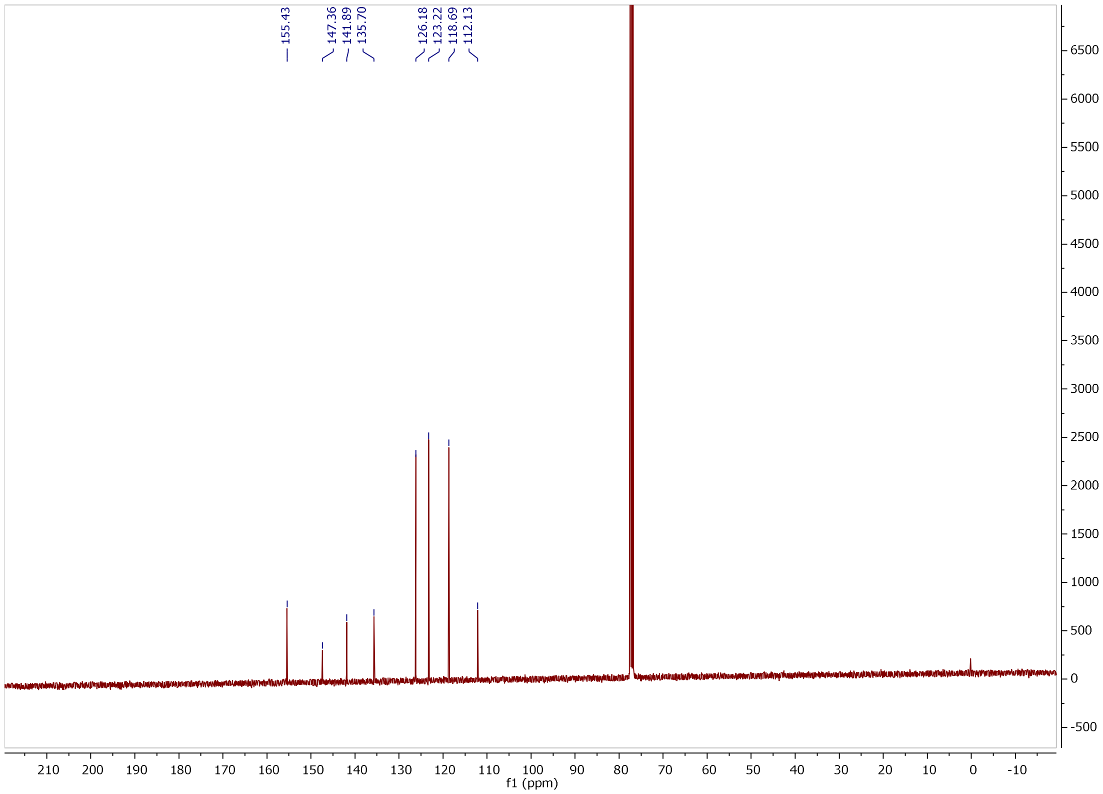

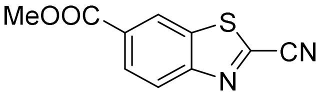

Compound 69

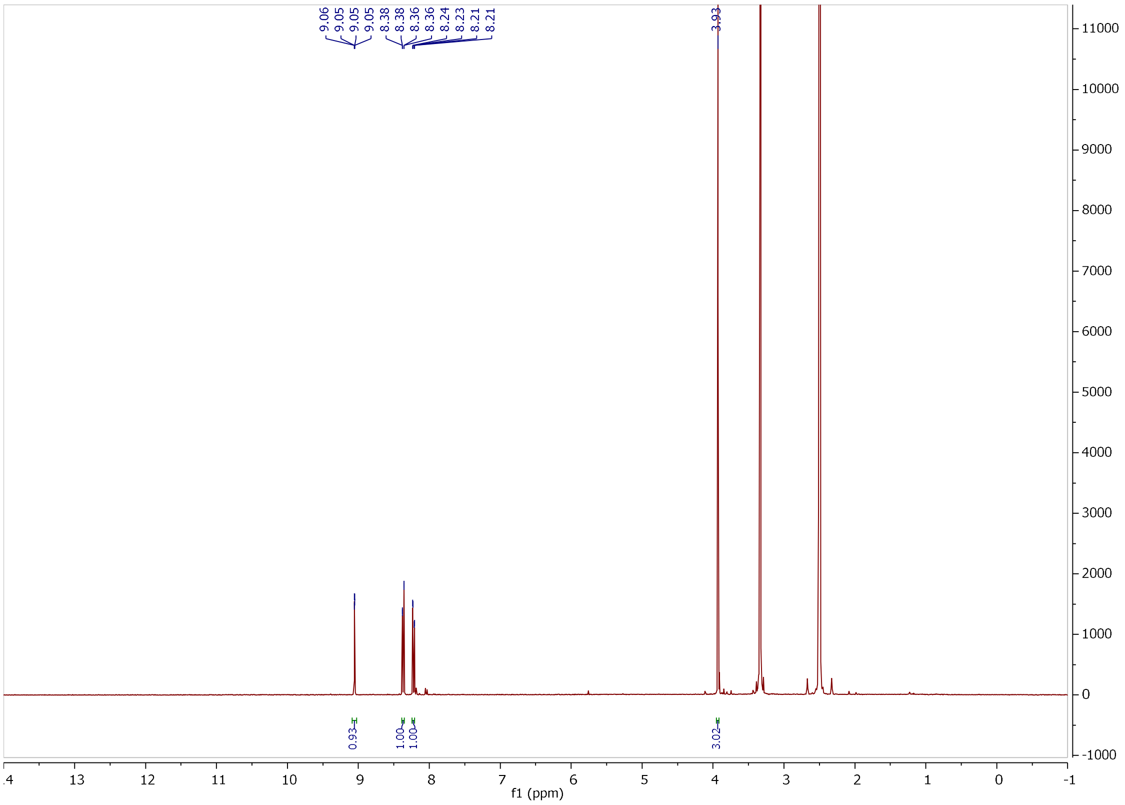
9.06
9.05
9.05
9.05
8.38
8.38
8.36
8.36
8.24
8.23
8.21
8.21
3.93
0.93
1.00
1.00
3.02
f1 (ppm)

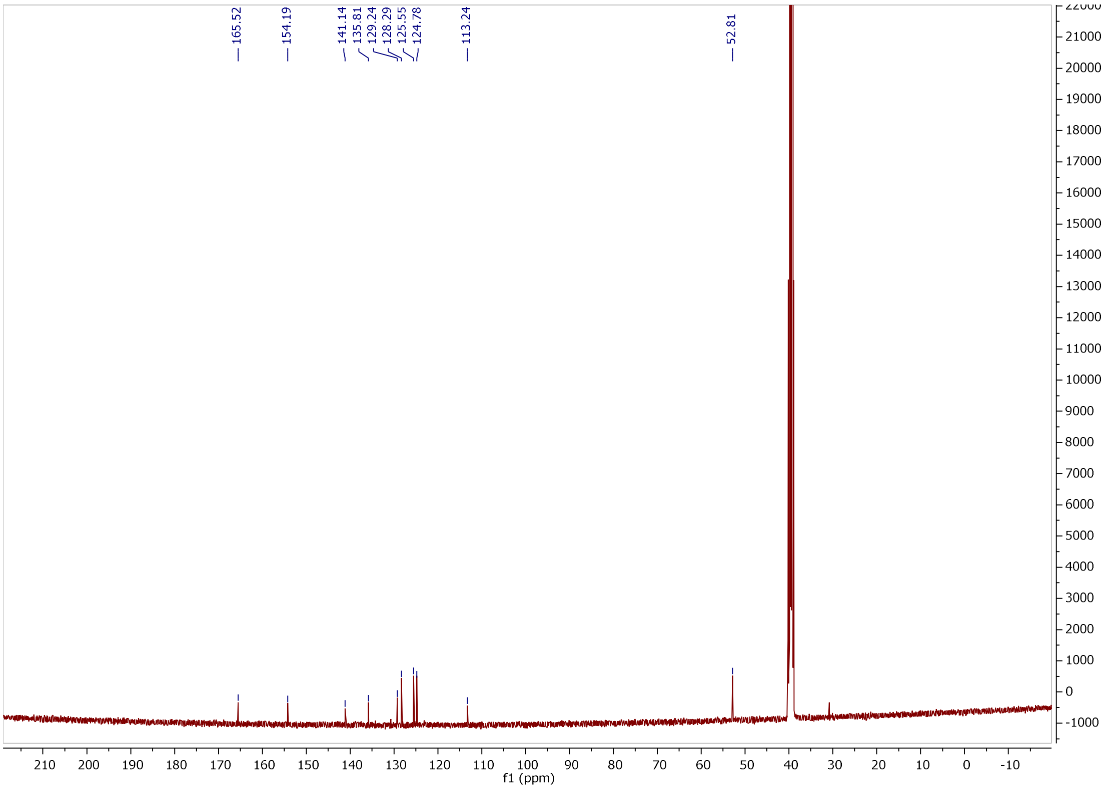
165.52
154.19
141.14
135.81
129.24
128.29
125.55
124.78
113.24
52.81
f1 (ppm)

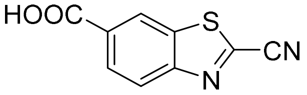
HOOC
S
CN
N

Compound 70

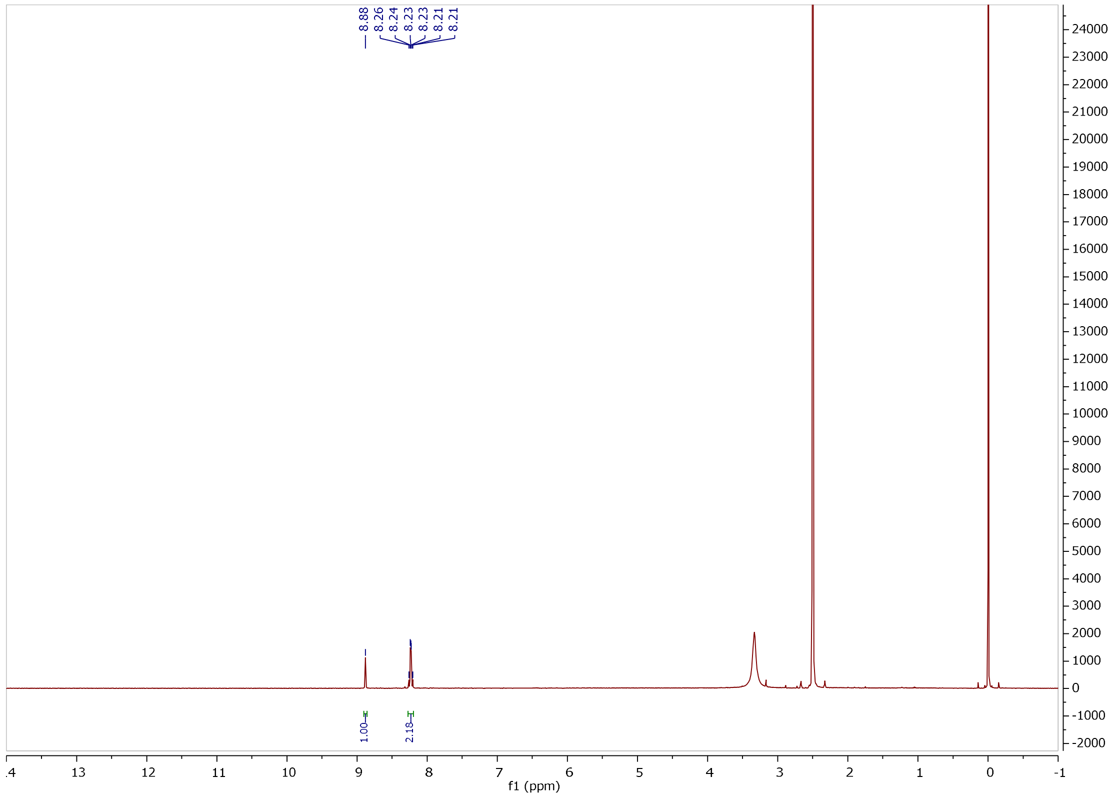
8.88
8.26
8.24
8.23
8.23
8.21
8.21
1.00
2.18
f1 (ppm)

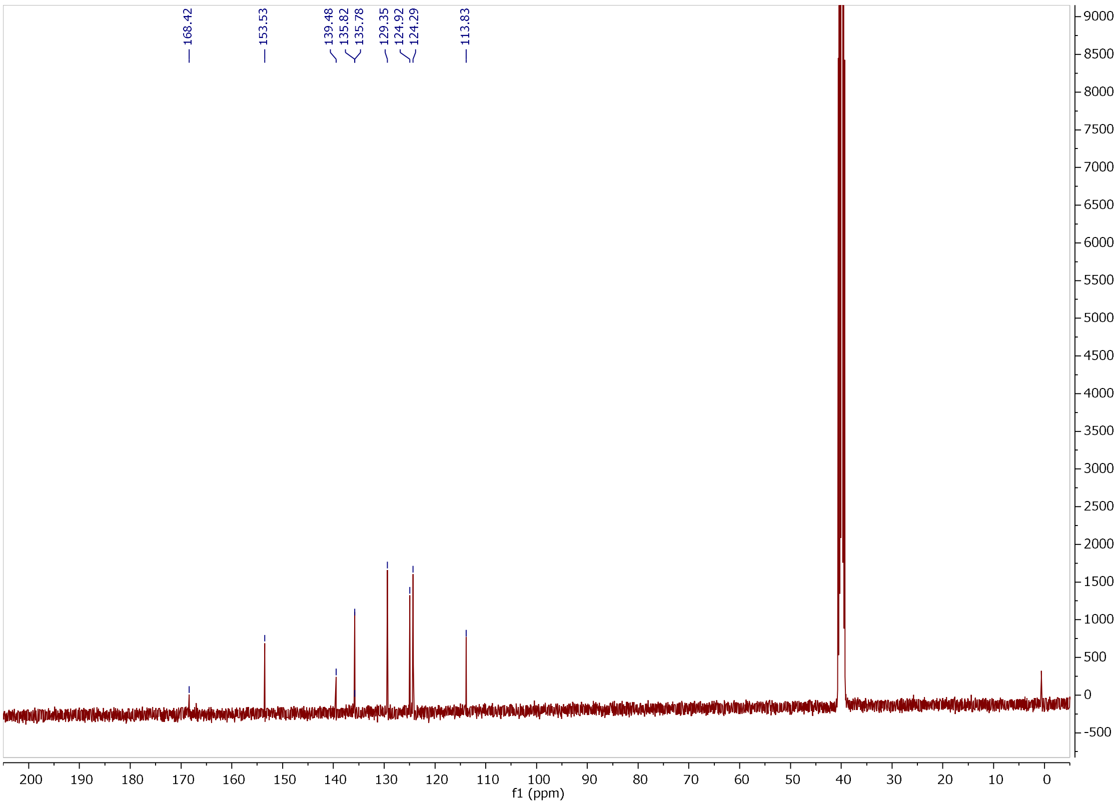
168.42
153.53
139.48
135.82
135.78
129.35
124.92
124.29
113.83
f1 (ppm)

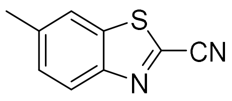

Compound 72

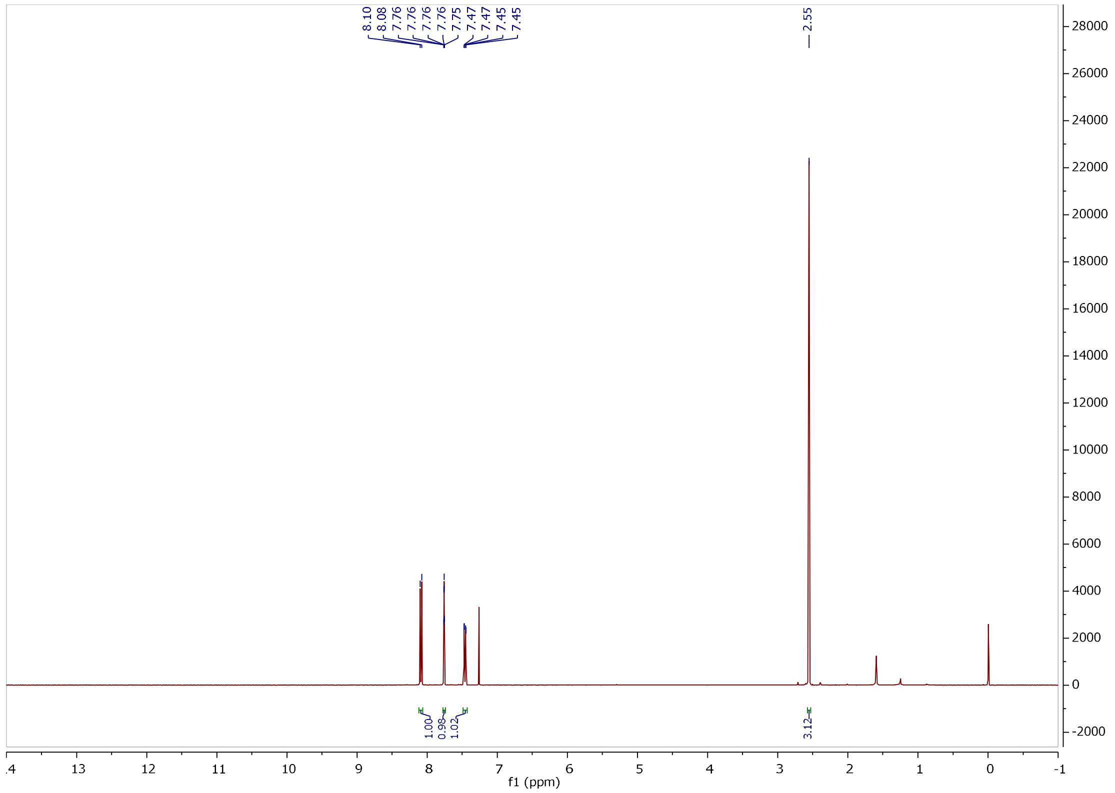
8.10
8.08
7.76
7.76
7.76
7.76
7.75
7.47
7.47
7.45
7.45
2.55
1.00
0.98
1.02
3.12
f1 (ppm)

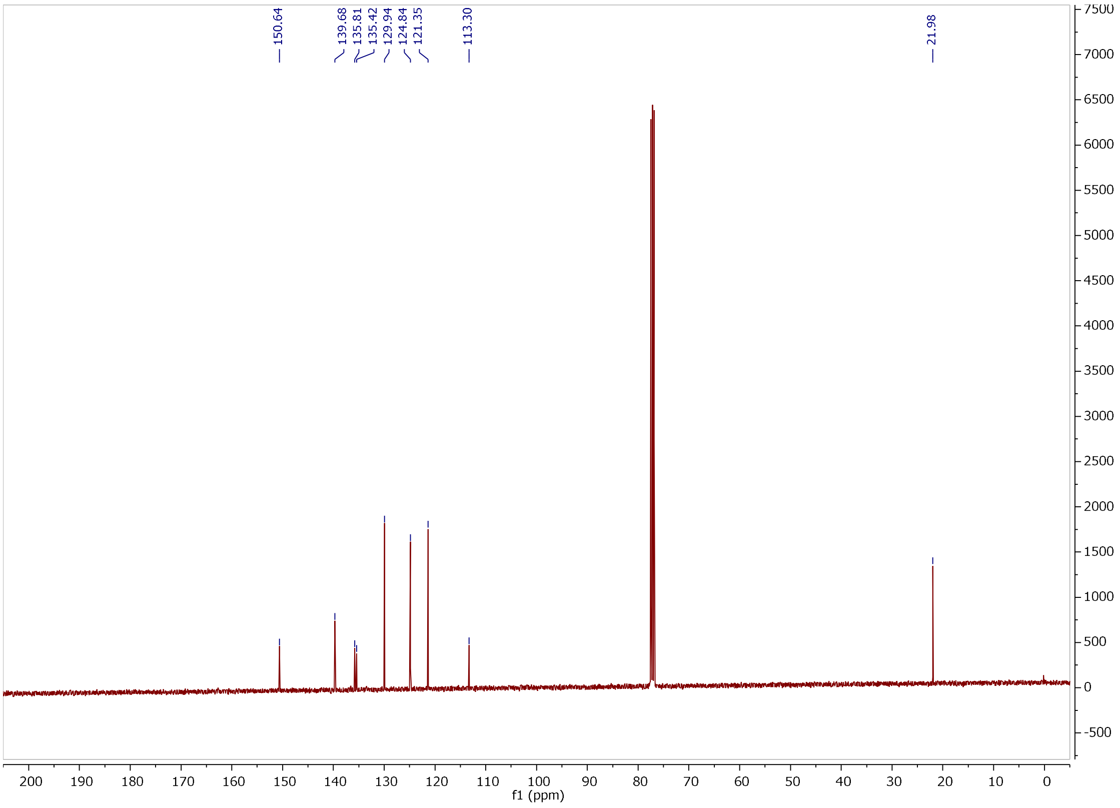
150.64
139.68
135.81
135.42
129.94
124.84
121.35
113.30
21.98
f1 (ppm)

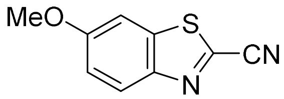

Compound 73

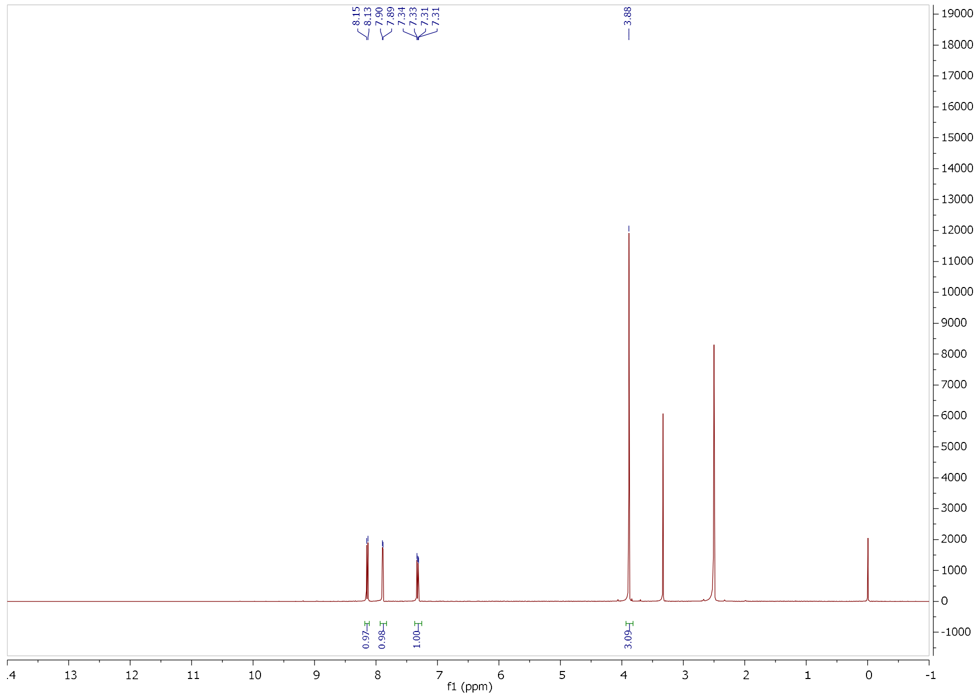

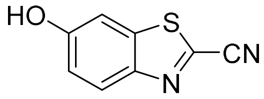

Compound 74

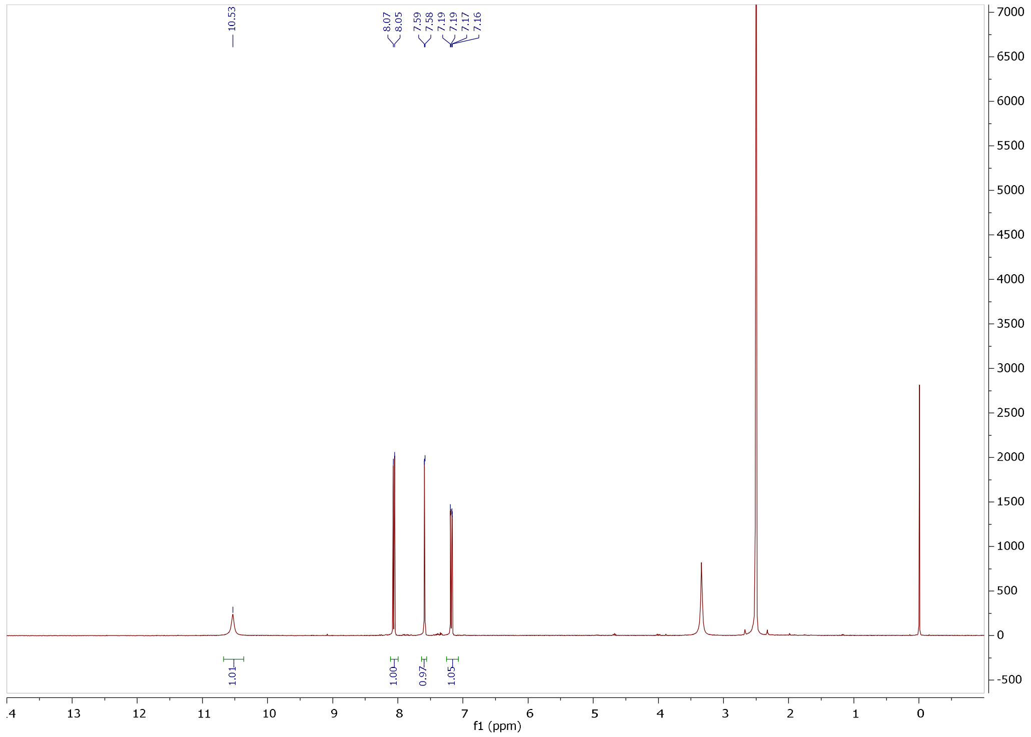

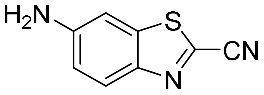
H2N
S
CN
N

Compound 75

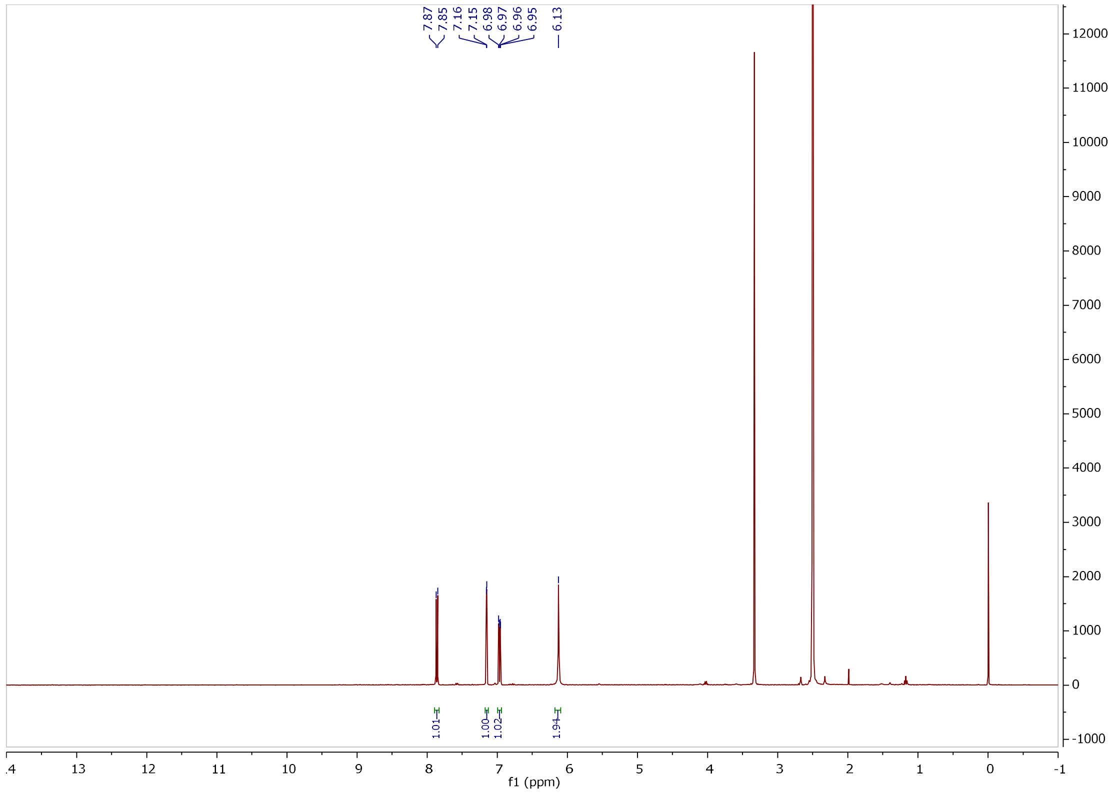
7.87
7.85
7.16
7.15
6.98
6.97
6.96
6.95
6.13
1.01
1.00
1.02
1.94
f1 (ppm)

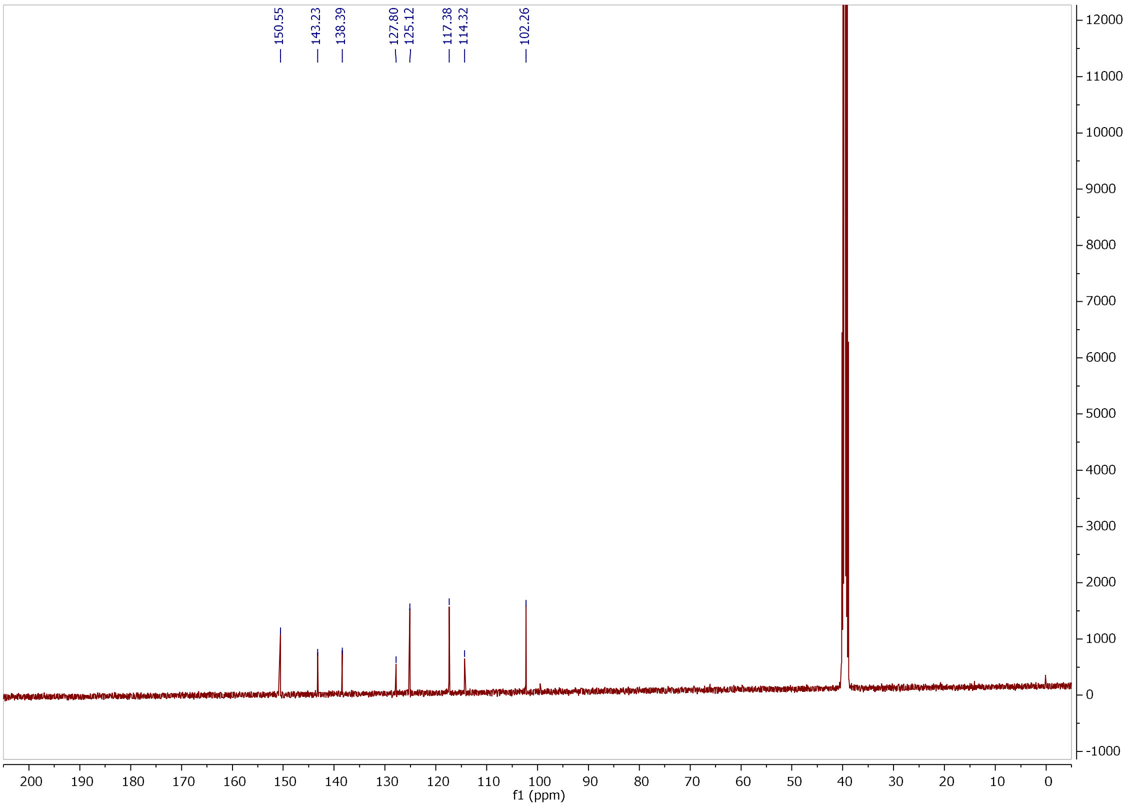
150.55
143.23
138.39
127.80
125.12
117.38
114.32
102.26
f1 (ppm)

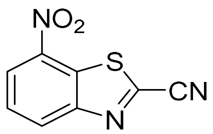

Compound 76

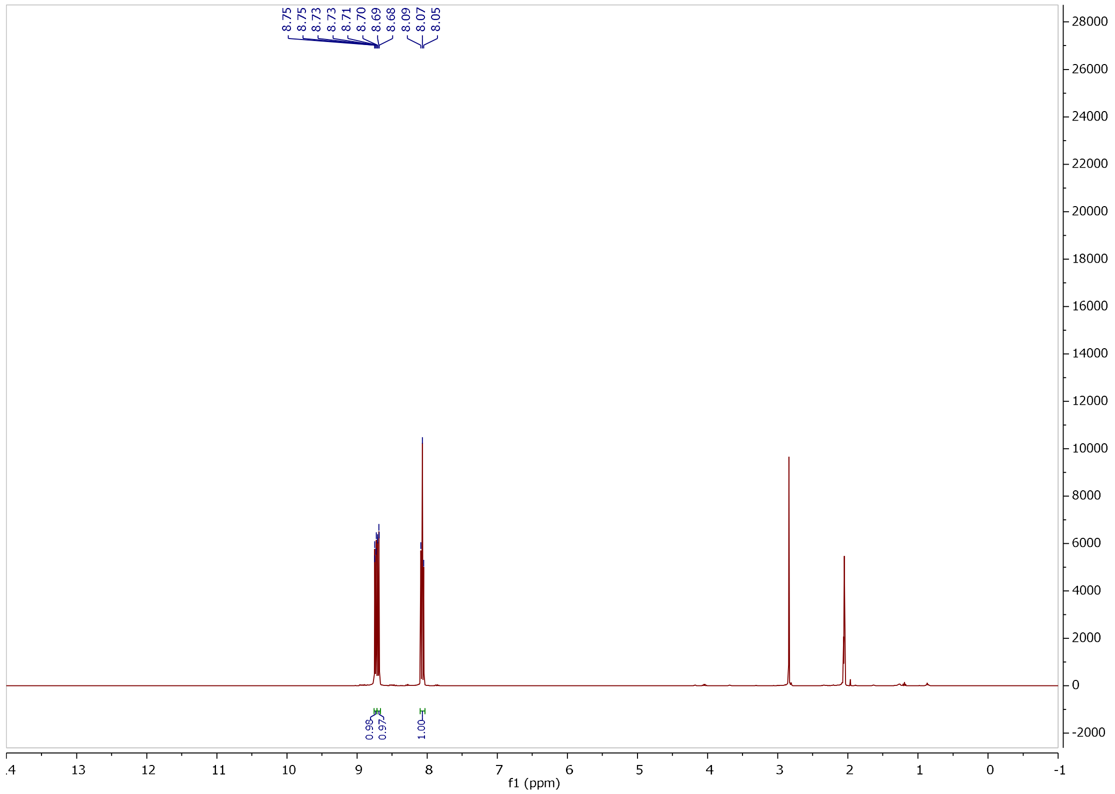
8.75
8.75
8.73
8.73
8.71
8.70
8.69
8.68
8.09
8.07
8.05
0.98
0.97
1.00
f1 (ppm)

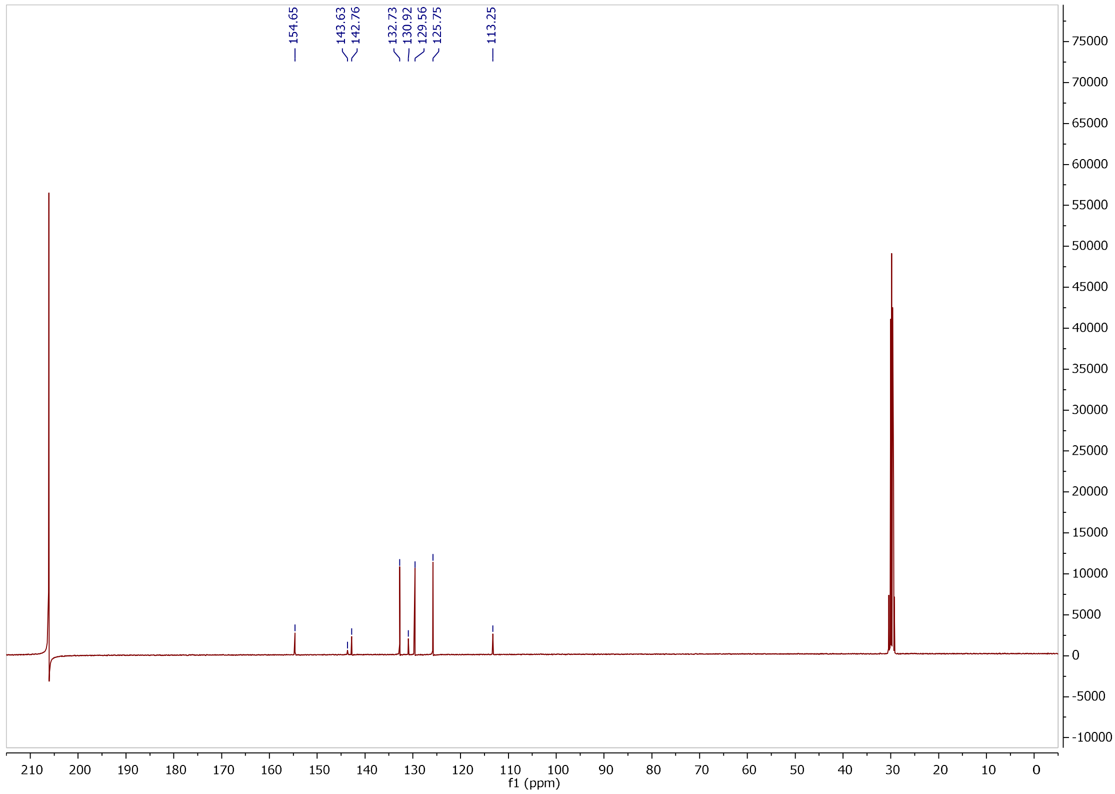
154.65
143.63
142.76
132.73
130.92
129.56
125.75
113.25
f1 (ppm)

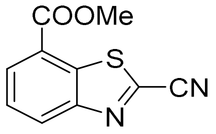
COOMe
S
CN
N

Compound 77

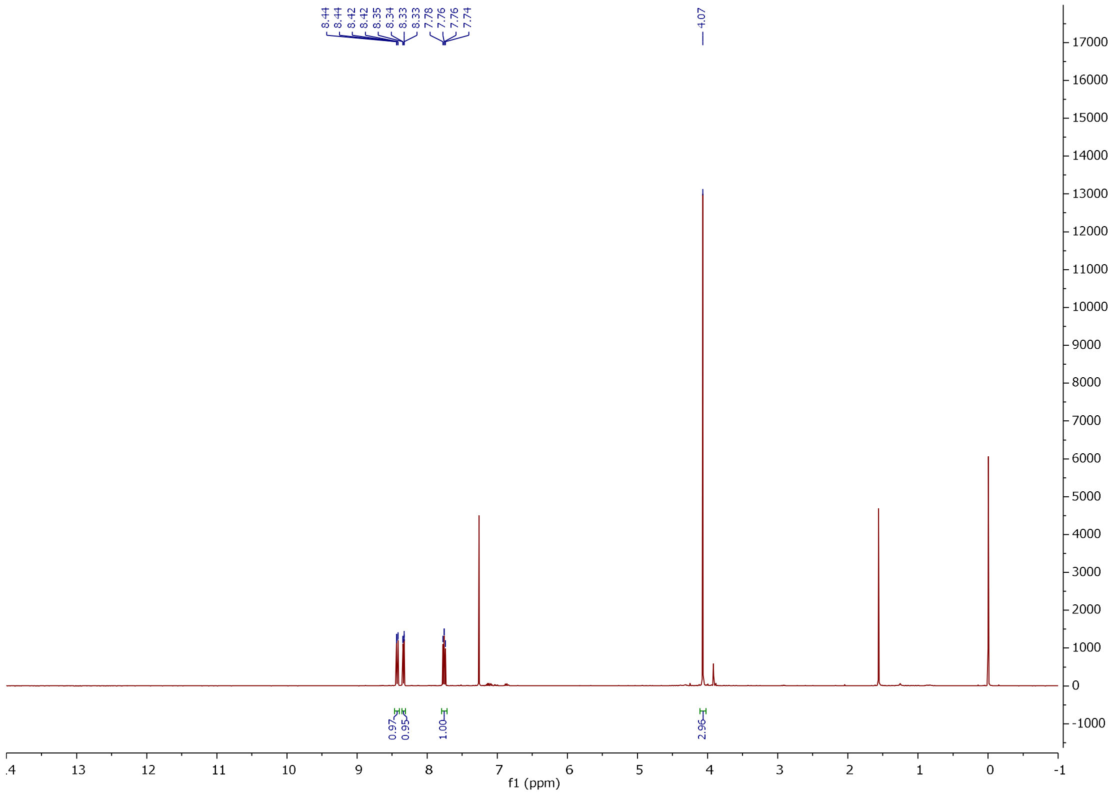
8.44
8.44
8.42
8.42
8.35
8.34
8.33
8.33
7.78
7.76
7.76
7.74
4.07
0.97
0.95
1.00
2.96
f1 (ppm)

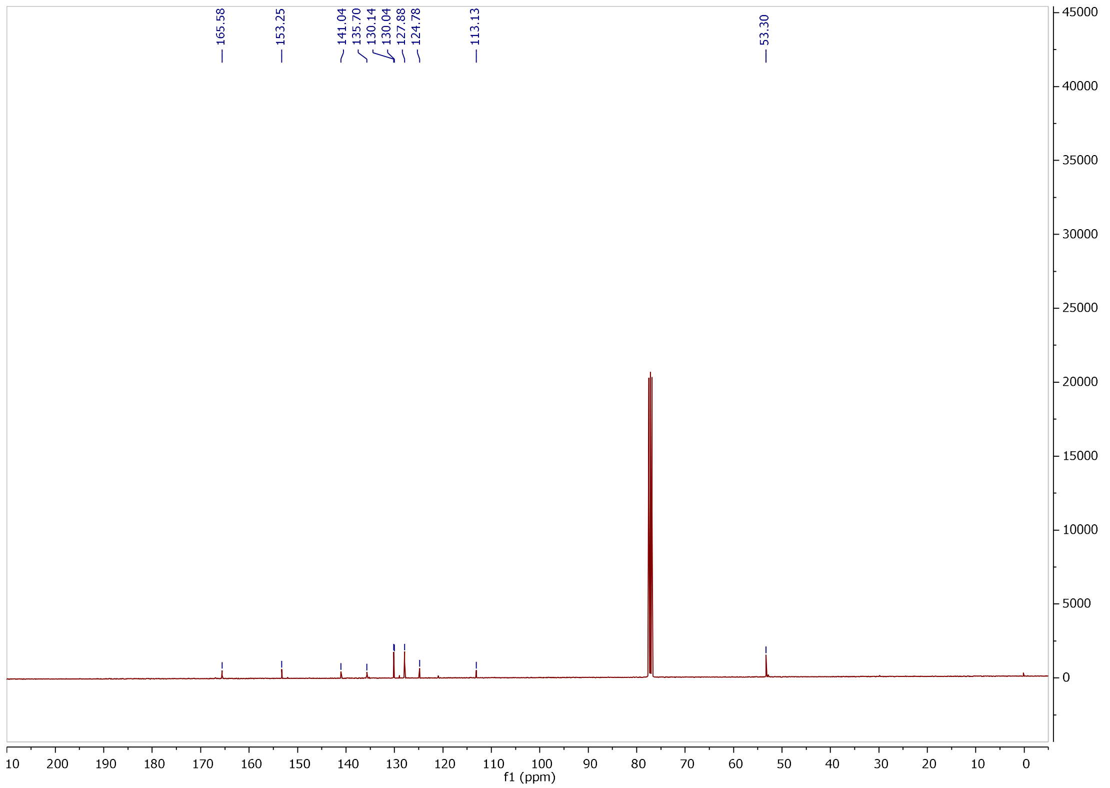
165.58
153.25
141.04
135.70
130.14
130.04
127.88
124.78
113.13
53.30
f1 (ppm)

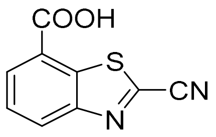

Compound 78

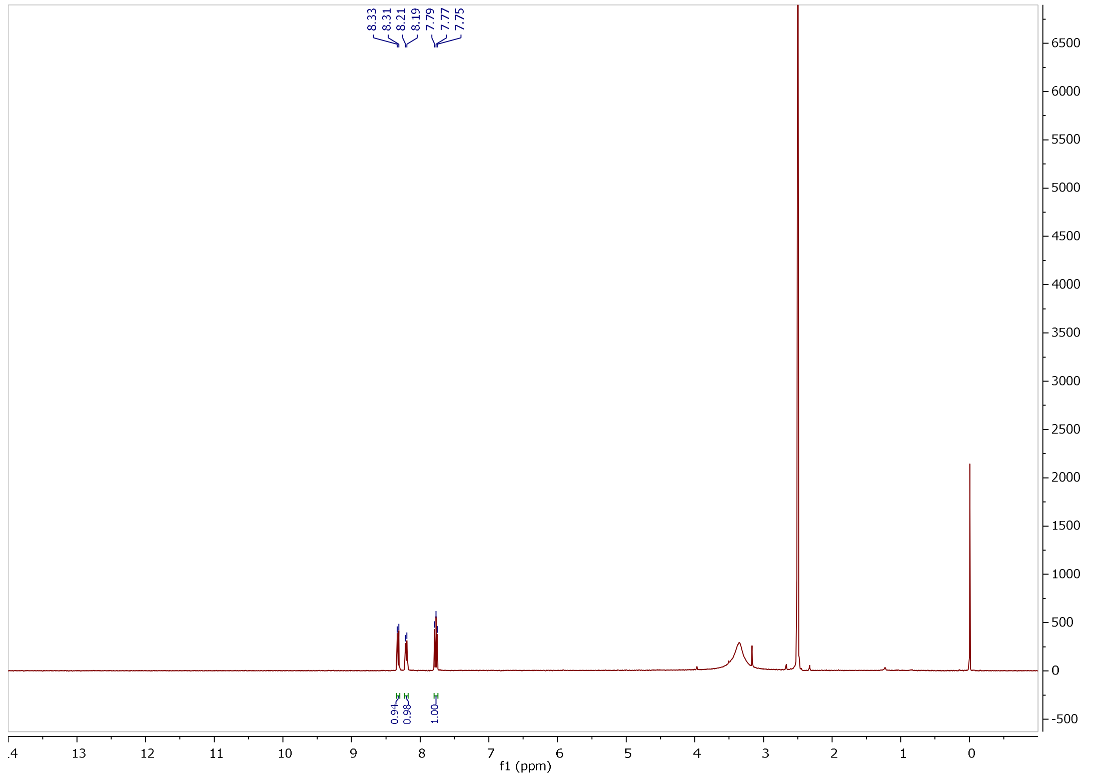
8.33
8.31
8.21
8.19
7.79
7.77
7.75
0.94
0.98
1.00
f1 (ppm)

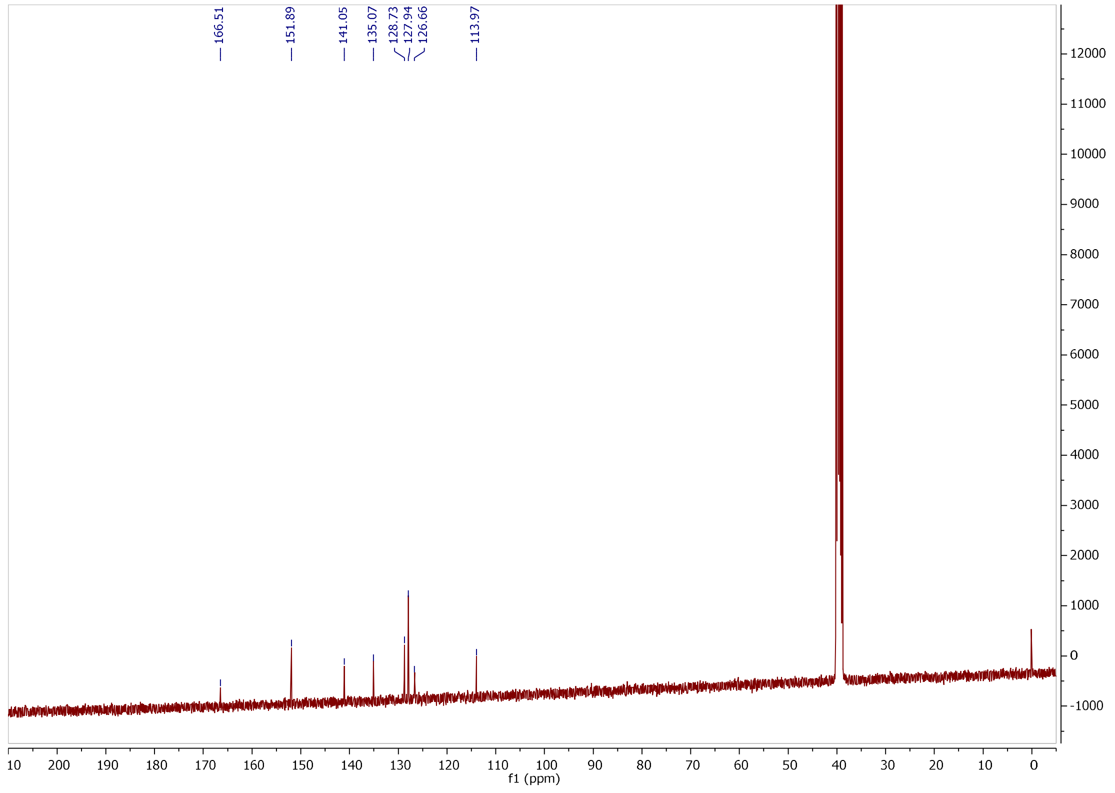
166.51
151.89
141.05
135.07
128.73
127.94
126.66
113.97
f1 (ppm)

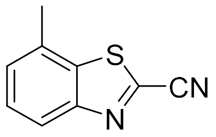
S
CN
N

Compound 80

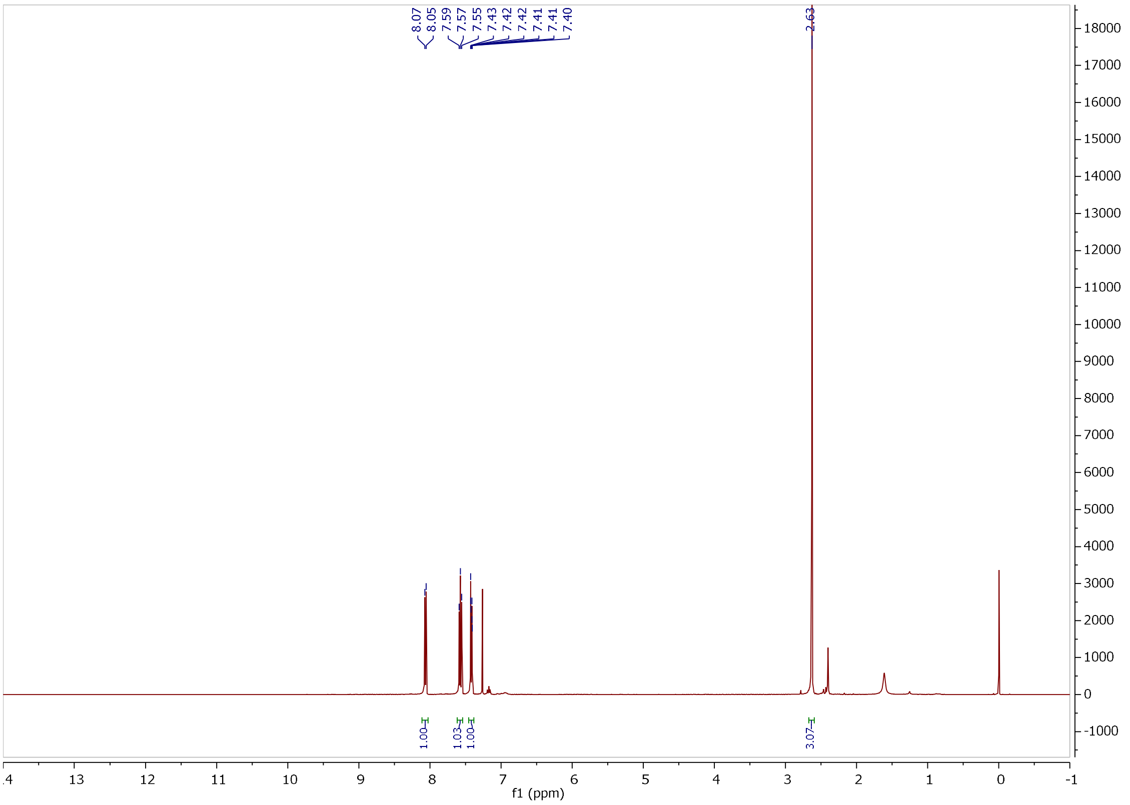
8.07
8.05
7.59
7.57
7.55
7.43
7.42
7.42
7.41
7.41
7.40
2.63
1.00
1.03
1.00
3.07
f1 (ppm)

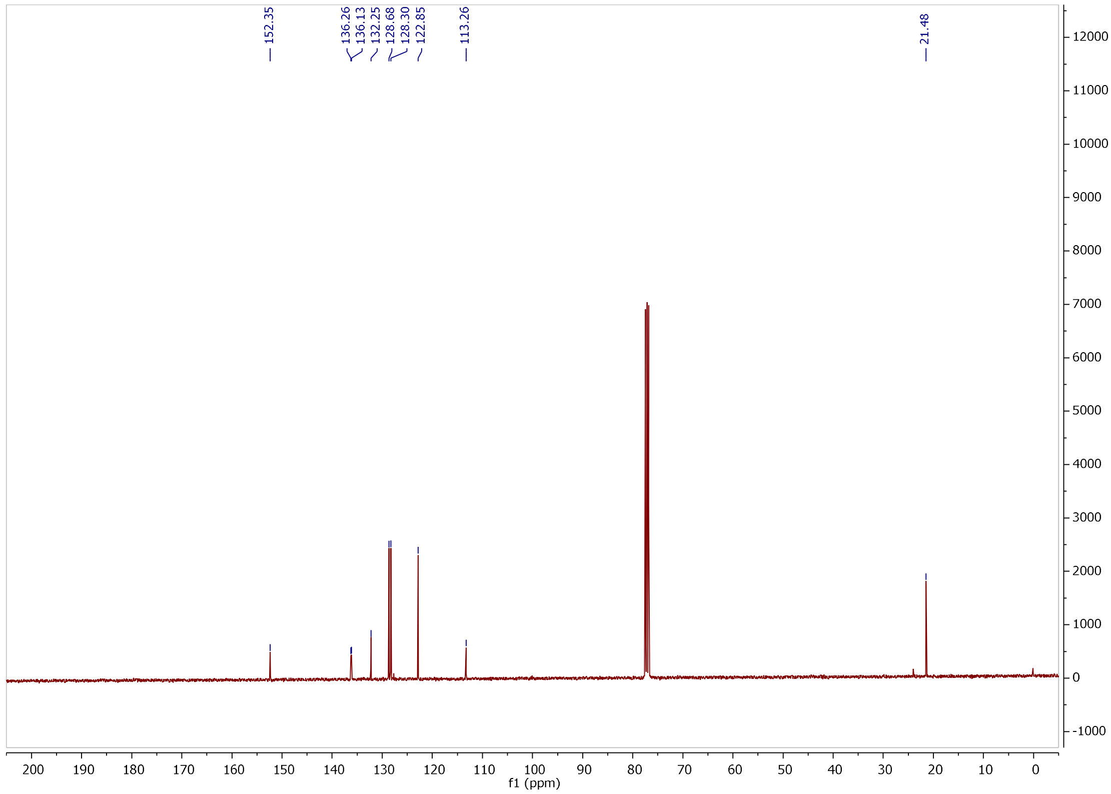
152.35
136.26
136.13
132.25
128.68
128.30
122.85
113.26
21.48
f1 (ppm)

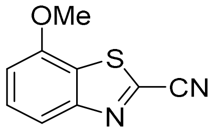
OMe
S
CN
N

Compound 81

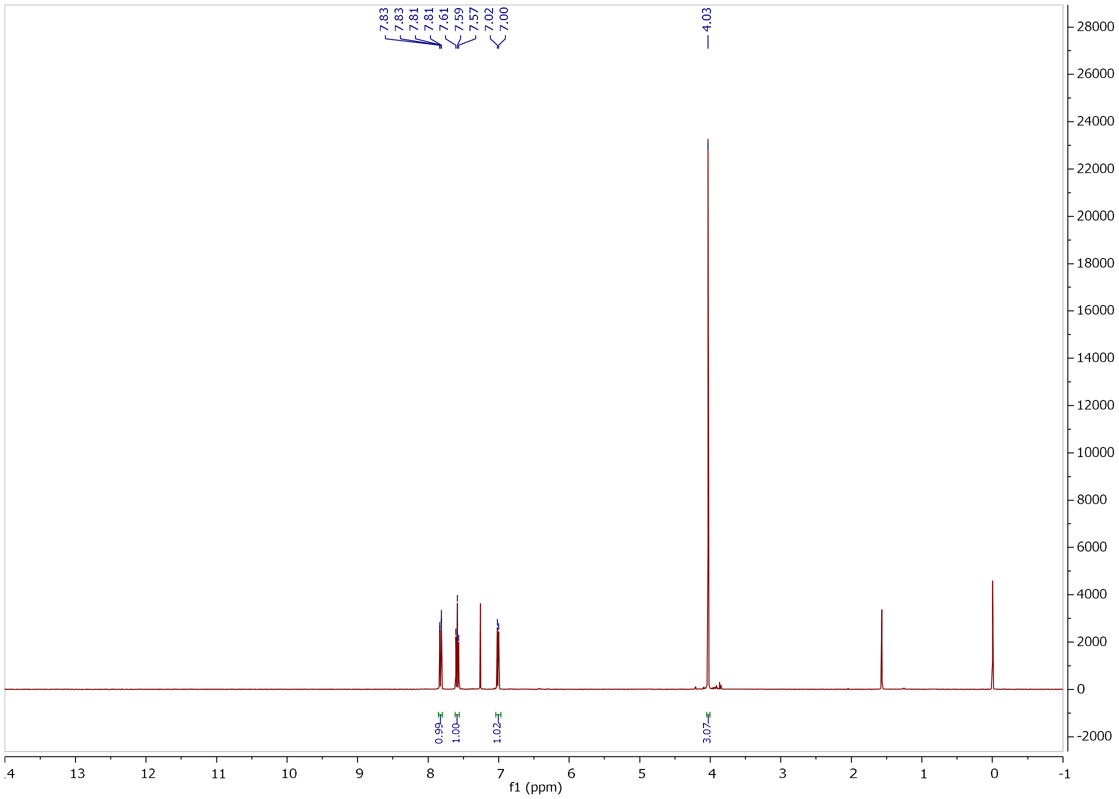
7.83
7.83
7.81
7.81
7.61
7.59
7.57
7.02
7.00
4.03
0.99
1.00
1.02
3.07
f1 (ppm)

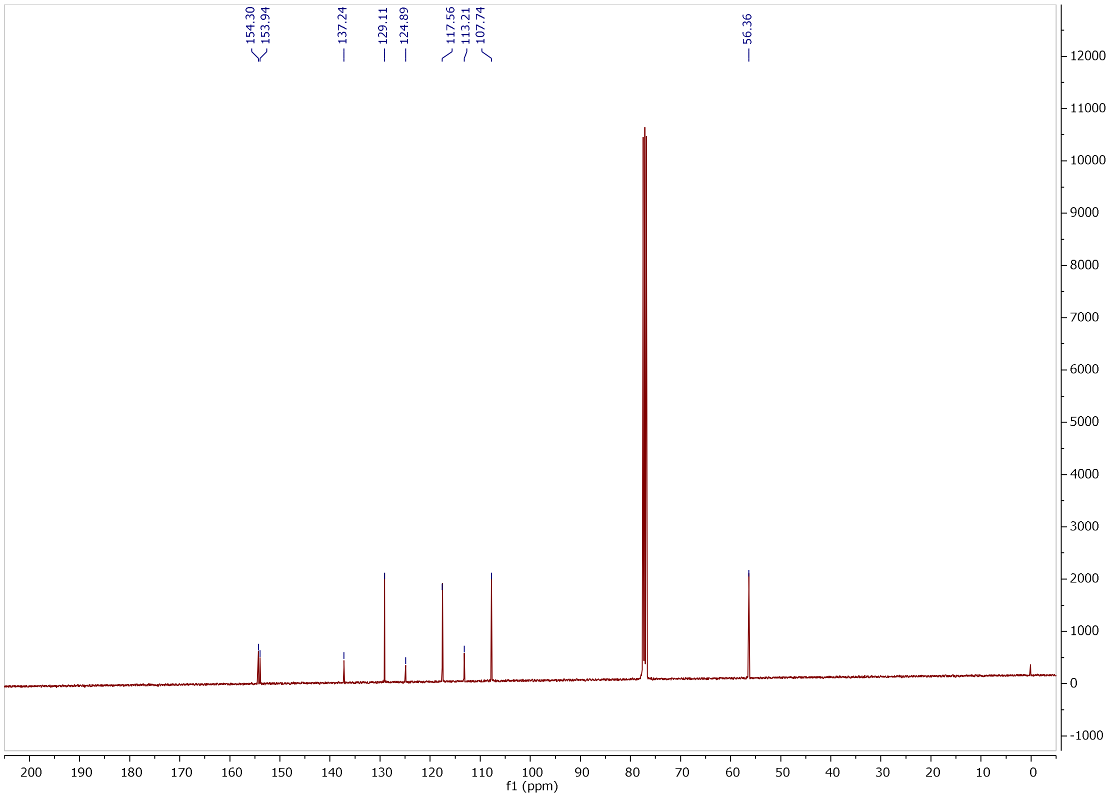
154.30
153.94
137.24
129.11
124.89
117.56
113.21
107.74
56.36
f1 (ppm)

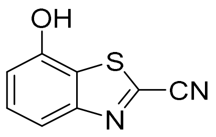
OH
S
CN
N

Compound 82

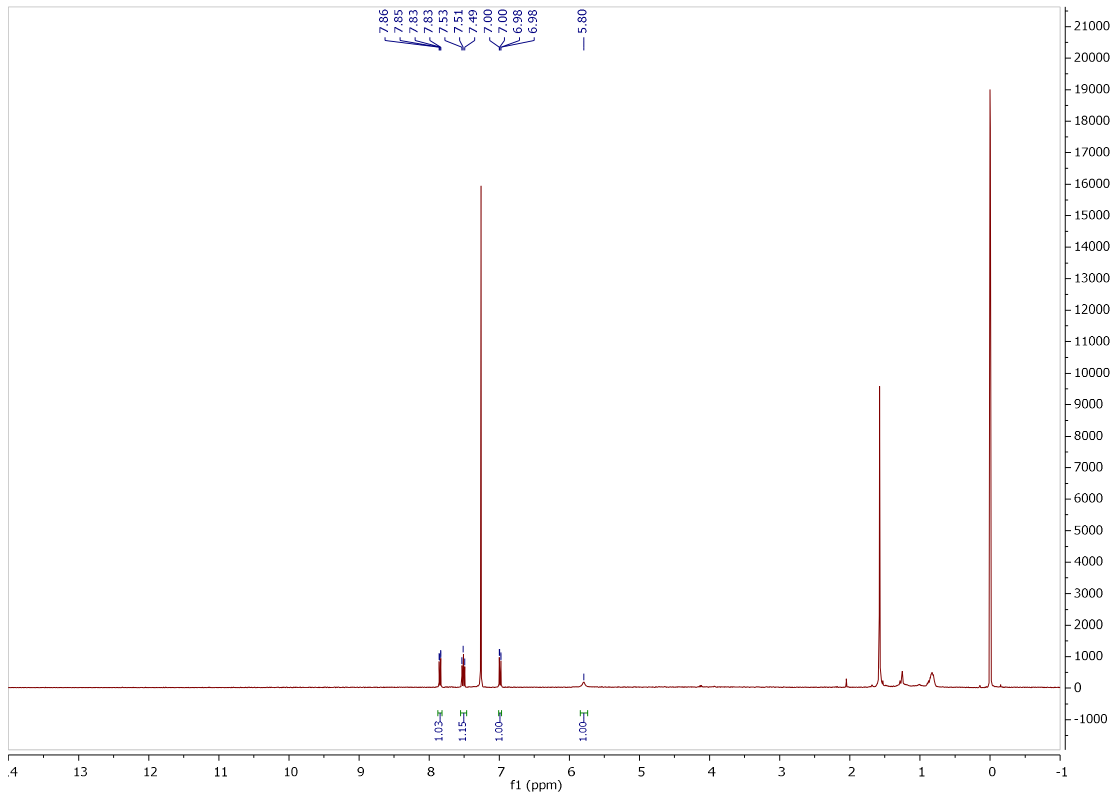
7.86
7.85
7.83
7.83
7.53
7.51
7.49
7.00
7.00
6.98
6.98
5.80
1.03
1.15
1.00
1.00
f1 (ppm)

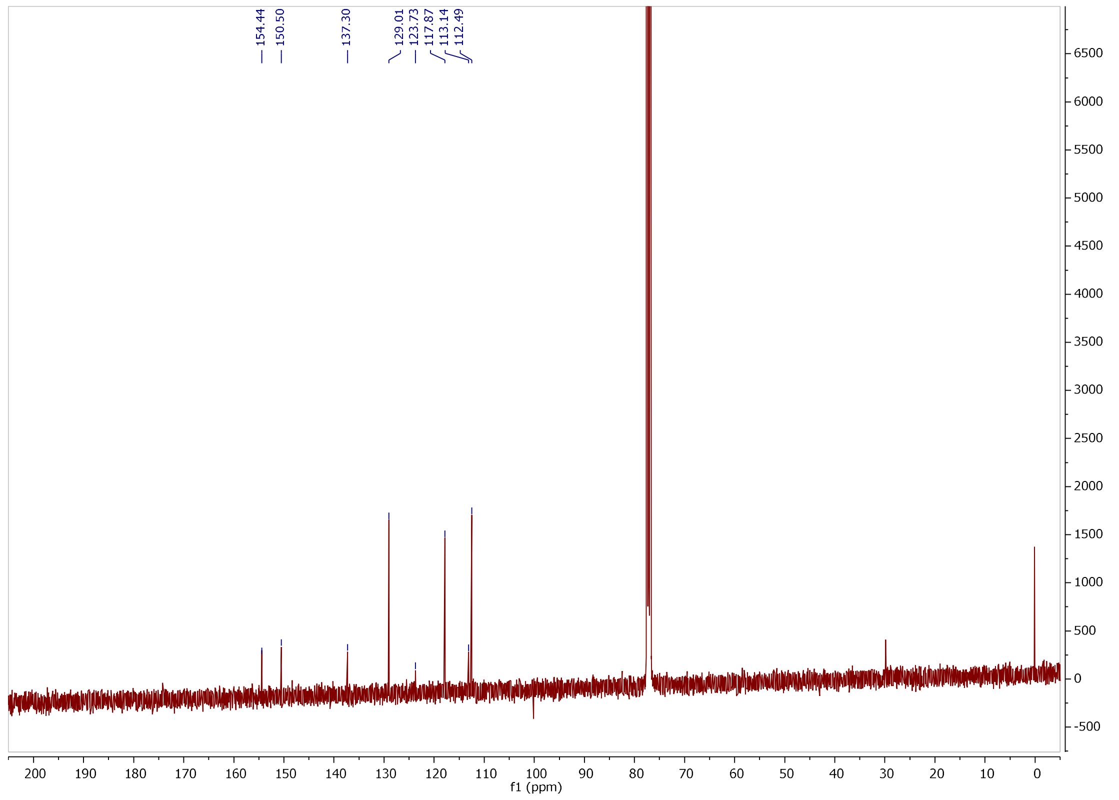
154.44
150.50
137.30
129.01
123.73
117.87
113.14
112.49
f1 (ppm)

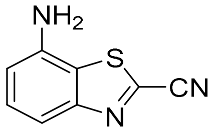
NH2
S
CN
N

Compound 83

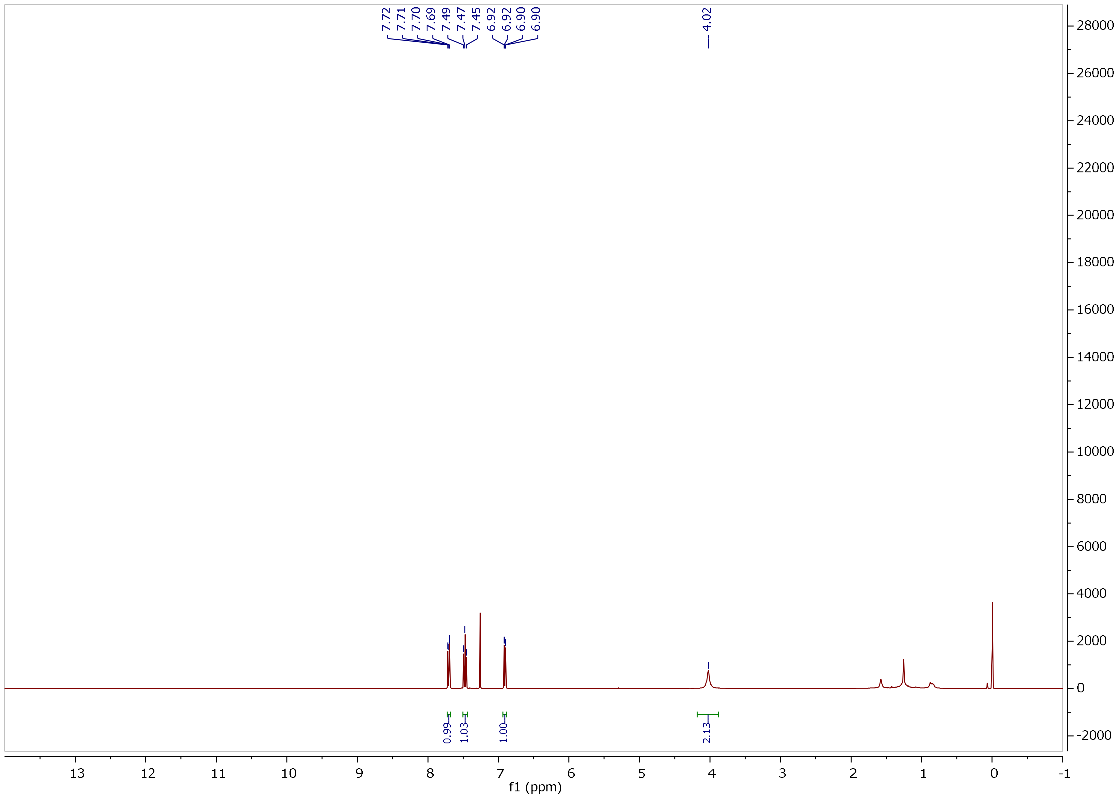
7.72
7.71
7.70
7.69
7.49
7.47
7.45
6.92
6.92
6.90
6.90
4.02
0.99
1.03
1.00
2.13
f1 (ppm)

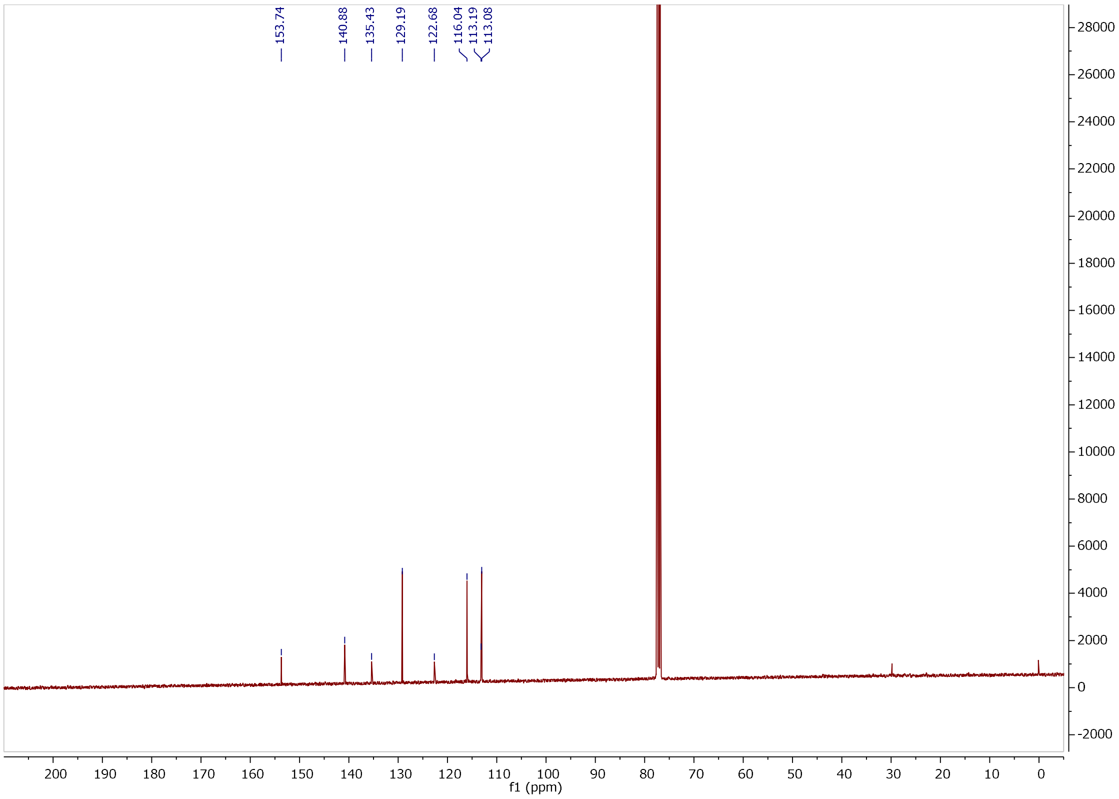
153.74
140.88
135.43
129.19
122.68
116.04
113.19
113.08
f1 (ppm)

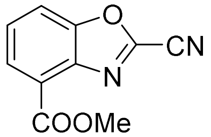

CN
N
O
COOMe

Compound 86

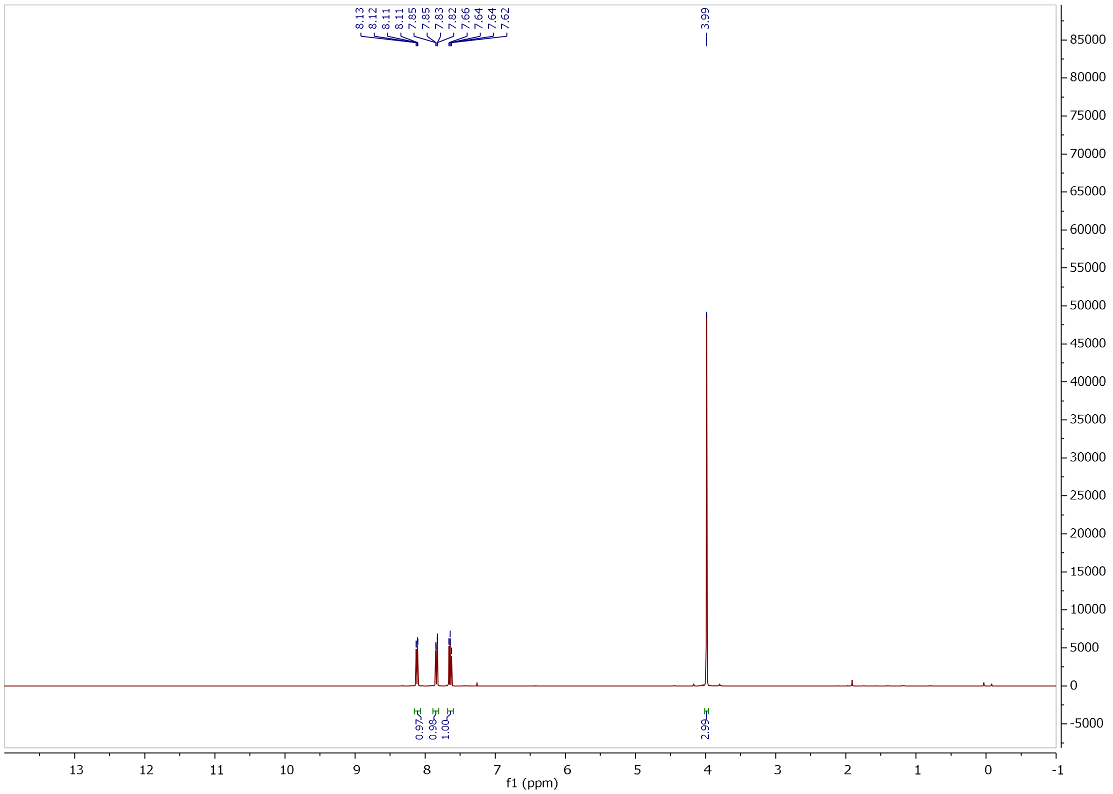

8.13
8.12
8.11
8.11
7.85
7.85
7.83
7.82
7.66
7.64
7.64
7.62
3.99
0.97
0.98
1.00
2.99
f1 (ppm)

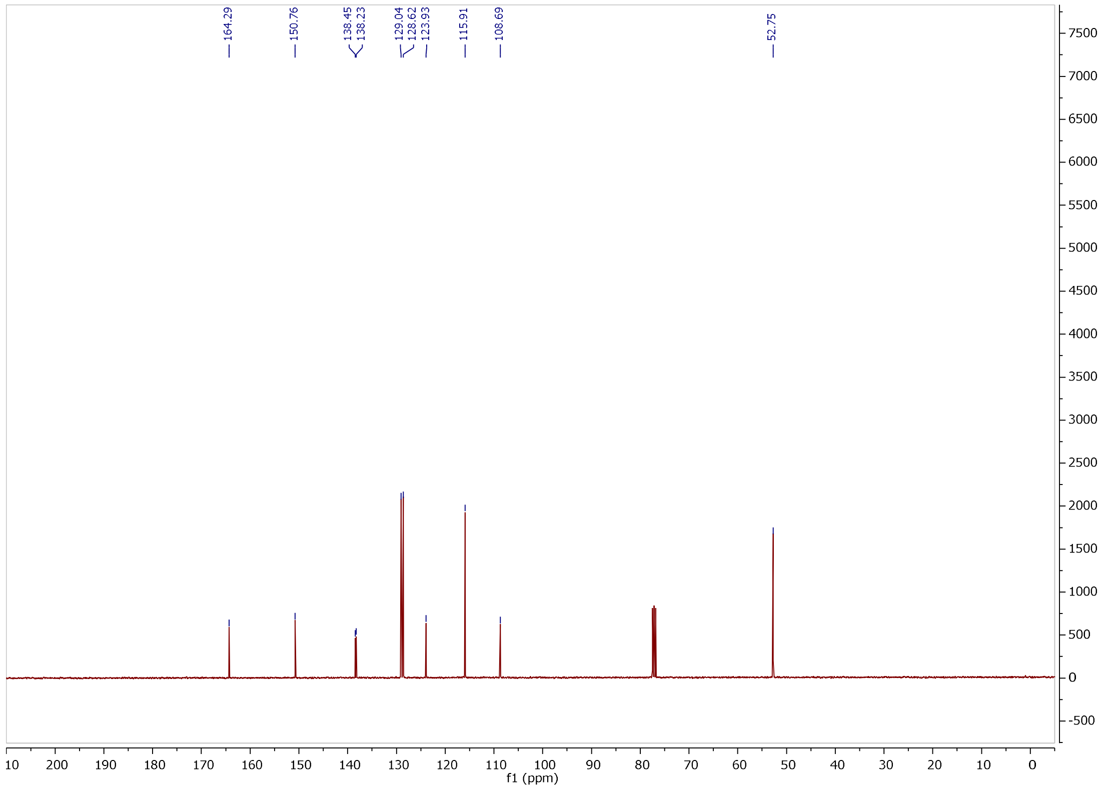

164.29
150.76
138.45
138.23
129.04
128.62
123.93
115.91
108.69
52.75
f1 (ppm)

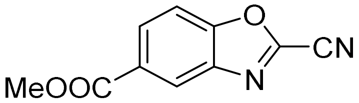
MeOOC
O
N
CN

Compound 94

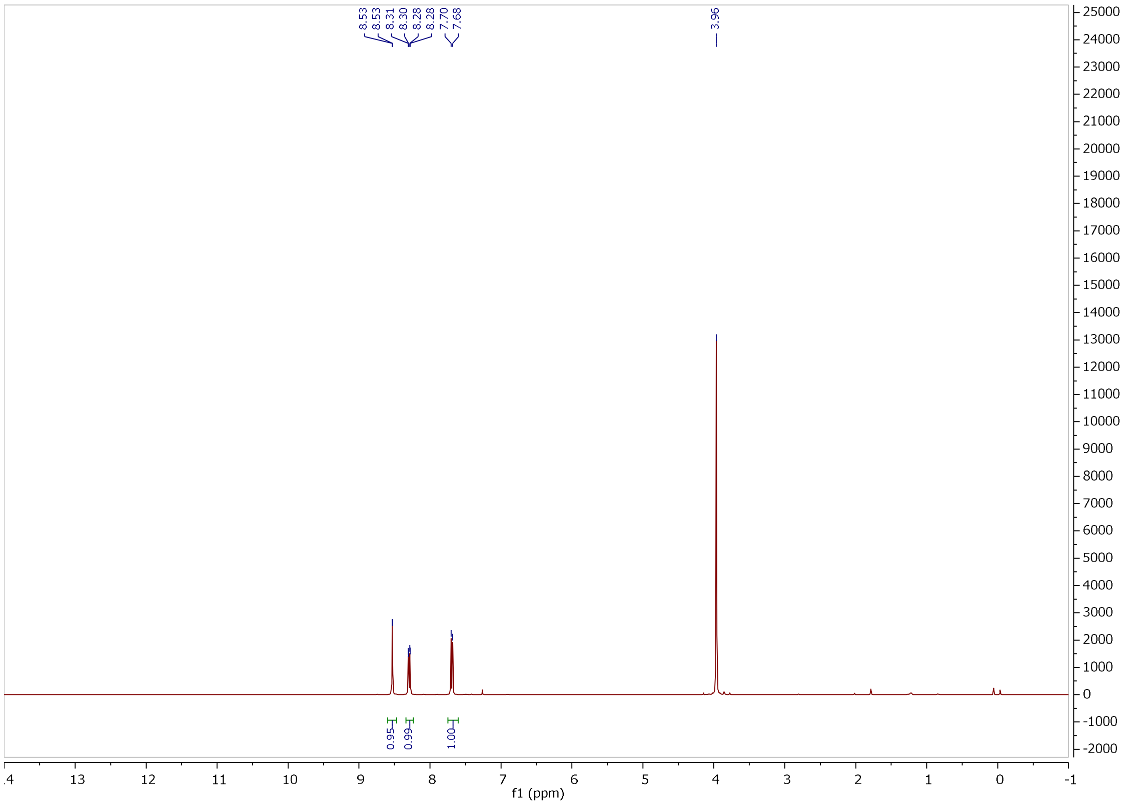
8.53
8.53
8.31
8.30
8.28
8.28
7.70
7.68
3.96
0.95
0.99
1.00
f1 (ppm)

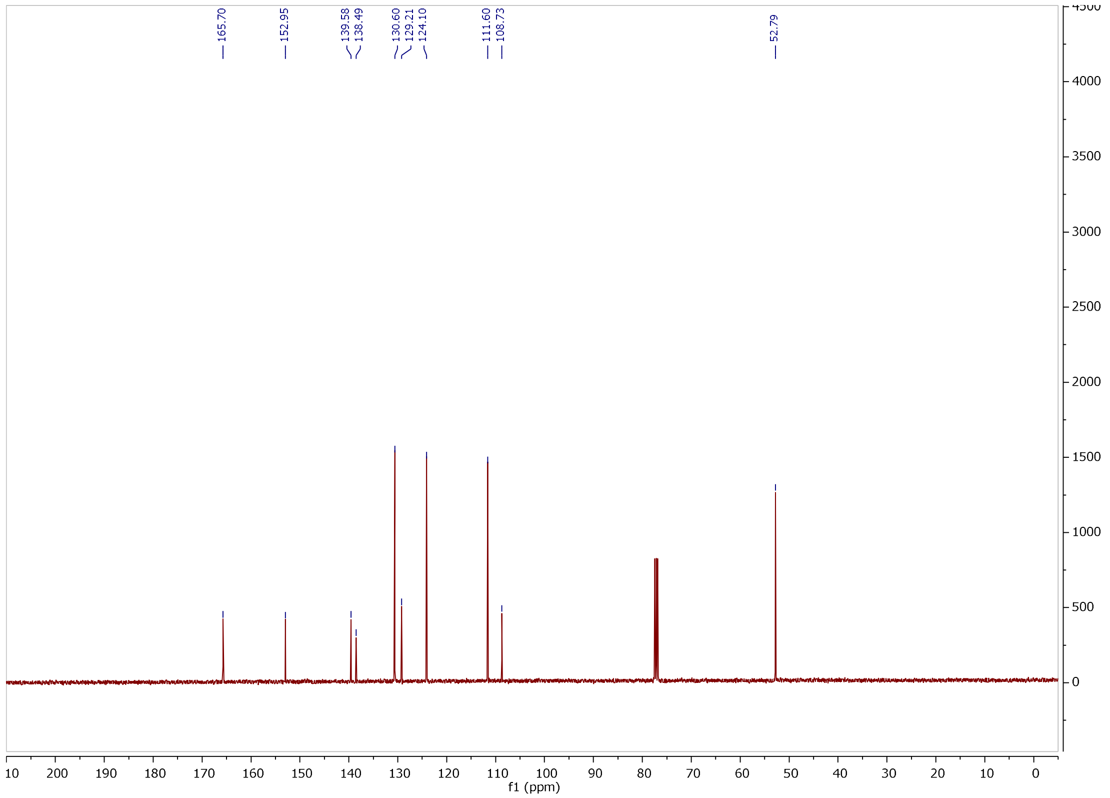
165.70
152.95
139.58
138.49
130.60
129.21
124.10
111.60
108.73
52.79
f1 (ppm)

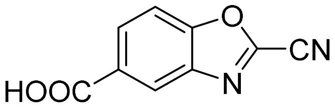

Compound 95

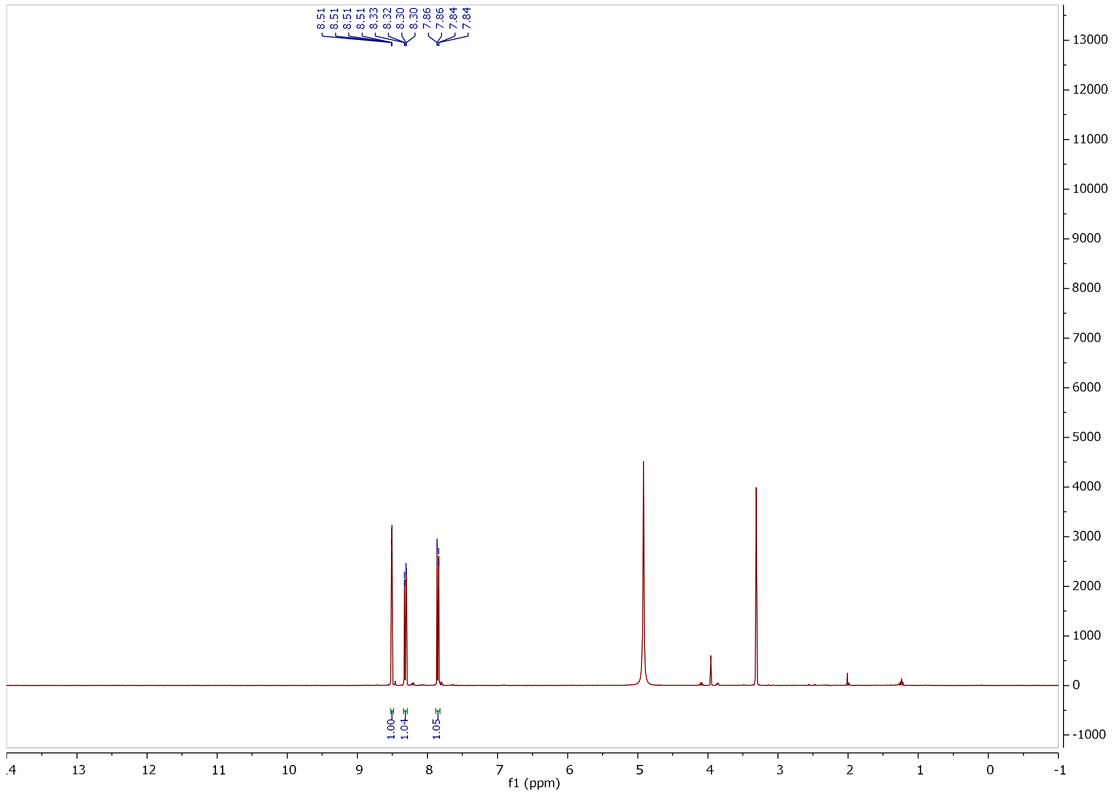

8.51
8.51
8.51
8.51
8.33
8.32
8.30
8.30
7.86
7.86
7.84
7.84
1.00
1.04
1.05
f1 (ppm)

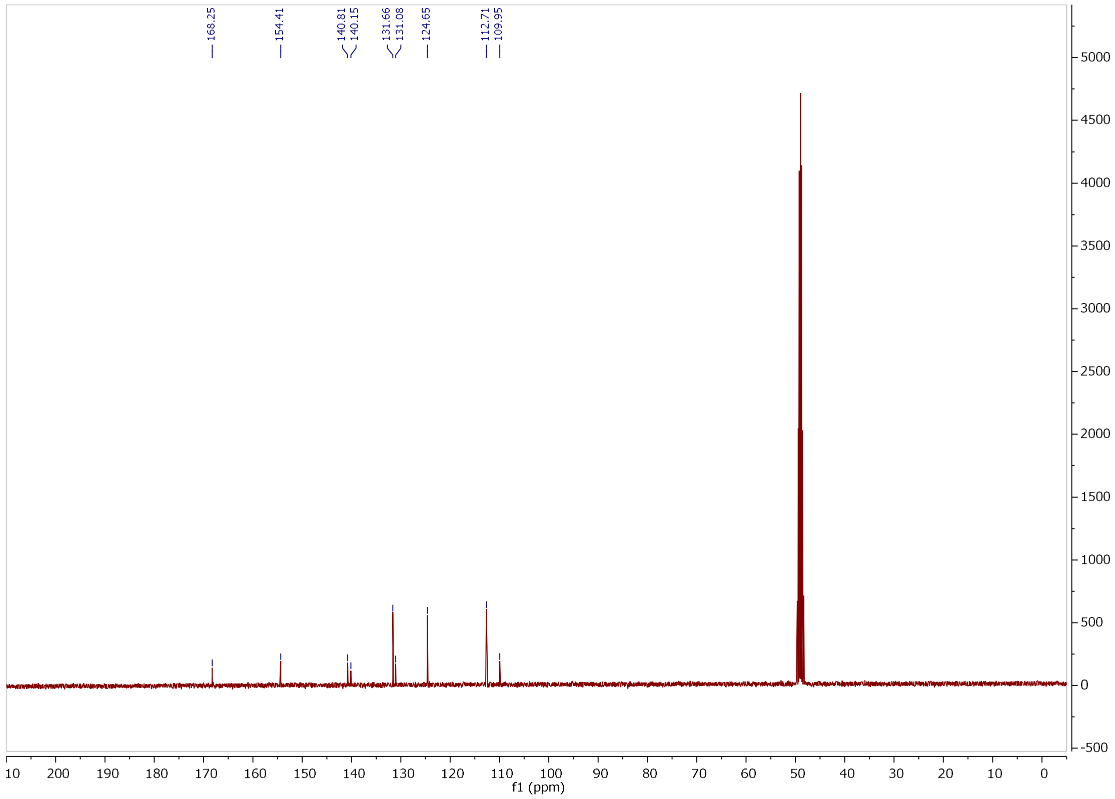

168.25
154.41
140.81
140.15
131.66
131.08
124.65
112.71
109.95
f1 (ppm)

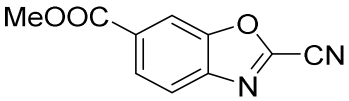

Compound 102

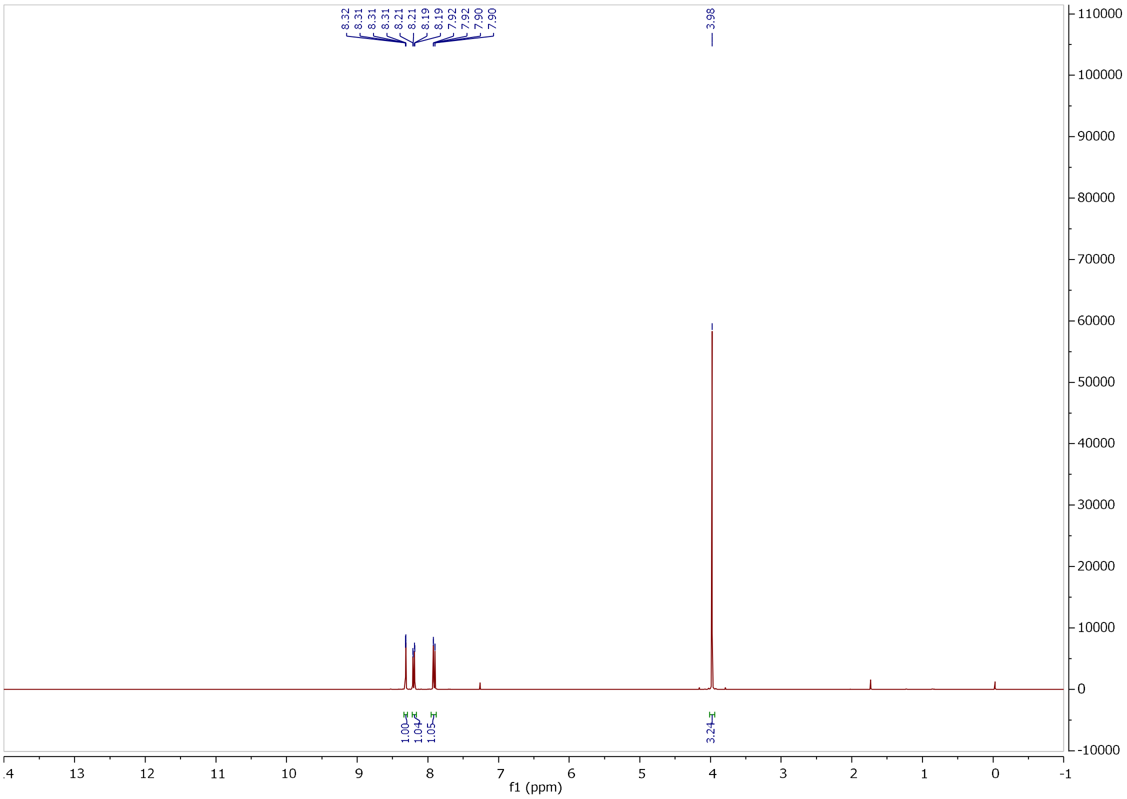

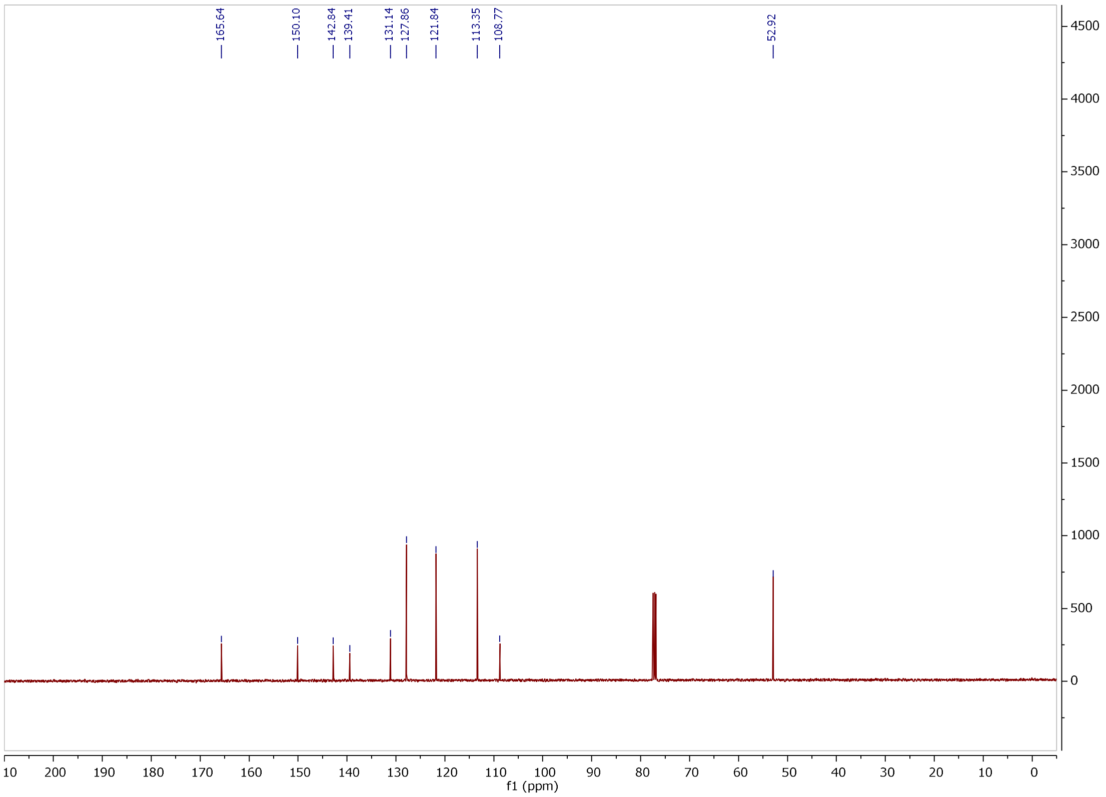

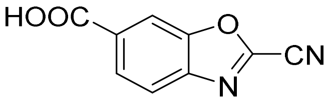

Compound 103

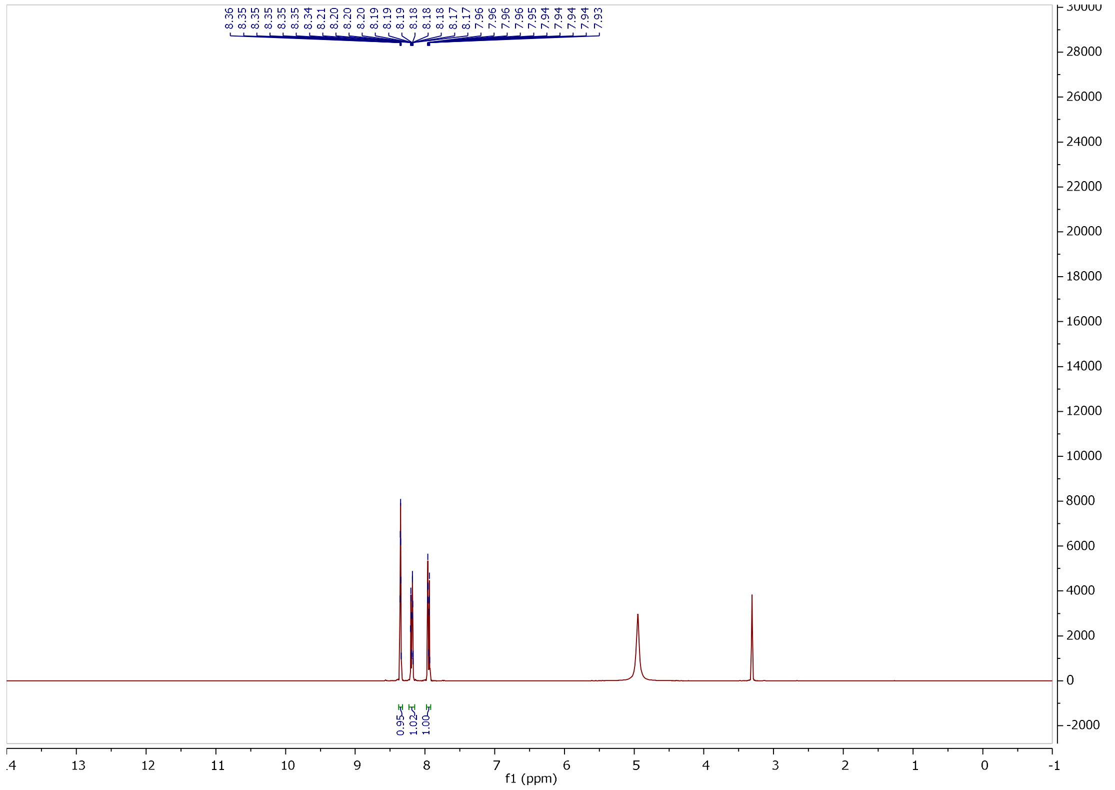

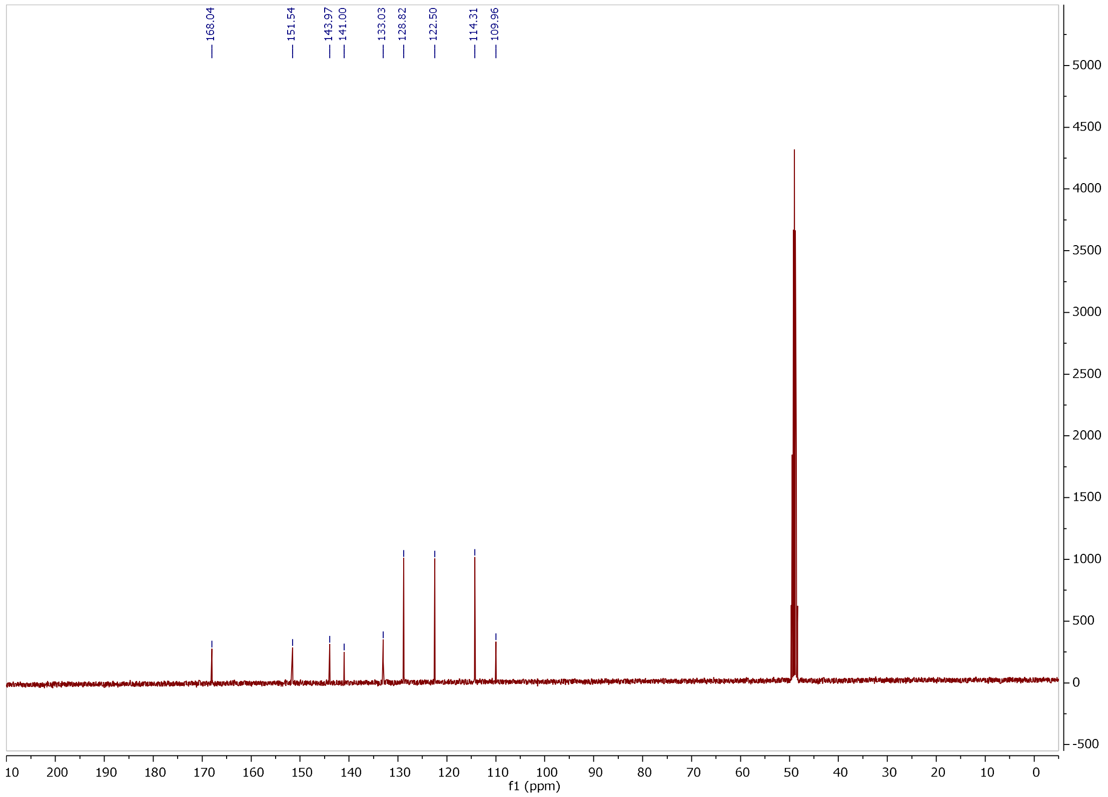

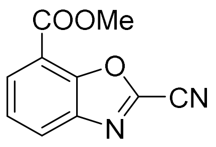

Compound 110

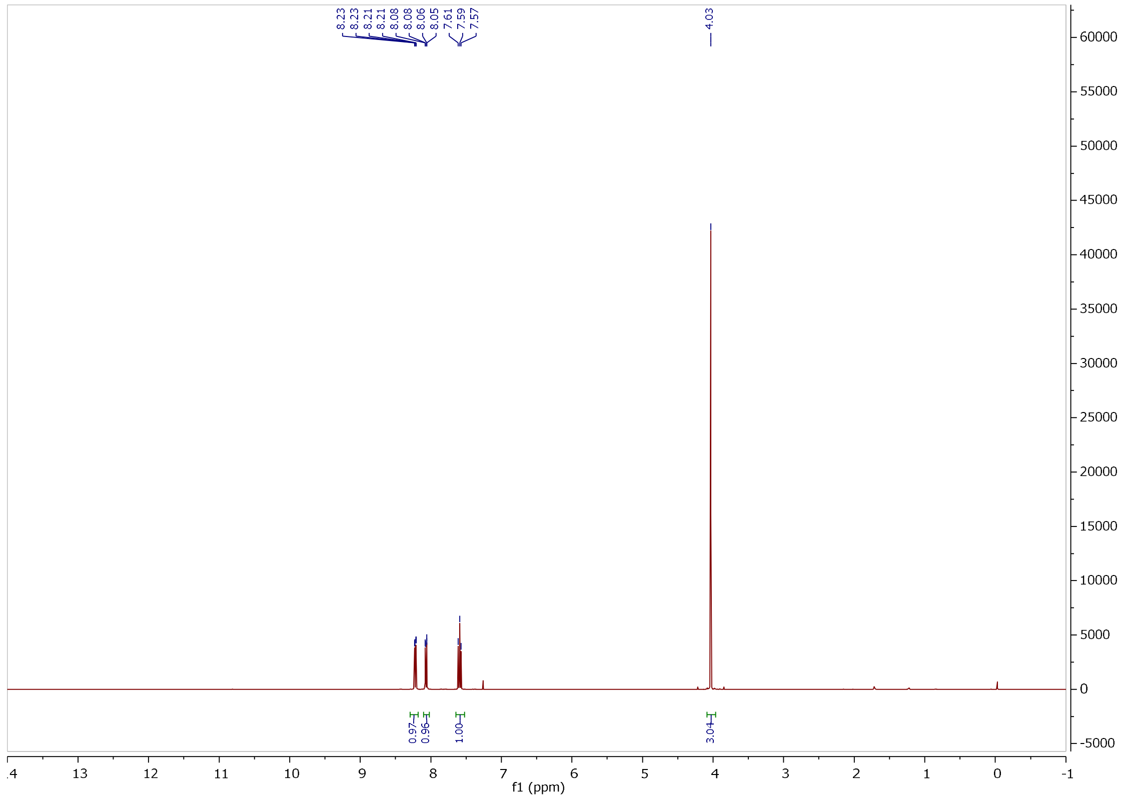

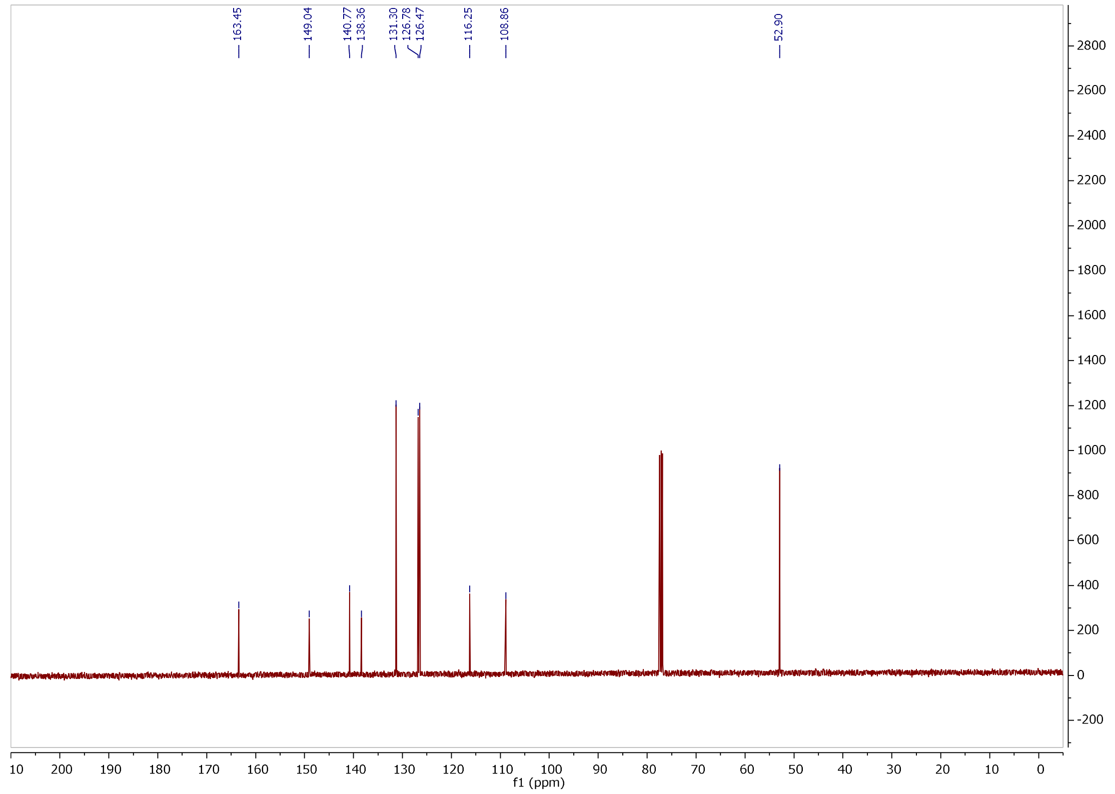

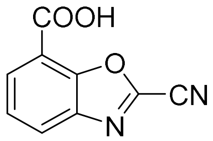

Compound 111

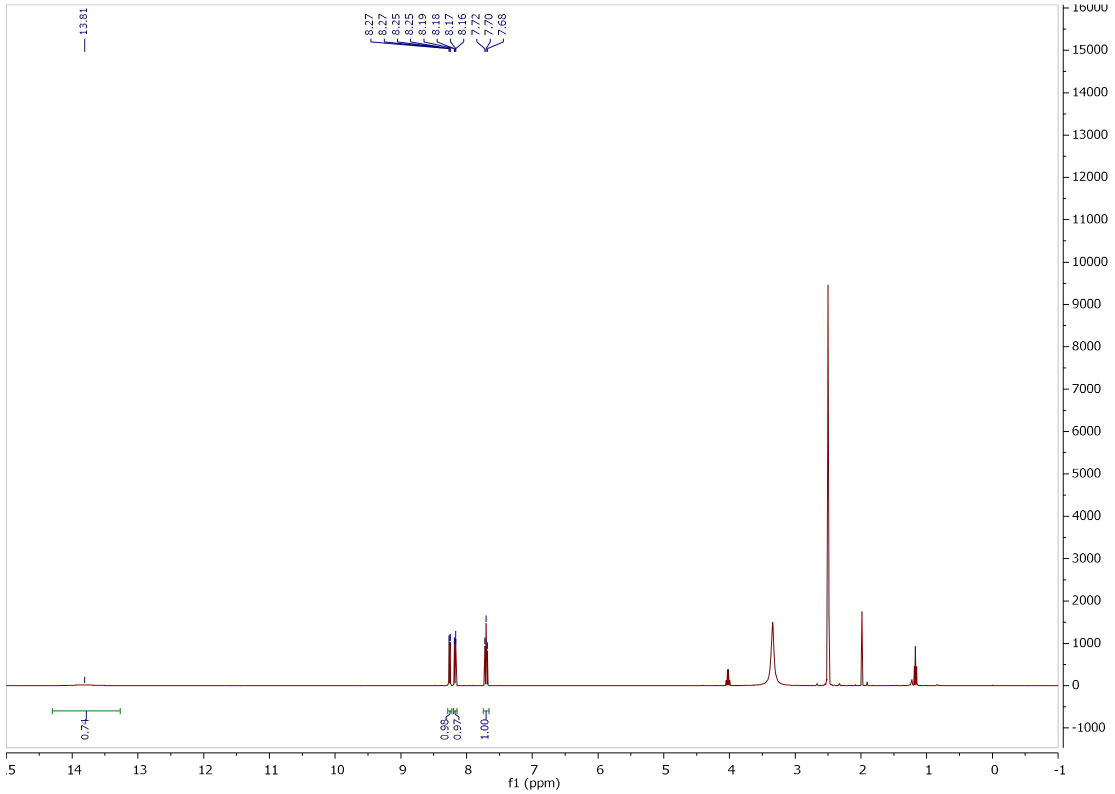

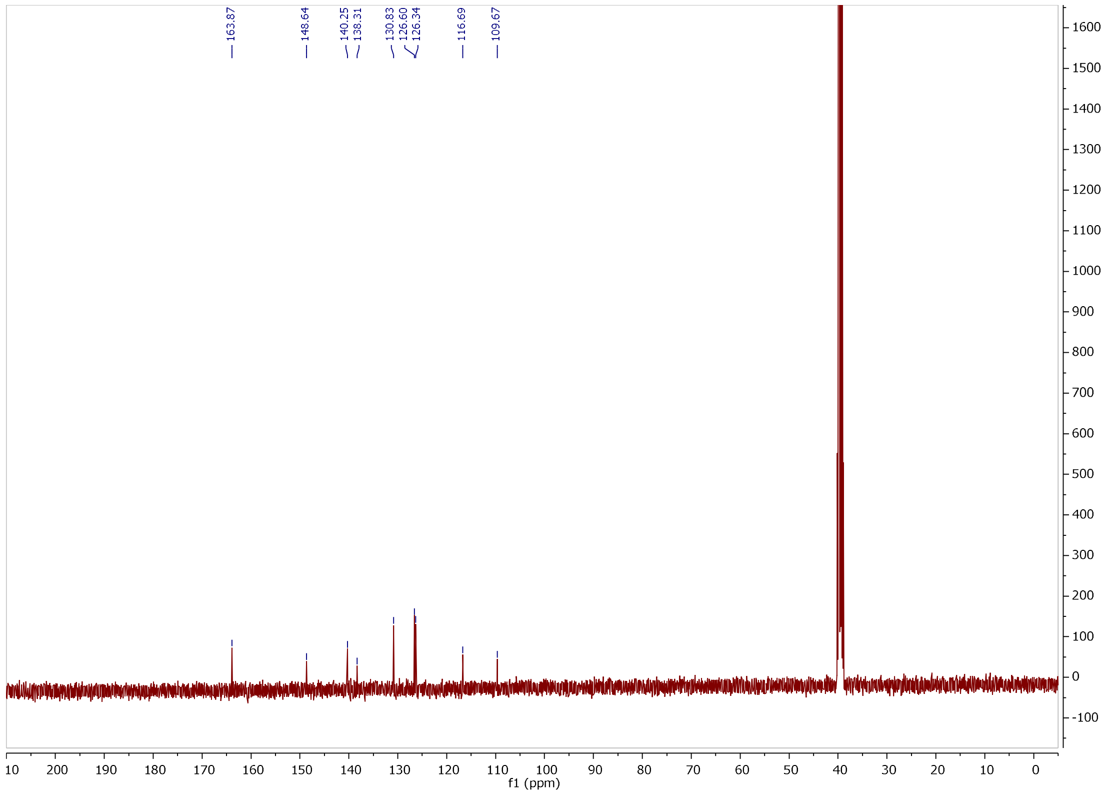

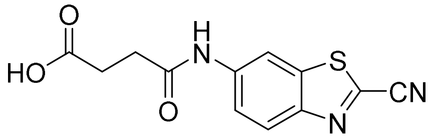

Compound 117

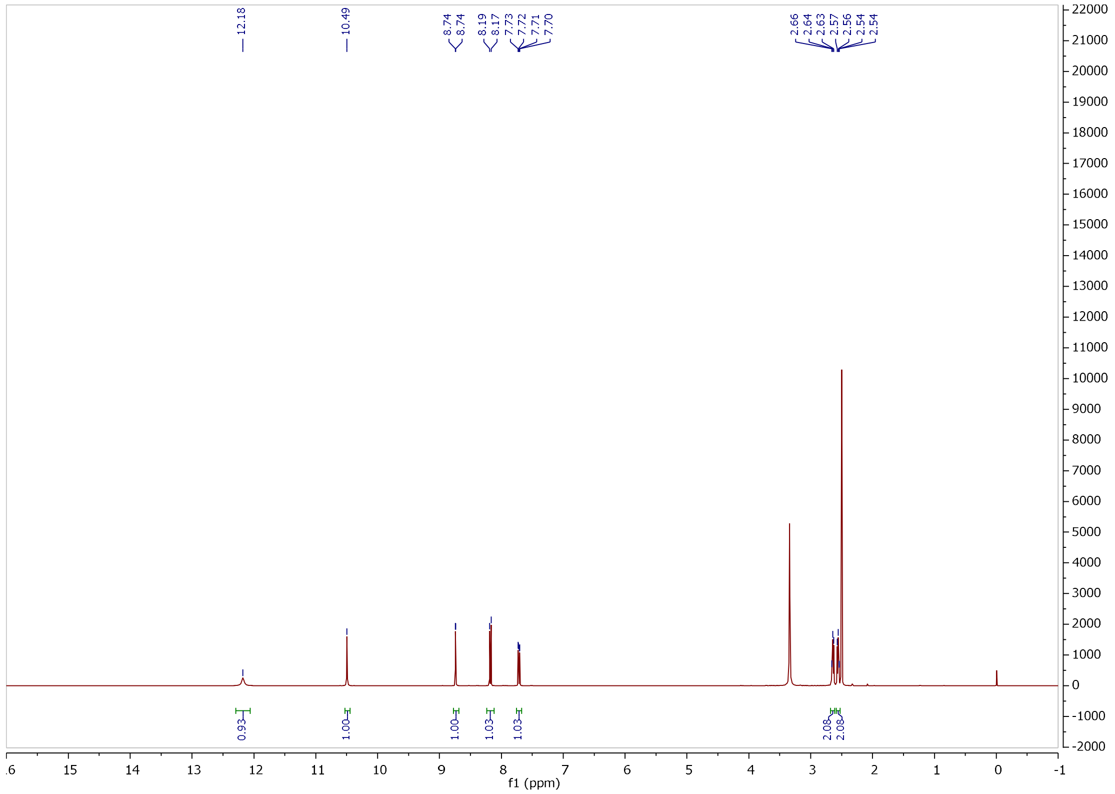

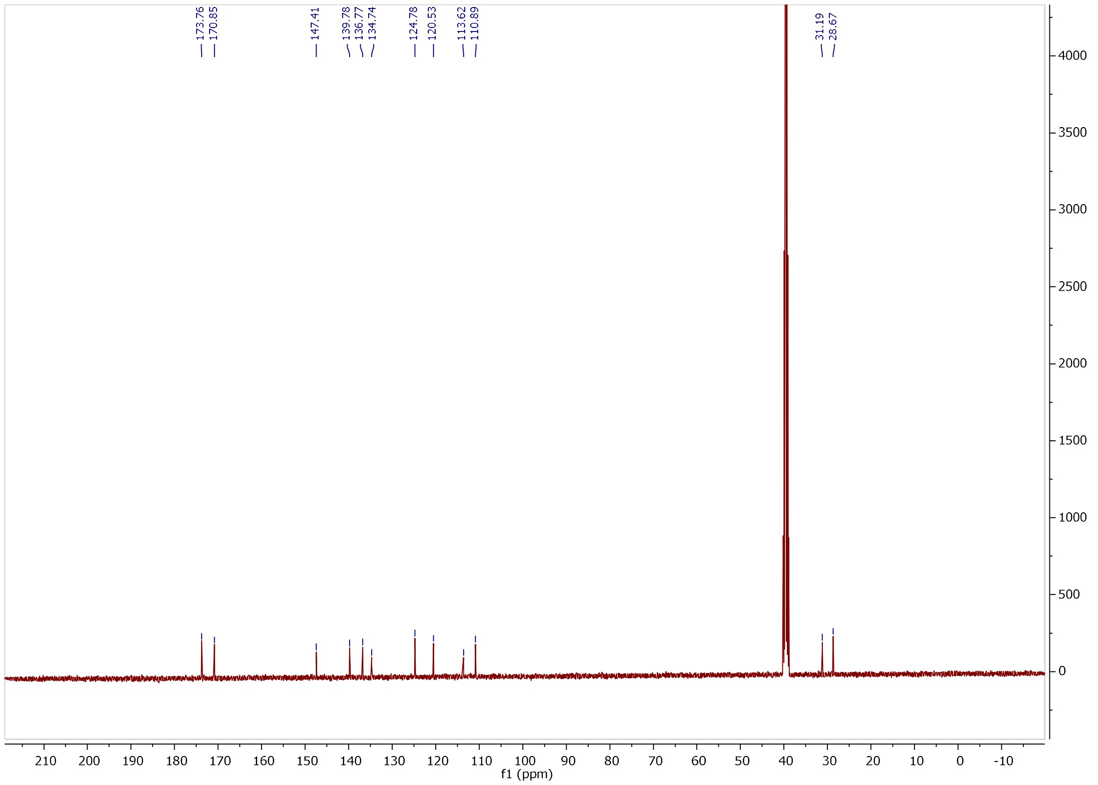

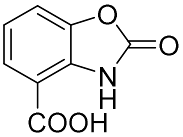

Compound 125

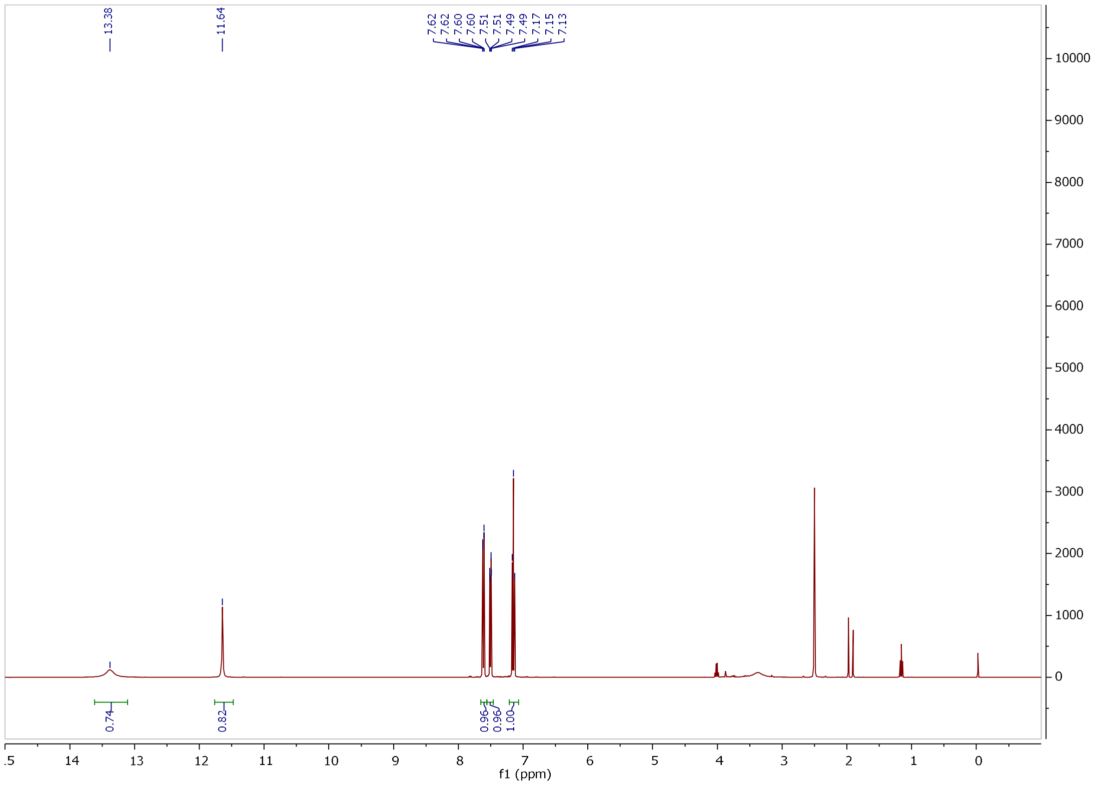

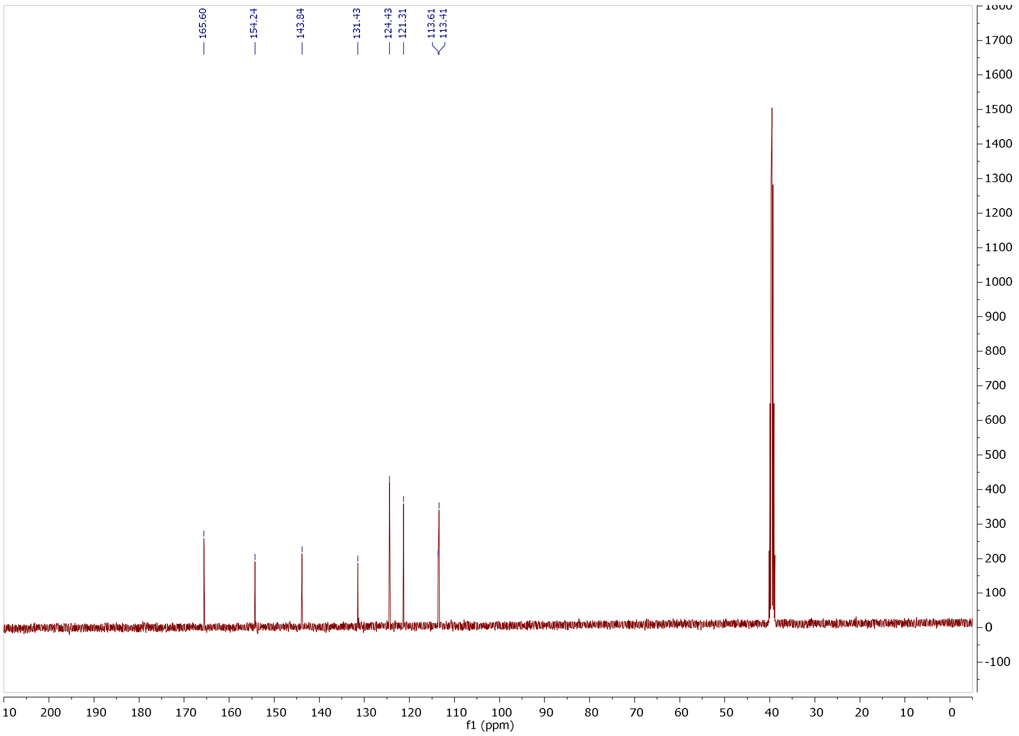

Supplement: Supplementary file 2 — bc3c00163_si_002.pdf [file bc3c00163_si_002.pdf]
