# Supplementary material for: Tunable Heteroaromatic Nitriles for Selective Bioorthogonal Click Reaction with Cysteine
Source: Bioconjug Chem. 2023 Jun 24;34(7):1271–81. doi: 10.1021/acs.bioconjchem.3c00163 (PMC10360065; doi:10.1021/acs.bioconjchem.3c00163)
Supplement: Supplementary file 3 — bc3c00163_si_003.pdf [file bc3c00163_si_003.pdf]

Supporting Information

Tunable heteroaromatic nitriles for selective bioorthogonal click reaction with cysteine

Matic Proj [a], Nika Strašek [a], Stane Pajk [a], Damijan Knez [a,*], Izidor Sosič [a,*]

[a] *University of Ljubljana, Faculty of Pharmacy, Department of Pharmaceutical Chemistry, Slovenia*

* *Corresponding authors. E-mail addresses: damijan.knez@ffa.uni-lj.si, izidor.sosic@ffa.uni-lj.si*

Table of contents

1 Experimental procedures S2

1.1 Chemistry – General Information S2

1.2 UV-Vis-based stability and reactivity assays S4

1.3 NMR-based stability assay S7

1.4 DTNB thiol reactivity assay S7

1.5 Oligopeptide labeling S7

1.6 Global score calculation S8

2 Supporting Schemes, Tables, and Figures S10

3 Stability and Reactivity Screening S36

4 Oligopeptide Labeling S51

5 Chemistry S97

6 Supporting References S130

1 Experimental procedures

1.1 Chemistry – General Information

The reagents and solvents were used as received from commercial suppliers. After extraction, organic phases were dried over anhydrous Na_2SO_4. Reactions were monitored using analytical thin-layer chromatography (TLC) on silica gel 60 F_{254} Al plates (Merck, 20 × 20 cm). Developed plates were inspected under UV light and visualized with ninhydrin, $FeCl_3$, or bromocresol green stains. Normal phase d column "flash" chromatography was performed on Silica gel 60 (particle size: 0.035–0.070 mm, Merck). Nuclear magnetic resonance (NMR) spectra were recorded on a Bruker Avance III 400 MHz spectrometer at 400 MHz for ^{1}H, 101 MHz for ^{13}C, respectively, using DMSO-d_6, $CDCl_3$, acetone-d_6, CD_3OD and TFA-*d* as solvents. Chemical shifts are reported in *parts per million* (*ppm*), the central peak of the residual non-deuterated solvent signal was used as the reference, *i.e.*, for $CDCl_3$ at 7.26 ppm for ^{1}H and 77.16 ppm for ^{13}C, for DMSO-d_6 at 2.50 ppm for ^{1}H and 39.52 ppm for ^{13}C, for acetone-d_6 at 2.05 ppm for ^{1}H and 29.84 ppm for ^{13}C, and for CD_3OD at 3.31 ppm for ^{1}H and 49.00 ppm for ^{13}C. The multiplicities are reported as s (singlet), d (doublet), t (triplet), q (quartet), p (pentet), m (multiplet), dd (doublet of doublets), ddd (doublet doublet of doublets), td (triplet of doublets), qd (quartet of doublets), and br (broad), and coupling constants (*J*) in Hertz (Hz). Mass spectra were recorded on Thermo Scientific Q Exactive Plus Orbitrap LC-MS/MS and Advion Expression CMS mass spectrometers, and IR spectra on Thermo Nicolet FT-IR spectrophotometer. UHPLC analyses were performed on Thermo Scientific Dionex UltiMate 3000 modular system (Thermo Fisher Scientific Inc.). The general method (Method I) used a Waters Acquity UPLC® HSS C18 SB column (2.1 × 50 mm, 1.8 μm) thermostated at 40 °C, with: injection volume, 5 μL; sample, 0.1–0.2 mg/mL in MeOH; flow rate, 0.4 mL/min; detector λ, 254 and 280 nm; mobile phase A: 0.1% TFA (v/v) in water; mobile phase B: MeCN. Gradient: 0–2 min, 10% B; 2–5 min, 10%–90% B; 5–8 min, 90% B. Method II had the same conditions except gradient: 0–2 min, 2% B; 2–8 min, 2–90% B; 5–8 min, 90% B. Method III had the same conditions as Method I except column – BEH Acquity UPLC® C18 (2.1 × 50 mm, 1.7 μm) thermostated at 40 °C, and injection volume, 1 μL. All compounds are >95% pure by UHPLC analysis.

General procedure 1 (GP1) – BenzXazole-2-carbonitrile formation using Appel's salt: Aniline derivative (1.0 eq) was dissolved in pyridine (0.1 M) under Ar and Appel's salt – 4,5-dichloro-1,2,3-dithiazolium chloride (1.2 eq.) was added. Reaction mixture was stirred for 16 h at 70 °C

before solvent was evaporated and EtOAc (50 mL) was added. Solid was filtered off, discarded and the organic filtrate washed with 2 M $HCl_{(aq)}$ (2 × 50 mL), saturated brine (50 mL), dried, filtered and volatile components evaporated *in vacuo*. The product was purified by column chromatography.

General procedure 2 (GP2) – Benzo[d]thiazole-2-carbonitrile formation using 2-bromoanilines: To a solution of starting 2-bromoaniline (1.0 eq) in anhydrous DCM (25 mL), Appel's salt – 4,5-dichloro-1,2,3-dithiazolium chloride (1.2 eq.) and anhydrous pyridine (2.0 eq) were added under Ar, The reaction mixture was stirred for 2 h at rt, then solid was filtered off (discarded), and the filtrate evaporated *in vacuo* to afford crude substituted 4-chloro-*N*-aryl-5*H*-1,2,3-dithiazol-5-imine [33]. Imine was charged in glass vial, dissolved in anhydrous pyridine (5 mL), CuI (2.0 eq) added, and heated under microwave irradiation for 20 min at 115 °C, solid filtered off, washed with EtOAc (20 mL) and organic phase evaporated *in vacuo*. The product was isolated by column chromatography.

General procedure 3 (GP3) – Methylation: 1*H*-Benzo[*d*]imidazole-2-carbonitrile (1.0 eq) was dissolved in MeCN (0.1 M) under Ar, and K_2CO_3 (1.2 eq.) and MeI (1.2 eq.) were added. After stirring the reaction mixture at rt for 72 h, volatile components were removed, residue dissolved in EtOAc (50 mL), and washed with H_2O (30 mL), saturated brine (50 mL), dried, filtered and volatile components evaporated *in vacuo*. Positional isomers were isolated and purified by column chromatography.

General procedure 4 (GP4) – Reduction: Starting nitro compound (1.0 eq) was dissolved in AcOH (5 mL), the iron powder (10 eq.) was added and the resulting mixture vigorously stirred at rt for 2 h. Excess iron was removed by filtration over a pad of Celite®545, washed with tetrahydrofuran and MeOH, volatile components evaporated *in vacuo*, and the residue purified by column chromatography or crystallization to afford pure product.

General procedure 5 (GP5) – Ester deprotection: Starting methyl ester (1.0 eq), aluminum (1.2 eq), and iodine (1.5 eq) were dissolved in anhydrous MeCN (5 mL) under Ar [35]. The reaction mixture was stirred at 80 °C for 18 h, then cooled to rt and quenched with 2 M $HCl_{(aq)}$ (10 mL), organic volatiles evaporated *in vacuo*, and the aqueous phase extracted with EtOAc (2 × 20 mL),

dried, filtered and volatile components evaporated *in vacuo*. The product was isolated by column chromatography.

General procedure 6 (GP6) – Ether deprotection: O-Methyl ether (1.0 eq) was dissolved or suspended in anhydrous DCM (10 mL) under Ar, and aluminum chloride (5.0 eq) was added. After stirring the reaction mixture at 40 °C for 18 h, volatile components were evaporated *in vacuo*, and residue dissolved in EtOAc (30 mL), washed with 1 M $HCl_{(aq)}$ (20 mL), saturated brine (20 mL), dried, filtered and volatile components evaporated *in vacuo*. The product was isolated by column chromatography.

General procedure 7 (GP7) – RP-CC purification: Compounds were purified by reversed-phase column chromatography (RP-CC) (Isolera Biotage One Flash Chromatography system, Biotage® Sfär C18 Duo 100 Å 30 µm column, 30 g) using a gradient of 0.1% $TFA_{(aq)}$ and MeCN as eluent (gradient 0–100% MeCN in 5 column volumes (300 mL); 100% MeCN for 1 column volumes (100 mL)). After the RP-CC, fractions containing the product were combined and organic volatiles were evaporated *in vacuo*. The remaining aqueous solution was made alkaline (pH = 14) with 2 M $NaOH_{(aq)}$ and extracted with DCM (2 × 20 mL). The combined organic phases were dried, filtered, and volatile components evaporated *in vacuo*.

1.2 UV-Vis-based stability and reactivity assays

The reagent solutions were freshly prepared before performing the experiments, and all solutions were pre-incubated at 37 °C. The analysis of the results was automated using a Python script (available at https://github.com/maticproj/UV-Vis-analysis). Figures were generated using Matplotlib v3.3.4 (https://github.com/matplotlib/matplotlib) for Python v3.7 (https://www.python.org/).

1.2.1 Screening

The aqueous stability and reactivity were determined spectrophotometrically by following the changes in the absorption spectra of the compounds as reported previously [34]. Experiments were performed in 96-well UV-transparent microplates (Corning, CLS3635) in assay buffer (50 mM Tris-HCl, 0.5 mM EDTA, pH 7.4). Briefly, 135 µL of assay buffer was pipetted in each well, followed by the addition of 15 µL of 1 mM compound stock solution in DMSO. Next, 150 µL of assay buffer or 1 mM reagent solution (TCEP, L-Lys, L-Ser, NAC or L-Cys) in assay buffer was

added. The final reaction mixture contained compound (50 µM), reagent (0.5 mM), and DMSO (5%) in assay buffer. The plate was incubated without lid at 37 °C in a plate reader (Synergy H4, BioTek Instruments, Inc., Santa Clara, CA, USA) and the absorbance spectrum (244–400 nm) was acquired in sweep mode after 0, 15, 30, 60, 120, 180, and 240 min using a discontinuous kinetic procedure in Gen5 software (BioTek Instruments, Inc., Santa Clara, CA, USA). The time required to read the entire 96-well plate was 3 min. To determine the baseline, the compound solution was replaced with pure DMSO and subtracted from each reading. Compounds with an absorbance maximum of less than 0.10 AU were not evaluated using these assays, and were labeled with low absorbance.

Aqueous stability was evaluated from the first experiment. The relative absorbance difference between the first time point and 240 min at the most responsive wavelength was calculated. If the relative absorbance difference for the compound in the buffer was below 0.1, between 0.1–0.2, and above 0.2, the compound was classified as stable, intermediate, and unstable, respectively.

From the experiments with added reagents, reactivity with L-Lys, L-Ser, TCEP, NAC, or L-Cys was evaluated. To detect hyperreactive compounds where the reaction with the reagent was complete before the first time point could be acquired, *i.e.*, up to 3 min, the spectra of the compound in the buffer solution with or without reagent at the first time point (t = 0) were compared. Significant changes (*i.e.*, relative absorbance difference at the most responsive wavelength above 0.2) indicated hyperreactivity (reactivity above the limit of detection). For other compounds, the relative absorbance difference between the first time point and 240 min at the most responsive wavelength was calculated. If the relative absorbance difference was above 0.2, the compound was classified as reactive.

1.2.2 Reaction rate evaluation from a single Cys concentration

For compounds reactive with Cys, the data from the screening experiment was used for second order reaction rate determination:

$$\text{cpd} + \text{Cys} \rightarrow \text{cpd-Cys}$$

$$\frac{-d[cpd]}{dt} = k_2[cpd][Cys] \qquad \text{(Equation 1)}$$

As the measurement was performed under pseudo-first-order conditions using 10-fold excess of Cys over compound, we stated as follows:

$$\frac{-d[cpd]}{dt} = k_{obs}[cpd] \qquad \text{(Equation 2)}$$

Then:

$$k_{obs}[cpd] = k_2[cpd][Cys]$$

$$k_{obs} = k_2[Cys] \qquad \text{(Equation 3)}$$

The pseudo-first-order rate constants (k_{obs}) were obtained by fitting the data using SciPy v1.6.2 (https://scipy.org/) function *curve_fit* for one-phase decay equation:

$$y = (y_0 - Plateau) * e^{-k_{obs} * x} + Plateau \qquad \text{(Equation 4)}$$

where x is time and y is absorbance with subtracted blank. The second-order reaction rate constant (k_2) was obtained by dividing k_{obs} with Cys concentration (0.5 mM).

1.2.3 Reaction rate evaluation from multiple Cys concentrations

For compounds hyperreactive with Cys under the screening conditions, a published procedure [47] was modified to achieve higher throughput. The measurements were performed under pseudo-first-order conditions using at least 10-fold excess of Cys over compound at seven different concentrations of Cys. Experiments were performed in 96-well UV-transparent microplates (Corning, CLS3635) in assay buffer (PBS, pH 7.4). Briefly, 135 µL of assay buffer was pipetted in each well, followed by the addition of 15 µL of 1 mM compound stock solution in DMSO. Next, 150 µL of Cys solution in buffer (0–4 mM) supplemented with 1 mM TCEP was added. The final reaction mixture contained compound (50 µM), Cys (0, 0.5, 0.75, 1, 1.25, 1.5, 1.75, or 2 mM), TCEP (0.5 mM), and DMSO (5%) in assay buffer. The plate was shaken, and the absorbance was monitored at the most responsive wavelength (260–390 nm) for 5 minutes using Synergy H4 microplate reader (BioTek Instruments, Inc., USA) for one compound at a time in duplicate. The delay between the addition of Cys and the first measurement was below 15 s. The most responsive wavelength was determined in the previous experiment. A blank (experiment without any Cys) was subtracted from each measurement. Next, the pseudo-first-order rate constants (k_{obs}) were

obtained by fitting the data using "one-phase association/decay" in GraphPad Prism (GraphPad Software, San Diego, CA, USA). The second-order reaction rate constant (k_2) was obtained by linear regression of k_{obs} plotted at different concentrations of Cys. Data were obtained from at least two independent experiments performed in duplicate, and are reported as the mean and standard deviation of the mean.

1.3 NMR-based stability assay

Compounds were dissolved in DMSO-*d*$_6$ (5 mM) and diluted 5–fold with buffer (50 mM Tris-HCl, 0.5 mM EDTA, pH 7.4). The solution was mixed, and an NMR spectrum was acquired immediately on a Bruker Avance III 400 MHz spectrometer using water suppression pulse sequence (N WATERSUP, noesygppr1d, default settings). Next, the NMR tube was incubated in a water bath at 37 °C and additional spectra were acquired after 1 h and 4 h.

1.4 DTNB thiol reactivity assay

The assay was performed as reported previously [43]. All reagent solutions were freshly prepared before performing the experiments. The reaction mixture contained compound (100 µM), DTNB (25 µM), TCEP (100 µM), and DMSO (5%) in buffer (20 mM sodium phosphate, 150 mM NaCl, pH 7.4). Absorbance at 412 nm was measured every 5 min for 4 h (Synergy H4, BioTek Instruments, Inc., USA) to monitor TNB^{2-} depletion. Compound background absorbance was subtracted from each measurement. 2-Chloro-*N*-(3-chlorophenyl)acetamide was used as a control.

1.5 Oligopeptide labeling

Oligopeptides (**UP1**, CGKGCGSGYGW; **UP2**, AGKGCGSGYGW) were prepared by GenScript Biotech (The Netherlands) with HPLC purity ≥95.0%. The oligopeptides were dissolved in DMSO (10 mM) and immediately diluted 2–fold with TCEP (100 mM) in assay buffer (PBS, pH 7.4) to obtain the oligopeptide stock solution containing oligopeptide (5 mM) and TCEP (50 mM). The reaction mixture contained compound (100 µM, 2 eq.), oligopeptide (50 µM, 1 eq.), TCEP (500 µM, 10 eq.), and DMSO (10%) in assay buffer. Samples were incubated in a shaker at 37 °C for 30 min, filtered into a vial, and analyzed immediately. For each peptide, a blank experiment was performed without compound.

For the reversibility assays (Cys and DTT treatment), samples were aliquoted after incubating compound with oligopeptides for 30 min. A 10 mM Cys or DTT solution was added to the first

aliquot to achieve a final concentration of 100 μM (2 eq.). Assay buffer was added to the second aliquot as a control. Both aliquots were incubated in a shaker at 37 °C for another 30 min, filtered into a vial, and analyzed immediately.

To determine the stability of adducts with **UP1**, we monitored the reaction for up to 24 h. Reaction mixtures were prepared in assay buffer (PBS, pH 7.4) at 10-fold higher concentrations than described above, containing compound (50 μM, 1 eq.), oligopeptide (50 μM, 1 eq.), TCEP (500 μM, 10 eq.), and DMSO (10%) in assay buffer. After 30 min incubation in a shaker at 37 °C, the reaction mixture was diluted with each of the final buffers (PBS, pH 7.4; 10 mM Tris-HCl, pH 7.4; 10 mM potassium phosphate buffer, pH 6.5; 10 mM potassium phosphate buffer, pH 5.5) and filtered into a vial, and analyzed immediately. The final mixture was incubated at 37 °C and analyzed at 4 h, 8 h, and 24 h intervals.

Samples were analyzed by an LCMS system, which included Thermo Scientific UltiMate 3000 UHPLC liquid chromatograph and Thermo Scientific Exactive Plus Hybrid Quadrupole-Orbitrap mass spectrometer. Chromatographic separation was performed on Waters Acquity UPLC BEH C18 column (50 × 2.1 mm, 1.7 μm particles), with Waters Acquity C18 BEH cartridge guard column (5 × 2.1 mm) and kept at 40 °C. Vials with samples were kept at 5 °C and the injection volume was 5.00 μL. The compounds were separated using mobile phase A consisting of water/acetonitrile/formic acid (950/50/1, *v*/*v* ratio), and mobile phase B consisting of water/acetonitrile/formic acid (950/50/1, *v*/*v* ratio). The flow rate was 0.40 mL/min with the following gradient: 0–1.0 min, 0% B; 1.0–5.0 min, 0%–100% B; 5.0–5.1 min, 100%–0% B; 5.1–8.0 min 0% B. The detection wavelength was set at 280 nm. From 1.0 min to 6.0 min the flow was diverted to mass spectrometer, mass analysis was performed between 1.1 min to 6.0 min. The mass spectrometer was operated in HESI positive or negative mode with the following MS parameters: sheath gas flow rate, 25 (arbitrary units); auxiliary gas flow rate, 10 (arbitrary units); capillary temperature, 350 °C; and spray voltage, 3.5 kV. HESI source was set at the height D.

1.6 Global score calculation

Second-order reaction rate constants (k_2 in s^{-1} M^{-1}) for reaction with Cys were converted to a negative logarithm and normalized between values 0–1. Selectivity was calculated as a ratio between **UP1** and **UP2** conversion, converted to a negative logarithm, and normalized between

values 0–1. Substituents with derivatization capability (amino, hydroxyl, and carboxylic acid) were scored with 1, whereas all other substituents were scored with 0. The global score (Equation 5) was calculated by addition of reactivity, selectivity, and derivatization capability with factors 1.5, 1, and 1, respectively.

$$Global\ score = 1.5 \times pk_2 + p_{selectivity} + derivatizatoion\ capability \qquad \text{(Equation 5)}$$

2 Supporting Schemes, Tables, and Figures

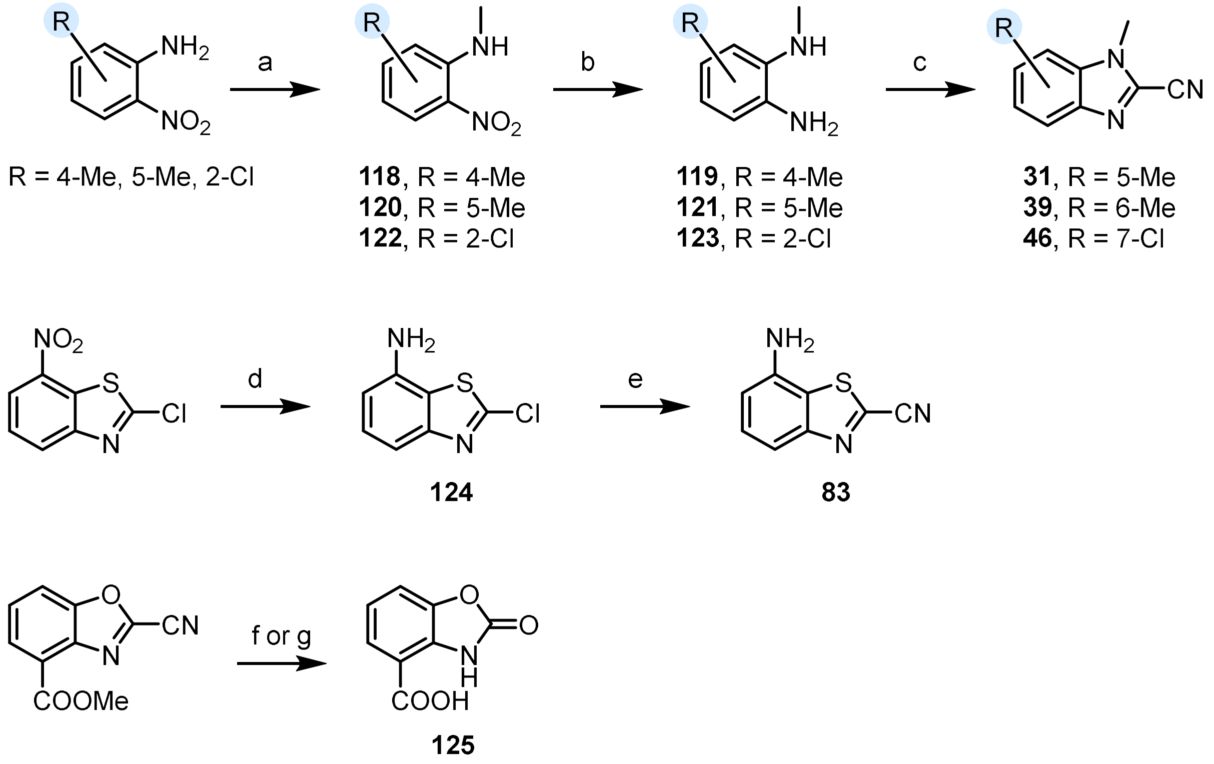

Scheme S1. Synthesis of benzXazoles and intermediates. Reaction conditions: a) corresponding 2-nitroaniline, MeI, NaH, dry DMF, 0 °C, 15 min, then rt, 18 h; b) $NaBH_4$, Pd/C (cat), MeOH, 0 °C, 1 h; c) Appel's salt (4,5-dichloro-1,2,3-dithiazolium chloride), pyridine, 70 °C, 16 h; d) Fe, AcOH, rt, 2 h; e) KCN, DABCO, dry DMF, 100 °C, 48 h; f) LiOH (2.0 eq), MeOH, H_2O, rt, 2 h; g) NaO*t*Bu, dry THF, rt, 24 h [1].

Table S1. References for compounds that were previously described or purchased from commercial vendors.

Substituent \ Core		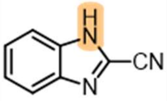 Benzimidazole	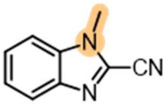 1-Methyl-benzimidazole	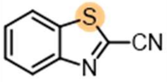 Benzothiazole	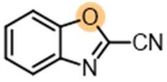 Benzoxazole
	/	1[a]	18[a]	51[a]	84[a]
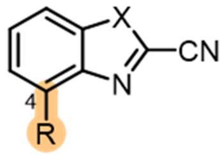	-NO_2	2	19	52	85[b]
	-COOMe	3	20	53	86
	-COOH	4	21	54	87
	-Cl	5	22	55[a]	88[b]
	-Me	6	23	56	89
	-OMe	7	24	57[c]	90[b]
	-OH	8	25	58	91[b]
	-NH_2	9	26	59	92[b]
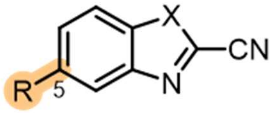	-NO_2	10	27	60[c]	93[b]
	-COOMe	11	28	61	94
	-COOH	12	29	62	95
	-Cl	13	30	63	96[a]
	-Me	14	31	64	97
	-OMe	15	32	65[c]	98[b]
	-OH	16	33	66	99[b]
	-NH_2	17	34	67[c]	100[b]
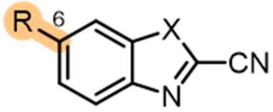	-NO_2		35	68	101[b]
	-COOMe		36	69	102
	-COOH		37	70	103
	-Cl		38	71[a]	104[a]
	-Me		39	72	105[b]
	-OMe		40	73[c]	106[b]
	-OH		41	74[c]	107[b]
	-NH_2		42	75	108[b]
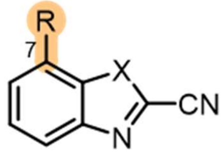	-NO_2		43	76	109[b]
	-COOMe		44	77	110
	-COOH		45	78	111
	-Cl		46	79[a]	112[a]
	-Me		47	80	113
	-OMe		48	81	114[b]
	-OH		49	82	115[b]
	-NH_2		50	83	116[b]
Unstable in solid or not accessible					

[a] Synthesis and characterization described in: Kollár et al., *European Journal of Medicinal Chemistry 219 (July 5, 2021): 113455.* https://doi.org/10.1016/j.ejmech.2021.113455.

[b] Synthesis and characterization described in: Kollár et al., *Cells 10, no. 12 (December 2021): 3431.* https://doi.org/10.3390/cells10123431.

[c] Purchased from commercial vendors

Table S2. Repeatability experiment for the UV-Vis-based stability assay. For each compound three independent experiments were performed.

Label	Aqueous stability	Most responsive wavelength	Absolute absorbance difference at the most responsive wavelength (0–240 min)	Relative absorbance difference at the most responsive wavelength (0–240 min)	Mean	SD	10×SD
**10**	Stable	262	0.034	0.0335	0.05	0.02	0.15
		264	0.062	0.0620			
		264	0.056	0.0579			
**34**	Stable	244	0.018	0.0419	0.05	0.01	0.13
		244	0.019	0.0507			
		244	0.027	0.0678			
**43**	Stable	260	0.025	0.0587	0.07	0.01	0.08
		260	0.026	0.0732			
		262	0.032	0.0713			
**85**	Unstable	300	0.112	0.3294	0.33	0.002	0.02
		296	0.111	0.3255			
		300	0.113	0.3294			
**101**	Unstable	282	0.237	0.4309	0.43	0.003	0.03
		282	0.234	0.4301			
		282	0.244	0.4349			

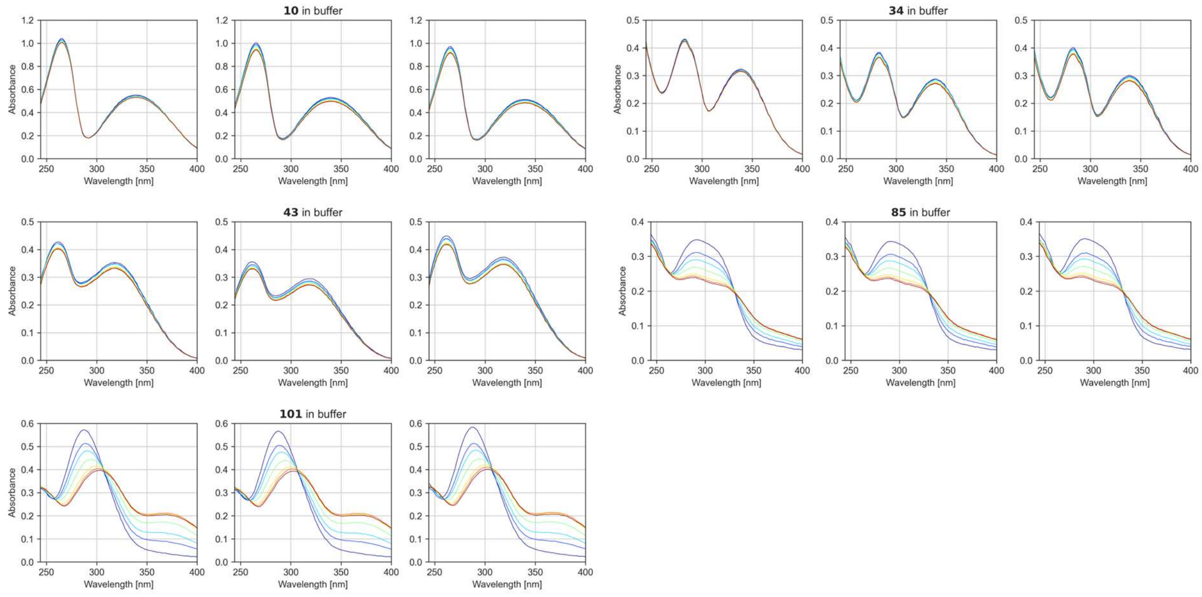

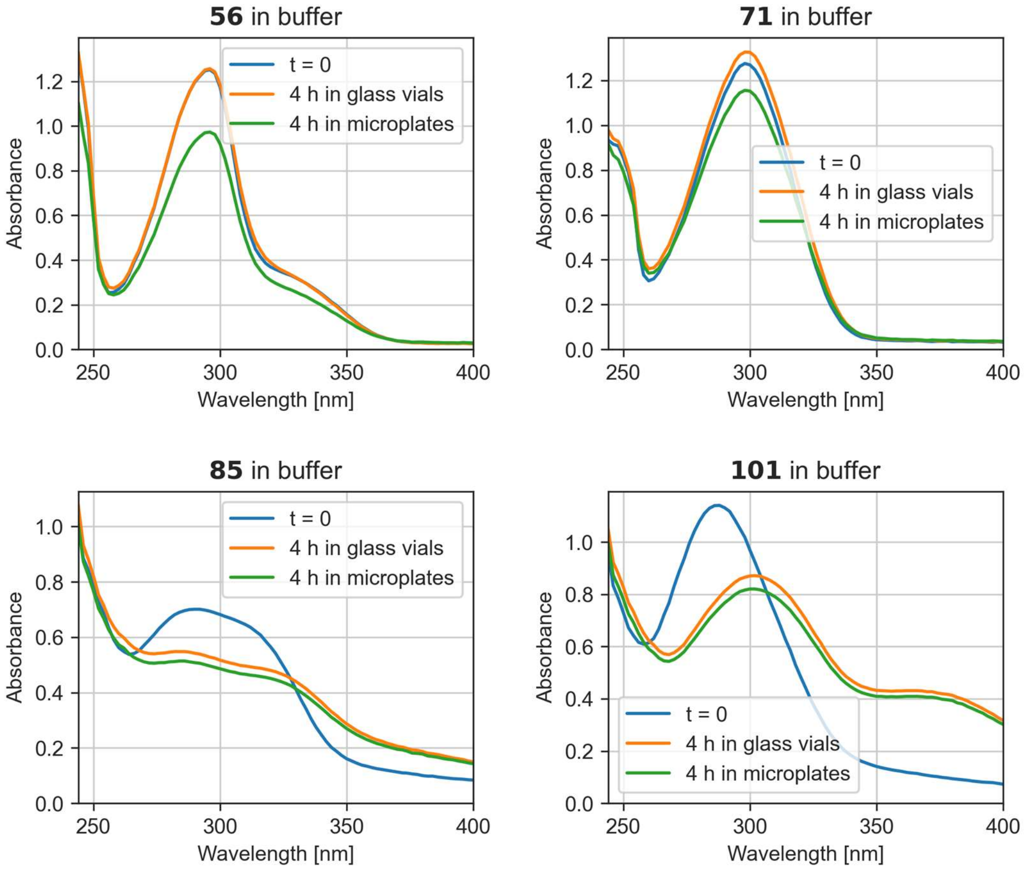

Figure S1. Compounds were incubated in buffer (50 mM Tris-HCl, 0.5 mM EDTA, pH 7.4) at 37 °C in glass vials or in acrylic copolymer UV-transparent microplates.

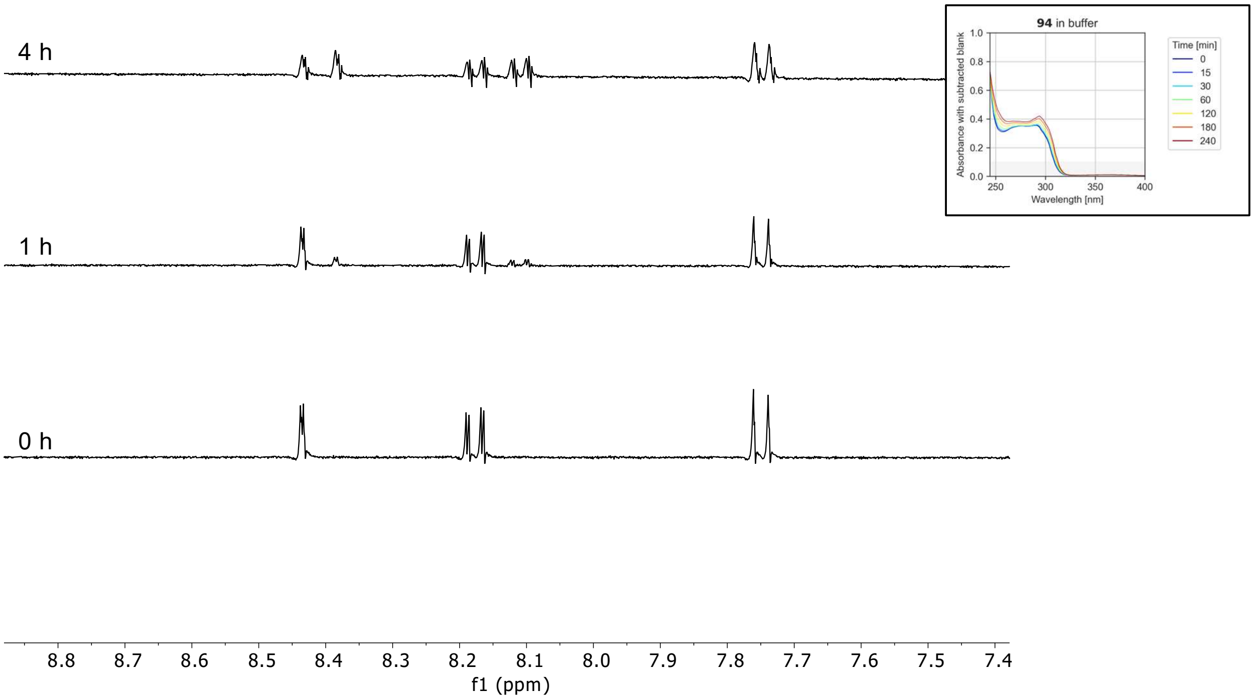

Figure S2. NMR-based stability assay for compound **94** along with time-dependent absorbance spectra from the UV-Vis-based stability assay (right).

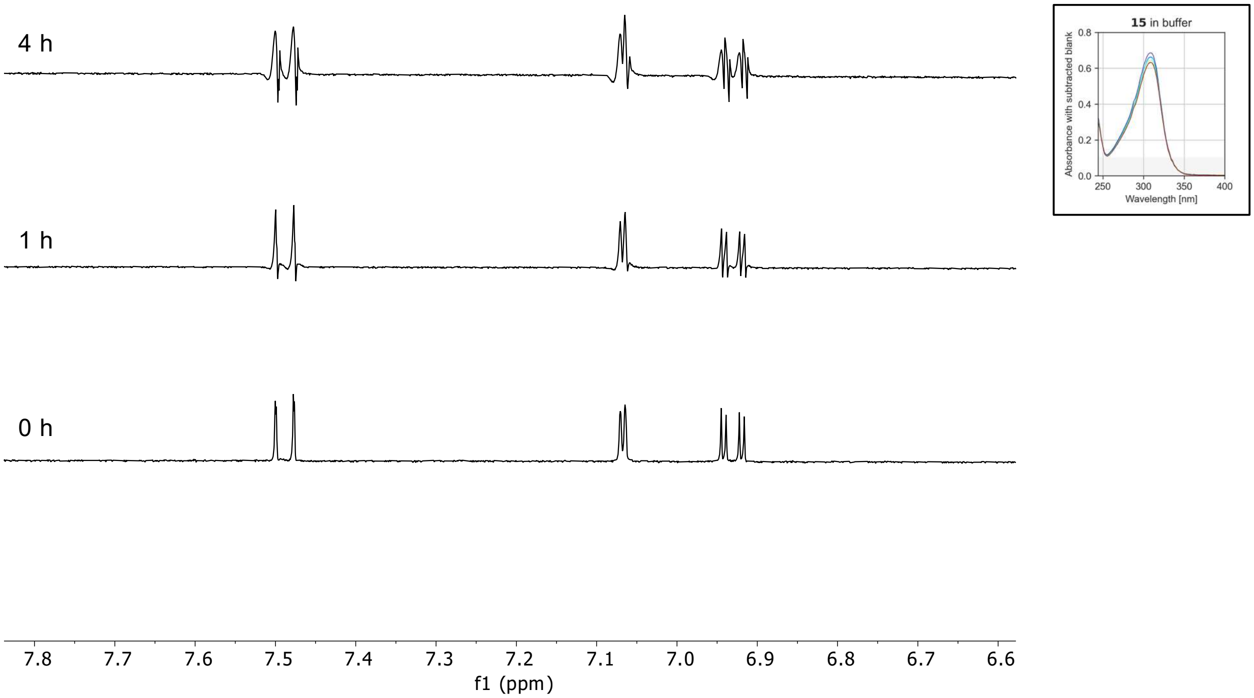

Figure S3. NMR-based stability assay for compound **15** along with time-dependent absorbance spectra from the UV-Vis-based stability assay (right).

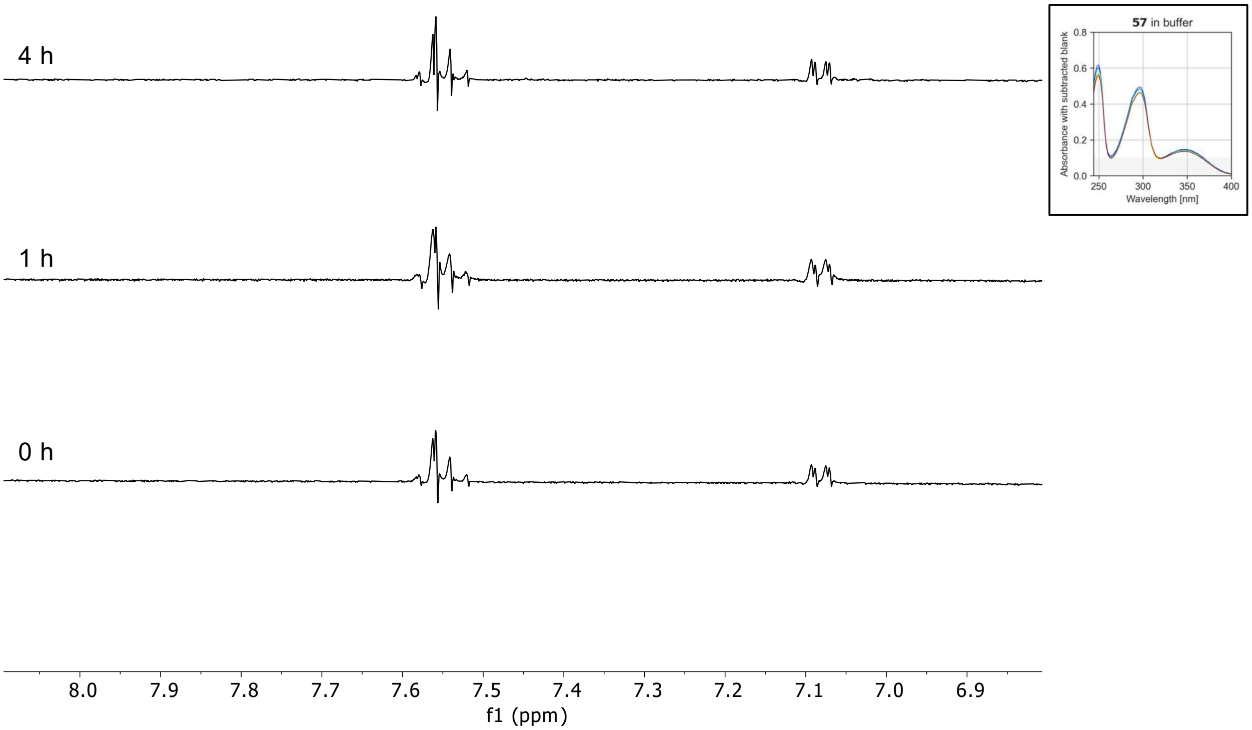

Figure S4. NMR-based stability assay for compound **57** along with time-dependent absorbance spectra from the UV-Vis-based stability assay (right).

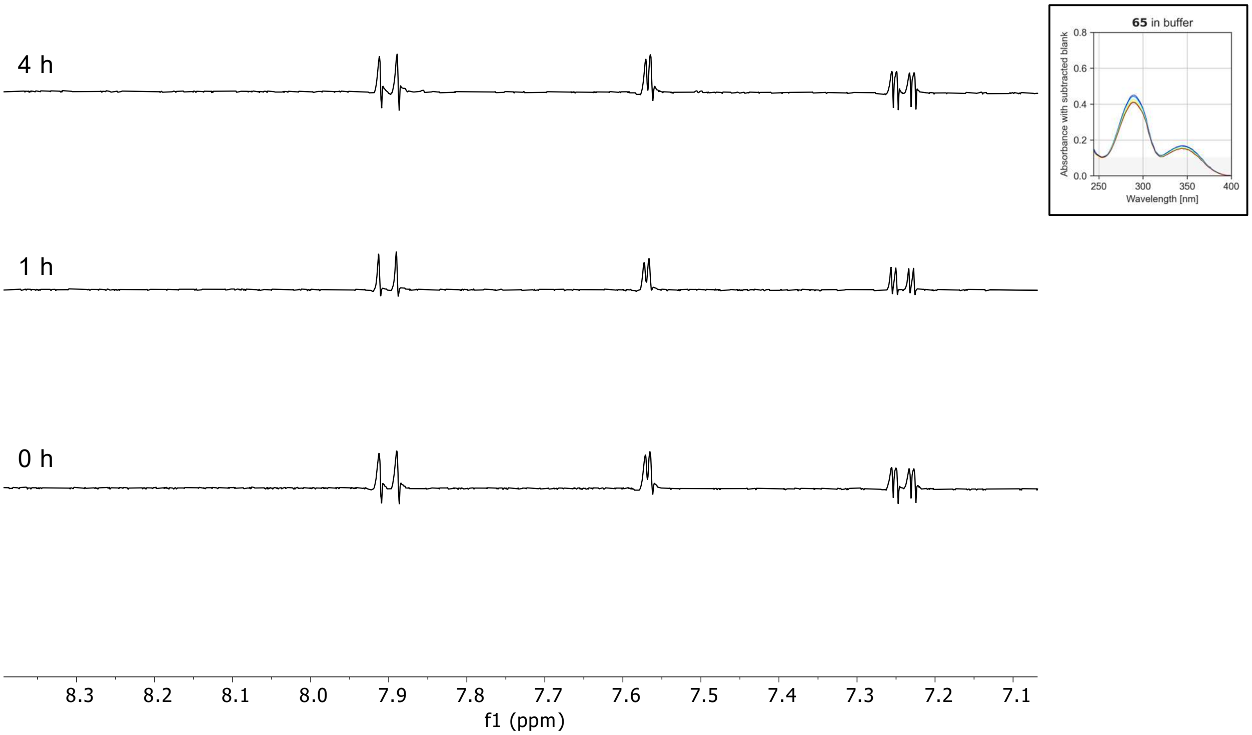

Figure S5. NMR-based stability assay for compound **65** along with time-dependent absorbance spectra from the UV-Vis-based stability assay (right).

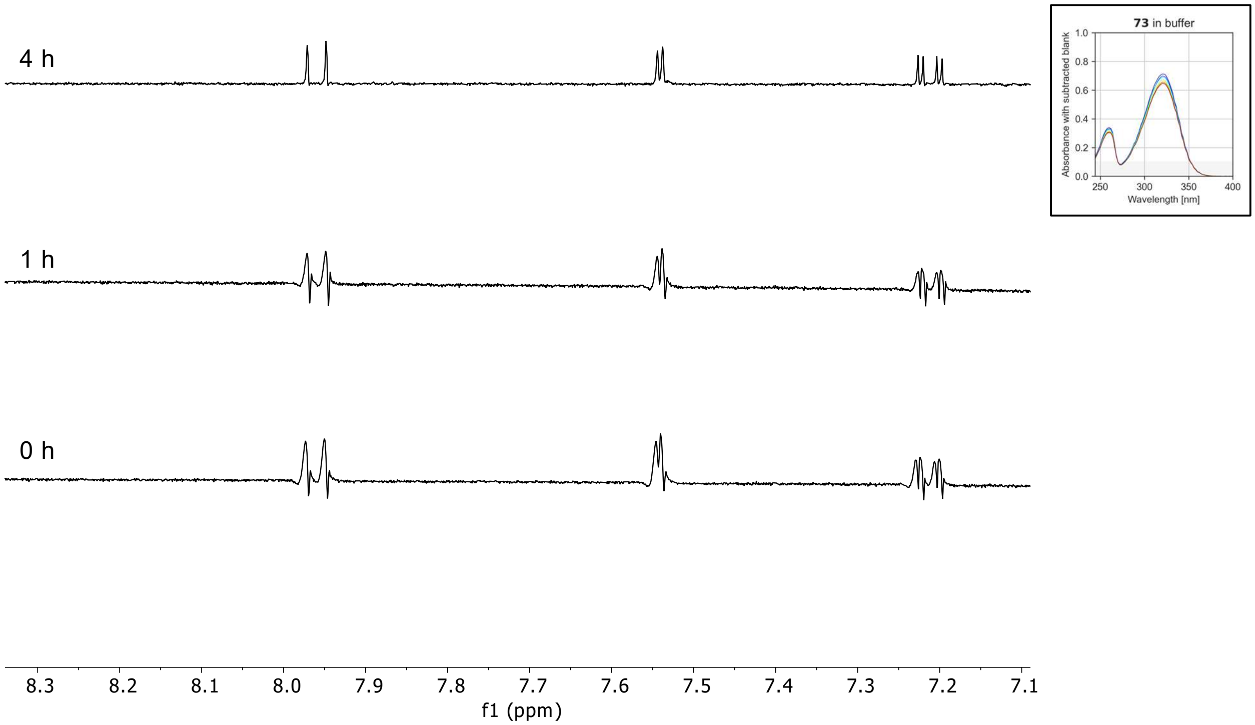

Figure S6. NMR-based stability assay for compound **73** along with time-dependent absorbance spectra from the UV-Vis-based stability assay (right).

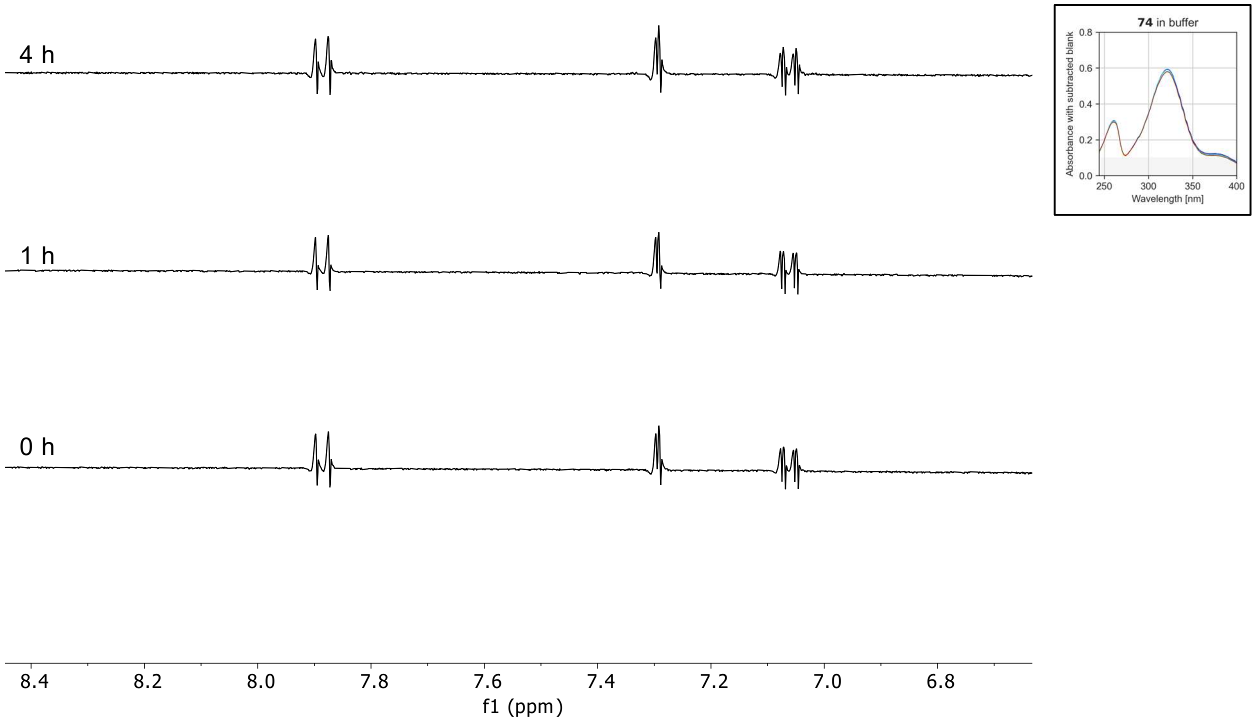

Figure S7. NMR-based stability assay for compound **74** along with time-dependent absorbance spectra from the UV-Vis-based stability assay (right).

Substituent \ Core	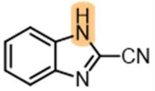 Benzimidazole	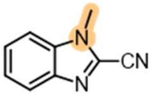 1-Methyl-benzimidazole	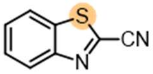 Benzothiazole	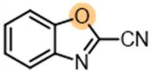 Benzoxazole
/	1	18	Binding to microplates	Binding to microplates
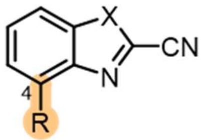 -NO$_2$	2	19	52	Unstable
-COOMe	3	20	53	Unstable
-COOH	4	21	Not accessible	Not accessible
-Cl	5	22	55	Binding to microplates
-Me	6	23	Binding to microplates	Unstable in solid
-OMe	7	24	57	90
-OH	8	25	58	91
-NH$_2$	9	26	59	92
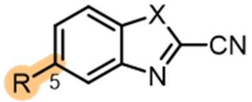 -NO$_2$	10	27	Binding to microplates	Not soluble
-COOMe	11	28	61	Unstable
-COOH	12	Unstable	62	Unstable
-Cl	13	30	Binding to microplates	Binding to microplates
-Me	14	31	Binding to microplates	Unstable in solid
-OMe	15	32	65	Binding to microplates
-OH	16	33	66	99
-NH$_2$	17	34	67	100
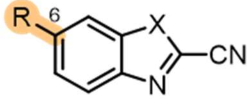 -NO$_2$		35	Unstable	Unstable
-COOMe		36	69	Unstable
-COOH		37	70	Unstable
-Cl		38	Binding to microplates	Binding to microplates
-Me		39	Binding to microplates	Low absorbance
-OMe		40	73	Binding to microplates
-OH		41	74	107
-NH$_2$		42	75	108
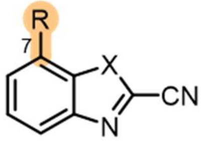 -NO$_2$		43	76	Unstable
-COOMe		44	77	Unstable
-COOH		45	78	Unstable
-Cl		46	Binding to microplates	Binding to microplates
-Me		47	Binding to microplates	Unstable in solid
-OMe		48	Binding to microplates	Unstable
-OH		49	82	115
-NH$_2$		50	83	116

Not reactive
Reactive
Hyperreactive

Figure S8. UV-Vis-based reactivity assay with L-Lys.

Core / Substituent		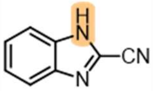 Benzimidazole	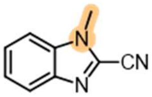 1-Methyl-benzimidazole	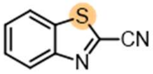 Benzothiazole	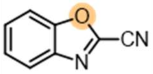 Benzoxazole
	/	1	18	Binding to microplates	Binding to microplates
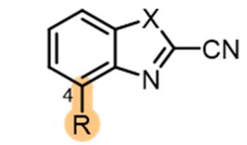	-NO_2	2	19	52	Unstable
	-COOMe	3	20	53	Unstable
	-COOH	4	21	Not accessible	Not accessible
	-Cl	5	22	55	Binding to microplates
	-Me	6	23	Binding to microplates	Unstable in solid
	-OMe	7	24	57	90
	-OH	8	25	58	91
	-NH_2	9	26	59	92
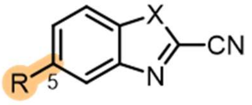	-NO_2	10	27	Binding to microplates	Not soluble
	-COOMe	11	28	61	Unstable
	-COOH	12	Unstable	62	Unstable
	-Cl	13	30	Binding to microplates	Binding to microplates
	-Me	14	31	Binding to microplates	Unstable in solid
	-OMe	15	32	65	Binding to microplates
	-OH	16	33	66	99
	-NH_2	17	34	67	100
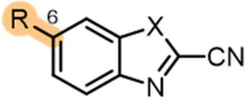	-NO_2		35	Unstable	Unstable
	-COOMe		36	69	Unstable
	-COOH		37	70	Unstable
	-Cl		38	Binding to microplates	Binding to microplates
	-Me		39	Binding to microplates	Low absorbance
	-OMe		40	73	Binding to microplates
	-OH		41	74	107
	-NH_2		42	75	108
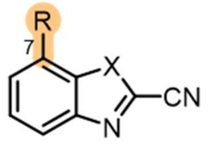	-NO_2		43	76	Unstable
	-COOMe		44	77	Unstable
	-COOH		45	78	Unstable
	-Cl		46	Binding to microplates	Binding to microplates
	-Me		47	Binding to microplates	Unstable in solid
	-OMe		48	Binding to microplates	Unstable
	-OH		49	82	115
	-NH_2		50	83	116
Not reactive					
Reactive					
Hyperreactive					

Figure S9. UV-Vis-based reactivity assay with L-Ser.

Substituent \ Core		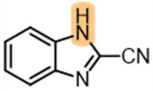 Benzimidazole	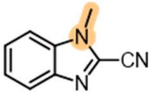 1-Methyl-benzimidazole	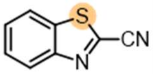 Benzothiazole	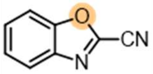 Benzoxazole
	/	1	18	Binding to microplates	Binding to microplates
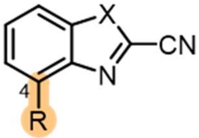	-NO_2	2	19	52	Unstable
	-COOMe	3	20	53	Unstable
	-COOH	4	21	Not accessible	Not accessible
	-Cl	5	22	55	Binding to microplates
	-Me	6	23	Binding to microplates	Unstable in solid
	-OMe	7	24	57	90
	-OH	8	25	58	91
	-NH_2	9	26	59	92
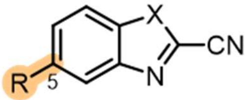	-NO_2	10	27	Binding to microplates	Not soluble
	-COOMe	11	28	61	Unstable
	-COOH	12	Unstable	62	Unstable
	-Cl	13	30	Binding to microplates	Binding to microplates
	-Me	14	31	Binding to microplates	Unstable in solid
	-OMe	15	32	65	Binding to microplates
	-OH	16	33	66	99
	-NH_2	17	34	67	100
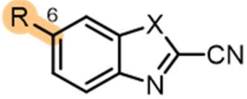	-NO_2		35	Unstable	Unstable
	-COOMe		36	69	Unstable
	-COOH		37	70	Unstable
	-Cl		38	Binding to microplates	Binding to microplates
	-Me		39	Binding to microplates	Low absorbance
	-OMe		40	73	Binding to microplates
	-OH		41	74	107
	-NH_2		42	75	108
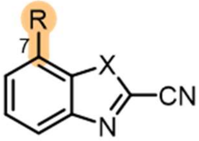	-NO_2		43	76	Unstable
	-COOMe		44	77	Unstable
	-COOH		45	78	Unstable
	-Cl		46	Binding to microplates	Binding to microplates
	-Me		47	Binding to microplates	Unstable in solid
	-OMe		48	Binding to microplates	Unstable
	-OH		49	82	115
	-NH_2		50	83	116
Not reactive					
Reactive					
Hyperreactive					

Figure S10. UV-Vis-based reactivity assay with TCEP. The result for **21** is false positive, based on an LCMS experiment, where **21** was classified as not reactive (see **Figure S15**).

Substituent \ Core		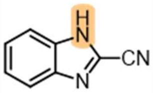 Benzimidazole	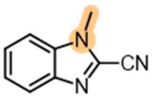 1-Methyl-benzimidazole	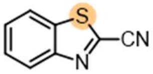 Benzothiazole	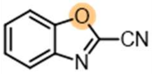 Benzoxazole
	/	1	18	Binding to microplates	Binding to microplates
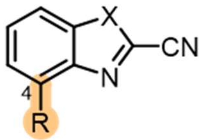	-NO_2	2	19	52	Unstable
	-COOMe	3	20	53	Unstable
	-COOH	4	21	Not accessible	Not accessible
	-Cl	5	22	55	Binding to microplates
	-Me	6	23	Binding to microplates	Unstable in solid
	-OMe	7	24	57	90
	-OH	8	25	58	91
	-NH_2	9	26	59	92
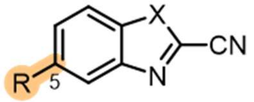	-NO_2	10	27	Binding to microplates	Not soluble
	-COOMe	11	28	61	Unstable
	-COOH	12	Unstable	62	Unstable
	-Cl	13	30	Binding to microplates	Binding to microplates
	-Me	14	31	Binding to microplates	Unstable in solid
	-OMe	15	32	65	Binding to microplates
	-OH	16	33	66	99
	-NH_2	17	34	67	100
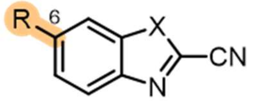	-NO_2		35	Unstable	Unstable
	-COOMe		36	69	Unstable
	-COOH		37	70	Unstable
	-Cl		38	Binding to microplates	Binding to microplates
	-Me		39	Binding to microplates	Low absorbance
	-OMe		40	73	Binding to microplates
	-OH		41	74	107
	-NH_2		42	75	108
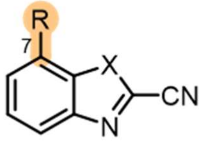	-NO_2		43	76	Unstable
	-COOMe		44	77	Unstable
	-COOH		45	78	Unstable
	-Cl		46	Binding to microplates	Binding to microplates
	-Me		47	Binding to microplates	Unstable in solid
	-OMe		48	Binding to microplates	Unstable
	-OH		49	82	115
	-NH_2		50	83	116

Not reactive
Reactive
Hyperreactive

Figure S11. UV-Vis-based reactivity assay with NAC.

Core / Substituent		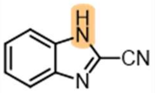 Benzimidazole	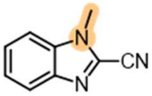 1-Methyl-benzimidazole	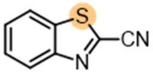 Benzothiazole	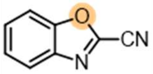 Benzoxazole
	/	1	18	Binding to microplates	Binding to microplates
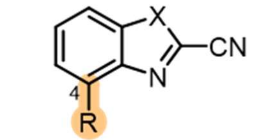	$-NO_2$	2	19	52	Unstable
	-COOMe	3	20	53	Unstable
	-COOH	4	21	Not accessible	Not accessible
	-Cl	5	22	55	Binding to microplates
	-Me	6	23	Binding to microplates	Unstable in solid
	-OMe	7	24	57	90
	-OH	8	25	58	91
	$-NH_2$	9	26	59	92
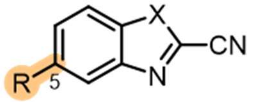	$-NO_2$	10	27	Binding to microplates	Not soluble
	-COOMe	11	28	61	Unstable
	-COOH	12	Unstable	62	Unstable
	-Cl	13	30	Binding to microplates	Binding to microplates
	-Me	14	31	Binding to microplates	Unstable in solid
	-OMe	15	32	65	Binding to microplates
	-OH	16	33	66	99
	$-NH_2$	17	34	67	100
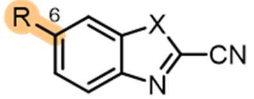	$-NO_2$		35	Unstable	Unstable
	-COOMe		36	69	Unstable
	-COOH		37	70	Unstable
	-Cl		38	Binding to microplates	Binding to microplates
	-Me		39	Binding to microplates	Low absorbance
	-OMe		40	73	Binding to microplates
	-OH		41	74	107
	$-NH_2$		42	75	108
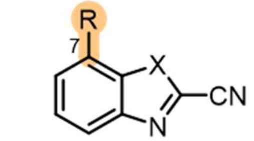	$-NO_2$		43	76	Unstable
	-COOMe		44	77	Unstable
	-COOH		45	78	Unstable
	-Cl		46	Binding to microplates	Binding to microplates
	-Me		47	Binding to microplates	Unstable in solid
	-OMe		48	Binding to microplates	Unstable
	-OH		49	82	115
	$-NH_2$		50	83	116
Not reactive					
Reactive					
Hyperreactive					

Figure S12. UV-Vis-based reactivity assay with L-Cys.

Substituent \ Core		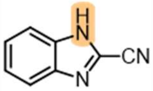 Benzimidazole	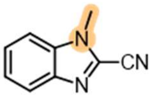 1-Methyl-benzimidazole	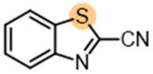 Benzothiazole	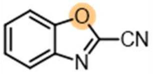 Benzoxazole
	/	1	18	Binding to microplates	Binding to microplates
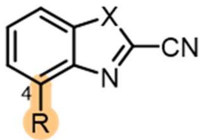	$-NO_2$	2	19	52	Unstable
	-COOMe	3	20	53	Unstable
	-COOH	4	21	Not accessible	Not accessible
	-Cl	5	22	55	Binding to microplates
	-Me	6	23	Binding to microplates	Unstable in solid
	-OMe	7	24	57	90
	-OH	8	25	58	91
	$-NH_2$	9	26	59	92
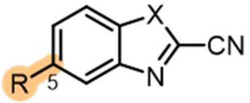	$-NO_2$	10	27	Binding to microplates	Not soluble
	-COOMe	11	28	61	Unstable
	-COOH	12	Unstable	62	Unstable
	-Cl	13	30	Binding to microplates	Binding to microplates
	-Me	14	31	Binding to microplates	Unstable in solid
	-OMe	15	32	65	Binding to microplates
	-OH	16	33	66	99
	$-NH_2$	17	34	67	100
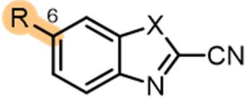	$-NO_2$		35	Unstable	Unstable
	-COOMe		36	69	Unstable
	-COOH		37	70	Unstable
	-Cl		38	Binding to microplates	Binding to microplates
	-Me		39	Binding to microplates	Low absorbance
	-OMe		40	73	Binding to microplates
	-OH		41	74	107
	$-NH_2$		42	75	108
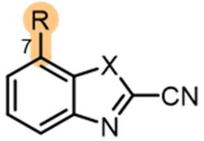	$-NO_2$		43	76	Unstable
	-COOMe		44	77	Unstable
	-COOH		45	78	Unstable
	-Cl		46	Binding to microplates	Binding to microplates
	-Me		47	Binding to microplates	Unstable in solid
	-OMe		48	Binding to microplates	Unstable
	-OH		49	82	115
	$-NH_2$		50	83	116
Not reactive					
Reactive					

Figure S13. DTNB thiol reactivity assay.

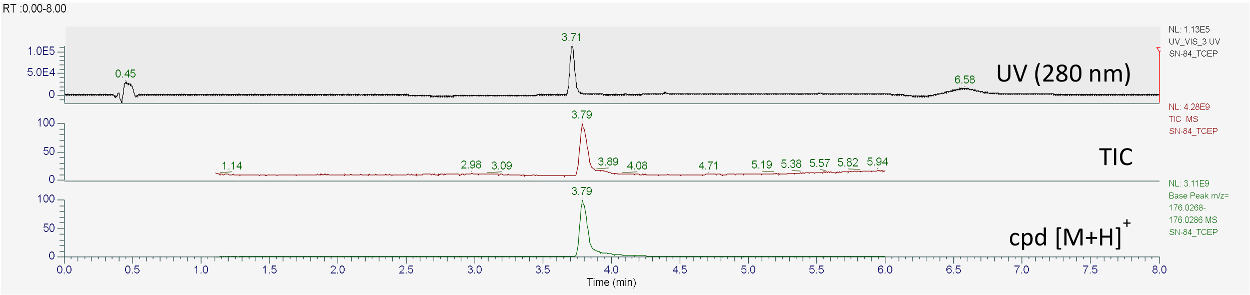

Figure S14. LCMS chromatograms for **75** in presence of TCEP (5 eq.). Besides the main compound peak (3.71 min), no additional peaks appeared at 280 nm.

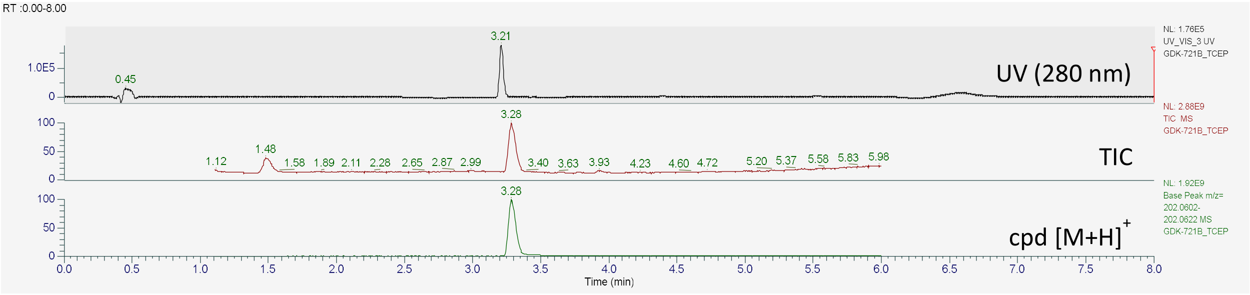

Figure S15. LCMS chromatograms for **21** in presence of TCEP (5 eq.). Besides the main compound peak (3.21 min), no additional peaks appeared at 280 nm.

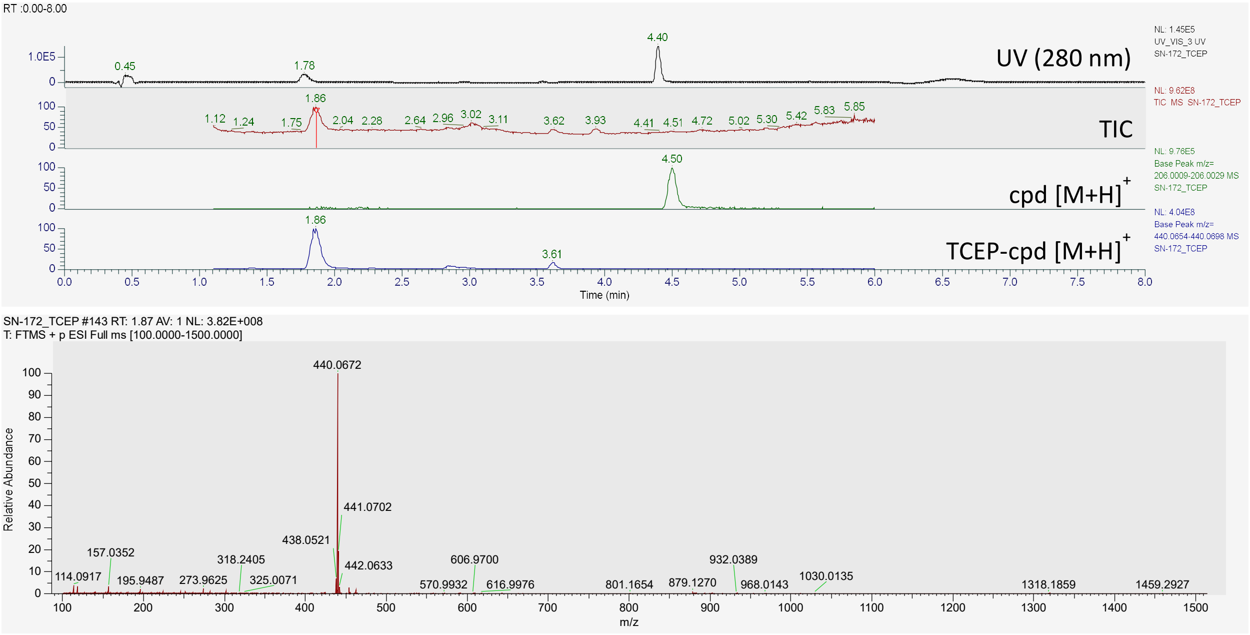

Figure S16. LCMS chromatograms at 280 nm for **76** in presence of TCEP (5 eq.). Besides the main compound peak (4.40 min), an additional peak appeared at 280 nm (1.78 min) with m/z = 440.0672 (mass spectrum below), corresponding to **76**–TCEP adduct $[M+H]^+$ with m/z = 440.0676 (-0.9 ppm) and chemical formula $C_{17}H_{19}N_3O_7PS^+$.

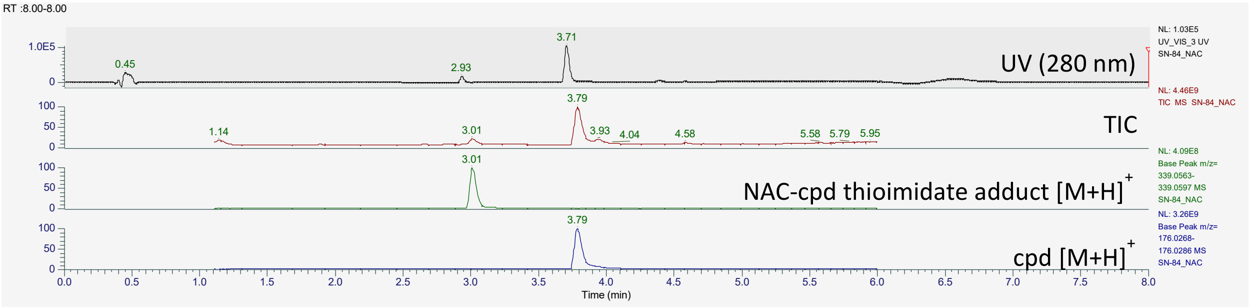

Figure S17. LCMS chromatograms at 280 nm for **75** in presence of NAC (5 eq.). Besides the main compound peak (3.71 min), an additional peak appeared at 280 nm (2.93 min), corresponding to **75**–NAC thioimidate adduct $[M+H]^+$ with m/z = 339.0580 (-0.3 ppm).

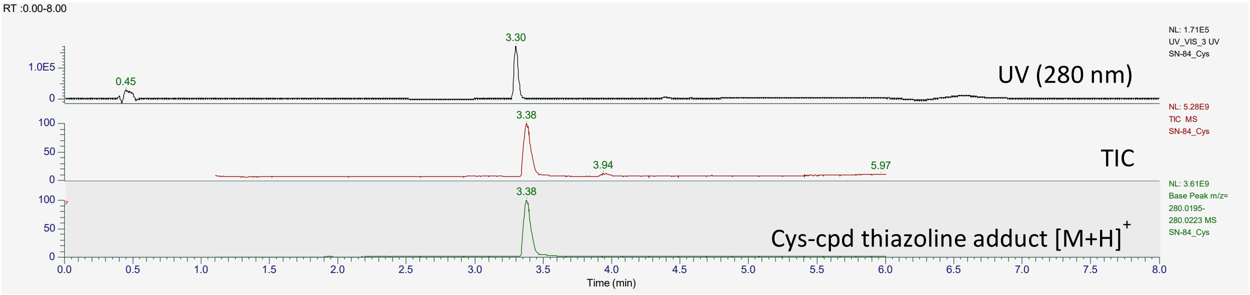

Figure S18. LCMS chromatograms at 280 nm for **75** in presence of Cys (5 eq.). A single peak appeared at 280 nm (3.30 min), corresponding to **75**–Cys thiazoline adduct.

Table S3. Second-order reaction rate constants (k_2) with Cys, obtained from two different methods and compared to the literature data.

Compound	k_2 with Cys [s^{-1} M^{-1}]		
	From a single Cys concentration [a]	From multiple Cys concentrations (mean ± SEM) [a]	Literature data [b]
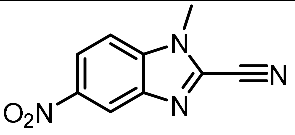 **27**	3.8	2.5 ± 0.2	/
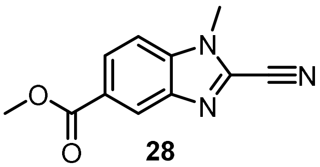 **28**	1.5	1.1 ± 0.1	/
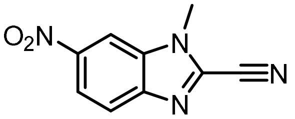 **35**	4.8	2.97 ± 0.01	/
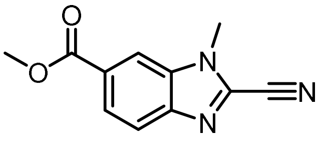 **36**	1.7	1.19 ± 0.02	/
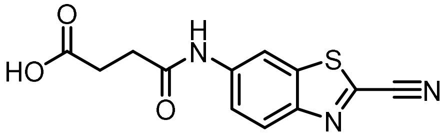 **117**	NA [c]	12.8 ± 1.6	9.19 [2]<br>2.9 [3]

[a] Determined at 37 °C

[b] Determined at room temperature (23 °C) with an HPLC method

[c] Not available due to high reactivity

Benzimidazoles

Position / Substituent	4	5	Mean
$-NO_2$	0.50	0.49	0.50
-COOMe	1.88	1.00	1.44
-COOH	1.11	1.66	1.39
-Cl	0.51	1.42	0.97
-Me	0.83	1.40	1.12
-OMe	1.03	1.20	1.12
-OH	1.16	1.26	1.21
$-NH_2$	1.10	1.12	1.11
**Mean**	1.0	1.2	1.11

1-Methylbenzimidazoles

Position / Substituent	4	5	6	7	Mean
$-NO_2$	2.23	3.78	4.76	1.53	3.08
-COOMe	0.60	1.53	1.19	0.71	1.01
-COOH	0.23	Unstable	0.74	0.33	0.44
-Cl	0.78	1.01	0.89	0.73	0.85
-Me	0.32	0.42	0.45	0.29	0.37
-OMe	0.41	0.46	0.36	0.38	0.40
-OH	0.37	0.44	0.33	0.35	0.37
$-NH_2$	0.34	0.29	0.29	0.40	0.33
**Mean**	0.7	1.1	1.1	0.6	0.87

Benzothiazoles

Position / Substituent	4	5	6	7	Mean
$-NO_2$	31.2	Binding to microplates	Unstable	Reacts with TCEP	31.2
-COOMe	13.8	31.3	26.8	17.5	22.4
-COOH	Not accessible	19.4	21.7	7.1	16.1
-Cl	Reacts with NAcCys	Binding to microplates	Binding to microplates	Binding to microplates	
-Me	Binding to microplates	Binding to microplates	Binding to microplates	Binding to microplates	
-OMe	9.3	14.1	9.9	Binding to microplates	11.1
-OH	10.1	12.6	7.2	9.9	10.0
$-NH_2$	7.9	9.5	5.7	18.1	10.3
**Mean**	14.5	17.4	14.3	13.1	14.9

Benzoxazoles

Position / Substituent	4	5	6	7	Mean
$-NO_2$	Unstable	Not soluble	Unstable	Unstable	
-COOMe	Unstable	Unstable	Unstable	Unstable	
-COOH	Not accessible	Unstable	Unstable	Unstable	
-Cl	Binding to microplates	Binding to microplates	Binding to microplates	Binding to microplates	
-Me	Unstable	Unstable in solid	Low absorbance	Unstable in solid	
-OMe	Reacts with NAcCys	Binding to microplates	Binding to microplates	Unstable	
-OH	28.3	Reacts with NAcCys	Reacts with NAcCys	Reacts with NAcCys	28.3
$-NH_2$	Reacts with NAcCys	Reacts with NAcCys	188.4	49.3	118.9
**Mean**	28.3		188.4	49.3	88.7

Figure S19. Second-order reaction rate constants (k_2 in s^{-1} M^{-1}) for reaction with Cys at 37 °C.

Table S4. Results from labeling oligopeptides (**UP1**, CGKGCGSGYGW; **UP2**, AGKGCGSGYGW). Exact masses for ions of adducts between compounds and oligopeptides and parent compounds that were detected are shaded. The conversion of **UP1** and **UP2** was calculated by comparing the AUCs of parent oligopeptide peaks at 280 nm with or without added compound. Selectivity was calculated as a ratio between **UP1** and **UP2** conversion. Substituents with derivatization capability are amino, hydroxyl, and carboxylic acid. The global score accounts for reactivity, selectivity, and derivatization capability. Chromatograms are provided in Supporting Information, Oligopeptide labeling.

Cpd	Core	Substitution	k_2 with Cys [M^{-1} s^{-1}]	m/z cpd $[M+H]^+$	m/z UP1-disulfide $[M+2H]^{2+}$	m/z UP1 $[M+2H]^{2+}$	m/z UP1-cpd $[M+2H]^{2+}$ (mono adduct)	m/z UP1-cpd_2 $[M+2H]^{2+}$ (di adduct)	m/z UP1-cpd_3 $[M+2H]^{2+}$ (tri adduct)	m/z UP1-cpd_3 $[M+NH_3+2H]^{2+}$ (tri adduct)	m/z cpd $[M+H]^+$	m/z UP2 $[M+2H]^{2+}$	m/z UP2-cpd $[M+2H]^{2+}$ (mono adduct)	m/z UP2-cpd_2 $[M+2H]^{2+}$ (di adduct)	m/z UP1-cpd_2 $[M+NH_3+2H]^{2+}$ (di adduct)	UP1 % conversion	UP2 % conversion	Selectivity	Derivatization capability	Global score
4	benzimidazole	4-COOH	1.1	**188.0455**	**536.7024**	**537.7102**	**622.7161**	716.2351	809.7543	818.2675	**188.0455**	**521.7242**	615.2433	708.7624	717.2757	60%	0%	>60	1	2.3
12	benzimidazole	5-COOH	1.7	**188.0455**	**536.7024**	**537.7102**	**622.7161**	716.2351	809.7543	818.2675	**188.0455**	**521.7242**	615.2433	708.7624	717.2757	51%	0%	>51	1	2.3
21	1-methylbenzimidazole	4-COOH	0.2	**202.0611**	**536.7024**	**537.7102**	**629.7239**	730.2507	830.7777	839.2909	**202.0611**	**521.7242**	622.2511	722.7780	731.2913	72%	2%	45	1	1.9
27	1-methylbenzimidazole	5-NO2	2.5	**203.0564**	536.7024	**537.7102**	**630.2215**	**731.2460**	832.2707	840.7839	**203.0564**	**521.7242**	622.7487	723.7733	732.2866	70%	0%	>70	0	1.5
35	1-methylbenzimidazole	6-NO2	3.0	**203.0564**	536.7024	**537.7102**	**630.2215**	731.2460	832.2707	840.7839	**203.0564**	**521.7242**	622.7487	723.7733	732.2866	71%	7%	10	0	1.0
37	1-methylbenzimidazole	6-COOH	0.7	**202.0611**	536.7024	**537.7102**	**629.7239**	730.2507	830.7777	839.2909	**202.0611**	**521.7242**	622.2511	722.7780	731.2913	28%	3%	10	1	1.7
43	1-methylbenzimidazole	7-NO2	1.5	**203.0564**	**536.7024**	**537.7102**	**630.2215**	731.2460	832.2707	840.7839	**203.0564**	**521.7242**	622.7487	723.7733	732.2866	41%	0%	>41	0	1.3
45	1-methylbenzimidazole	7-COOH	0.3	**202.0611**	**536.7024**	**537.7102**	**629.7239**	730.2507	830.7777	839.2909	**202.0611**	**521.7242**	622.2511	722.7780	731.2913	57%	2%	31	1	1.8
52	benzothiazole	4-NO2	31.2	**206.0019**	536.7024	537.7102	**631.6943**	**734.1916**	**836.6889**	845.2021	**206.0019**	**521.7242**	**624.2215**	**726.7188**	735.2321	100%	26%	4	0	1.3
53	benzothiazole	4-COOMe	13.8	**219.0223**	536.7024	537.7102	**638.2045**	**747.2119**	856.2195	864.7327	**219.0223**	**521.7242**	**630.7317**	739.7392	748.2525	100%	13%	8	0	1.3
57	benzothiazole	4-OMe	9.3	**191.0274**	**536.7024**	537.7102	**624.2070**	**719.2170**	814.2272	822.7404	**191.0274**	**521.7242**	**616.7342**	711.7443	720.2576	100%	14%	7	0	1.2
58	benzothiazole	4-OH	10.1	**177.0117**	**536.7024**	537.7102	**617.1992**	**705.2013**	793.2036	801.7168	**177.0117**	**521.7242**	**609.7264**	697.7286	706.2419	100%	10%	10	1	2.3
59	benzothiazole	4-NH2	7.9	**176.0277**	**536.7024**	537.7102	**616.7072**	**704.2173**	791.7276	800.2408	**176.0277**	**521.7242**	**609.2344**	696.7446	705.2579	100%	9%	11	1	2.3
61	benzothiazole	5-COOMe	31.3	**219.0223**	536.7024	537.7102	**638.2045**	**747.2119**	856.2195	**864.7327**	**219.0223**	**521.7242**	**630.7317**	739.7392	748.2525	100%	26%	4	0	1.3
62	benzothiazole	5-COOH	19.4	**205.0066**	**536.7024**	537.7102	**631.1966**	**733.1962**	835.1960	843.7092	**205.0066**	**521.7242**	**623.7238**	725.7235	734.2368	100%	19%	5	1	2.2
65	benzothiazole	5-OMe	14.1	**191.0274**	536.7024	537.7102	**624.2070**	**719.2170**	814.2272	822.7404	**191.0274**	**521.7242**	**616.7342**	711.7443	720.2576	100%	13%	8	0	1.3
66	benzothiazole	5-OH	12.6	**177.0117**	536.7024	537.7102	**617.1992**	**705.2013**	793.2036	801.7168	**177.0117**	**521.7242**	**609.7264**	697.7286	706.2419	100%	14%	7	1	2.2
67	benzothiazole	5-NH2	9.5	**176.0277**	536.7024	537.7102	**616.7072**	**704.2173**	791.7276	800.2408	**176.0277**	**521.7242**	**609.2344**	696.7446	705.2579	100%	9%	12	1	2.3
69	benzothiazole	6-COOMe	26.8	**219.0223**	536.7024	537.7102	**638.2045**	**747.2119**	856.2195	**864.7327**	**219.0223**	**521.7242**	**630.7317**	739.7392	748.2525	100%	21%	5	0	1.3
70	benzothiazole	6-COOH	21.7	**205.0067**	536.7024	537.7102	**631.1967**	**733.1963**	835.1961	843.7093	**205.0067**	**521.7242**	**623.7239**	725.7236	734.2369	100%	15%	7	1	2.3
73	benzothiazole	6-OMe	9.9	**191.0274**	**536.7024**	537.7102	**624.2070**	**719.2170**	814.2272	822.7404	**191.0274**	**521.7242**	**616.7342**	711.7443	720.2576	100%	10%	10	0	1.3
74	benzothiazole	6-OH	7.2	**177.0117**	**536.7024**	537.7102	**617.1992**	**705.2013**	793.2036	801.7168	**177.0117**	**521.7242**	**609.7264**	697.7286	706.2419	100%	7%	15	1	2.3
75	benzothiazole	6-NH2	5.7	**176.0277**	536.7024	537.7102	**616.7072**	**704.2174**	791.7276	800.2408	**176.0277**	**521.7242**	**609.2344**	696.7446	705.2579	100%	10%	10	1	2.2
77	benzothiazole	7-COOMe	17.5	**219.0223**	536.7024	537.7102	**638.2045**	**747.2119**	856.2195	864.7327	**219.0223**	**521.7242**	**630.7317**	739.7392	748.2525	100%	19%	5	0	1.2
78	benzothiazole	7-COOH	7.1	**205.0066**	**536.7024**	537.7102	**631.1966**	**733.1962**	835.1960	843.7092	**205.0066**	**521.7242**	**623.7238**	725.7235	734.2368	100%	7%	14	1	2.3
82	benzothiazole	7-OH	9.9	**177.0117**	**536.7024**	537.7102	**617.1992**	**705.2013**	793.2036	801.7168	**177.0117**	**521.7242**	**609.7264**	697.7286	706.2419	100%	8%	12	1	2.3
83	benzothiazole	7-NH2	18.1	**176.0277**	**536.7024**	537.7102	**616.7072**	**704.2173**	791.7276	800.2408	**176.0277**	**521.7242**	**609.2344**	696.7446	705.2579	100%	16%	6	1	2.3
91	benzoxazole	4-OH	28.3	**161.0346**	536.7024	537.7102	**609.2106**	**689.2242**	769.2380	777.7512	**161.0346**	**521.7242**	**601.7378**	681.7515	690.2648	100%	36%	3	1	2.1
108	benzoxazole	6-NH2	188.4	**160.0506**	536.7024	537.7102	**608.7186**	**688.2402**	767.7620	776.2752	**160.0506**	**521.7242**	**601.2458**	**680.7675**	689.2808	100%	31%	3	1	2.6
116	benzoxazole	7-NH2	49.3	**160.0506**	536.7024	537.7102	**608.7186**	**688.2402**	767.7620	776.2752	**160.0506**	**521.7242**	**601.2458**	**680.7675**	689.2808	100%	45%	2	1	2.2

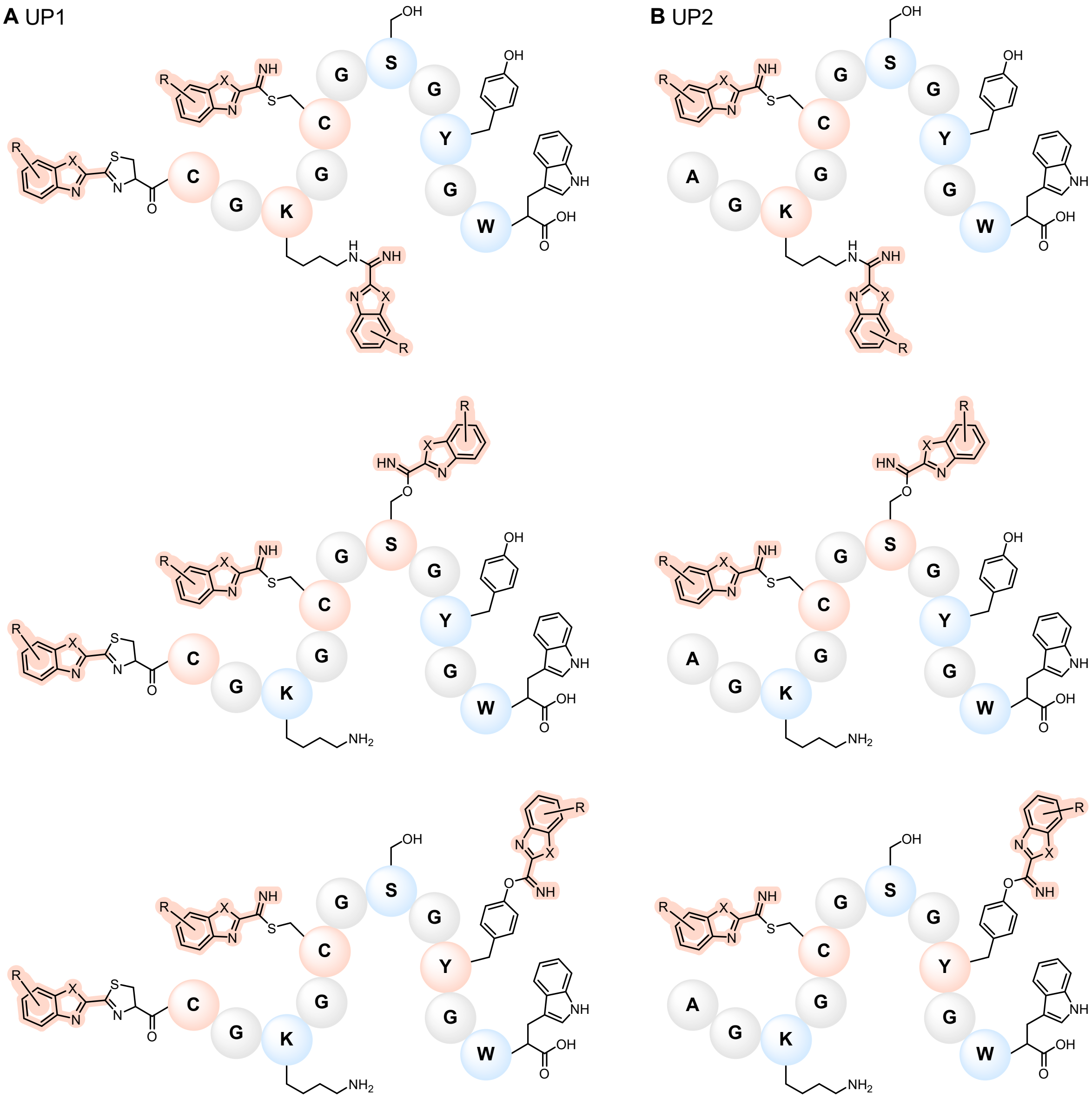

Figure S20. (**A**) Proposed **UP1**-cpd$_3$ triadducts. (**B**) Proposed **UP2**-cpd$_2$ diadducts. Labeling of nucleophilic residues other than Cys is possible, *i.e.*, Lys, Ser, and Tyr. **UP1**, CGKGCGSGYGW; **UP2**, AGKGCGSGYGW.

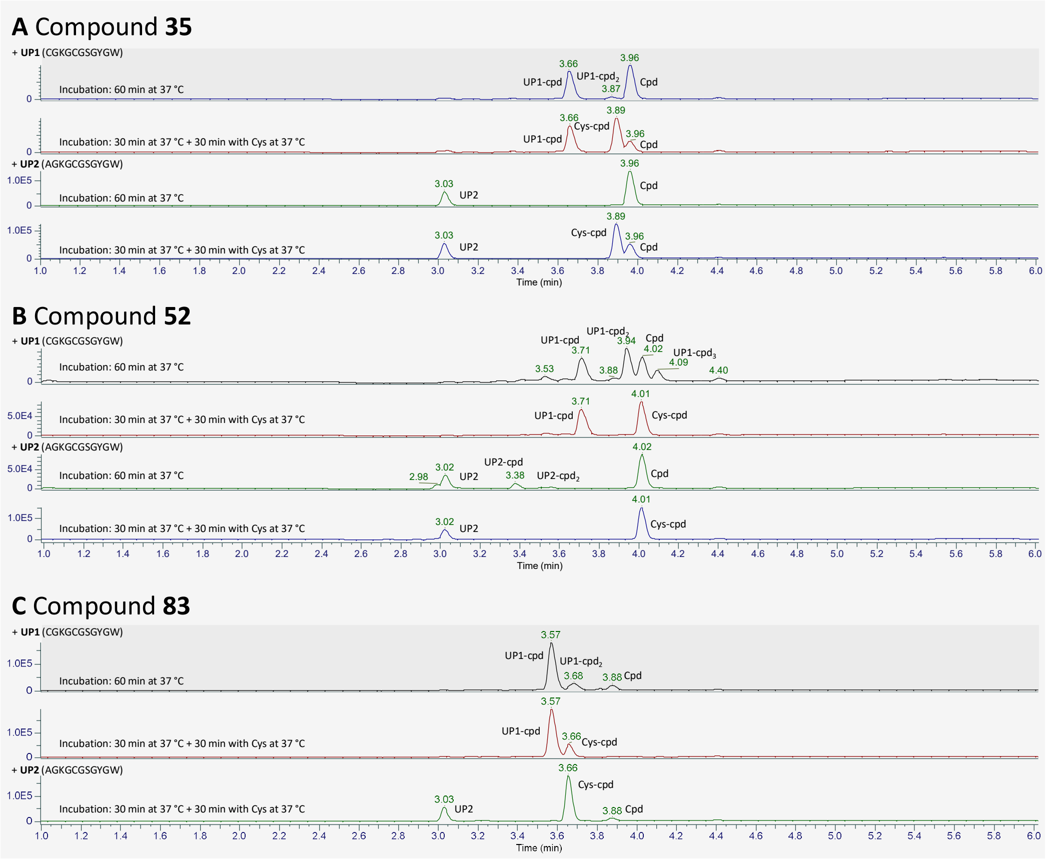

Figure S21. HPLC chromatograms at 280 nm for Cys treatment experiment with compounds **35**, **52**, and **83**. Species present in each peak were determined from the mass spectra (see Supporting Information, Oligopeptide labeling).

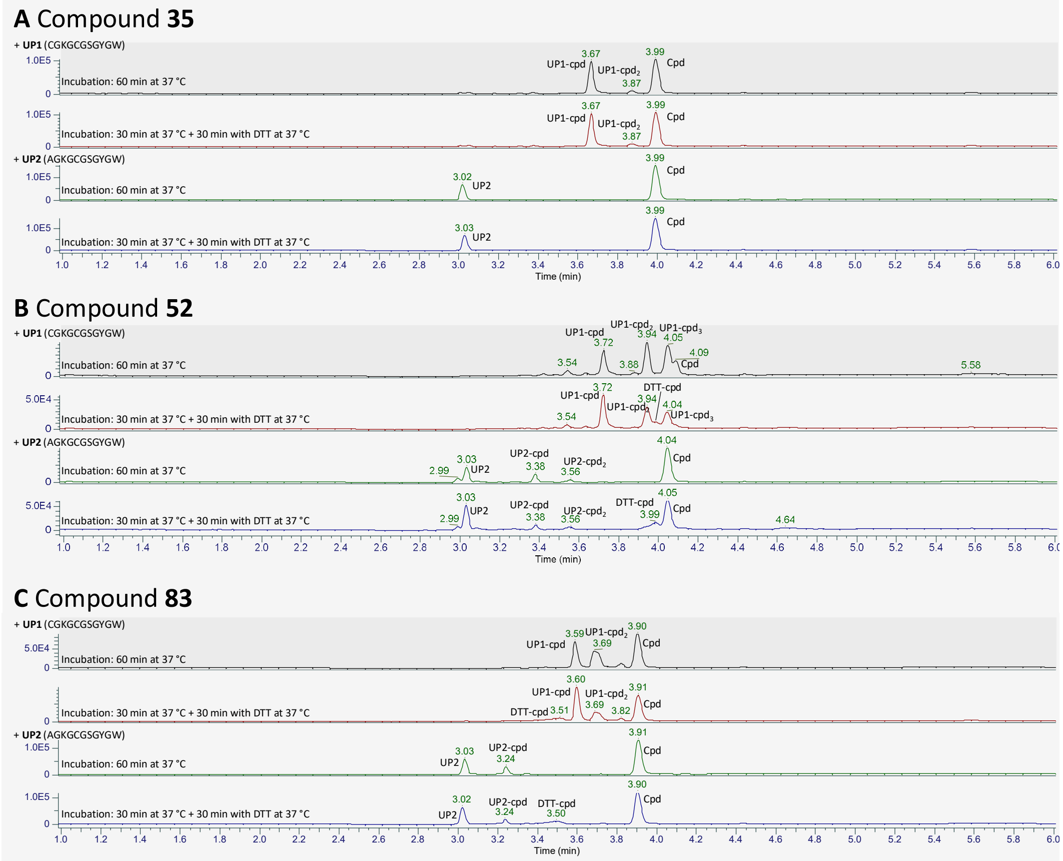

Figure S22. HPLC chromatograms at 280 nm for DTT treatment experiment with compounds **35**, **52**, and **83**. Species present in each peak were determined from the mass spectra (see Supporting Information, Oligopeptide labeling).

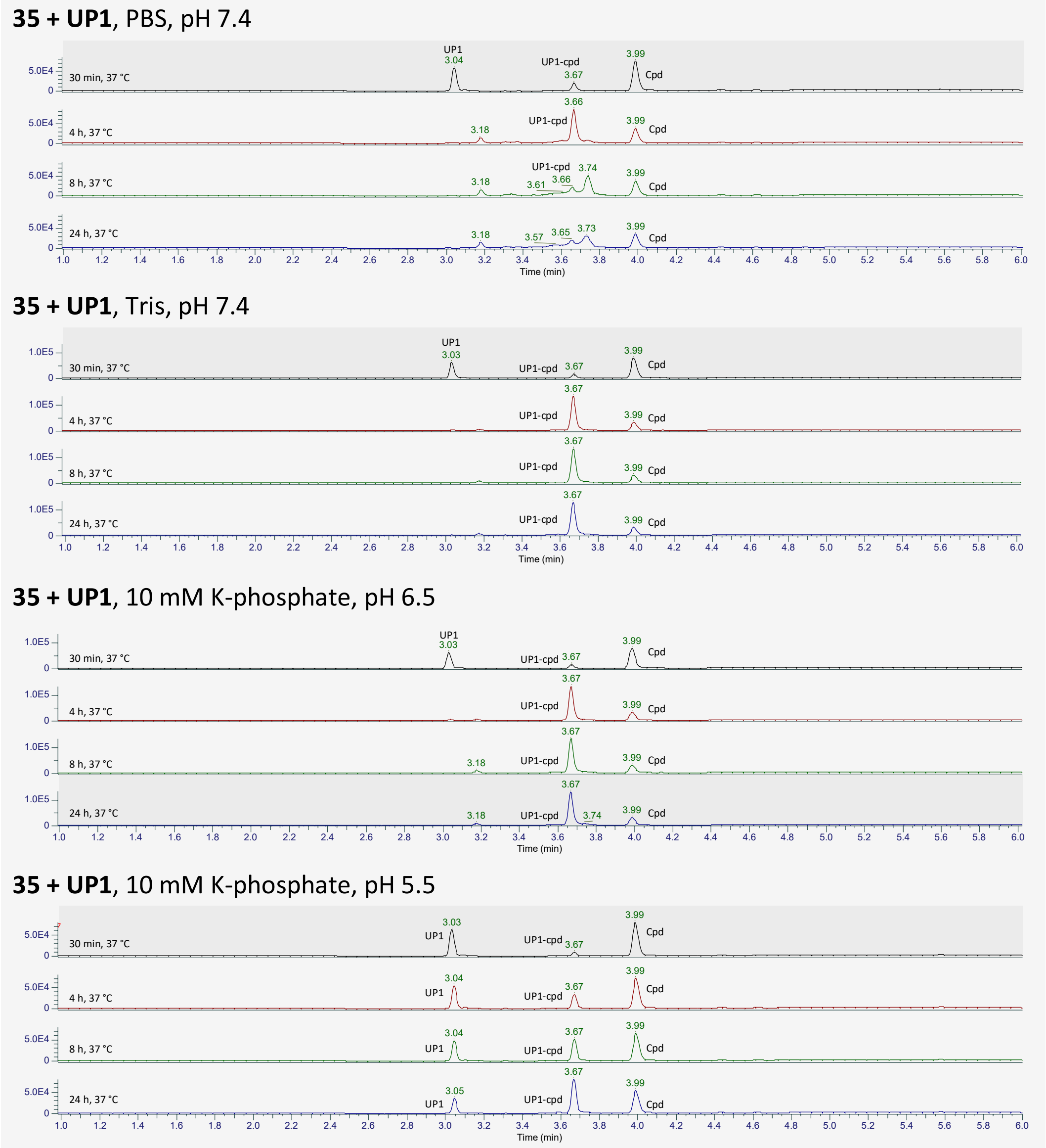

Figure S23. HPLC chromatograms at 280 nm for stability of adducts between **UP1** and **35**. Adduct is stable for at least 4 h in PBS, pH 7.4, and more than 24 h in Tris, pH 7.4, and phosphate buffer, pH 6.5 or 5.5. Species present in each peak were determined from the mass spectra.

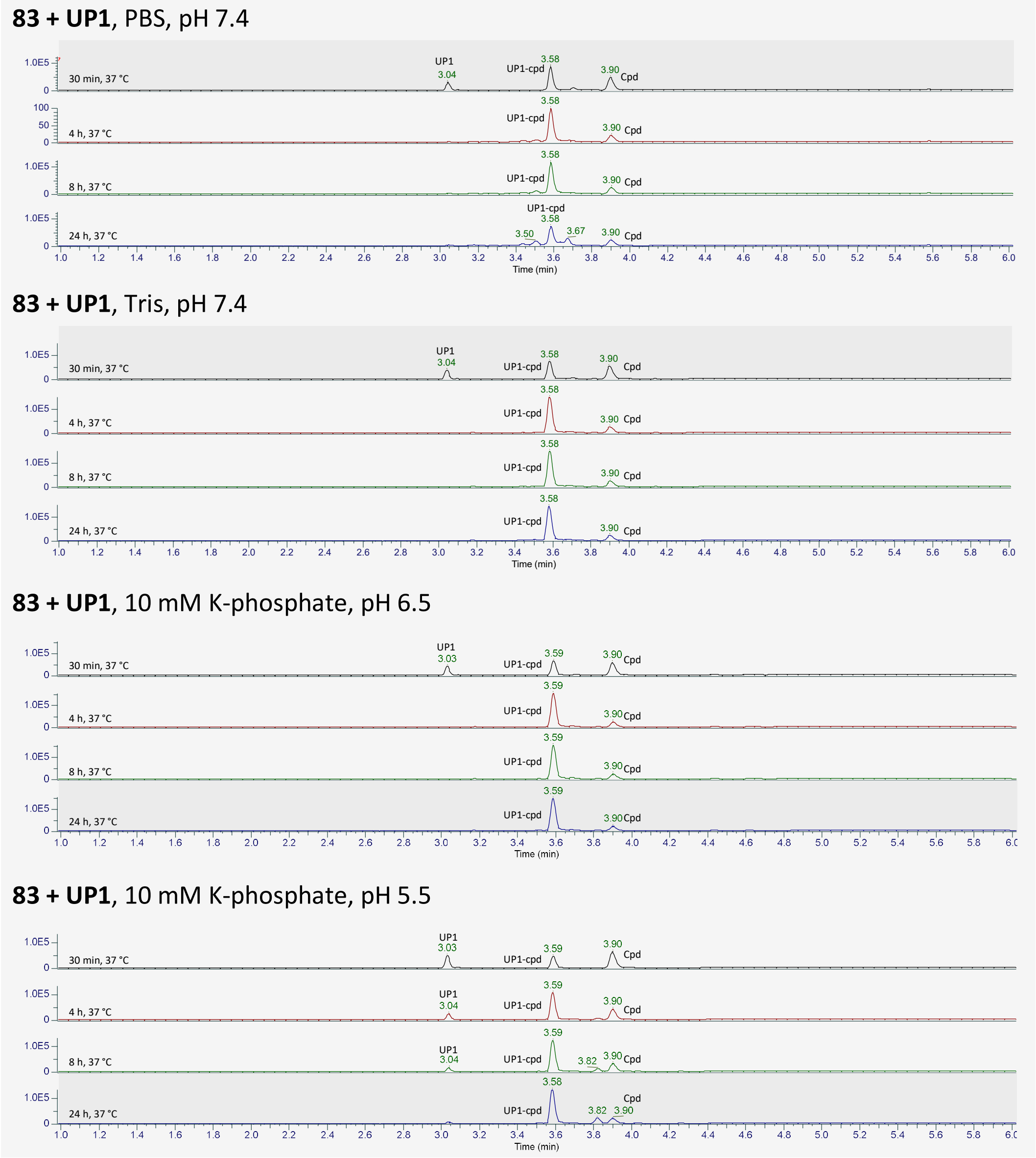

Figure S24. HPLC chromatograms at 280 nm for stability of adducts between **UP1** and **83**. Adduct is stable for at least 8 h in PBS, pH 7.4, and more than 24 h in Tris, pH 7.4, and phosphate buffer, pH 6.5 or 5.5. Species present in each peak were determined from the mass spectra.

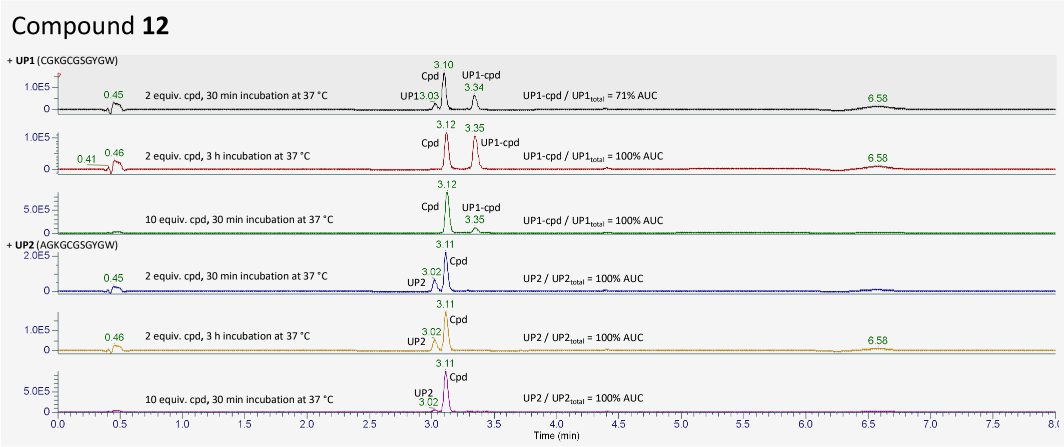

Figure S25. HPLC chromatograms at 280 nm comparing selectivity of oligopeptide labeling and **UP1** conversion for 2-cyanobenzimidazole **12** under different experimental conditions (10 *vs* 2 eq. of compound, 3 h *vs* 30 min incubation time). Species present in each peak were determined from the mass spectra (see Supporting Information, Oligopeptide labeling).

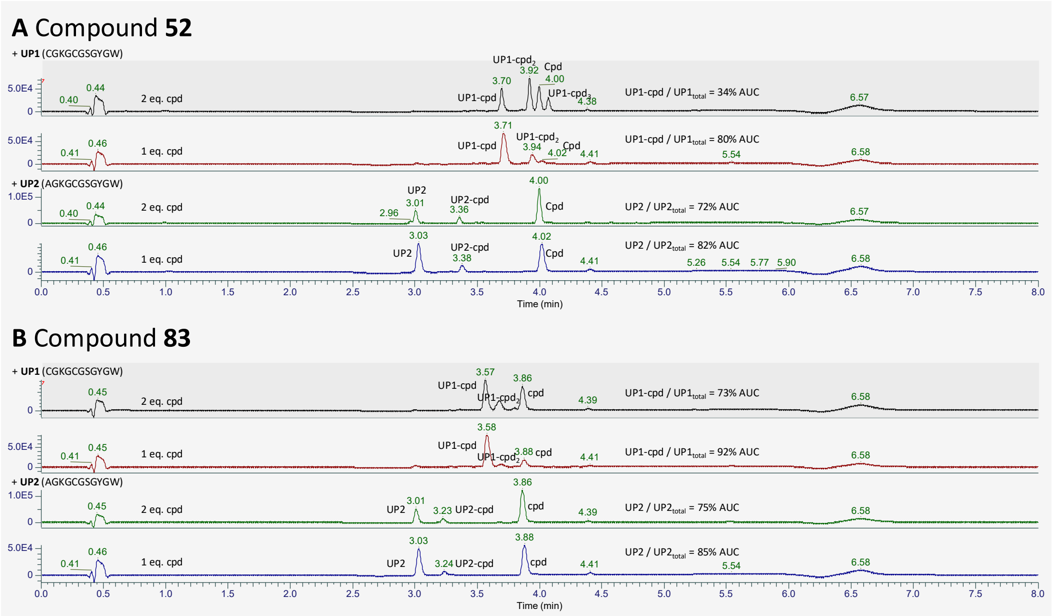

Figure S26. HPLC chromatograms at 280 nm comparing selectivity of oligopeptide labeling in presence of 1 or 2 eq. of 2-cyanobenzothiazoles after 30 min incubation at 37 °C. Species present in each peak were determined from the mass spectra (see Supporting Information, Oligopeptide labeling).

3 Stability and Reactivity Screening

Time-dependent absorbance spectra from the UV-Vis-based stability and reactivity assays. Unstable compounds were evaluated only in assay buffer. Stable and intermediately stable compounds were evaluated at six different conditions. Spectra are color-coded from 0 to 240 min (from blue to red). A black dashed line denotes compound background spectrum in buffer for the first timepoint. For compounds that were flagged as reactive in the UV-Vis-based screening assay with Cys, the timepoints obtained at the most responsive wavelength (denoted with red vertical line) were used to evaluate the rate of reaction with Cys.

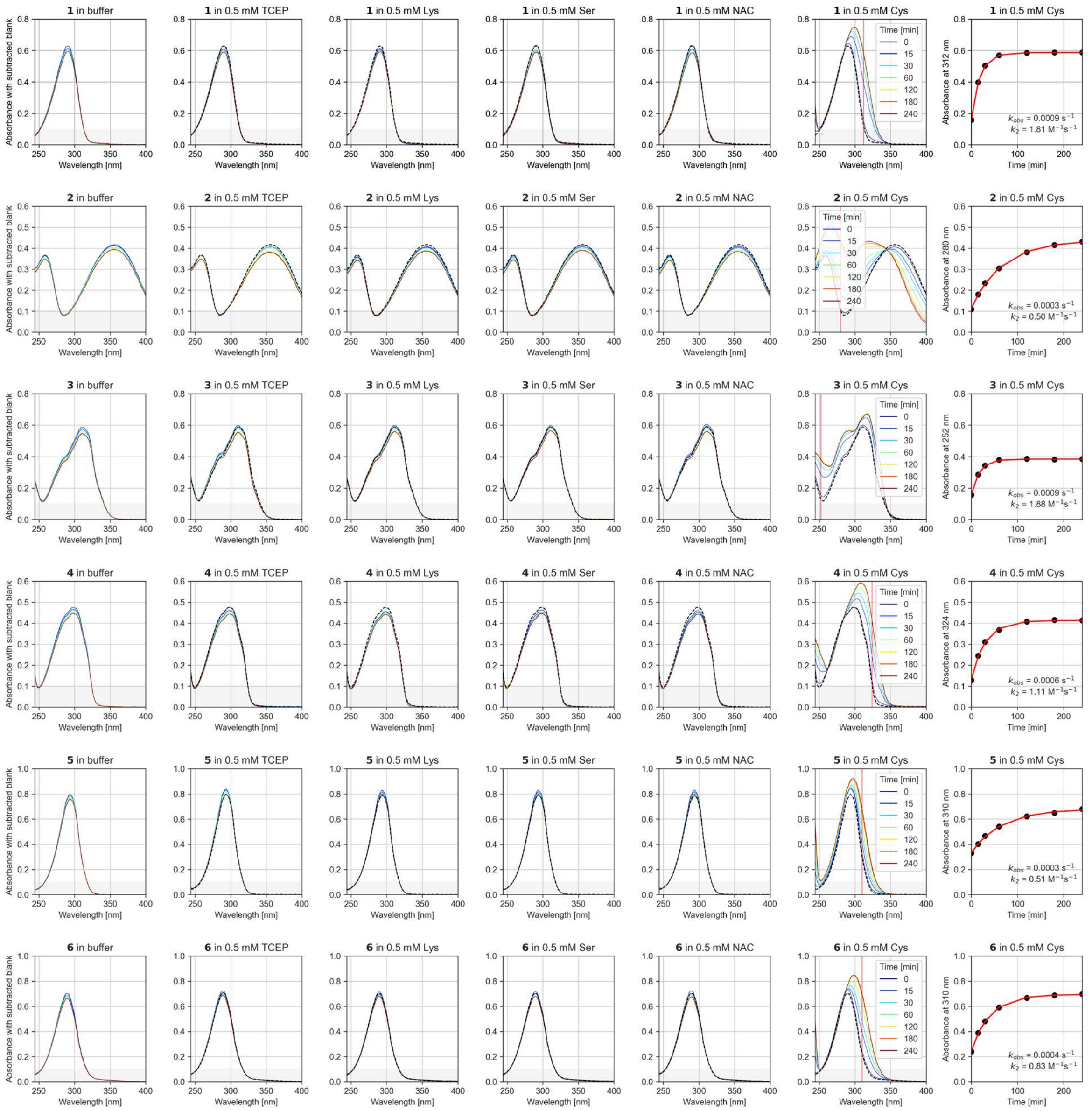

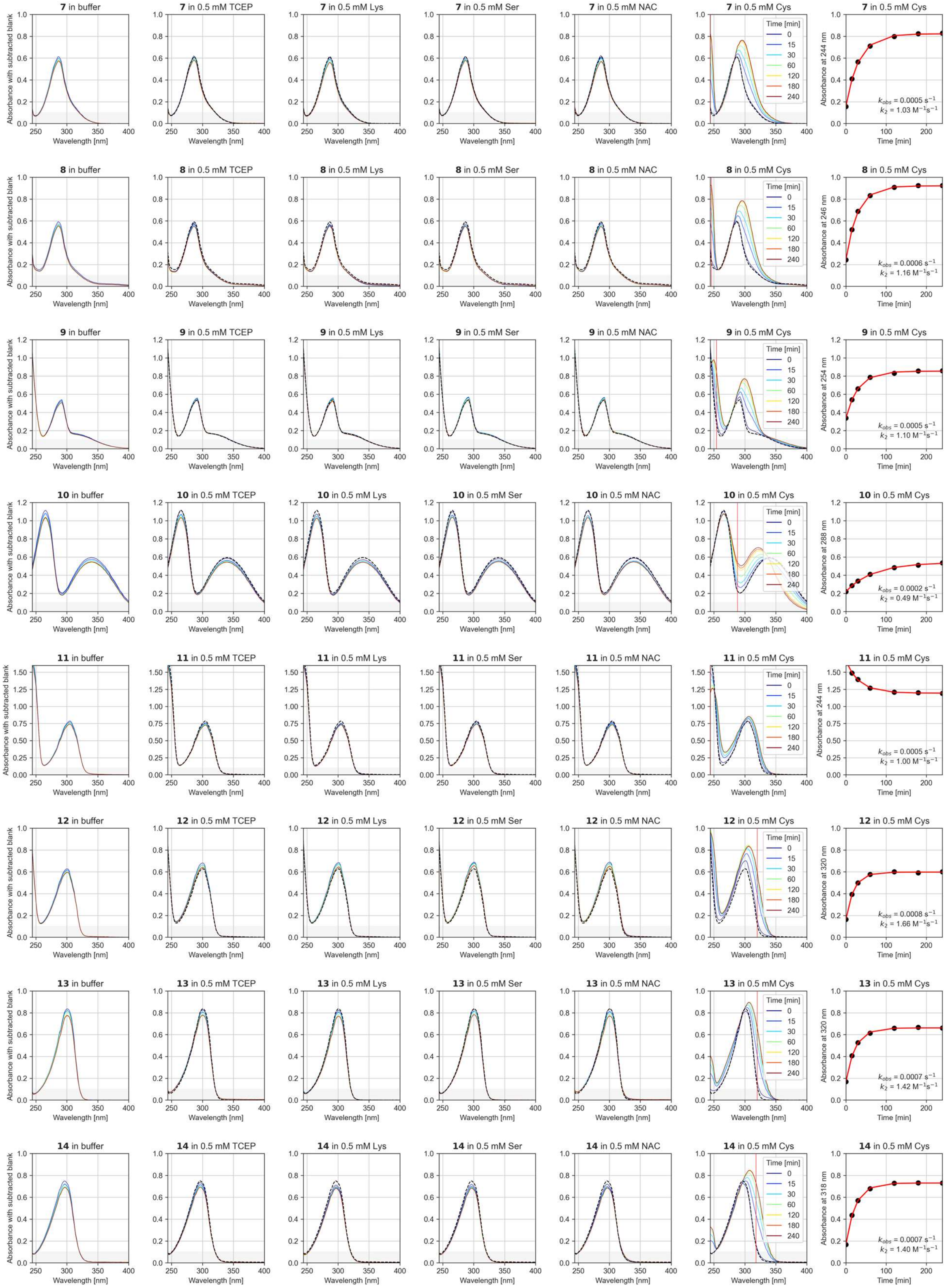
7 in buffer
7 in 0.5 mM TCEP
7 in 0.5 mM Lys
7 in 0.5 mM Ser
7 in 0.5 mM NAC
7 in 0.5 mM Cys
7 in 0.5 mM Cys
Absorbance at 244 nm
k_obs = 0.0005 s^-1
k_2 = 1.03 M^-1s^-1
8 in buffer
8 in 0.5 mM TCEP
8 in 0.5 mM Lys
8 in 0.5 mM Ser
8 in 0.5 mM NAC
8 in 0.5 mM Cys
8 in 0.5 mM Cys
Absorbance at 246 nm
k_obs = 0.0006 s^-1
k_2 = 1.16 M^-1s^-1
9 in buffer
9 in 0.5 mM TCEP
9 in 0.5 mM Lys
9 in 0.5 mM Ser
9 in 0.5 mM NAC
9 in 0.5 mM Cys
9 in 0.5 mM Cys
Absorbance at 254 nm
k_obs = 0.0005 s^-1
k_2 = 1.10 M^-1s^-1
10 in buffer
10 in 0.5 mM TCEP
10 in 0.5 mM Lys
10 in 0.5 mM Ser
10 in 0.5 mM NAC
10 in 0.5 mM Cys
10 in 0.5 mM Cys
Absorbance at 288 nm
k_obs = 0.0002 s^-1
k_2 = 0.49 M^-1s^-1
11 in buffer
11 in 0.5 mM TCEP
11 in 0.5 mM Lys
11 in 0.5 mM Ser
11 in 0.5 mM NAC
11 in 0.5 mM Cys
11 in 0.5 mM Cys
Absorbance at 244 nm
k_obs = 0.0005 s^-1
k_2 = 1.00 M^-1s^-1
12 in buffer
12 in 0.5 mM TCEP
12 in 0.5 mM Lys
12 in 0.5 mM Ser
12 in 0.5 mM NAC
12 in 0.5 mM Cys
12 in 0.5 mM Cys
Absorbance at 320 nm
k_obs = 0.0008 s^-1
k_2 = 1.66 M^-1s^-1
13 in buffer
13 in 0.5 mM TCEP
13 in 0.5 mM Lys
13 in 0.5 mM Ser
13 in 0.5 mM NAC
13 in 0.5 mM Cys
13 in 0.5 mM Cys
Absorbance at 320 nm
k_obs = 0.0007 s^-1
k_2 = 1.42 M^-1s^-1
14 in buffer
14 in 0.5 mM TCEP
14 in 0.5 mM Lys
14 in 0.5 mM Ser
14 in 0.5 mM NAC
14 in 0.5 mM Cys
14 in 0.5 mM Cys
Absorbance at 318 nm
k_obs = 0.0007 s^-1
k_2 = 1.40 M^-1s^-1
Absorbance with subtracted blank
Wavelength [nm]
Time [min]
Time [min]
0
15
30
60
120
180
240

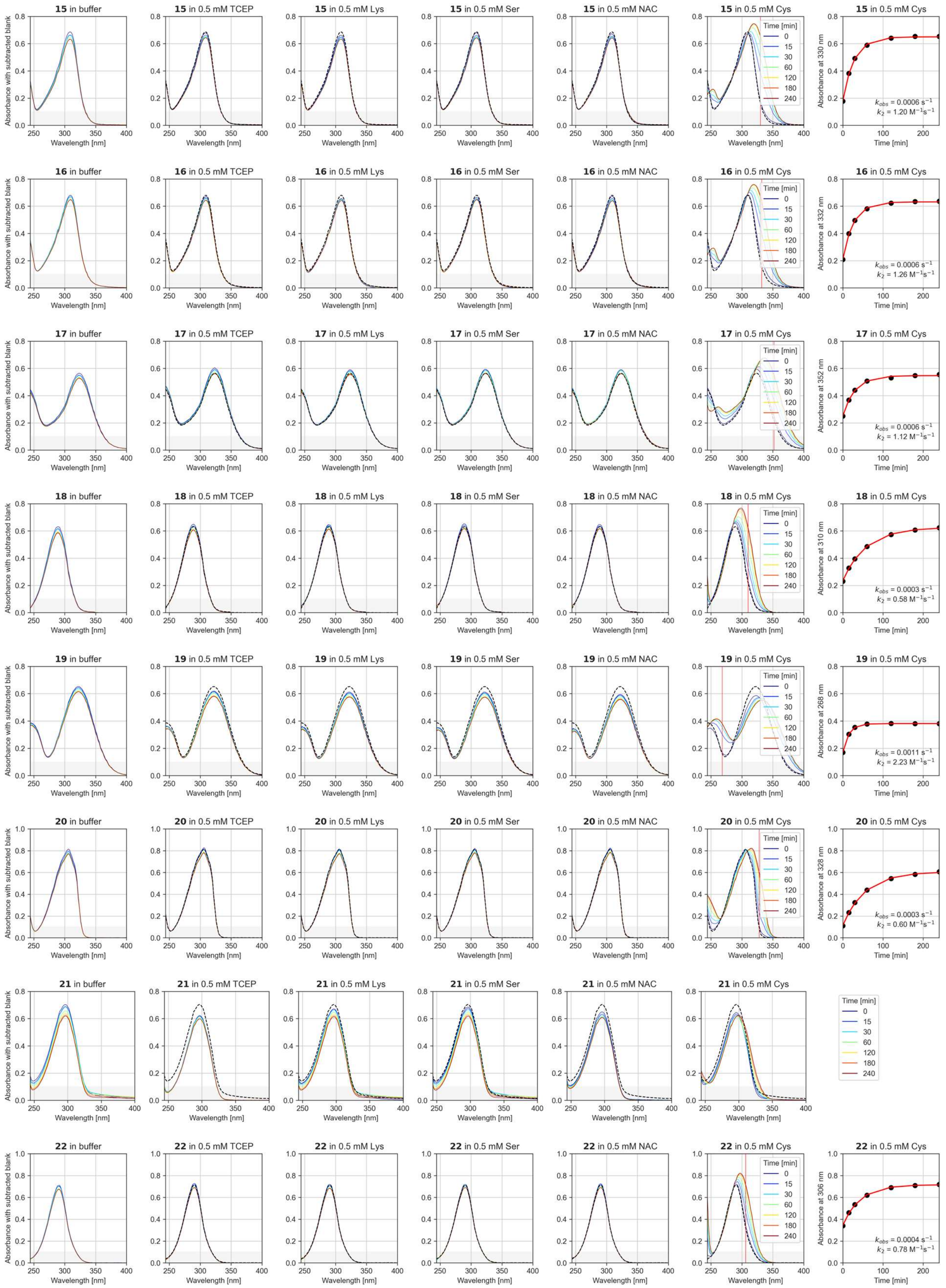
15 in buffer
15 in 0.5 mM TCEP
15 in 0.5 mM Lys
15 in 0.5 mM Ser
15 in 0.5 mM NAC
15 in 0.5 mM Cys
15 in 0.5 mM Cys
Absorbance at 330 nm
k_{obs} = 0.0006 s^{-1}
k_2 = 1.20 $M^{-1}s^{-1}$
16 in buffer
16 in 0.5 mM TCEP
16 in 0.5 mM Lys
16 in 0.5 mM Ser
16 in 0.5 mM NAC
16 in 0.5 mM Cys
16 in 0.5 mM Cys
Absorbance at 332 nm
k_{obs} = 0.0006 s^{-1}
k_2 = 1.26 $M^{-1}s^{-1}$
17 in buffer
17 in 0.5 mM TCEP
17 in 0.5 mM Lys
17 in 0.5 mM Ser
17 in 0.5 mM NAC
17 in 0.5 mM Cys
17 in 0.5 mM Cys
Absorbance at 352 nm
k_{obs} = 0.0006 s^{-1}
k_2 = 1.12 $M^{-1}s^{-1}$
18 in buffer
18 in 0.5 mM TCEP
18 in 0.5 mM Lys
18 in 0.5 mM Ser
18 in 0.5 mM NAC
18 in 0.5 mM Cys
18 in 0.5 mM Cys
Absorbance at 310 nm
k_{obs} = 0.0003 s^{-1}
k_2 = 0.58 $M^{-1}s^{-1}$
19 in buffer
19 in 0.5 mM TCEP
19 in 0.5 mM Lys
19 in 0.5 mM Ser
19 in 0.5 mM NAC
19 in 0.5 mM Cys
19 in 0.5 mM Cys
Absorbance at 268 nm
k_{obs} = 0.0011 s^{-1}
k_2 = 2.23 $M^{-1}s^{-1}$
20 in buffer
20 in 0.5 mM TCEP
20 in 0.5 mM Lys
20 in 0.5 mM Ser
20 in 0.5 mM NAC
20 in 0.5 mM Cys
20 in 0.5 mM Cys
Absorbance at 328 nm
k_{obs} = 0.0003 s^{-1}
k_2 = 0.60 $M^{-1}s^{-1}$
21 in buffer
21 in 0.5 mM TCEP
21 in 0.5 mM Lys
21 in 0.5 mM Ser
21 in 0.5 mM NAC
21 in 0.5 mM Cys
22 in buffer
22 in 0.5 mM TCEP
22 in 0.5 mM Lys
22 in 0.5 mM Ser
22 in 0.5 mM NAC
22 in 0.5 mM Cys
22 in 0.5 mM Cys
Absorbance at 306 nm
k_{obs} = 0.0004 s^{-1}
k_2 = 0.78 $M^{-1}s^{-1}$
Absorbance with subtracted blank
Wavelength [nm]
Time [min]
Time [min]
0
15
30
60
120
180
240

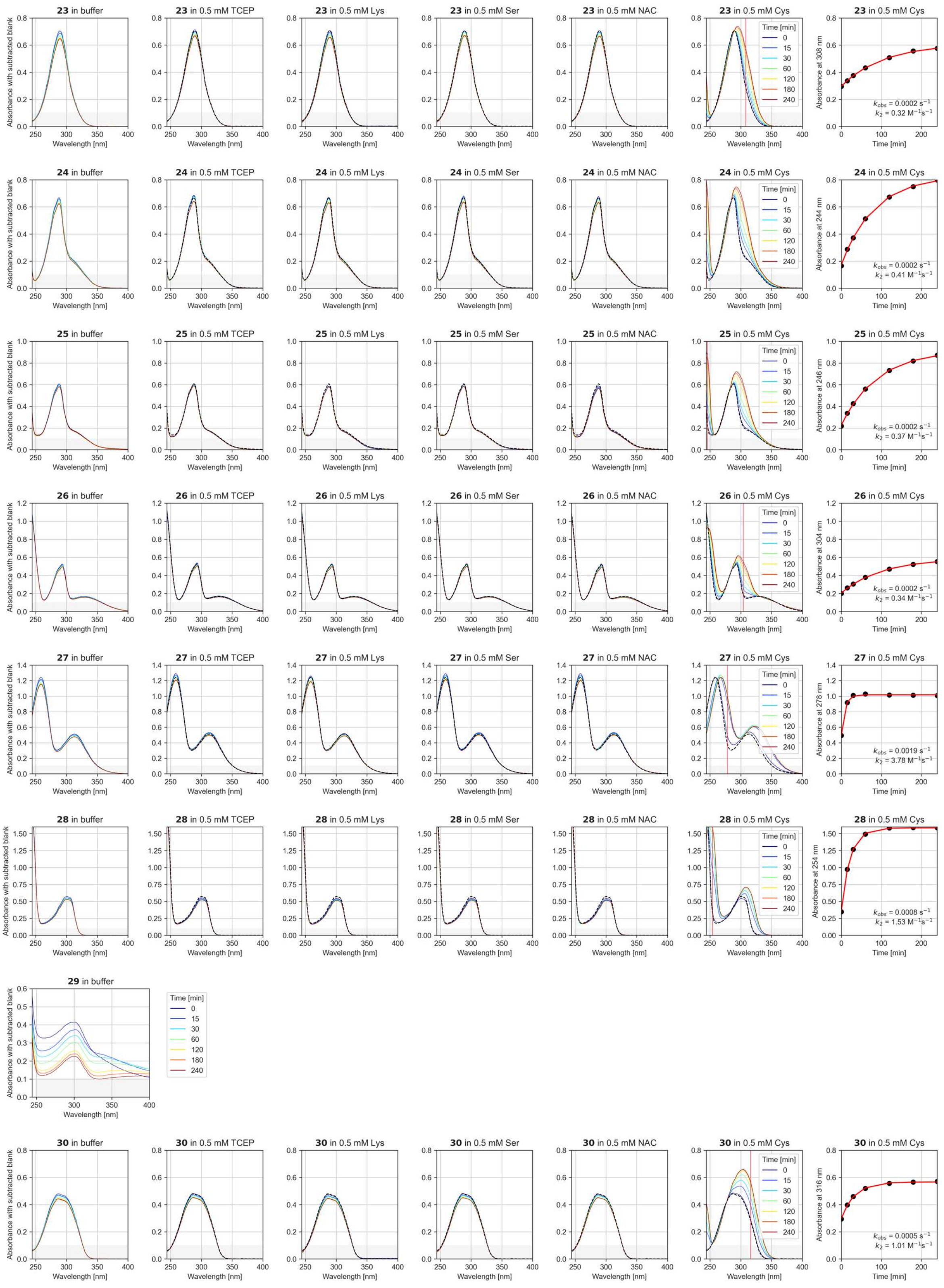
23 in buffer
23 in 0.5 mM TCEP
23 in 0.5 mM Lys
23 in 0.5 mM Ser
23 in 0.5 mM NAC
23 in 0.5 mM Cys
23 in 0.5 mM Cys
Absorbance at 308 nm
k_{obs} = 0.0002 s^{-1}
k_2 = 0.32 $M^{-1}s^{-1}$
24 in buffer
24 in 0.5 mM TCEP
24 in 0.5 mM Lys
24 in 0.5 mM Ser
24 in 0.5 mM NAC
24 in 0.5 mM Cys
24 in 0.5 mM Cys
Absorbance at 244 nm
k_{obs} = 0.0002 s^{-1}
k_2 = 0.41 $M^{-1}s^{-1}$
25 in buffer
25 in 0.5 mM TCEP
25 in 0.5 mM Lys
25 in 0.5 mM Ser
25 in 0.5 mM NAC
25 in 0.5 mM Cys
25 in 0.5 mM Cys
Absorbance at 246 nm
k_{obs} = 0.0002 s^{-1}
k_2 = 0.37 $M^{-1}s^{-1}$
26 in buffer
26 in 0.5 mM TCEP
26 in 0.5 mM Lys
26 in 0.5 mM Ser
26 in 0.5 mM NAC
26 in 0.5 mM Cys
26 in 0.5 mM Cys
Absorbance at 304 nm
k_{obs} = 0.0002 s^{-1}
k_2 = 0.34 $M^{-1}s^{-1}$
27 in buffer
27 in 0.5 mM TCEP
27 in 0.5 mM Lys
27 in 0.5 mM Ser
27 in 0.5 mM NAC
27 in 0.5 mM Cys
27 in 0.5 mM Cys
Absorbance at 278 nm
k_{obs} = 0.0019 s^{-1}
k_2 = 3.78 $M^{-1}s^{-1}$
28 in buffer
28 in 0.5 mM TCEP
28 in 0.5 mM Lys
28 in 0.5 mM Ser
28 in 0.5 mM NAC
28 in 0.5 mM Cys
28 in 0.5 mM Cys
Absorbance at 254 nm
k_{obs} = 0.0008 s^{-1}
k_2 = 1.53 $M^{-1}s^{-1}$
29 in buffer
30 in buffer
30 in 0.5 mM TCEP
30 in 0.5 mM Lys
30 in 0.5 mM Ser
30 in 0.5 mM NAC
30 in 0.5 mM Cys
30 in 0.5 mM Cys
Absorbance at 316 nm
k_{obs} = 0.0005 s^{-1}
k_2 = 1.01 $M^{-1}s^{-1}$
Absorbance with subtracted blank
Wavelength [nm]
Time [min]
0
15
30
60
120
180
240

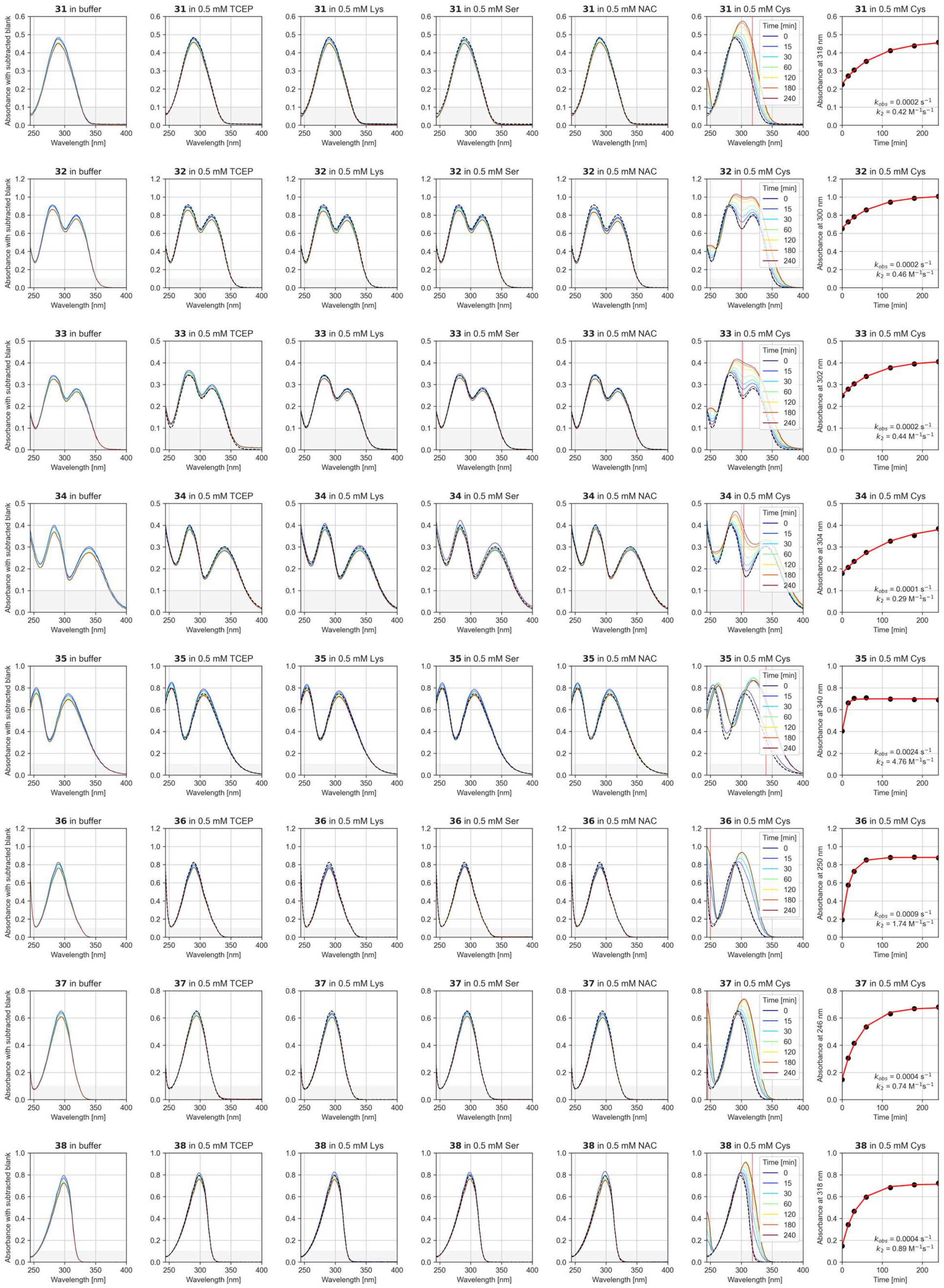
31 in buffer
31 in 0.5 mM TCEP
31 in 0.5 mM Lys
31 in 0.5 mM Ser
31 in 0.5 mM NAC
31 in 0.5 mM Cys
31 in 0.5 mM Cys
Absorbance at 318 nm
k_{obs} = 0.0002 s^{-1}
k_2 = 0.42 M^{-1}s^{-1}
32 in buffer
32 in 0.5 mM TCEP
32 in 0.5 mM Lys
32 in 0.5 mM Ser
32 in 0.5 mM NAC
32 in 0.5 mM Cys
32 in 0.5 mM Cys
Absorbance at 300 nm
k_{obs} = 0.0002 s^{-1}
k_2 = 0.46 M^{-1}s^{-1}
33 in buffer
33 in 0.5 mM TCEP
33 in 0.5 mM Lys
33 in 0.5 mM Ser
33 in 0.5 mM NAC
33 in 0.5 mM Cys
33 in 0.5 mM Cys
Absorbance at 302 nm
k_{obs} = 0.0002 s^{-1}
k_2 = 0.44 M^{-1}s^{-1}
34 in buffer
34 in 0.5 mM TCEP
34 in 0.5 mM Lys
34 in 0.5 mM Ser
34 in 0.5 mM NAC
34 in 0.5 mM Cys
34 in 0.5 mM Cys
Absorbance at 304 nm
k_{obs} = 0.0001 s^{-1}
k_2 = 0.29 M^{-1}s^{-1}
35 in buffer
35 in 0.5 mM TCEP
35 in 0.5 mM Lys
35 in 0.5 mM Ser
35 in 0.5 mM NAC
35 in 0.5 mM Cys
35 in 0.5 mM Cys
Absorbance at 340 nm
k_{obs} = 0.0024 s^{-1}
k_2 = 4.76 M^{-1}s^{-1}
36 in buffer
36 in 0.5 mM TCEP
36 in 0.5 mM Lys
36 in 0.5 mM Ser
36 in 0.5 mM NAC
36 in 0.5 mM Cys
36 in 0.5 mM Cys
Absorbance at 250 nm
k_{obs} = 0.0009 s^{-1}
k_2 = 1.74 M^{-1}s^{-1}
37 in buffer
37 in 0.5 mM TCEP
37 in 0.5 mM Lys
37 in 0.5 mM Ser
37 in 0.5 mM NAC
37 in 0.5 mM Cys
37 in 0.5 mM Cys
Absorbance at 246 nm
k_{obs} = 0.0004 s^{-1}
k_2 = 0.74 M^{-1}s^{-1}
38 in buffer
38 in 0.5 mM TCEP
38 in 0.5 mM Lys
38 in 0.5 mM Ser
38 in 0.5 mM NAC
38 in 0.5 mM Cys
38 in 0.5 mM Cys
Absorbance at 318 nm
k_{obs} = 0.0004 s^{-1}
k_2 = 0.89 M^{-1}s^{-1}
Absorbance with subtracted blank
Wavelength [nm]
Time [min]
0
15
30
60
120
180
240

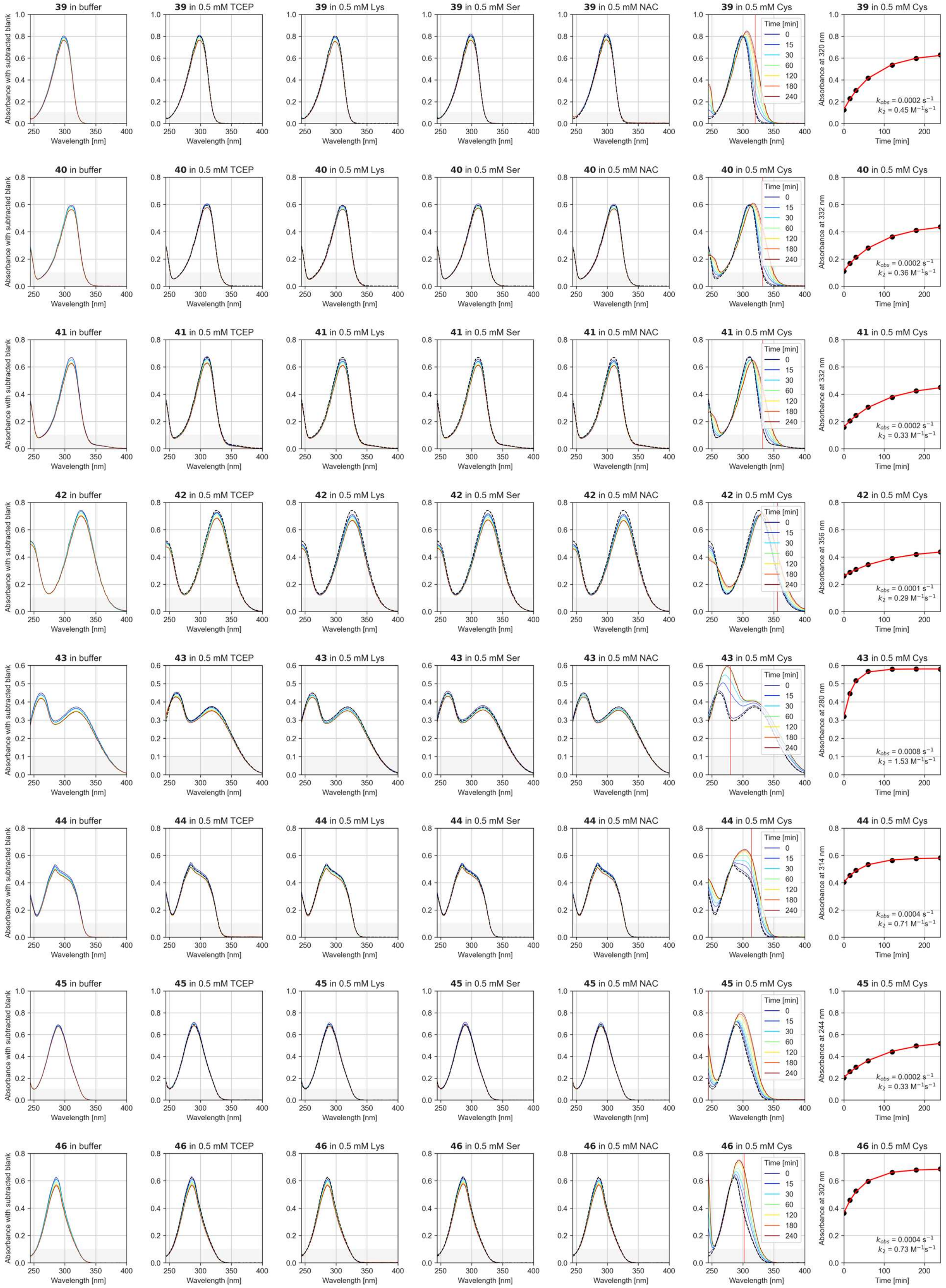
39 in buffer
39 in 0.5 mM TCEP
39 in 0.5 mM Lys
39 in 0.5 mM Ser
39 in 0.5 mM NAC
39 in 0.5 mM Cys
39 in 0.5 mM Cys
k_{obs} = 0.0002 s^{-1}
k_2 = 0.45 M^{-1}s^{-1}
Absorbance at 320 nm
40 in buffer
40 in 0.5 mM TCEP
40 in 0.5 mM Lys
40 in 0.5 mM Ser
40 in 0.5 mM NAC
40 in 0.5 mM Cys
40 in 0.5 mM Cys
k_{obs} = 0.0002 s^{-1}
k_2 = 0.36 M^{-1}s^{-1}
Absorbance at 332 nm
41 in buffer
41 in 0.5 mM TCEP
41 in 0.5 mM Lys
41 in 0.5 mM Ser
41 in 0.5 mM NAC
41 in 0.5 mM Cys
41 in 0.5 mM Cys
k_{obs} = 0.0002 s^{-1}
k_2 = 0.33 M^{-1}s^{-1}
Absorbance at 332 nm
42 in buffer
42 in 0.5 mM TCEP
42 in 0.5 mM Lys
42 in 0.5 mM Ser
42 in 0.5 mM NAC
42 in 0.5 mM Cys
42 in 0.5 mM Cys
k_{obs} = 0.0001 s^{-1}
k_2 = 0.29 M^{-1}s^{-1}
Absorbance at 356 nm
43 in buffer
43 in 0.5 mM TCEP
43 in 0.5 mM Lys
43 in 0.5 mM Ser
43 in 0.5 mM NAC
43 in 0.5 mM Cys
43 in 0.5 mM Cys
k_{obs} = 0.0008 s^{-1}
k_2 = 1.53 M^{-1}s^{-1}
Absorbance at 280 nm
44 in buffer
44 in 0.5 mM TCEP
44 in 0.5 mM Lys
44 in 0.5 mM Ser
44 in 0.5 mM NAC
44 in 0.5 mM Cys
44 in 0.5 mM Cys
k_{obs} = 0.0004 s^{-1}
k_2 = 0.71 M^{-1}s^{-1}
Absorbance at 314 nm
45 in buffer
45 in 0.5 mM TCEP
45 in 0.5 mM Lys
45 in 0.5 mM Ser
45 in 0.5 mM NAC
45 in 0.5 mM Cys
45 in 0.5 mM Cys
k_{obs} = 0.0002 s^{-1}
k_2 = 0.33 M^{-1}s^{-1}
Absorbance at 244 nm
46 in buffer
46 in 0.5 mM TCEP
46 in 0.5 mM Lys
46 in 0.5 mM Ser
46 in 0.5 mM NAC
46 in 0.5 mM Cys
46 in 0.5 mM Cys
k_{obs} = 0.0004 s^{-1}
k_2 = 0.73 M^{-1}s^{-1}
Absorbance at 302 nm
Absorbance with subtracted blank
Wavelength [nm]
Time [min]
0
15
30
60
120
180
240

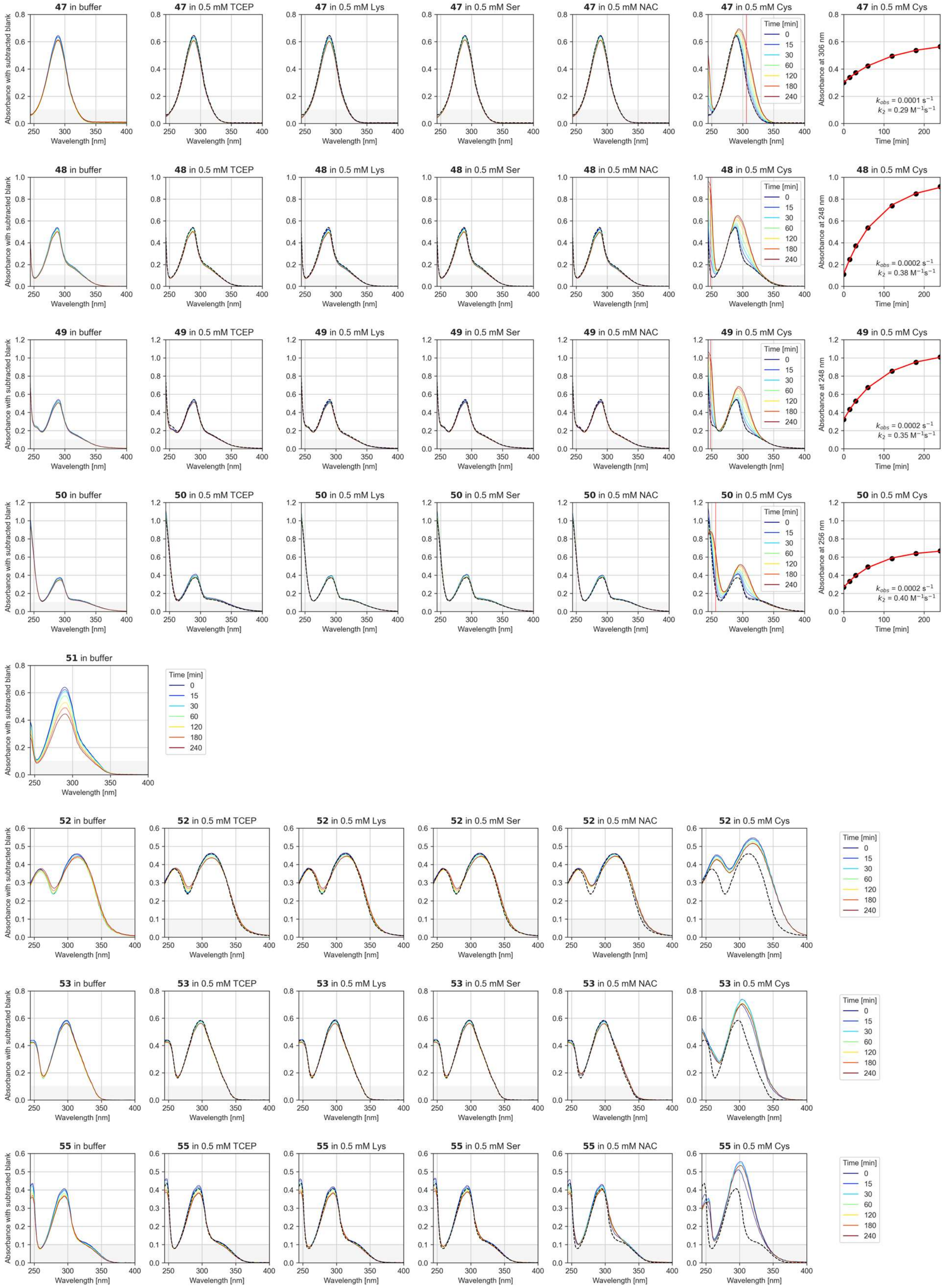
47 in buffer
47 in 0.5 mM TCEP
47 in 0.5 mM Lys
47 in 0.5 mM Ser
47 in 0.5 mM NAC
47 in 0.5 mM Cys
47 in 0.5 mM Cys
Absorbance at 306 nm
k_{obs} = 0.0001 s^{-1}
k_2 = 0.29 M^{-1}s^{-1}
48 in buffer
48 in 0.5 mM TCEP
48 in 0.5 mM Lys
48 in 0.5 mM Ser
48 in 0.5 mM NAC
48 in 0.5 mM Cys
48 in 0.5 mM Cys
Absorbance at 248 nm
k_{obs} = 0.0002 s^{-1}
k_2 = 0.38 M^{-1}s^{-1}
49 in buffer
49 in 0.5 mM TCEP
49 in 0.5 mM Lys
49 in 0.5 mM Ser
49 in 0.5 mM NAC
49 in 0.5 mM Cys
49 in 0.5 mM Cys
Absorbance at 248 nm
k_{obs} = 0.0002 s^{-1}
k_2 = 0.35 M^{-1}s^{-1}
50 in buffer
50 in 0.5 mM TCEP
50 in 0.5 mM Lys
50 in 0.5 mM Ser
50 in 0.5 mM NAC
50 in 0.5 mM Cys
50 in 0.5 mM Cys
Absorbance at 256 nm
k_{obs} = 0.0002 s^{-1}
k_2 = 0.40 M^{-1}s^{-1}
51 in buffer
52 in buffer
52 in 0.5 mM TCEP
52 in 0.5 mM Lys
52 in 0.5 mM Ser
52 in 0.5 mM NAC
52 in 0.5 mM Cys
53 in buffer
53 in 0.5 mM TCEP
53 in 0.5 mM Lys
53 in 0.5 mM Ser
53 in 0.5 mM NAC
53 in 0.5 mM Cys
55 in buffer
55 in 0.5 mM TCEP
55 in 0.5 mM Lys
55 in 0.5 mM Ser
55 in 0.5 mM NAC
55 in 0.5 mM Cys
Absorbance with subtracted blank
Wavelength [nm]
Time [min]
Time [min]
0
15
30
60
120
180
240

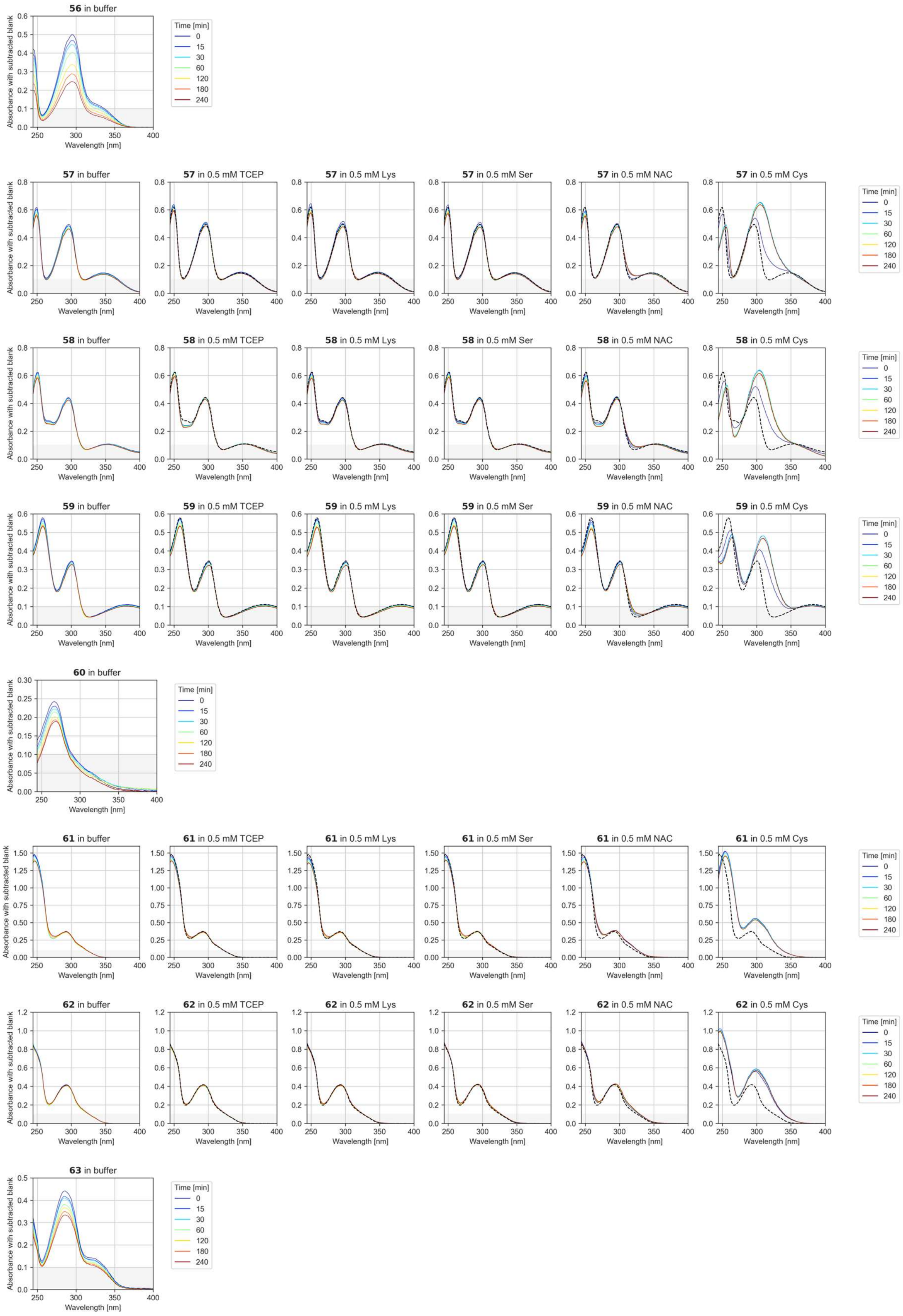

56 in buffer
57 in buffer
57 in 0.5 mM TCEP
57 in 0.5 mM Lys
57 in 0.5 mM Ser
57 in 0.5 mM NAC
57 in 0.5 mM Cys
58 in buffer
58 in 0.5 mM TCEP
58 in 0.5 mM Lys
58 in 0.5 mM Ser
58 in 0.5 mM NAC
58 in 0.5 mM Cys
59 in buffer
59 in 0.5 mM TCEP
59 in 0.5 mM Lys
59 in 0.5 mM Ser
59 in 0.5 mM NAC
59 in 0.5 mM Cys
60 in buffer
61 in buffer
61 in 0.5 mM TCEP
61 in 0.5 mM Lys
61 in 0.5 mM Ser
61 in 0.5 mM NAC
61 in 0.5 mM Cys
62 in buffer
62 in 0.5 mM TCEP
62 in 0.5 mM Lys
62 in 0.5 mM Ser
62 in 0.5 mM NAC
62 in 0.5 mM Cys
63 in buffer
Absorbance with subtracted blank
Wavelength [nm]
Time [min]
0
15
30
60
120
180
240

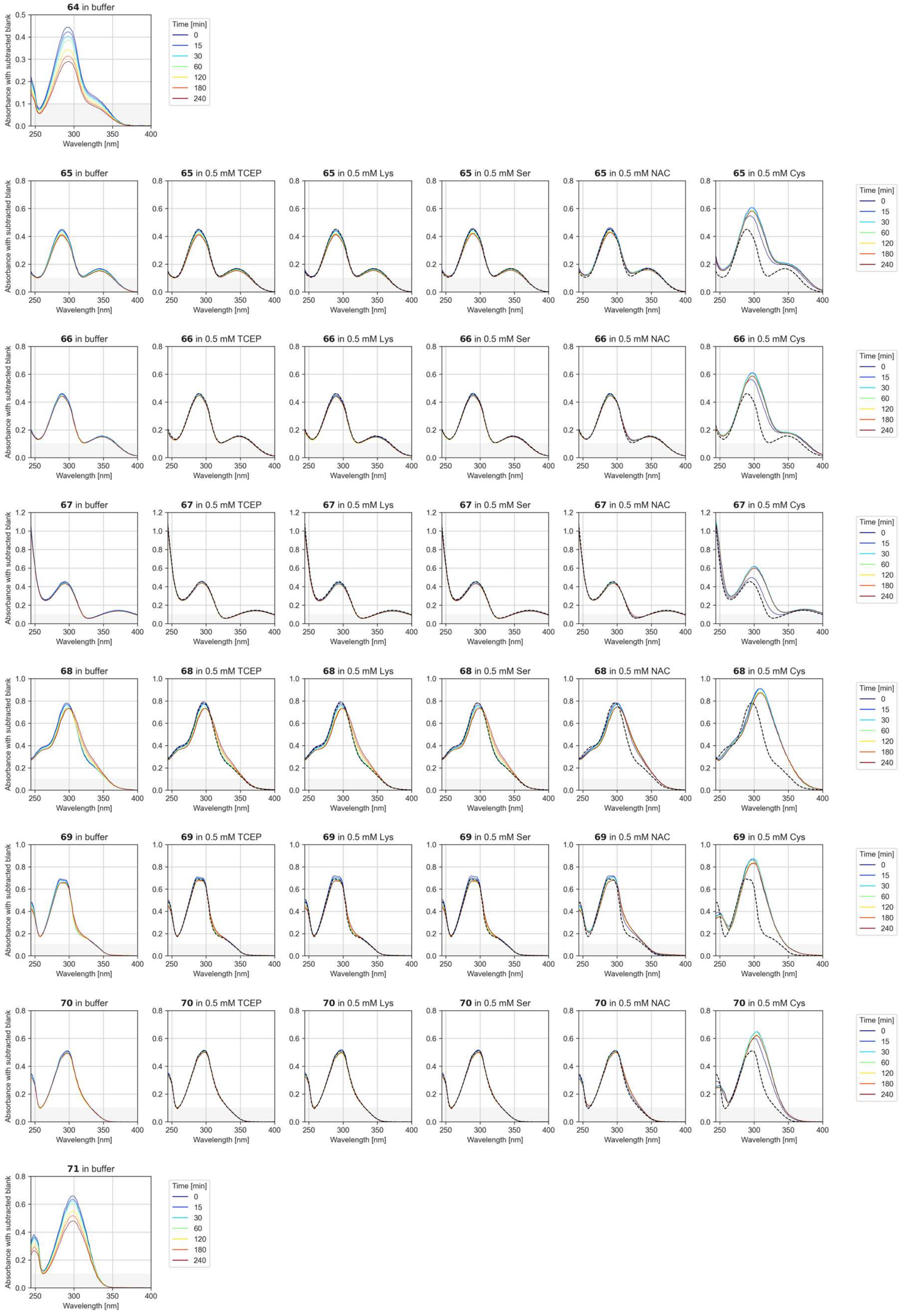
64 in buffer
65 in buffer
65 in 0.5 mM TCEP
65 in 0.5 mM Lys
65 in 0.5 mM Ser
65 in 0.5 mM NAC
65 in 0.5 mM Cys
66 in buffer
66 in 0.5 mM TCEP
66 in 0.5 mM Lys
66 in 0.5 mM Ser
66 in 0.5 mM NAC
66 in 0.5 mM Cys
67 in buffer
67 in 0.5 mM TCEP
67 in 0.5 mM Lys
67 in 0.5 mM Ser
67 in 0.5 mM NAC
67 in 0.5 mM Cys
68 in buffer
68 in 0.5 mM TCEP
68 in 0.5 mM Lys
68 in 0.5 mM Ser
68 in 0.5 mM NAC
68 in 0.5 mM Cys
69 in buffer
69 in 0.5 mM TCEP
69 in 0.5 mM Lys
69 in 0.5 mM Ser
69 in 0.5 mM NAC
69 in 0.5 mM Cys
70 in buffer
70 in 0.5 mM TCEP
70 in 0.5 mM Lys
70 in 0.5 mM Ser
70 in 0.5 mM NAC
70 in 0.5 mM Cys
71 in buffer
Absorbance with subtracted blank
Wavelength [nm]
Time [min]
0
15
30
60
120
180
240

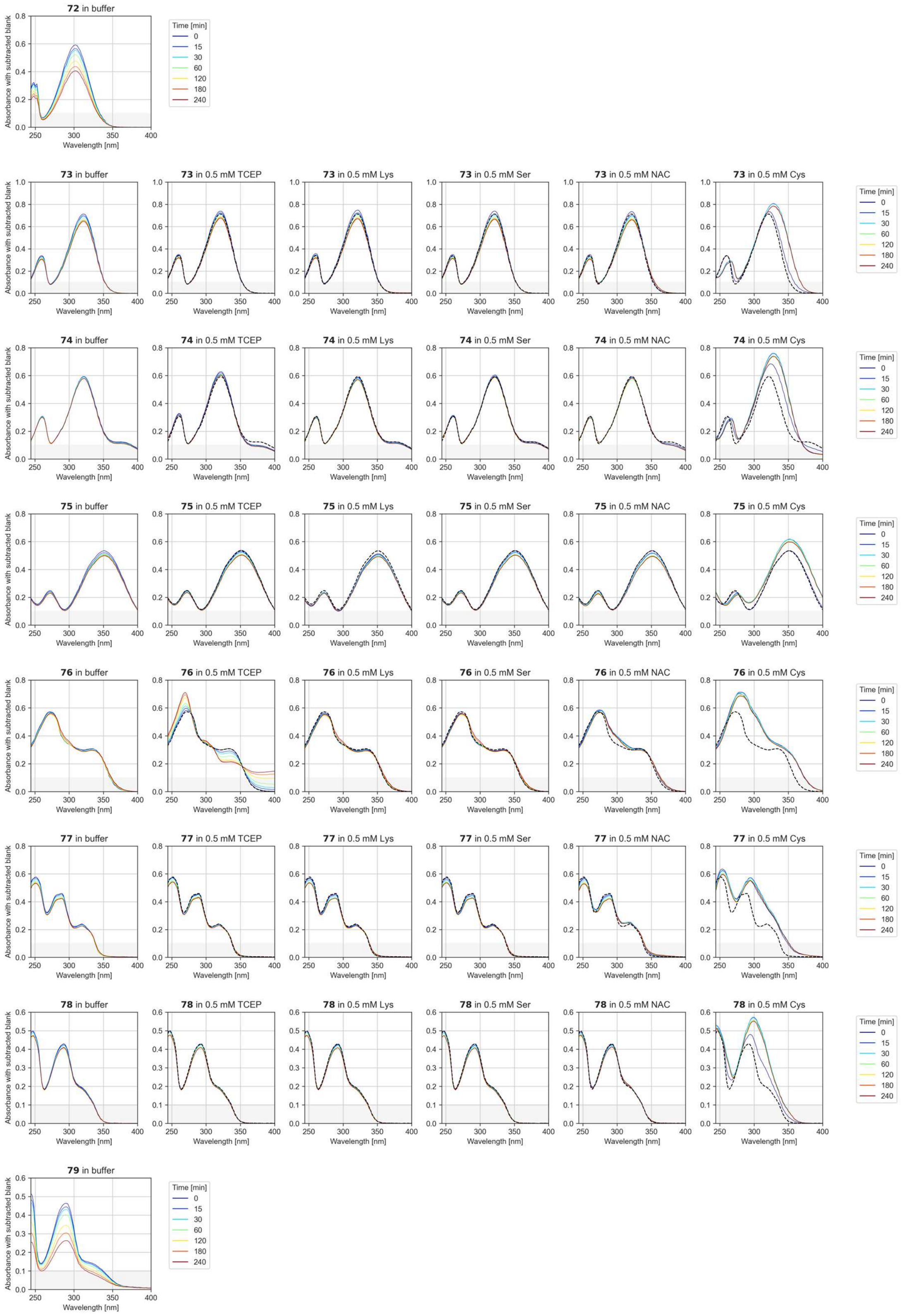
72 in buffer
73 in buffer
73 in 0.5 mM TCEP
73 in 0.5 mM Lys
73 in 0.5 mM Ser
73 in 0.5 mM NAC
73 in 0.5 mM Cys
74 in buffer
74 in 0.5 mM TCEP
74 in 0.5 mM Lys
74 in 0.5 mM Ser
74 in 0.5 mM NAC
74 in 0.5 mM Cys
75 in buffer
75 in 0.5 mM TCEP
75 in 0.5 mM Lys
75 in 0.5 mM Ser
75 in 0.5 mM NAC
75 in 0.5 mM Cys
76 in buffer
76 in 0.5 mM TCEP
76 in 0.5 mM Lys
76 in 0.5 mM Ser
76 in 0.5 mM NAC
76 in 0.5 mM Cys
77 in buffer
77 in 0.5 mM TCEP
77 in 0.5 mM Lys
77 in 0.5 mM Ser
77 in 0.5 mM NAC
77 in 0.5 mM Cys
78 in buffer
78 in 0.5 mM TCEP
78 in 0.5 mM Lys
78 in 0.5 mM Ser
78 in 0.5 mM NAC
78 in 0.5 mM Cys
79 in buffer
Absorbance with subtracted blank
Wavelength [nm]
Time [min]
0
15
30
60
120
180
240

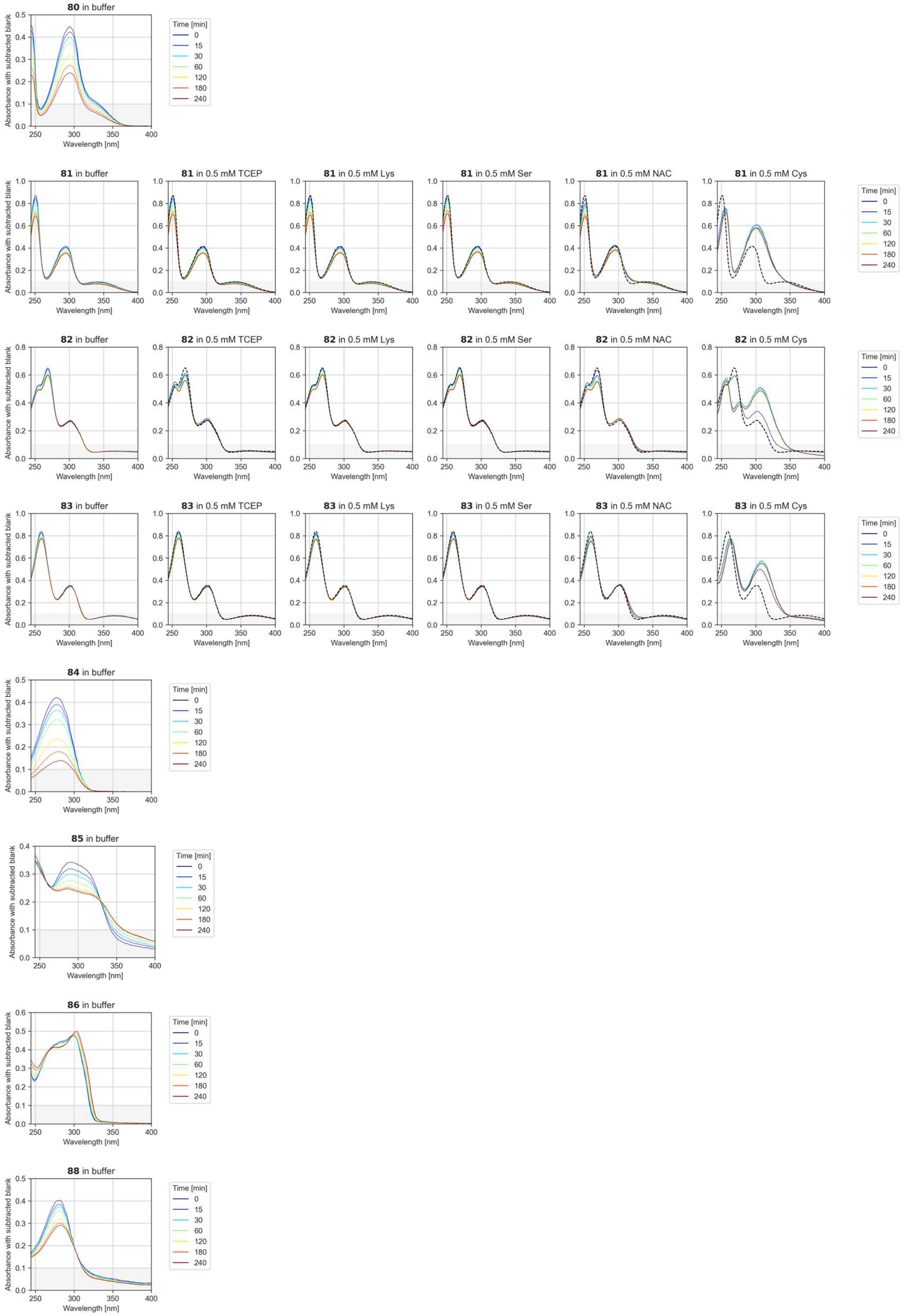
80 in buffer
81 in buffer
81 in 0.5 mM TCEP
81 in 0.5 mM Lys
81 in 0.5 mM Ser
81 in 0.5 mM NAC
81 in 0.5 mM Cys
82 in buffer
82 in 0.5 mM TCEP
82 in 0.5 mM Lys
82 in 0.5 mM Ser
82 in 0.5 mM NAC
82 in 0.5 mM Cys
83 in buffer
83 in 0.5 mM TCEP
83 in 0.5 mM Lys
83 in 0.5 mM Ser
83 in 0.5 mM NAC
83 in 0.5 mM Cys
84 in buffer
85 in buffer
86 in buffer
88 in buffer
Absorbance with subtracted blank
Wavelength [nm]
Time [min]
0
15
30
60
120
180
240

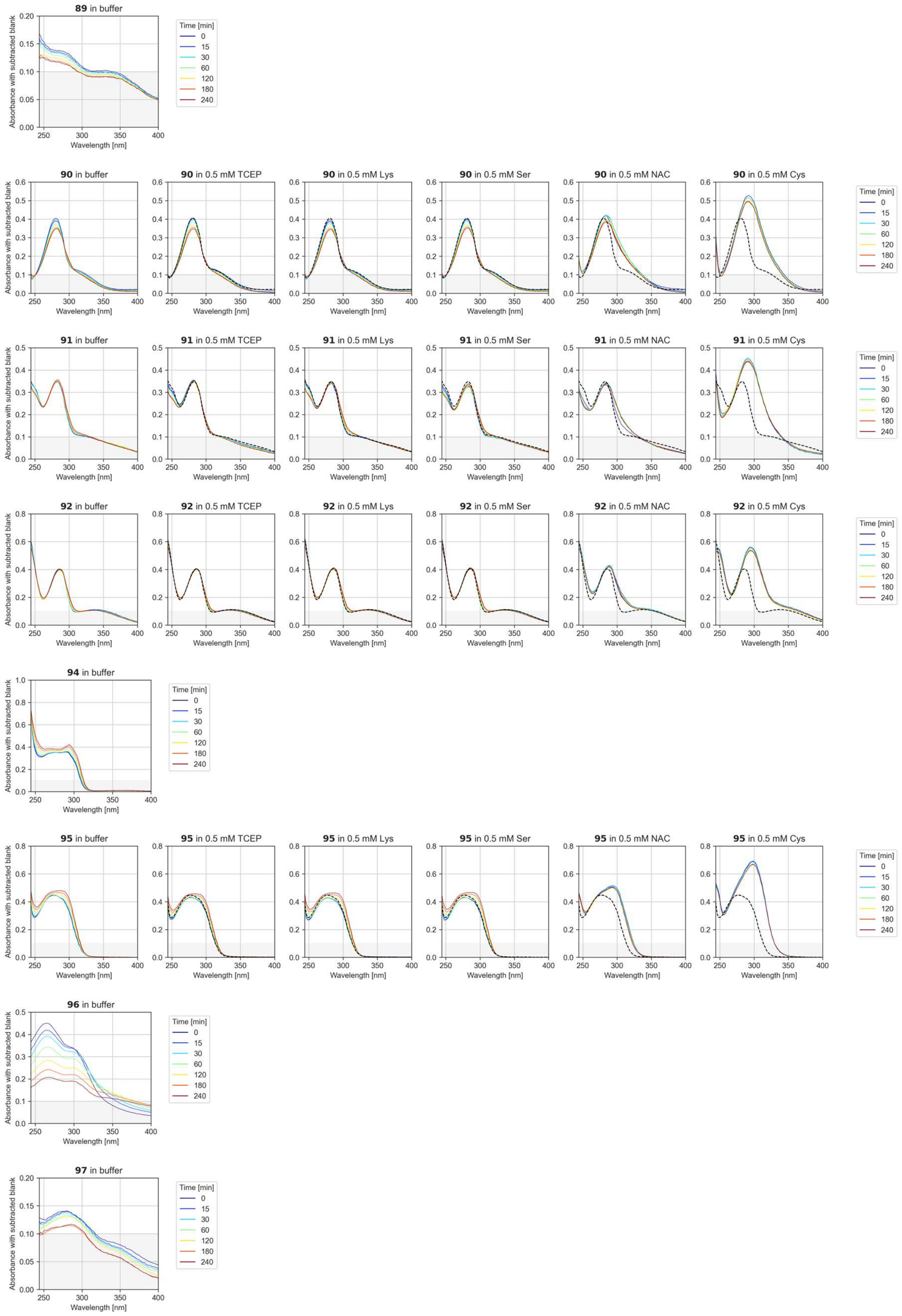
89 in buffer
90 in buffer
90 in 0.5 mM TCEP
90 in 0.5 mM Lys
90 in 0.5 mM Ser
90 in 0.5 mM NAC
90 in 0.5 mM Cys
91 in buffer
91 in 0.5 mM TCEP
91 in 0.5 mM Lys
91 in 0.5 mM Ser
91 in 0.5 mM NAC
91 in 0.5 mM Cys
92 in buffer
92 in 0.5 mM TCEP
92 in 0.5 mM Lys
92 in 0.5 mM Ser
92 in 0.5 mM NAC
92 in 0.5 mM Cys
94 in buffer
95 in buffer
95 in 0.5 mM TCEP
95 in 0.5 mM Lys
95 in 0.5 mM Ser
95 in 0.5 mM NAC
95 in 0.5 mM Cys
96 in buffer
97 in buffer
Absorbance with subtracted blank
Wavelength [nm]
Time [min]
0
15
30
60
120
180
240

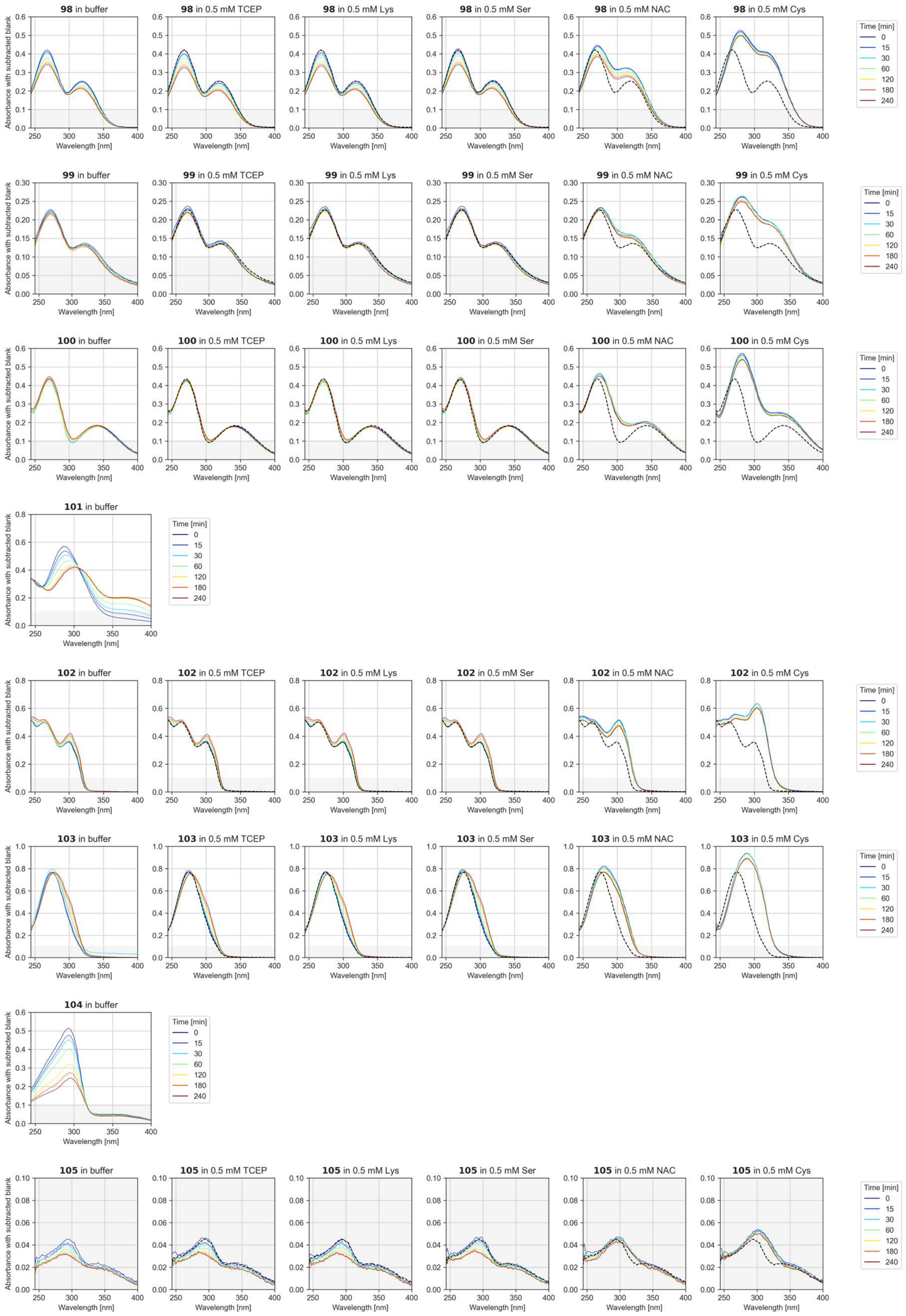
98 in buffer
98 in 0.5 mM TCEP
98 in 0.5 mM Lys
98 in 0.5 mM Ser
98 in 0.5 mM NAC
98 in 0.5 mM Cys
99 in buffer
99 in 0.5 mM TCEP
99 in 0.5 mM Lys
99 in 0.5 mM Ser
99 in 0.5 mM NAC
99 in 0.5 mM Cys
100 in buffer
100 in 0.5 mM TCEP
100 in 0.5 mM Lys
100 in 0.5 mM Ser
100 in 0.5 mM NAC
100 in 0.5 mM Cys
101 in buffer
102 in buffer
102 in 0.5 mM TCEP
102 in 0.5 mM Lys
102 in 0.5 mM Ser
102 in 0.5 mM NAC
102 in 0.5 mM Cys
103 in buffer
103 in 0.5 mM TCEP
103 in 0.5 mM Lys
103 in 0.5 mM Ser
103 in 0.5 mM NAC
103 in 0.5 mM Cys
104 in buffer
105 in buffer
105 in 0.5 mM TCEP
105 in 0.5 mM Lys
105 in 0.5 mM Ser
105 in 0.5 mM NAC
105 in 0.5 mM Cys
Absorbance with subtracted blank
Wavelength [nm]
Time [min]
0
15
30
60
120
180
240

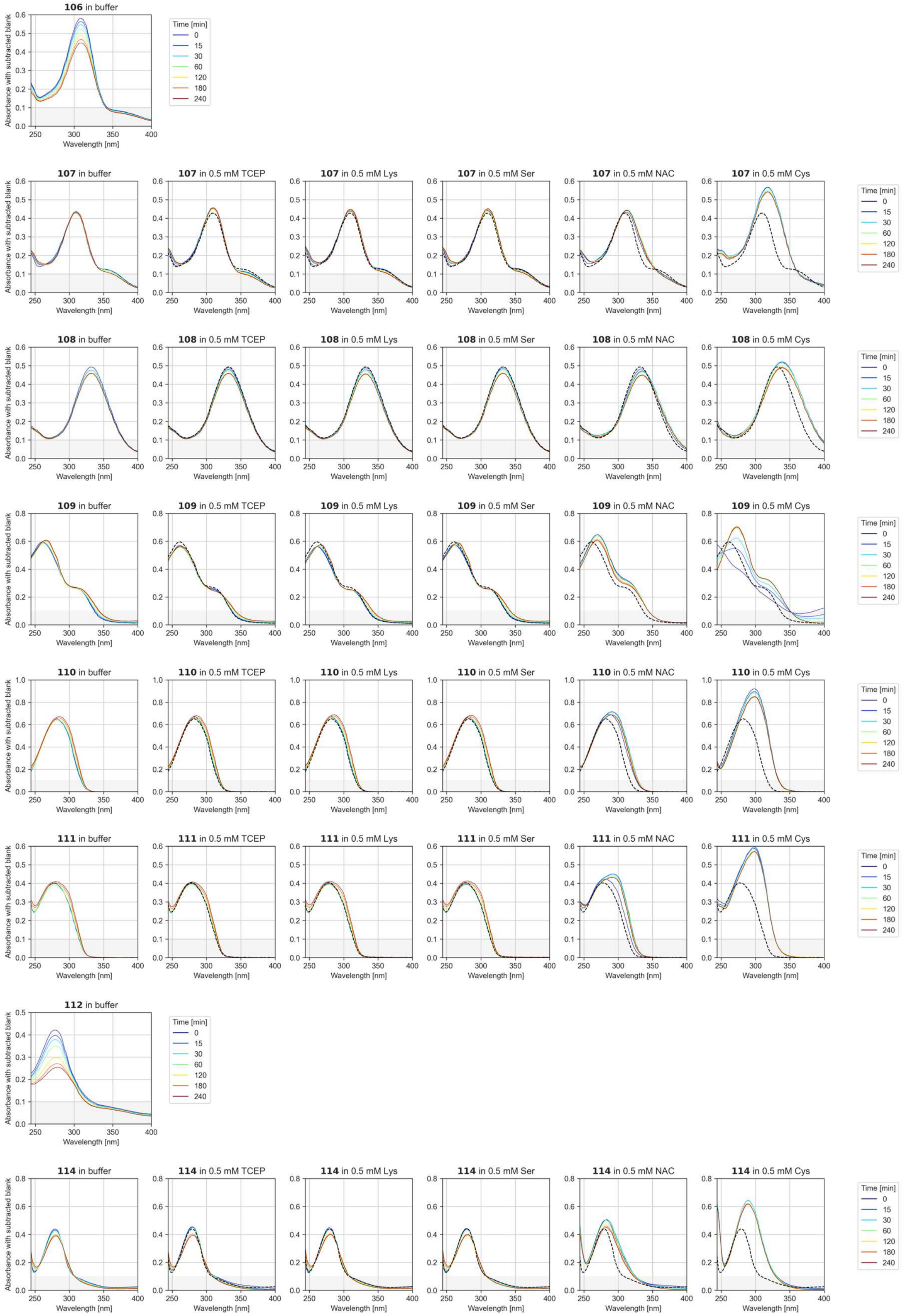

106 in buffer
107 in buffer
107 in 0.5 mM TCEP
107 in 0.5 mM Lys
107 in 0.5 mM Ser
107 in 0.5 mM NAC
107 in 0.5 mM Cys
108 in buffer
108 in 0.5 mM TCEP
108 in 0.5 mM Lys
108 in 0.5 mM Ser
108 in 0.5 mM NAC
108 in 0.5 mM Cys
109 in buffer
109 in 0.5 mM TCEP
109 in 0.5 mM Lys
109 in 0.5 mM Ser
109 in 0.5 mM NAC
109 in 0.5 mM Cys
110 in buffer
110 in 0.5 mM TCEP
110 in 0.5 mM Lys
110 in 0.5 mM Ser
110 in 0.5 mM NAC
110 in 0.5 mM Cys
111 in buffer
111 in 0.5 mM TCEP
111 in 0.5 mM Lys
111 in 0.5 mM Ser
111 in 0.5 mM NAC
111 in 0.5 mM Cys
112 in buffer
114 in buffer
114 in 0.5 mM TCEP
114 in 0.5 mM Lys
114 in 0.5 mM Ser
114 in 0.5 mM NAC
114 in 0.5 mM Cys
Absorbance with subtracted blank
Wavelength [nm]
Time [min]
0
15
30
60
120
180
240

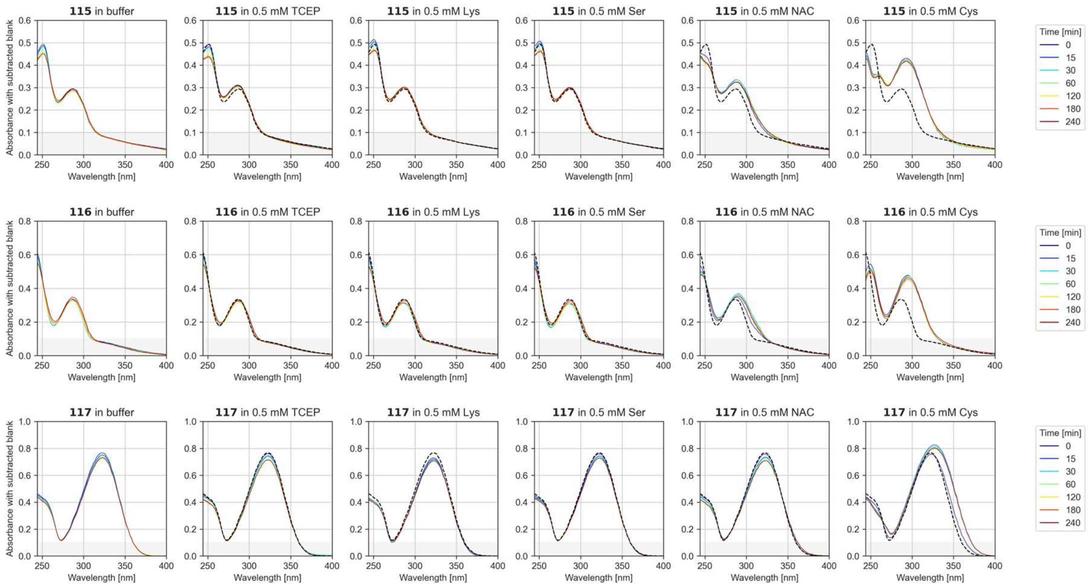
115 in buffer
115 in 0.5 mM TCEP
115 in 0.5 mM Lys
115 in 0.5 mM Ser
115 in 0.5 mM NAC
115 in 0.5 mM Cys
116 in buffer
116 in 0.5 mM TCEP
116 in 0.5 mM Lys
116 in 0.5 mM Ser
116 in 0.5 mM NAC
116 in 0.5 mM Cys
117 in buffer
117 in 0.5 mM TCEP
117 in 0.5 mM Lys
117 in 0.5 mM Ser
117 in 0.5 mM NAC
117 in 0.5 mM Cys
Absorbance with subtracted blank
Wavelength [nm]
Time [min]
0
15
30
60
120
180
240

4 Oligopeptide Labeling

The following chromatograms show absorbance at 280 nm, total ion count (TIC), and extracted ions for oligopeptides, compounds and their adducts. The compounds (2 eq.) were incubated with the oligopeptides (1 eq.) in presence of TCEP (10 eq.) at 37 °C for 30 min and analyzed by LCMS, unless stated otherwise.

Blank **UP1** (CGKGCGSGYGW):

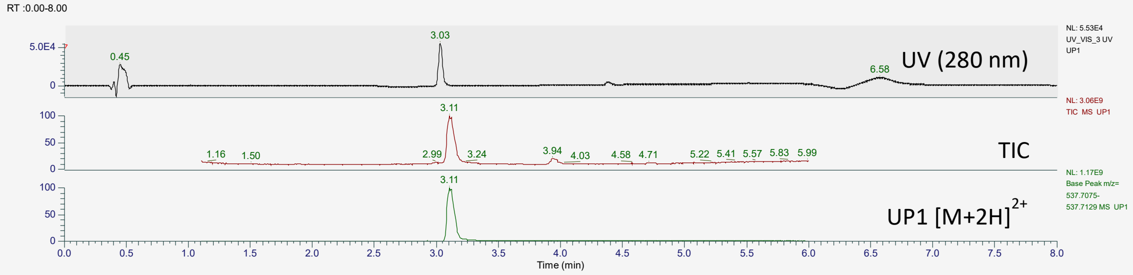

Blank **UP2** (AGKGCGSGYGW):

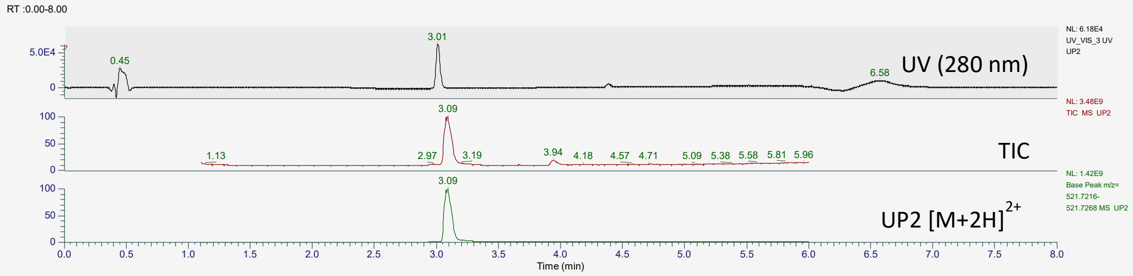

4 + UP1 (CGKGCGSGYGW):

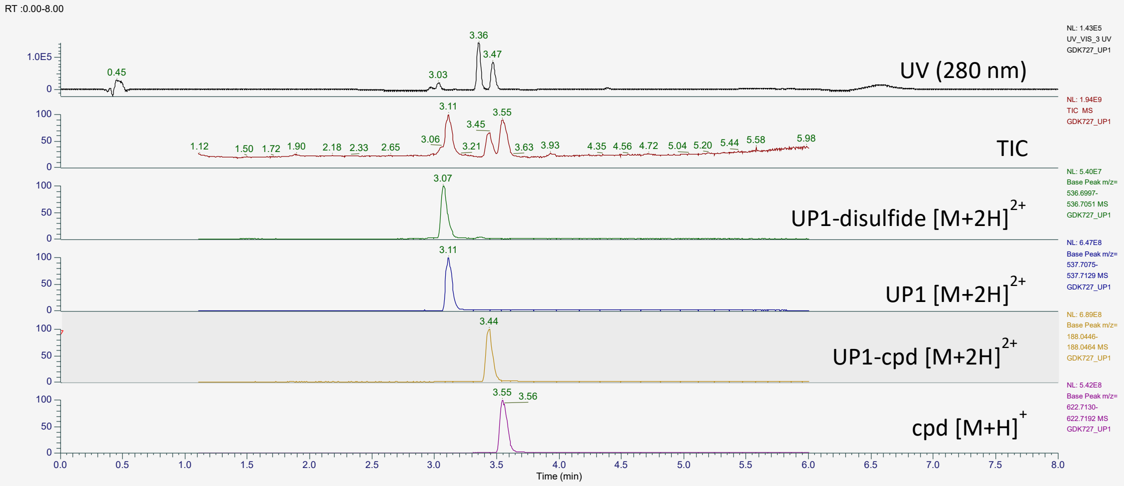

4 + UP2 (AGKGCGSGYGW):

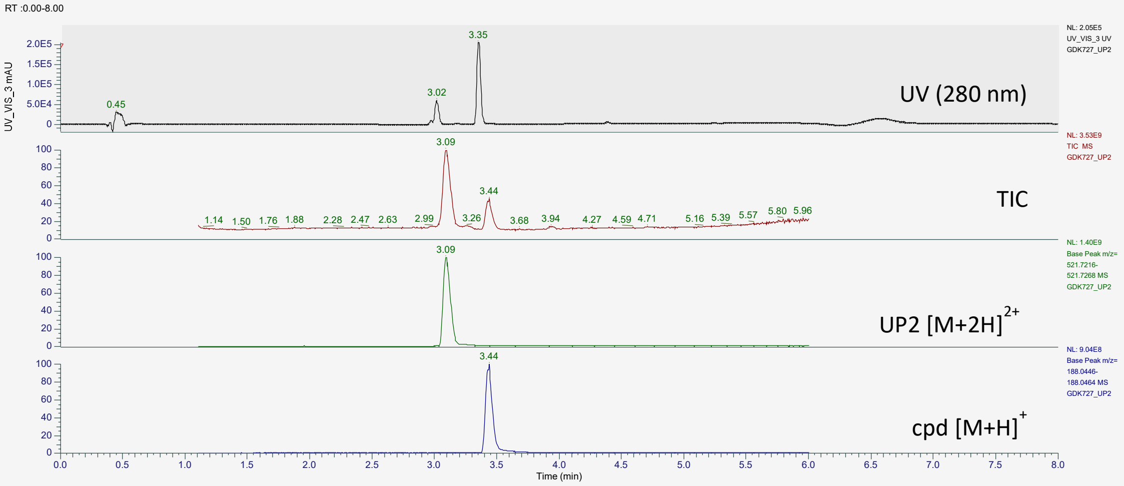

12 + **UP1** (CGKGCGSGYGW):

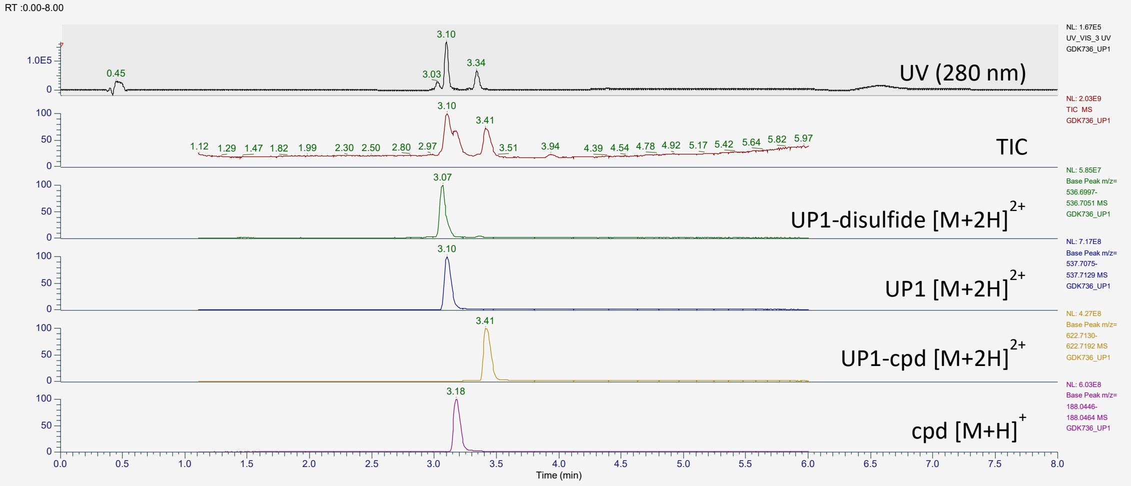

12 + **UP2** (AGKGCGSGYGW):

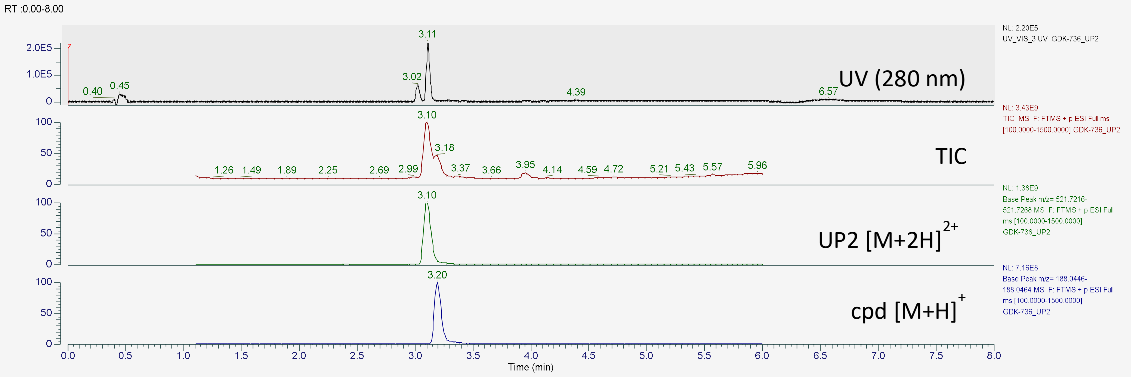

12 + **UP1** (CGKGCGSGYGW), 3 h incubation:

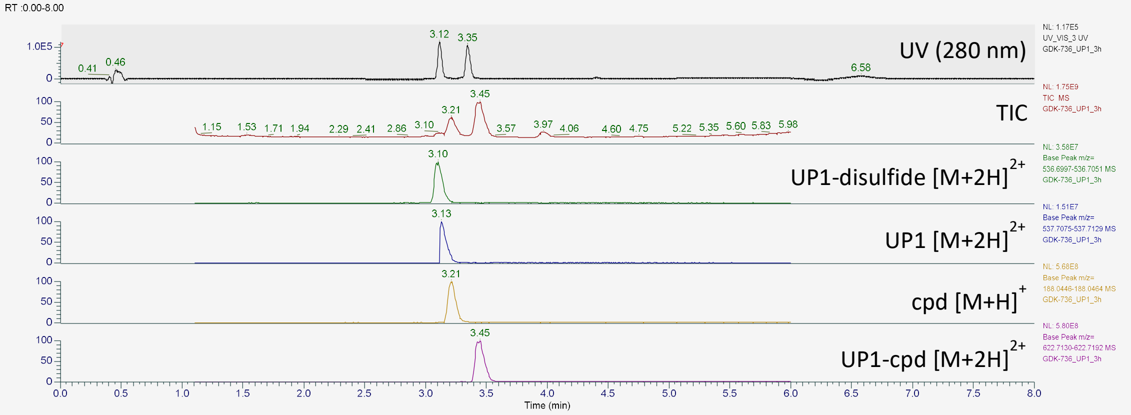

12 + **UP2** (AGKGCGSGYGW), 3 h incubation:

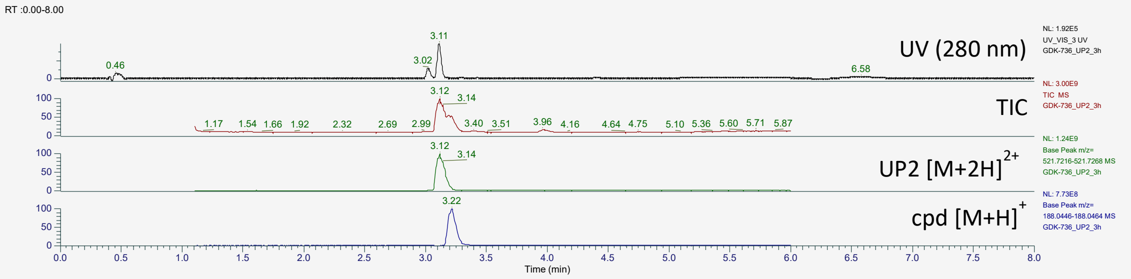

12 (10 eq.) + **UP1** (CGKGCGSGYGW):

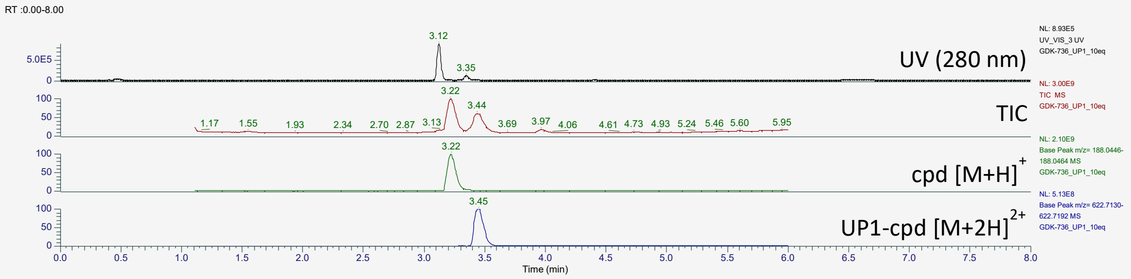

12 (10 eq.) + **UP2** (AGKGCGSGYGW):

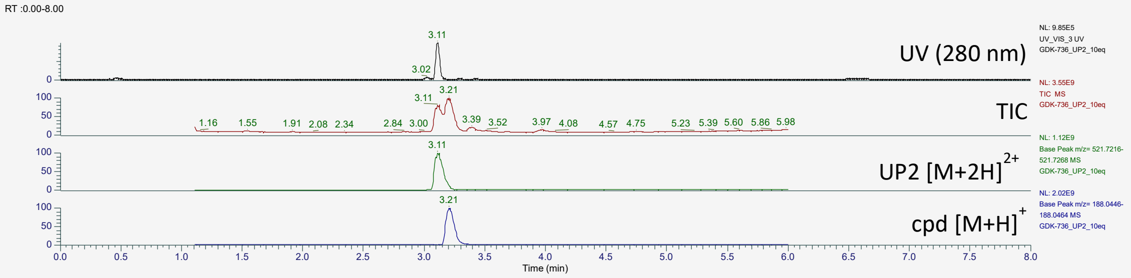

21 + UP1 (CGKGCGSGYGW):

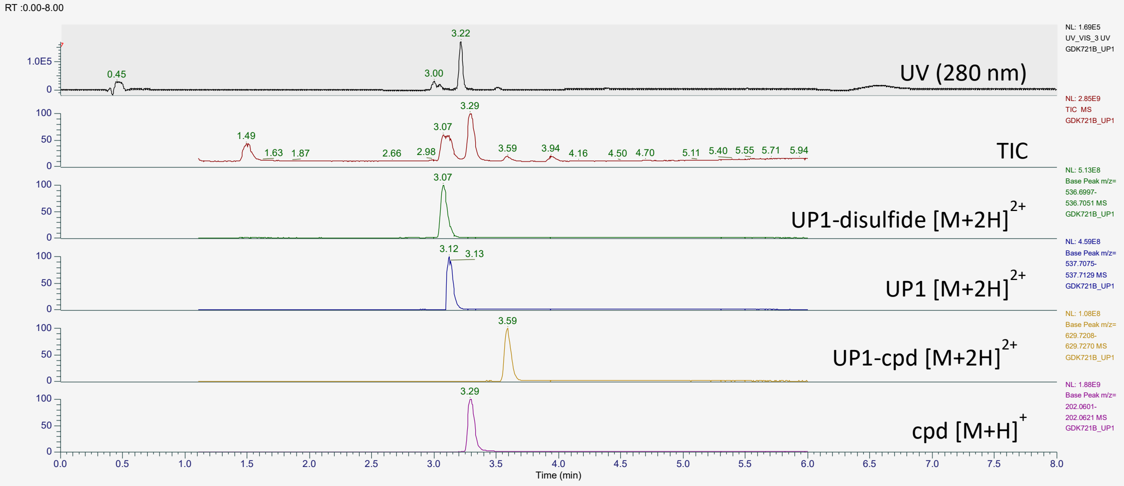

21 + UP2 (AGKGCGSGYGW):

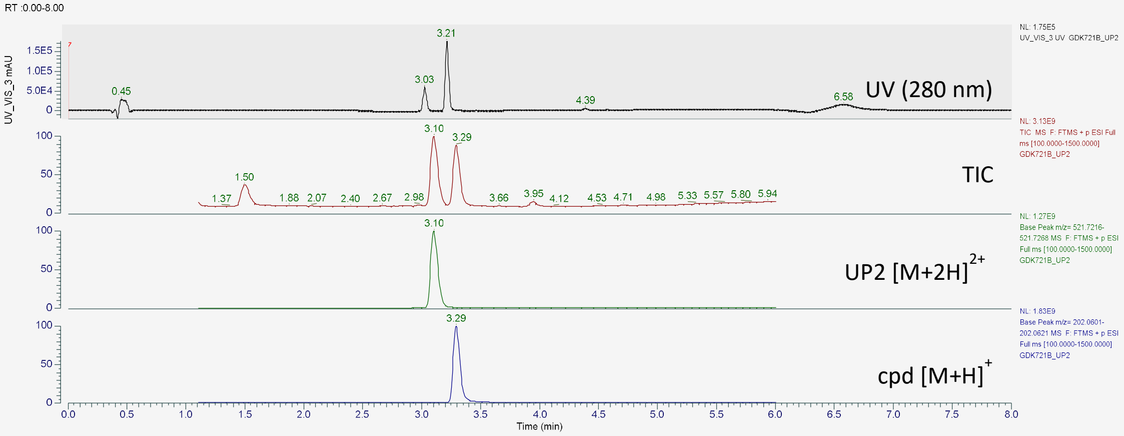

27 + UP1 (CGKGCGSGYGW):

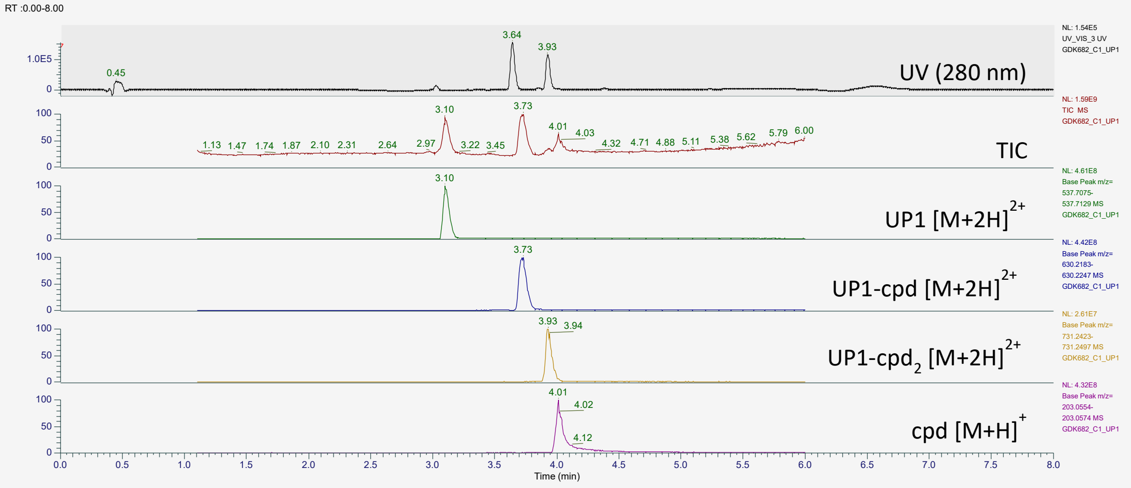

27 + UP2 (AGKGCGSGYGW):

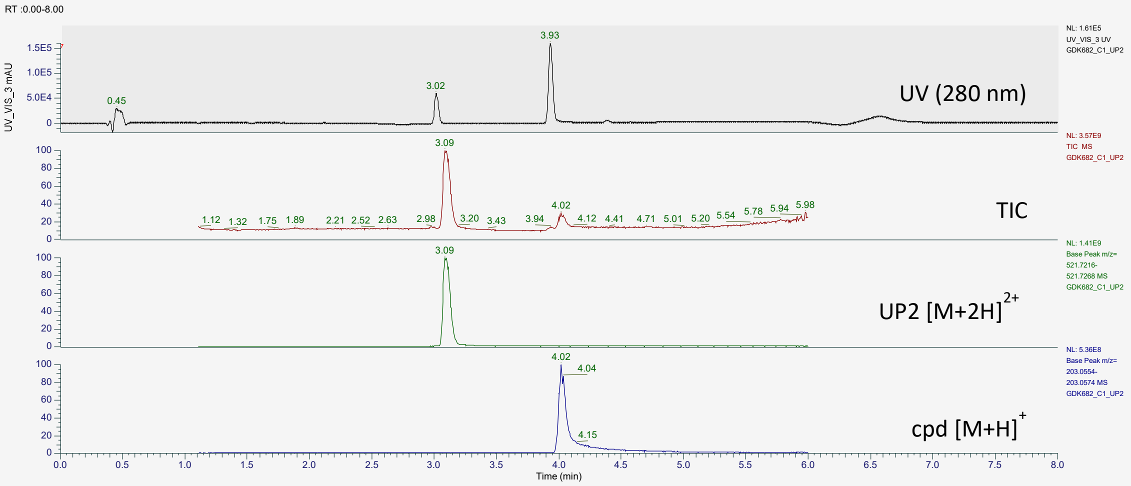

35 + UP1 (CGKGCGSGYGW):

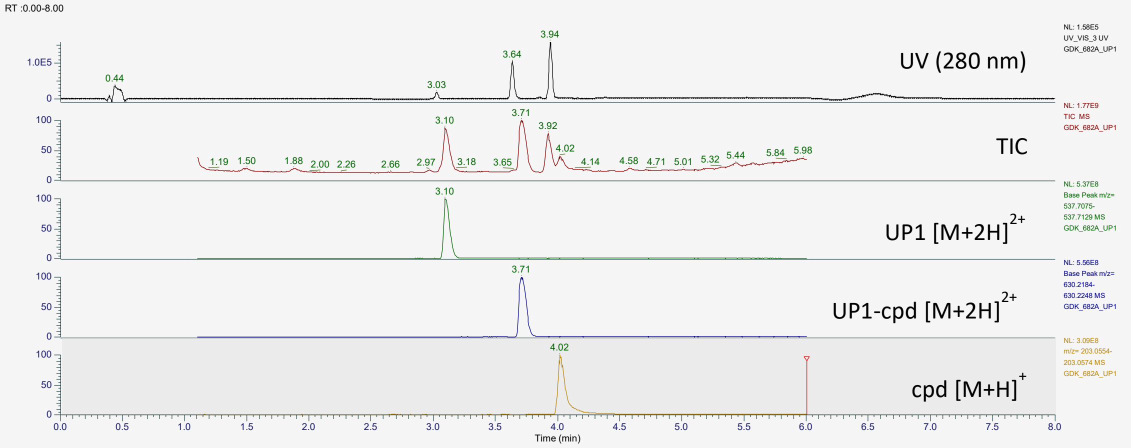

35 + UP2 (AGKGCGSGYGW):

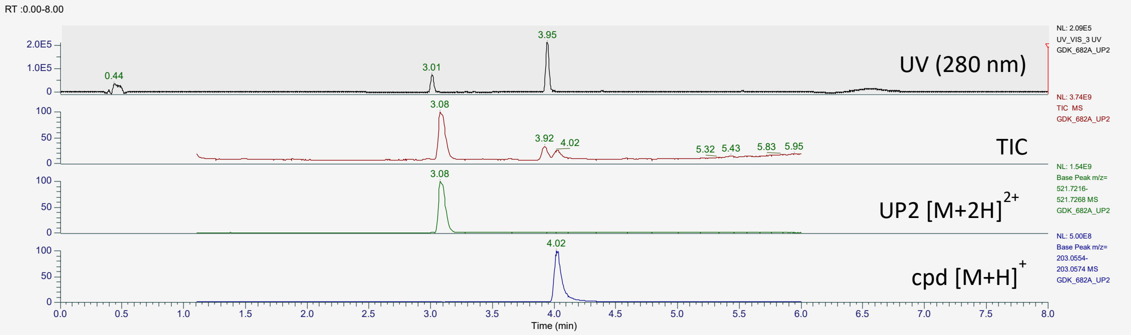

35 + UP1 (CGKGCGSGYGW)

Incubation: 60 min at 37 °C

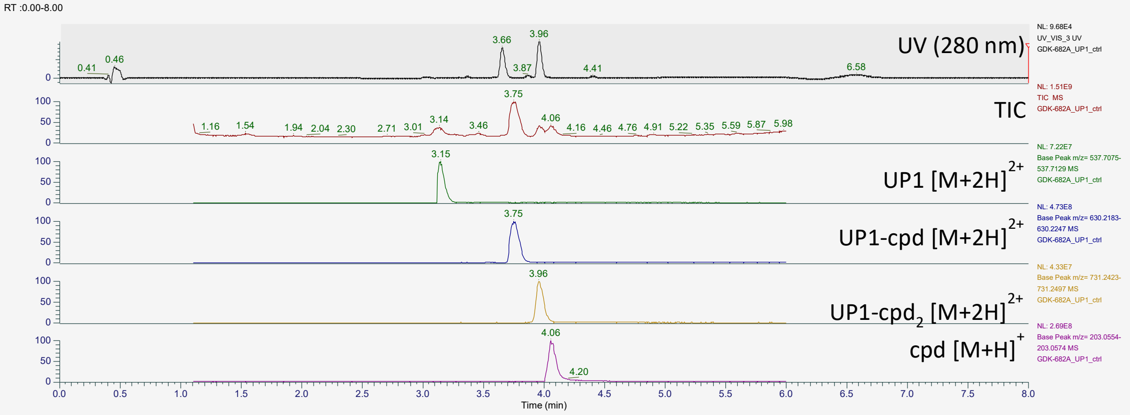

35 + UP1 (CGKGCGSGYGW)

Incubation: 30 min at 37 °C + 30 min with Cys at 37 °C

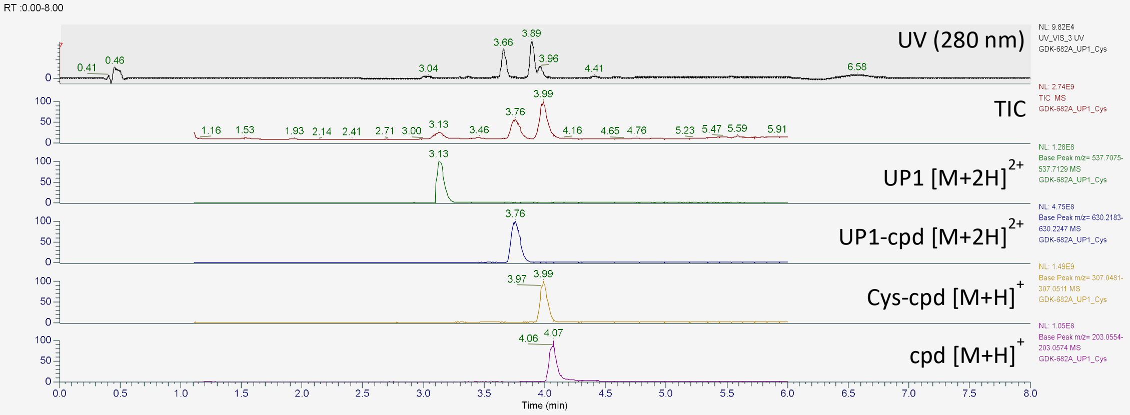

35 + UP2 (AGKGCGSGYGW)

Incubation: 60 min at 37 °C

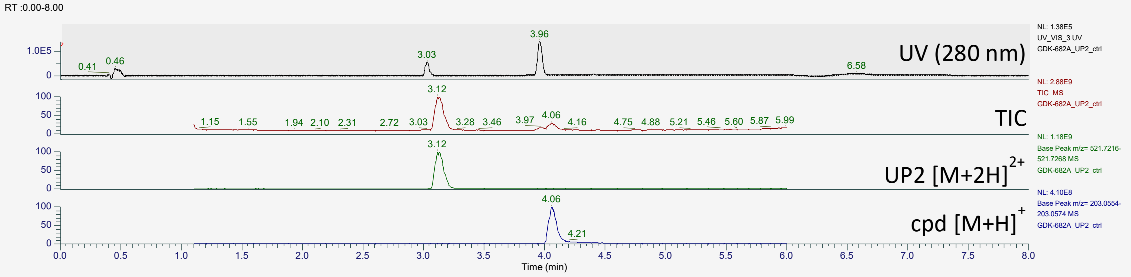

35 + UP2 (AGKGCGSGYGW)

Incubation: 30 min at 37 °C + 30 min with Cys at 37 °C

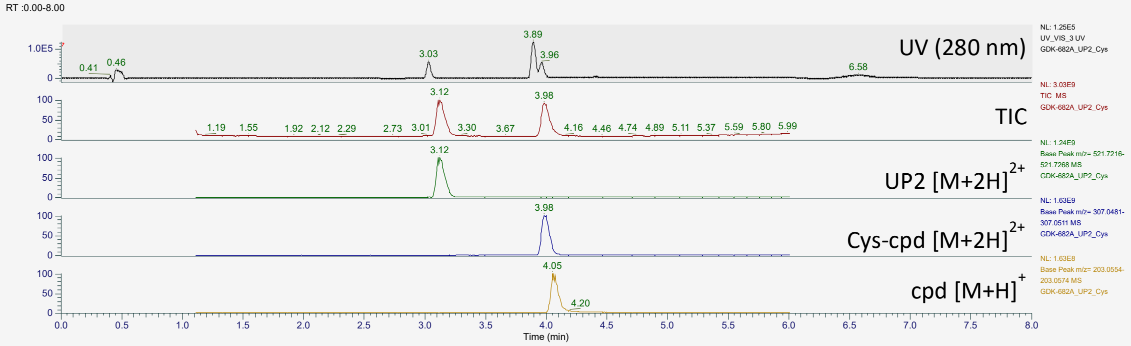

35 + UP1 (CGKGCGSGYGW)

Incubation: 60 min at 37 °C

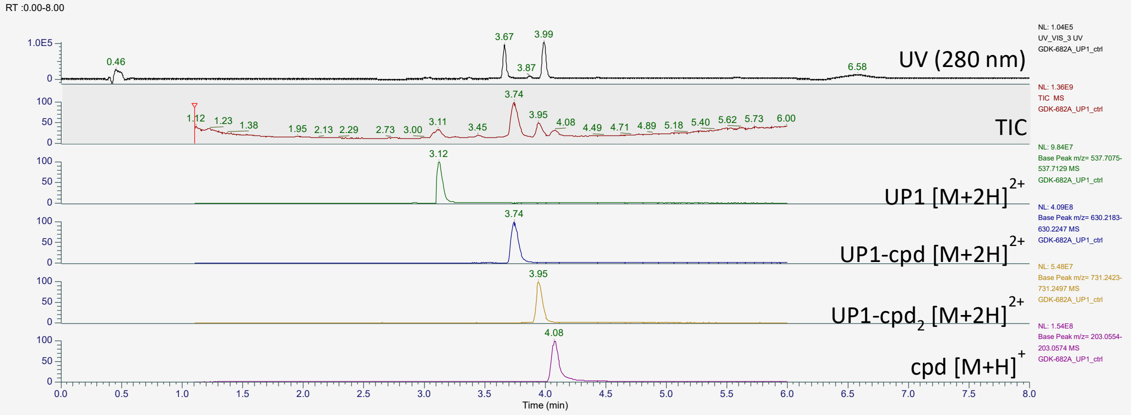

35 + UP1 (CGKGCGSGYGW)

Incubation: 30 min at 37 °C + 30 min with DTT at 37 °C

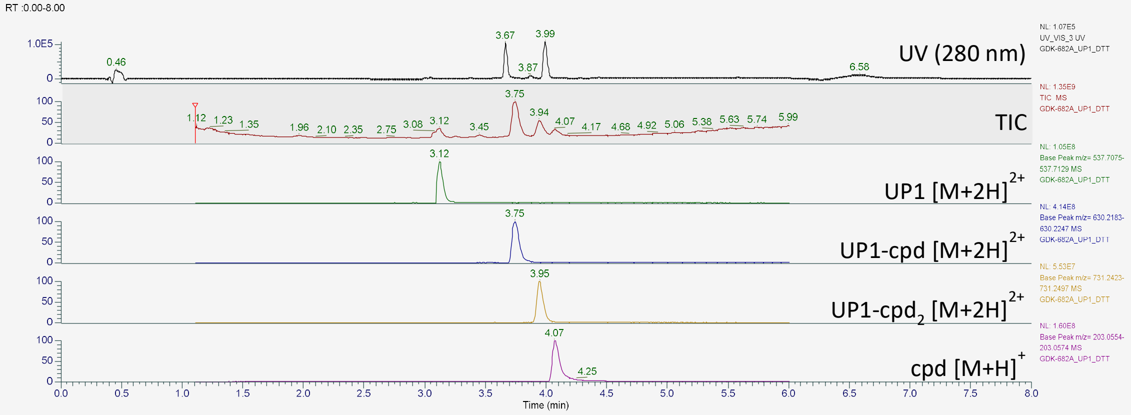

35 + UP2 (AGKGCGSGYGW)

Incubation: 60 min at 37 °C

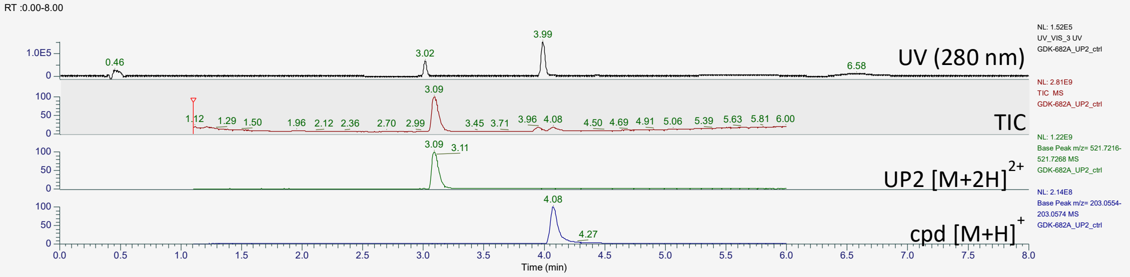

35 + UP2 (AGKGCGSGYGW)

Incubation: 30 min at 37 °C + 30 min with DTT at 37 °C

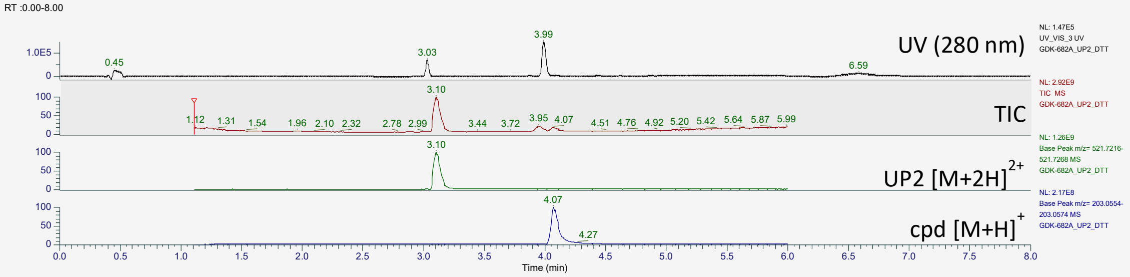

37 + UP1 (CGKGCGSGYGW):

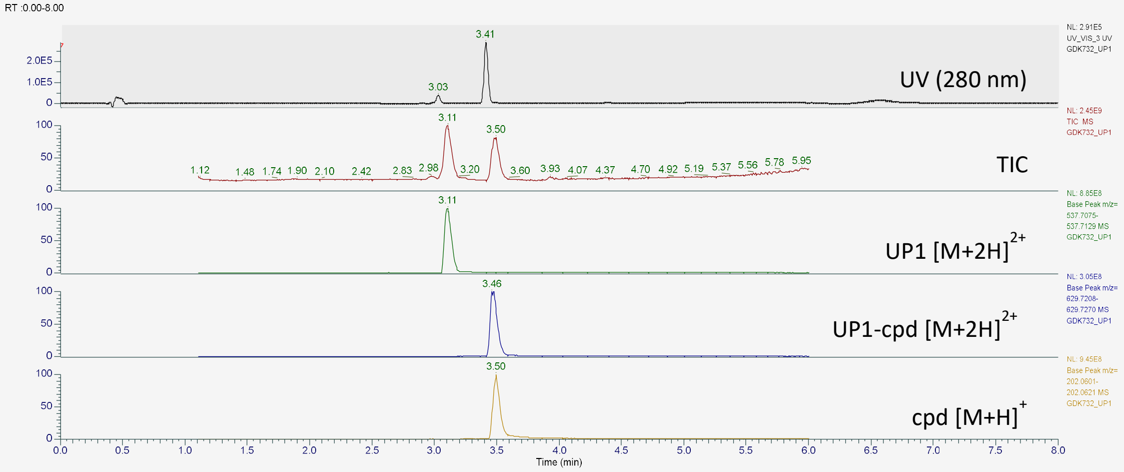

37 + UP2 (AGKGCGSGYGW):

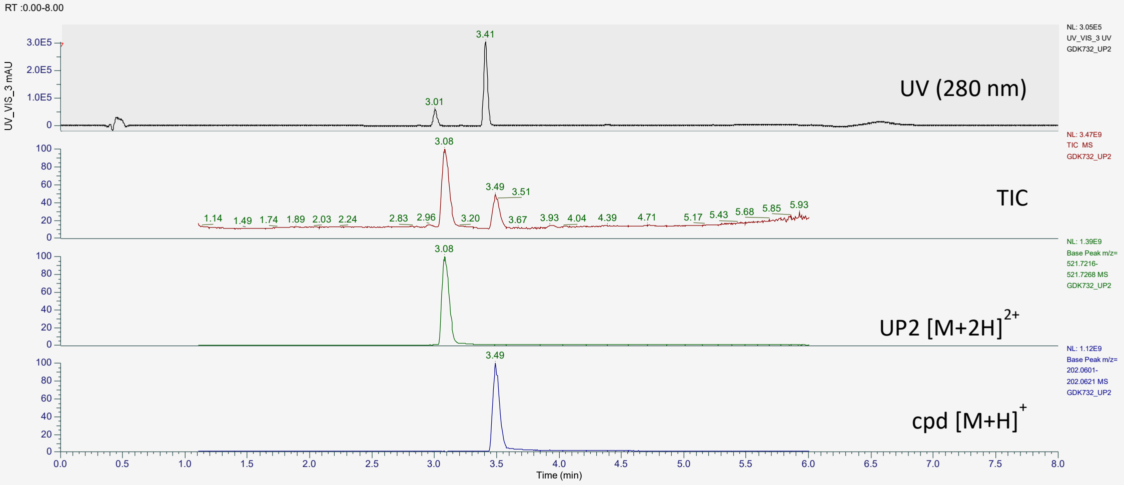

43 + UP1 (CGKGCGSGYGW):

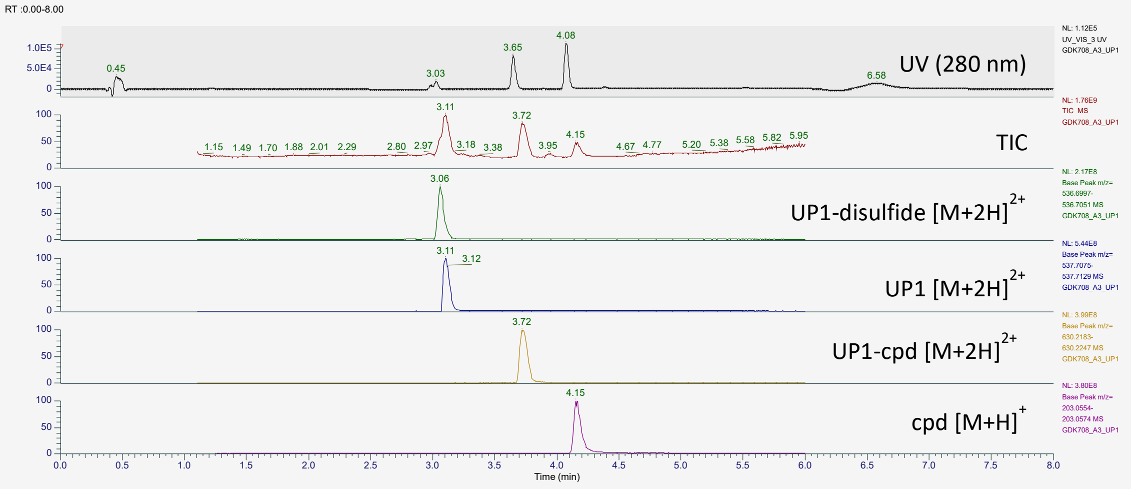

43 + UP2 (AGKGCGSGYGW):

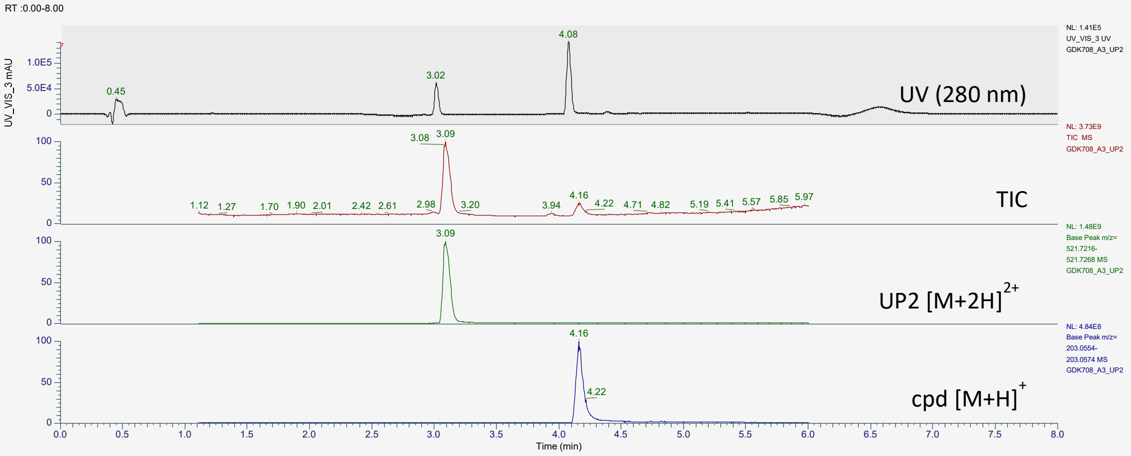

45 + UP1 (CGKGCGSGYGW):

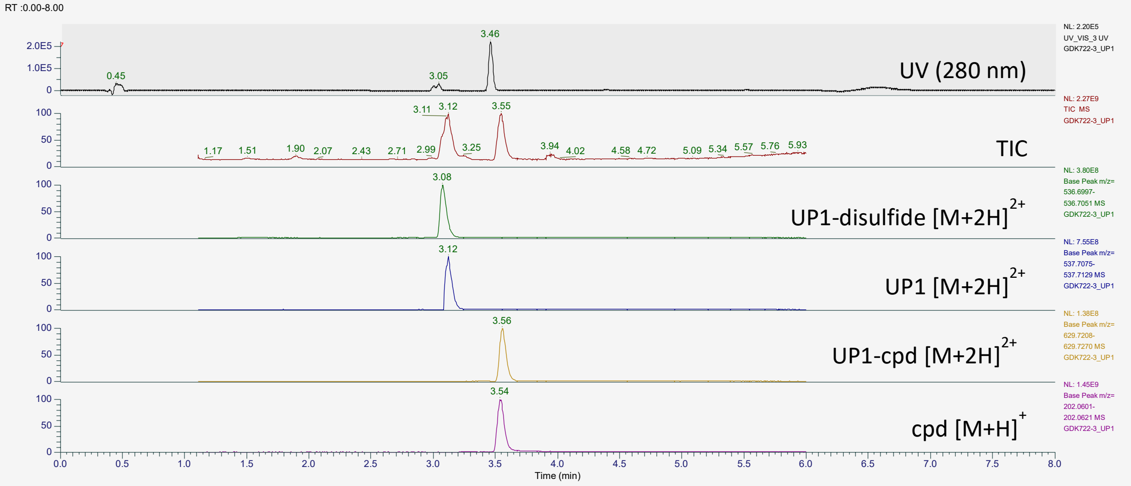

45 + UP2 (AGKGCGSGYGW):

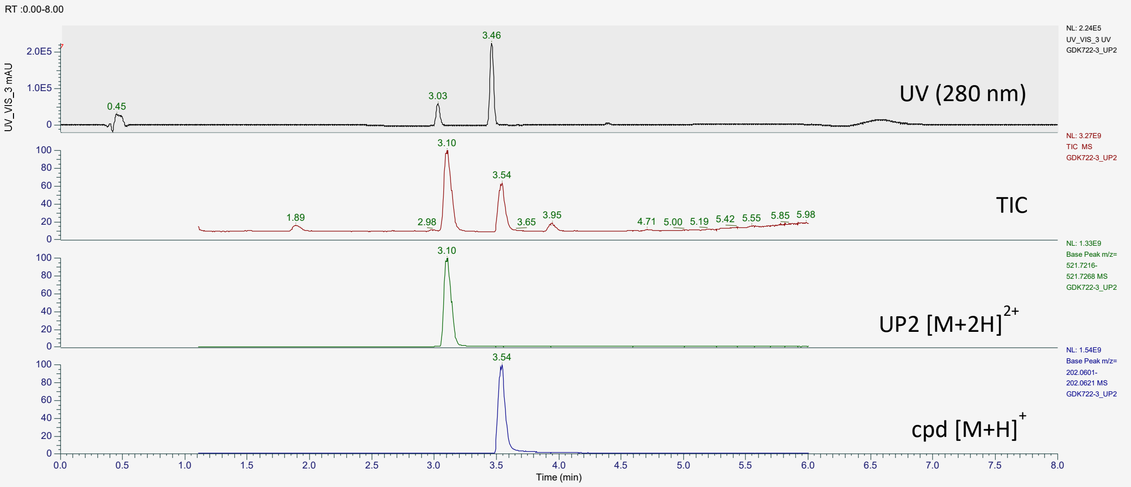

52 + UP1 (CGKGCGSGYGW):

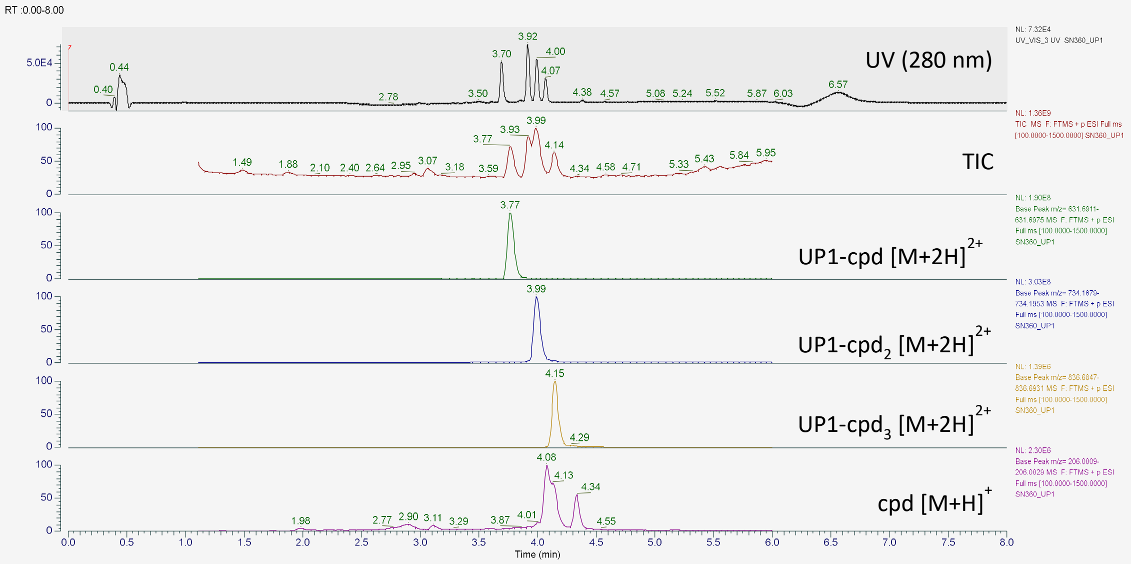

52 + UP2 (AGKGCGSGYGW):

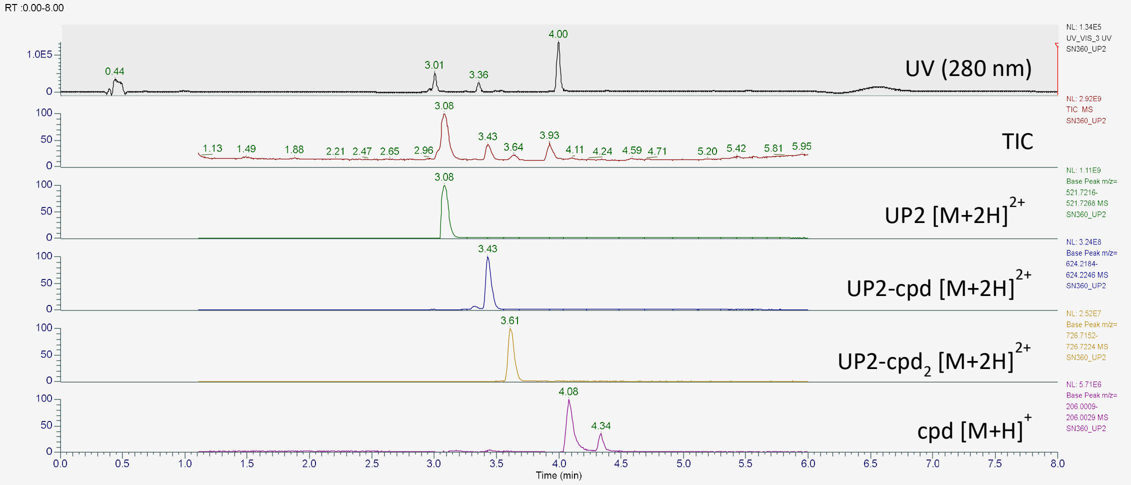

52 + UP1 (CGKGCGSGYGW)

Incubation: 60 min at 37 °C

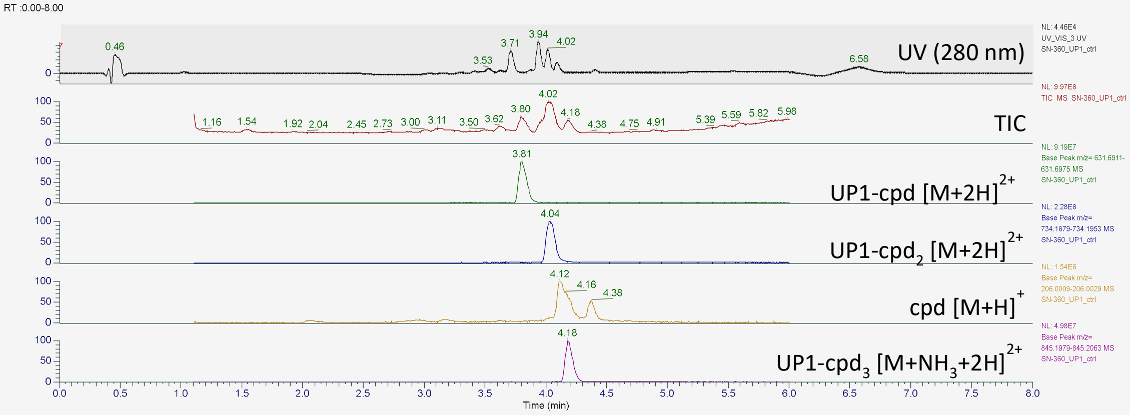

52 + UP1 (CGKGCGSGYGW)

Incubation: 30 min at 37 °C + 30 min with Cys at 37 °C

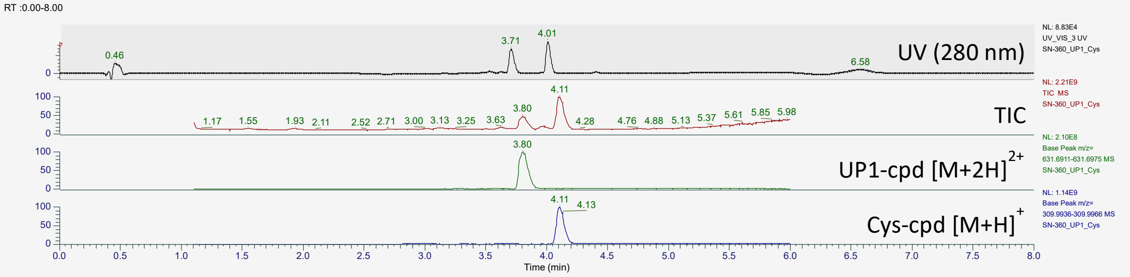

52 + UP2 (AGKGCGSGYGW)

Incubation: 60 min at 37 °C

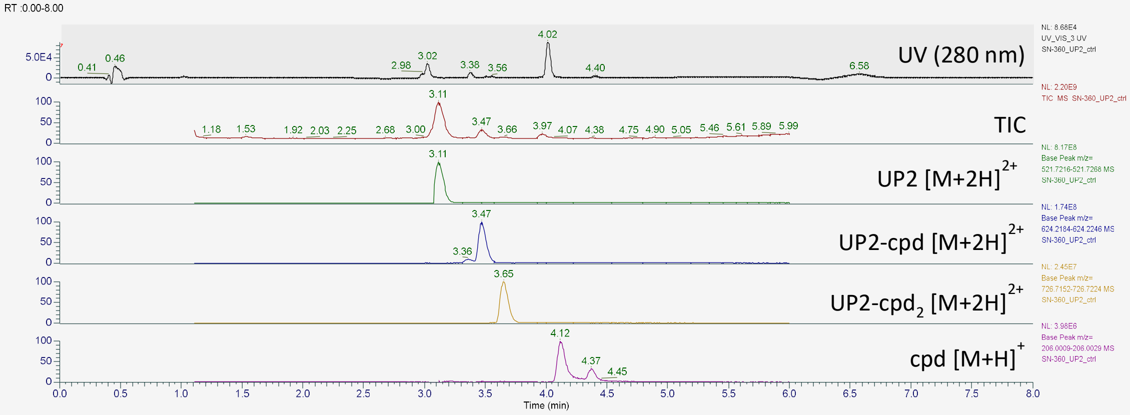

52 + UP2 (AGKGCGSGYGW)

Incubation: 30 min at 37 °C + 30 min with Cys at 37 °C

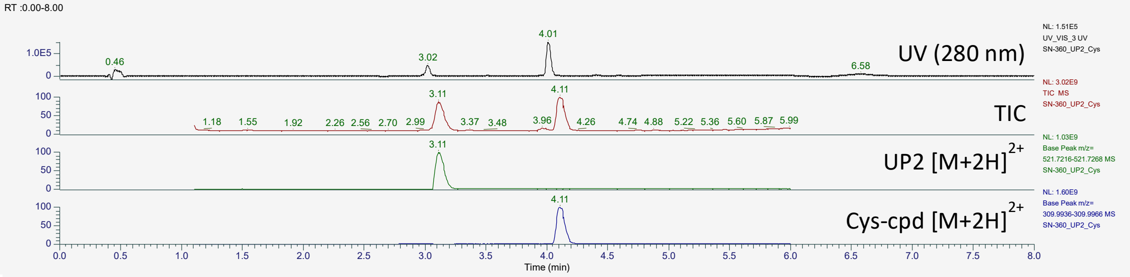

52 + UP1 (CGKGCGSGYGW)

Incubation: 60 min at 37 °C

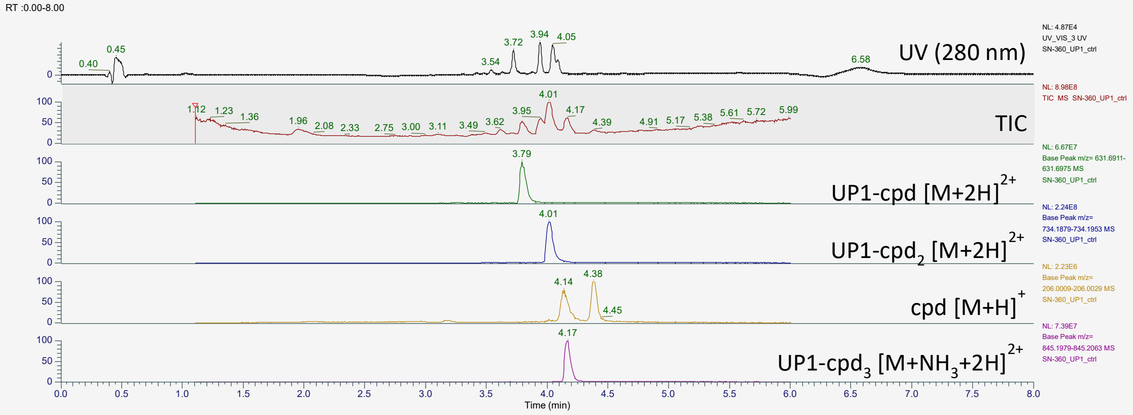

52 + UP1 (CGKGCGSGYGW)

Incubation: 30 min at 37 °C + 30 min with DTT at 37 °C

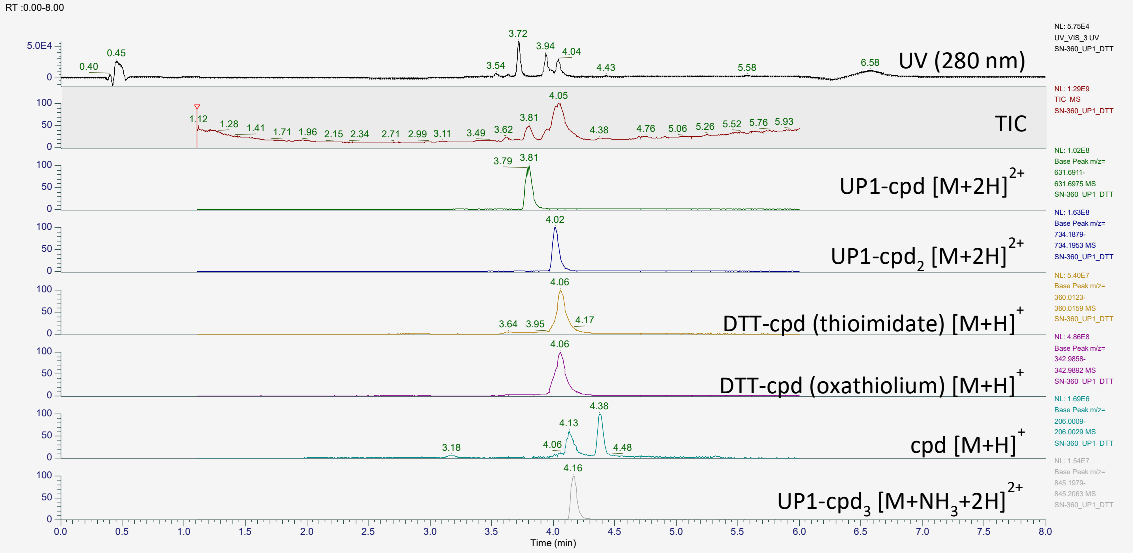

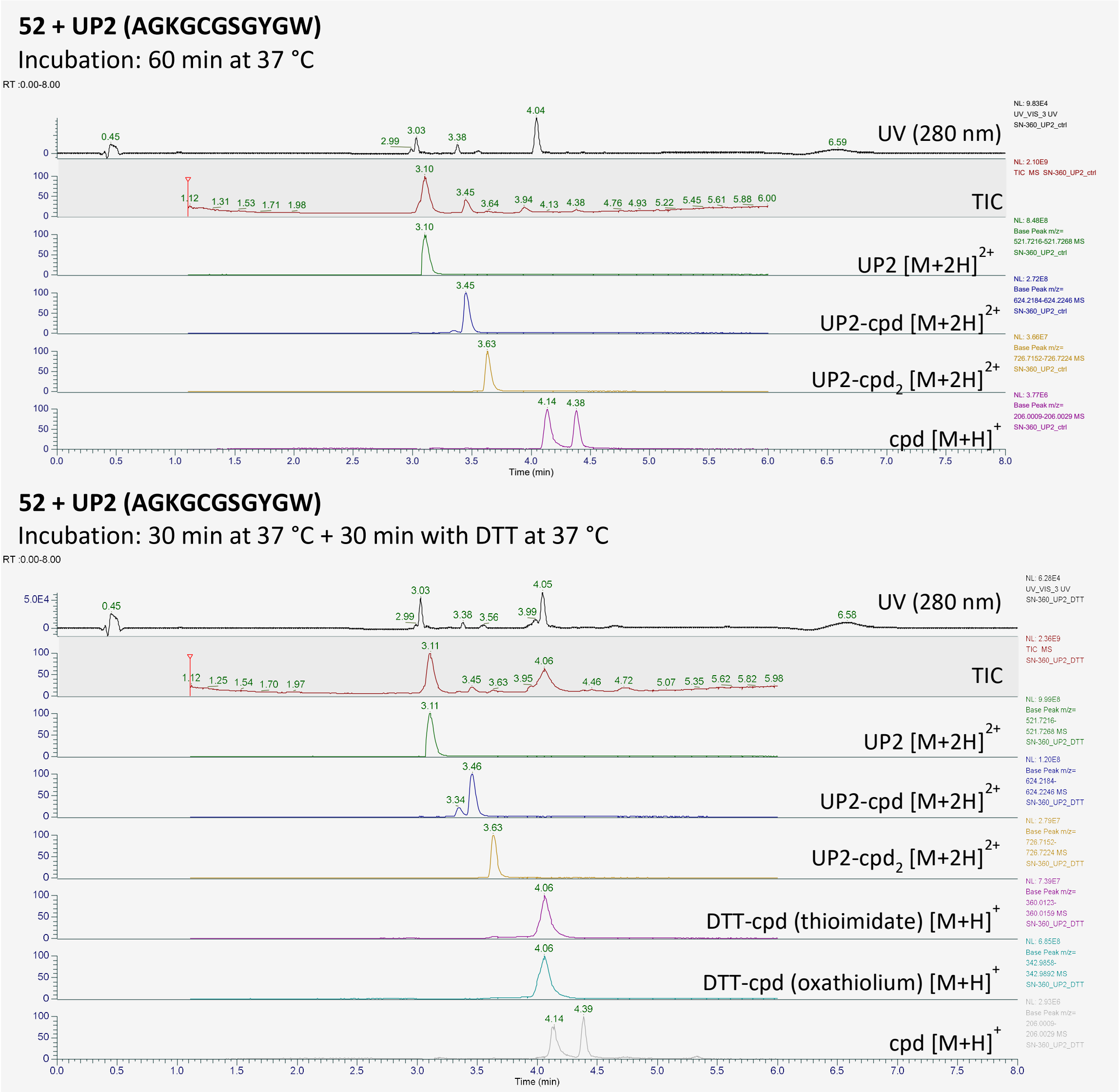
52 + UP2 (AGKGCGSGYGW)
Incubation: 60 min at 37 °C
RT :0.00-8.00
UV (280 nm)
TIC
UP2 [M+2H]2+
UP2-cpd [M+2H]2+
UP2-cpd2 [M+2H]2+
cpd [M+H]+
Time (min)
52 + UP2 (AGKGCGSGYGW)
Incubation: 30 min at 37 °C + 30 min with DTT at 37 °C
RT :0.00-8.00
UV (280 nm)
TIC
UP2 [M+2H]2+
UP2-cpd [M+2H]2+
UP2-cpd2 [M+2H]2+
DTT-cpd (thioimidate) [M+H]+
DTT-cpd (oxathiolium) [M+H]+
cpd [M+H]+
Time (min)

52 (1 eq.) + UP1 (CGKGCGSGYGW):

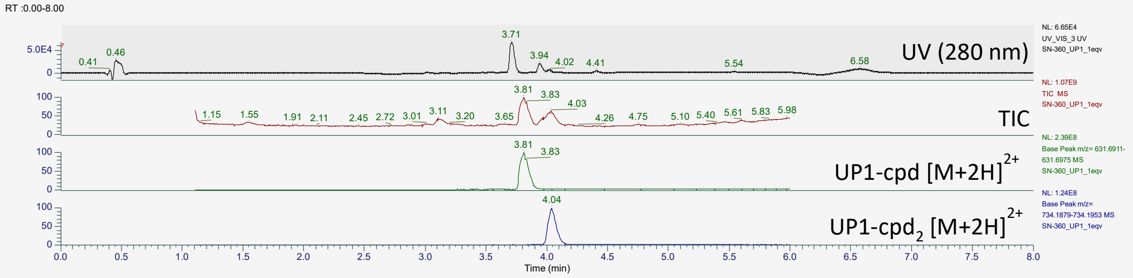

52 (1 eq.) + UP2 (AGKGCGSGYGW):

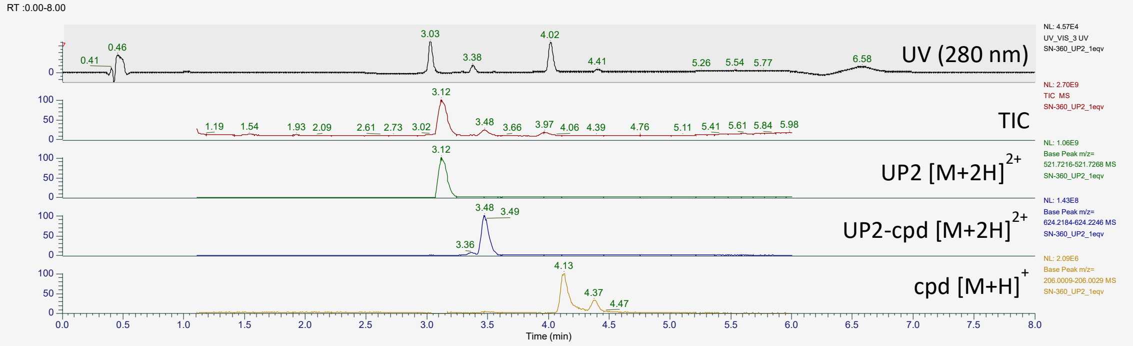

53 + UP1 (CGKGCGSGYGW):

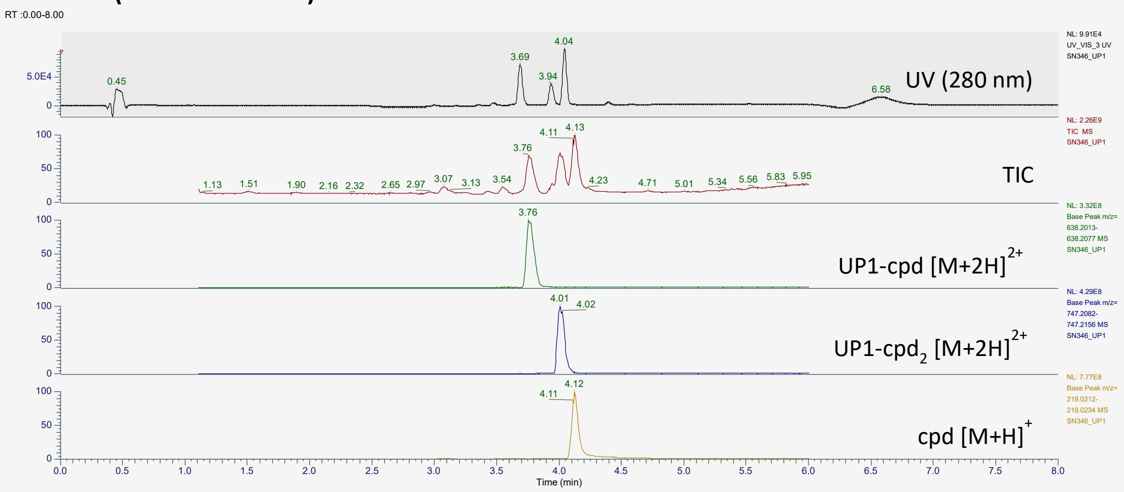

53 + UP2 (AGKGCGSGYGW):

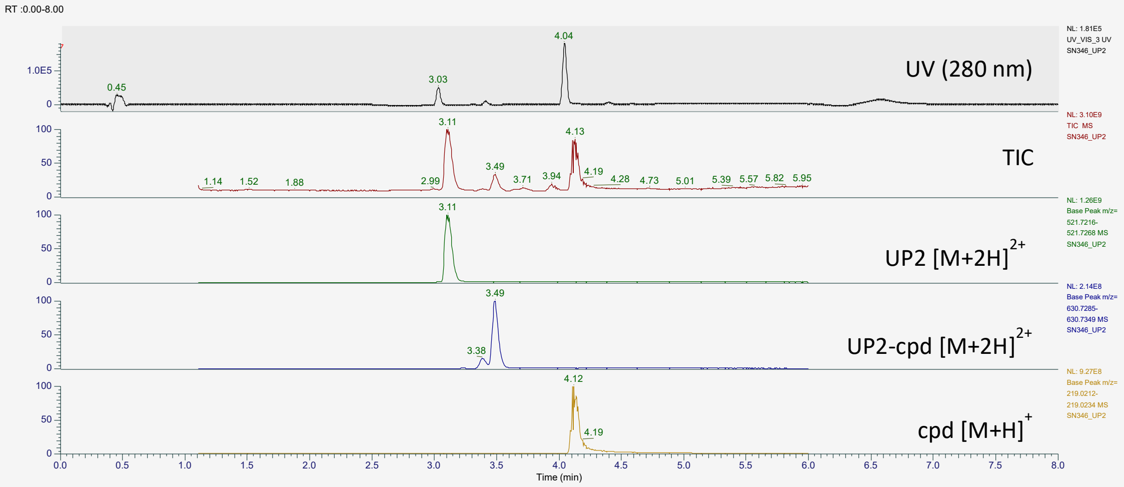

57 + UP1 (CGKGCGSGYGW):

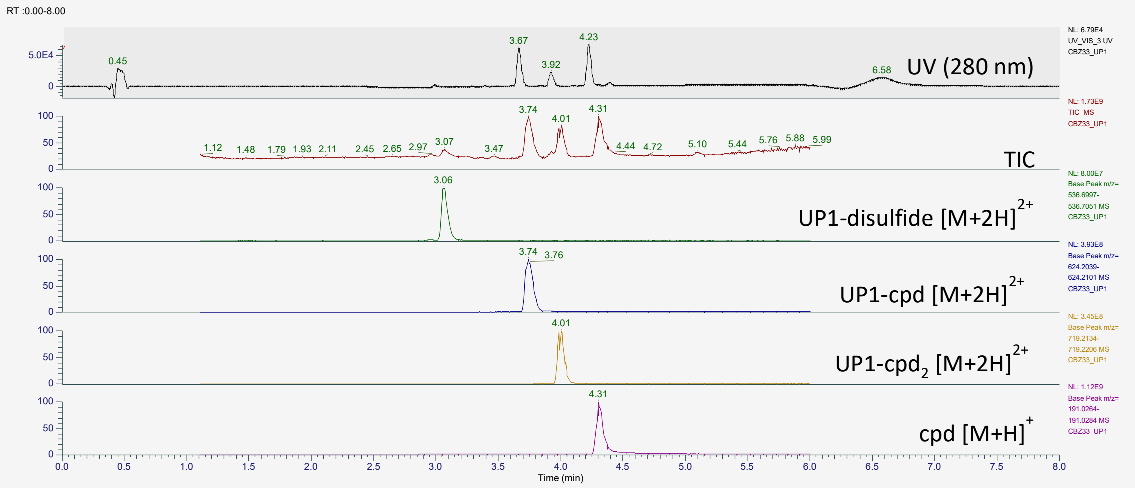

57 + UP2 (AGKGCGSGYGW):

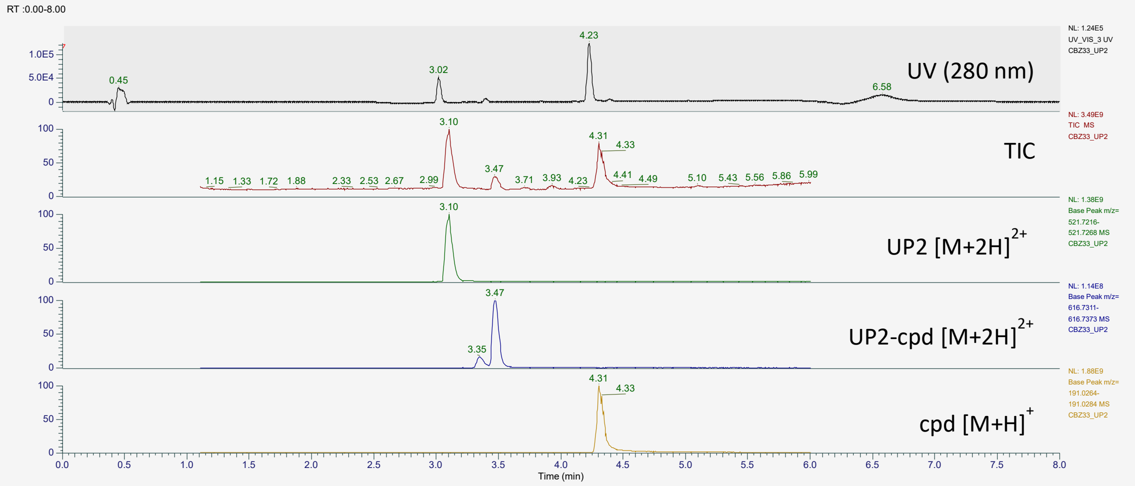

58 + UP1 (CGKGCGSGYGW):

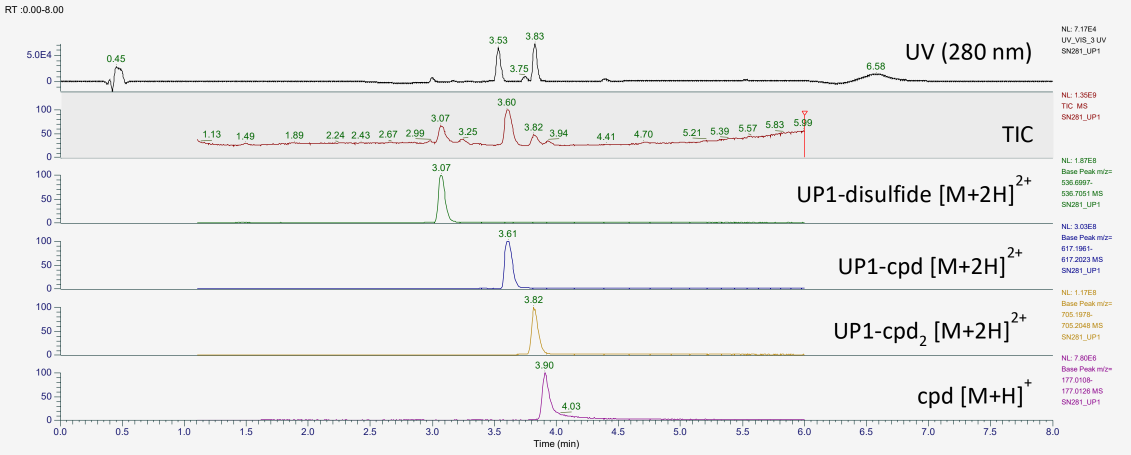

58 + UP2 (AGKGCGSGYGW):

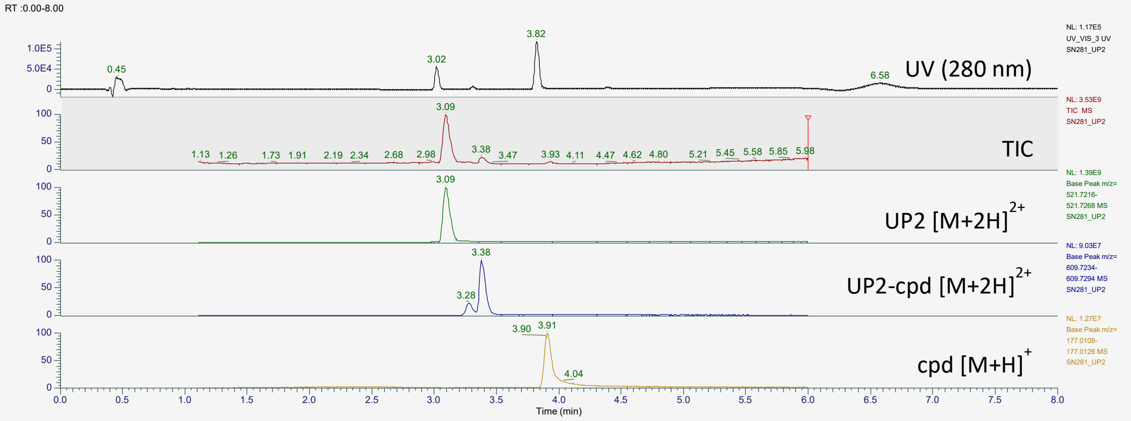

59 + UP1 (CGKGCGSGYGW):

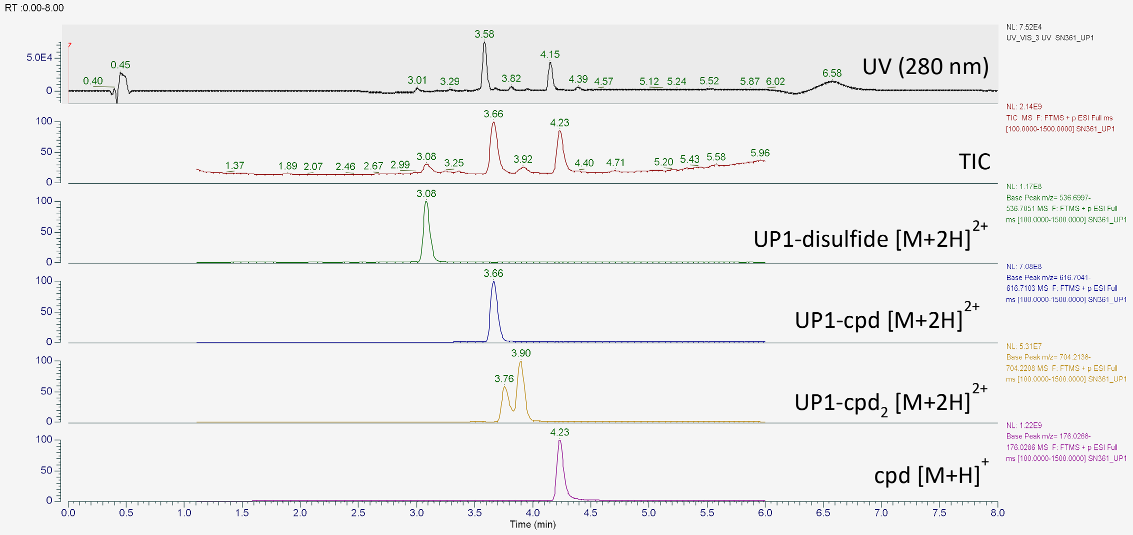

59 + UP2 (AGKGCGSGYGW):

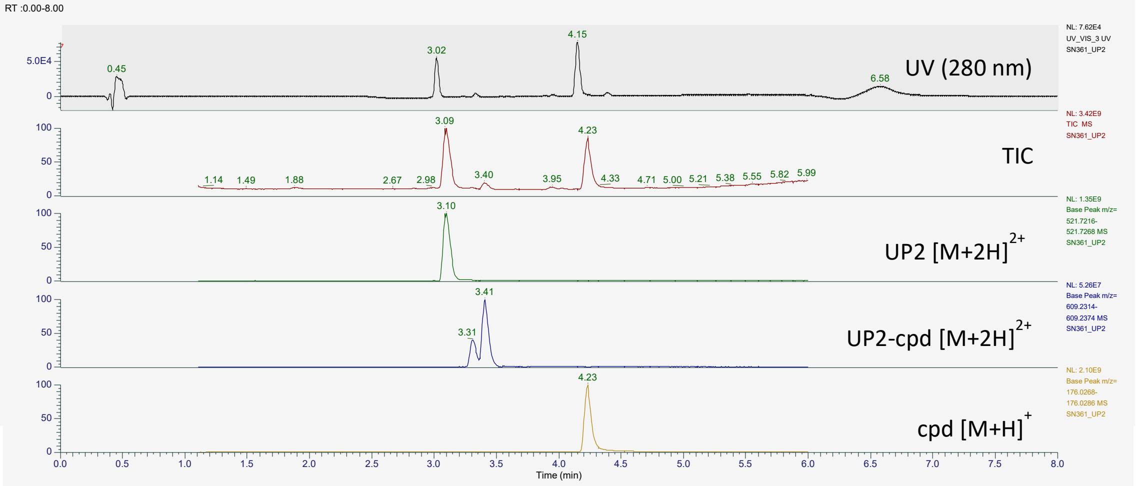

61 + UP1 (CGKGCGSGYGW):

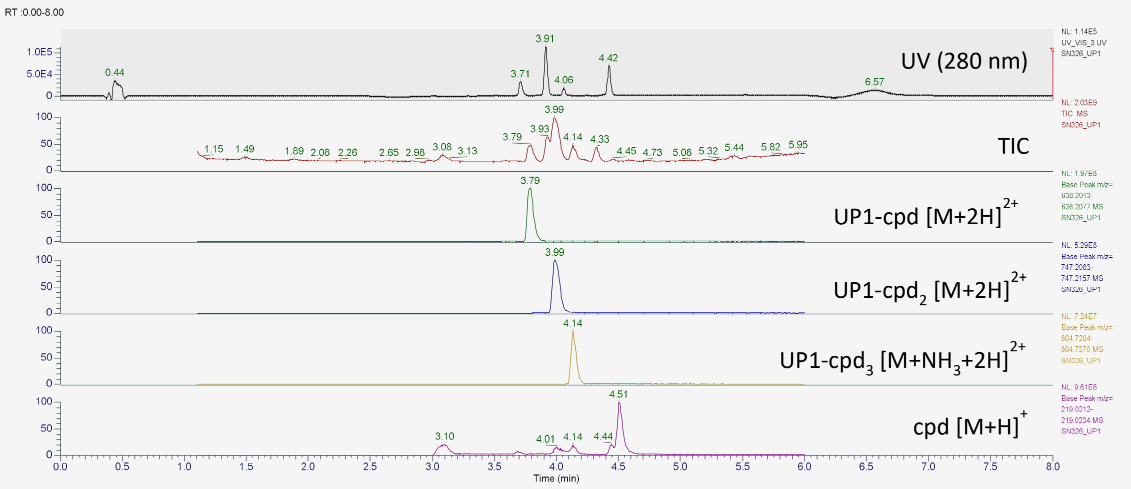

61 + UP2 (AGKGCGSGYGW):

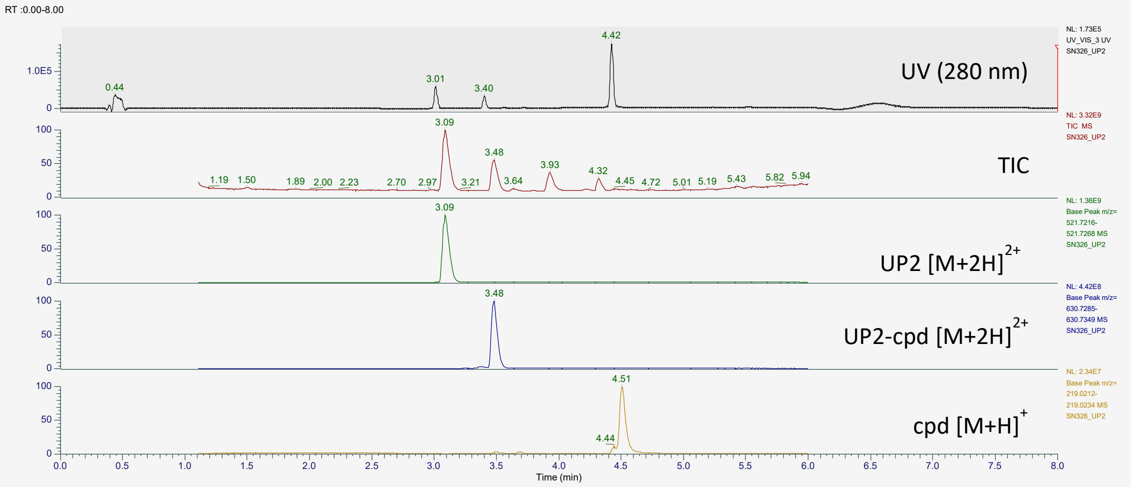

62 + UP1 (CGKGCGSGYGW):

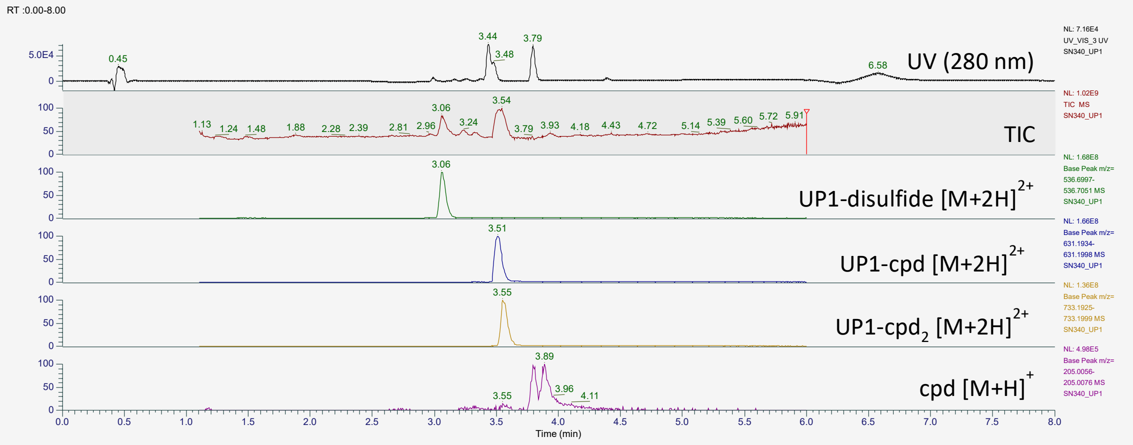

62 + UP2 (AGKGCGSGYGW):

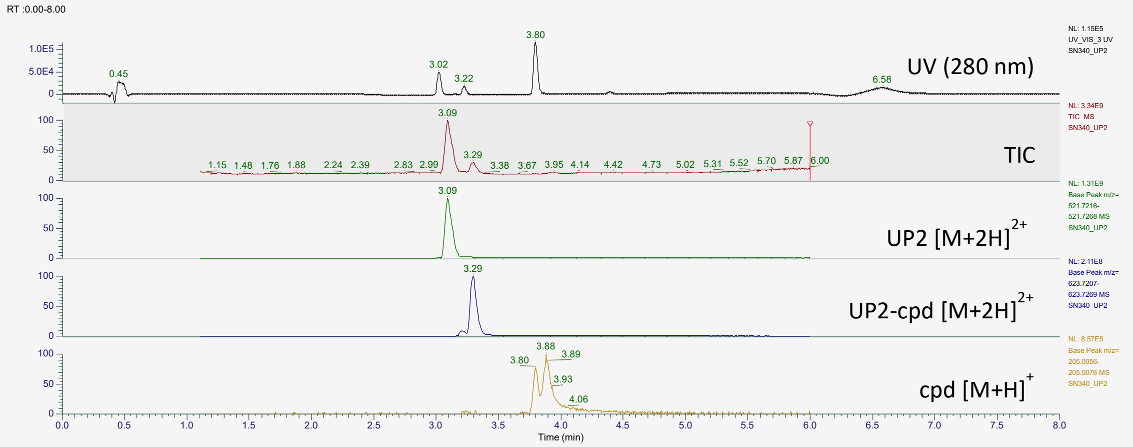

65 + UP1 (CGKGCGSGYGW):

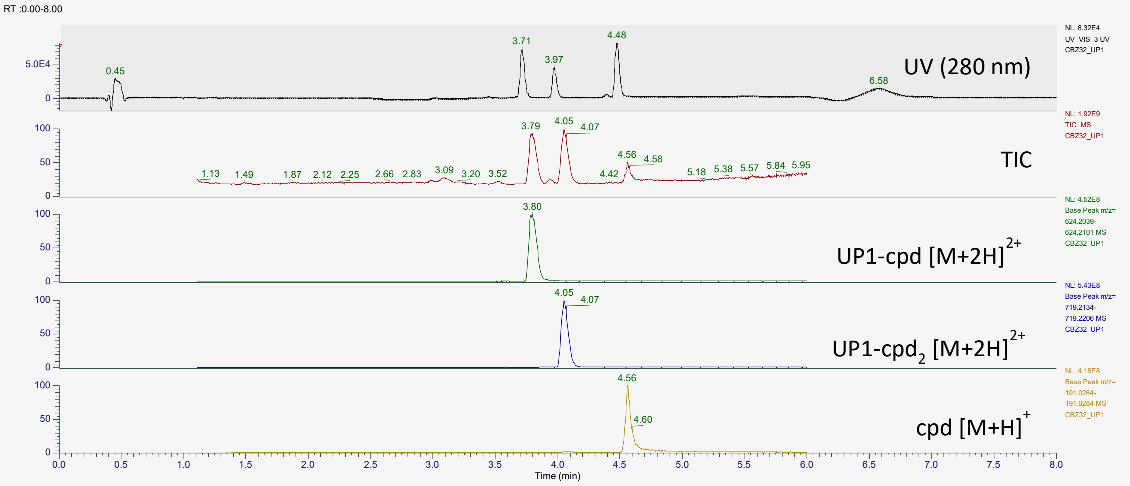

65 + UP2 (AGKGCGSGYGW):

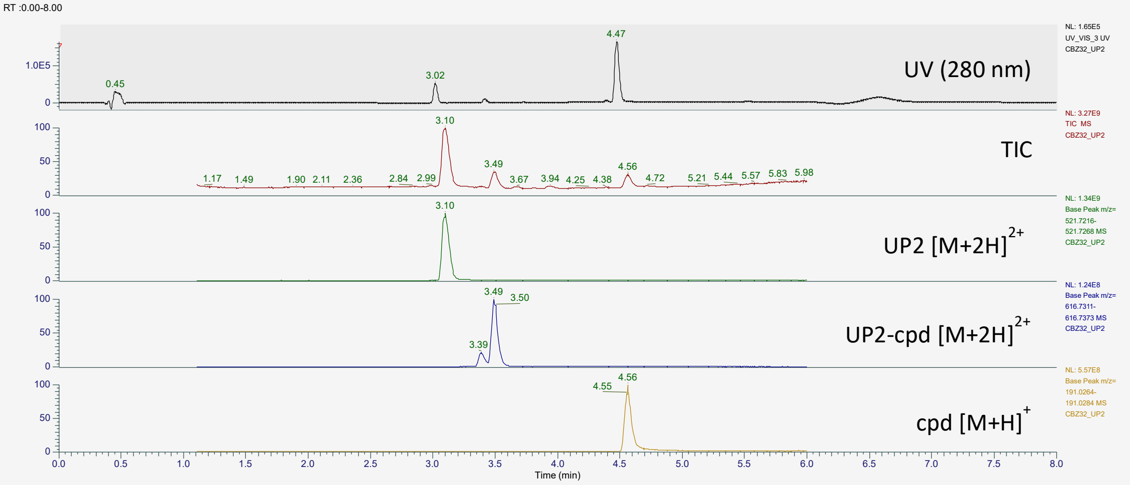

66 + UP1 (CGKGCGSGYGW):

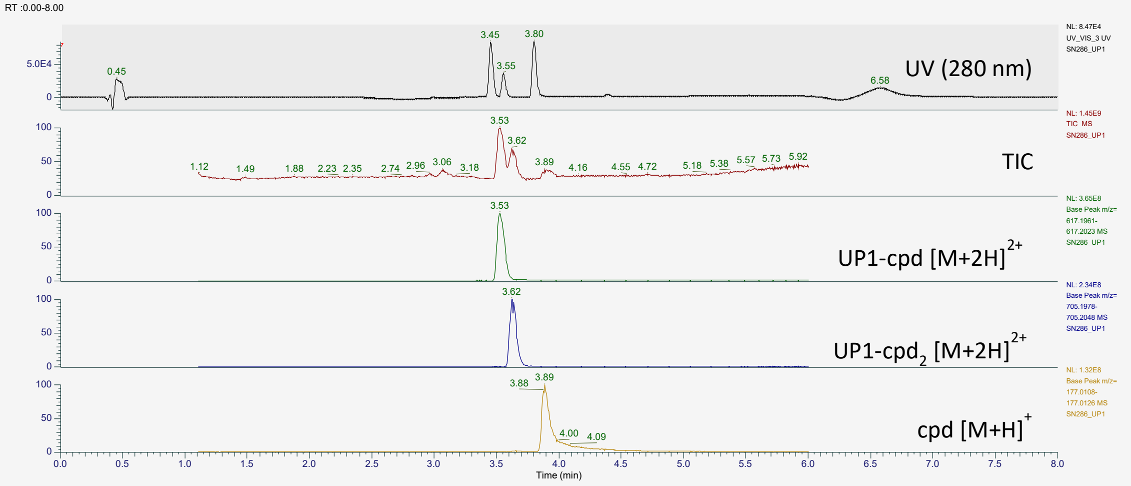

66 + UP2 (AGKGCGSGYGW):

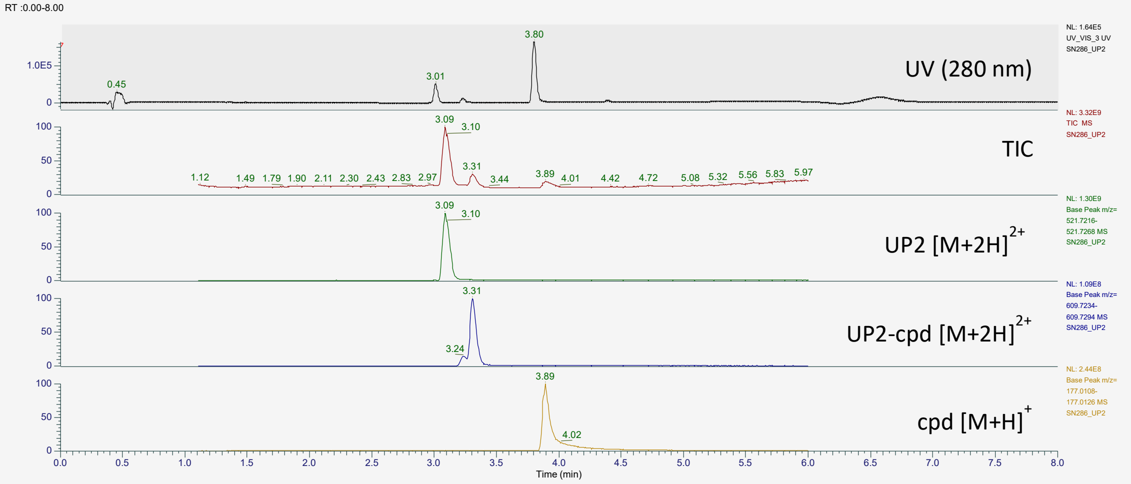

67 + UP1 (CGKGCGSGYGW):

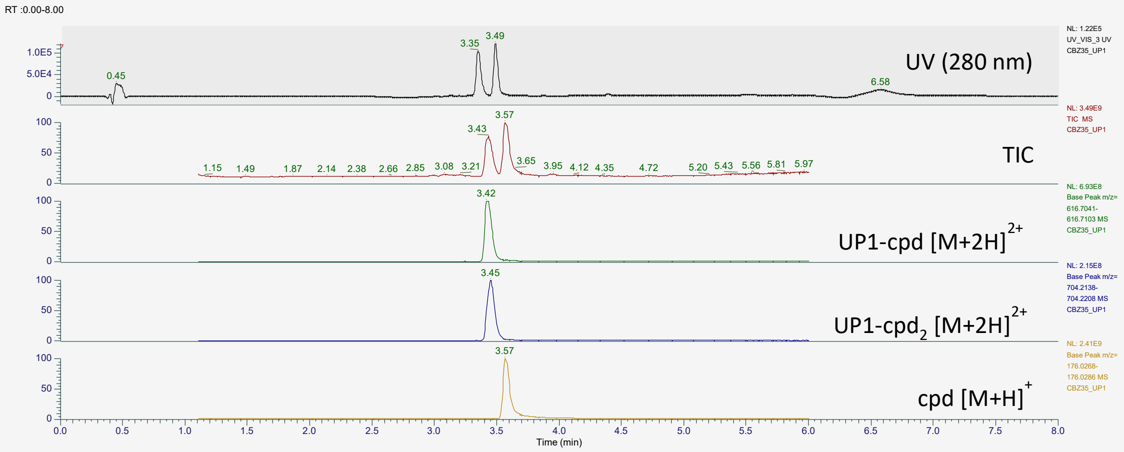

67 + UP2 (AGKGCGSGYGW):

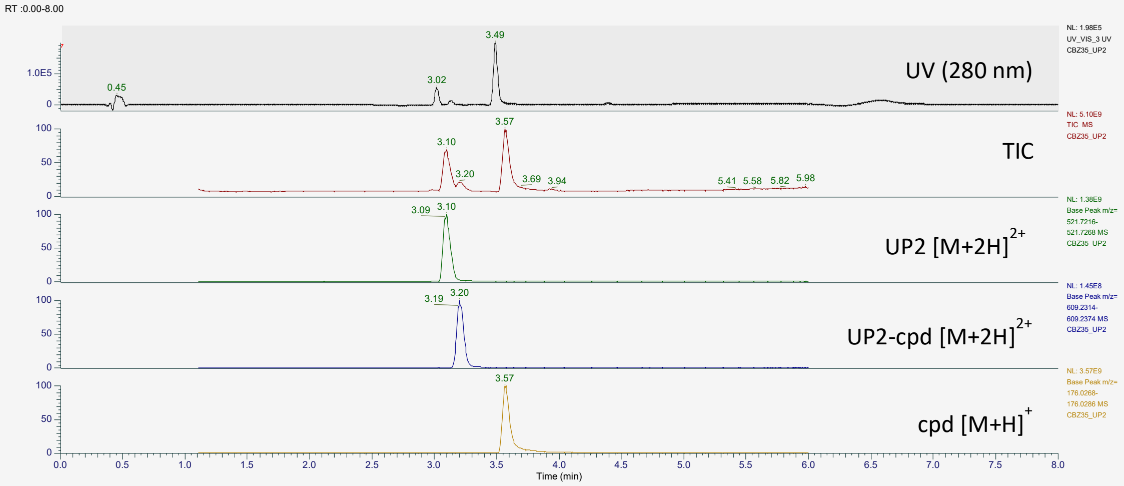

69 + UP1 (CGKGCGSGYGW):

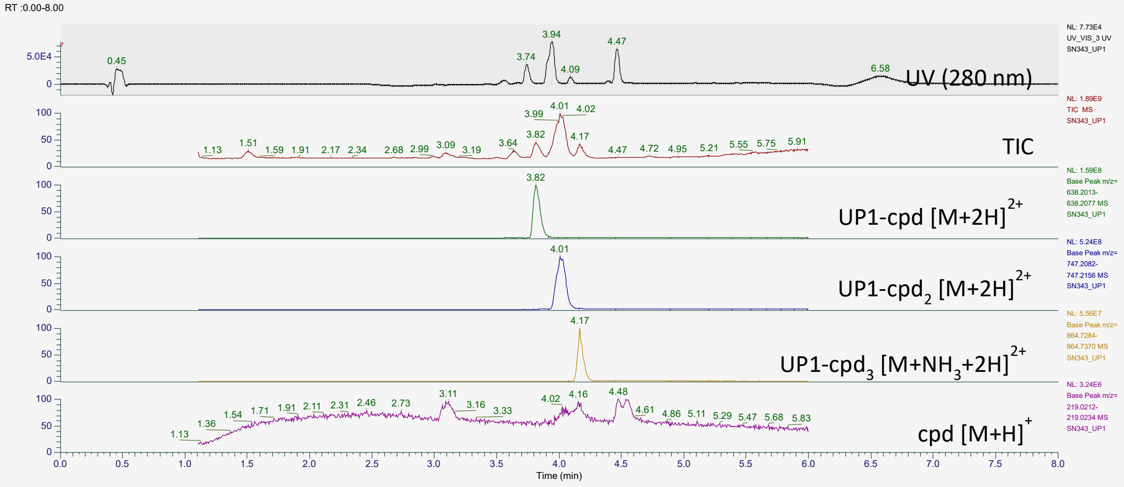

69 + UP2 (AGKGCGSGYGW):

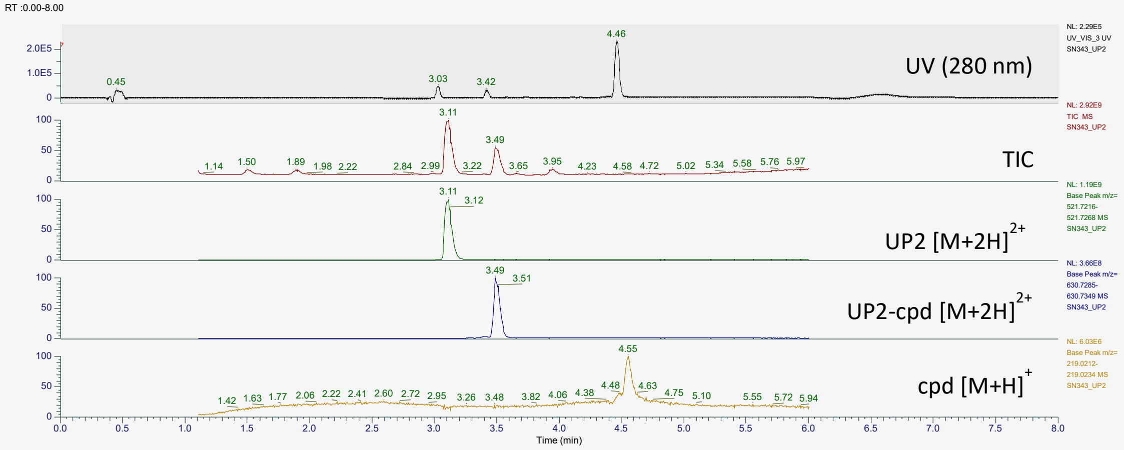

70 + UP1 (CGKGCGSGYGW):

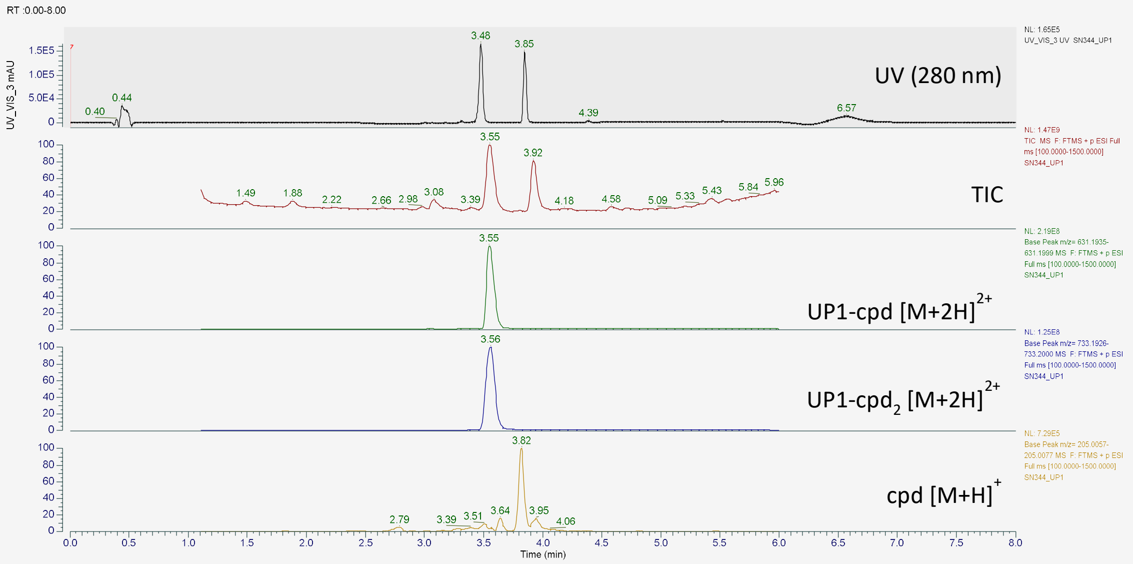

70 + UP2 (AGKGCGSGYGW):

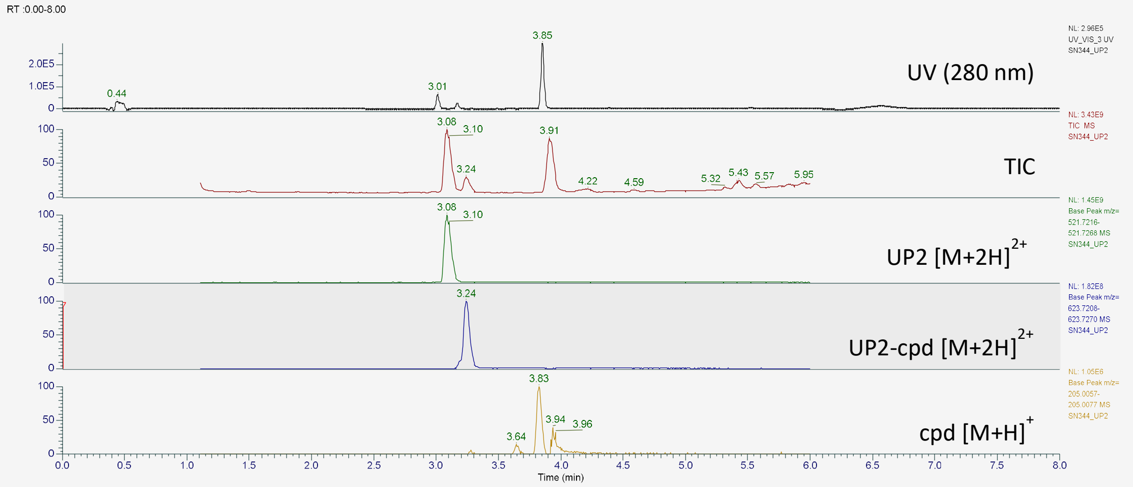

73 + UP1 (CGKGCGSGYGW):

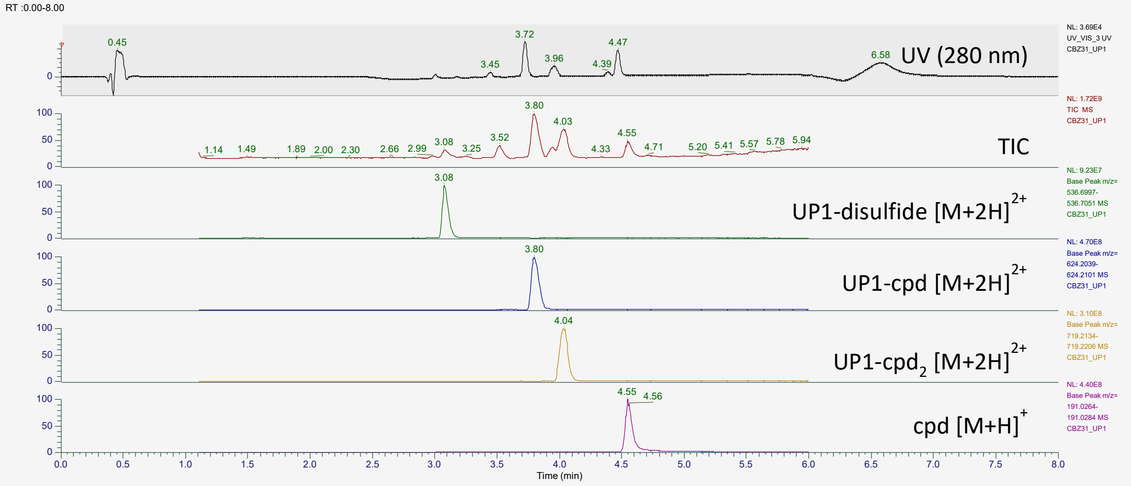

73 + UP2 (AGKGCGSGYGW):

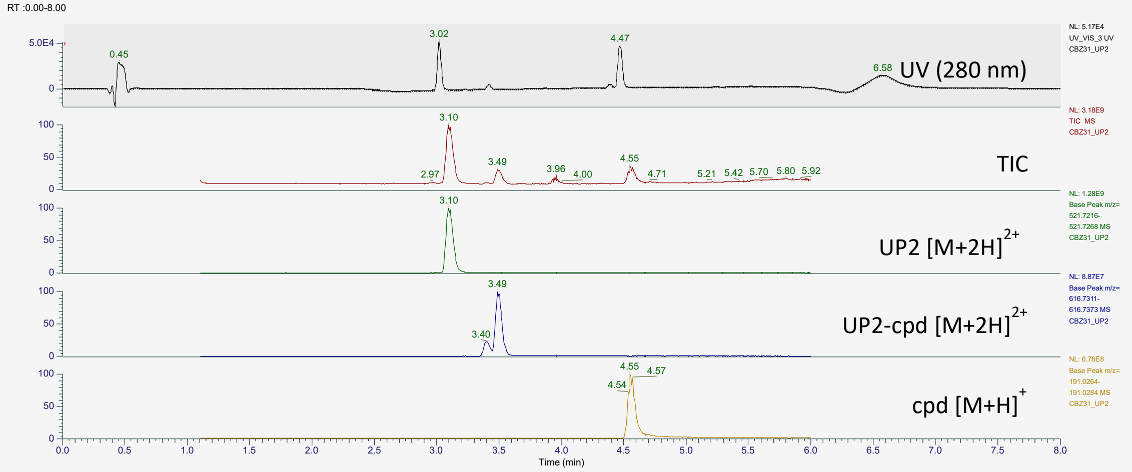

74 + UP1 (CGKGCGSGYGW):

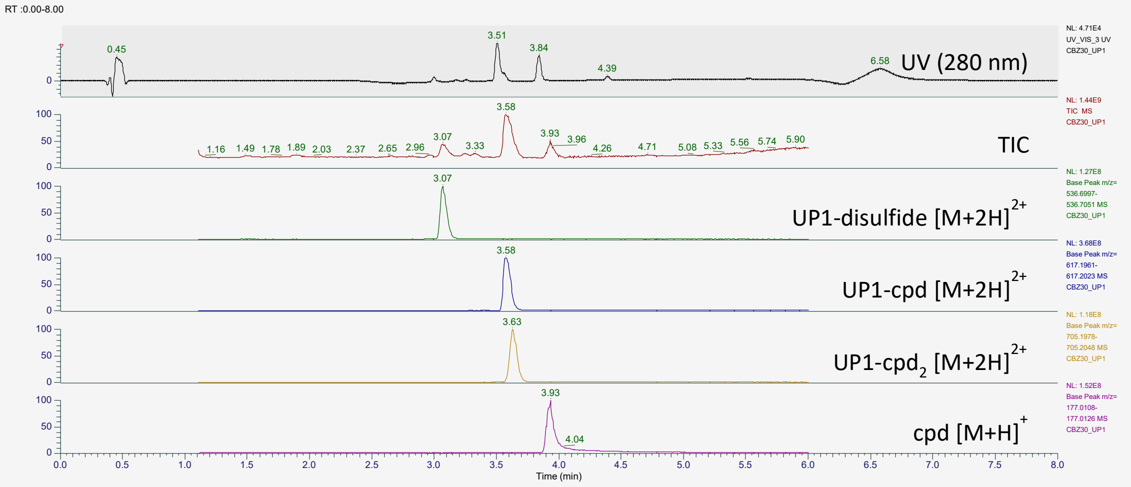

74 + UP2 (AGKGCGSGYGW):

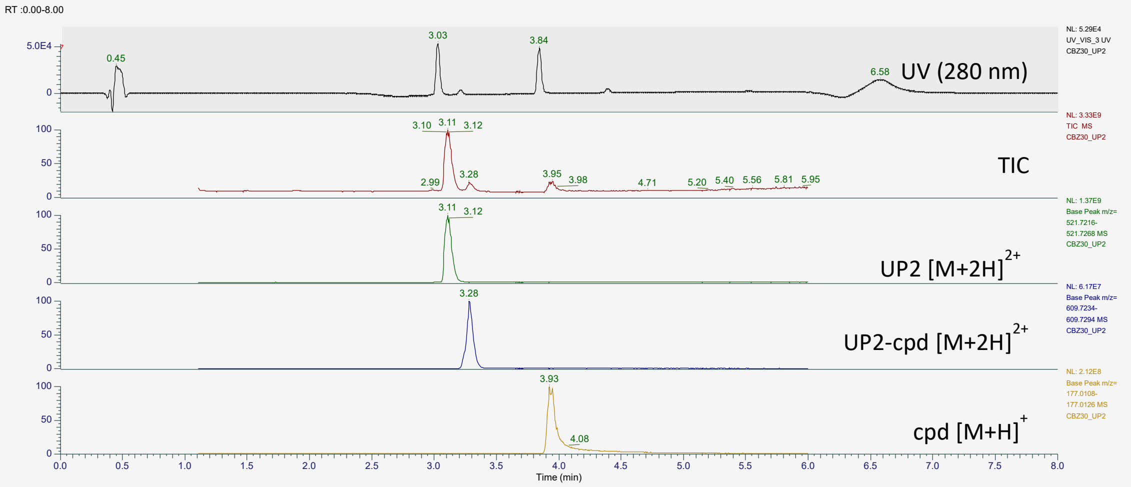

75 + UP1 (CGKGCGSGYGW):

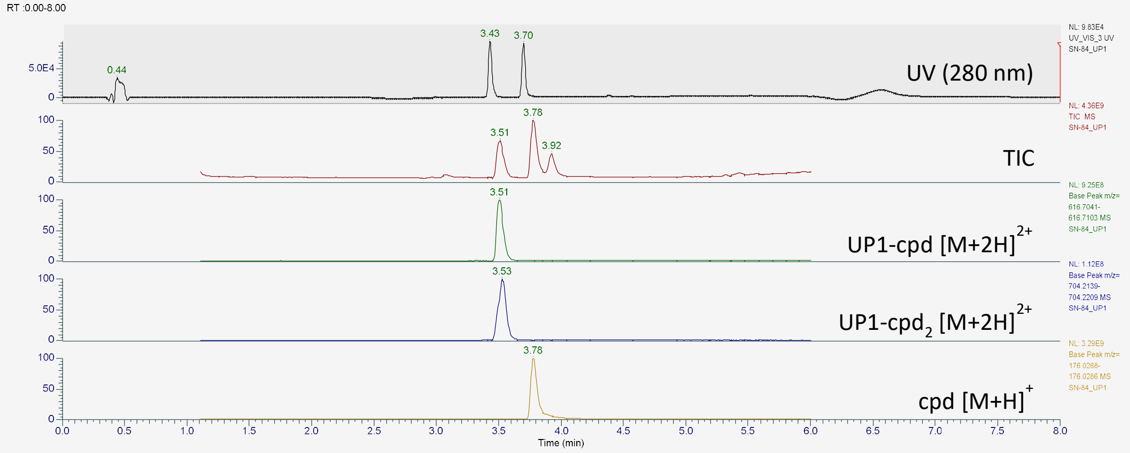

75 + UP2 (AGKGCGSGYGW):

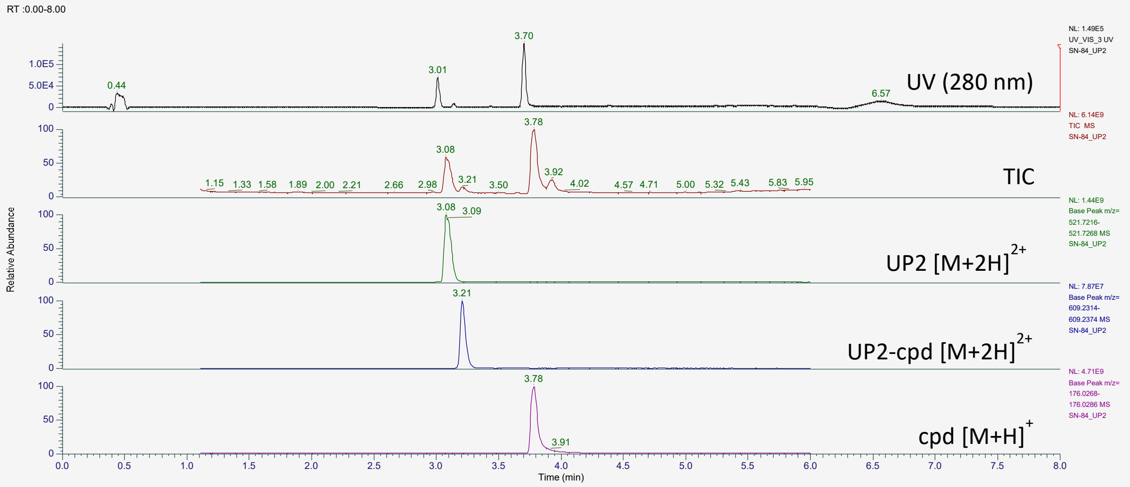

77 + UP1 (CGKGCGSGYGW):

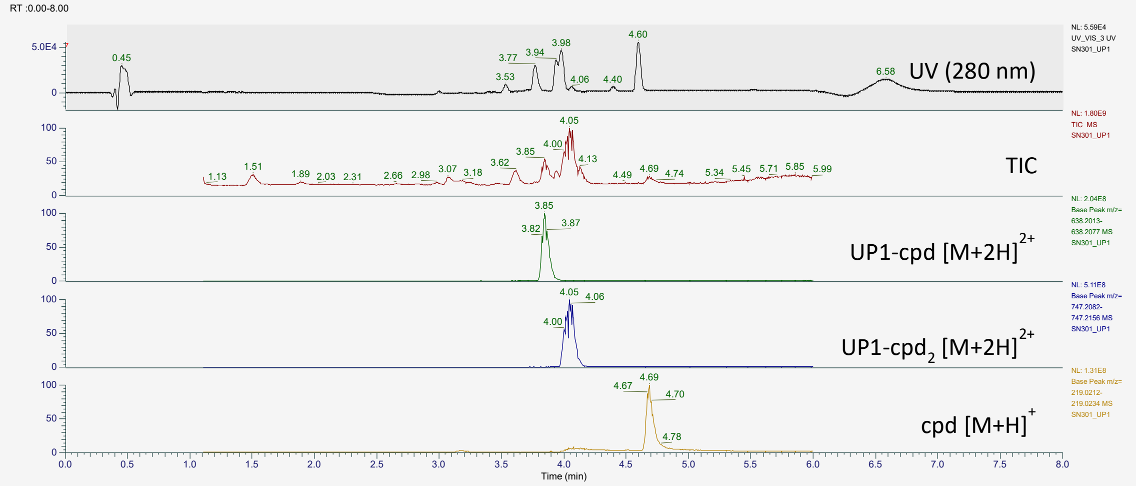

77 + UP2 (AGKGCGSGYGW):

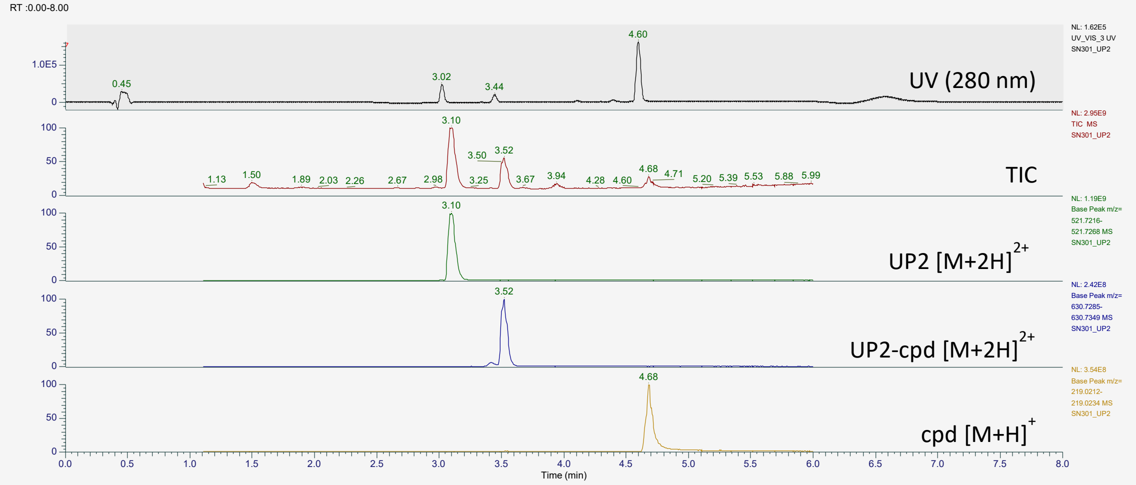

78 + UP1 (CGKGCGSGYGW):

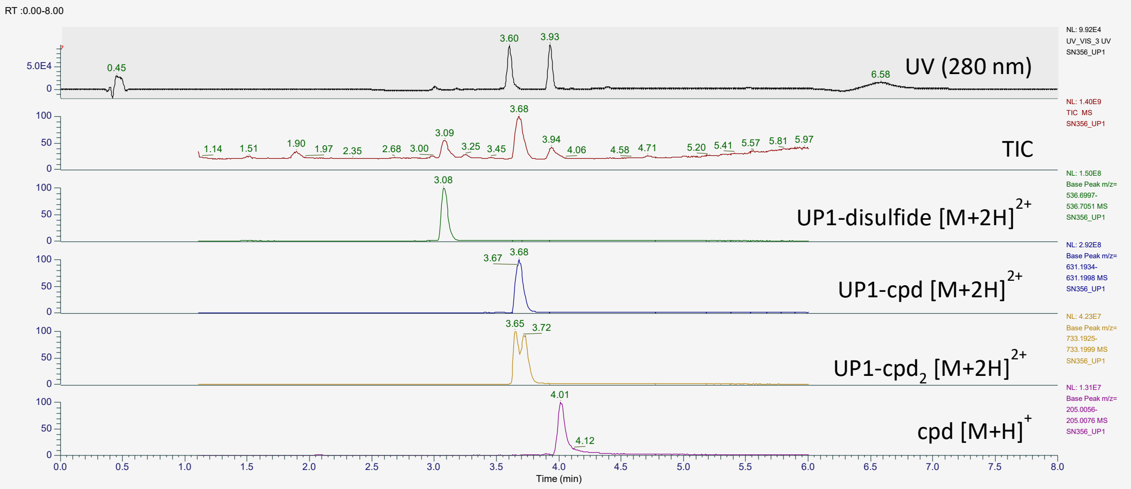

78 + UP2 (AGKGCGSGYGW):

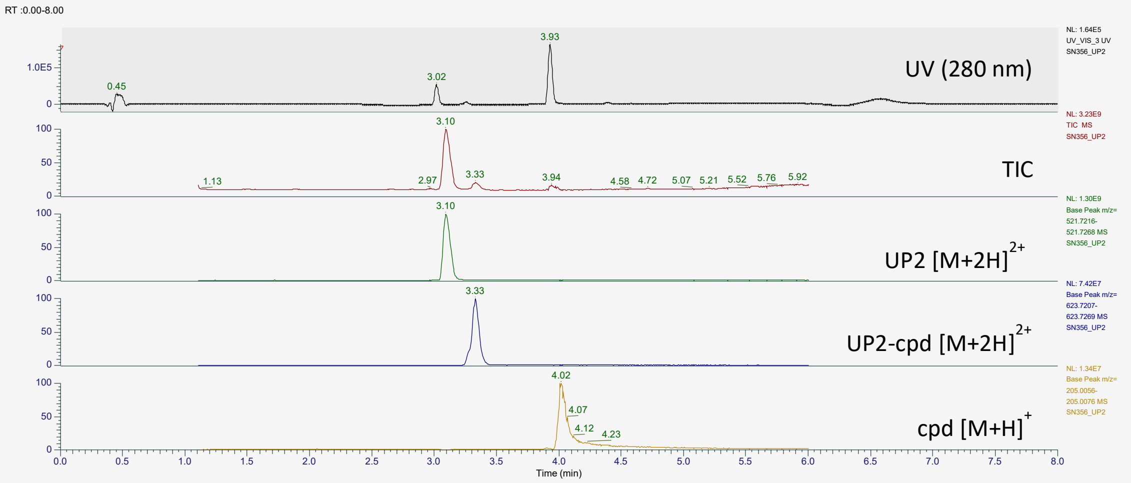

82 + UP1 (CGKGCGSGYGW):

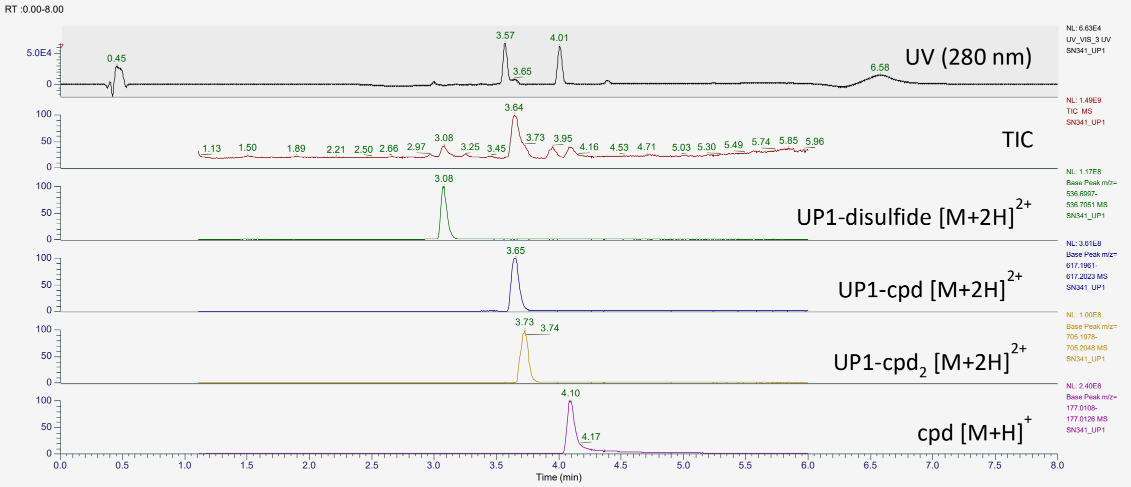

82 + UP2 (AGKGCGSGYGW):

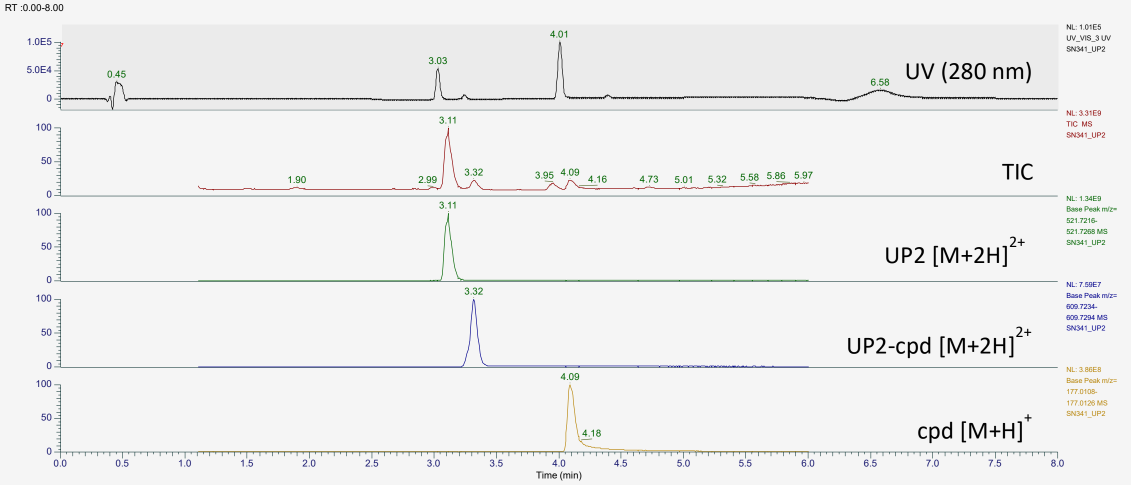

83 + UP1 (CGKGCGSGYGW):

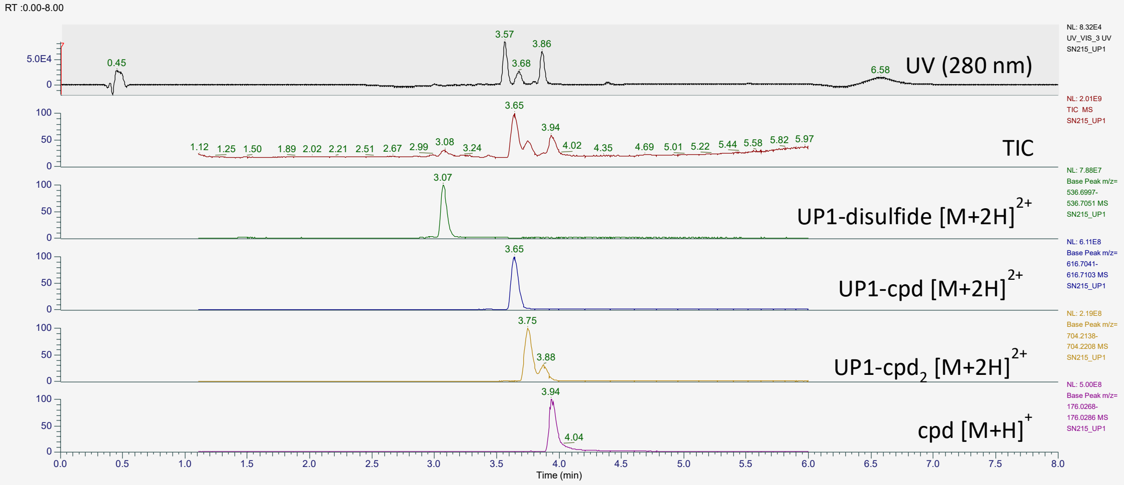

83 + UP2 (AGKGCGSGYGW):

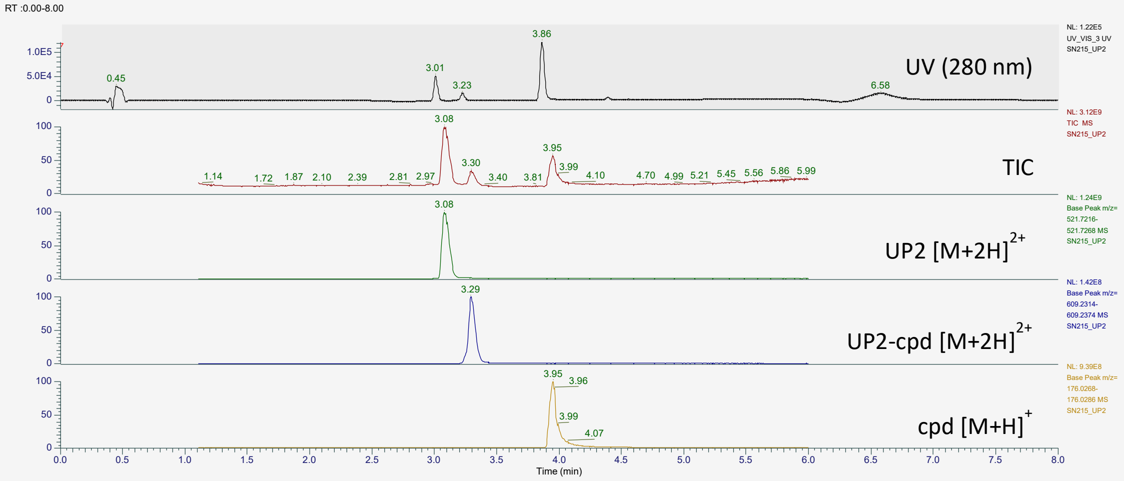

83 + UP1 (CGKGCGSGYGW)

Incubation: 60 min at 37 °C

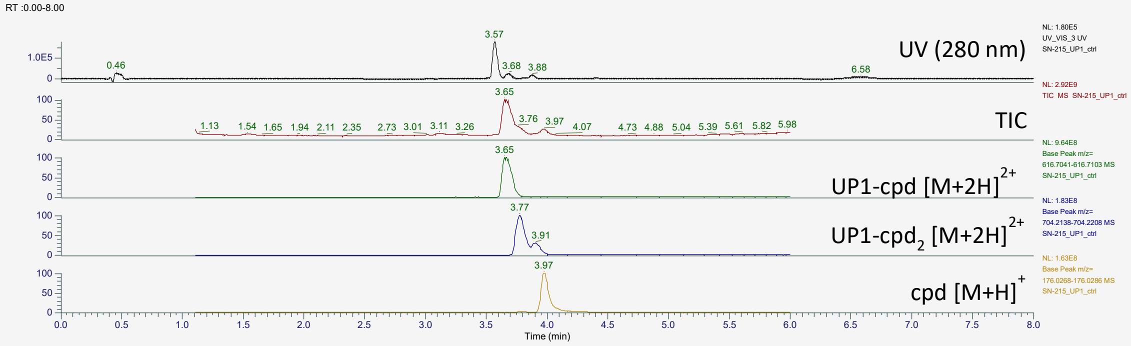

83 + UP1 (CGKGCGSGYGW)

Incubation: 30 min at 37 °C + 30 min with Cys at 37 °C

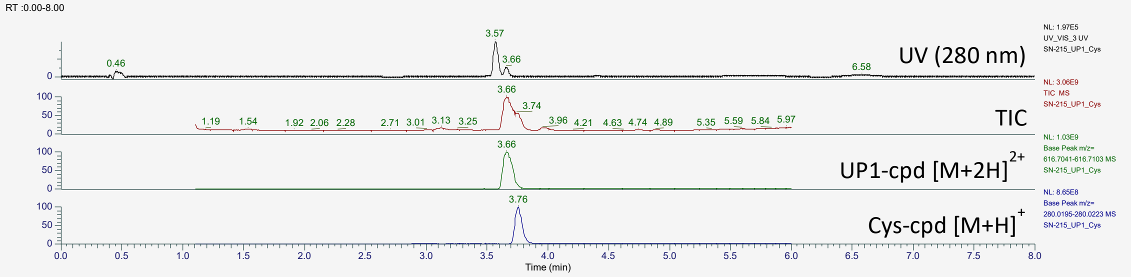

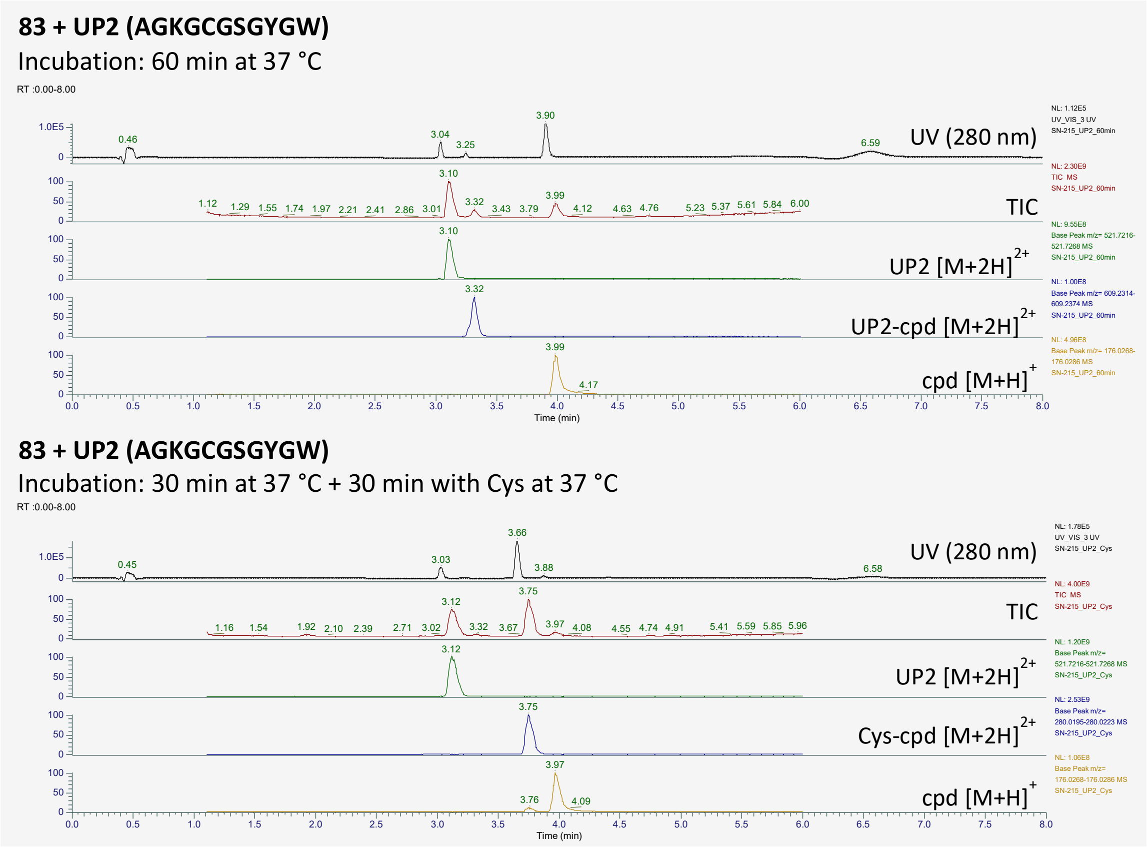
83 + UP2 (AGKGCGSGYGW)
Incubation: 60 min at 37 °C
RT :0.00-8.00
UV (280 nm)
TIC
UP2 [M+2H]2+
UP2-cpd [M+2H]2+
cpd [M+H]+
Time (min)
83 + UP2 (AGKGCGSGYGW)
Incubation: 30 min at 37 °C + 30 min with Cys at 37 °C
RT :0.00-8.00
UV (280 nm)
TIC
UP2 [M+2H]2+
Cys-cpd [M+2H]2+
cpd [M+H]+
Time (min)

s

83 + UP1 (CGKGCGSGYGW)

Incubation: 60 min at 37 °C

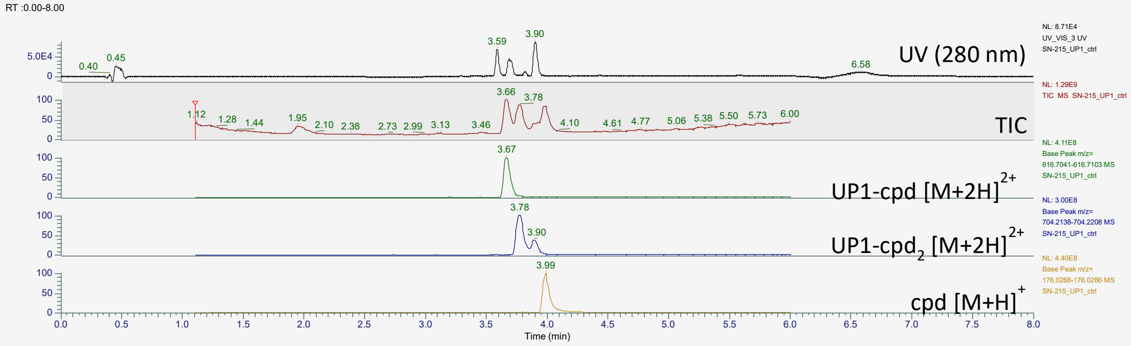

83 + UP1 (CGKGCGSGYGW)

Incubation: 30 min at 37 °C + 30 min with DTT at 37 °C

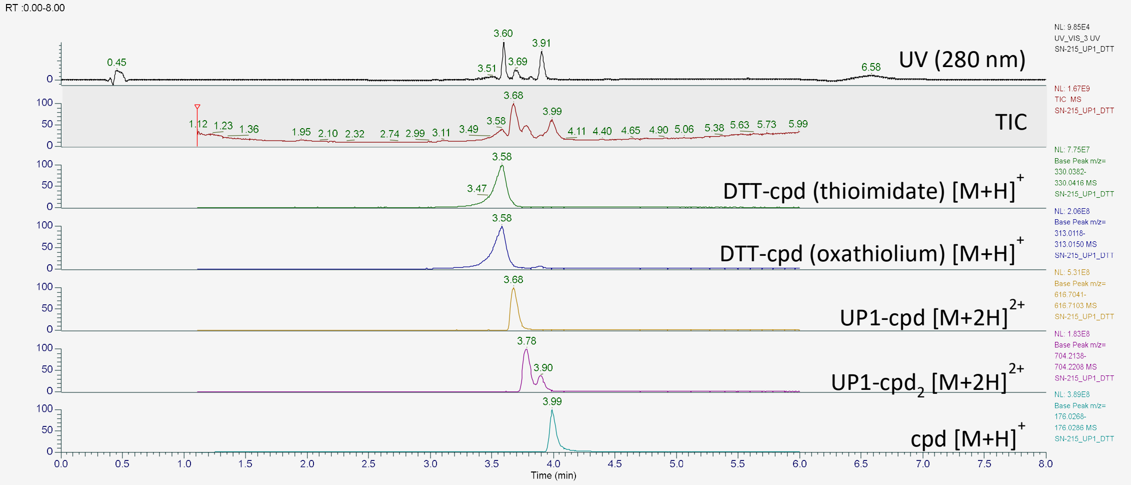

83 + UP2 (AGKGCGSGYGW)

Incubation: 60 min at 37 °C

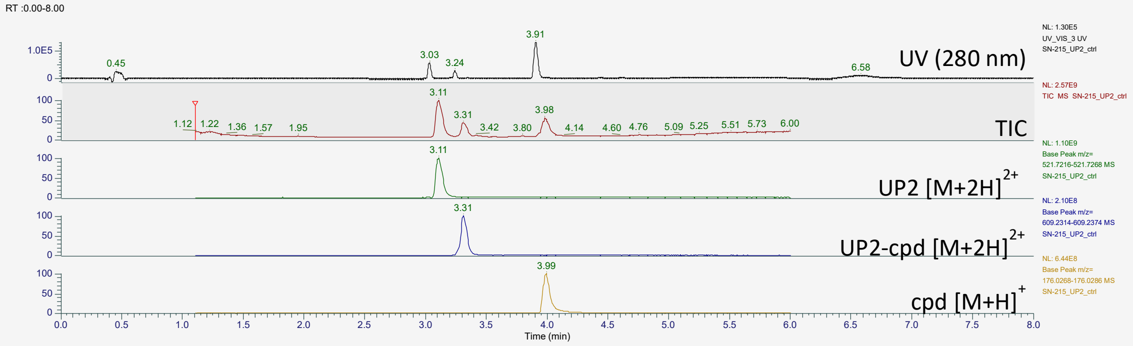

83 + UP2 (AGKGCGSGYGW)

Incubation: 30 min at 37 °C + 30 min with DTT at 37 °C

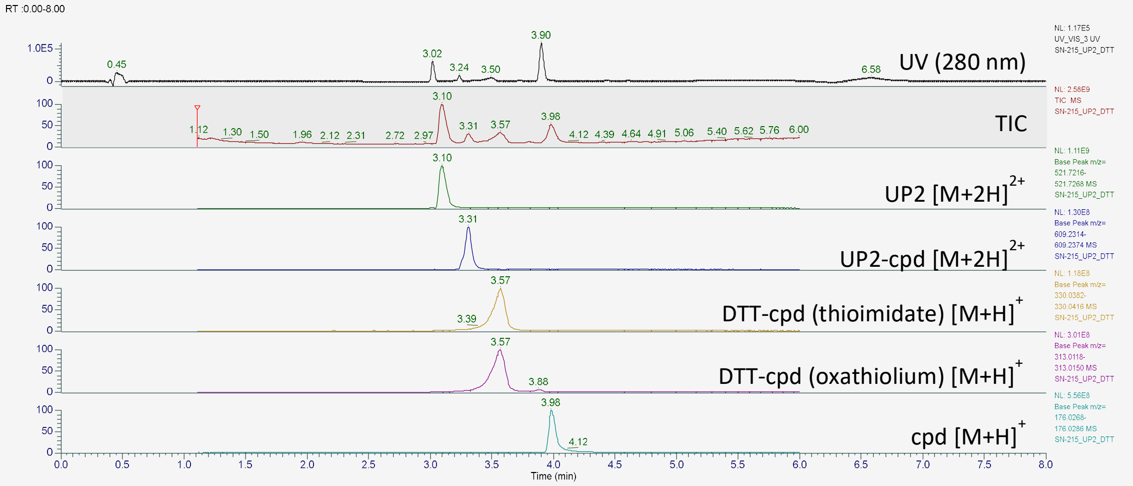

83 (1 eq.) + UP1 (CGKGCGSGYGW):

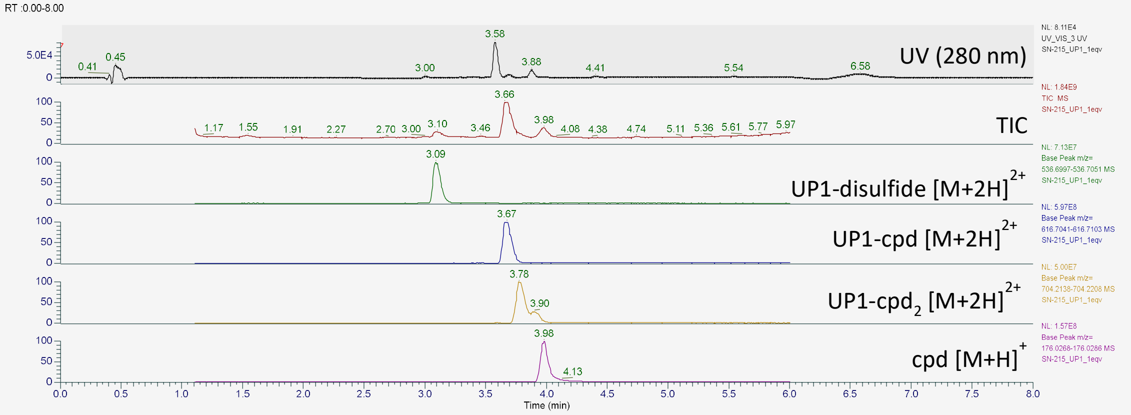

83 (1 eq.) + UP2 (AGKGCGSGYGW):

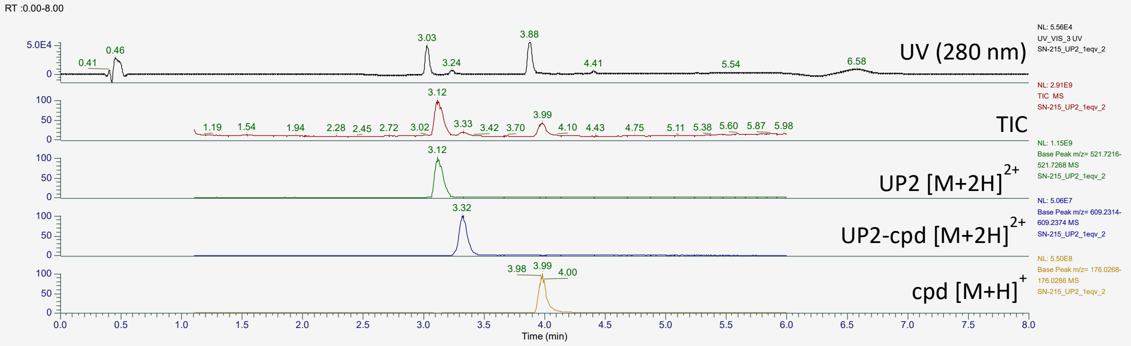

91 + UP1 (CGKGCGSGYGW):

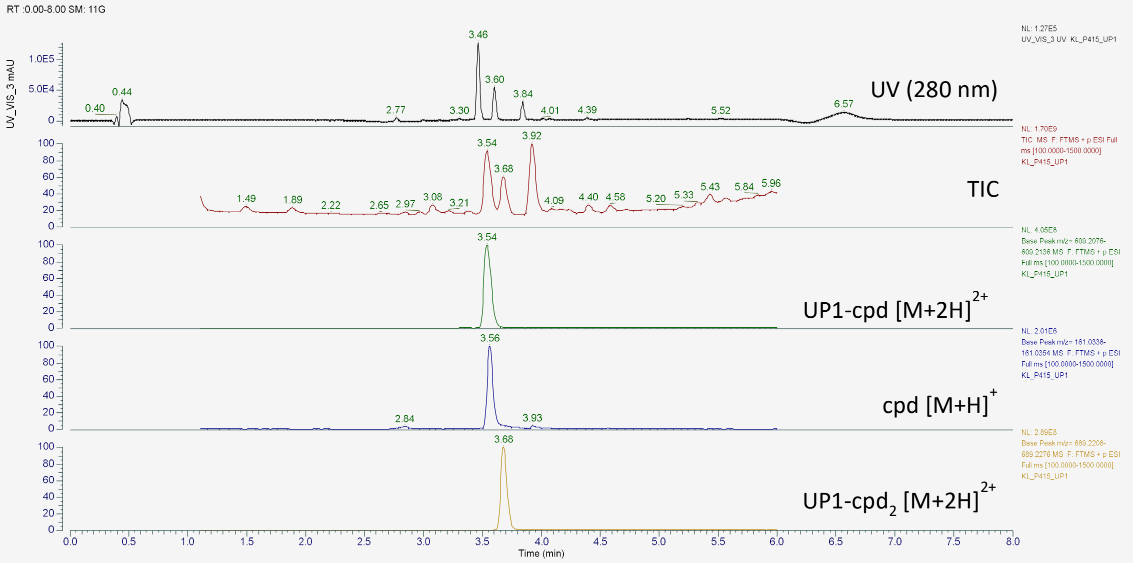

91 + UP2 (AGKGCGSGYGW):

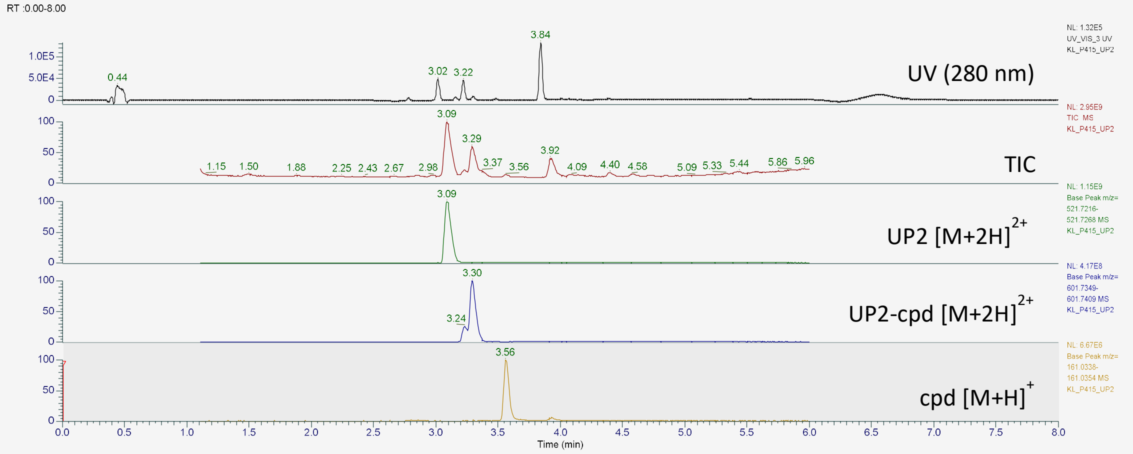

108 + UP1 (CGKGCGSGYGW):

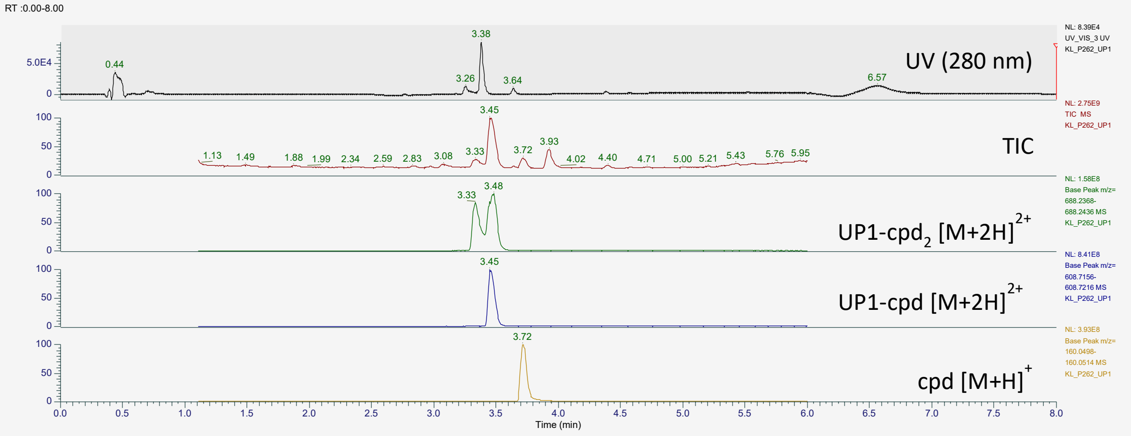

108 + UP2 (AGKGCGSGYGW):

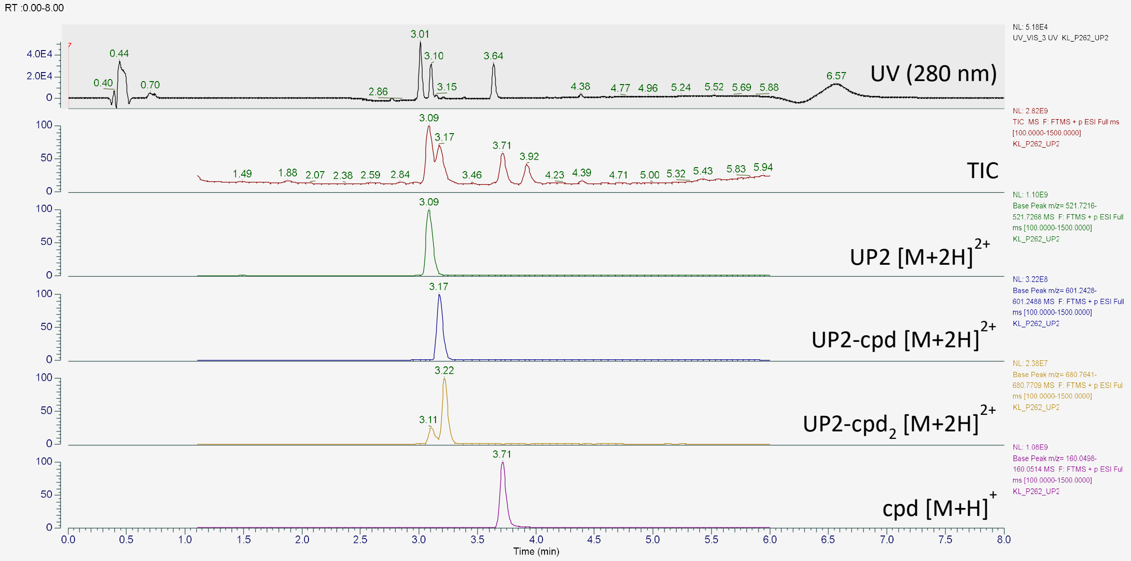

116 + UP1 (CGKGCGSGYGW):

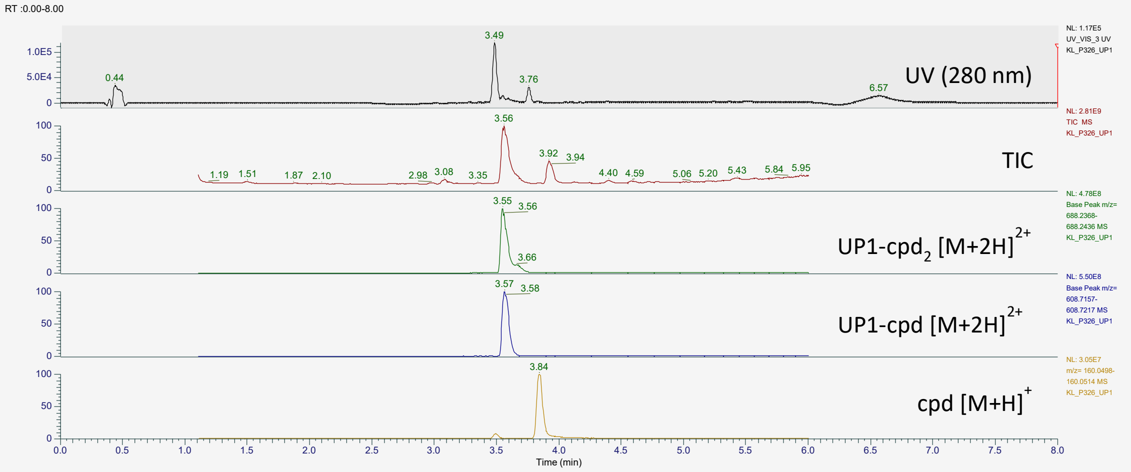

116 + UP2 (AGKGCGSGYGW):

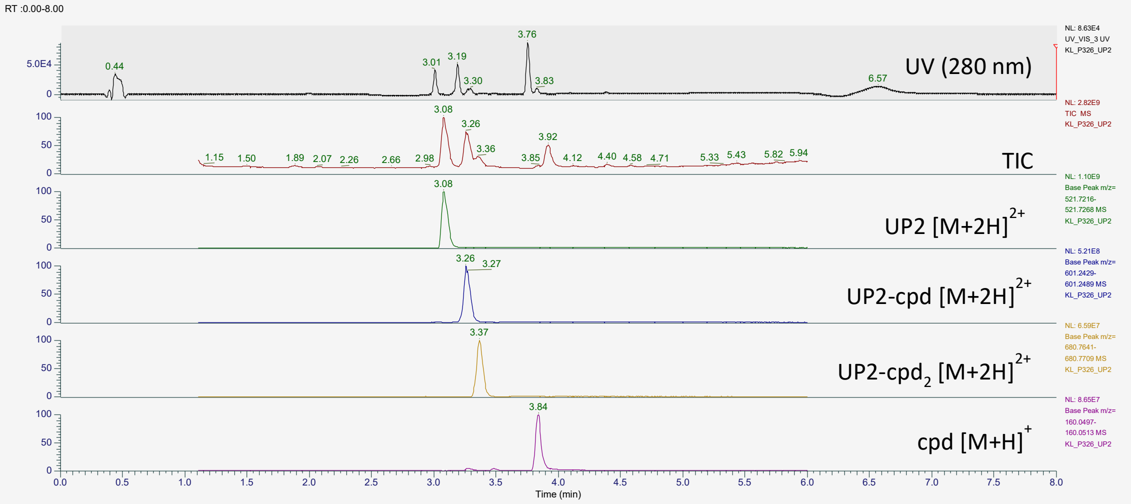

5 Chemistry

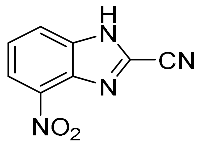

4-Nitro-1*H*-benzo[*d*]imidazole-2-carbonitrile (**2**) was prepared following *GP1* from 3-nitrobenzene-1,2-diamine (5.0 g, 32.6 mmol), and isolated by column chromatography on silica (EtOAc/*n*-hex = 1:3). Yield: 614 mg (10%) of pale-orange solid. ^{1}H NMR (400 MHz, DMSO-d_6 + TFA-*d*) δ 7.54 (t, *J* = 8.1 Hz, 1H), 8.22 (dd, *J* = 8.2, 0.9 Hz, 1H), 8.32 (dd, *J* = 8.1, 0.9 Hz, 1H). ^{13}C NMR (101 MHz, DMSO-d_6 + $CDCl_3$) δ 112.86, 113.17, 124.37, 132.80, 135.30, 147.58, 148.92, 159.29. ESI-HRMS ($[M+H]^+$, m/z): Calcd for $C_8H_5O_2N_4$: 189.04070, found 189.04061. IR (cm^{-1}): 2262 (CN group). Purity: UHPLC (Method I, 254 nm): tr = 4.843 min, 95.5% total area.

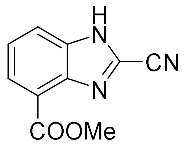

Methyl 2-cyano-1*H*-benzo[*d*]imidazole-4-carboxylate (**3**) was prepared following *GP1* from methyl 2,3-diaminobenzoate (1.03 g, 6.32 mmol), and isolated by column chromatography on silica (1. EtOAc/*n*-hex= 1:4; 2. EtOAc/*n*-hex= 1:2). Yield: 430 mg (34%) of brown solid. ^{1}H NMR (400 MHz, $CDCl_3$) δ 4.06 (s, 3H), 7.49 (dd, *J* = 8.3, 7.6 Hz, 1H), 8.10 (dd, *J* = 8.3, 1.0 Hz, 1H), 8.13 (dd, *J* = 7.6, 1.0 Hz, 1H), 11.43 (s, 1H). ^{13}C NMR (101 MHz, $CDCl_3$) δ 52.90, 111.48, 114.85, 124.23, 125.40, 126.98, 128.73, 133.21, 143.26, 166.33. ESI-HRMS ($[M+H]^+$, m/z): Calcd for $C_{10}H_8O_2N_3$ 202.06110, found 202.06066. IR (cm^{-1}): 2244 (CN group). Purity: UHPLC (Method I, 254 nm): tr = 4.173 min, 96.3% total area.

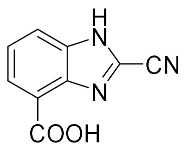

2-Cyano-1*H*-benzo[*d*]imidazole-4-carboxylic acid (**4**) was prepared following *GP5* from methyl 2-cyano-1*H*-benzo[*d*]imidazole-4-carboxylate (200 mg, 0.99 mmol), and isolated by column chromatography on silica (DCM/MeOH= 9:1) and subsequent RP-CC (*GP7*). Yield: 22 mg (12%) of beige solid. ^{1}H NMR (400 MHz, DMSO-d_6 + TFA-*d*) δ 7.43 – 7.49 (m, 1H),

8.04 (td, J = 7.7, 1.1 Hz, 2H). ^{13}C NMR (101 MHz, DMSO-d_6 + TFA-d) δ 112.24, 116.56, 123.68, 125.58, 126.35, 128.29, 132.74, 143.25, 166.04. ESI-HRMS ([M+H]$^+$, m/z): Calcd for $C_9H_6O_2N_3$ 188.04545, found 188.04525. IR (cm^{-1}): 2246 (CN group). Purity: UHPLC (Method I, 254 nm): tr = 3.560 min, 96.3% total area.

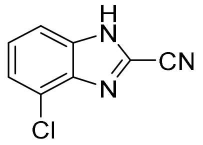

4-Chloro-1*H*-benzo[*d*]imidazole-2-carbonitrile (**5**) was prepared following *GP1* from 3-chlorobenzene-1,2-diamine (2.0 g, 14,0 mmol), and isolated by column chromatography on silica (EtOAc/*n*-hex = 1:3) and subsequent crystallization (EtOH). Yield: 573 mg (23%) of yellow solid. ^{1}H NMR (400 MHz, DMSO-d_6 + TFA-d) δ 7.37–7.44 (m, 1H), 7.49 (dd, J = 7.7, 1.0 Hz, 1H), 7.67 (dd, J = 8.1, 1.0 Hz, 1H). ^{13}C NMR (101 MHz, DMSO-d_6 + TFA-d) δ 112.18, 114.28, 122.12, 124.25, 125.16, 126.38, 137.19, 137.33. ESI-HRMS ([M+H]$^+$, m/z): Calcd for $C_8H_5N_3Cl$ 178.01665, found 178.01647. IR (cm^{-1}): 2246 (CN group). Purity: UHPLC (Method I, 254 nm): tr = 4.123 min, 96.5% total area.

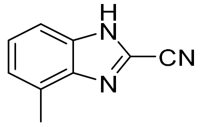

4-Methyl-1*H*-benzo[*d*]imidazole-2-carbonitrile (**6**) was prepared following *GP1* from 3-methylbenzene-1,2-diamine (6.0 g, 49.1 mmol), and isolated by column chromatography on silica (EtOAc/*n*-hex = 1:3). Yield: 1.16 g (15%) of yellow crystals. ^{1}H NMR (400 MHz, DMSO-d_6 + TFA-d) δ 2.52 (s, 3H), 7.17 (dt, J = 7.2, 1.0 Hz, 1H), 7.27 (dd, J = 8.2, 7.2 Hz, 1H), 7.50 (dt, J = 8.2, 0.9 Hz, 1H). ^{13}C NMR (101 MHz, DMSO-d_6 + TFA-d) δ 16.58, 112.78, 113.76, 123.67, 124.87, 125.18, 126.83, 137.50, 138.07. ESI-HRMS ([M+H]$^+$, m/z): Calcd for $C_9H_8N_3$ 158.07127, found 158.07148. IR (cm^{-1}): 2245 (CN group). Purity: UHPLC (Method I, 254 nm): tr = 4.070 min, 95.4% total area.

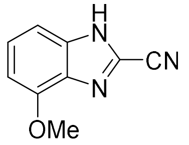

4-Methoxy-1*H*-benzo[*d*]imidazole-2-carbonitrile (**7**) was prepared following *GP1* from 3-methoxybenzene-1,2-diamine (1.0 g, 7.96 mmol), and isolated by column chromatography on silica (EtOAc/*n*-hex= 1:3) and subsequent crystallization (EtOH). Yield: 406 mg (29%) of pale-brown solid. ^{1}H NMR (400 MHz, DMSO-d_6 + TFA-*d*) δ 3.94 (s, 3H), 6.87 (dd, *J* = 7.8, 0.9 Hz, 1H), 7.24 (dd, *J* = 8.3, 0.8 Hz, 1H), 7.27 – 7.35 (m, 1H). ^{13}C NMR (101 MHz, DMSO-d_6 + TFA-*d*) δ 55.85, 104.91, 108.27, 112.67, 123.23, 126.23, 129.66, 138.90, 149.72. ESI-HRMS ($[M+H]^+$, m/z): Calcd for $C_9H_8ON_3$ 174.06619, found 174.06623. IR (cm^{-1}): 2243 (CN group). Purity: UHPLC (Method I, 254 nm): tr = 3.773 min, 96.5% total area.

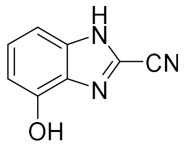

4-Hydroxy-1*H*-benzo[*d*]imidazole-2-carbonitrile (**8**) was prepared following *GP1* from 3-hydroxybenzene-1,2-diamine (3.55 g, 28.6 mmol), and isolated by column chromatography on silica (1. EtOAc/*n*-hex= 1:1; 2. EtOAc/*n*-hex= 3:1) and subsequent RP-CC (*GP7*). Yield: 88 mg (2%) of beige solid. ^{1}H NMR (400 MHz, DMSO-d_6) δ 6.82 (dd, *J* = 7.7, 0.9 Hz, 1H), 7.18 (dd, *J* = 8.3, 0.9 Hz, 1H), 7.26 (dd, *J* = 8.3, 7.7 Hz, 1H), 9.97 (br s, 2H). ^{13}C NMR (101 MHz, Acetone-d_6) δ 106.80, 109.30, 112.86, 124.01, 127.51, 131.22, 139.14, 148.84. ESI-HRMS ($[M+H]^+$, m/z): Calcd for $C_8H_6ON_3$ 160.05054, found 160.05071. IR (cm^{-1}): 2258 (CN group). Purity: UHPLC (Method I, 254 nm): tr = 1.793 min, 96.8% total area.

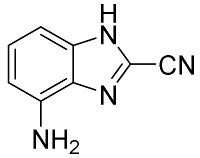

4-Amino-1*H*-benzo[*d*]imidazole-2-carbonitrile (**9**) was prepared following *GP4* from 4-nitro-1*H*-benzo[*d*]imidazole-2-carbonitrile (586 mg, 3.11 mmol), and isolated by column chromatography on silica (DCM/MeOH = 15:1) and subsequent RP-CC (*GP7*). Yield: 79 mg (16%) of dark brown solid. ^{1}H NMR (400 MHz, DMSO-d_6 + TFA-*d*) δ 7.17 – 7.26 (m, 1H), 7.41 – 7.49 (m, 1H), 7.56 (dd, *J* = 8.1, 2.1 Hz, 1H). ^{13}C NMR (101 MHz, DMSO-d_6 + TFA-*d*) δ 111.07, 112.25, 115.66, 124.51, 126.55, 126.67, 126.77, 135.18. ESI-HRMS ($[M+H]^+$, m/z): Calcd for $C_8H_7N_4$ 159.06652, found 159.06638. IR (cm^{-1}): 2244 (CN group). Purity: UHPLC (Method II, 280 nm): tr = 1.860 min, 95.2% total area.

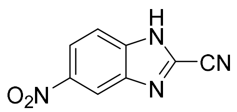

5-Nitro-1*H*-benzo[*d*]imidazole-2-carbonitrile (**10**) was prepared following *GP1* from 4-nitrobenzene-1,2-diamine (5.0 g, 32.7 mmol), and isolated by column chromatography on silica (1. EtOAc/*n*-hex= 1:1; 2. EtOAc/*n*-hex= 2:1) and subsequent crystallization (EtOH). Yield: 1.38 g (22%) of orange crystals. ^{1}H NMR (400 MHz, DMSO-d_6) δ 7.92 (d, *J* = 9.1 Hz, 1H), 8.26 (dd, *J* = 9.1, 2.2 Hz, 1H), 8.67 (d, *J* = 2.2 Hz, 1H). ^{13}C NMR (101 MHz, DMSO-d_6 + $CDCl_3$) δ 112.86, 113.17, 124.37, 132.80, 135.30, 147.58, 148.92, 159.29. ESI-HRMS ($[M–H]^-$, m/z): Calcd for $C_8H_3O_2N_4$ 187.02615, found 187.02538. IR (cm^{-1}): 2254 (CN group). Purity: UHPLC (Method I, 254 nm): tr = 3.947 min, 96.6% total area.

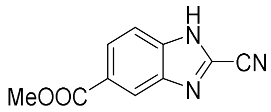

Methyl 2-cyano-1*H*-benzo[*d*]imidazole-5-carboxylate (**11**) was prepared following *GP1* from methyl 3,4-diaminobenzoate (8.3 g, 49.9 mmol), and isolated by column chromatography on silica (1. EtOAc/*n*-hex= 1:1; 2. EtOAc/*n*-hex= 2:1) and subsequent RP-CC (*GP7*). Yield: 156 mg (2%) of off-white solid. ^{1}H NMR (400 MHz, $CDCl_3$) δ 3.89 (s, 3H), 7.81 (d, *J* = 8.7 Hz, 1H), 7.99 (dd, *J* = 8.7, 1.6 Hz, 1H), 8.31 (s, 1H), 14.53 (br s, 1H). ^{13}C NMR (101 MHz, DMSO-d_6 + TFA-*d*) δ 52.42, 112.29, 116.19, 119.51, 125.78, 126.37, 126.84, 138.62, 140.18, 166.42. ESI-HRMS ($[M+H]^+$, m/z): Calcd for $C_{10}H_8O_2N_3$ 202.06110, found 202.06088. IR (cm^{-1}): 2259 (CN group). Purity: UHPLC (Method I, 254 nm): tr = 4.043 min, 99.7% total area.

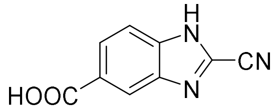

2-Cyano-1*H*-benzo[*d*]imidazole-5-carboxylic acid (**12**) was prepared following *GP5* from methyl 2-cyano-1*H*-benzo[*d*]imidazole-5-carboxylate (200 mg, 0.99 mmol), and isolated by column chromatography on silica (DCM/MeOH= 9:1). Yield: 67 mg (36%) of yellow solid. ^{1}H NMR (400 MHz, DMSO-d_6 + TFA-*d*) δ 7.79 (dd, *J* = 8.7, 0.8 Hz, 1H), 7.99 (dd, *J* = 8.7, 1.6 Hz, 1H), 8.31 (dd, *J* = 1.4, 0.7 Hz, 1H). ^{13}C NMR (101 MHz, DMSO-d_6 + TFA-*d*) δ 112.24, 116.02, 119.37, 125.93, 126.47, 127.38, 138.41, 140.00, 167.25. ESI-HRMS ($[M+H]^+$, m/z):

Calcd for $C_9H_6O_2N_3$ 188.04545, found 188.04519. IR (cm^{-1}): 2254 (CN group). Purity: UHPLC (Method I, 280 nm): tr = 2.907 min, 96.6% total area.

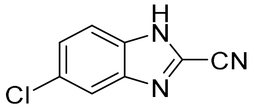

5-Chloro-1*H*-benzo[*d*]imidazole-2-carbonitrile (**13**) was prepared following *GP1* from 4-chlorobenzene-1,2-diamine (5.0 g, 35.1 mmol), and isolated by column chromatography on silica (EtOAc/*n*-hex = 1:3) and subsequent RP-CC (*GP7*). Yield: 573 mg (9%) of yellow crystals. ^{1}H NMR (400 MHz, DMSO-d_6 + TFA-*d*) δ 7.42 (dd, *J* = 8.8, 2.0 Hz, 1H), 7.74 (d, *J* = 8.8 Hz, 1H), 7.82 (d, *J* = 1.9 Hz, 1H). ^{13}C NMR (101 MHz, DMSO-d_6 + TFA-*d*) δ 112.27, 116.29, 118.16, 125.53, 125.58, 129.71, 136.69, 138.63. ESI-HRMS ([M+H]$^+$, m/z): Calcd for $C_8H_5N_3Cl$ 178.01665, found 178.01647. IR (cm^{-1}): 2242 (CN group). Purity: UHPLC (Method I, 254 nm): tr = 4.257 min, 97.1% total area.

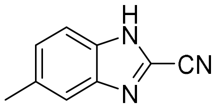

5-Methyl-1*H*-benzo[*d*]imidazole-2-carbonitrile (**14**) was prepared following *GP1* from 4-methylbenzene-1,2-diamine (6.0 g, 49.1 mmol), and isolated by column chromatography on silica (EtOAc/*n*-hex = 1:3) and subsequent crystallization (EtOH). Yield: 849 mg (11%) of orange crystals. ^{1}H NMR (400 MHz, DMSO-d_6 + TFA-*d*) δ 2.43 (s, 3H), 7.22 (dd, *J* = 8.4, 1.6 Hz, 1H), 7.48 (dt, *J* = 1.7, 0.9 Hz, 1H), 7.60 (dd, *J* = 8.4, 0.7 Hz, 1H). ^{13}C NMR (101 MHz, DMSO-d_6 + TFA-*d*) δ 21.33, 112.77, 115.22, 116.91, 123.67, 126.78, 135.10, 136.98, 137.46. ESI-HRMS ([M+H]$^+$, m/z): Calcd for $C_9H_8N_3$ 158.07127, found 158.07149. IR (cm^{-1}): 2236 (CN group). Purity: UHPLC (Method I, 254 nm): tr = 4.083 min, 99.5% total area.

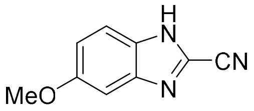

5-Methoxy-1*H*-benzo[*d*]imidazole-2-carbonitrile (**15**) was prepared following *GP1* from 4-methoxybenzene-1,2-diamine (7.0 g, 50.7 mmol), and isolated by column chromatography on silica (1. EtOAc/*n*-hex= 1:3; 2. EtOAc/*n*-hex= 1:1) and subsequent crystallization (EtOH). Yield: 443 mg (5%) of brown solid. ^{1}H NMR (400 MHz, DMSO-d_6 + TFA-*d*) δ 3.80 (s, 3H), 7.00 (dd, *J* = 9.0, 2.4 Hz, 1H), 7.11 (d, *J* = 2.4 Hz, 1H), 7.61 (d, *J* = 9.0 Hz, 1H). ^{13}C NMR

(101 MHz, DMSO-d_6 + TFA-d) δ 55.74, 96.39, 112.90, 116.08, 119.45, 123.20, 134.38, 137.31, 158.40. ESI-HRMS ($[M+H]^+$, m/z): Calcd for $C_9H_8ON_3$ 174.06619, found 174.06608. IR (cm^{-1}): 2229 (CN group). Purity: UHPLC (Method I, 254 nm): tr = 3.860 min, 97.3% total area.

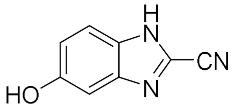

5-Hydroxy-1*H*-benzo[*d*]imidazole-2-carbonitrile (**16**) was prepared following *GP6* from 5-methoxy-1*H*-benzo[*d*]imidazole-2-carbonitrile (100 mg, 0.58 mmol), and isolated by column chromatography on silica (EtOAc/*n*-hex = 1:1). Yield: 18 mg (20%) of pale-yellow crystalls. ^{1}H NMR (400 MHz, DMSO-d_6 + TFA-d) δ 6.90 (dd, J = 9.2, 2.2 Hz, 1H), 6.91 (s, 1H), 7.51 – 7.57 (m, 1H). ^{13}C NMR (101 MHz, DMSO-d_6 + TFA-d) δ 97.87, 112.95, 115.71, 119.29, 122.51, 134.29, 136.78, 156.35. ESI-HRMS ($[M+H]^+$, m/z): Calcd for $C_8H_6ON_3$ 160.05054, found 160.05054. IR (cm^{-1}): 2234 (CN group). Purity: UHPLC (Method II, 254 nm): tr = 3.740 min, 95.6% total area.

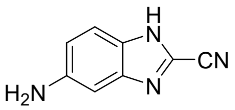

5-Amino-1*H*-benzo[*d*]imidazole-2-carbonitrile (**17**) was prepared following *GP4* from 5-nitro-1*H*-benzo[*d*]imidazole-2-carbonitrile (350 mg, 1.86 mmol), and isolated by column chromatography on silica (DCM/MeOH= 20:1). Yield: 43 mg (15%) of brown solid. ^{1}H NMR (400 MHz, DMSO-d_6 + TFA-d) δ 7.40 (dd, J = 8.7, 2.1 Hz, 1H), 7.77 (d, J = 1.9 Hz, 1H), 7.87 (d, J = 8.7 Hz, 1H). ^{13}C NMR (101 MHz, DMSO-d_6 + TFA-d) δ 111.58, 112.21, 117.89, 120.53, 125.99, 128.55, 137.02, 138.13. ESI-HRMS ($[M+H]^+$, m/z): Calcd for $C_8H_7N_4$ 159.06652, found 159.06664. IR (cm^{-1}): 2228 (CN group). Purity: UHPLC (Method II, 280 nm): tr = 1.177 min, 96.6% total area.

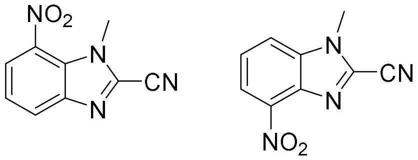

1-Methyl-4-nitro-1*H*-benzo[*d*]imidazole-2-carbonitrile (**19**) and 1-methyl-7-nitro-1*H*-benzo[*d*]imidazole-2-carbonitrile (**43**) were prepared following *GP3* from 4-nitro-1*H*-

benzo[*d*]imidazole-2-carbonitrile (1.45 g, 7.71 mmol), and isolated by column chromatography on silica (1. EtOAc/*n*-hex= 1:3; 2. EtOAc/*n*-hex= 1:1; 2. EtOAc/*n*-hex= 1:4) and subsequent RP-CC (*GP7*). Overall yield: 788 mg (51%).

1-Methyl-4-nitro-1*H*-benzo[*d*]imidazole-2-carbonitrile (**19**). **19** was additionally purified by crystallization (EtOH). Yield: 740 mg (47%) of yellow solid. ^{1}H NMR (400 MHz, $CDCl_3$) δ 4.13 (s, 3H), 7.65 (t, *J* = 8.1 Hz, 1H), 7.82 (dd, *J* = 8.3, 1.0 Hz, 1H), 8.30 (dd, *J* = 7.9, 1.0 Hz, 1H). ^{13}C NMR (101 MHz, $CDCl_3$) δ 32.19, 110.12, 117.12, 121.79, 125.79, 130.41, 135.68, 137.05, 140.11. ESI-HRMS ($[M+H]^+$, m/z): Calcd for $C_9H_7O_2N_4$ 203.05635, found 203.05628. IR (cm^{-1}): 2245 (CN group). Purity: UHPLC (Method I, 254 nm): tr = 3.627 min, 96.0% total area.

1-Methyl-7-nitro-1*H*-benzo[*d*]imidazole-2-carbonitrile (**43**). **43** was additionally purified by column chromatography on silica (1. DCM/*n*-hex= 1:1; 2. DCM/*n*-hex= 9:1). Yield: 48 mg (3%) of brown solid. ^{1}H NMR (400 MHz, $CDCl_3$) δ 4.20 (s, 3H), 7.50 (t, *J* = 8.1 Hz, 1H), 8.15 (dd, *J* = 8.2, 1.0 Hz, 1H), 8.18 (dd, *J* = 8.0, 1.0 Hz, 1H). ^{13}C NMR (101 MHz, $CDCl_3$) δ 36.33, 110.32, 123.63, 124.07, 127.02, 128.57, 131.24, 136.61, 145.85. ESI-HRMS ($[M+H]^+$, m/z): Calcd for $C_9H_7O_2N_4$ 203.05635, found 203.05637. IR (cm^{-1}): 2241 (CN group). Purity: UHPLC (Method I, 254 nm): tr = 4.430 min, 99.8% total area.

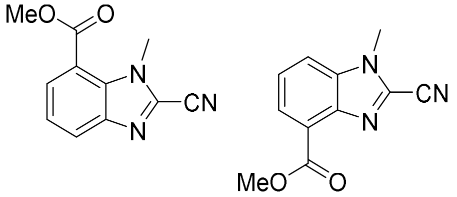

Methyl 2-cyano-1-methyl-1*H*-benzo[*d*]imidazole-4-carboxylate (**20**) and methyl 2-cyano-1-methyl-1*H*-benzo[*d*]imidazole-7-carboxylate (**44**) were prepared following *GP3* from methyl 2-cyano-1*H*-benzo[*d*]imidazole-4-carboxylate (438 mg, 2.18 mmol), and isolated by column chromatography on silica (EtOAc/*n*-hex= 1:1). Overall yield: 356 mg (76%).

Methyl 2-cyano-1-methyl-1*H*-benzo[*d*]imidazole-4-carboxylate (**20**). Yield: 185 mg (39%) of pale-yellow solid. ^{1}H NMR (400 MHz, Acetone-d_6) δ 3.95 (s, 3H), 4.13 (s, 3H), 7.58 (dd, *J* = 8.3, 7.5 Hz, 1H), 7.93 (dd, *J* = 8.3, 1.1 Hz, 1H), 7.98 (dd, *J* = 7.5, 1.1 Hz, 1H). ^{13}C NMR (101 MHz, Acetone-d_6) δ 32.07, 52.33, 111.94, 116.67, 123.88, 126.06, 127.51, 129.40, 136.88, 141.31, 166.32. ESI-HRMS ($[M+H]^+$, m/z): Calcd for $C_{11}H_{10}O_2N_3$ 216.07675, found

216.07632. IR (cm^{-1}): 2239 (CN group). Purity: UHPLC (Method I, 254 nm): tr = 4.003 min, 98.5% total area.

Methyl 2-cyano-1-methyl-1*H*-benzo[*d*]imidazole-7-carboxylate (**44**). Yield: 171 mg (36%) of white solid. ^{1}H NMR (400 MHz, Acetone-*d$_6$*) δ 3.98 (s, 3H), 4.17 (s, 3H), 7.41 – 7.46 (m, 1H), 7.93 – 7.94 (m, 1H), 7.95 – 7.97 (m, 1H). ^{13}C NMR (101 MHz, Acetone-*d$_6$*) δ 36.10, 52.88, 111.97, 118.68, 124.09, 126.52, 129.80, 130.90, 133.37, 145.04, 166.15. ESI-HRMS ([M+H]$^+$, m/z): Calcd for $C_{11}H_{10}O_2N_3$ 216.07675, found 216.07637. IR (cm^{-1}): 2240 (CN group). Purity: UHPLC (Method I, 280 nm): tr = 4.443 min, 95.7% total area.

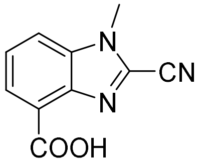

2-Cyano-1-methyl-1*H*-benzo[*d*]imidazole-4-carboxylic acid (**21**) was prepared following *GP5* from methyl 2-cyano-1-methyl-1*H*-benzo[*d*]imidazole-4-carboxylate (100 mg, 0.47 mmol), and isolated by column chromatography on silica (DCM/MeOH= 9:1) and subsequent crystallization (EtOH). Yield: 58 mg (61%) of pale-brown solid. ^{1}H NMR (400 MHz, DMSO-*d$_6$*) δ 4.03 (s, 3H), 7.60 (dd, *J* = 8.3, 7.4 Hz, 1H), 7.95 (dd, *J* = 7.5, 1.1 Hz, 1H), 8.03 (dd, *J* = 8.3, 1.1 Hz, 1H), 13.02 (br s, 1H). ^{13}C NMR (101 MHz, DMSO-*d$_6$*) δ 31.71, 111.43, 116.28, 123.26, 125.33, 126.61, 128.11, 135.73, 166.20. ESI-HRMS ([M+H]$^+$, m/z): Calcd for $C_{10}H_8O_2N_3$ 202.06110, found 202.06096. IR (cm^{-1}): 2241 (CN group). Purity: UHPLC (Method I, 280 nm): tr = 3.070 min, 95.0% total area.

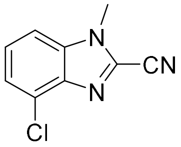

4-Chloro-1-methyl-1*H*-benzo[*d*]imidazole-2-carbonitrile (**22**) was prepared following *GP3* from 4-chloro-1*H*-benzo[*d*]imidazole-2-carbonitrile (2.78 g, 15.7 mmol), and isolated by column chromatography on silica (EtOAc/*n*-hex = 1:3) and subsequent RP-CC (*GP7*). Yield: 90 mg (3%) of yellow crystals. ^{1}H NMR (400 MHz, DMSO-*d$_6$*) δ 4.01 (s, 3H), 7.47–7.54 (m, 2H), 7.77 (dd, *J* = 6.5, 2.7 Hz, 1H). ^{13}C NMR (101 MHz, DMSO-*d$_6$*) δ 31.97, 111.12, 111.20, 123.77, 124.27, 126.71, 127.67, 135.92, 138.79. ESI-HRMS ([M+H]$^+$, m/z): Calcd for

$C_9H_7N_3Cl$ 192.03230, found 192.03207. IR (cm^{-1}): 2293 (CN group). Purity: UHPLC (Method I, 254 nm): tr = 4.397 min, 95.1% total area.

Positional isomer 7-chloro-1-methyl-1*H*-benzo[*d*]imidazole-2-carbonitrile (**46**) was not obtained in sufficient purity, thus alternative synthetic route was used.

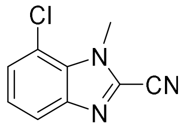

7-Chloro-1-methyl-1*H*-benzo[*d*]imidazole-2-carbonitrile (**46**)

2-Chloro-6-nitroaniline (5.0 g, 29.0 mmol) was dissolved in anhydrous DMF (40 mL), flushed with Ar, cooled on an icebath, then NaH (60% dispersion on mineral oil, 1.16 g, 29.0 mmol) was added, the reaction mixture stirred for 15 min followed by the addition of MeI (1.98 mL, 31.9 mmol). The reaction mixture was stirred under Ar at rt for 18 h, volatile components evaporated *in vacuo* and the residue dissolved in EtOAc (50 mL), washed with H_2O (50 mL), saturated brine (50 mL), dried, filtered, solvents evaporated *in vacuo* and 2-chloro-*N*-methyl-6-nitroaniline (**122**) isolated by column chromatography on silica (PE/Et_2O = 9:1). Yield: 3.93 g (73%) of orange oil. ^1H NMR (400 MHz, $CDCl_3$) δ 3.08 (d, J = 5.5 Hz, 3H), 6.68 (dd, J = 8.5, 7.8 Hz, 1H), 6.71 (br s, 1H), 7.46 (dd, J = 7.8, 1.6 Hz, 1H), 7.88 (dd, J = 8.5, 1.6 Hz, 1H). ^{13}C NMR (101 MHz, $CDCl_3$) δ 34.16, 117.04, 123.58, 125.50, 136.20, 137.57, 143.46. ESI-HRMS ($[M+H]^+$, m/z): Calcd for $C_7H_8O_2N_2Cl$ 187.02688, found 187.02659.

2-Chloro-*N*-methyl-6-nitroaniline (**122**) (3.0 g, 16.1 mmol) was dissolved in MeOH (30 mL), flushed with Ar, then 10% Pd/C (100 mg) was added, and $NaBH_4$ was added in 100 mg portions with stirring on an icebath until the yellow solution turned greyish. The catalyst was removed by filtration, washed with MeOH, and the filtrate evaporated *in vacuo*. Oily residue was acidified with 6 M $HCl_{(aq)}$ (50 mL), then made alkaline (pH = 13–14) using 50% $NaOH_{(aq)}$, extracted with EtOAc (2 × 30 mL), organic layer washed with saturated brine (30 mL), dried, filtered and volatile components evaporated *in vacuo* to afford crude 6-chloro-N^1-methylbenzene-1,2-diamine (**123**). Yield: 1.39 g (55%) of dark-brown oil. ESI-HRMS ($[M+H]^+$, m/z): Calcd for $C_7H_{10}N_2Cl$ 157.05270, found 157.05259.

7-Chloro-1-methyl-1*H*-benzo[*d*]imidazole-2-carbonitrile (**46**) was prepared following *GP1* from 6-chloro-N^1-methylbenzene-1,2-diamine (**123**) (1.39 g, 8.91 mmol), and isolated by

column chromatography on silica (EtOAc/*n*-hex = 1:3). Yield: 376 mg (22%) of beige solid. ^{1}H NMR (400 MHz, $CDCl_3$) δ 4.28 (s, 3H), 7.27 (dd, *J* = 8.2, 7.7 Hz, 1H), 7.38 (dd, *J* = 7.7, 1.0 Hz, 1H), 7.70 (dd, *J* = 8.3, 1.0 Hz, 1H). ^{13}C NMR (101 MHz, $CDCl_3$) δ 34.33, 110.65, 117.24, 120.61, 125.01, 127.43, 128.78, 130.97, 144.48. ESI-HRMS ($[M+H]^+$, m/z): Calcd for $C_9H_7N_3Cl$ 192.03230, found 192.03193. IR (cm^{-1}): 2243 (CN group). Purity: UHPLC (Method I, 254 nm): tr = 4.583 min, 99.0% total area.

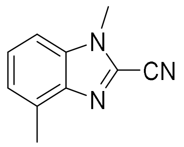

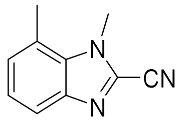

1,4-Dimethyl-1*H*-benzo[*d*]imidazole-2-carbonitrile (**23**) and 1,7-dimethyl-1*H*-benzo[*d*]imidazole-2-carbonitrile (**47**) were prepared following *GP3* from 4-methyl-1*H*-benzo[*d*]imidazole-2-carbonitrile (1.19 g, 7.57 mmol), and isolated by column chromatography on silica (EtOAc/*n*-hex = 1:3) and subsequent RP-CC (*GP7*). Overall yield: 441 mg (34%).

1,4-Dimethyl-1*H*-benzo[*d*]imidazole-2-carbonitrile (**23**). Yield: 194 mg (15%) of yellow crystals. ^{1}H NMR (400 MHz, $CDCl_3$) δ 2.65 (s, 3H), 3.95 (s, 3H), 7.18 (dt, *J* = 7.2, 1.0 Hz, 1H), 7.23 (dt, *J* = 8.3, 0.9 Hz, 1H), 7.36 (dd, *J* = 8.3, 7.2 Hz, 1H). ^{13}C NMR (101 MHz, $CDCl_3$) δ 16.55, 31.41, 107.74, 111.31, 124.53, 126.15, 126.56, 132.00, 134.61, 142.22. ESI-HRMS ($[M+H]^+$, m/z): Calcd for $C_{10}H_{10}N_3$ 172.08692, found 172.08700. IR (cm^{-1}): 2237 (CN group). Purity: UHPLC (Method I, 254 nm): tr = 4.430 min, 97.5% total area.

1,7-Dimethyl-1*H*-benzo[*d*]imidazole-2-carbonitrile (**47**). Yield: 246 mg (19%) of yellow crystals. ^{1}H NMR (400 MHz, $CDCl_3$) δ 2.75 (s, 3H), 4.21 (s, 3H), 7.16 (dt, *J* = 7.2, 1.0 Hz, 1H), 7.22–7.28 (m, 1H), 7.65 (dt, *J* = 8.2, 1.0 Hz, 1H). ^{13}C NMR (101 MHz, $CDCl_3$) δ 18.60, 34.22, 111.33, 119.74, 122.36, 124.61, 127.81, 128.46, 133.72, 143.37. ESI-HRMS ($[M+H]^+$, m/z): Calcd for $C_{10}H_{10}N_3$ 172.08692, found 172.08700. IR (cm^{-1}): 2235 (CN group). Purity: UHPLC (Method I, 254 nm): tr = 4.373 min, 95.1% total area.

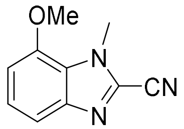

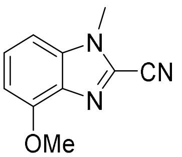

4-Methoxy-1-methyl-1*H*-benzo[*d*]imidazole-2-carbonitrile (**24**) and 7-methoxy-1-methyl-1*H*-benzo[*d*]imidazole-2-carbonitrile (**48**) were prepared following *GP3* from methyl 4-methoxy-1*H*-benzo[*d*]imidazole-2-carbonitrile (300 mg, 1.73 mmol), and isolated by column chromatography on silica (EtOAc/*n*-hex= 1:3). Overall yield: 199 mg (61%).

4-Methoxy-1-methyl-1*H*-benzo[*d*]imidazole-2-carbonitrile (**24**). Yield: 96 mg (30%) of yellow crystals. ^{1}H NMR (400 MHz, $CDCl_3$) δ 3.97 (s, 3H), 4.03 (s, 3H), 6.76 (dd, *J* = 8.0, 0.7 Hz, 1H), 7.00 (dd, *J* = 8.3, 0.7 Hz, 1H), 7.40 (t, *J* = 8.1 Hz, 1H). ^{13}C NMR (101 MHz, $CDCl_3$) δ 31.54, 56.05, 102.69, 104.08, 111.11, 125.65, 127.68, 133.50, 136.33, 152.47. ESI-HRMS ($[M+H]^+$, m/z): Calcd for $C_{10}H_{10}ON_3$ 188.08184, found 188.08185. IR (cm^{-1}): 2234 (CN group). Purity: UHPLC (Method I, 254 nm): tr = 4.150 min, 95.8% total area.

7-Methoxy-1-methyl-1*H*-benzo[*d*]imidazole-2-carbonitrile (**48**). Yield: 103 mg (32%) of yellow solid. ^{1}H NMR (400 MHz, $CDCl_3$) δ 3.94 (s, 3H), 4.13 (s, 3H), 6.76 (dd, *J* = 7.8, 0.8 Hz, 1H), 7.22 (t, *J* = 8.1 Hz, 1H), 7.34 (dd, *J* = 8.4, 0.8 Hz, 1H). ^{13}C NMR (101 MHz, $CDCl_3$) δ 34.19, 55.82, 105.86, 111.07, 113.73, 124.83, 125.06, 127.06, 144.36, 147.58. ESI-HRMS ($[M+H]^+$, m/z): Calcd for $C_{10}H_{10}ON_3$ 188.08184, found 188.08186. IR (cm^{-1}): 2240 (CN group). Purity: UHPLC (Method I, 254 nm): tr = 4.457 min, 95.7% total area.

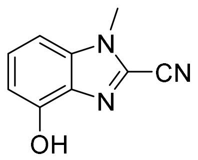

4-Hydroxy-1-methyl-1*H*-benzo[*d*]imidazole-2-carbonitrile (**25**) was prepared following *GP6* from 4-methoxy-1-methyl-1*H*-benzo[*d*]imidazole-2-carbonitrile (50 mg, 0.27 mmol), and isolated by column chromatography on silica (EtOAc/*n*-hex = 1:1). Yield: 16 mg (34%) of white solid. ^{1}H NMR (400 MHz, DMSO-*d*$_6$) δ 3.94 (s, 3H), 6.71 (dd, *J* = 7.8, 0.8 Hz, 1H), 7.11 (dd, *J* = 8.3, 0.8 Hz, 1H), 7.25 – 7.33 (m, 1H), 10.37 (s, 1H). ^{13}C NMR (101 MHz, DMSO-*d*$_6$) δ 31.47, 101.79, 107.86, 111.76, 124.99, 127.28, 132.48, 136.70, 150.07. ESI-HRMS ($[M+H]^+$, m/z): Calcd for $C_9H_8ON_3$ 174.06619, found 174.06613. IR (cm^{-1}): 2242 (CN group). Purity: UHPLC (Method II, 254 nm): tr = 4.090 min, 99.4% total area.

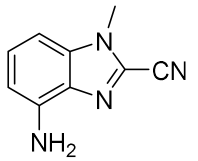

4-Amino-1-methyl-1*H*-benzo[*d*]imidazole-2-carbonitrile (**26**) was prepared following *GP4* from 1-methyl-4-nitro-1*H*-benzo[*d*]imidazole-2-carbonitrile (320 mg, 1.58 mmol), and isolated by column chromatography on silica (EtOAc/*n*-hex= 1:1). Yield: 72 mg (26%) of orange crystals. ^{1}H NMR (400 MHz, $CDCl_3$) δ 3.93 (s, 3H), 4.52 (br s, 2H), 6.58 (d, *J* = 7.8 Hz, 1H), 6.74 (d, *J* = 8.2 Hz, 1H), 7.26 (t, *J* = 8.0 Hz, 1H). ^{13}C NMR (101 MHz, $CDCl_3$) δ 31.51, 99.19, 106.66, 111.48, 124.69, 128.09, 132.50, 135.93, 140.11. ESI-HRMS ($[M+H]^+$, m/z): Calcd for $C_9H_9N_4$ 173.08217, found 173.08206. IR (cm^{-1}): 2231 (CN group). Purity: UHPLC (Method I, 254 nm): tr = 2.007 min, 98.7% total area.

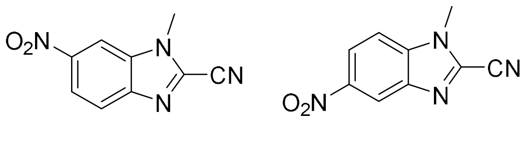

1-Methyl-5-nitro-1*H*-benzo[*d*]imidazole-2-carbonitrile (**27**) and 1-methyl-6-nitro-1*H*-benzo[*d*]imidazole-2-carbonitrile (**35**) were prepared following *GP3* from 5-nitro-1*H*-benzo[*d*]imidazole-2-carbonitrile (460 mg, 2.44 mmol), and isolated by column chromatography on silica (1. EtOAc/*n*-hex= 1:3; 2. EtOAc/*n*-hex= 1:1) and subsequent crystallization (EtOH). Overall yield: 231 mg (47%).

1-Methyl-5-nitro-1*H*-benzo[*d*]imidazole-2-carbonitrile (**27**). **27** was additionally purified by column chromatography on silica (DCM/acetone= 8:1). Yield: 175 mg (35%) of beige solid. ^{1}H NMR (400 MHz, DMSO-d_6) δ 4.07 (s, 3H), 8.03 (dd, *J* = 9.1, 0.6 Hz, 1H), 8.37 (dd, *J* = 9.1, 2.2 Hz, 1H), 8.74 (dd, *J* = 2.2, 0.6 Hz, 1H). ^{13}C NMR (101 MHz, DMSO-d_6) δ 32.12, 110.84, 112.88, 117.19, 120.76, 131.04, 138.59, 140.64, 144.41. ESI-HRMS ($[M+H]^+$, m/z): Calcd for $C_9H_7O_2N_4$ 203.05635, found 203.05616. IR (cm^{-1}): 2240 (CN group). Purity: UHPLC (Method I, 254 nm): tr = 4.213 min, 96.5% total area.

1-Methyl-6-nitro-1*H*-benzo[*d*]imidazole-2-carbonitrile (**35**). Yield: 56 mg (11%) of orange crystals. ^{1}H NMR (400 MHz, DMSO-d_6) δ 4.12 (s, 3H), 8.03 (dd, *J* = 9.1, 0.6 Hz, 1H), 8.24 (dd, *J* = 9.0, 2.3 Hz, 1H), 8.88 (dd, *J* = 2.2, 0.6 Hz, 1H). ^{13}C NMR (101 MHz, DMSO-d_6) δ 32.20, 109.40, 110.85, 118.93, 121.45, 131.69, 134.19, 145.23, 145.39. ESI-HRMS ($[M+H]^+$, m/z): Calcd for $C_9H_7O_2N_4$ 203.05635, found 203.05634. IR (cm^{-1}): 2252 (CN group). Purity: UHPLC (Method I, 254 nm): tr = 4.190 min, 96.3% total area.

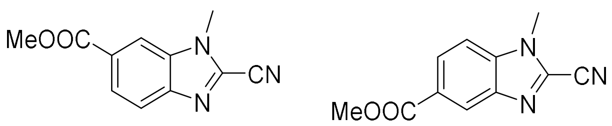

Methyl 2-cyano-1-methyl-1*H*-benzo[*d*]imidazole-5-carboxylate (**28**) and methyl 2-cyano-1-methyl-1*H*-benzo[*d*]imidazole-6-carboxylate (**36**) were prepared following *GP3* from methyl 2-cyano-1*H*-benzo[*d*]imidazole-5-carboxylate (3.38 g, 16.8 mmol), and isolated by column chromatography on silica (1. EtOAc/*n*-hex= 1:3; 1. EtOAc/*n*-hex= 1:1) and subsequent crystallization (EtOH). Overall yield: 1.62 g (45%).

Methyl 2-cyano-1-methyl-1*H*-benzo[*d*]imidazole-5-carboxylate (**28**). Yield: 1.32 g (37%) of brown crystalls. ^{1}H NMR (400 MHz, DMSO-d_6) δ 3.90 (s, 3H), 4.03 (s, 3H), 7.90 (dd, *J* = 8.8, 0.7 Hz, 1H), 8.09 (dd, *J* = 8.7, 1.5 Hz, 1H), 8.38 (dd, *J* = 1.6, 0.7 Hz, 1H). ^{13}C NMR (101 MHz, DMSO-d_6) δ 31.76, 52.33, 111.15, 112.19, 122.42, 125.58, 126.21, 129.18, 137.71, 141.23, 166.11. ESI-HRMS ([M+H]$^+$, m/z): Calcd for $C_{11}H_{10}O_2N_3$ 216.07675, found 216.07639. IR (cm^{-1}): 2241 (CN group). Purity: UHPLC (Method I, 254 nm): tr = 4.303 min, 96.2% total area.

Methyl 2-cyano-1-methyl-1*H*-benzo[*d*]imidazole-6-carboxylate (**36**). Yield: 295 mg (8%) of yellow crystals. ^{1}H NMR (400 MHz, $CDCl_3$) δ 3.95 (s, 3H), 4.04 (s, 3H), 7.83 (d, *J* = 8.7 Hz, 1H), 8.04 (dd, *J* = 8.7, 1.5 Hz, 1H), 8.14 – 8.19 (m, 1H). ^{13}C NMR (101 MHz, $CDCl_3$) δ 31.67, 52.62, 110.61, 112.86, 121.49, 125.33, 128.19, 129.45, 134.45, 145.46, 166.60. ESI-HRMS ([M+H]$^+$, m/z): Calcd for $C_{11}H_{10}O_2N_3$ 216.07675, found 216.07643. IR (cm^{-1}): 2241 (CN group). Purity: UHPLC (Method I, 254 nm): tr = 4.317 min, 97.4% total area.

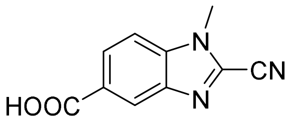

2-Cyano-1-methyl-1*H*-benzo[*d*]imidazole-5-carboxylic acid (**29**) was prepared following *GP5* from methyl 2-cyano-1-methyl-1*H*-benzo[*d*]imidazole-5-carboxylate (230 mg, 1.07 mmol), and isolated by column chromatography on silica (DCM/MeOH= 15:1) and subsequent crystallization (EtOH). Yield: 46 mg (21%) of beige solid. ^{1}H NMR (400 MHz, DMSO-d_6) δ 4.03 (s, 3H), 7.88 (dd, *J* = 8.7, 0.6 Hz, 1H), 8.08 (dd, *J* = 8.7, 1.5 Hz, 1H), 8.36 (dd, *J* = 1.5, 0.6 Hz, 1H), 13.08 (br s, 1H). ^{13}C NMR (101 MHz, DMSO-d_6) δ 31.74, 111.24, 111.93, 122.48, 126.51, 126.81, 128.94, 137.55, 141.30, 167.19. ESI-HRMS ([M+H]$^+$, m/z): Calcd for

$C_{10}H_8O_2N_3$ 202.06110, found 202.06075. IR (cm^{-1}): 2240 (CN group). Purity: UHPLC (Method I, 280 nm): tr = 3.647 min, 96.8% total area.

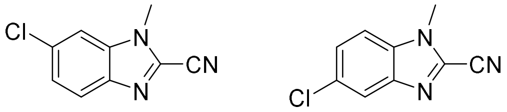

5-Chloro-1-methyl-1*H*-benzo[*d*]imidazole-2-carbonitrile (**30**) and 6-chloro-1-methyl-1*H*-benzo[*d*]imidazole-2-carbonitrile (**38**) were prepared following *GP3* from 5-chloro-1*H*-benzo[*d*]imidazole-2-carbonitrile (2.90 g, 16.3 mmol), and isolated by column chromatography on silica (EtOAc/*n*-hex = 1:3) and subsequent RP-CC (*GP7*). Overall yield: 1.28 g (41%).

5-Chloro-1-methyl-1*H*-benzo[*d*]imidazole-2-carbonitrile (**30**). Yield: 781 mg (25%) of yellow crystals. ^{1}H NMR (400 MHz, $CDCl_3$) δ 4.01 (s, 3H), 7.38 (dd, *J* = 8.8, 0.6 Hz, 1H), 7.47 (dd, *J* = 8.8, 1.9 Hz, 1H), 7.84 (dd, *J* = 1.9, 0.6 Hz, 1H). ^{13}C NMR (101 MHz, $CDCl_3$) δ 31.63, 110.71, 111.31, 121.20, 127.24, 128.13, 130.45, 133.37, 143.15. ESI-HRMS ([M+H]$^+$, m/z): Calcd for $C_9H_7N_3Cl$ 192.03230, found 192.03209. IR (cm^{-1}): 2242 (CN group). Purity: UHPLC (Method I, 254 nm): tr = 4.530 min, 95.3% total area.

6-Chloro-1-methyl-1*H*-benzo[*d*]imidazole-2-carbonitrile (**38**). Yield: 500 mg (16%) of yellow crystals. ^{1}H NMR (400 MHz, $CDCl_3$) δ 3.98 (s, 3H), 7.38 (dd, *J* = 8.8, 1.9 Hz, 1H), 7.45 (dd, *J* = 1.9, 0.6 Hz, 1H), 7.77 (dd, *J* = 8.8, 0.6 Hz, 1H). ^{13}C NMR (101 MHz, $CDCl_3$) δ 31.56, 110.45, 110.78, 122.72, 125.62, 127.81, 132.81, 135.33, 141.16. ESI-HRMS ([M+H]$^+$, m/z): Calcd for $C_9H_7N_3Cl$ 192.03230, found 192.03206. IR (cm^{-1}): 2241 (CN group). Purity: UHPLC (Method I, 254 nm): tr = 4.530 min, 97.4% total area.

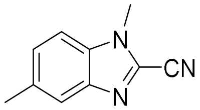

1,5-Dimethyl-1*H*-benzo[*d*]imidazole-2-carbonitrile (**31**)

4-Methyl-2-nitroaniline (2.0 g, 13.1 mmol) was dissolved in anhydrous DMF (15 mL), flushed with Ar, cooled on an icebath, then NaH (60% dispersion on mineral oil, 524 mg, 13.1 mmol) was added, the reaction mixture stirred for 15 min followed by the addition of MeI (0.90 mL, 14.4 mmol). The reaction mixture was stirred under Ar at rt for 18 h, volatile components evaporated *in vacuo* and the residue dissolved in EtOAc (50 mL), washed with H_2O (30 mL), saturated brine (30 mL), dried, filtered, solvents evaporated *in vacuo* and *N,*4-dimethyl-2-

nitroaniline (**118)** isolated by crystallization (EtOH). Yield: 2.05 g (94%) of yellow crystals. ^{1}H NMR (400 MHz, $CDCl_3$) δ 2.26 (s, 3H), 3.00 (d, *J* = 5.1 Hz, 3H), 6.75 (d, *J* = 8.7 Hz, 1H), 7.28 (dd, *J* = 8.7, 2.2 Hz, 1H), 7.92 (s, 1H), 7.95 (dd, *J* = 2.1, 1.0 Hz, 1H). ^{13}C NMR (101 MHz, $CDCl_3$) δ 20.06, 29.87, 113.46, 124.80, 126.08, 131.55, 137.93, 144.75. ESI-HRMS ($[M+H]^+$, m/z): Calcd for $C_8H_{11}O_2N_2$ 167.08150, found 167.08140.

*N,*4-Dimethyl-2-nitroaniline (**118)** (2.0 g, 12.0 mmol) was dissolved in MeOH (30 mL), flushed with Ar, then 10% Pd/C (75 mg) was added, and $NaBH_4$ was added in 100 mg portions with stirring on an icebath until the yellow solution turned greyish. The catalyst was removed by filtration, washed with MeOH, and the filtrate evaporated *in vacuo*. Oily residue was acidified with 6 M $HCl_{(aq)}$ (40 mL), then made alkaline (pH = 13–14) using 50% $NaOH_{(aq)}$, extracted with EtOAc (2 × 30 mL), organic layer washed with saturated brine (30 mL), dried, filtered and volatile components evaporated *in vacuo* to afford crude *N*^{1},4-dimethylbenzene-1,2-diamine **119**. Yield: 1.63 g (quantitative) of greyish semisolid. ESI-HRMS ($[M+H]^+$, m/z): Calcd for $C_8H_{13}N_2$ 137.10732, found 137.10716.

1,5-Dimethyl-1*H*-benzo[*d*]imidazole-2-carbonitrile (**31**) was prepared following *GP1* from *N*^{1},4-dimethylbenzene-1,2-diamine (**119)** (1.63 g, 12.0 mmol), and isolated by column chromatography on silica (EtOAc/*n*-hex = 1:3) and subsequent crystallization (EtOH). Yield: 267 mg (13%) of brown crystals. ^{1}H NMR (400 MHz, Acetone-d_6) δ 2.47 (s, 3H), 4.05 (s, 3H), 7.32–7.38 (m, 1H), 7.53–7.58 (m, 2H). ^{13}C NMR (101 MHz, Acetone-d_6) δ 21.47, 31.73, 111.53, 112.29, 121.13, 127.83, 128.66, 134.25, 134.71, 143.80. ESI-HRMS ($[M+H]^+$, m/z): Calcd for $C_{10}H_{10}N_3$ 172.08692, found 172.08689. IR (cm^{-1}): 2233 (CN group). Purity: UHPLC (Method I, 254 nm): tr = 4.423 min, 95.5% total area.

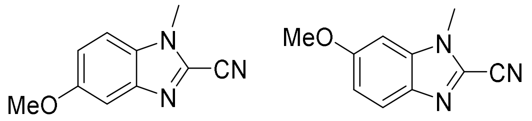

5-Methoxy-1-methyl-1*H*-benzo[*d*]imidazole-2-carbonitrile (**32**) and 6-methoxy-1-methyl-1*H*-benzo[*d*]imidazole-2-carbonitrile (**40**) were prepared following *GP3* from methyl 5-methoxy-1*H*-benzo[*d*]imidazole-2-carbonitrile (320 mg, 1.85 mmol), and isolated by column chromatography on silica (EtOAc/*n*-hex= 1:2) and subsequent crystallization (EtOH). Overall yield: 129 mg (37%).

5-Methoxy-1-methyl-1*H*-benzo[*d*]imidazole-2-carbonitrile (**32**). Yield: 46 mg (13%) of yellow crystals. ^{1}H NMR (400 MHz, $CDCl_3$) δ 3.86 (s, 3H), 3.96 (s, 3H), 7.13 (dd, *J* = 9.0, 2.4 Hz, 1H), 7.20 (d, *J* = 2.3 Hz, 1H), 7.27 – 7.32 (m, 1H). ^{13}C NMR (101 MHz, $CDCl_3$) δ 31.52, 55.85, 101.90, 110.85, 111.32, 118.29, 126.73, 129.67, 143.60, 157.86. ESI-HRMS ($[M+H]^+$, m/z): Calcd for $C_{10}H_{10}ON_3$ 188.08184, found 188.08157. IR (cm^{-1}): 2239 (CN group). Purity: UHPLC (Method I, 254 nm): tr = 4.217 min, 99.6% total area.

6-Methoxy-1-methyl-1*H*-benzo[*d*]imidazole-2-carbonitrile (**40**). Yield: 83 mg (24%) of brown crystals. ^{1}H NMR (400 MHz, $CDCl_3$) δ 3.89 (s, 3H), 3.92 (s, 3H), 6.75 (d, *J* = 2.3 Hz, 1H), 7.02 (dd, *J* = 9.0, 2.4 Hz, 1H), 7.68 (d, *J* = 9.0 Hz, 1H). ^{13}C NMR (101 MHz, $CDCl_3$) δ 31.29, 55.95, 92.04, 111.44, 115.65, 122.41, 125.77, 135.79, 137.22, 159.61. ESI-HRMS ($[M+H]^+$, m/z): Calcd for $C_{10}H_{10}ON_3$ 188.08184, found 188.08183. IR (cm^{-1}): 2232 (CN group). Purity: UHPLC (Method I, 254 nm): tr = 4.247 min, 99.5% total area.

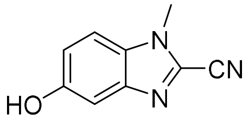

5-Hydroxy-1-methyl-1*H*-benzo[*d*]imidazole-2-carbonitrile (**33**) was prepared following *GP6* from 5-methoxy-1-methyl-1*H*-benzo[*d*]imidazole-2-carbonitrile (50 mg, 0.27 mmol), and isolated by column chromatography on silica (EtOAc/*n*-hex = 1:1). Yield: 18 mg (38%) of off-white solid. ^{1}H NMR (400 MHz, CD_3OD) δ 3.97 (s, 3H), 7.03 (dd, *J* = 2.3, 0.7 Hz, 1H), 7.06 (dd, *J* = 8.8, 2.3 Hz, 1H), 7.44 (dd, *J* = 8.8, 0.7 Hz, 1H). ^{13}C NMR (101 MHz, CD_3OD) δ 31.87, 104.38, 111.91, 112.58, 118.69, 127.71, 130.43, 144.18, 156.82. ESI-HRMS ($[M+H]^+$, m/z): Calcd for $C_9H_8ON_3$ 174.06619, found 174.06612. IR (cm^{-1}): 2237 (CN group). Purity: UHPLC (Method II, 254 nm): tr = 4.013 min, 97.5% total area.

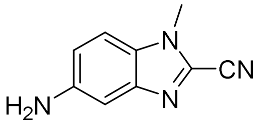

5-Amino-1-methyl-1*H*-benzo[*d*]imidazole-2-carbonitrile (**34**) was prepared following *GP4* from 1-methyl-5-nitro-1*H*-benzo[*d*]imidazole-2-carbonitrile (1.16 g, 1.58 mmol), and isolated by column chromatography on silica (EtOAc/*n*-hex= 2:1). Yield: 161 mg (16%) of pale-brown crystals. ^{1}H NMR (400 MHz, CD_3OD) δ 3.96 (s, 3H), 6.92 – 6.98 (m, 1H), 6.98 – 7.04 (m, 1H), 7.38 (d, *J* = 8.8 Hz, 1H). ^{13}C NMR (101 MHz, CD_3OD) δ 31.77, 103.82, 112.08, 112.33,

119.30, 127.01, 129.92, 144.51, 147.24. ESI-HRMS ([M+H]$^+$, m/z): Calcd for $C_9H_9N_4$ 173.08217, found 173.08213. IR (cm^{-1}): 2231 (CN group). Purity: UHPLC (Method II, 254 nm): tr = 1.963 min, 95.5% total area.

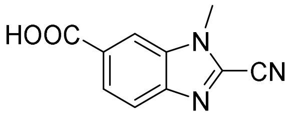

2-Cyano-1-methyl-1*H*-benzo[*d*]imidazole-6-carboxylic acid (**37**) was prepared following *GP5* from methyl 2-cyano-1-methyl-1*H*-benzo[*d*]imidazole-6-carboxylate (100 mg, 0.46 mmol), and isolated by column chromatography on silica (DCM/MeOH= 15:1) and subsequent crystallization (EtOH). Yield: 22 mg (24%) of white solid. ^{1}H NMR (400 MHz, DMSO-*d$_6$*) δ 4.01 (s, 3H), 7.76 (d, *J* = 8.6 Hz, 1H), 8.02 (dd, *J* = 8.6, 1.5 Hz, 1H), 8.30 (s, 1H). ^{13}C NMR (101 MHz, DMSO-*d$_6$*) δ 31.57, 111.45, 113.11, 119.66, 125.23, 128.31, 133.12, 134.45, 143.60, 169.71. ESI-HRMS ([M+H]$^+$, m/z): Calcd for $C_{10}H_8O_2N_3$ 202.06110, found 202.06079. IR (cm^{-1}): 2245 (CN group). Purity: UHPLC (Method I, 280 nm): tr = 3.703 min, 95.8% total area.

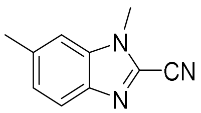

1,6-Dimethyl-1*H*-benzo[*d*]imidazole-2-carbonitrile (**39**)

5-Methyl-2-nitroaniline (5.0 g, 32.9 mmol) was dissolved in anhydrous DMF (30 mL), flushed with Ar, cooled on an icebath, then NaH (60% dispersion on mineral oil, 1.32 g, 32.9 mmol) was added, the reaction mixture stirred for 15 min followed by the addition of MeI (2.25 mL, 36.2 mmol). The reaction mixture was stirred under Ar at rt for 18 h, volatile components evaporated *in vacuo* and the residue dissolved in EtOAc (60 mL), washed with H_2O (40 mL), saturated brine (40 mL), dried, filtered, solvents evaporated *in vacuo* and *N,*5-dimethyl-2-nitroaniline (**120**) isolated by crystallization (EtOH). Yield: 3.55 g (65%) of orange crystals. ^{1}H NMR (400 MHz, $CDCl_3$) δ 2.35 (s, 3H), 3.01 (d, *J* = 5.1 Hz, 3H), 6.46 (dd, *J* = 8.7, 1.7 Hz, 1H), 6.60 (d, *J* = 1.6 Hz, 1H), 8.05 (d, *J* = 8.7 Hz, 1H). 8.06 (br s, 1H). ^{13}C NMR (101 MHz, $CDCl_3$) δ 22.33, 29.81, 113.09, 117.08, 126.89, 130.17, 146.53, 147.98. ESI-HRMS ([M+H]$^+$, m/z): Calcd for $C_8H_{11}O_2N_2$ 167.08150, found 167.08133.

*N,*5-Dimethyl-2-nitroaniline (**120**) (3.50 g, 21.1 mmol) was dissolved in MeOH (40 mL), flushed with Ar, then 10% Pd/C (100 mg) was added, and $NaBH_4$ was added in 100 mg portions with stirring on an icebath until the yellow solution turned greyish. The catalyst was removed by filtration, washed with MeOH, and the filtrate evaporated *in vacuo*. Oily residue was acidified with 6 M $HCl_{(aq)}$ (50 mL), then made alkaline (pH = 13–14) using 50% $NaOH_{(aq)}$, extracted with EtOAc (2 × 40 mL), organic layer washed with saturated brine (40 mL), dried, filtered and volatile components evaporated *in vacuo* to afford crude N^1,5-dimethylbenzene-1,2-diamine (**121**). Yield: 0.98 g (34%) of dark-brown semisolid. ESI-HRMS ($[M+H]^+$, m/z): Calcd for $C_8H_{13}N_2$ 137.10732, found 137.10716.

1,6-Dimethyl-1*H*-benzo[*d*]imidazole-2-carbonitrile (**39**) was prepared following *GP1* from N^1,4-dimethylbenzene-1,2-diamine (**121**) (980 mg, 7.20 mmol), and isolated by column chromatography on silica (1. PE/DCM= 3:1; 2. DCM; 3. DCM/MeOH= 50:1) and subsequent crystallization (EtOH). Yield: 126 mg (10%) of pale-brown solid. ^{1}H NMR (400 MHz, $CDCl_3$) δ 2.53 (d, *J* = 0.8 Hz, 3H), 3.94 (s, 3H), 7.18 – 7.20 (m, 1H), 7.22 (dd, *J* = 8.4, 1.5 Hz, 1H), 7.70 (dd, *J* = 8.4, 0.7 Hz, 1H). ^{13}C NMR (101 MHz, $CDCl_3$) δ 22.16, 31.26, 109.92, 111.36, 121.19, 126.44, 126.58, 135.12, 137.20, 140.87. ESI-HRMS ($[M+H]^+$, m/z): Calcd for $C_{10}H_{10}N_3$ 172.08692, found 172.08671. IR (cm^{-1}): 2233 (CN group). Purity: UHPLC (Method I, 254 nm): tr = 4.413 min, 99.8% total area.

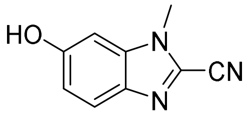

Reaction of 6-methoxy-1-methyl-1*H*-benzo[*d*]imidazole-2-carbonitrile (50 mg, 0.27 mmol) according to *GP6* failed to produce 6-hydroxy-1-methyl-1*H*-benzo[*d*]imidazole-2-carbonitrile (**41**) in sufficient purity. 6-Hydroxy-1-methyl-1*H*-benzo[*d*]imidazole-2-carbonitrile (**41**) was prepared by deprotecting 6-methoxy-1-methyl-1*H*-benzo[*d*]imidazole-2-carbonitrile (100 mg, 0.53 mmol) according to *GP5*, and isolated by column chromatography on silica (1. EtOAc/*n*-hex = 1:1; 2. EtOAc). Yield: 8 mg (9%) of yellow solid. ^{1}H NMR (400 MHz, DMSO-d_6) δ 3.87 (s, 3H), 6.92 (d, *J* = 8.2 Hz, 2H), 7.55 – 7.63 (m, 1H), 9.94 (s, 1H). ^{13}C NMR (101 MHz, DMSO-d_6) δ 31.12, 95.23, 111.96, 115.31, 121.36, 124.89, 135.67, 136.09, 156.85. ESI-HRMS ($[M+H]^+$, m/z): Calcd for $C_9H_8ON_3$ 174.06619, found 174.06609. IR (cm^{-1}): 2236 (CN group). Purity: UHPLC (Method I, 280 nm): tr = 3.260 min, 95.1% total area.

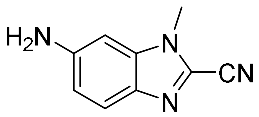

6-Amino-1-methyl-1*H*-benzo[*d*]imidazole-2-carbonitrile (**42**) was prepared following *GP4* from 1-methyl-6-nitro-1*H*-benzo[*d*]imidazole-2-carbonitrile (445 mg, 2.20 mmol), and isolated by column chromatography on silica (DCM/MeOH= 20:1) and subsequent RP-CC (*GP7*). Yield: 36 mg (10%) of off-white crystals. ^{1}H NMR (400 MHz, DMSO-*d*$_6$) δ 3.79 (s, 3H), 5.57 (br s, 2H), 6.55 (d, *J* = 2.0 Hz, 1H), 6.75 (dd, *J* = 8.8, 2.1 Hz, 1H), 7.42 (d, *J* = 8.8 Hz, 1H). ^{13}C NMR (101 MHz, DMSO-*d*$_6$) δ 30.80, 91.28, 112.42, 115.12, 120.99, 122.92, 134.10, 136.67, 148.51. ESI-HRMS ([M+H]$^+$, m/z): Calcd for $C_9H_9N_4$ 173.08217, found 173.08217. IR (cm^{-1}): 2227 (CN group). Purity: UHPLC (Method II, 254 nm): tr = 2.417 min, 97.6% total area.

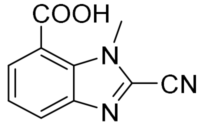

2-Cyano-1-methyl-1*H*-benzo[*d*]imidazole-7-carboxylic acid (**45**) was prepared following *GP5* from methyl 2-cyano-1-methyl-1*H*-benzo[*d*]imidazole-7-carboxylate (100 mg, 0.47 mmol), and isolated by column chromatography on silica (1. DCM/MeOH= 20:1; 2. DCM/MeOH= 4:1) and subsequent crystallization (EtOH). Yield: 38 mg (40%) of white solid. ^{1}H NMR (400 MHz, DMSO-*d*$_6$) δ 4.11 (s, 3H), 7.48 (dd, *J* = 8.2, 7.5 Hz, 1H), 7.95 (dd, *J* = 7.5, 1.1 Hz, 1H), 8.04 (dd, *J* = 8.3, 1.1 Hz, 1H), 13.58 (br s, 1H). ^{13}C NMR (101 MHz, DMSO-*d*$_6$) δ 35.53, 111.51, 119.17, 123.53, 125.11, 128.75, 129.62, 132.16, 143.58, 166.52. ESI-HRMS ([M+H]$^+$, m/z): Calcd for $C_{10}H_8O_2N_3$ 202.06110, found 202.066. IR (cm^{-1}): 2241 (CN group). Purity: UHPLC (Method I, 254 nm): tr = 3.763 min, 97.2% total area.

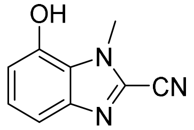

7-Hydroxy-1-methyl-1*H*-benzo[*d*]imidazole-2-carbonitrile (**49**) was prepared following *GP6* from 7-methoxy-1-methyl-1*H*-benzo[*d*]imidazole-2-carbonitrile (50 mg, 0.27 mmol), and isolated by column chromatography on silica (EtOAc/*n*-hex = 1:1) and subsequent RP-CC (*GP7*). Yield: 18 mg (38%) of white crystals. ^{1}H NMR (400 MHz, DMSO-*d*$_6$) δ 4.16 (s, 3H),

6.79 (dd, J = 7.4, 1.2 Hz, 1H), 7.14 (dd, J = 8.3, 7.4 Hz, 1H), 7.19 (dd, J = 8.3, 1.2 Hz, 1H), 10.48 (s, 1H). ^{13}C NMR (101 MHz, DMSO-d_6) δ 33.80, 109.88, 111.31, 111.62, 124.57, 124.77, 126.69, 144.16, 145.29. ESI-HRMS ([M+H]$^+$, m/z): Calcd for $C_9H_8ON_3$ 174.06619, found 174.06636. IR (cm^{-1}): 2237 (CN group). Purity: UHPLC (Method II, 254 nm): tr = 4.207 min, 99.1% total area.

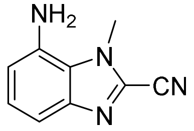

7-Amino-1-methyl-1*H*-benzo[*d*]imidazole-2-carbonitrile (**50**) was prepared following *GP4* from 1-methyl-7-nitro-1*H*-benzo[*d*]imidazole-2-carbonitrile (50 mg, 0.25 mmol), and isolated by column chromatography on silica (1. EtOAc/*n*-hex= 2:1; 1. EtOAc/*n*-hex= 4:1) and subsequent RP-CC (*GP7*). Yield: 3 mg (7%) of brown solid. ^{1}H NMR (400 MHz, $CDCl_3$) δ 3.84 (s, 2H), 4.29 (s, 3H), 6.71 (dd, J = 7.5, 0.9 Hz, 1H), 7.16 (dd, J = 8.4, 7.5 Hz, 1H), 7.32 (dd, J = 8.3, 0.9 Hz, 1H). ^{13}C NMR (101 MHz, $CDCl_3$) δ 33.99, 111.23, 113.58, 113.63, 125.32, 125.88, 127.50, 133.01, 144.63. ESI-HRMS ([M+H]$^+$, m/z): Calcd for $C_9H_9N_4$ 173.08217, found 173.08222. IR (cm^{-1}): 2237 (CN group). Purity: UHPLC (Method I, 254 nm): tr = 1.997 min, 97.8% total area.

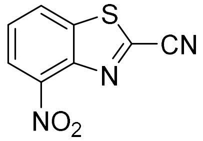

4-Nitrobenzo[*d*]thiazole-2-carbonitrile (**52**) was prepared following *GP2* from 2-bromo-6-nitroaniline (400 mg, 1.84 mmol), and isolated by column chromatography on silica (1. EtOAc/*n*-hex = 1:2; 2. EtOAc/*n*-hex = 1:1). Yield: 117 mg (31%) of orange solid. ^{1}H NMR (400 MHz, $CDCl_3$) δ 7.82 (t, J = 8.1 Hz, 1H), 8.32 (dd, J = 8.2, 1.1 Hz, 1H), 8.38 (dd, J = 7.9, 1.1 Hz, 1H). ^{13}C NMR (101 MHz, $CDCl_3$) δ 112.17, 124.59, 127.53, 128.42, 138.28, 140.44, 143.86, 144.41. ESI-HRMS ([M+H]$^+$, m/z): Calcd for $C_8H_4O_2N_3S$ 206.00187, found 206.00136. IR (cm^{-1}): 2230.85 (CN group). Purity: UHPLC (Method III, 254 nm): tr = 4.277 min, 98.1% total area.

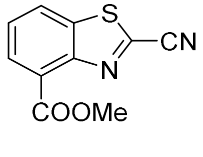

Methyl 2-cyanobenzo[*d*]thiazole-4-carboxylate (**53**) was prepared following *GP2* from methyl 2-amino-3-bromobenzoate (1.0 g, 4.35 mmol), and isolated by column chromatography on silica (EtOAc/*n*-hex = 1:4). Yield: 390 mg (41%) of orange solid. ^{1}H NMR (400 MHz, $CDCl_3$) δ 4.05 (s, 3H), 7.68 – 7.74 (m, 1H), 8.18 (dd, *J* = 8.3, 1.2 Hz, 1H), 8.25 (dd, *J* = 7.4, 1.2 Hz, 1H). ^{13}C NMR (101 MHz, $CDCl_3$) δ 53.04, 112.83, 126.01, 127.40, 128.20, 130.66, 136.96, 138.35, 150.02, 165.61. ESI-HRMS ($[M+H]^+$, m/z): Calcd for $C_{10}H_7O_2N_2S$ 219.02227, found 219.02166. IR (cm^{-1}): 2229 (CN group). Purity: UHPLC (Method III, 254 nm): tr = 4.343 min, 99.0% total area.

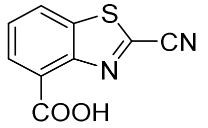

Synthesis of 2-cyanobenzo[*d*]thiazole-4-carboxylic acid (**54**) following *GP5* from methyl 2-cyanobenzo[*d*]thiazole-4-carboxylate (360 mg, 1.65 mmol), and isolation by column chromatography on silica (DCM/MeOH= 4:1) failed to produce the desired product.

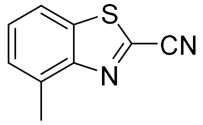

4-Methylbenzo[*d*]thiazole-2-carbonitrile (**56**) was prepared following *GP2* from 2-bromo-6-methylaniline (400 mg, 2.15 mmol), and isolated by column chromatography on silica (EtOAc/*n*-hex = 1:6). Yield: 8 mg (2%) of yellow solid. ^{1}H NMR (400 MHz, $CDCl_3$) δ 2.80 (s, 3H), 7.43 (dt, *J* = 7.3, 1.1 Hz, 1H), 7.49 – 7.55 (m, 1H), 7.80 (dd, *J* = 8.1, 1.0 Hz, 1H). ^{13}C NMR (101 MHz, $CDCl_3$) δ 18.43, 113.38, 119.24, 128.32, 128.84, 135.31, 135.53, 135.92, 152.05. ESI-HRMS ($[M+H]^+$, m/z): Calcd for $C_9H_7N_2S$ 175.03245, found 175.03208. IR (cm^{-1}): 2228 (CN group). Purity: UHPLC (Method III, 254 nm): tr = 5.010 min, 99.0% total area.

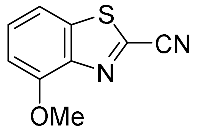

4-Methoxybenzo[*d*]thiazole-2-carbonitrile (**57**). Commercially available. ^{1}H NMR (400 MHz, DMSO-d_6) δ 4.00 (s, 3H), 7.22 (dd, *J* = 8.1, 0.9 Hz, 1H), 7.67 (t, *J* = 8.1 Hz, 1H), 7.84 (dd, *J* = 8.3, 0.9 Hz, 1H). HRMS (ESI$^+$) *m/z* $[M+H]^+$, calcd. for $C_9H_7N_2OS$ 191.02736, found 191.02705. Purity: UHPLC (Method I, 254 nm): tr = 4.500 min, 95.5% total area.

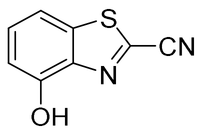

4-Hydroxybenzo[*d*]thiazole-2-carbonitrile (**58**) was prepared following *GP6* from 4-methoxybenzo[*d*]thiazole-2-carbonitrile (50 mg, 0.26 mmol), and isolated by column chromatography on silica (EtOAc/hex = 1:4). Yield: 34 mg (74%) of yellow solid. ^{1}H NMR (400 MHz, $CDCl_3$) δ 6.84 (br s, 1H), 7.12 (dd, *J* = 7.8, 1.0 Hz, 2H), 7.48 (dd, *J* = 8.2, 1.0 Hz, 1H), 7.55 (t, *J* = 8.0 Hz, 1H). ^{13}C NMR (101 MHz, $CDCl_3$) δ 112.00, 112.93, 113.18, 130.98, 134.75, 136.16, 142.34, 151.56. ESI-HRMS ($[M+H]^+$, m/z): Calcd for $C_8H_5ON_2S$ 177.01171, found 177.01131. IR (cm^{-1}): 2229 (CN group). Purity: UHPLC (Method III, 254 nm): tr = 4.130 min, 99.0% total area.

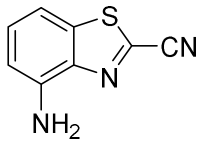

4-Aminobenzo[*d*]thiazole-2-carbonitrile (**59**) was prepared following *GP4* from 4-nitrobenzo[*d*]thiazole-2-carbonitrile (102 mg, 0.50 mmol), and isolated by column chromatography on silica (EtOAc/*n*-hex = 1:1). Yield: 31 mg (35%) of orange solid. ^{1}H NMR (400 MHz, $CDCl_3$) δ 4.78 (br s, 2H), 6.79 (dd, *J* = 7.8, 0.9 Hz, 1H), 7.24 (dd, *J* = 8.1, 1.0 Hz, 1H), 7.38 (t, *J* = 8.0 Hz, 1H). ^{13}C NMR (101 MHz, $CDCl_3$) δ 109.94, 110.37, 113.49, 130.48, 132.81, 136.59, 141.40, 143.18. ESI-HRMS ($[M+H]^+$, m/z): Calcd for $C_8H_6N_3S$ 176.02769, found 176.02733. IR (cm^{-1}): 2223 (CN group). Purity: UHPLC (Method III, 254 nm): tr = 4.370 min, 96.4% total area.

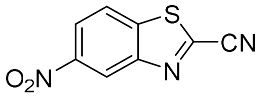

5-Nitrobenzo[*d*]thiazole-2-carbonitrile (**60**). Commercially available. ^{1}H NMR (400 MHz, DMSO-d_6) δ 8.52 (dd, *J* = 9.0, 2.2 Hz, 1H), 8.63 (d, *J* = 9.0 Hz, 1H), 9.09 (d, *J* = 2.3 Hz, 1H). HRMS (ESI^+) *m/z* $[M+H]^+$, calcd. for $C_8H_4N_3O_2S$ 206.00187, found 206.00168. Purity: UHPLC (Method I, 254 nm): tr = 4.530 min, 96.4% total area.

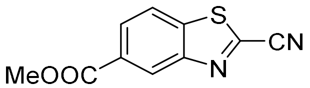

Methyl 2-cyanobenzo[*d*]thiazole-5-carboxylate (**61**) was prepared following *GP2* from methyl 3-amino-4-bromobenzoate (330 mg, 1.43 mmol), and isolated by column chromatography on silica (EtOAc/*n*-hex = 1:3). Yield: 160 mg (50%) of yellow solid. ^{1}H NMR (400 MHz, $CDCl_3$) δ 4.01 (s, 3H), 8.06 (dd, *J* = 8.6, 0.5 Hz, 1H), 8.30 (dd, *J* = 8.6, 1.6 Hz, 1H), 8.89 (d, *J* = 1.5 Hz, 1H). ^{13}C NMR (101 MHz, $CDCl_3$) δ 52.88, 112.69, 121.99, 127.05, 129.07, 130.57, 138.20, 139.61, 152.26, 166.06. ESI-HRMS ($[M+H]^+$, m/z): Calcd for $C_{10}H_7O_2N_2S$ 219.02227, found 219.02175. IR (cm^{-1}): 2232 (CN group). Purity: UHPLC (Method III, 254 nm): tr = 4.707min, 97.0% total area.

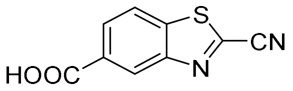

2-Cyanobenzo[*d*]thiazole-5-carboxylic acid (**62**) was prepared following *GP5* from methyl 2-cyanobenzo[*d*]thiazole-5-carboxylate (80 mg, 0.37 mmol), and isolated by column chromatography on silica (1. DCM/MeOH= 9:1; 2. DCM/MeOH/AcOH= 9:1:0.1). Yield: 35 mg (2%) of yellow solid. ^{1}H NMR (400 MHz, DMSO-d_6) δ 8.23 (dd, *J* = 8.6, 1.5 Hz, 1H), 8.41 (d, *J* = 8.6 Hz, 1H), 8.70 (dd, *J* = 1.6, 0.6 Hz, 2H). ^{13}C NMR (101 MHz, DMSO-d_6) δ 113.28, 123.16, 125.21, 128.77, 138.64, 138.73, 151.50, 167.13, 172.06. ESI-HRMS ($[M–H]^-$, m/z): Calcd for $C_9H_3O_2N_2S$ 202.99207, found 202.99114. IR (cm^{-1}): 2237 (CN group). Purity: UHPLC (Method III, 254 nm): tr = 4.123min, 96.6% total area.

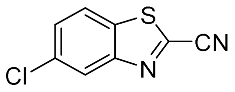

5-Chlorobenzo[*d*]thiazole-2-carbonitrile (**63**) was prepared following *GP1* from 2-amino-4-chlorobenzenethiol (500 mg, 3.13 mmol), and isolated by column chromatography on silica (EtOAc/*n*-hex = 1:6). Yield: 40 mg (7%) of orange solid. ^{1}H NMR (400 MHz, DMSO-d_6) δ 7.79 (dd, *J* = 8.8, 2.0 Hz, 1H), 8.37 – 8.43 (m, 2H). NMR (101 MHz, DMSO-d_6) δ 113.17, 123.86, 124.78, 128.86, 132.99, 134.29, 139.43, 152.34. ESI-HRMS ($[M+H]^+$, m/z): Calcd for $C_8H_4N_2ClS$ 194.97782, found 194.97744. IR (cm^{-1}): 2235 (CN group). Purity: UHPLC (Method III, 254 nm): tr = 4.997 min, 98.1% total area.

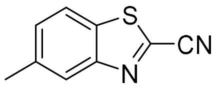

5-Methylbenzo[*d*]thiazole-2-carbonitrile (**64**) was prepared following *GP2* from 2-bromo-5-methylaniline (0.5 g, 2.17 mmol), and isolated by column chromatography on silica (EtOAc/*n*-hex = 1:6). Yield: 95 mg (57%) of yellow solid. ^{1}H NMR (400 MHz, $CDCl_3$) δ 2.56 (s, 3H), 7.46 (dd, *J* = 8.4, 1.6 Hz, 1H), 7.85 (d, *J* = 8.3 Hz, 1H), 7.98 – 8.04 (m, 1H). ^{13}C NMR (101 MHz, $CDCl_3$) δ 152.77, 138.44, 136.44, 132.46, 130.53, 124.95, 121.19, 113.14, 21.49. ESI-HRMS ($[M+H]^+$, m/z): Calcd for $C_9H_7N_2S$ 175.03245, found 175.03204. IR (cm^{-1}): 2228 (CN group). Purity: UHPLC (Method III, 254 nm): tr = 4.933 min, 99.0% total area.

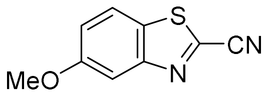

5-Methoxybenzo[*d*]thiazole-2-carbonitrile (**65**). Commercially available. ^{1}H NMR (400 MHz, DMSO-d_6) δ 3.89 (s, 3H), 7.37 (dd, *J* = 9.0, 2.5 Hz, 1H), 7.77 (d, *J* = 2.5 Hz, 1H), 8.21 (d, *J* = 9.0 Hz, 1H). HRMS (ESI^+) *m/z* $[M+H]^+$, calcd. for $C_9H_7N_2OS$ 191.02736, found 191.02712. Purity: UHPLC (Method I, 254 nm): tr = 4.673 min, 98.2% total area.

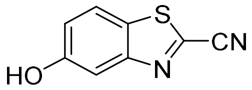

5-Hydroxybenzo[*d*]thiazole-2-carbonitrile (**66**) was prepared following *GP6* from 5-methoxybenzo[*d*]thiazole-2-carbonitrile (50 mg, 0.26 mmol), and isolated by column chromatography on silica (EtOAc/hex = 1:3). Yield: 30 mg (65%) of yellow solid. ^{1}H NMR (400 MHz, Acetone-d_6) δ 7.30 (dd, *J* = 8.8, 2.2 Hz, 1H), 7.57 (d, *J* = 2.0 Hz, 1H), 8.02 – 8.09 (m, 1H), 9.16 (br s, 1H). ^{13}C NMR (101 MHz, Acetone-d_6) δ 109.56, 114.01, 120.35, 120.41, 123.72, 127.34, 138.03, 154.75, 158.82. ESI-HRMS ($[M+H]^+$, m/z): Calcd for $C_8H_5ON_2S$ 177.01171, found 177.01129. IR (cm^{-1}): 2240 (CN group). Purity: UHPLC (Method III, 254 nm): tr = 4.110 min, 96.5% total area.

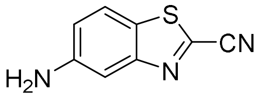

5-Aminobenzo[*d*]thiazole-2-carbonitrile (**67**). Commercially available. ^{1}H NMR (400 MHz, DMSO-d_6) δ 5.67 (br s, 2H), 7.05 (dd, *J* = 8.8, 2.2 Hz, 1H), 7.24 (d, *J* = 2.2 Hz, 1H), 7.90 (d, *J* = 8.8 Hz, 1H). HRMS (ESI^+) *m/z* $[M+H]^+$, calcd. for $C_8H_6N_3S$ 176.02769, found 176.02769. Purity: UHPLC (Method I, 254 nm): tr = 1.853 min, 98.2% total area.

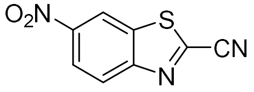

6-Nitrobenzo[*d*]thiazole-2-carbonitrile (**68**). To a suspension of 2-chloro-6-nitrobenzo[*d*]thiazole (150 mg, 0.70 mmol) in MeCN (10 mL), DABCO (12 mg, 0.11 mmol) was added, followed by a solution of NaCN (41 mg, 0.84 mmol) in H_2O (1 mL). After stirring the reaction mixture at rt for 24 h, a solution of $FeCl_{3(aq)}$ (10 mL) was added, extracted with EtOAc (3 × 20 mL), organic layer washed with saturated brine (30 mL), dried, filtered, volatile components evaporated *in vacuo* and product isolated by column chromatography on silica (EtOAc/*n*-hex = 1:5). Yield: 77 mg (54%) of yellow solid. ^{1}H NMR (400 MHz, $CDCl_3$) δ 8.38 (dd, *J* = 9.1, 0.6 Hz, 1H), 8.52 (dd, *J* = 9.1, 2.2 Hz, 1H), 8.96 (dd, *J* = 2.2, 0.6 Hz, 1H). ^{13}C NMR (101 MHz, $CDCl_3$) δ 112.13, 118.69, 123.22, 126.18, 135.70, 141.89, 147.36, 155.43. ESI-HRMS ($[M+H]^+$, m/z): Calcd for $C_8H_4O_2N_3S$ 206.00187, found 206.00138. IR (cm^{-1}): 2237 (CN group). Purity: UHPLC (Method III, 254 nm): tr = 4.597 min, 98.3% total area.

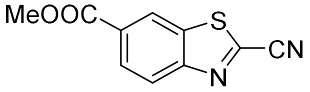

Methyl 2-cyanobenzo[*d*]thiazole-6-carboxylate (**69**) was prepared following *GP2* from methyl 4-amino-3-bromobenzoate (440 mg, 1.91 mmol), and isolated by column chromatography on silica (EtOAc/*n*-hex = 1:3). Yield: 140 mg (34%) of yellow solid. ^{1}H NMR (400 MHz, DMSO-*d*$_6$) δ 3.93 (s, 3H), 8.22 (dd, *J* = 8.7, 1.7 Hz, 1H), 8.37 (dd, *J* = 8.7, 0.5 Hz, 1H), 9.05 (dd, *J* = 1.7, 0.5 Hz, 1H). ^{13}C NMR (101 MHz, DMSO-*d*$_6$) δ 52.81, 113.24, 124.78, 125.55, 128.29, 129.24, 135.81, 141.14, 154.19, 165.52. ESI-HRMS ($[M+H]^+$, m/z): Calcd for $C_{10}H_7O_2N_2S$ 219.02227, found 219.02183. IR (cm^{-1}): 2234 (CN group). Purity: UHPLC (Method III, 254 nm): tr = 4.733 min, 95.0% total area.

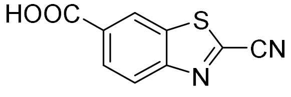

2-Cyanobenzo[*d*]thiazole-6-carboxylic acid (**70**) was prepared following *GP5* from methyl 2-cyanobenzo[*d*]thiazole-6-carboxylate (119 mg, 0.55 mmol), and isolated by column chromatography on silica (EtOAc/*n*-hex = 1:3). Yield: 39 mg (35%) of brown solid. ^{1}H NMR (400 MHz, DMSO-*d*$_6$) δ 8.19 – 8.27 (m, 2H), 8.88 (s, 1H). ^{13}C NMR (101 MHz, DMSO-*d*$_6$) δ 113.83, 124.29, 124.92, 129.35, 135.78, 135.82, 139.48, 153.53, 168.42. ESI-HRMS ($[M+H]^+$,

m/z): Calcd for $C_9H_5O_2N_2S$ 205.00662, found 205.00625. IR (cm^{-1}): 2237 (CN group). Purity: UHPLC (Method III, 254 nm): tr = 4.157 min, 98.0% total area.

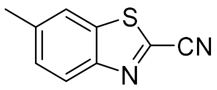

6-Methylbenzo[*d*]thiazole-2-carbonitrile (**72**) was prepared following *GP1* from 2-amino-5-methylbenzenethiol (400 mg, 2.87 mmol), and isolated by column chromatography on silica (EtOAc/*n*-hex = 1:6) and RP-CC (*GP7*). Yield: 20 mg (4%) of white solid. ^{1}H NMR (400 MHz, $CDCl_3$) δ 2.55 (s, 3H), 7.46 (dd, *J* = 8.6, 1.6 Hz, 1H), 7.76 (p, *J* = 0.8 Hz, 2H), 8.09 (d, *J* = 8.5 Hz, 1H). ^{13}C NMR (101 MHz, $CDCl_3$) δ 21.98, 113.30, 121.35, 124.84, 129.94, 135.42, 135.81, 139.68, 150.64. ESI-HRMS ($[M+H]^+$, m/z): Calcd for $C_9H_7N_2S$ 175.03245, found 175.03202. IR (cm^{-1}): 2229 (CN group). Purity: UHPLC (Method III, 254 nm): tr = 4.923 min, 97.2% total area.

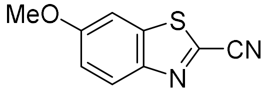

6-Methoxybenzo[*d*]thiazole-2-carbonitrile (**73**). Commercially available. ^{1}H NMR (400 MHz, DMSO-d_6) δ 3.88 (s, 3H), 7.32 (dd, *J* = 9.1, 2.6 Hz, 1H), 7.89 (d, *J* = 2.6 Hz, 1H), 8.14 (d, *J* = 9.1 Hz, 1H). HRMS (ESI^+) *m/z* $[M+H]^+$, calcd. for $C_9H_7N_2OS$ 191.02736, found 191.02702. Purity: UHPLC (Method I, 254 nm): tr = 4.680 min, 98.5% total area.

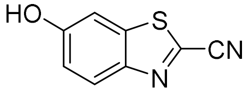

6-Hydroxybenzo[*d*]thiazole-2-carbonitrile (**74**). Commercially available. ^{1}H NMR (400 MHz, DMSO-d_6) δ 7.18 (dd, *J* = 9.0, 2.4 Hz, 1H), 7.59 (d, *J* = 2.4 Hz, 1H), 8.06 (d, *J* = 9.0 Hz, 1H), 10.53 (s, 1H). HRMS (ESI^+) *m/z* $[M+H]^+$, calcd. for $C_8H_5N_2OS$ 177.01171, found 177.01157. Purity: UHPLC (Method I, 254 nm): tr = 4.060 min, 99.6% total area.

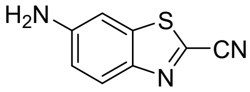

6-Aminobenzo[*d*]thiazole-2-carbonitrile (**75**) was prepared following *GP4* from 6-nitrobenzo[*d*]thiazole-2-carbonitrile (300 mg, 1.45 mmol), and isolated by column

chromatography on silica (EtOAc/*n*-hex = 1:1). Yield: 86 mg (33%) of yellow solid. ^{1}H NMR (400 MHz, DMSO-d_6) δ 6.97 (dd, *J* = 9.0, 2.3 Hz, 1H), 7.15 (d, *J* = 2.2 Hz, 1H), 7.86 (d, *J* = 8.9 Hz, 1H). ^{13}C NMR (101 MHz, DMSO-d_6) δ 102.26, 114.32, 117.38, 125.12, 127.80, 138.39, 143.23, 150.55. ESI-HRMS ([M+H]$^+$, m/z): Calcd for $C_8H_6N_3S$ 176.02769 found 176.02726. IR (cm^{-1}): 2228 (CN group). Purity: UHPLC (Method III, 254 nm): tr = 3.533 min, 99.0% total area.

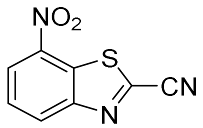

7-Nitrobenzo[*d*]thiazole-2-carbonitrile (**76**). To a suspension of 2-chloro-7-nitrobenzo[*d*]thiazole (150 mg, 0.70 mmol) in MeCN (10 mL), DABCO (12 mg, 0.11 mmol) was added, followed by a solution of NaCN (41 mg, 0.84 mmol) in H_2O (1 mL). After stirring the reaction mixture at rt for 7 h, a solution of $FeCl_{3(aq)}$ (10 mL) was added, extracted with EtOAc (3 × 20 mL), organic layer washed with saturated brine (30 mL), dried, filtered, volatile components evaporated *in vacuo* and product isolated by column chromatography on silica (EtOAc/*n*-hex = 1:3). Yield: 102 mg (71%) of white solid. ^{1}H NMR (400 MHz, Acetone-d_6) δ 8.07 (t, *J* = 8.1 Hz, 1H), 8.69 (dd, *J* = 8.2, 0.9 Hz, 1H), 8.74 (dd, *J* = 8.1, 0.9 Hz, 1H). ^{13}C NMR (101 MHz, Acetone-d_6) δ 113.25, 125.75, 129.56, 130.92, 132.73, 142.76, 143.63, 154.65. ESI-HRMS ([M+H]$^+$, m/z): Calcd for $C_8H_4O_2N_3S$ 206.00187, found 206.00142. IR (cm^{-1}): 2241 (CN group). Purity: UHPLC (Method III, 254 nm): tr = 4.667 min, 99.0% total area.

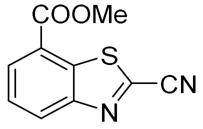

Methyl 2-cyanobenzo[*d*]thiazole-7-carboxylate (**77**) was prepared following *GP2* from methyl 3-amino-2-bromobenzoate (200 mg, 0.87 mmol), and isolated by column chromatography on silica (EtOAc/*n*-hex = 1:4). Yield: 7 mg (4%) of yellow solid. ^{1}H NMR (400 MHz, $CDCl_3$) δ 4.07 (s, 3H), 7.76 (dd, *J* = 8.2, 7.5 Hz, 1H), 8.34 (dd, *J* = 7.6, 1.1 Hz, 1H), 8.43 (dd, *J* = 8.2, 1.1 Hz, 1H).. ^{13}C NMR (101 MHz, $CDCl_3$) δ 53.30, 113.13, 124.78, 127.88, 130.04, 130.14, 135.70, 141.04, 153.25, 165.58. ESI-HRMS ([M+H]$^+$, m/z): Calcd for $C_{10}H_7O_2N_2S$ 219.02227, found 219.02171. IR (cm^{-1}): 2231 (CN group). Purity: UHPLC (Method III, 254 nm): tr = 4.850 min, 99.0% total area.

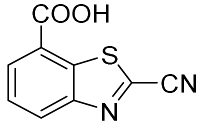

2-Cyanobenzo[*d*]thiazole-7-carboxylic acid (**78**) was prepared following *GP5* from methyl 2-cyanobenzo[*d*]thiazole-7-carboxylate (180 mg, 0.82 mmol), and isolated by column chromatography on silica (EtOAc/*n*-hex = 1:2). Yield: 85 mg (50%) of brown solid. ^{1}H NMR (400 MHz, DMSO-d_6) δ 7.74 – 7.80 (m, 1H), 8.20 (d, *J* = 7.4 Hz, 1H), 8.32 (d, *J* = 8.1 Hz, 1H). ^{13}C NMR (101 MHz, DMSO-d_6) δ 113.97, 126.66, 127.94, 128.73, 135.07, 141.05, 151.89, 166.51. ESI-HRMS ([M+H]$^+$, m/z): Calcd for $C_9H_5O_2N_2S$ 205.00615, found 205.00662. IR (cm^{-1}): 2237 (CN group). Purity: UHPLC (Method III, 254 nm): tr = 4.283 min, 97.4% total area.

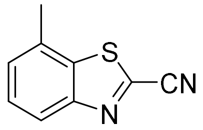

7-Methylbenzo[*d*]thiazole-2-carbonitrile (**80**) was prepared following *GP2* from 2-bromo-3-methylaniline (300 mg, 1.61 mmol), and isolated by column chromatography on silica (EtOAc/*n*-hex = 1:3). Yield: 40 mg (14%) of yellow solid. ^{1}H NMR (400 MHz, $CDCl_3$) δ 2.63 (s, 3H), 7.42 (dt, *J* = 7.3, 0.9 Hz, 1H), 7.55 – 7.59 (m, 1H), 8.06 (d, *J* = 8.3 Hz, 1H). ^{13}C NMR (101 MHz, $CDCl_3$) δ 21.48, 113.26, 122.85, 128.30, 128.68, 132.25, 136.13, 136.26, 152.35. ESI-HRMS ([M+H]$^+$, m/z): Calcd for $C_9H_7N_2S$ 175.03245, found 175.03202. IR (cm^{-1}): 2229 (CN group). Purity: UHPLC (Method III, 254 nm): tr = 4.947 min, 95.0% total area.

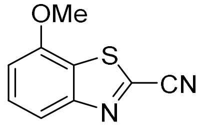

7-Methoxybenzo[*d*]thiazole-2-carbonitrile (**81**) was prepared following *GP2* from 2-bromo-3-methoxyaniline (300 mg, 1.48 mmol), and isolated by column chromatography on silica (EtOAc/*n*-hex = 1:3). Yield: 167 mg (59%) of yellow solid. ^{1}H NMR (400 MHz, $CDCl_3$) δ 4.03 (s, 3H), 7.01 (d, *J* = 8.0 Hz, 1H), 7.59 (t, *J* = 8.2 Hz, 1H), 7.82 (dd, *J* = 8.3, 0.6 Hz, 1H). ^{13}C NMR (101 MHz, $CDCl_3$) δ 56.36, 107.74, 113.21, 117.56, 124.89, 129.11, 137.24, 153.94, 154.30. ESI-HRMS ([M+H]$^+$, m/z): Calcd for $C_9H_7ON_2S$ 191.02736 found 191.02672. IR (cm^{-1}): 2233 (CN group). Purity: UHPLC (Method III, 254 nm): tr = 4.887 min, 95.8% total area.

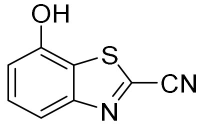

7-Hydroxybenzo[*d*]thiazole-2-carbonitrile (**82**) was prepared following *GP6* from 7-methoxybenzo[*d*]thiazole-2-carbonitrile (50 mg, 0.26 mmol), and isolated by column chromatography on silica (EtOAc/hex = 1:6). Yield: 37 mg (80%) of yellow solid. ^{1}H NMR (400 MHz, $CDCl_3$) δ 5.80 (br s, 1H), 6.99 (dd, *J* = 7.8, 0.6 Hz, 1H), 7.51 (t, *J* = 8.1 Hz, 1H), 7.84 (dd, *J* = 8.3, 0.6 Hz, 1H). ^{13}C NMR (101 MHz, $CDCl_3$) δ 112.49, 113.14, 117.87, 123.73, 129.01, 137.30, 150.50, 154.44. ESI-HRMS ($[M+H]^+$, m/z): Calcd for $C_8H_5ON_2S$ 177.01171, found 177.01121. IR (cm^{-1}): 2223 (CN group). Purity: UHPLC (Method III, 254 nm): tr = 4.317 min, 99.1% total area.

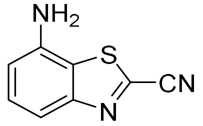

7-Aminobenzo[*d*]thiazole-2-carbonitrile (**83**). 2-Chloro-7-aminobenzo[*d*]thiazole (**124**) was prepared following *GP4* from 2-chloro-7-nitrobenzo[*d*]thiazole (1.0 g, 4.66 mmol), and isolated by crystallization (Et_2O). Yield: 860 mg (quantitative) of yellow solid. ^{1}H NMR (400 MHz, DMSO-d_6) δ 5.81 (s, 2H), 6.68 (d, *J* = 7.8 Hz, 1H), 7.17 (d, *J* = 7.8 Hz, 1H), 7.25 (t, *J* = 7.9 Hz, 1H).

To a solution of 2-chloro-7-aminobenzo[*d*]thiazole (247 mg, 1.15 mmol) in anhydrous DMF (5 mL), DABCO (12 mg, 0.11 mmol) was added. The reaction mixture was cooled on icebath, and KCN (90 mg, 1.38 mmol) was added. After stirring the reaction mixture at 100 °C for 48 h, a solution of $FeCl_{3(aq)}$ (10 mL) was added, extracted with EtOAc (3 × 20 mL), organic layer washed with saturated brine (30 mL), dried, filtered, volatile components evaporated *in vacuo*, and product isolated by column chromatography on silica (EtOAc/*n*-hex = 1:1). Yield: 10 mg (5%) of yellow solid. ^{1}H NMR (400 MHz, $CDCl_3$) δ 4.02 (br s, 2H), 6.91 (dd, *J* = 7.7, 0.6 Hz, 2H), 7.47 (t, *J* = 8.0 Hz, 1H), 7.71 (dd, *J* = 8.2, 0.6 Hz, 1H). ^{13}C NMR (101 MHz, $CDCl_3$) δ 113.08, 113.19, 116.04, 122.68, 129.19, 135.43, 140.88, 153.74. ESI-HRMS ($[M+H]^+$, m/z): Calcd for $C_8H_6N_3S$ 176.02769, found 176.02724. IR (cm^{-1}): 2241.25 (CN group). Purity: UHPLC (Method III, 254 nm): tr = 4.117 min, 98.3% total area.

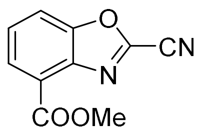

Methyl 2-cyanobenzo[*d*]oxazole-4-carboxylate (**86**) was prepared following *GP1* from methyl 2-amino-3-hydroxybenzoate (1.0 g, 6.00 mmol), and isolated by column chromatography on silica (EtOAc/*n*-hex = 1:3). Yield: 146 mg (10%) of off-white crystals. ^{1}H NMR (400 MHz, $CDCl_3$) δ 3.99 (s, 3H), 7.64 (dd, *J* = 8.4, 7.7 Hz, 1H), 7.84 (dd, *J* = 8.4, 1.1 Hz, 1H), 8.12 (dd, *J* = 7.7, 1.1 Hz, 1H). ^{13}C NMR (101 MHz, $CDCl_3$) δ 52.75, 108.69, 115.91, 123.93, 128.62, 129.04, 138.23, 138.45, 150.76, 164.29. ESI-HRMS ($[M+H]^+$, m/z): Calcd for $C_{10}H_7O_3N_2$ 203.04512, found 203.04481. IR (cm^{-1}): 2252 (CN group). Purity: UHPLC (Method I, 254 nm): tr = 4.317 min, 96.6% total area.

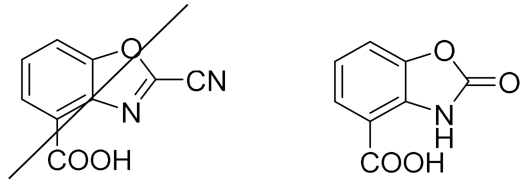

Synthesis of 2-cyanobenzo[*d*]oxazole-4-carboxylic acid (**87**) following *GP5* from methyl 2-cyanobenzo[*d*]oxazole-5-carboxylate (110 mg, 0.54 mmol), and isolation by column chromatography on silica (DCM/MeOH= 9:1) and subsequent RP-CC (*GP7*) failed to produce the desired product. Yield: 20 mg (20%) of dark brown solid. ESI-HRMS ($[M–H]^-$, m/z): Calcd for $C_9H_3O_3N_2$ 187.01492, no clear mass peak found. IR (cm^{-1}): no clear peak for CN group. Purity: UHPLC (Method I, 254 nm): mixture of two major compounds tr = 3.583 and 3.683 min, 81.2 % total area. Alternative strategies, i.e. using trimethylsilyl iodide [4], sodium *tert*-butoxide in THF [1], or LiOH catalyzed hydrolysis of ester, resulted in complete degradation of starting methyl 2-cyanobenzo[*d*]oxazole-5-carboxylate (using trimethylsilyl iodide) or formation of 2-oxo-2,3-dihydrobenzo[*d*]oxazole-4-carboxylic acid (**125)** (sodium *tert*-butoxide in THF and LiOH catalyzed hydrolysis of ester). ^{1}H NMR (400 MHz, DMSO-d_6) δ 7.15 (t, *J* = 8.0 Hz, 1H), 7.50 (dd, *J* = 8.0, 1.1 Hz, 1H), 7.61 (dd, *J* = 8.1, 1.1 Hz, 1H), 11.64 (s, 1H), 13.38 (br s, 1H). ^{13}C NMR (101 MHz, DMSO-d_6) δ 113.41, 113.61, 121.31, 124.43, 131.43, 143.84, 154.24, 165.60. ESI-HRMS ($[M–H]^-$, m/z): Calcd for $C_8H_4O_4N$ 178.01348, found 178.01364. IR (cm^{-1}): no signal for CN group, signals at 1760, 1678, 1630.

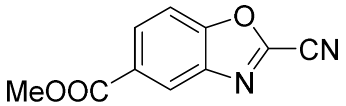

Methyl 2-cyanobenzo[*d*]oxazole-5-carboxylate (**94**) was prepared following *GP1* from methyl 3-amino-4-hydroxybenzoate (3.0 g, 17.9 mmol), and isolated by column chromatography on silica (EtOAc/*n*-hex = 1:3). Yield: 617 mg (17%) of brown crystals. ^{1}H NMR (400 MHz, $CDCl_3$) δ 3.96 (s, 3H), 7.69 (d, *J* = 8.8 Hz, 1H), 8.29 (dd, *J* = 8.8, 1.7 Hz, 1H), 8.53 (d, *J* = 1.7 Hz, 1H). ^{13}C NMR (101 MHz, $CDCl_3$) δ 52.79, 108.73, 111.60, 124.10, 129.21, 130.60, 138.49, 139.58, 152.95, 165.70. ESI-HRMS ($[M+H]^+$, m/z): Calcd for $C_{10}H_7O_3N_2$ 203.04512, found 203.04491. IR (cm^{-1}): 2254 (CN group). Purity: UHPLC (Method I, 254 nm): tr = 4.623 min, 95.3% total area.

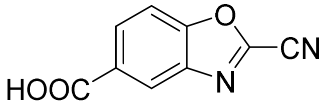

2-Cyanobenzo[*d*]oxazole-5-carboxylic acid (**95**) was prepared following *GP5* from methyl 2-cyanobenzo[*d*]oxazole-5-carboxylate (100 mg, 0.50 mmol), and isolated by column chromatography on silica (DCM/MeOH= 9:1) and subsequent RP-CC (*GP7*). Yield: 24 mg (26%) of yellow-orange crystals. ^{1}H NMR (400 MHz, CD_3OD) δ 7.85 (dd, *J* = 8.8, 0.7 Hz, 1H), 8.31 (dd, *J* = 8.7, 1.6 Hz, 1H), 8.51 (dd, *J* = 1.7, 0.6 Hz, 1H). ^{13}C NMR (101 MHz, CD_3OD) δ 109.95, 112.71, 124.65, 131.08, 131.66, 140.15, 140.81, 154.41, 168.25. ESI-HRMS ($[M–H]^-$, m/z): Calcd for $C_9H_3O_3N_2$ 187.01492, found 187.01432. IR (cm^{-1}): 2255 (CN group). Purity: UHPLC (Method I, 254 nm): tr = 4.040 min, 96.5% total area.

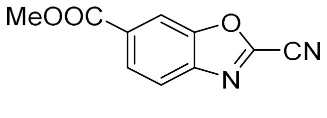

Methyl 2-cyanobenzo[*d*]oxazole-6-carboxylate (**102**) was prepared following *GP1* from methyl 4-amino-3-hydroxybenzoate (5.0 g, 29.9 mmol), and isolated by column chromatography on silica (EtOAc/*n*-hex = 1:3). Yield: 1.81 g (30%) of orange crystals. ^{1}H NMR (400 MHz, $CDCl_3$) δ 3.98 (s, 3H), 7.91 (dd, *J* = 8.5, 0.7 Hz, 1H), 8.20 (dd, *J* = 8.5, 1.5 Hz, 1H), 8.31 (dd, *J* = 1.5, 0.7 Hz, 1H). ^{13}C NMR (101 MHz, $CDCl_3$) δ 52.92, 108.77, 113.35, 121.84, 127.86, 131.14, 139.41, 142.84, 150.10, 165.64. ESI-HRMS ($[M+H]^+$, m/z): Calcd for $C_{10}H_7O_3N_2$ 203.04512, found 203.04483. IR (cm^{-1}): 2257 (CN group). Purity: UHPLC (Method I, 254 nm): tr = 4.650 min, 97.5% total area.

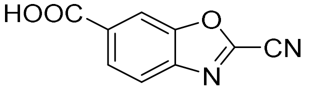

2-Cyanobenzo[*d*]oxazole-6-carboxylic acid (**103**) was prepared following *GP5* from methyl 2-cyanobenzo[*d*]oxazole-6-carboxylate (200 mg, 0.99 mmol), and isolated by column chromatography on silica (DCM/MeOH= 9:1) and subsequent RP-CC (*GP7*). Yield: 65 mg (35%) of white crystals. ^{1}H NMR (400 MHz, CD_3OD) δ 7.92 – 7.98 (m, 1H), 8.15 – 8.23 (m, 1H), 8.32 – 8.38 (m, 1H). ^{13}C NMR (101 MHz, CD_3OD) δ 109.96, 114.31, 122.50, 128.82, 133.03, 141.00, 143.97, 151.54, 168.04. ESI-HRMS ($[M–H]^-$, m/z): Calcd for $C_9H_3O_3N_2$ 187.01492, found 187.01435. IR (cm^{-1}): 2255 (CN group). Purity: UHPLC (Method I, 254 nm): tr = 4.067 min, 99.8% total area.

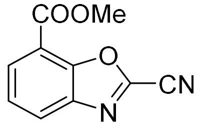

Methyl 2-cyanobenzo[*d*]oxazole-7-carboxylate (**110**) was prepared following *GP1* from methyl 3-amino-2-hydroxybenzoate (1.0 g, 6.00 mmol), and isolated by column chromatography on silica (EtOAc/*n*-hex = 1:3) and subsequent RP-CC (*GP7*). Yield: 315 mg (26%) of brown solid. ^{1}H NMR (400 MHz, $CDCl_3$) δ 4.03 (s, 3H), 7.59 (t, *J* = 7.9 Hz, 1H), 8.07 (dd, *J* = 8.2, 1.2 Hz, 1H), 8.22 (dd, *J* = 7.7, 1.2 Hz, 1H). ^{13}C NMR (101 MHz, $CDCl_3$) δ 52.90, 108.86, 116.25, 126.47, 126.78, 131.30, 138.36, 140.77, 149.04, 163.45. ESI-HRMS ($[M+H]^+$, m/z): Calcd for $C_{10}H_7O_3N_2$ 203.04512, found 203.04494. IR (cm^{-1}): 2255 (CN group). Purity: UHPLC (Method I, 254 nm): tr = 4.540 min, 97.2% total area.

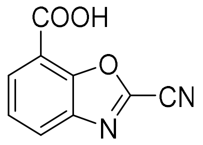

2-Cyanobenzo[*d*]oxazole-7-carboxylic acid (**111**) was prepared following *GP5* from methyl 2-cyanobenzo[*d*]oxazole-7-carboxylate (200 mg, 0.99 mmol), and isolated by column chromatography on silica (DCM/MeOH= 9:1) and subsequent RP-CC (*GP7*). Yield: 65 mg (35%) of yellow-orange crystals. ^{1}H NMR (400 MHz, DMSO-*d*$_6$) δ 7.70 (t, *J* = 7.9 Hz, 1H), 8.17 (dd, *J* = 7.7, 1.2 Hz, 1H), 8.26 (dd, *J* = 8.1, 1.2 Hz, 1H), 13.81 (br s, 1H). ^{13}C NMR (101 MHz, DMSO-*d*$_6$) δ 109.67, 116.69, 126.34, 126.60, 130.83, 138.31, 140.25, 148.64, 163.87.

ESI-HRMS ([M–H]$^{-}$, m/z): Calcd for $C_9H_3O_3N_2$ 187.01492, found 187.01433. IR (cm^{-1}): 2256 (CN group). Purity: UHPLC (Method I, 254 nm): tr = 3.883 min, 95.3% total area.

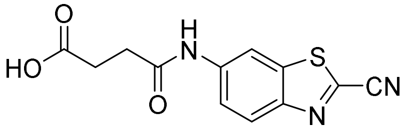

4-((2-Cyanobenzo[*d*]thiazol-6-yl)amino)-4-oxobutanoic acid (**117**) was synthesized by reacting 6-aminobenzo[*d*]thiazole-2-carbonitrile (25 mg, 0.143 mmol) with succinic anhydride (57 mg, 0.571 mmol) and *N*-methylmorpholine (79 µL, 0.715 mmol) dissolved in THF (6 mL) at 80 °C for 24 h (pressure tube). Volatile components were evaporated *in vacuo*, and the product isolated by RP-CC (*GP7*) with H_2O replacing 0.1% $TFA_{(aq)}$ as the aqueous component of the mobile phase. Fractions containing the product were combined and organic volatiles were evaporated *in vacuo*. The precipitate formed was collected by filtration and dried at 50 °C for 18 h. Yield: 30 mg (76%) of white solid. ^{1}H NMR (400 MHz, DMSO-*d$_6$*) δ 2.53 – 2.59 (m, 2H), 2.64 (t, *J* = 6.6 Hz, 2H), 7.72 (dd, *J* = 9.0, 2.1 Hz, 1H), 8.18 (d, *J* = 9.0 Hz, 1H), 8.74 (d, *J* = 2.0 Hz, 1H), 10.49 (s, 1H), 12.18 (s, 1H). ^{13}C NMR (101 MHz, DMSO-*d$_6$*) δ 28.67, 31.19, 110.89, 113.62, 120.53, 124.78, 134.74, 136.77, 139.78, 147.41, 170.85, 173.76. ESI-HRMS ([M–H]$^{-}$, m/z): Calcd for $C_{12}H_8O_3N_3S$ 274.05919, found 274.02960. IR (cm^{-1}): 2230 (CN group).

6 Supporting References

1. Yang, H.S.; Macha, L.; Ha, H.-J.; Yang, J.W. Functionalisation of Esters via 1,3-Chelation Using NaOtBu: Mechanistic Investigations and Synthetic Applications. *Org. Chem. Front.* **2021**, *8*, 53–60, doi:10.1039/D0QO01135E.
2. Ren, H.; Xiao, F.; Zhan, K.; Kim, Y.-P.; Xie, H.; Xia, Z.; Rao, J. A Biocompatible Condensation Reaction for the Labeling of Terminal Cysteine Residues on Proteins. *Angew. Chem. Int. Ed.* **2009**, *48*, 9658–9662, doi:10.1002/anie.200903627.
3. Chen, Z.; Chen, M.; Cheng, Y.; Kowada, T.; Xie, J.; Zheng, X.; Rao, J. Exploring the Condensation Reaction between Aromatic Nitriles and Amino Thiols To Optimize In Situ Nanoparticle Formation for the Imaging of Proteases and Glycosidases in Cells. *Angew. Chem. Int. Ed.* **2020**, *59*, 3272–3279, doi:10.1002/anie.201913314.
4. Structural Analysis and Optimization of NK1 Receptor Antagonists through Modulation of Atropisomer Interconversion Properties | Journal of Medicinal Chemistry Available online: https://pubs.acs.org/doi/10.1021/jm030197g (accessed on 2 February 2023).
